# Rational Design of a Synthetic Galliphore for the Diagnostic Nuclear Imaging and Treatment of Bacterial Infections

**DOI:** 10.1002/anie.9696366

**Published:** 2026-09-04

**Authors:** Phuong Nguyen Tran, Axia Marlin, Edith K. Amason, Marie Huynh, Hannah Carlisle, Eszter Boros

**Affiliations:** ^1^ Department of Chemistry University of Wisconsin Madison Madison Wisconsin USA

**Keywords:** bacterial metal homeostasis, imaging, infection, metal chelation

## Abstract

We introduce a solid‐phase synthesis platform for the rapid, modular, and scalable synthesis of the synthetic metallophore KO‐NH_2_, which is readily recognized by bacterial siderophore transporters, but has optimized affinity for Ga^3+^ over Fe^3+^. Four synthetic protochelin (PC) analogues, KK‐COOH, KO‐NH_2_, KO‐COOH, and OK‐COOH were constructed. Growth recovery, radioactive uptake, and covalent photocrosslinker studies in *Escherichia coli* identified CirA and Fiu as the dominant outer membrane transport proteins capable of binding KO‐NH_2_‐linked, bifunctional constructs. Computational studies further supported that the structural framework of PC can be effectively mimicked by KO or OK peptides. Thermodynamic stability studies revealed an atypical inversion of thermodynamic stability trends, with logK_GaL_ of 39.9 and a logK_FeL_ of 36, demonstrating improved stability with Ga^3+^. Studies in naïve mice with ^68^Ga‐labeled derivatives identify the lead [^68^Ga][Ga(KO‐NH_2_)]^3−^, which remained intact in vivo and demonstrated selective, enhanced uptake in an *E. coli* soft tissue model of infection (1.28 % ID/g, with a 3:1 ratio of infected to inflamed/normal muscle tissue). Linkage of KO‐NH_2_ to the antibiotic ampicillin produces an efficacious antibiotic, which supersedes the potency of the parent antibiotic ampicillin 4‐fold (MIC_98_ = 1.9 µM) effectively treats a soft tissue infection of ampicillin‐resistant *E. coli*.

## Graphical abstract

KO‐NH_2_ is a synthetic metallophore that preferentially binds Ga^3+^ over Fe^3+^ and is recognized by bacterial transporters. Photocrosslinking identifies CirA and Fiu as key uptake proteins *in E. coli*. KO‐NH_2_ enables targeted imaging and treatment of *E. coli* soft tissue infections. When linked to ampicillin, KO‐NH_2_‐Amp effectively treats ampicillin‐resistant, *E. coli* infections in mice.

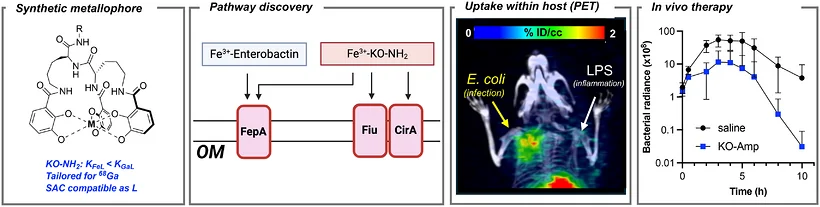

## 1 Introduction

The overuse of broad‐spectrum antibiotics has contributed to rising antibiotic resistance, particularly among Gram‐negative bacterial pathogens (GNB) [1, 2]. Among GNB, *Enterobacteriaceae* account for about 80% of Gram‐negative clinical isolates, causing pathologies such as urinary tract infections, pneumonia, diarrhea, meningitis, sepsis, and endotoxic shock. Rapidly evolving resistance mechanisms necessitate new therapeutic strategies that reduce the associated risk [3].

Bacterial virulence is closely associated with nutrient acquisition, especially iron, which is necessary for bacterial growth and metabolic functions [4]. In the host environment, iron availability is tightly regulated by the host proteins and becomes further restricted during infection as a defense mechanism to prevent pathogenic bacterial invasion [5, 6]. To overcome this, bacteria produce siderophores, small iron‐chelating molecules, that scavenge iron and are imported via active membrane transporters to support growth and pathogenicity [7, 8].

The siderophore uptake machinery is unique to bacteria, and absent in mammalian hosts, thus providing a selective, active uptake mechanism that can be taken advantage of for selective drug delivery [9]. Indeed, a natural product class termed sideromycins, siderophore‐antibiotic conjugates (SACs), such as albomycin and salmycin, can readily inhibit growth of bacterial strains that thrive in mammalian hosts [10, 11]. Over the past 2 decades, synthetic SACs have also been extensively explored: Potent apo‐(non‐metal containing) and pro‐drug versions of such SACs were introduced by Miller, Nolan, and Wenczewicz [12, 13, 14, 15]. More recently, the US Food and Drug Administration (FDA) approved cefiderocol (Fetroja), a bidentate catechol‐cephalosporin conjugate, for the treatment of Gram‐negative urinary tract infections, re‐invigorating the field of SAC‐drug discovery [16, 17, 18, 19].

Our work in this area has focused on the displacement of Fe^3+^ with Ga^3+^. Indeed, we have demonstrated that Ga^3+^ complexes can significantly potentiate growth inhibitory activity of corresponding SACs [20, 21, 22]. A Ga‐hydroxamate ciprofloxacin conjugate, Ga‐LDFC‐CIP, derived from the natural product albomycin, outperformed the parent antibiotic ciprofloxacin in inhibiting *Staphylococcus aureus* growth both in vitro and in vivo [23, 24, 25]. Our mechanism of action studies revealed, that Ga^3+^‐SACs sustain high expression levels of siderophore‐transmembrane transport proteins in bacteria, resulting in enhanced SAC uptake when compared to Fe^3+^ chelated analogues [26]. We have also demonstrated that radioactively labeled, synthetic siderophore mimics such as [^68^Ga][Ga(TREN‐CAM)]^3−^ can monitor the treatment response to cefiderocol with positron emission tomography (PET) [27].

A significant challenge with using Ga^3+^ as a Fe^3+^ analogue is the diminished thermodynamic stability and kinetic inertness of the Ga^3+^ complex analogues. Indeed, early work by Raymond has documented a 3‐4 order of magnitude discrepancy for the logβ_110_ values for several synthetic enterobactin analogues: TREN‐CAM, ME‐CAM, TPAM‐CAM affirm this trend (Figure 1) [28, 29, 30, 31, 32]. The origin of this discrepancy arises from the diminished covalency of the Ga‐L bonds and the increased charge density (smaller ionic radius) of Ga^3+^ [33, 34].

**FIGURE 1:**
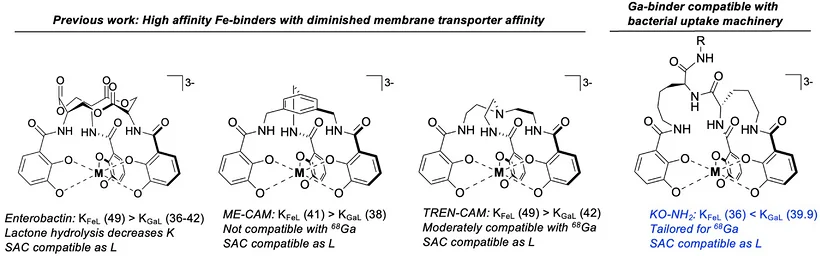
Chemical structures of the siderophore enterobactin and synthetic enterobactin mimics previously employed for the development of in vitro active siderophore antibiotic conjugates (SACs) and the peptidic, protochelin mimic KO‐NH_2_, subject of this work.

The development of a chelate which exhibits improved, or even superior thermodynamic stability with Ga^3+^, requires synthetic strategies that offer facile structural modifications combined with high‐throughput iteration. A recent report detailed the first structural characterization of [Fe(Ent)]^3−^ and protochelin (PC) bound to the PfeA siderophore transporter in *Pseudomonas aeruginosa*. The co‐crystal structure suggests that the PfeA transporter recognizes both [Fe(Ent)]^3−^ and [Fe(PC)]^3−^ [35, 36], indicating the ability to bind a spectrum of metal‐bound polycatecholates [37].

Here, we exploit a modular solid phase peptide synthesis (SPPS) assembled, [Fe(PC)]^3−^ mimicking polypeptide structures and their corresponding metal complexes, with the intention to identify structural entities with enhanced Ga^3+^ complex stability and retained recognition by bacterial transmembrane transport. The ability to modulate the length of the amino acid sidechain offers adjustment of the O─M─O coordinative angles around the metal center without perturbing interactions with target transmembrane transporters. This versatile SPPS strategy overcomes the synthetic limitations of Ent and TREN‐CAM, providing access to structure‐activity relationship studies with a library of siderophore mimics. Using bacterial growth recovery and uptake studies, we probe the impact of these small structural modifications to the chelate structure. Spectrophotometric and computational studies examine the impact on structure and thermodynamics of corresponding Fe^3+^ and Ga^3+^ complexes. We employed radiochemical studies with ^68^Ga to identify a lead chelator, suitable as a diagnostic agent, and examine if linkage of beta‐lactam antibiotics provides access to therapeutic agents to inhibit Gram‐negative *Escherichia coli* growth in vivo within soft tissue infections.

## 2 Results and Discussion

### 2.1 Solid‐Phase Synthesis of a Protochelin‐Inspired Siderophore Library

*Azotobacter vinelandii* secretes PC to scavenge iron from the host or neighbor organisms in iron‐deficient environments. PC is comprised of a lysine, linked to 1,4‐diaminobutane, capped with three catecholamides. We proposed to replace 1,4‐diaminobutane with ornithine or lysine, to render the construction of PC analogues with varying backbone chain lengths compatible with SPPS (Figure 2). We hypothesized that this strategy would enable control of the catechol tilt angle in direct dependence of amino acid side chain length. Change in the catechol tilt angle may alter the O─M─O angle, subsequently favoring Ga^3+^ coordination over Fe^3+^.

**FIGURE 2:**
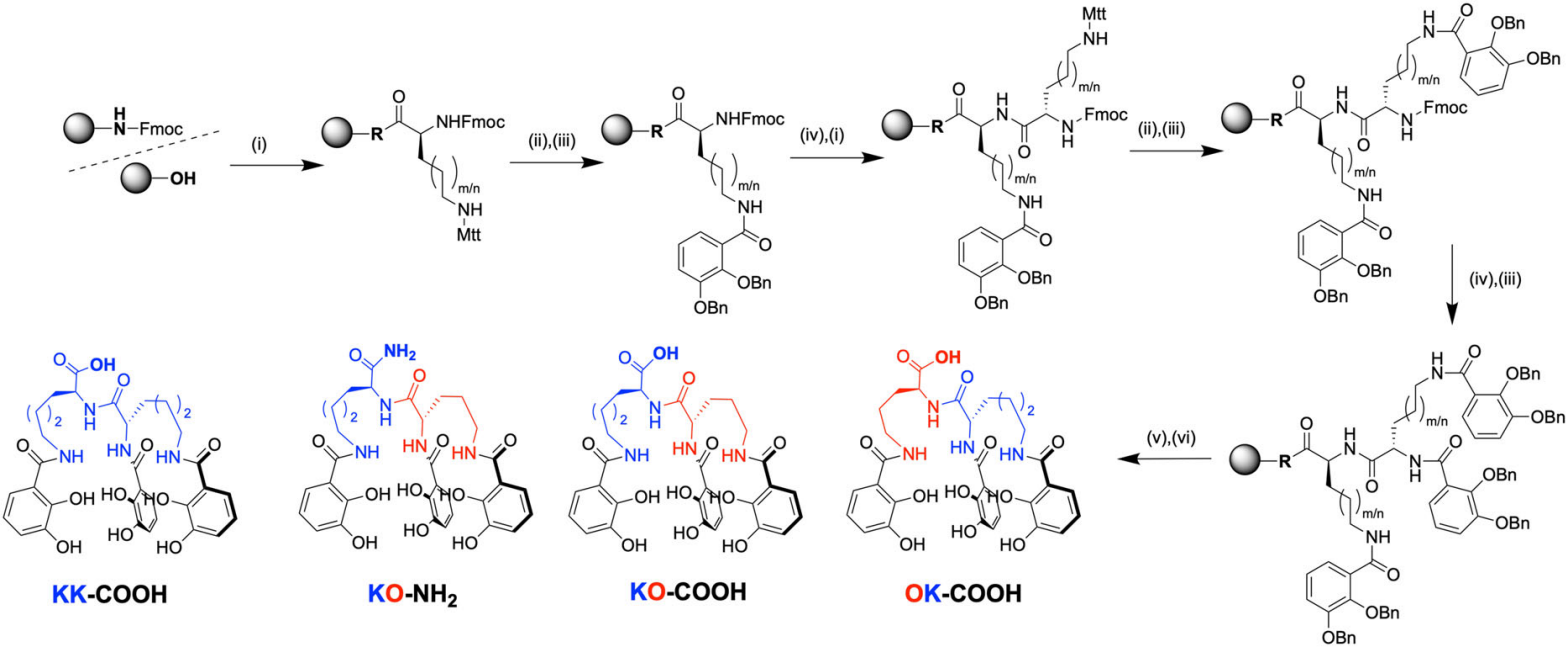
Protochelin peptide‐based solid phase synthesis to synthesize the 4 target analogues KK‐COOH, KO‐NH_2_, KO‐COOH, and OK‐COOH. (i) Fmoc‐Lys(Mtt)‐OH/Fmoc‐Orn(Mtt)‐OH, PyBOP, DIPEA. (ii) 1% TFA in DCM. (iii) Bzl‐CAM, PyBOP, DIPEA, (iv) 20% piperidine in DMF, (v) TFA/TIPS/H_2_O (18:1:1). (vi) Pd/C, H_2_, MeOH. Red: ornithine. Blue: lysine.

To establish the solid‐phase synthesis workflow, Wang and Rink amide resin were loaded with either Fmoc‐Lys‐4‐methyltrityl (Mtt) or Fmoc‐Orn‐(Mtt) (Figure 2). The Mtt protecting group was selected for its mild deprotection conditions, allowing orthogonal coupling of 2,3‐bis(benzyloxy)benzoic acid to the lysine or ornithine residues on resin. On‐resin synthesis proceeded through standard peptide elongation steps. Alternating Fmoc‐deprotection and Mtt deprotection were followed by amidation with 2,3‐bis(benzyloxy)benzoic acid to introduce O‐benzyl‐catechols. The fully assembled, orthogonally protected constructs were cleaved from the solid support, followed by debenzylation of the catechols by catalytic hydrogenation under atmospheric pressure [38, 39]. Reverse phase chromatography was employed to afford four synthetic PC analogues in their *apo*‐form: KK‐COOH, KO‐NH_2_, KO‐COOH, and OK‐COOH (Figure 2) with overall synthetic yields of 15%–20% (Scheme S1).

### 2.2 Metal Complexation and Computational Characterization

It is hypothesized Trojan horse antibiotic cefiderocol relies on local Fe^3+^ pools at the site of infection to translocate to the bacterial periplasm [40]; specifically, the formation of a 1:1 M:L complex is required for effective recognition of transmembrane transporters.

KK‐COOH, KO‐NH_2_, KO‐COOH, and OK‐COOH were employed to form complexes with Fe^3+^ and Ga^3+^. To this end, ligands were combined with ferric chloride in 20% dimethyl sulfoxide (DMSO) in water and formed the corresponding deep purple ferric complexes readily. Ga‐complexes were formed using Ga(acac)_3_ in methanol, yielding colorless complex solutions. Electrospray ionization mass spectrometry (ESI‐MS) identified the molecular peaks of mononuclear 1:1 ligand‐to‐metal complexes for all complexes.

Fe‐complexes have distinct UV–vis spectroscopic features in addition to a shift of the characteristic catechol ligand peak at 316 nm (*ε* = 9500 M^−1^ cm^−1^) upon metal coordination [41, 42]. Specifically, the ferric complexes show a characteristic ligand‐to‐metal charge transfer band (LMCT) at 550 nm of catecholate (π‐ligand‐based orbital) to Fe^3+^ (primarily 3d_z2_, d_x2‐dy2_) (Figures S55, S64, S73, and S82) [43]. ^1^H NMR spectra of Ga‐complexes were obtained and further evidence the formation of symmetric 1:1 complexes (Figures S58–S62, S67–S71, S76–S80, S85–S89).

In the absence of successful crystallographic analysis, we probed the structural parameters computationally. Specifically, we conducted DFT structure optimization of Fe^3+^ and Ga^3+^‐complexed PC‐based siderophores, and additionally modeled permutations that were not synthesized but are readily accessible (KK‐NH_2_ and OO‐COOH). For all complexes, calculations were performed with CAM‐B3LYP level of theory with the D3(BJ) dispersion and the def2‐TZVP basis set [44, 45, 46]. Furthermore, all complexes were modeled using the conductor‐like polarizable continuum model (CPCM) with water as the solvent to best represent synthetic and biological conditions [47, 48, 49].

A representative example for the [Fe(KO‐NH_2_)]^3−^ complex (blue) in direct overlay with the corresponding [Fe(PC)]^3−^ complex (grey) is shown in Figure 3. Comparison of average O─M─O angles and O─M bond lengths (Figure 3, Tables S1–S12) reveals several trends. Average M─O bond lengths cluster tightly around 2.05 Å for Fe^3+^ complexes but are shortened to 2 Å for the corresponding Ga^3+^ complexes. Accordingly, the shortened bond length results in a greater O─M─O bond angle (82° vs. 79° for Fe^3+^ complexes). This correlates well with experimental, crystallography‐derived structural data from literature [34, 50]. Modulation of the terminal functional group (carboxylate vs. amide) had no significant impact on the coordinative environment. These observations support our synthetic design strategy suggesting that lysine/ornithine side chain variation could bias the coordination environment toward geometries better suited for Ga^3+^ over Fe^3+^.

**FIGURE 3:**
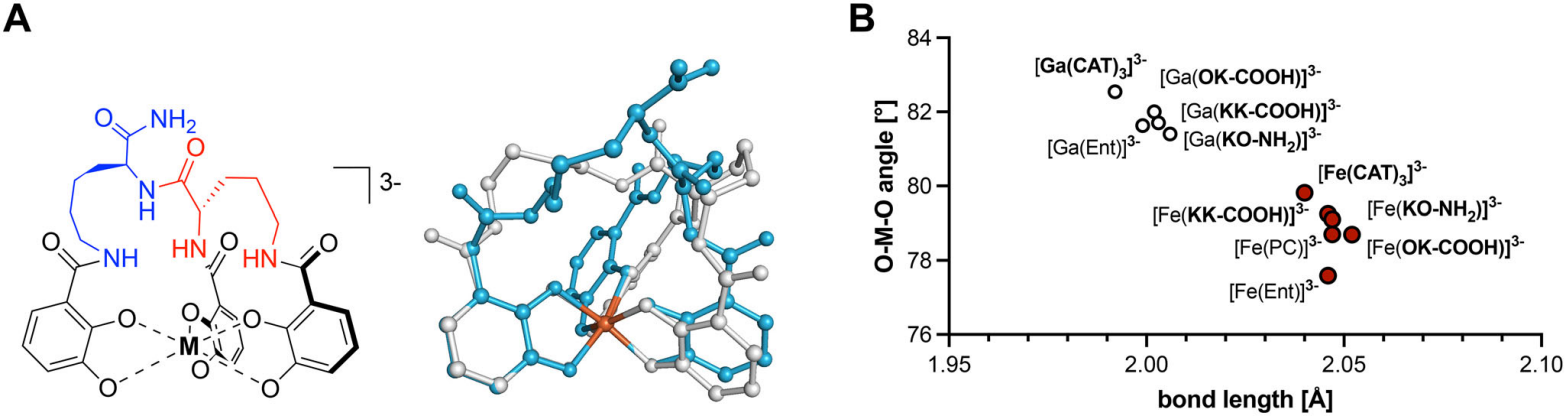
(A) Structure of KO‐NH_2_ metal complexes and DFT‐optimized structure of [Fe(KO‐NH_2_)]^3−^(M refers to Fe^3+^). (B) Plot of average M‐O bond length versus O‐M‐O angle of DFT‐computed, energy‐minimized complex structures.

### 2.3 Substrate Recognition and Utilization by *E. coli* Δent (Growth Recovery Assay)

To determine if the synthetic PC constructs supply iron via the *E. coli* uptake machinery, we employed a growth recovery experiment using *E. coli* Δent, which is defective in Ent biosynthesis but retains the transport machinery required to import and metabolize iron‐bound siderophores [51]. Specifically, this assay probes if a synthetic siderophore can provide metabolically accessible iron involving both transport via the FepABCD machinery and siderophore degradation to release iron, presumably by Fes. As a positive control, we supplemented cultures with increasing concentrations of the endogenous siderophore Ent. As anticipated, increasing amounts of Ent produced increased OD_600_ with log‐phase onset within 8–10 h (Figure 4). KO‐COOH and OK‐COOH both restored growth with log and stationary phases profiles like Ent, whereas KK‐COOH resulted in comparatively diminished growth. Finally, KO‐NH_2_ produced an extended lag phase and restored growth only at the highest ligand concentration tested (0.1 mM), indicating that it provided the least metabolically accessible Fe^3+^ resource among the analogues. To probe whether this reduced growth recovery also reflected diminished uptake efficiency or rather indicated impaired downstream Fe^3+^ utilization, we conducted quantitative, radioactive uptake experiments.

**FIGURE 4:**
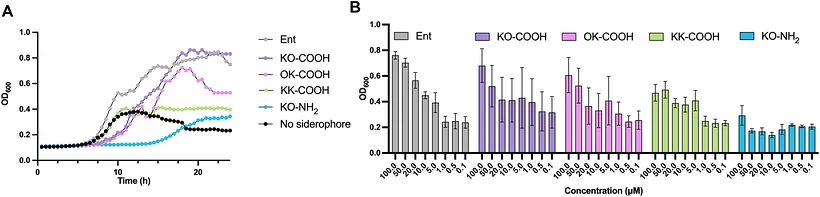
(A) Growth behavior of KK‐COOH, KO‐NH_2_, KO‐COOH, and OK‐COOH (0.1 mM) in *E. coli* Δent (ATCC 33475) compared to Ent for 24 h in iron‐depleted media. (B) Growth promotion of KK‐COOH, KO‐NH_2_, KO‐COOH, and OK‐COOH across siderophore concentrations by measuring OD_600_ at 18 h. Data is demonstrated as the average of three independent experiments (*n* = 9), and error bars are the standard deviation of the mean.

### 2.4 Radiochemical uptake experiments in *E. coli* K12 and *E. coli* ΔfepA/ΔfepC With ^67^Ga

Radioactive (tracer) uptake experiments are ideally suited to probe bacterial metabolism without disruption of protein expression due to downstream regulation; furthermore, we sought to identify chelators that can effectively image infection in vivo using the PET nuclide ^68^Ga (*t*_½_ = 1.1 h). In line with longer timepoints of interest for this assay, we employed the long‐lived radioisotope ^67^Ga (*t*_½_ = 79 h) as a surrogate [52].

We successfully formed the radiochemical complexes [^67^Ga][Ga(KK‐COOH)]^3−^, [^67^Ga][Ga(KO‐COOH)]^3−^, [^67^Ga][Ga(OK‐COOH)]^3−^, [^67^Ga][Ga(KO‐NH_2_)]^3−^, and [^67^Ga][Ga(Ent)]^3−^ under mild radiolabeling conditions (pH 7, 50 mM HEPES, 10 min, room temperature) with quantitative yields (Figures S156–S160) and 0.1 mCi/µmol molar activity.

We first tested uptake in *wt E. coli*. Among all synthetic siderophores tested, [^67^Ga][Ga(KK‐COOH)]^3−^ and [^67^Ga][Ga(KO‐NH_2_)]^3−^ showed efficient uptake (37% and 31%, respectively at the 2‐h time point), compared to 47% for [^67^Ga][Ga(Ent)]^3−^. [^67^Ga][Ga(KO‐COOH)]^3−^ and [^67^Ga][Ga(OK‐COOH)]^3−^ uptake was lower, reaching 18% and 20%, respectively, after 2 h of incubation (Figure 5). To probe selective uptake via the Ent‐mediated uptake pathway, we measured uptake following preincubation of excess [Fe(Ent)]^3−^ [23]. [Fe(Ent)]^3−^ repression resulted in a statistically significant decrease in ^67^Ga‐complexes uptake within the bacterial pellet (Figure 5). Notably, under these conditions the [^67^Ga][Ga(Ent)]^3−^ uptake is fully blocked, whereas uptake of the synthetic PC analogues is repressed, but not fully muted, indicating that these Ga‐chelates may efficiently utilize outer membrane transporters (Fiu, CirA). We were able to further affirm this hypothesis by quantification of uptake in *E. coli* mutant strains (ΔfepA) and (ΔfepC) [51, 53, 54]. These mutant strains were generated by kanamycin gene insertion in the fepA/fepC gene cluster, which represents the primary uptake pathway of [Fe(Ent)]^3−^ [55, 56]. The FepA receptor is selective and recognizes the iron‐binding domain and amide linkage domains of [Fe(Ent)]^3−^. FepB delivers [Fe(Ent)]^3−^ to the cytoplasmic pores formed by FepD and FepG, powered by a cytoplasmic ATPase, FepC [57]. Prior literature characterizes FepA, Fiu, and CirA as transporters of N‐(2,3‐dihyroxybenzoyl) serine (DHBS), a hydrolyzed [Fe(Ent)]^3−^ species, and other synthetic analogues [58].

**FIGURE 5:**
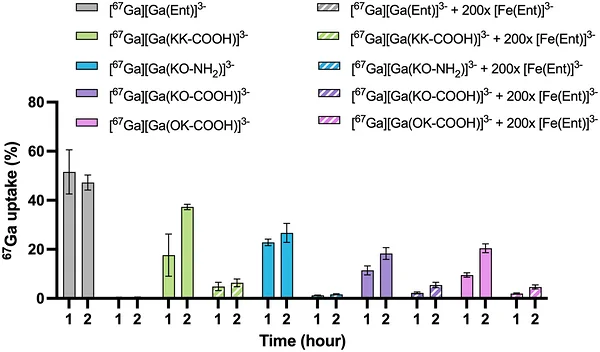
Time‐dependent, radiochemical bacterial uptake studies in *E. coli K12* of [^67^Ga][Ga(KK‐COOH)]^3−^, [^67^Ga][Ga(KO‐NH_2_)]^3−^, [^67^Ga][Ga(KO‐COOH)]^3−^, and [^67^Ga][Ga(OK‐COOH)]^3−^ in iron‐depleted, DP‐treated media pH 7.4 at 1 and 2 h incubation, with or without excess [Fe(Ent)]^3−^.

Indeed, [^67^Ga][Ga(Ent)]^3−^ uptake was suppressed (< 2%) in *E. coli* ΔfepA, whereas uptake with synthetic [^67^Ga][Ga(KK‐COOH)]^3−^, [^67^Ga][Ga(KO‐NH_2_)]^3−^, [^67^Ga][Ga(KO‐COOH)]^3−^, and [^67^Ga][Ga(OK‐COOH)]^3−^ were diminished, but remained significant when compared to [^67^Ga][Ga(Ent)]^3−^ (5%, 5%, 4%, and 5%, respectively, (Figure S152, Table S23), underscoring the possibility that these analogues are also imported by Fiu and CirA. Experiments with *E. coli* ΔfepC resulted in no uptake for any chelate, indicating that all derivatives rely on FepC for translocation to the cytoplasm of *E. coli* (Figure S153 and Table S24).

Conclusively, [^67^Ga][Ga(KK‐COOH)]^3−^ and [^67^Ga][Ga(KO‐NH_2_)]^3−^ emerged as lead complexes for further investigation. Their efficient uptake, paired with moderate regrowth (indicating poor Fe^3+^ mobilization intracellularly, potentially due to incompatibility with Fes‐mediated degradation), renders them ideal for further development.

### 2.5 Radiolabeling and In Vivo Biodistribution With ^68^Ga

After identification of suitable leads in vitro, we next assessed the pharmacokinetics and metabolic stability in vivo. To this end, naïve Balb/c mice (female, 8–10 weeks) were injected with ^68^Ga‐complexes intravenously, followed by a biodistribution urine metabolite analysis at 2 h and 1 h post injection, respectively. KK‐COOH and KO‐NH_2_ were selected as lead structures based on their favorable uptake and bacterial internalization properties and compared with the corresponding [^68^Ga][Ga(Ent)]^3−^ complex. Biodistribution reveals enterohepatic and renal clearance for all ^68^Ga‐complexes (Figures S165). The [^68^Ga][Ga(KO‐NH_2_)]^3−^ complex demonstrated most efficient blood and organ clearance and no dechelation in urine metabolites, exhibiting compound properties that compare favorably to [^68^Ga][Ga(Ent)]^3−^. In contrast, enhanced organ retention was observed for [^68^Ga][Ga(KK‐COOH)]^3−^ combined with diminished metabolic inertness (Figure S167).

### 2.6 Thermodynamic Stability Constants of [M(KO‐NH_2_)]^3−^

In good correlation with previous reports, we determined that Ent forms both thermodynamically stable Fe^3+^ and Ga^3+^ complexes with log β_110_ values of 50.1 and 48.5, respectively [57, 59]. Both synthetic analogues TREN‐CAM and ME‐CAM (Table 1) have diminished thermodynamic stability constants: 49.4 for [Fe(TREN‐CAM)]^3−^ and 44 for [Fe(ME‐CAM)]^3−^ versus 49.4 and 41 for [Ga(TREN‐CAM)]^3−^and [Ga(ME‐CAM)]^3−^, respectively [60, 61, 62, 63]. The decrease in the thermodynamic stability constant for Ga^3+^ is in accordance with expectations based on the lack of covalent bonding character when compared to the corresponding Fe^3+^ complex.

**TABLE 1:** Measured log β_110_ values of select synthetic and natural product Fe^3+^ and Ga^3+^ complexes, in direct comparison with those obtained for the KO‐NH_2_ complexes.

Complex	log β_110_ (Fe)	log β_110_ (Ga)
[M(ME‐CAM)]^3−^	41	38
[M(Ent)]^3−^	50.1	48.5
[M(3,4‐LICAM)]^3−^	41	38.5
[M(HDFO)]^3−^	30.6	27.6
[M(TREN‐CAM)]^3−^	50.1	49.4
[M(OK‐COOH)]^3−^	31.1	30.0
[M(KO‐NH_2_)]^3−^	36	39.9

However, our results with KO‐NH_2_ revealed the opposite trend, which is unprecedented for siderophore and siderophore mimics: the [Ga(KO‐NH_2_)]^3−^ complex exhibited a near 4‐order of magnitude greater log β_110_ value (39.9) when compared with the corresponding [Fe(KO‐NH_2_)]^3−^ complex (36). Indeed, the Fe^3+^ complex persists as the salicylate complex under physiological conditions in contrast to the Ga^3+^ complex (Figure S115, Table S14), which forms the tris‐catecholate at lower pH by taking advantage of the greater relative Lewis acidity. The preferential binding affinity to Ga^3+^ supports the designation of KO‐NH_2_ as a galliphore. This characteristic appears amino acid sequence dependent, as OK‐COOH shows no inversion of the typical thermodynamic stability trend, with a log β_110_ of 30.0 [Ga(OK‐COOH)]^3−^ versus 31.1 for [Fe(OK‐COOH)]^3−^, and significant contribution of the salicylate species to the physiologically relevant species. The high thermodynamic stability, paired with kinetic inertness observed for the tracer [^68^Ga][Ga(KO‐NH_2_)]^3−^ further qualifies the KO‐NH_2_ dipeptide as a lead for further development.

### 2.7 PET Imaging of Soft Tissue Infection

We next assessed [^68^Ga][Ga(KO‐NH_2_)]^3−^ for diagnostic imaging in a preclinical model of soft tissue infection. The animal model requires the induction of a localized infection in mice using 10^8^ colony forming units (CFU) *E. coli* in the left triceps and inflammation with lipopolysaccharide (LPS) in the right, contralateral triceps [25, 64, 65, 66]. After a 5‐hour latency period following the introduction of the soft tissue infection, mice were intravenously injected with [^68^Ga][Ga(KO‐NH_2_)]^3−^, followed by PET imaging at 90 min post injection. Biodistribution was assessed by PET imaging and 2‐h terminal biodistribution. [^68^Ga][Ga(KO‐NH_2_)]^3−^ readily localized at the site of infection with statistically significant uptake (*p* < 0.05) in the infected muscle tissue, in comparison with inflamed and healthy muscle (Figure 6A,B). The uptake into the *E. coli*‐infected muscle amounted to 1.28 ± 0.12 %ID/g, 3.0‐fold higher than into the sterile inflamed muscle (0.42 ± 0.08 %ID/g, *p* = 0.0009, Figure 6B). Indeed, Brönstrup work previously reported 1.7‐fold increased uptake in *E. coli*‐infected muscle over inflamed muscle from their cyclen‐based probes [64], suggesting [^68^Ga][Ga(KO‐NH_2_)]^3−^ as an efficient diagnostic agent for Gram‐negative, *E. coli* infection. Urine metabolite analysis showed 95% intact complex at 2‐h postinjection in accordance with the naïve mouse data (Figure 6C). Furthermore, [^68^Ga][Ga(KO‐NH_2_)]^3−^ exhibited enhanced renal clearance and reduced blood retention when compared with [^68^Ga][Ga(Ent)]^3−^ (Figure S165, Table S27 and S28) [67, 68].

**FIGURE 6:**
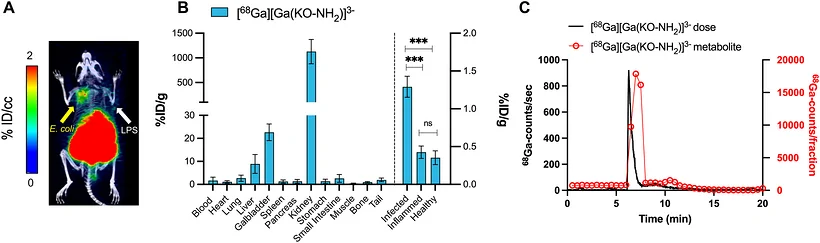
Evaluation of [^68^Ga][Ga(KO‐NH_2_)]^3−^ in an *E. coli* K12 infection model. (A) Representative positron emission tomography/computed tomography images of [^68^Ga][Ga(KO‐NH_2_)]^3−^ 1 h post administration of radiotracer in mice. Images are normalized to units of %ID/cc and presented as maximum intensity projection scans (MIPS). % ID/cc is the percentage of injected dose per cubic centimeter. (B)  Ex vivo biodistribution profile of [^68^Ga][Ga(KO‐NH_2_)]^3−^ 2 h postinjection (n = 4). % ID/g of organ is the percentage of injected dose per gram of organ weight. Significant higher uptake (paired *t*‐test, *p* < 0.05) in the infected muscled is observed as compared to the control muscle. (C) Metabolite analysis and dose formulation prior to administration of [^68^Ga][Ga(KO‐NH_2_)]^3−^.

### 2.8 Construction of a Siderophore‐Antibiotic Conjugate

Following our expansive structure–activity relationship (SAR) chelate campaign to identify a suitable gallium chelate, we also probed if KO‐NH_2_ was compatible with the construction of potent siderophore antibiotic. Therefore, we designed a conjugate incorporating periplasmic antibiotic ampicillin [69]. As solid‐ and solution‐phase amidation approaches yielded no appreciable amounts of desired product, we drew inspiration from Nolan, Brönstrup, and coworkers who had successfully adapted copper‐mediated click chemistry to introduce an alkyne‐functionalized ampicillin [30, 70]. Fmoc‐Lys(N_3_) was used as a third amino acid, and synthesis was completed on resin, followed by removal of the benzoyl protecting groups using BCl_3_ to prevent degradation of the azide functional group. Conventional Cu‐click chemistry produced the desired product incorporating the potent β‐lactam (Figure 7, Scheme S2).

**FIGURE 7:**
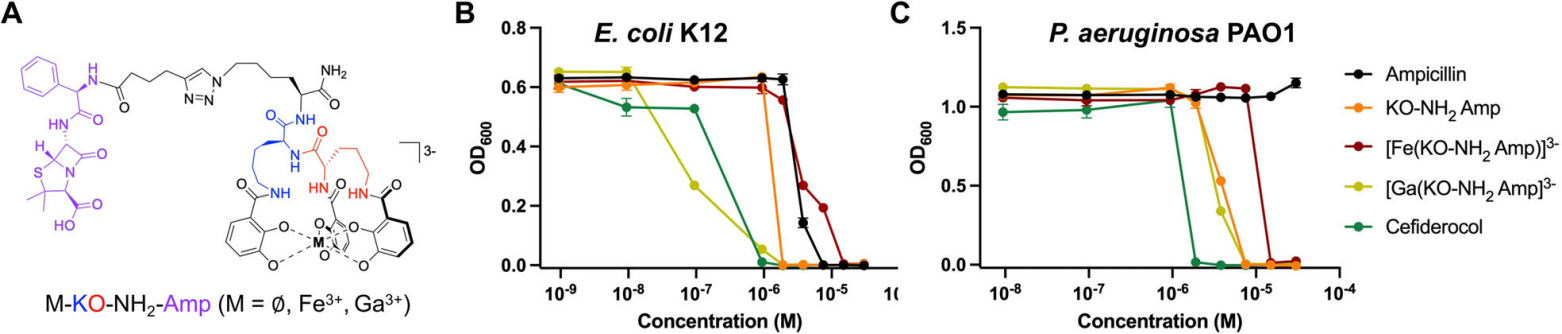
(A) Structure of M‐KO‐NH_2_‐Amp. (B) Antibacterial activity of M‐KO‐NH_2_‐Amp in *E. coli* K12 (n = 9). (C) Antibacterial activity of M‐KO‐NH_2_‐Amp in *P. aeruginosa* PAO1.

Antibacterial activity of the novel SAC was probed in *E. coli* and *P. aeruginosa wt* (PAO1) strains by conventional microbroth dilution assay quantifying the MIC_98_. MIC_98_ is determined by the lowest concentration of SAC required to inhibit the growth of bacterial population by 98%. Ampicillin and cefiderocol were used as controls, with both providing MIC_98_ values of 7.5 and 0.94 µM, respectively, in *E. coli* K12 (Table 2) [71, 72]. The β‐lactam functionalized derivative KO‐NH_2_‐Amp exhibited a growth‐inhibitory profile consistent with previously reported siderophore‐linked ampicillins [31]. successfully enhancing the potency of the parent antibiotic by nearly 4‐fold in *E. coli* K12 (MIC_98_ = 1.9 µM, Figure 7). As the transmembrane transporter expression decreases in the presence of iron, we observe reduced potency for the Fe^3+^ conjugate (MIC_98_ = 15 µM) in good agreement with our previous studies [23]. The Ga^3+^ complex retained potency comparable to the metal‐free KO‐NH_2_‐Amp conjugate (MIC_98_ = 1.9 µM). Unlike our previous cytoplasm‐targeting SAC, the ampicillin payload targets the periplasm, providing a potential rationale for a lack of potentiation by the Ga^3+^ complex [26].

**TABLE 2:** MIC_98_ values in µM obtained in strains of interest with KO derivatives, in direct comparison with clinically employed antibiotics ampicillin and cefiderocol. Nd = not determined. R = resistant.

Bacterial strain	KO‐NH_2_‐Amp	Ga(KO‐ NH_2_‐Amp)]^3−^	[Fe(KO‐NH_2_‐Amp)]^3−^	Cefiderocol	Ampicillin
*E. coli* K12	1.9 µM	1.9 µM	15 µM	0.94 µM	7.5 µM
*PA* O1	7.5 µM	7.5 µM	15 µM	1.9 µM	> 30 µM/**R**
*E. coli* Δfiu	1.9 µM	Nd	Nd	0.94 µM	15 µM
*E. coli* ΔfepA	1.9 µM	Nd	Nd	0.94 µM	15 µM
*E. coli Δ*cirA	1.9 µM	Nd	Nd	1.9 µM	15 µM
*E. coli* Xen14	1.9 µM	1.9 µM	Nd	0.94 µM	> 30 µM/**R**

^a^ iron‐deficient growth conditions obtained by treatment with aq. solution of 2,2′‐bipyridine (DP).

To probe the putative pathway of internalization, we determined MIC_98_ values in strains with incompetent outer membrane transporters implicated in Ent/triscatechol transport. In accordance with the trifurcated internalization pathway for catechols, which can utilize FepA, Fiu, and the CirA outer membrane transporter, we observed that KO‐NH_2_‐Amp retained its 1.9 µM MIC_98_ value in *E. coli* ΔfepA*, E. coli* Δfiu, and *E. coli* ΔcirA, in good correlation with cefiderocol; of note, cefiderocol shows a minor decrease in potency in the *E. coli* ΔcirA strain, correlating well with the hypothesized, dominant outer membrane uptake pathway of cefiderocol in *E. coli*. This is further corroborated by radioactive uptake experiments with [^67^Ga][Ga(KO‐NH_2_‐Amp)]^3−^ in *E. coli K12* and *E. coli* ΔfepA, showing that uptake is diminished in *E. coli* ΔfepA, but remains above 5% (Figures S154 and S155, Tables S25 and S26).

In *P. aeruginosa*, we observed comparable MIC_98_ values as with *E. coli* for KO‐NH_2_‐Amp and [Ga(KO‐NH_2_‐Amp)]^3−^ with MIC_98_ of 7.5 µM. This demonstrates resensitization to ampicillin, which is not active in *P. aeruginosa* due to low permeability of porins [73]. While similar behavior has been observed previously with other polycatechol‐ampicillin conjugates, the potency of KO‐NH_2_‐Amp readily exceeds those of previous SAC's and indicates that combination with more potent beta‐lactams could further enhance and repotentiate their activity [31].

### 2.9 Direct Identification of Putative Transmembrane Transporters in *E. coli* and *P. aeruginosa*

Despite the availability of knockout strains, the identification of membrane transporters for synthetic and natural siderophores remains indirect and therefore elusive. Recently, we have developed a covalent photoaffinity enrichment strategy, paired with MS/MS sequence analysis and subtraction of off‐target binders which identifies siderophore‐binding outer membrane proteins that interact with our constructs from live bacterial culture [74]. Accordingly, we synthesized a diazirine‐biotin‐linked conjugate, KO‐NH_2_‐azir‐biotin, and conducted photoaffinity enrichment experiments in *E. coli* K12 and *P. aeruginosa* PAO1 (Figures 8A–B). Due to Ent's role in *E. coli*, the uptake of endogenous catechols and catechol‐based siderophore mimics is well established. Indeed, enrichment assays using apo KO‐NH_2_‐azir‐biotin show preferential tagging of CirA, Fiu, and FepA relative to the alkyne‐azir‐biotin control, with CirA and Fiu exhibiting higher spectral counts (Figure 8C). These data are consistent with recognition of KO‐NH_2_ conjugates by catecholate‐associated outer membrane receptors. Photoaffinity enrichment experiments with Fe^3+^ and Ga^3+^ complexes of KO‐NH_2_‐azir‐biotin confirmed the role of CirA and Fiu as the main transporters of KO‐NH_2_ conjugates, whereas FepA showed minimal enrichment. These results suggest that metal preorganization of KO‐NH_2_ scaffold may favor recognition by CirA and Fiu over FepA, which is consistent with the radiochemical uptake data indicating FepA‐independent uptake contributions. Moreover, while several abundant porins, including NmpC, OmpA, and OmpC, were preferentially enriched with the apo KO‐NH_2_‐azir‐biotin probe compared to the control, they did not appear to be involved in the internalization of either the iron‐ or gallium‐bound KO‐NH_2_‐azir‐biotin complexes. It is likely that the pre‐organization in metal complexes facilitates recognition by specific transporters in contrast to apo siderophores, which may interact nonspecifically with abundant outer membrane porins.

**FIGURE 8:**
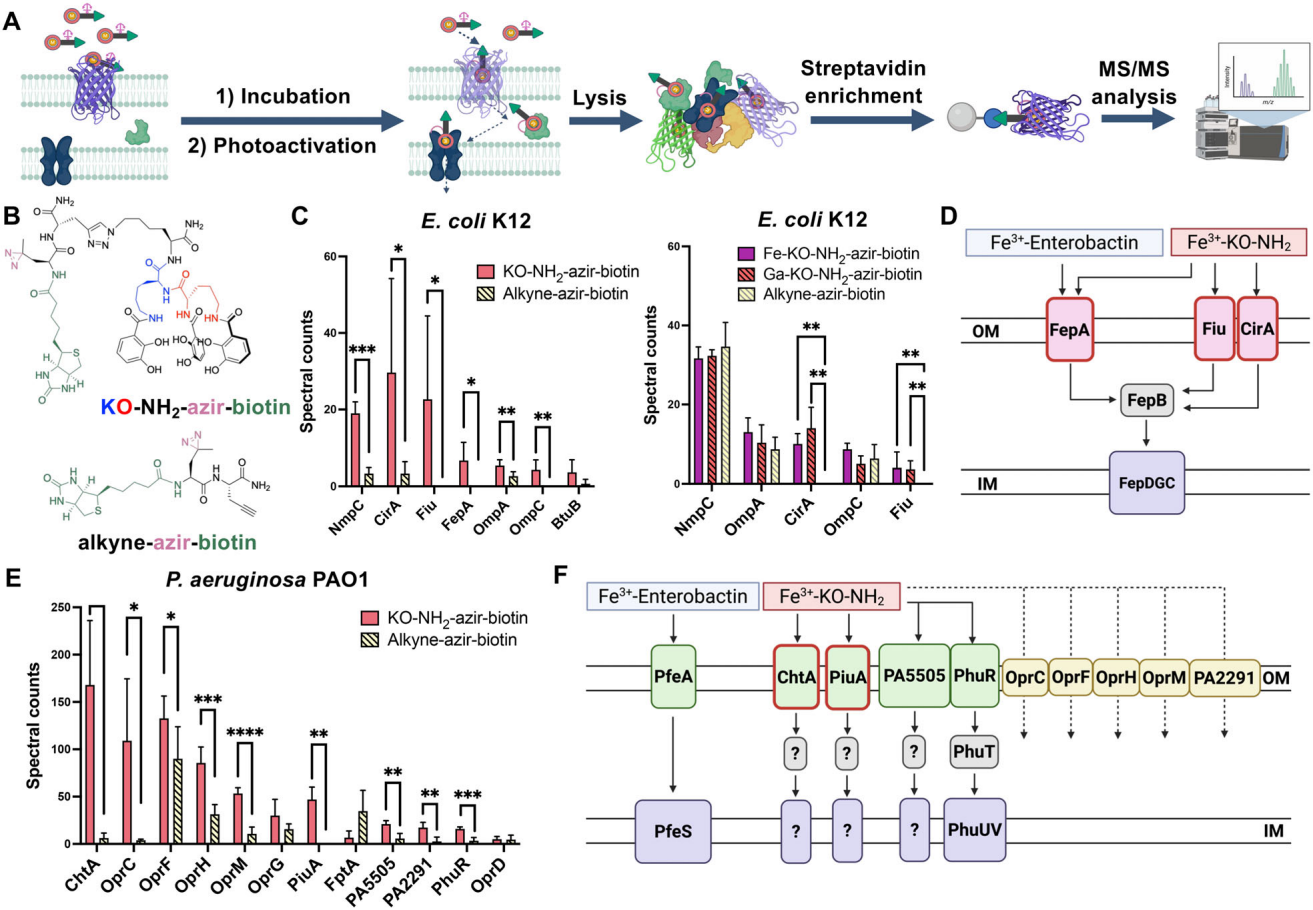
KO‐NH_2_‐azir enrichment in *E. coli* K12 and *P. aeruginosa* PAO1 cells. (A) Enrichment protocol: incubation of KO‐NH_2_‐azir probe or control azir‐biotin, photo‐conjugation to interacting proteins, followed by biotin‐streptavidin enrichment and MS/MS analysis. (B) Structure of KO‐NH_2_‐azir and control alkyne‐azir‐biotin. (C) Quantification of transporters enriched with M‐KO‐NH_2_‐azir‐biotin (M = ∅, Fe, Ga) and control alkyne‐azir‐biotin in *E. coli* K12, by spectral counts. (D) Schematic representation of transporters of KO‐NH_2_ derivatives identified in *E. coli* K12 by enrichment experiments. (E) Quantification of transporters enriched with KO‐NH_2_‐azir‐biotin and control alkyne‐azir‐biotin in *P. aeruginosa* PAO1, by spectral counts. (F) Schematic representation of transporters of KO‐NH_2_ derivatives identified in *P. aeruginosa* PAO1 by enrichment experiments. The results shown are representative of three independent experiments (Tables S29–S31). * *p* < 0.1; ** *p* < 0.05; *** *p* < 0.01; **** *p* < 0.001.

We next applied our photocrosslinking strategy to *P. aeruginosa* for which the uptake pathways of enterobactin and enterobactin mimics have not been studied as extensively as in *E. coli*. Indeed, the recent co‐crystal structure study of PfeA and enterobactin, as well as PC represented unprecedented insight into substrate recognition of non‐endogenous siderophores and siderophore mimics in this organism. Our direct pulldown strategy did not identify PfeA as an outer membrane transporter that significantly contributes to the recognition and uptake of KO‐NH_2_‐azir‐biotin (Figure 8E). Instead, we identified ChtA, a TonB‐dependent outer membrane transporter previously implicated in internalization of hydroxamate‐containing xenosiderophores [75], and PiuA, a second TonB‐dependent transporter internalizing catecholates (Figure 8E) [76]. Similar to *E. coli* K12, KO‐NH_2_‐azir‐biotin appeared to bind to several outer membrane porins, including major porin OprF.

In addition to identification of the two dominant TonB‐dependent outer membrane proteins ChtA and PiuA, our photocrosslinker probe also identified TonB‐dependent receptor OprC, whose crystal structure was recently resolved and is hypothesized as a copper transporter [77]. Furthermore, another probable TonB‐dependent receptor, PA5505, was also identified as a target.

### 2.10 Therapeutic Intervention: Treatment of an *E. coli* Soft Tissue Infection

The promising growth‐inhibitory activity of [Ga(KO‐NH_2_‐Amp)]^3−^ and KO‐NH_2_‐Amp prompted us to investigate its potential to treat a soft tissue infection via systemic administration in direct comparison with cefiderocol. To allow for the noninvasive monitoring of disease progression with optical imaging, we employed *E. coli* Xen14, a bioluminescent bacterial strain derived from the enteropathogenic *E. coli* strain WS2572, which possesses a stable chromosomal copy of the photorhabdus luminescens lux operon. MIC_98_ determination indicated that growth‐inhibitory activity correlated with those measured in *E. coli* K12 (Table 2) but was resistant ampicillin [78, 79]. This activity profile affirms KO‐NH_2_‐Amp's ability to resensitize a bacterial strain to an antibiotic it had already gained resistance to.

Soft tissue infections were induced in accordance with the previously employed model for PET imaging: Balb/c mice were anesthetized, and bacterial inoculum (2 x 10^8^ CFU/mL) was injected in the left triceps of the mice. Subsequently, we administered antibiotic compounds subcutaneously at 1 and 25 h post‐bacterial inoculation [24]. The in vitro activity profile provided guidance for dosing considerations for the systemic treatment of a soft tissue infection with antibiotics. Thus, cefiderocol was dosed at 94 µmol kg^−1^, whereas KO‐NH_2_‐Amp and [Ga(KO‐NH_2_‐Amp)]^3−^ were dosed at 190 µmol kg^−1^ in accordance with MIC_98_ values. Treatment progression was monitored for 3 days postinfection using optical imaging. After 3 days, mice were euthanized, and infection burden was determined by CFU counting (Figure 9, Figures S193–S196, Table S33).

**FIGURE 9:**
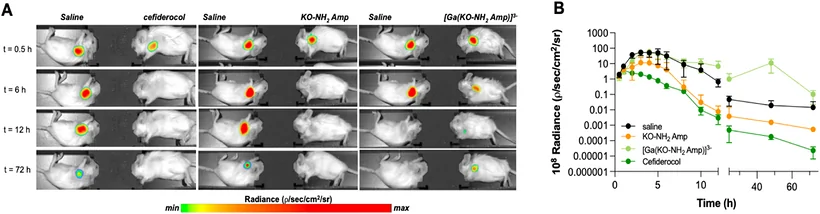
(A) Optical imaging of mice with soft tissue infection in the right triceps, before (*t* = 0.5 h) and after (6–72 h) receiving antibiotic treatment with cefiderocol, KO‐NH_2_‐Amp, and [Ga(KO‐NH_2_‐Amp)]^3−^. (B) Summary of radiance values obtained for individual cohorts via region of interest analysis, demonstrating that KO‐NH_2_‐Amp and cefiderocol provide most effective treatment (*n* = 5, Table S34). Welch's *t*‐test showed statistically significant between cohorts at *t* = 12 h; *p‐value = 0.015 (saline vs. KO‐NH_2_‐Amp) and *p = 0.015 (saline vs. cefiderocol).

Bioluminescence imaging and region of interest (ROI) analysis (Figure 9, Table S34) revealed rapid loss of the infection burden with cefiderocol at 6 h after treatment. KO‐NH_2_‐Amp also reduced the bioluminescence signal in direct comparison with the saline cohort. CFU measurements were in accordance with imaging results. Treatment with KO‐NH_2_‐Amp eradicated the infection in 2/5 animals and reduced it by 2 orders of magnitude in the other 3. Cefiderocol provided successful treatment in 5/5 mice, a reflection of the greater antibiotic potency of this compound due to the differing, dihydrothiazine lactam structure. In stark contrast, [Ga(KO‐NH_2_‐Amp)]^3−^ showed no beneficial efficacy based on bioluminescence imaging and CFU counting (Supporting Information Figures S193–S196, Table S33). While follow‐up studies are ongoing, we hypothesize that the presence of the hydrophilic Ga^3+^ complex alters pharmacokinetics significantly, leading to accelerated renal excretion, reducing exposure at the infection site [26, 27].

## 3 Conclusion

In conclusion, this study demonstrates the feasibility of the rational design of PC‐inspired, SPPS‐compatible siderophore mimics with optimized coordinative properties for Ga^3+^, a redox‐inactive Fe^3+^ mimic, while maintaining compatibility with bacterial iron uptake pathways. Among the small library of ornithine (O) and lysine (K) dipeptide permutations, KO‐NH_2_ emerged as a uniquely promising scaffold, exhibiting not only efficient bacterial uptake but also an unprecedented thermodynamic preference for Ga^3+^ over Fe^3+^. This inversion of expected stability trends supports the designation of KO‐NH_2_ as a synthetic “galliphore,” with implications for both diagnostic and therapeutic applications. Specifically, the [^68^Ga][Ga(KO‐NH_2_)]^3−^ complex exhibits high in vivo stability, favorable pharmacokinetics, and enables effective PET imaging of *E. coli* soft tissue infection model. Conjugation of KO‐NH_2_ to ampicillin yielded a potent SAC capable of resensitizing resistant bacterial strains; a biotin‐linked analogue revealed the implication of multiple outer membrane receptors in *E. coli* and *P. aeruginosa*. Determination of putative transmembrane transporters responsible for the translocation of the synthetic siderophore opens possibilities to identify internalization pathways for in other bacteria beyond the ones studied in this work. While [Ga(KO‐NH_2_‐Amp)]^3−^ did not enhance therapeutic efficacy in vivo, likely due to altered pharmacokinetics, KO‐NH_2_‐Amp demonstrated significant antibacterial activity and treatment potential in an ampicillin‐resistant, enteropathogenic strain of *E. coli*.

Collectively, these findings highlight the versatility of PC‐inspired, peptidic scaffolds as platforms for selective metal chelation and delivery to bacteria within the mammalian host. The ability to decouple metal‐binding thermodynamics from biological recognition opens new avenues for the design of dual‐function agents that integrate targeted drug delivery with diagnostic imaging.

## Author Contributions

**Phuong Nguyen Tran**: conceptualization, investigation, writing – original draft, methodology, validation, visualization, formal analysis, data curation. **Axia Marlin**: methodology, validation, data curation. **Edith K. Amason**: methodology, data curation. **Marie Huynh**: methodology, data curation. **Hannah Carlisle**: methodology, data curation. **Eszter Boros**: conceptualization, investigation, funding acquisition, writing – original draft, methodology, visualization, writing – review and editing, formal analysis, project administration, resources, supervision, data curation.

## Conflicts of Interest

The authors declare no conflicts of interest.

## Supporting information



The authors provide detailed synthetic procedures and characterization, raw data, and have cited additional references within the Supporting Information.**Supporting File**: anie74267‐sup‐0001‐SuppMat.pdf.

## Data Availability

The data that support the findings of this study are available on request from the corresponding author. The data are not publicly available due to privacy or ethical restrictions.
