# Supplementary material for: Rational Design of a Synthetic Galliphore for the Diagnostic Nuclear Imaging and Treatment of Bacterial Infections
Source: Angew Chem Int Ed Engl. 2026 Sep 4;65(43):e9696366. doi: 10.1002/anie.9696366 (PMC13676416; doi:10.1002/anie.9696366)
Supplement: Supplementary file 1 [file ANIE-65-e9696366-s001.pdf]

Rational Design of a Synthetic Galliphore for the Diagnostic Nuclear Imaging and Treatment of Bacterial Infections.

Phuong Nguyen Tran, Axia Marlin, Edith K. Amason, Marie Huynh, Hannah Carlisle and Eszter Boros.*

Department of Chemistry, University of Wisconsin Madison, 1101 University Avenue, Madison, Wisconsin, 53705, USA *Email: eboros@wisc.edu

1. Experimental procedures ... 3

1.1 General methods ... 3
1.2 Small molecule building block synthesis ... 5
1.3 Synthesis of protochelin-inspired ligands ... 6
1.4 Synthesis of the ampicillin conjugates ... 10
1.5 Synthesis of biotin-based photoaffinity probe ... 13
1.6 Analytical characterization of ligands ... 16
1.6.1 Analytical HPLC traces ... 16
1.6.2 HRMS Spectra ... 18
1.6.3 NMR Spectra ... 20
1.7 Analytical characterization of conjugates and probes ... 28
1.7.1 Analytical HPLC traces ... 28
1.7.2 HRMS spectra ... 30
1.7.3 NMR spectra ... 32
1.8 Complex synthesis ... 42
1.8.1 Iron complexation ... 42
1.8.2 Gallium complexation ... 42
1.9 Analytical and spectroscopic characterization of the complex ... 43
1.9.1 KK-COOH ... 43
1.9.2 KO-NH_2 ... 47
1.9.3 KO-COOH ... 53
1.9.4 OK-COOH ... 58
1.9.5 KO-NH_2-Amp ... 63
1.9.6 KO-NH_2-azir-biotin ... 66
1.10 Density Functional Theory (DFT) Calculations ... 70
1.11 pH-dependent UV-Vis spectrophotometric speciation ... 140
1.11.1 KO-NH_2 ... 140
1.11.2 Ent ... 144
1.11.3 TREN-CAM ... 149
1.11.4 OK-COOH ... 154

2. Microbiology assays ... 159

2.1 Growth promotion assay ... 159
2.2 Antimicrobial activity assay (MIC_{98}) ... 159
2.2.1. E. coli K12 MIC experiments for ligands ... 160
2.2.2. E. coli (K12, mutants) MIC and growth curves for conjugates ... 162
2.2.3. PAO1 MIC experiments for ligands ... 166
2.3 Bacterial uptake using ^{67}Ga-based complexes ... 168

3. Radiochemistry ... 174

3.1 Production and labeling procedures ... 174

3.1.1. $^{67}GaCl_3$ 174
3.1.2. $^{68}GaCl_3$ 174
3.2 Radio-HPLC traces 175
3.2.1. ^{67}Ga-based complexes 175
3.2.2. ^{68}Ga-based complexes 178

4. Pharmacokinetics in naive and infected mice models 180

4.1 Evaluation of naive mice with $[^{68}Ga][Ga(KK\text{-}COOH)]^{3-}$ and $[^{68}Ga][Ga(KO\text{-}NH_2)]^{3-}$ 180
4.1.1 Biodistribution 180
4.1.2 Metabolite analysis 182
4.2 Evaluation of E. coli infected mice with $[^{68}Ga][Ga(KO\text{-}NH_2)]^{3-}$ 183
Procedure 183
4.2.1 PET-CT imaging 183
4.2.2 Biodistribution 184
4.2.3 Tissue dissociation and plating 186

5. Biotin-Streptavidin Pull Down Assay 187

5.1.1 Biotin-Streptavidin enrichment 187
5.1.2 SDS-PAGE 187
5.1.3 MS/MS analysis 188

6. Efficacy assessment in bioluminescent E. coli XEN 14 infected mice 197

6.1.1 Sample preparation 197
6.1.2 Bacteria inoculum preparation and compounds administration 197
6.1.3 Bioluminescent imaging 198
6.1.4 CFU determination 204
6.1.5 Bioluminescence ROI analysis 207

7. References 208

1. Experimental procedures

1.1 General methods

All starting materials were purchased from Acros Organics, Alfa Aesar, Sigma Aldrich, Thermo Fisher, Ambeed, or TCI America and used without further purification. ^{45}Ti was received from the University of Wisconsin-Madison Cyclotron Lab (GE PETtrace cyclotron), and ^{67}Ga was received from the Jubiliant Radiopharma.

NMR spectra (^1H, $^{13}C\{^1H\}$, COSY, HSQC and HMBC) were collected at the University of Wisconsin-Madison Department of Chemistry Paul Bender Chemical Instrumentation Center (CIC) using a Bruker Avance III 500 with a DCH liquid He cryoprobe (Bender Fund), a Bruker Avance Neo 500 with a 5mm Prodigy-BBO liquid N_2 cryoprobe (NSF CHE-2017891), and a Bruker Avance III 600 with a TCI-F liquid He cryoprobe (NIH S10 OD012245). Data were processed using MestReNova 14.3.3-33362. Chemical shifts (δ) are reported in parts per million (ppm) relative to tetramethylsilane. ^1H, $^{13}C\{^1H\}$, COSY, HSQC NMR spectra are referenced to residual solvent signals. NMR spectra are referenced in MestReNova using a ^1H NMR spectra as an absolute reference.

High resolution ESI mass spectrometry was carried out at the University of Wisconsin-Madison Department of Chemistry Paul Bender Chemical Instrumentation Center (CIC) using a Thermo Scientific Q Exactive Focus Orbitrap MS system, and at the University of Wisconsin-Madison School of Pharmacy Analytical Instrumentation Cluster using a Bruker MaXis Ultra-High Resolution Quadrupole Time-of-Flight MS system.

Ultraviolet-visible spectra were collected with the NanoDrop 1C instrument (AZY1706045). Spectra were recorded from 190 to 850 nm in a quartz cuvette with 1 cm path length.

Inductively Coupled Plasma- Optical Emission Spectroscopy (ICP-OES): Metal ion concentrations were determined using an Agilent 5110 ICP-OES. A 10-point standard with respect to gallium and iron was used, and lines of best fit were found with R^2 of 0.999. Sample concentration was determined based on this calibration curve.

Analytical- and radio-HPLC analysis were carried out an Agilent 1260 Infinity II system equipped with a binary gradient pump, UV-vis detector, and automatic injector. Both instruments were set to detect UV absorption was recorded at 220 nm and 254 nm and were coupled to an in-line LabLogic Dual Scan-RAM detector and were controlled using the LabLogic Laura software package. Radio-HPLC analyses utilised a LabLogic 1” NaI photomultiplier tube detector with 2” lead shielding and radio-TLC analyses utilised a LabLogic plastic photomultiplier tube detector.

Semipreparative HPLC was carried out using an Agilent 1260 Infinity II system equipped with a binary gradient pump, UV-vis detector, and manual injector. UV absorption was recorded at 220 and 254 nm. Flash chromatography was carried out using a Combi Flash Rf system with UV detection at 220 and 254 nm.

Liquid chromatography mass spectrometry (LCMS) was carried out on a Phenomenex Luna C18 column (5 µm, 150 mm × 3 mm, 100 Å, AXIA packed) at a flow rate of 0.8 mL/min using a single quadrupole Agilent 1200 Infinity II LC/MSD system equipped with a binary gradient pump, UV- vis detector, automatic injector, and an atmospheric pressure electrospray ionization (AP-ESI) source. Ultraviolet absorption was recorded at 220 nm and 254 nm, and positive and negative mass spectra were collected from m/z = 100-800.

Chromatography Solvent Systems:

Analytical HPLC - Method A: binary solvent system (A: water + 0.1% FA; B: MeCN + 0.1% FA); gradient (0–2 min: 5% B; 2–14 min: 5–95% B; 14–16 min: 95% B; 16–16.5 min: 95–5% B; 16.5–20 min 5% B);

flow rate: 0.8 mL/min; column: Phenomenex Luna C18 column (5 µm, 150 mm × 3 mm, 100 Å, AXIA packed).

Analytical HPLC - Method B: binary solvent system (A: water + 0.1% TFA; B: MeCN + 0.1% TFA); gradient (0–2 min: 5% B; 2–14 min: 5–95% B; 14–16 min: 95% B; 16–16.5 min: 95–5% B; 16.5–20 min 5% B); flow rate: 0.8 mL/min; column: Phenomenex Luna C18 column (5 µm, 150 mm × 3 mm, 100 Å, AXIA packed).

Analytical HPLC - Method C: binary solvent system (A: 10 mM ammonium formate pH 7.4; B: MeCN); gradient (0–2 min: 5% B; 2–14 min: 5–95% B; 14–16 min: 95% B; 16–16.5 min: 95–5% B; 16.5–20 min 5% B); flow rate: 0.8 mL/min; Phenomenex Luna C18 column (5 µm, 150 mm × 3 mm, 100 Å, AXIA packed).

Analytical HPLC - Method D: binary solvent system (A: 10 mM sodium acetate pH 4.5; B: MeCN); gradient (0–2 min: 5% B; 2–14 min: 5–95% B; 14–16 min: 95% B; 16–16.5 min: 95–5% B; 16.5–20 min 5% B); flow rate: 0.8 mL/min; column: Phenomenex Luna C18 column (5 µm, 150 mm × 3 mm, 100 Å, AXIA packed).

Semipreparative HPLC - Method E: binary solvent system (A: water + 0.1% TFA; B: MeCN + 0.1% TFA); gradient: (0–3 min: 5% B; 3–23 min: 5–95% B; 23–26 min: 95% B; 26–27 min: 95–5% B; 27–30 min: 5% B); flow rate: 30 mL/min; column: Phenomenex Luna C18 column (250 mm × 21.2 mm, 100 Å, AXIA packed).

Semipreparative HPLC - Method E1: binary solvent system (A: water + 0.05% TFA; B: MeCN + 0.05% TFA); gradient: (0–3 min: 5% B; 3–23 min: 5–95% B; 23–26 min: 95% B; 26–27 min: 95–5% B; 27–30 min: 5% B); flow rate: 30 mL/min; column: Phenomenex Luna C18 column (250 mm × 21.2 mm, 100 Å, AXIA packed).

Flash chromatography - Method F: Binary solvent system (A: water + 0.1% TFA; B: MeCN + 0.1% TFA); flow rate: 40 mL/min; column: RediSep C18 column (50 g HP gold); gradient (0-2min: 5% B; 2-12 min: 5-95% B; 12-14 min: 95% B; 14-16 min: 95-5% B).

LCMS - Method G: binary solvent system (A: water + 0.1% FA; B: MeCN + 0.1% FA); gradient (0-3 min: 5% B; 3-10 min: 5-95% B; 10-13 min: 95% B; 13-13.5 min: 95-5% B; 13.5-16 min: 5% B); flow rate: 0.8 mL/min; column: Phenomenex Luna C18 column (5 µm, 150 mm × 3 mm, 100 Å, AXIA packed).

LCMS - Method H: binary solvent system (A: 10 mM ammonium formate pH 7.4; B: MeCN); gradient (0-3 min: 5% B; 3-10 min: 5-95% B; 10-13 min: 95% B; 13-13.5 min: 95-5% B; 13.5-16 min: 5% B); flow rate: 0.8 mL/min; column: Phenomenex Luna C18 column (5 µm, 150 mm × 3 mm, 100 Å, AXIA packed).

1.2 Small molecule building block synthesis

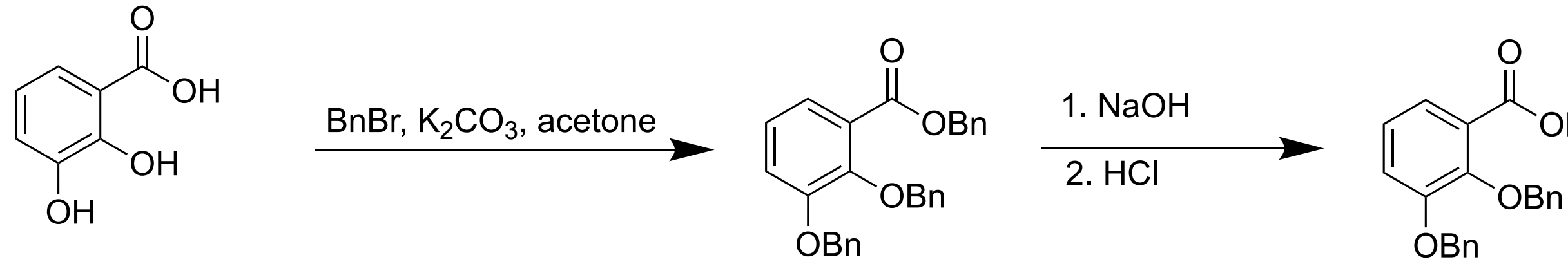

(1)

***2,3-bis(benzyloxy)benzoic acid,* (1).** A solution of 2,3-dihydroxybenzoic acid (10.00 g, 65.0 mmol), benzyl bromide (67.00 g, 390 mmol), and K_2CO_3 (54.00 g, 390 mmol) in acetone (250 mL) was refluxed for 1 day under nitrogen. After filtration, the solution was concentrated in vacuo to give the crude product, as a yellow oil. The crude product was dissolved in methanol (200 mL) and an aqueous solution of 5 M NaOH (110 mL) was added. The mixture was refluxed for 3 h and the solution concentrated in vacuo to remove the methanol. The residue was dissolved in water and extracted thrice with hexane. Then the water phase was acidified with 3 M HCl to pH 2 and filtered to give the product **1** as a white solid (20.55 g, 93%). R_f 0.62 (40% acetone/hexane); ^{1}H NMR (500 MHz, $CDCl_3$) δ 7.77 (dd, J = 7.9, 1.6 Hz, 1H), 7.51 (d, J = 6.7 Hz, 2H), 7.44 (dt, J = 11.9, 6.9 Hz, 2H), 7.37 (s, 5H), 7.32 – 7.27 (m, 1H), 7.22 (t, J = 8.0 Hz, 1H), 5.29 (s, 2H), 5.22 (s, 2H); ESI-MS m/z calcd for $C_{21}H_{18}O_4Na$ $[M + Na]^+$ 357.1097, found 357.1116. The data are consistent with those previously reported.[1]

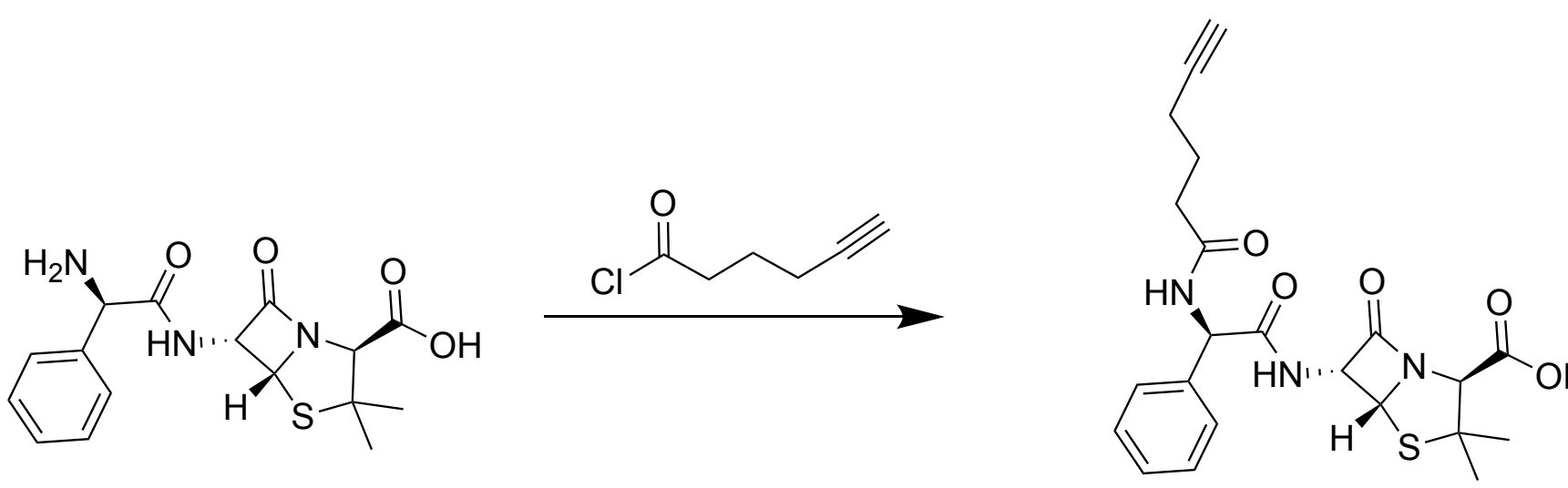

(3)

***(2S,5R,6R)-6-((R)-2-(hex-5-ynamido)-2-phenylacetamido)-3,3-dimethyl-7-oxo-4-thia-1-azabicyclo[3.2.0]heptane-2-carboxylic acid* (3)**. 5-Hexynoic acid (113 µL, 1.00 mmol) and thionyl chloride (1.00 mL, 13.8 mmol) were combined and refluxed for 1 h. The reaction was cooled to room temperature and concentrated under reduced pressure, and the resulting crude acyl chloride was dissolved in acetone (0.5 mL) and carried on to the next step without purification. Ampicillin sodium salt (186 mg, 0.500 mmol) was dissolved in a solution of $NaHCO_3$ (210 mg, 2.5 mmol) in 4:1 water/acetone (2.5 mL) and cooled on ice, to which the acyl chloride was added slowly stirring. The reaction was subsequently warmed to room temperature and stirred for 1 h. Water (3 mL) was added to the reaction, and the aqueous phase was washed with EtOAc (2 × 10 mL), acidified to pH 2 by addition of HCl, and extracted with EtOAc (20 mL). The resulting organic phase was washed with cold water (2 × 5 mL), dried over Na_2SO_4, and concentrated under reduced pressure. The crude reaction was triturated with hexanes, which afforded (3) as a yellow solid (180 mg, 77%). Crude was purified via HPLC (Method E1). MS (ESI): $[M+Na]^+$ m/z calcd 466.1407, found 466.1404. The data are consistent with those previously reported.[2]

1.3 Synthesis of protochelin-inspired ligands

All ligands were synthesized by solid-phase peptide synthesis on a 0.5 mmol scale in fritted glass vessels. DMF was used as the solvent for all coupling and deprotection steps unless otherwise stated. Coupling efficiency was monitored after each coupling step by test cleavage followed by LCMS analysis. Wang resin, 0.57 mmol g^{-1}, was used for C-terminal carboxylate ligands, whereas Rink amide resin, 0.62 mmol g^{-1}, was used for C-terminal amide ligands.

First donor coupling on 0.5 mmol scale. Preloaded Fmoc-Lys(Mtt)-Wang resin (0.877 g, 0.5 mmol) was swolen in DMF (15 mL) for 20 min. 1% TFA in DCM was used to deprotect Mtt protecting group until yellow solution disappeared. The resin was washed with DCM (3 x 5 mL) and DMF (3 x 5 mL). **Bzl-CAM(1)** (0.5012 g, 1.5 mmol), PyBOP (0.7806 g, 1.5 mmol), and DIPEA (523 mL, 3.0 mmol) was dissolved in DMF (10.5 mL) and added to the resin. The coupling solution was shaken for 16 h at 25°C. After draining the coupling solution, the resin was washed with DMF (3 x 5 mL) and DCM (3 x 5 mL).

Second amino acid coupling on 0.5 mmol scale. To the resin, 20% v/v piperidine in DMF (10 mL) was added and rocked for 20 min(2 x). After deprotection, the resin was washed with DMF (3 x 5 mL) and DCM (3 x 5 mL). Following, Fmoc-Lys(Mtt) (0.936 g, 1.5 mmol), PyBOP (0.7806 g, 1.5 mmol), and DIPEA (523 mL, 3.0 mmol) was dissolved in DMF (10.5 mL) and added to the resin. The coupling solution was shaken for 16 h at 25°C. After coupling, the resin was washed with DMF (3 x 5 mL) and DCM (3 x 5 mL).

Second donor coupling on 0.5 mmol scale. To the resin, 1% TFA in DCM was used to deprotect Mtt protecting group until yellow solution disappeared. The resin was washed with DCM (3 x 5 mL) and DMF (3 x 5 mL). **Bzl-CAM(1)** (0.5012 g, 1.5 mmol), PyBOP (0.7806 g, 1.5 mmol), and DIPEA (523 mL, 3.0 mmol) was dissolved in DMF (10.5 mL) and added to the resin. The coupling solution was shaken for 16 h at 25°C. After draining the coupling solution, the resin was washed with DMF (3 x 5 mL) and DCM (3 x 5 mL).

Third donor coupling on 0.5 mmol scale. To the resin, 20% v/v piperidine in DMF (10 mL) was added and rocked for 20 min (2 x). After deprotection, the resin was washed with DMF (3 x 5 mL) and DCM (3 x 5 mL). **Bzl-CAM(1)** (0.5012 g, 1.5 mmol), PyBOP (0.7806 g, 1.5 mmol), and DIPEA (523 mL, 3.0 mmol) was dissolved in DMF (10.5 mL) and added to the resin. The coupling solution was shaken for 16 h at 25°C. After draining the coupling solution, the resin was washed with DMF (3 x 5 mL) and DCM (3 x 5 mL).

Resin cleavage and global deprotection. For compounds synthesized on a 0.50 mmol scale, a 16 mL cleavage cocktail consisting of 95% TFA, 2.5% TIPS, and 2.5% water was added to resin. After 30 min of rocking, the resin slurry was filtered and washed with TFA (3 x 3 mL) and DCM (3 x 3 mL). The filtrate was concentrated to yield yellow residue.

Ether precipitation. Diethyl ether (~15 mL) was added to the residue, and the mixture was sonicated to obtain a white precipitate, which was separated from the liquid phase by centrifugation for 5.0 min at 4,000 rpm. The pellet was washed twice more with ~15 mL of diethyl ether, each time breaking up the pellet by sonication, and the crude product was allowed to air dry.

Benzyl Deprotection. The crude was dissolved in anhydrous MeOH. Pd/C was added 20% w/w to the solution, followed by H_2 gas. The reaction was monitored for 24 h. Pd/C was filtered out and the reaction was evaporated to yield yellow-brown oil.

Purification: Crude samples were dissolved using a mixture of 50% MeOH in MeCN before being subjected to CombiFlash Reverse Phase (Method F). Fractions were analyzed for the product using LCMS and lyophilized to obtain pure product.

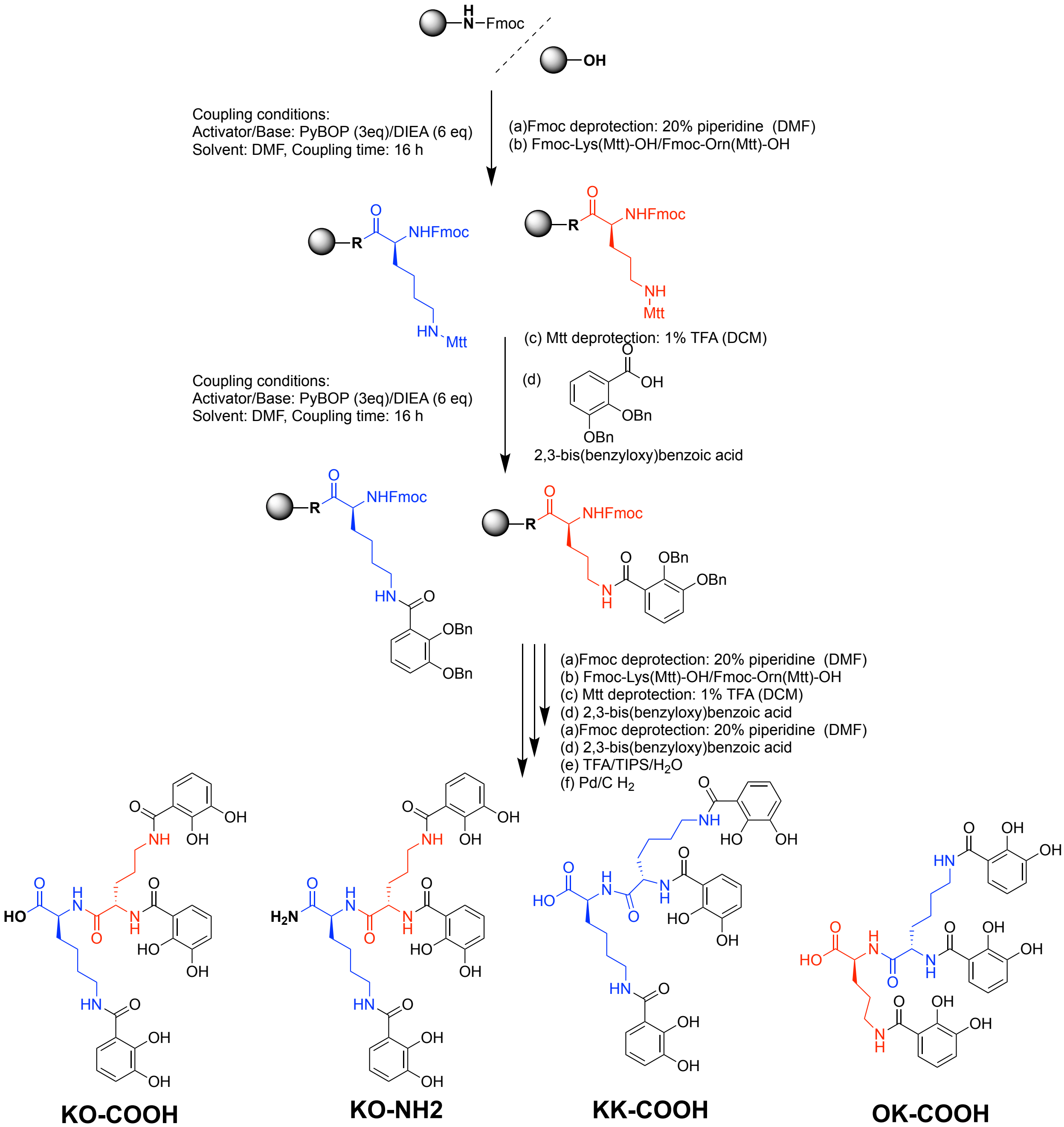

Scheme S1. Solid-Phase workflow of protochelin-based siderophore library

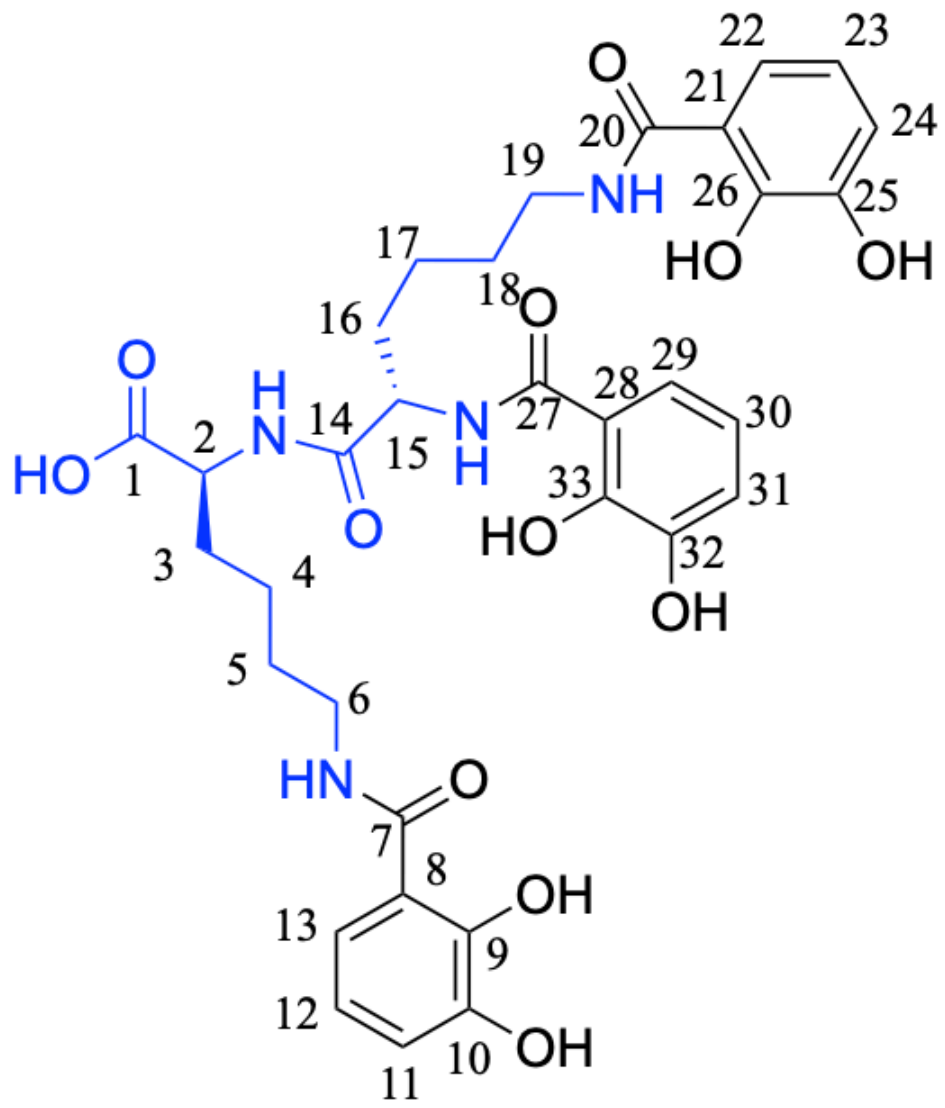

***N*²-(*N*²,*N*⁶-bis(2,3-dihydroxybenzoyl)-*L*-lysyl)-*N*⁶-(2,3-dihydroxybenzoyl)-*L*-lysine** (**KK-COOH**). The desired product was obtained was a white solid in 15 % (76 mg, 0.11 mmol). ^{1}H NMR (500 MHz, MeOD, 298 K) δ 7.21 (dd, *J* = 8.1, 1.5 Hz, 1H, H^{29}), 7.09 (ddd, *J* = 7.6, 5.9, 1.5 Hz, 2H, H^{22} and H^{13}), 6.87 – 6.76 (m, 3H, H^{30}, H^{23}, H^{12}), 6.64 – 6.53 (m, 3H, H^{31}, H^{24}, H^{11}), 4.53 (dd, *J* = 8.4, 5.7 Hz, 1H, H^{15}), 4.32 (dd, *J* = 8.9, 4.8 Hz, 1H, H^2), 3.27 (dt, *J* = 12.1, 7.0 Hz, 4H, H^{19} and H^6), 1.85 (dddt, *J* = 18.4, 11.6, 9.4, 5.5 Hz, 2H, H^{16}), 1.78 – 1.63 (m, 2H, H^3), 1.55 (tqd, *J* = 13.4, 6.9, 3.4 Hz, 4H, H^{18} and H^5), 1.48 – 1.35 (m, 4H, H^{17} and H^4). ^{13}C NMR (126 MHz, MeOD, 298 K) δ 174.24 (1C, C^1), 172.97 (1C, C^{14}), 170.10 (1C, C^{27}), 169.46 (2C, C^7 and C^{20}), 148.82 (3C, C^9, C^{26}, and C^{33}), 145.77 (3C, C^{10}, C^{25}, and C^{32}), 118.16 (3C, C^{13}, C^{29}, and C^{22}), 117.27 (3C, C^8, C^{21}, and C^{28}), 115.76 (3C, C^{12}, C^{23}, and C^{30}), 115.40 (3C, C^{11}, C^{24}, and C^{31}), 53.42 (1C, C^2), 52.47 (1C, C^{15}), 38.88 (2C, C^6 and C^{19}), 31.44 (2C, C^5 and C^{18}), 31.03 (1C, C^3), 28.55 (1C, C^{16}), 22.86 (2C, C^4 and C^{18}). ESI-HR-MS calcd. for $C_{33}H_{32}N_4O_{12}$ $[M + H]^+$: *m/ z* 683.2559. Found $[M + H]^+$: 683.2547 and $[M + Na]^+$: 705.2263. HPLC Retention time: 8.93 min. (Method A).

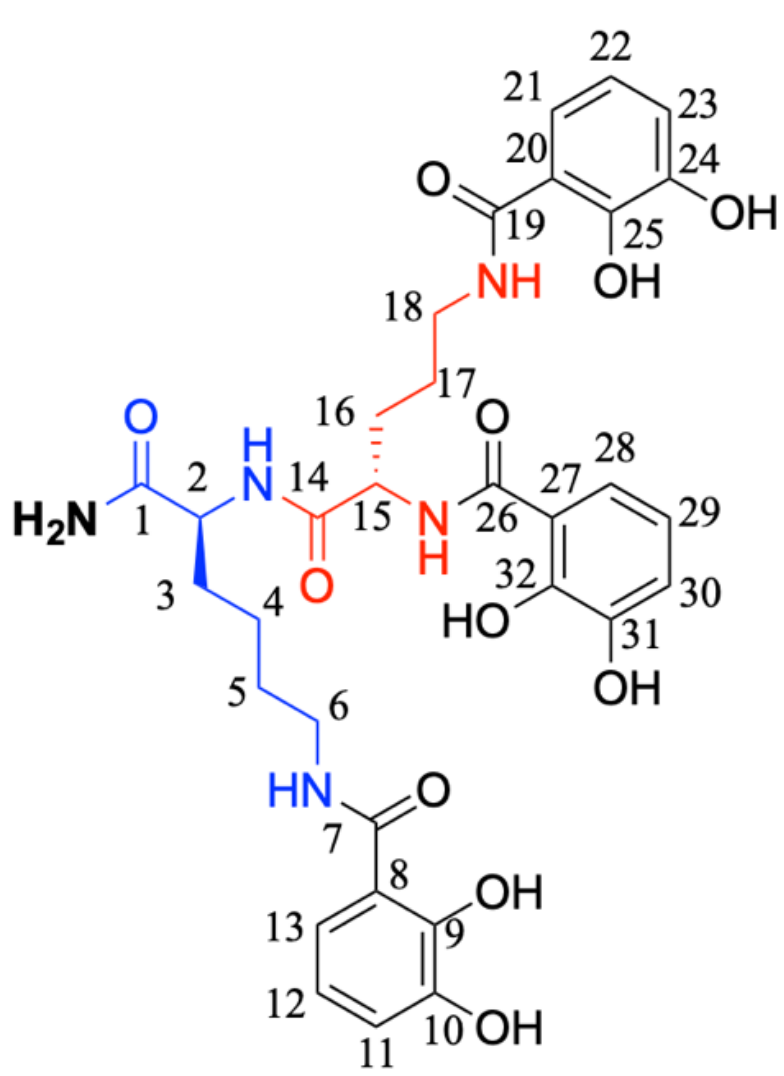

***N*,*N*'-((*S*)-5-(((*S*)-1-amino-6-(2,3-dihydroxybenzamido)-1-oxohexan-2-yl)amino)-5-oxopentane-1,4-diyl)bis(2,3-dihydroxybenzamide)** (**KO-NH₂**). The desired product was obtained as a white solid in 20 % (64 mg, 0.096 mmol). ^{1}H NMR (500 MHz, MeOD, 298 K) δ 7.24 (dd, *J* = 8.1, 1.5 Hz, 1H, H^{28}), 7.12 (td, *J* = 8.4, 1.4 Hz, 2H, H^{13} and H^{21}), 6.92 – 6.80 (m, 3H, H^{12}, H^{22} and H^{29}), 6.69 – 6.56 (m, 3H, H^{11}, H^{23} and H^{30}), 4.32 – 4.24 (m, 2H, H^{15} and H^2), 3.40 – 3.29 (m, 2H, H^{18}), 3.22 (td, *J* = 3.8, 2.1 Hz, 2H, H^6), 1.97 – 1.86 (m, 1H, H^3), 1.79 (tdd, *J* = 15.0, 10.1, 5.7 Hz, 2H, H^{16}), 1.66 (tq, *J* = 13.3, 6.9 Hz, 4H, H^3 and H^5), 1.52 (ddq, *J* = 13.4, 9.9, 6.6 Hz, 2H, H^{17}), 1.44 – 1.29 (m, 2H, H^4). ^{13}C NMR (126 MHz, MeOD, 298 K) δ 176.05 (1C, C^1), 175.99 – 175.91 (1C, C^{14}), 173.45 –173.35 (1C, C^{26}), 170.68 –170.00 (2C, C^7 and C^{19}), 149.05 – 148.32 (3C, C^9, C^{32}, and C^{25}), 146.03 – 145.94 (3C, C^{10}, C^{31}, and C^{24}), 128.88 – 128.25 (3C, C^{13}, C^{28} and C^{21}), 119.05 – 118.77 (3C, C^8, C^{27}, and C^{20}), 117.91 – 117.85 (3C, C^{12}, C^{29}, and C^{22}), 116.23 – 115.77 (3C, C^{11}, C^{30}, and C^{23}), 56.71– 56.37 (1C, C^2), 53.97 – 53.84 (1C, C^{15}), 53.55 – 53.42 (1C, C^{18}), 39.38 – 38.98 (1C, C^6), 31.61 – 31.57 (1C, C^3), 29.30 – 28.79 (1C, C^5), 25.75 (1C, C^{16}), 23.17 (1C, C^{17}), 16.37 –16.07 (1C, C^4). ESI-HR-MS calcd. for $C_{32}H_{37}N_5O_{11}$ $[M + H]^+$: *m/ z* 668.2562. Found $[M + H]^+$: 668.2553 and $[M + Na]^+$: 690.2368. HPLC Retention time: 8.38 min. (Method A).

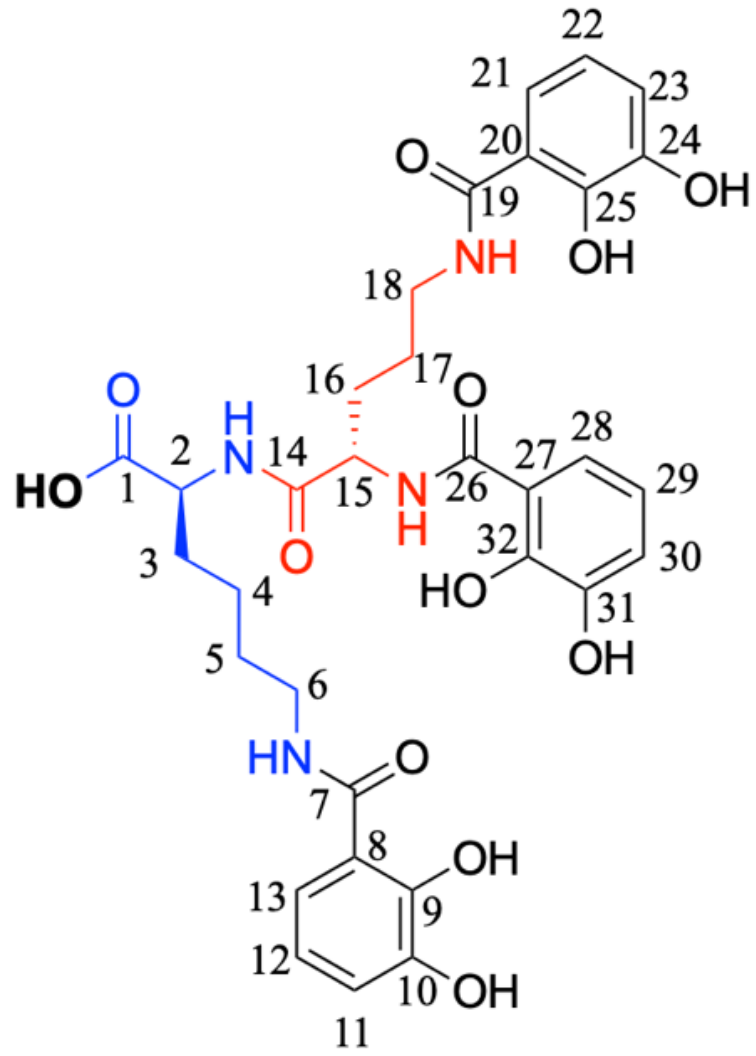

***N*[2]-((*S*)-2,5-bis(2,3-dihydroxybenzamido)pentanoyl)-*N*[6]-(2,3-dihydroxybenzoyl)-*L*-lysine** (**KO-COOH**). The desired product was obtained as a white solid in 17 % (57 mg, 0.085 mmol). ^{1}H NMR (500 MHz, MeOD, 298 K) δ 7.24 (dd, J = 8.1, 1.6 Hz, 1H, H^{28}), 7.13 – 6.98 (m, 2H, H^{13} and H^{21}), 6.86 – 6.77 (m, 3H, H^{12}, H^{22} and H^{29}), 6.61 (dq, J = 15.4, 7.8 Hz, 3H, H^{11}, H^{23} and H^{30}), 4.59 (dd, J = 8.1, 5.7 Hz, 1H, H^{15}), 4.33 (dd, J = 9.0, 4.7 Hz, 1H, H^{2}), 3.35 (dp, J = 20.5, 6.9 Hz, 2H, H^{18}), 3.27 (t, J = 7.1 Hz, 2H, H^{6}), 1.92 – 1.73 (m, 3H, H^{3} and H^{16}), 1.68 (dqd, J = 11.2, 8.1, 4.0 Hz, 3H, H^{16} and H^{5}), 1.60 – 1.49 (m, 2H, H^{17}), 1.42 (tq, J = 14.5, 7.9 Hz, 2H, H^{4}). ^{13}C NMR (126 MHz, MeOD, 298 K) δ 174.61 (1C, C^{1}), 173.55(1C, C^{14}), 170.89 (1C, C^{26}), 170.06 (2C, C^{7}, C^{19}), 149.49 (3C, C^{9}, C^{32}, C^{25}), 148.85 (3C, C^{10}, C^{31}, C^{24}), 146.42 (3C, C^{13}, C^{28}, C^{21}), 118.75 (3C, C^{8}, C^{27}, C^{20}), 117.86 (3C, C^{12}, C^{29}, C^{22}), 116.38 (3C, C^{11}, C^{30}, C^{23}), 115.97 (1C, C^{2}), 53.69 (1C, C^{15}), 52.89 (1C, C^{18}), 39.48 (1C, C^{6}), 31.50 (1C, C^{3}), 29.88 (1C, C^{5}), 29.16 (1C, C^{16}), 26.03 (1C, C^{17}), 23.52 (1C, C^{4}). ESI-HR-MS calcd. for $C_{32}H_{36}N_4O_{12}$ $[M + H]^+$: *m/ z* 669.2402. Found $[M + H]^+$: 669.2395 and $[M + Na]^+$: 691.2212. HPLC Retention time: 8.80 min (Method A).

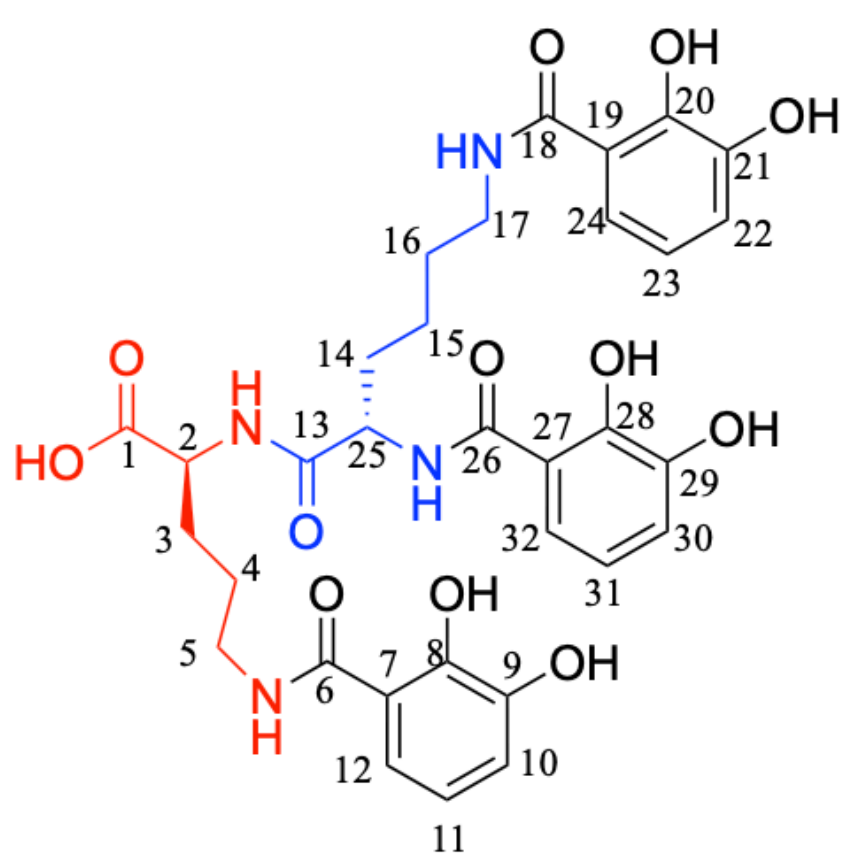

(*S*)-2-((*S*)-2,6-bis(2,3-dihydroxybenzamido)hexanamido)-5-(2,3-dihydroxybenzamido)pentanoic acid (**OK-COOH**). The desired product was obtained as a white solid in 16 % (54 mg, 0.081 mmol). ^{1}H NMR (500 MHz, MeOD, 298 K) δ 7.21 (d, J = 8.1 Hz, 1H, H^{32}), 7.08 (t, J = 6.9 Hz, 2H, H^{12} and H^{24}), 6.82 (t, J = 8.1 Hz, 3H, H^{11}, H^{23} and H^{31}), 6.60 (p, J = 7.2 Hz, 3H, H^{10}, H^{22} and H^{30}), 4.53 (dd, J = 8.3, 5.9 Hz, 1H, H^{25}), 4.37 (dd, J = 8.6, 4.8 Hz, 1H, H^{2}), 3.30 (dt, J = 13.5, 7.0 Hz, 4H, H^{5} and H^{17}), 1.95 – 1.83 (m, 2H, H^{14}), 1.81 – 1.62 (m, 4H, H^{3} and H^{16}), 1.58 (q, J = 7.3 Hz, 2H, H^{4}), 1.45 (h, J = 7.8 Hz, 2H, H^{15}). ^{13}C NMR (126 MHz, MeOD, 298 K) δ 173.79 (1C, C^{1}), 173.10 (1C, C^{13}), 170.24 (1C, C^{26}), 169.42 (2C, C^{6} and C^{18}), 148.95 (3C, C^{8}, C^{20}, and C^{28}), 145.93 (3C, C^{9}, C^{21}, and C^{29}), 118.16 (3C, C^{12}, C^{24}, and C^{32}), 117.21 (3C, C^{7}, C^{27}, and C^{19}), 115.81 (3C, C^{11}, C^{23}, and C^{31}), 115.28 (3C, C^{10}, C^{22}, and C^{30}), 53.40 (1C, C^{2}), 52.12 (1C, C^{25}), 38.87 (1C, C^{5}), 38.52 (1C, C^{17}), 31.49 (1C, C^{16}), 28.66 (1C, C^{3}), 25.53 (1C, C^{14}), 22.89 (2C, C^{4} and C^{15}). ESI-HR-MS calcd. for $C_{32}H_{36}N_4O_{12}$ $[M + H]^+$: *m/ z* 669.2402. Found $[M + H]^+$: 669.2395 and $[M + Na]^+$: 691.2212. HPLC Retention time: 8.89 min (Method A).

1.4 Synthesis of the ampicillin conjugates

All conjugates were synthesized by solid-phase synthesis on a 0.5 mmol scale in fritted glass vessels. DMF was used as the solvent for all solid-phase synthesis steps. Coupling efficiency was monitored after each coupling step by test cleavage followed by LCMS analysis. Wang or Rink amide resin was used depending on the desired C-terminal functionality.

First amino acid coupling on 0.5 mmol scale. Wang/Rink resin (0.5 mmol) was swollen in DMF (15 mL) for 20 min. Fmoc-Lys(N_3) (0.5916 g, 1.5 mmol), PyBOP (0.781 g, 1.5 mmol), and DIPEA (523 mL, 3.0 mmol) was dissolved in DMF (7.5 mL) and added to the resin. The coupling solution was shaken for 16 h at 25ºC. After draining the coupling solution, the resin was washed with DMF (3 x 5 mL) and DCM (3 x 5 mL).

Second amino acid coupling on 0.5 mmol scale. To the resin, 20% v/v piperidine in DMF (10 mL) was added and rocked for 20 min (2 x). After deprotection, the resin was washed with DMF (3 x 5 mL) and DCM (3 x 5 mL). Following, Fmoc-Lys(Mtt) (0.936 g, 1.5 mmol), PyBOP (0.781 g, 1.5 mmol), and DIPEA (523 mL, 3.0 mmol) was dissolved in DMF (10.5 mL) and added to the resin. The coupling solution was shaken for 16 h at 25ºC. After coupling, the resin was washed with DMF (3 x 5 mL) and DCM (3 x 5 mL).

First donor coupling on 0.5 mmol scale. To the resin, 1% TFA in DCM was used to deprotect Mtt protecting group until yellow solution disappeared. The resin was washed with DCM (3 x 5 mL) and DMF (3 x 5 mL). **Bzl-CAM(1)** (0.5012 g, 1.5 mmol), PyBOP (0.781 g, 1.5 mmol), and DIPEA (523 mL, 3.0 mmol) was dissolved in DMF (10.5 mL) and added to the resin. The coupling solution was shakenfor 16 h at 25ºC. After draining the coupling solution, the resin was washed with DMF (3 x 5 mL) and DCM (3 x 5 mL).

Third amino acid coupling on 0.5 mmol scale. To the resin, 20% v/v piperidine in DMF (10 mL) was added and rocked for 20 min (2 x). After deprotection, the resin was washed with DMF (3 x 5 mL) and DCM (3 x 5 mL). Following, Fmoc-Orn(Mtt) (0.915 g, 1.5 mmol), PyBOP (0.781 g, 1.5 mmol), and DIPEA (523 mL, 3.0 mmol) was dissolved in DMF (10.5 mL) and added to the resin. The coupling solution was shaken for 16 h at 25ºC. After coupling, the resin was washed with DMF (3 x 5 mL) and DCM (3 x 5 mL).

Second donor coupling on 0.5 mmol scale. To the resin, 1% TFA in DCM was used to deprotect Mtt protecting group until yellow solution disappeared. The resin was washed with DCM (3 x 5 mL) and DMF (3 x 5 mL). **Bzl-CAM(1)** (0.5012 g, 1.5 mmol), PyBOP (0.7806 g, 1.5 mmol), and DIPEA (523 mL, 3.0 mmol) was dissolved in DMF (10.5 mL) and added to the resin. The coupling solution was shaken for 16 h at 25ºC. After draining the coupling solution, the resin was washed with DMF (3 x 5 mL) and DCM (3 x 5 mL).

Third donor coupling on 0.5 mmol scale. To the resin, 20% v/v piperidine in DMF (10 mL) was added and rocked for 20 min (2 x). After deprotection, the resin was washed with DMF (3 x 5 mL) and DCM (3 x 5 mL). **Bzl-CAM (1)** (0.5012 g, 1.5 mmol), PyBOP (0.7806 g, 1.5 mmol), and DIPEA (523 mL, 3.0 mmol) was dissolved in DMF (10.5 mL) and added to the resin. The coupling solution was shaked for 16 h at 25ºC. After draining the coupling solution, the resin was washed with DMF (3 x 5 mL) and DCM (3 x 5 mL).

Resin cleavage and global deprotection. For compounds synthesized on a 0.50 mmol scale, a 16 mL cleavage cocktail consisting of 95% TFA, 2.5% TIPS, and 2.5% water was added to resin. After 30 min of rocking, the resin slurry was filtered and washed with TFA (3 x 3 mL) and DCM (3 x 3 mL). The filtrate was concentrated to yield a yellow residue.

Ether precipitation. Diethyl ether (~15 mL) was added to the residue, and the mixture was sonicated to obtain a white precipitate, which was separated from the liquid phase by centrifugation for 5.0 min at 4,000

rpm. The pellet was washed twice more with ~15 mL of diethyl ether, each time breaking up the pellet by sonication, and the crude product was allowed to air dry.

***Benzyl Deprotection for KO-NH₂-N₃*.** Benzyl-protected **KO-NH_2-N_3** (540 mg, 0.396 mmol) and pentamethylbenzene (PMB, 1.06 g, 7.128 mmol) were dissolved in 30 mL of anhydrous CH_2Cl_2 to give a light-yellow solution. This solution was cooled to −78 °C in an acetone/dry ice bath under N_2, and BCl_3 (4.752 mL of 1 M solution in CH_2Cl_2, 4.752 mmol) was added slowly along the flask wall. After the solution was stirred for 18 h, DIPEA (2.21 mL, 12.672 mmol) was added to the flask, followed by MeOH (15 mL) to quench the reaction. The reaction was then warmed to room temperature, and the solvents were removed under reduced pressure.

Purification: Crude samples were dissolved using a mixture of 50% MeOH in MeCN before being subjected to CombiFlash Reverse Phase (Method F). Fractions were analyzed for the product using LCMS and lyophilized to obtain pure product.

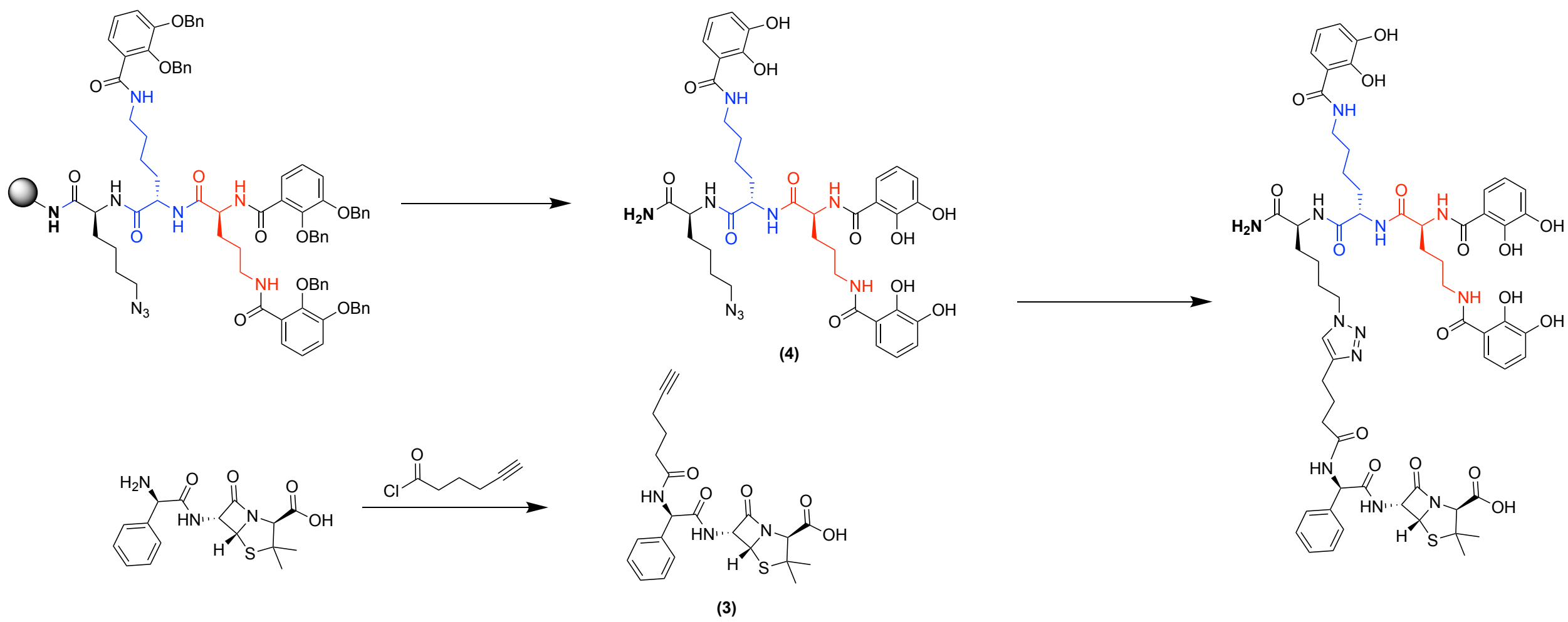

Scheme S2. CuAAC strategy for beta-lactam antibiotic conjugates, **KO-NH_2-Amp**.

***Synthesis KO-NH_2 Amp*.** Ampicillin-alkyne **3** (0.0099 g, 12 µmol) and **KO-NH_2-N_3 4** (0.0160 g, 35 µmol) were combined, and 500 µL of DMSO was added. $CuSO_4$ (74 µL of a 0.4417 M solution in water, 33 µmol) and tris[(1-benzyl-1*H*-1,2,3-triazol-4-yl)methyl]amine (TBTA, 260 µL of a 0.1402 M solution in DMSO, 36 µmol) were combined, and 300 µL of DMSO was added to give a blue-green solution, to which NaAsc (390 µL of a 0.3372 M solution in water, 130 µL) was added. This solution became light yellow and was immediately added to the alkyne/azide solution. The reaction was stirred for 3 h at room temperature, diluted by 3- to 4-fold with 1:1 MeCN/water, centrifuged (13,000 rpm × 10 min, 4 °C), and purified by semi-preparative HPLC (Method E1). The HPLC fractions containing product were collected manually and flash frozen in liquid N_2 immediately after collection to prevent β-lactam decomposition. The product was obtained as white powder (10.8 mg, 71%).

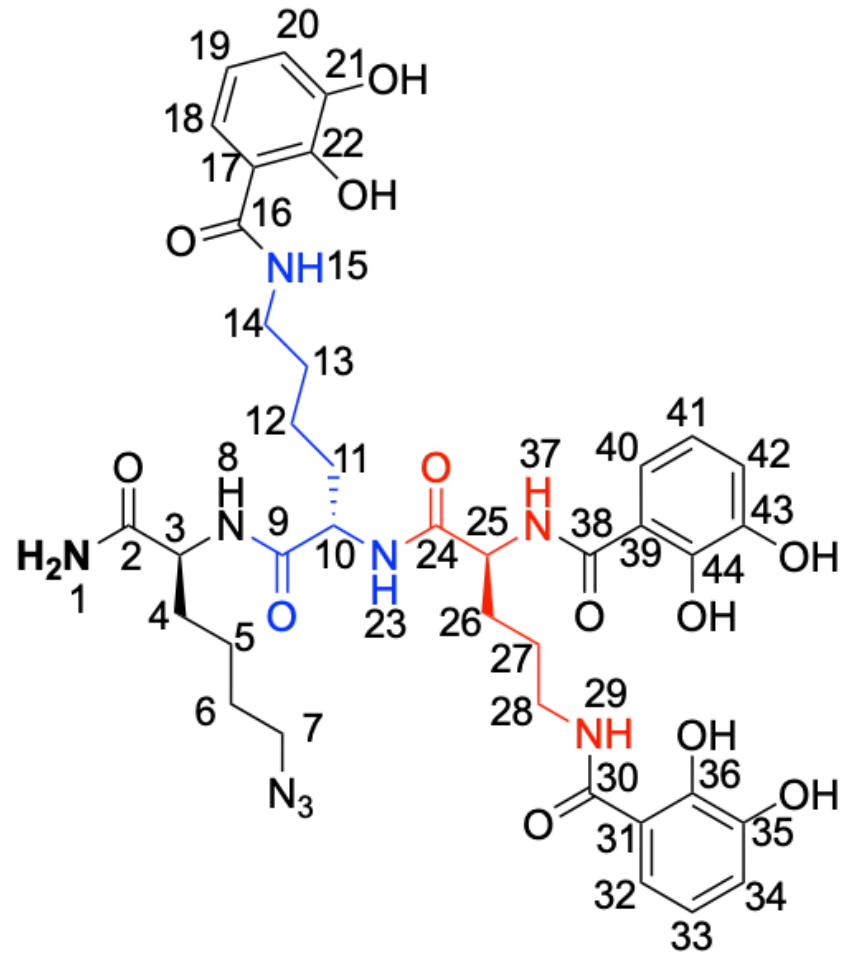

(2*S*,5*R*,6*R*)-6-((*R*)-2-(4-(1-((*S*)-6-amino-5-((*S*)-2-((*S*)-2,5-bis(2,3-dihydroxybenzamido)pentanamido)-6-(2,3-dihydroxybenzamido)hexanamido)-6-oxohexyl)-1*H*-1,2,3-triazol-4-yl)butanamido)-2-phenylacetamido)-3,3-dimethyl-7-oxo-4-thia-1-azabicyclo[3.2.0]heptane-2-carboxylic acid (KO-NH$_2$-N$_3$). The desired product was obtained was a white solid in 20 % (82 mg, 99.7 µmol). ^{1}H NMR (600 MHz, DMSO-d_6, 298 K) δ 8.81 – 8.72 (m, 2H, H^{37}, H^{29}), 8.21 (d, J = 7.8 Hz, 1H, H^{15}), 7.78 (d, J = 8.1 Hz, 1H, H^{23}), 7.41 (dd, J = 8.2, 1.5 Hz, 1H, H^{8}), 7.37 (d, J = 2.2 Hz, 1H, H^{40}), 7.30 – 7.21 (m, 2H, H^{18}, H^{32}), 7.05 (s, 2H, H^{1}), 7.00 – 6.86 (m, 3H, H^{20}, H^{34}, H^{42}), 6.74 – 6.61 (m, 3H, H^{19}, H^{33}, H^{41}), 4.54 (ddd, J = 9.2, 7.6, 5.1 Hz, 1H, H^{25}), 4.26 (td, J = 8.3, 5.2 Hz, 1H, H^{10}), 4.18 (td, J = 8.3, 5.3 Hz, 1H, H^{3}), 3.30 (q, J = 6.9 Hz, 2H, H^{28}), 3.25 (dt, J = 15.4, 7.0 Hz, 4H, H^{7}, H^{14}), 1.87 – 1.68 (m, 2H, H^{26}), 1.67 – 1.56 (m, 4H, H^{4}, H^{11}), 1.55 – 1.44 (m, 6H, H^{6}, H^{13}, H^{27}), 1.42 – 1.19 (m, 4H, H^{5}, H^{12}). ^{13}C NMR (151 MHz, DMSO-d_6, 298 K) δ 173.84 (1C, C^{2}), 171.83 (2C, C^{9}, C^{24}), 171.74 (1C, C^{38}), 170.25 (2C, C^{16}, C^{30}), 170.16 (1C, C^{44}), 169.32 (2C, C^{22}, C^{36}), 150.22 (1C, C^{43}), 149.12 (2C, C^{21}, C^{35}), 146.65 (1C, C^{40}), 146.51 (2C, C^{18}, C^{32}), 119.26 (1C, C^{39}), 119.19 (2C, C^{17}, C^{31}), 118.80 (1C, C^{41}), 118.55 (2C, C^{19}, C^{33}), 118.30 (1C, C^{42}), 117.53 (2C, C^{20}, C^{34}), 116.21 (1C, C^{3}), 115.35 (1C, C^{25}), 53.30 (1C, C^{10}), 53.18 (1C, C^{7}), 52.41 (1C, C^{28}), 50.97 (1C, C^{14}), 39.35 (1C, C^{4}), 39.14 (1C, C^{13}), 32.09 (1C, C^{26}), 29.77 (1C, C^{11}), 28.31(1C, C^{6}), 26.07 (1C, C^{5}), 23.34 (1C, C^{27}), 22.82 (1C, C^{12}). ESI-HR-MS calcd. for $C_{38}H_{47}N_9O_{12}$ [M + H]$^+$: *m/ z* 822.3416 and [M + Na]$^+$: *m/ z* 844.3236. Found [M + H]$^+$: 822.376 and [M + Na]$^+$: 844.3278.

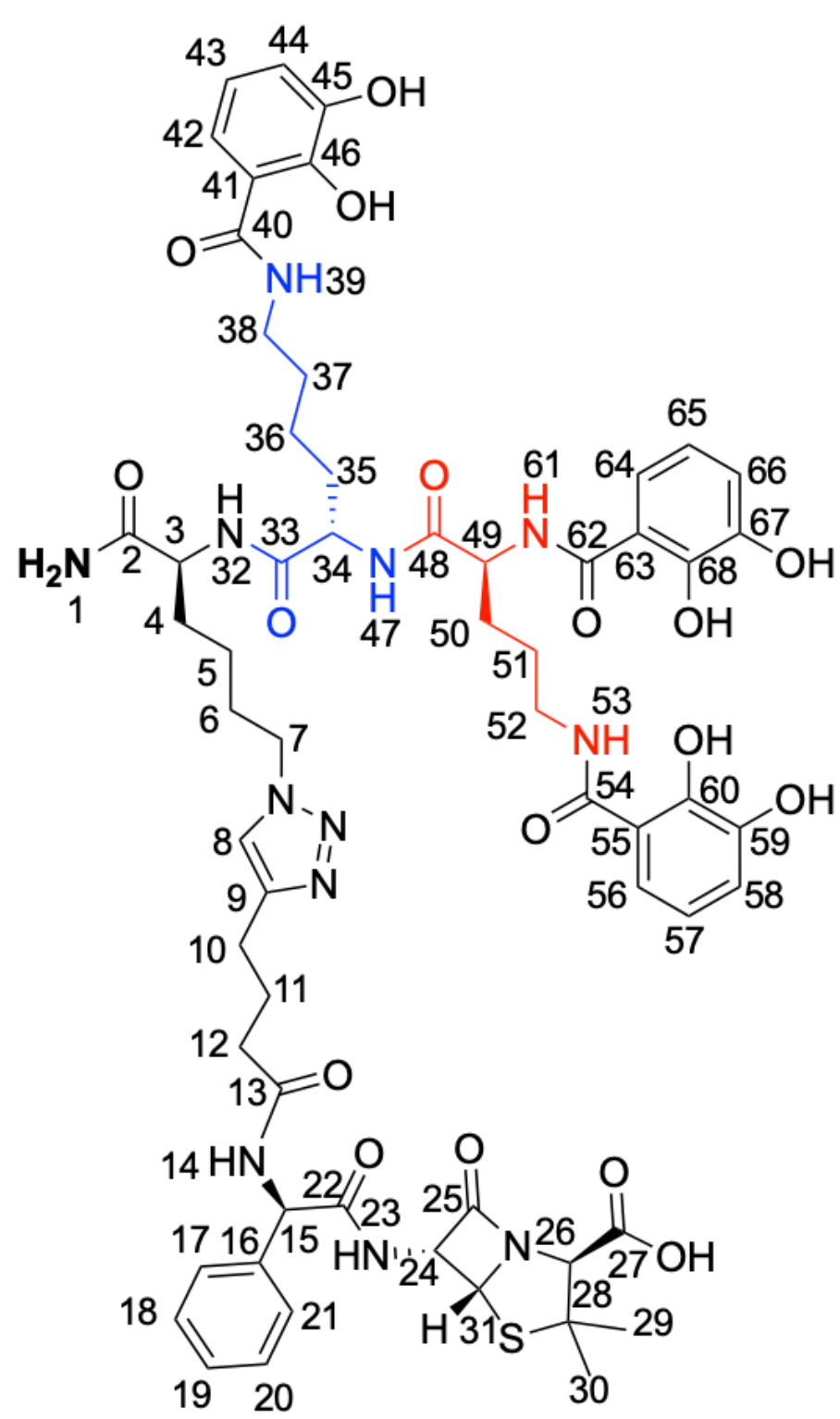

(2*S*,5*R*,6*R*)-6-((*R*)-2-(4-(1-((*S*)-6-amino-5-((*S*)-2-((*S*)-2,5-bis(2,3-dihydroxybenzamido)pentanamido)-6-(2,3-dihydroxybenzamido)hexanamido)-6-oxohexyl)-1*H*-1,2,3-triazol-4-yl)butanamido)-2-phenylacetamido)-3,3-dimethyl-7-oxo-4-thia-1-azabicyclo[3.2.0]heptane-2-carboxylic acid (KO-NH$_2$-Amp). The desired product was obtained was a white solid in 71 % (10.8 mg, 8.54 µmol) ^{1}H NMR (600 MHz, DMSO-d_6, 298 K) δ 9.06 – 9.00 (m, 1H, H^{61}), 8.73 – 8.65 (m, 2H, H^{53}, H^{39}), 8.48 (t, J = 8.6 Hz, 1H, H^{23}), 8.12 (d, 1H, H^{14}), 7.74 (d, J = 17.1 Hz, 2H, H^{47}, H^{32}), 7.72 (s, 1H, H^{8}), 7.36 – 7.34 (t, 2H, H^{18}, H^{20}), 7.29 – 7.27 (m, 1H, H^{19}), 7.25 (ddd, J = 7.8, 5.3, 3.6 Hz, 2H, H^{17}, H^{21}), 7.22 – 7.17 (m, 3H, H^{42}, H^{56}, H^{64}), 6.96 (s, 2H, H^{1}), 6.84 (ddd, J = 20.4, 7.8, 1.4 Hz, 3H, H^{44}, H^{58}, H^{66}), 6.65 – 6.55 (m, 3H, H^{43}, H^{57}, H^{65}), 5.65 (dd, J = 15.1, 8.1 Hz, 1H, H^{24}), 5.46 – 5.43 (m, 1H, H^{49}), 5.32 (dd, J = 4.1, 2.5 Hz, 1H, H^{31}), 4.47 (td, J = 8.2, 5.0 Hz, 1H, H^{34}), 4.22 – 4.14 (m, 2H, H^{7}), 4.13 (s, 1H, H^{26}), 4.10 (td, J = 8.3, 5.7 Hz, 1H, H^{3}), 2.51 (d, J = 15.2, 5.2 Hz, 2H, H^{52}), 2.25 – 2.16 (m, 2H, H^{10}), 2.11 – 1.99 (m, 2H, H^{12}), 1.71 (dp, J = 28.7, 7.4 Hz, 6H, H^{4}, H^{35}, H^{50}), 1.62 – 1.51 (m, 4H, H^{6}, H^{11}), 1.48 (m, 2H, H^{51}), 1.47 (s, 3H, H^{29}), 1.34 (s, 3H, H^{30}),

1.31 – 1.22 (m, 2H, H^{37}), 1.17 (m, 4H, H^{5}, H^{36}). ^{13}C NMR (151 MHz, DMSO-d_6, 298 K) δ 173.83 (1C, C^{25}), 172.20 (1C, C^{2}), 171.88 (1C, C^{13}), 171.83 (1C, C^{54}), 171.77 (1C, C^{40}), 170.72 (1C, C^{48}), 170.25 (1C, C^{33}), 170.17 (1C, C^{22}), 169.38 (1C, C^{62}), 158.19 (1C, C^{27}), 150.21 (1C, C^{68}), 149.15 (1C, C^{60}), 146.88 (1C, C^{46}), 146.66 (1C, C^{59}), 146.51 (1C, C^{67}), 138.67 (1C, C^{45}), 128.65 (1C, C^{16}), 127.99 (1C, C^{9}), 127.64 (2C, C^{17}, C^{21}), 127.61 (2C, C^{18}, C^{20}), 122.08 (1C, C^{19}), 119.27 (1C, C^{8}), 119.19 (1C, C^{66}), 118.31 (2C, C^{44}, C^{58}), 117.52 (3C, C^{43}, C^{57}, C^{65}), 116.19 (3C, C^{42}, C^{56}, C^{64}), 115.35 (3C, C^{41}, C^{55}, C^{63}), 84.59 (1C, C^{26}), 71.95 (1C, C^{31}), 70.75 (1C, C^{28}), 67.68 (1C, C^{24}), 64.18 (1C, C^{15}), 58.57 (1C, C^{48}), 55.87 (1C, C^{33}), 53.32 (1C, C^{3}), 53.16 (1C, C^{7}), 52.41 (1C, C^{38}), 49.48 (1C, C^{52}), 34.88 (1C, C^{12}), 31.95 (1C, C^{35}), 30.79 (1C, C^{4}), 29.88 (1C, C^{10}), 29.08 (1C, C^{37}), 27.07 (1C, C^{29}), 26.10 (1C, C^{30}), 25.72 (1C, C^{50}), 25.15 (1C, C^{51}), 24.80 (1C, C^{6}), 23.36 (1C, C^{11}), 22.64 (1C, C^{36}), 17.88 (1C, C^{5}). ESI-HR-MS calcd. for $C_{60}H_{72}N_{12}O_{17}S$ $[M + H]^+$: *m/z* 1265.4932. Found $[M + H]^+$: 1265.4921.

1.5 Synthesis of biotin-based photoaffinity probe

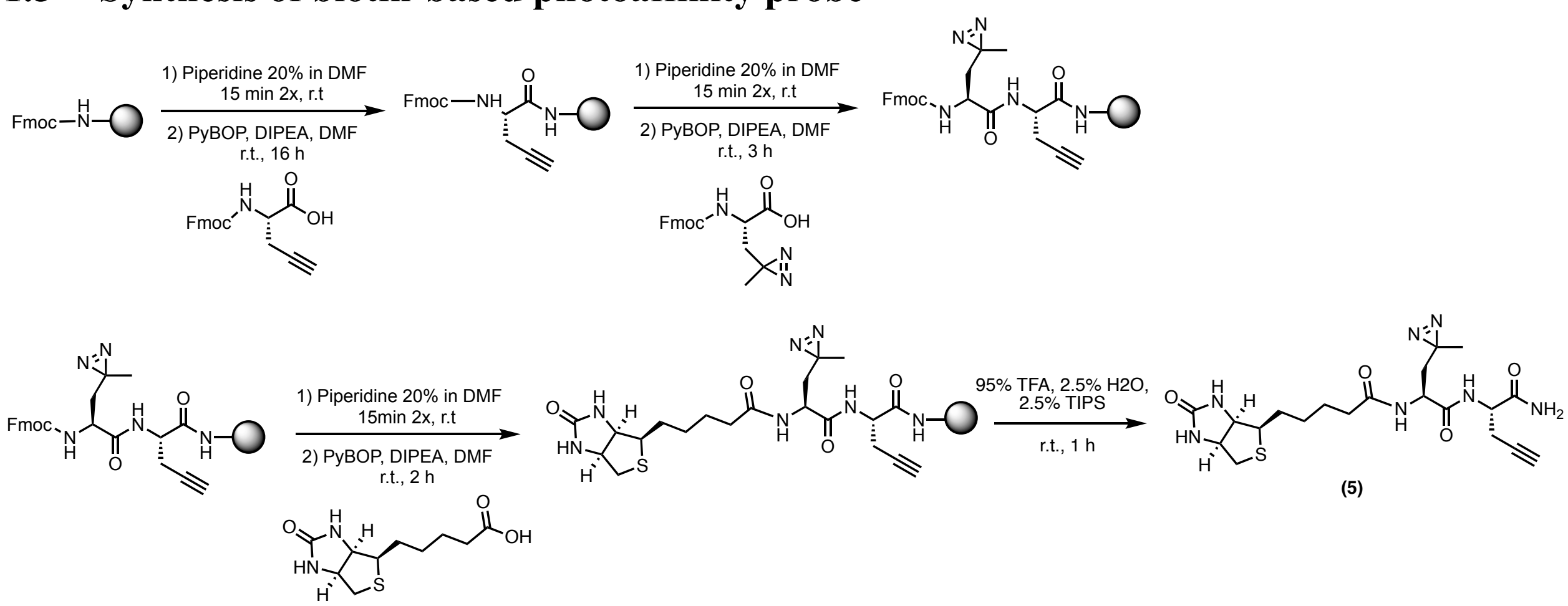

Scheme S3. Synthesis for **alkyne-azir-biotin** moiety.

Alkyne-azir-biotin (5) was synthesized by solid phase synthesis using Rink Amide (RA) resin (100 mg, 0.8 mmol/g, 1 eq.). The RA resin was swollen in DCM (2 mL) and DMF (2 mL) for 1 min, repeated three times for each solvent. Fmoc deprotection of the RA resin was achieved using a 20% (v/v) solution of piperidine in DMF, rocking for 15 min twice. Fmoc-L-Pra-OH (107 mg, 0.16 mmol, 2 eq) was loaded onto the resin using PyBOP (125 mg, 0.12 mmol, 1.5 eq) as a coupling reagent in the presence of DIPEA (111 µL, 0.32 mmol, 4 eq.). The first coupling reaction proceeded at room temperature for 16 hours. The Fmoc group was subsequently removed using a 20% (v/v) solution of piperidine in DMF, rocking for 15 min twice. Fmoc-L-photo-leucine (87.7 mg, 0.12 mmol, 1.5 eq) was loaded onto the resin using PyBOP (125 mg, 0.12 mmol, 1.5 eq) as a coupling reagent in the presence of DIPEA (111 µL, 0.32 mmol, 4 eq.). The coupling reaction proceeded at room temperature for 3 hours. The Fmoc group was subsequently removed using a 20% (v/v) solution of piperidine in DMF, rocking for 15 min twice. Biotin (78.2 mg, 0.16 mmol, 2 eq) was loaded onto the resin using PyBOP (125 mg, 0.12 mmol, 1.5 eq) as a coupling reagent in the presence of DIPEA (111 µL, 0.32 mmol, 4 eq.). The coupling reaction proceeded at room temperature for 2 hours. The compound was cleaved from the resin by treating it with a mixture of TFA/TIS/H_2O (95%/2.5%/2.5%) for 1 hour at room temperature. The product was air-dried, dissolved in DMSO, and purified by preparative HPLC (method E) to yield **alkyne-azir-biotin (5)** as a white solid (46%, 0.037 mmol).

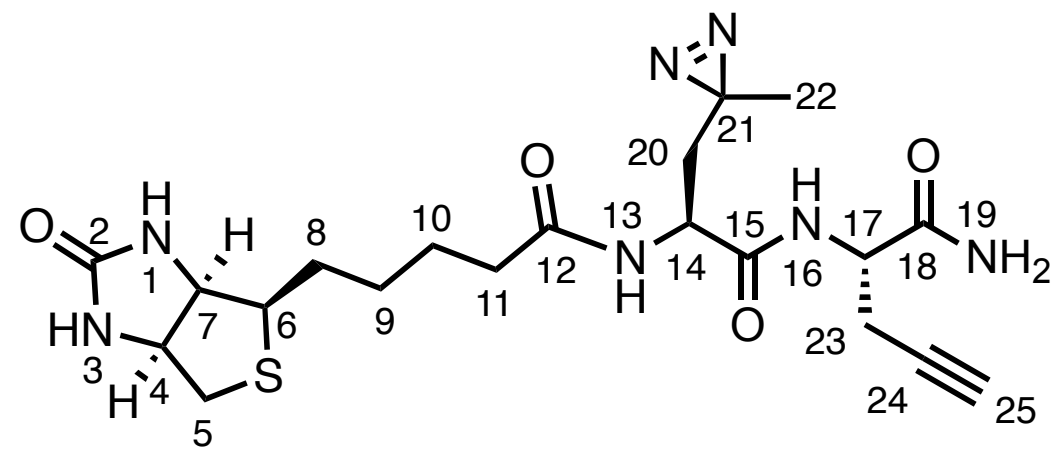

(*S*)-2-((*S*)-3-(3-methyl-3*H*-diazirin-3-yl)-2-(5-((3a*R*,4*R*,6a*S*)-2-oxohexahydro-1*H*-thieno[3,4-*d*]imidazol-4-yl)pentanamido)propanamido)pent-4-ynamide (Alkyne-azir-biotin). ^{1}H NMR (600 MHz, DMSO-d_6, 298 K) δ 8.14 (d, J = 8.8 Hz, 1H, H^{13}), 7.99 (d, J = 8.4 Hz, 1H, H^{16}), 7.40 (s, 1H, H^{19}), 7.22 (s, 1H, H^{19}), 6.45 (s, 1H, H^{1}), 6.37 (s, 1H, H^{3}), 4.31 (dd, J = 8.3, 5.7 Hz, 1H, H^{4}), 4.25 (td, J = 7.9, 5.9 Hz, 1H, H^{17}), 4.19 (ddd, J = 11.1, 8.5, 4.3 Hz, 1H, H^{14}), 4.16 – 4.11 (m, 1H, H^{7}), 3.10 (ddd, J = 8.8, 7.1, 4.8 Hz, 1H, H^{6}), 2.83 (d, J = 5.0 Hz, 2H, H^{5}), 2.82 (s, 1H, H^{25}), 2.57 (d, J = 12.7 Hz, 2H, H^{23}), 2.19 – 2.13 (m, 2H, H^{11}), 1.87 (dd, J = 15.2, 4.7 Hz, 1H, H^{20}), 1.69 – 1.58 (m, 2H, H^{9}), 1.57 (td, J = 8.9, 6.7 Hz, 2H, H^{10}), 1.52 – 1.45 (m, 2H, C^{8}), 1.46 (dd, J = 15.4, 10.9 Hz, 1H, H^{20}), 1.02 (s, 3H, H^{22}). ^{13}C NMR (151 MHz, DMSO-d_6, 298 K) δ 172.18 (1C, C^{12}), 171.21 (1C, C^{18}), 170.67 (1C, C^{15}), 162.63 (1C, C^{2}), 80.24 (1C, C^{24}), 72.86 (1C, C^{25}), 60.84 (1C, C^{7}), 59.08 (1C, C^{4}), 55.25 (1C, C^{6}), 51.09 (1C, C^{14}), 48.53 (1C, C^{17}), 39.73 (1C, C^{5}), 36.09 (1C, C^{20}), 35.00 (1C, C^{11}), 28.00 (1C, C^{8}), 27.90 (1C, C^{9}), 25.11 (1C, C^{10}), 24.30 (1C, C^{21}), 21.49 (1C, C^{23}), 19.47 (1C, C^{22}). ESI-HR-MS calcd. for $C_{20}H_{29}N_7O_4S$ $[M + H]^+$: *m/z* 464.2074. Found $[M + H]^+$:464.2083.

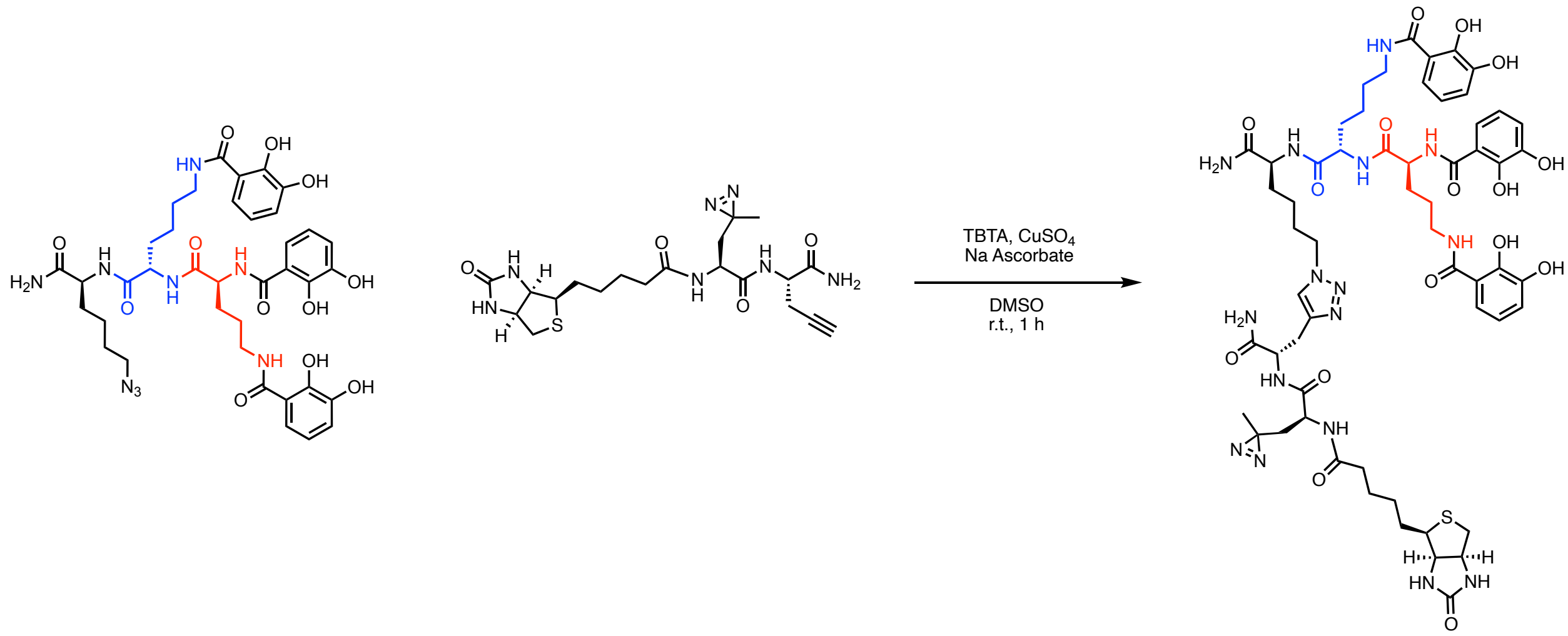

Scheme S4. CuAAC strategy for pull-down probe **KO-NH$_2$-azir-biotin**.

KO-NH$_2$-azir-biotin was synthesized by CuAAC. In a vial, 240 µL of a solution of 100 mM of **alkyne-azir-biotin** was mixed with 83 µL of a solution of 100 mM of **KO-NH$_2$ N$_3$**. 300 µL of DMSO was added and the solution was degassed with N_2 for 10 minutes. In a different vial, 110 µL of a 200 mM solution of $CuSO_4$ in water and 250 µL of a 100 mM of TBTA in DMSO were mixed, and 100 µL of DMSO was added. Then, 450 µL of a 200 mM solution of sodium ascorbate in water was added. The resulting solution was degassed with N_2 for 10 minutes. The two solutions were combined and stirred at room temperature under N_2 for 1 h. The reaction mixture was immediately purified by preparative HPLC (method F) and pure fractions were flash frozen upon collection to keep the compound from degrading, to yield **KO-NH$_2$-azir biotin** as a white solid (50%, 8.3 µmol).

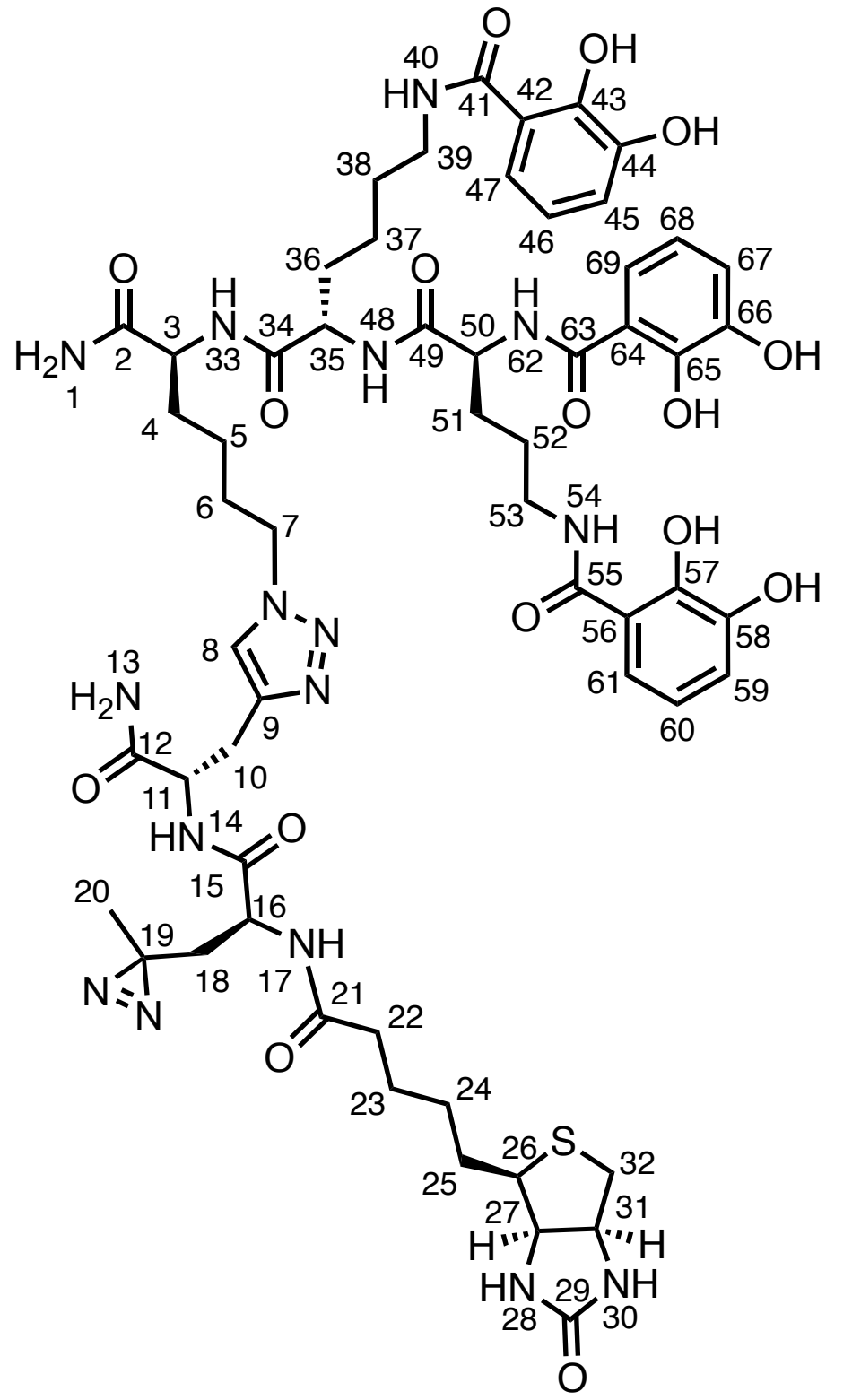

***N*,*N*'-((*S*)-5-(((*S*)-1-(((*S*)-1-amino-6-(4-((*S*)-3-amino-2-((*S*)-3-(3-methyl-3*H*-diazirin-3-yl)-2-(5-((3a*R*,4*R*,6a*S*)-2-oxohexahydro-1*H*-thieno[3,4-*d*]imidazol-4-yl)pentanamido)propanamido)-3-oxopropyl)-1*H*-1,2,3-triazol-1-yl)-1-oxohexan-2-yl)amino)-6-(2,3-dihydroxybenzamido)-1-oxohexan-2-yl)amino)-5-oxopentane-1,4-diyl)bis(2,3-dihydroxybenzamide)** (**KO-NH_2-azir-biotin**). ^{1}H NMR (600 MHz, DMSO-d_6, 298 K) δ 8.78 (q, J = 7.4, 6.2 Hz, 3H, H^{40}, H^{54}, H^{62}), 8.21 (d, J = 8.3 Hz, 1H, H^{33}), 8.12 (d, J = 8.8 Hz, 2H, H^{14}, H^{17}), 7.85 (d, J = 8.7 Hz, 1H, H^{48}), 7.76 (s, 1H, H^8), 7.42 (dd, J = 8.3, 1.6 Hz, 1H, H^{69}), 7.38 – 7.36 (m, 1H, H^1), 7.35 – 7.31 (m, 1H, H^{13}), 7.30 – 7.23 (m, 2H, H^{47}, H^{61}), 7.14 – 7.10 (m, 1H, H^{13}), 7.08 – 7.03 (m, 1H, H^1), 6.95 – 6.86 (m, 3H, H^{45}, H^{59}, H^{67}), 6.75 – 6.60 (m, 3H, H^{46}, H^{60}, H^{68}), 4.62 – 4.51 (m, 1H, H^{50}), 4.38 (td, J = 8.6, 5.2 Hz, 1H, H^{11}), 4.30 (dd, J = 8.1, 5.2 Hz, 1H, H^{31}), 4.24 (t, J = 7.5 Hz, 2H, H^7), 4.17 (td, J = 8.1, 5.8 Hz, 1H, H^3), 4.11 (ddt, J = 11.7, 8.3, 4.6 Hz, 2H, H^{16}, H^{27}), 3.31 (q, J = 6.9 Hz, 2H, H^{53}), 3.24 (dq, J = 14.0, 6.9 Hz, 2H, H^{39}), 3.14 – 2.99 (m, 2H, H^{10}, H^{26}), 2.91 (dd, J = 15.4, 8.9 Hz, 1H, H^{10}), 2.85 – 2.77 (m, 1H, H^{32}), 2.60 – 2.64 (m, 1H, H^{32}), 2.16 (d, J = 8.4 Hz, 2H, H^{22}), 1.83 (dd, J = 15.0, 4.3 Hz, 1H, H^{18}), 1.80 – 1.58 (m, 8H, H^4, H^6, H^{38}, H^{52}), 1.56 – 1.45 (m, 8H, H^{18}, H^{23}, H^{25}, H^{38}), 1.39 – 1.26 (m, 4H, H^{24}, H^{37}), 1.26 – 1.12 (m, 4H, H^5, H^{51}), 0.99 (s, 3H, H^{20}). ^{13}C NMR (151 MHz, DMSO-d_6, 298 K) δ ^{13}C NMR (151 MHz, DMSO) δ 173.42 (1C, C^2), 172.49 (1C, C^{21}, 172.35 (1C, C^{12}), 171.39 (2C, C^{34}, C^{49}), 170.75 (1C, C^{15}), 169.77 (2C, C^{41}, C^{55}), 168.95 (1C, C^{63}), 162.83 (1C, C^{29}), 149.74 (2C, C^{44}, C^{58}), 148.72 (1C, C^{66}), 146.19 (2C, C^{42}, C^{56}), 146.05 (1C, C^{64}), 142.82 (1C, C^9), 122.58 (1C, C^8), 118.81 (1C, C^{67}), 118.72 (2C, C^{45}, C^{59}), 118.30 (1C, C^{69}), 118.08 (1C, C^{68}) 117.84 (2C, C^{46}, C^{60}), 117.08 (2C, C^{47}, C^{61}), 115.68 (2C, C^{43}, C^{57}), 114.89 (1C, C^{65}), 60.99 (1C, C^{27}), 59.23 (1C, C^{31}), 55.40 (1C, C^{26}), 52.84 (1C, H^{50}), 52.67 (1C, C^7), 52.21 (1C, C^{11}), 52.05 (1C, C^{16}), 49.06 (1C, C^{26}), 48.80 (1C, C^{27}), 40.06 (1C, C^{32}), 38.90 (1C, C^{39}), 38.68 (1C, C^{53}), 35.93 (1C, C^{18}), 35.07 (1C, C^{22}), 31.61 (2C, C^4, C^{52}), 29.44 (2C, C^6, C^{38}), 28.13 (1C, C^{10}), 28.04 (2C, C^{23}, C^{24}), 25.21 (1C, C^{23}, C^{25}), 24.42 (1C, C^{19}), 22.31 (1C, C^5, C^{51}), 19.64 (1C, C^{20}). ESI-HR-MS calcd. for $C_{58}H_{77}N_{16}O_{16}S$ $[M + H]^+$: m/z 1285.5419, $[M+2H]^{2+}$: m/z 662.2525. Found $[M + H]^+$:1285.5453, $[M+2H]^{2+}$: 662.2495.

1.6 Analytical characterization of ligands

1.6.1 Analytical HPLC traces

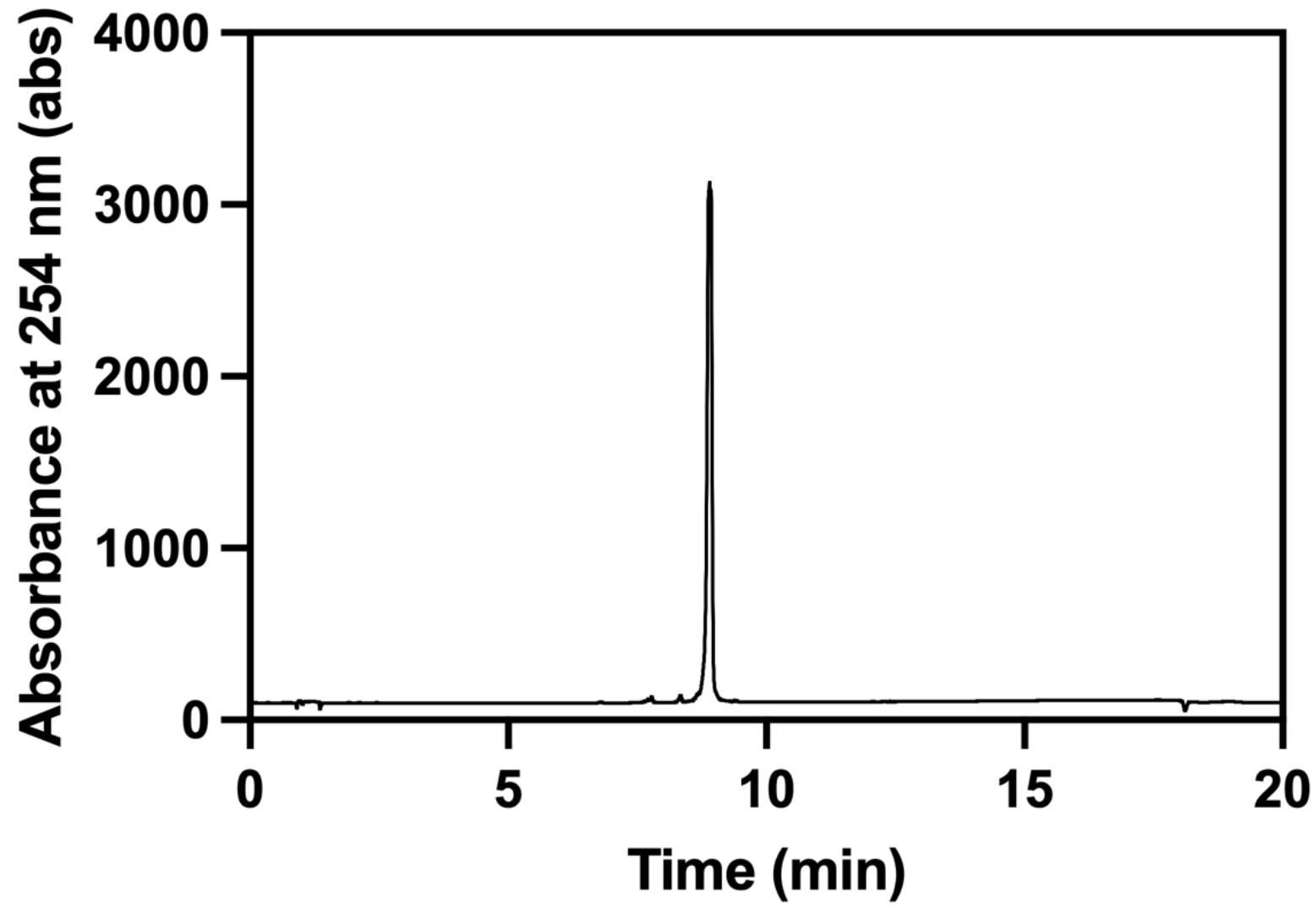

Figure S1. HPLC chromatogram of **KK-COOH**. Retention time: 8.93 min. (Method A).

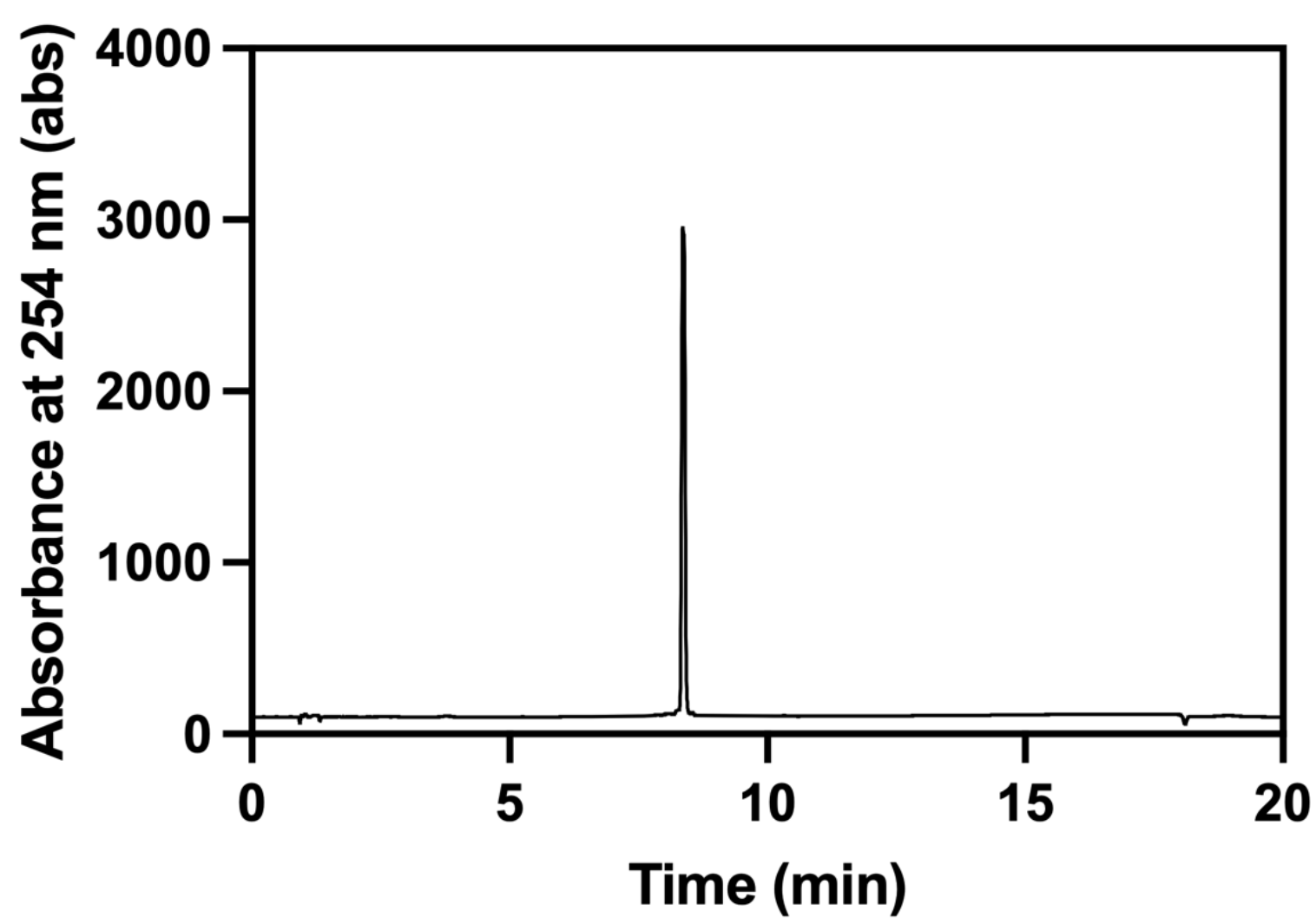

Figure S2. HPLC chromatogram of **KO-NH_2**. Retention time: 8.38 min. (Method A).

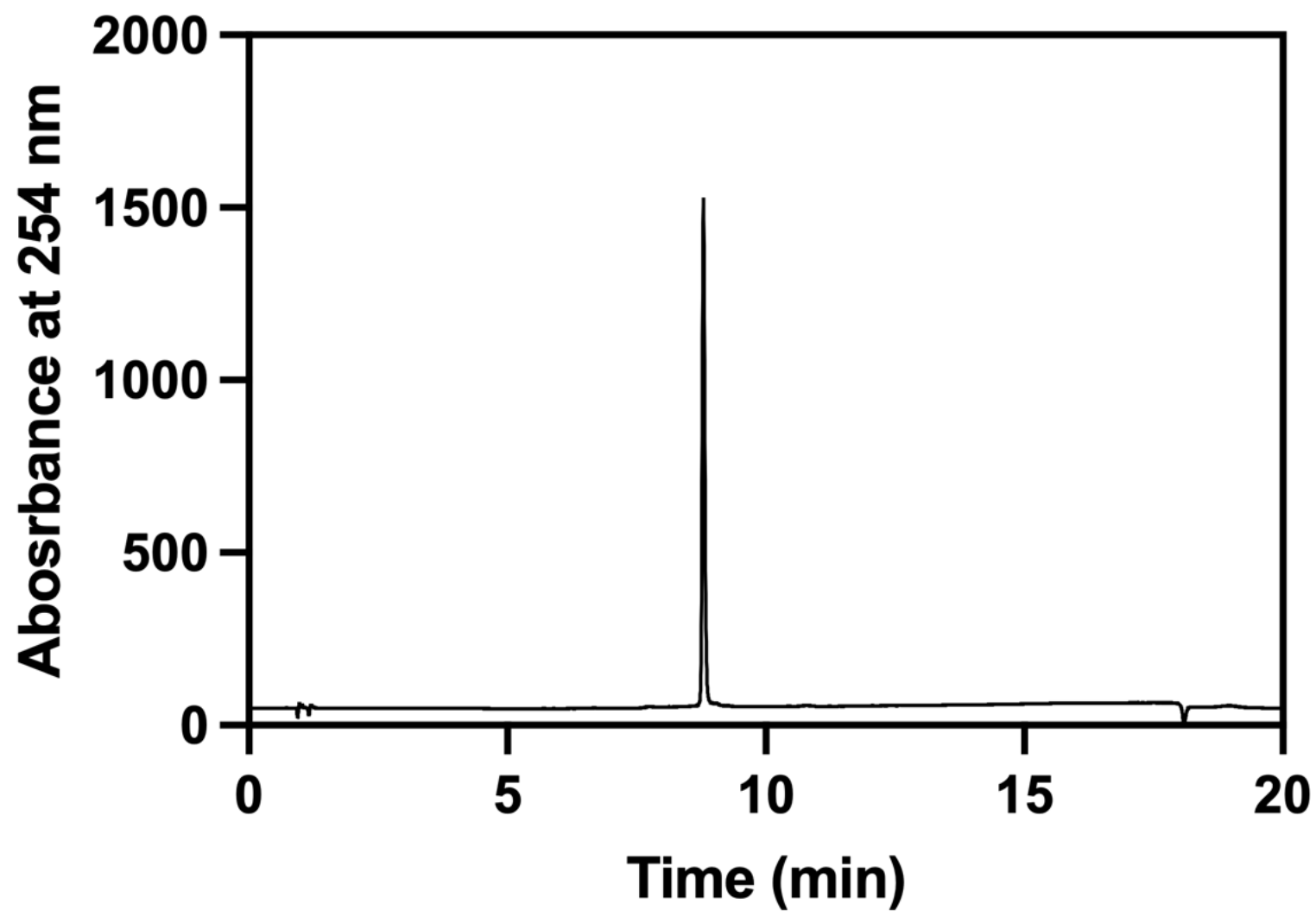

Figure S3. HPLC chromatogram of **KO-COOH**. Retention time: 8.80 min. (Method A).

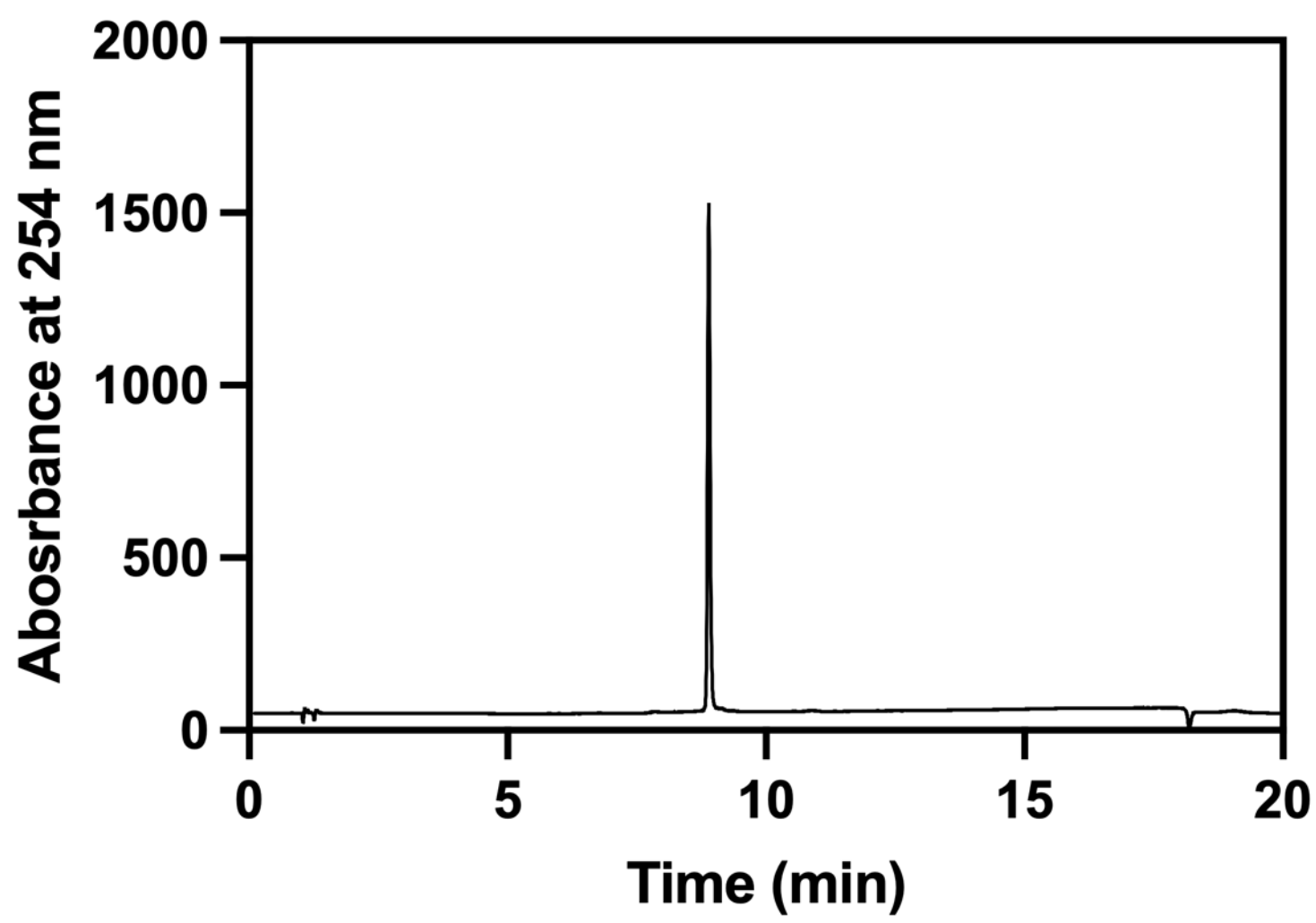

Figure S4. HPLC chromatogram of **OK-COOH**. Retention time: 8.89 min. (Method A).

1.6.2 *HRMS Spectra*

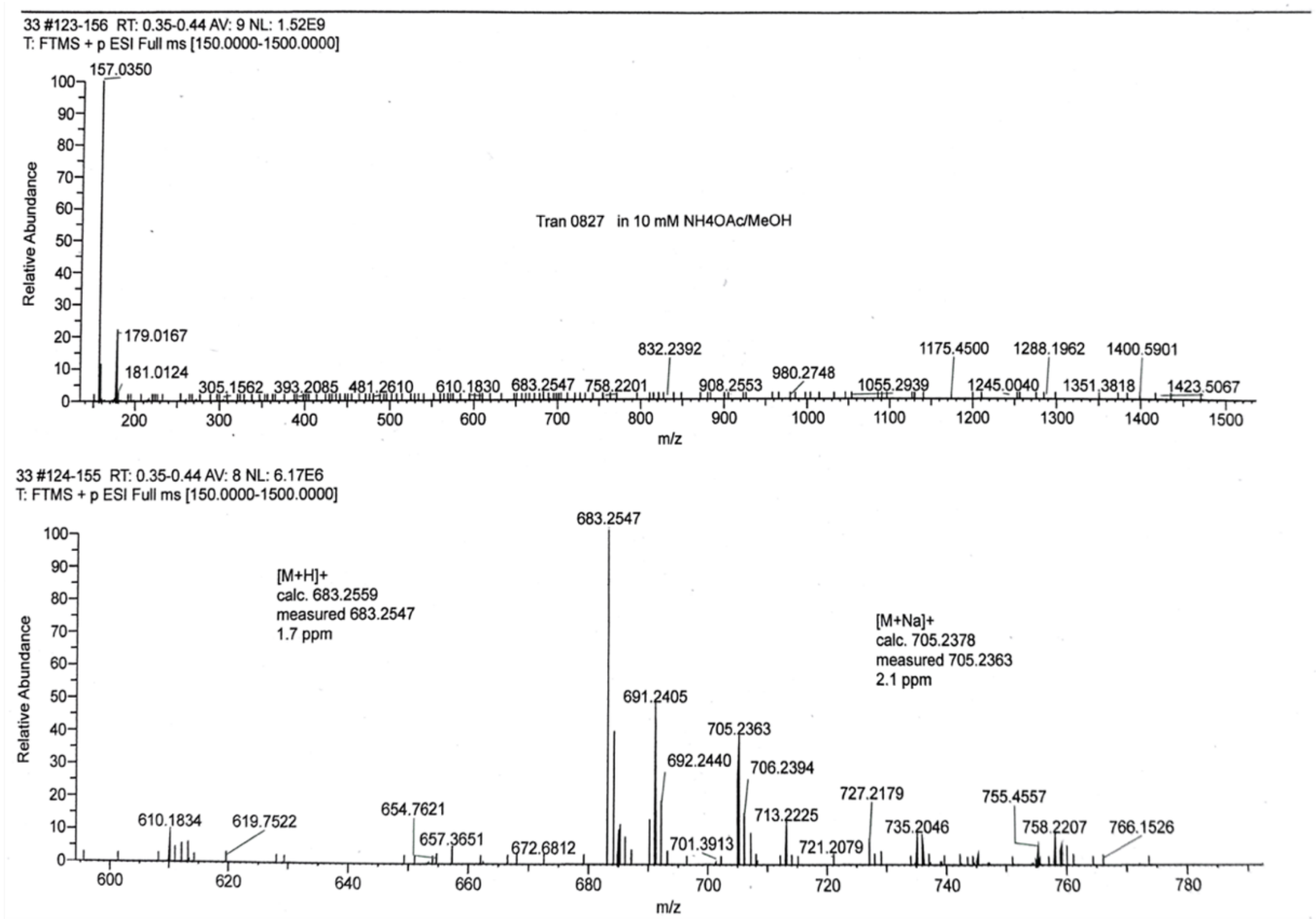

Figure S5. ESI (+) HR-MS spectrum of **KK-COOH**. ESI-HRMS calcd. for $C_{33}H_{32}N_4O_{12}$ [M + H]$^+$: *m/ z* 683.2559. Found 683.2547.

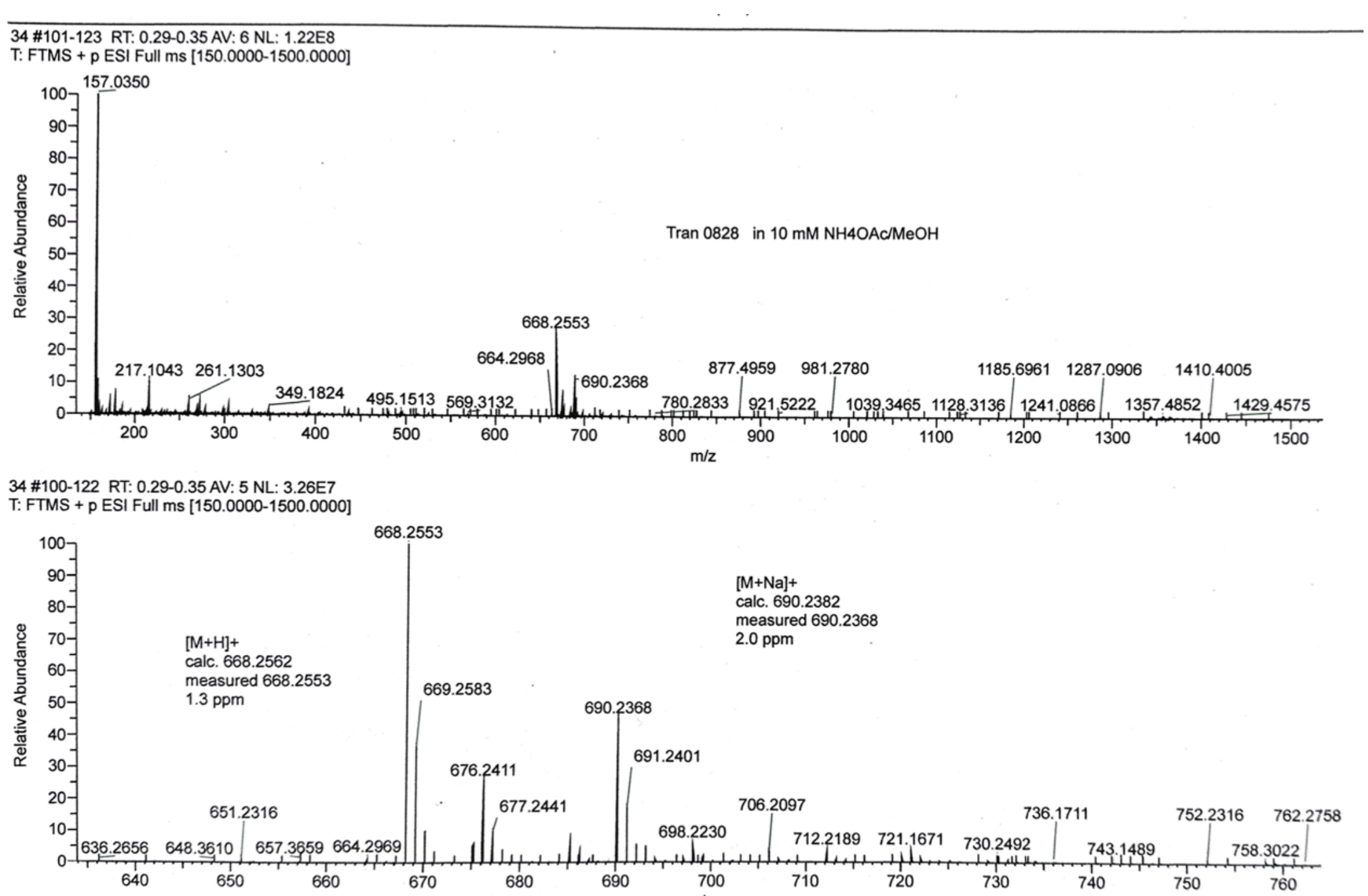

Figure S6. ESI (+) HR-MS spectrum of **KO-NH₂**. ESI-HRMS calcd. for $C_{32}H_{37}N_5O_{11}$ [M + H]$^+$: *m/ z* 668.2562. Found 668.2553.

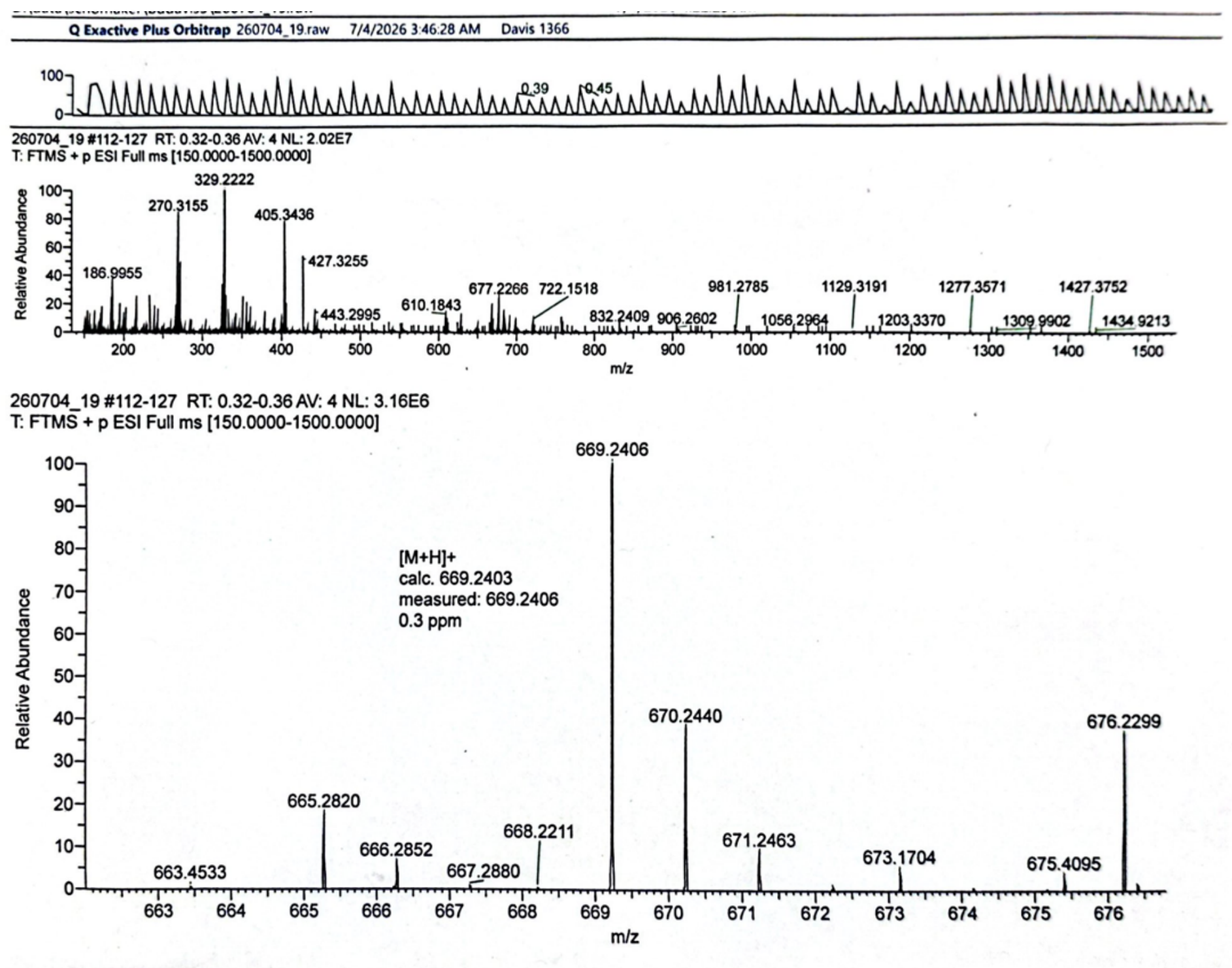

Figure S7. ESI (+) HR-MS spectrum of **KO-COOH**. ESI-HR-MS calcd. for $C_{32}H_{36}N_4O_{12}$ $[M + H]^+$: *m/ z* 669.2403. Found 669.2406.

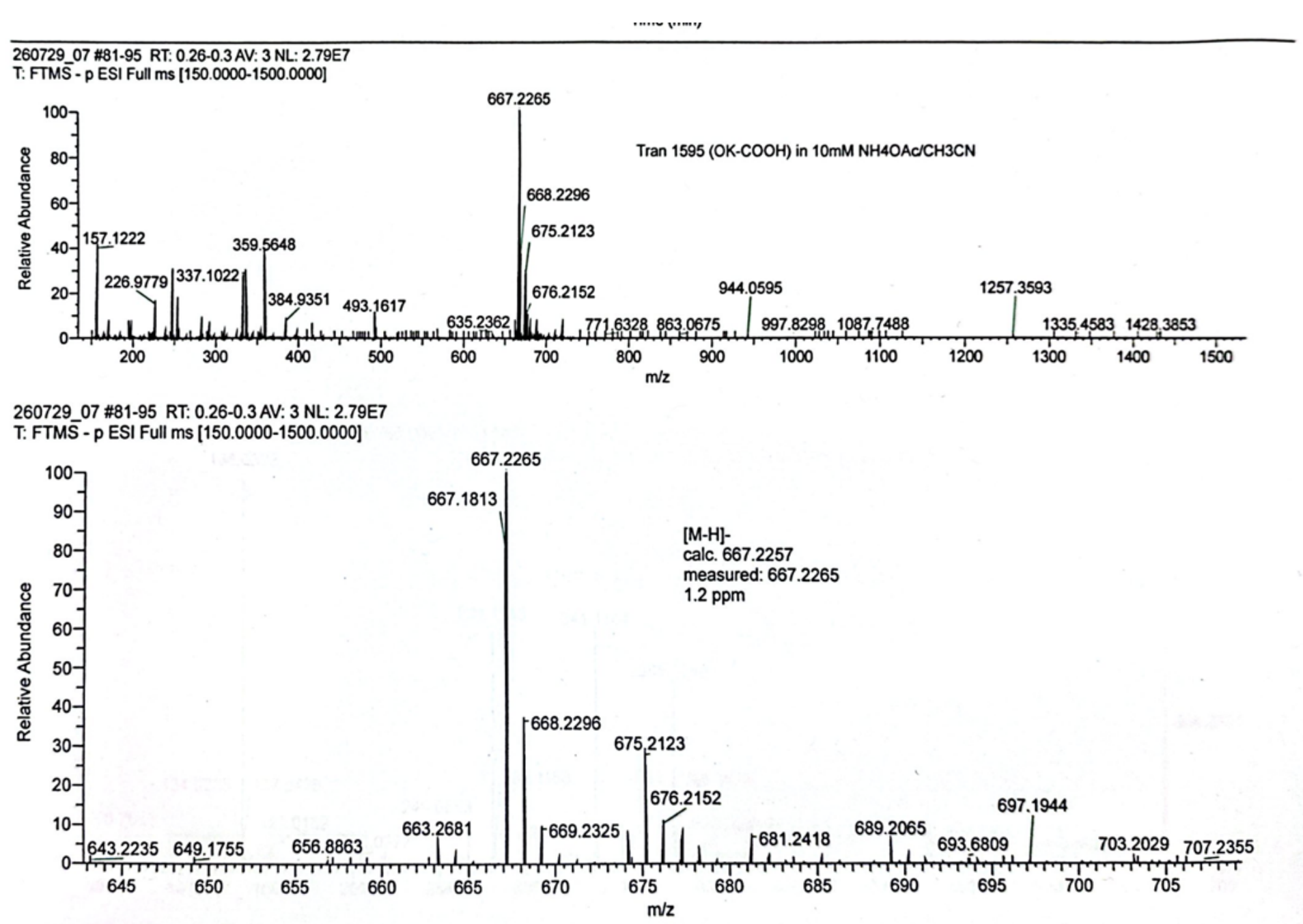

Figure S8. ESI (-) HR-MS spectrum of **OK-COOH**. ESI-HR-MS calcd. for $C_{32}H_{36}N_4O_{12}$ $[M - H]^-$: *m/ z* 667.2257. Found 667.2265.

1.6.3 NMR Spectra

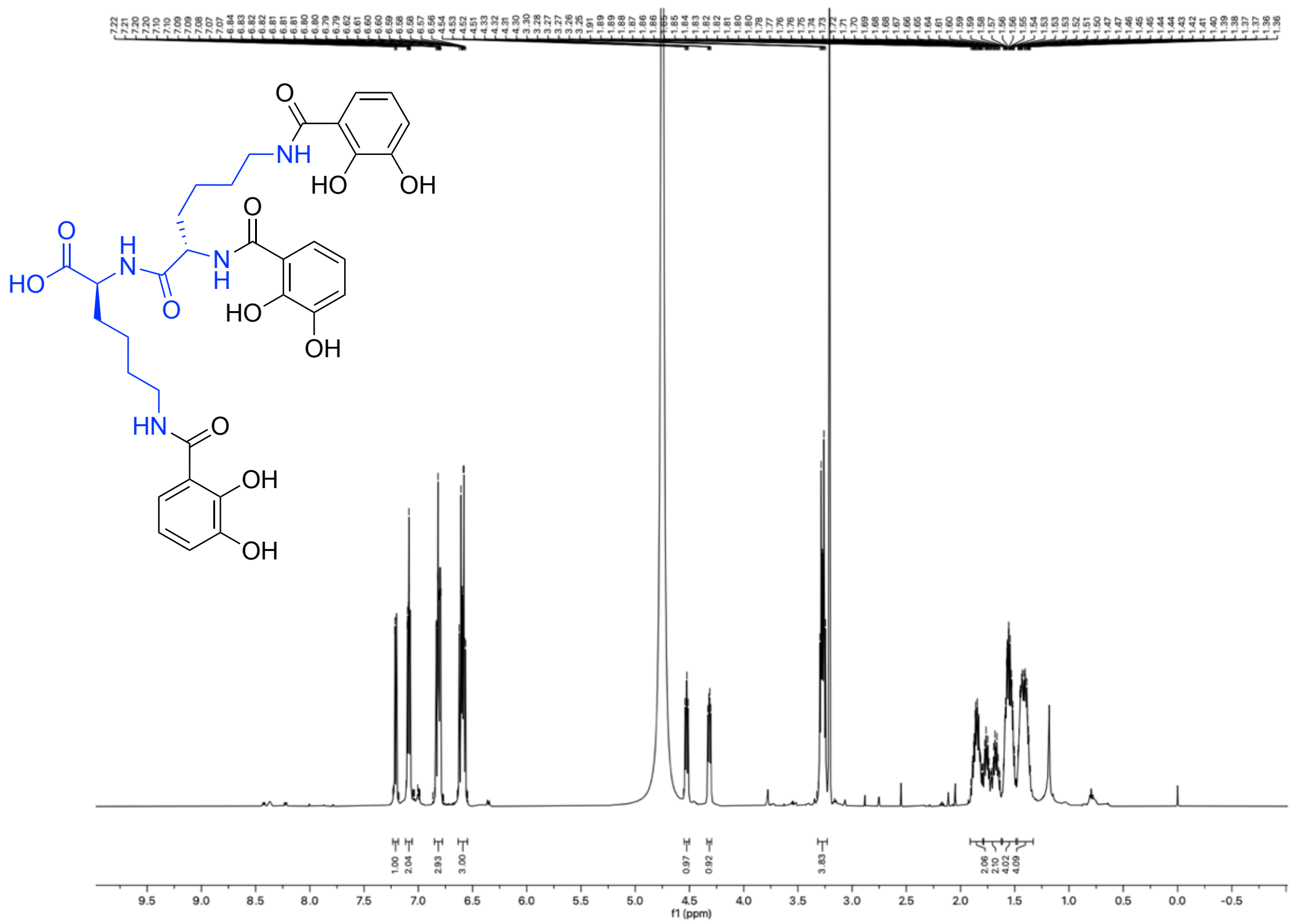

Figure S9. ^{1}H NMR (500 MHz, MeOD-*d4*, 298 K) of **KK-COOH**.

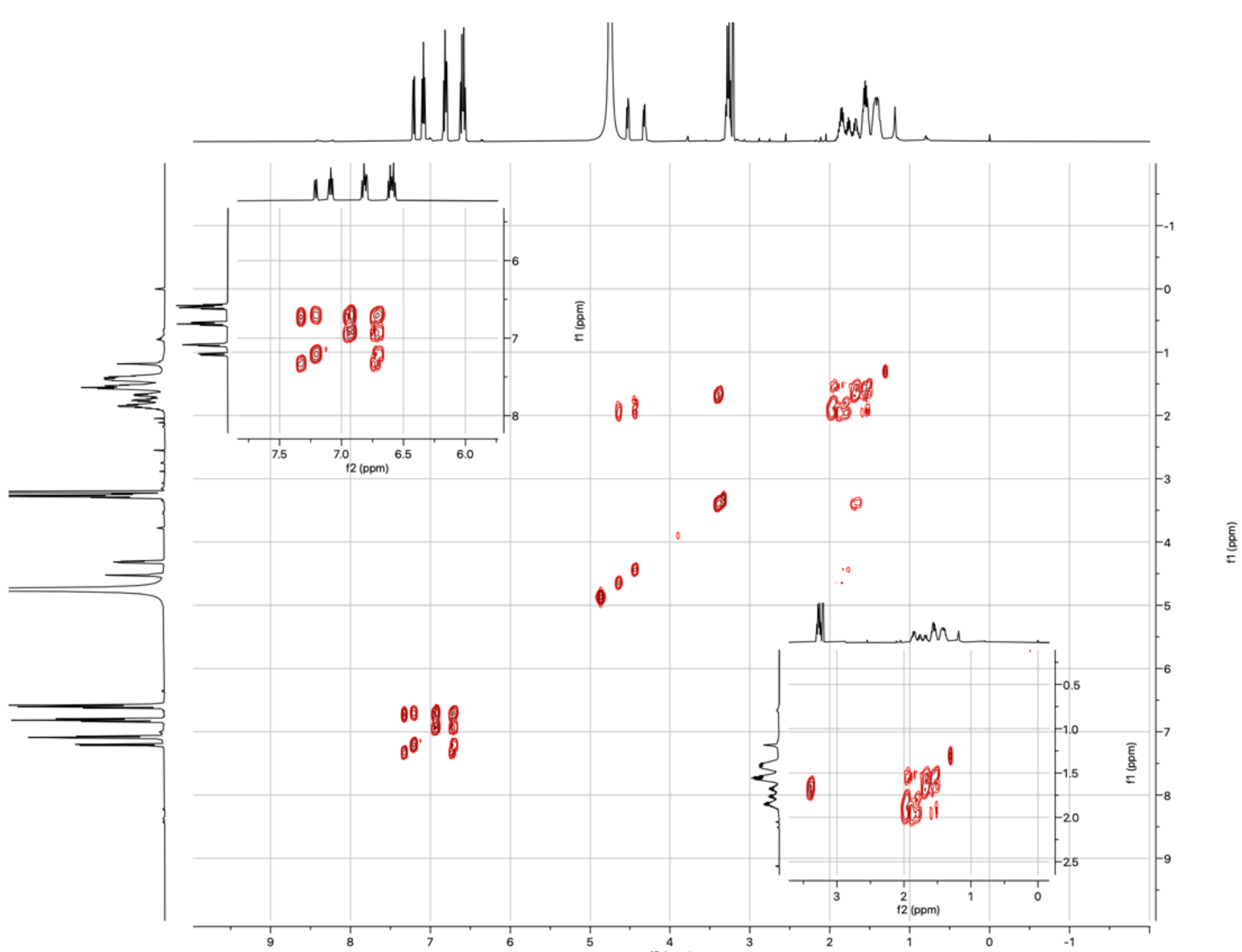

Figure S10. ^{1}H-^{1}H COSY NMR (500 MHz, MeOD-*d4*, 298 K) of **KK-COOH**.

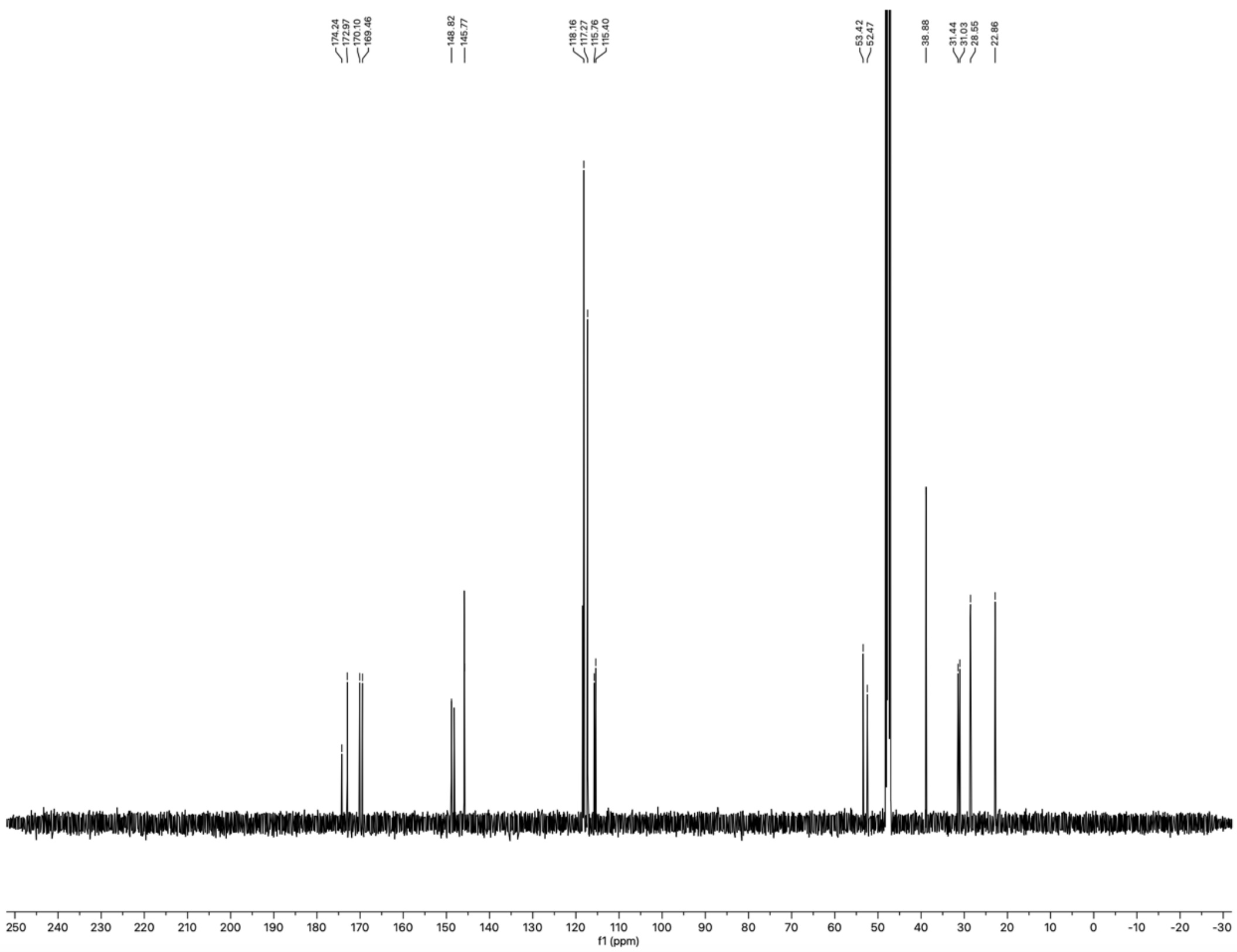

Figure S11. $^{13}C\{^{1}H\}$ NMR (126 MHz, MeOD-*d4*, 298 K) of **KK-COOH**.

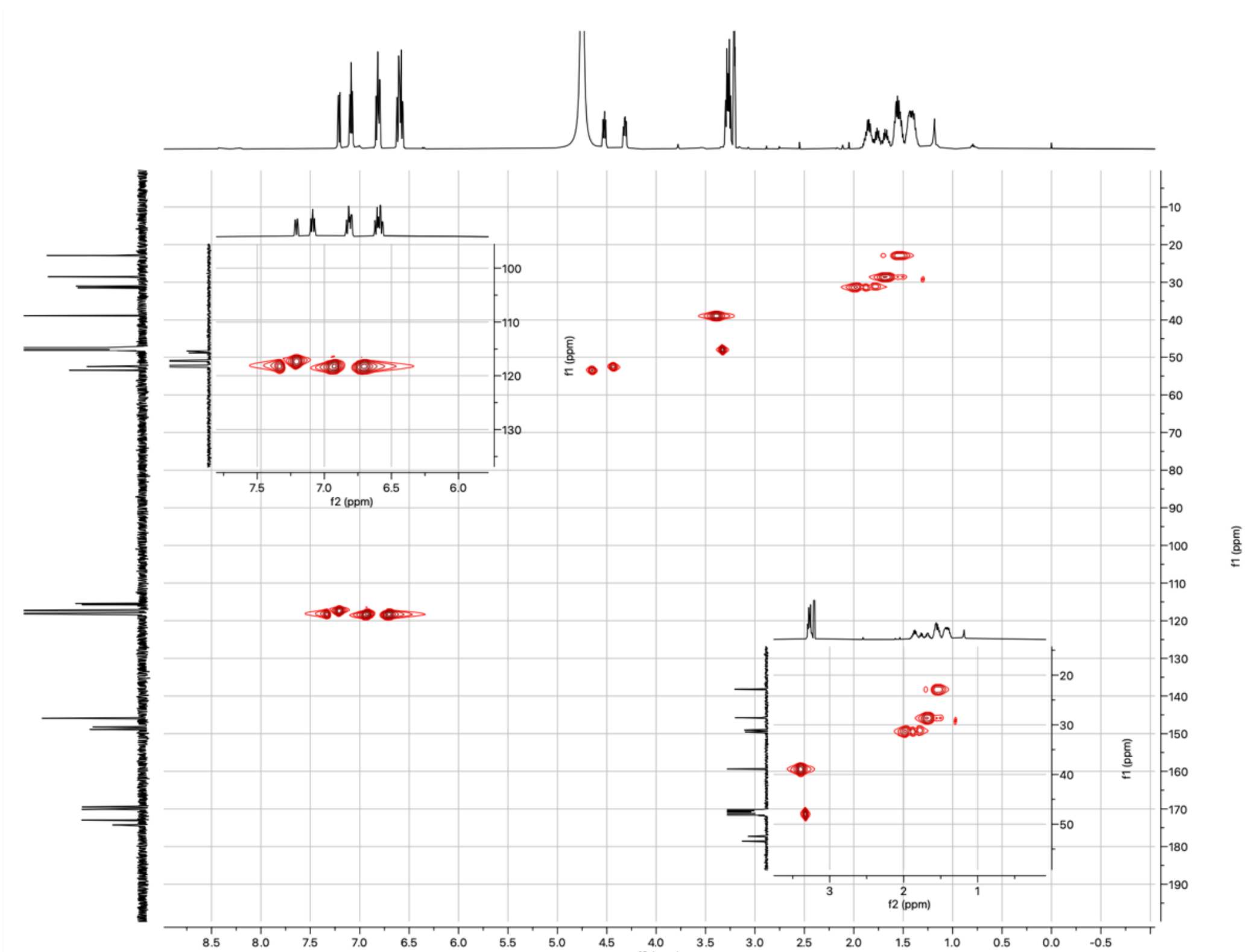

Figure S12. ^{1}H-^{13}C HSQC NMR (500 MHz, MeOD-*d4*, 298 K) of **KK-COOH**.

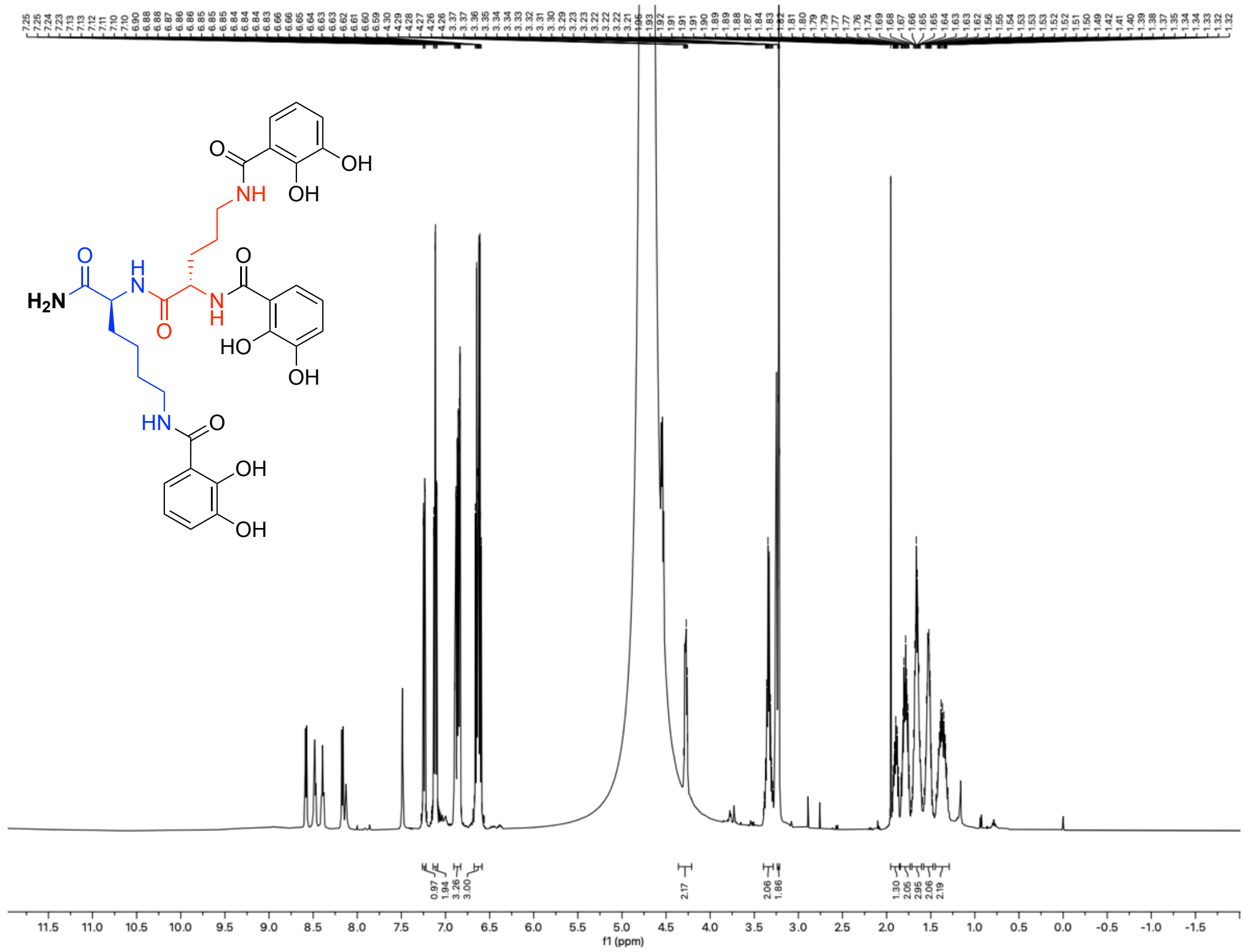

Figure S13. ^{1}H NMR (500 MHz, MeOD-*d4*, 298 K) of **KO-NH$_2$**.

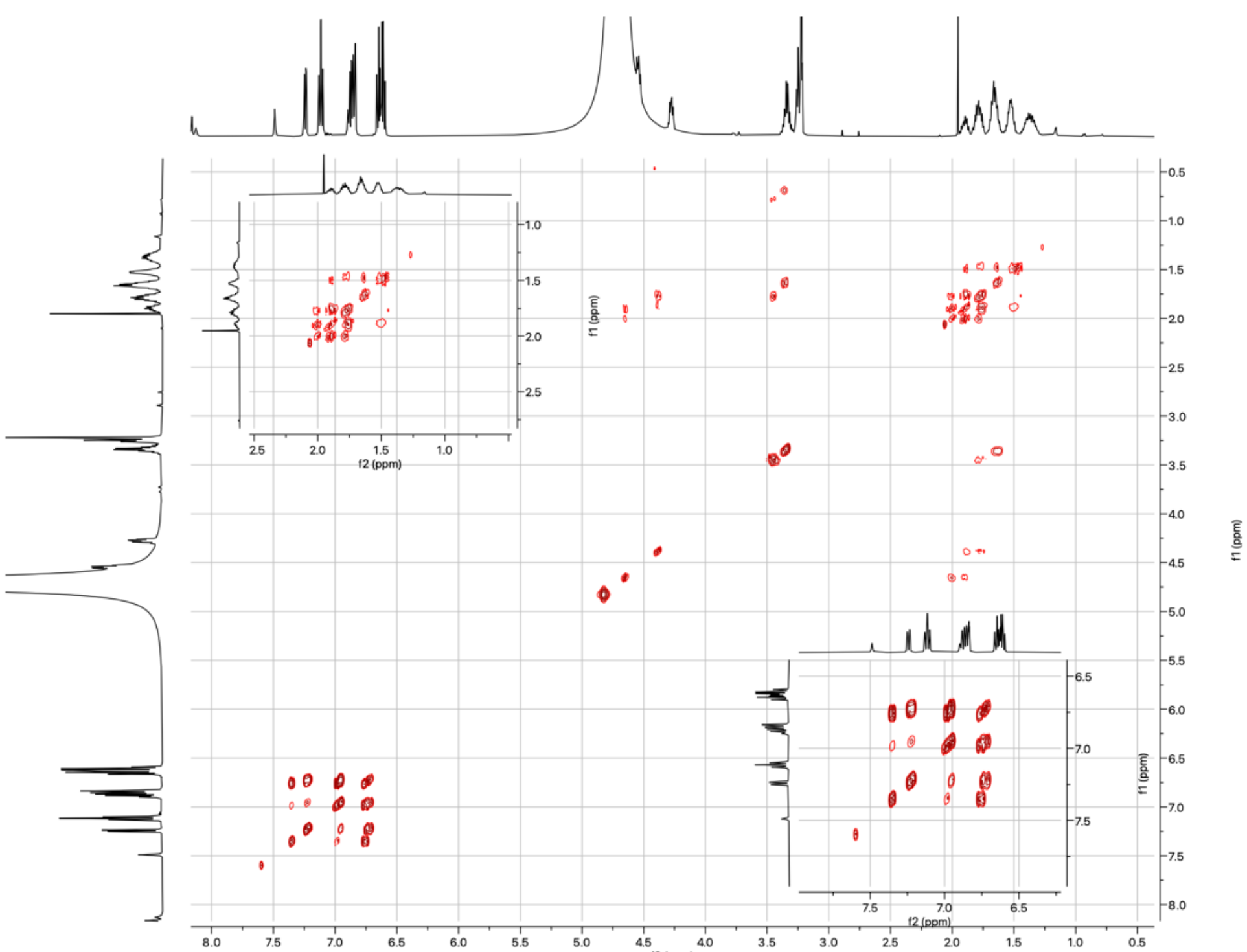

Figure S14. ^{1}H-^{1}H COSY NMR (500 MHz, MeOD-*d4*, 298 K) of **KO-NH$_2$**.

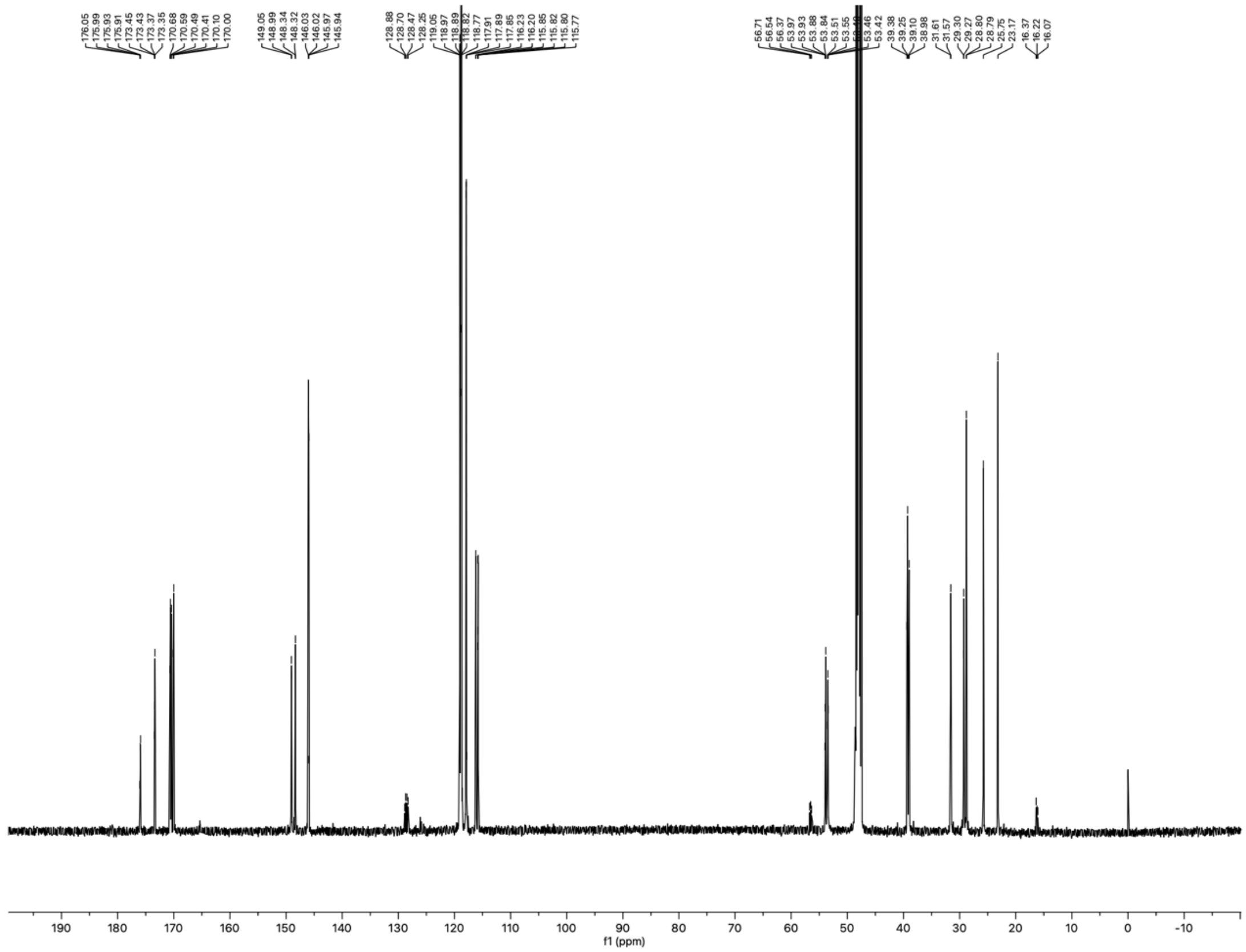

Figure S15. $^{13}C\{^{1}H\}$ NMR (126 MHz, MeOD-*d4*, 298 K) of **KO-NH$_2$.**

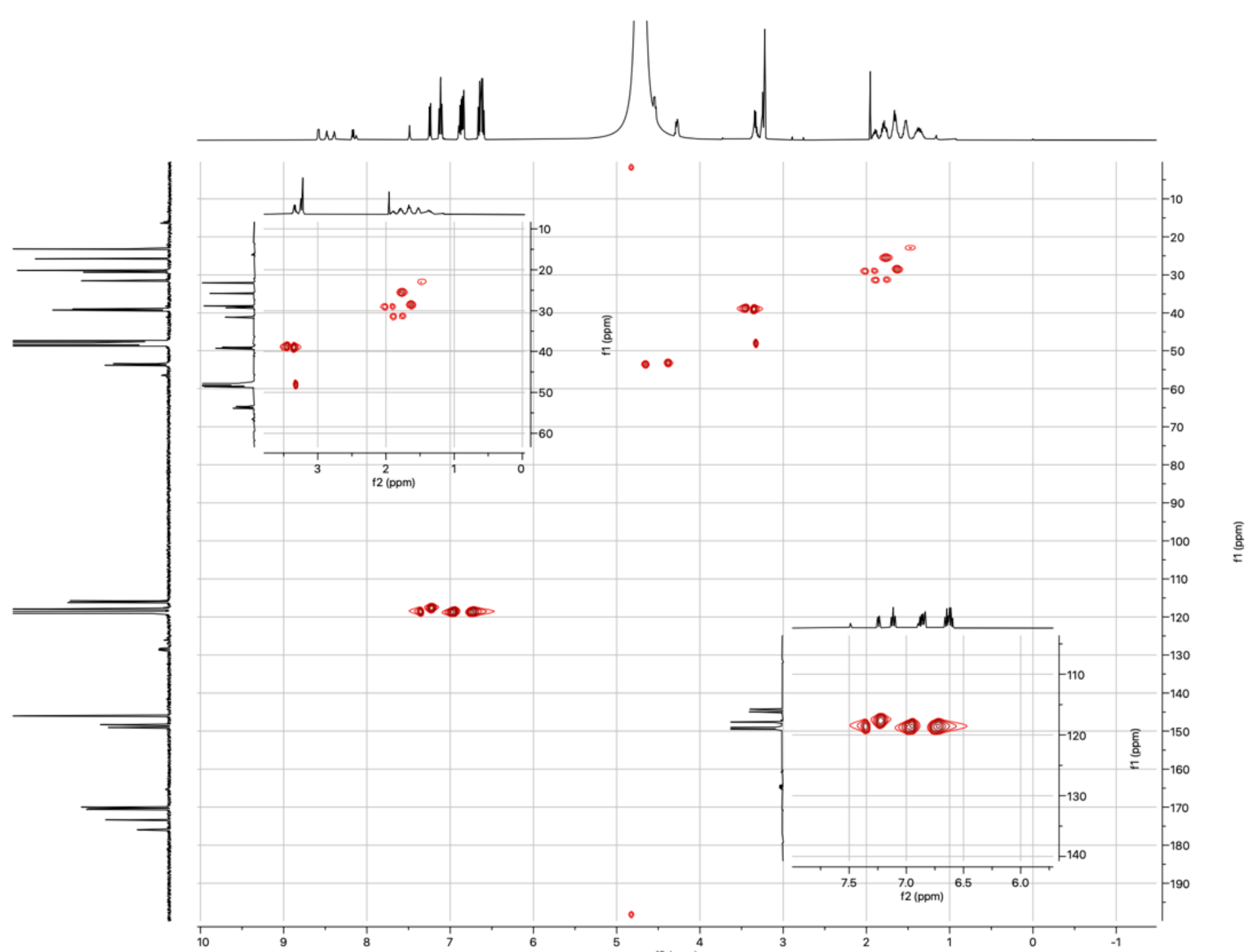

Figure S16. ^{1}H-^{13}C HSQC NMR (500 MHz, MeOD-*d4*, 298 K) of **KO-NH$_2$.**

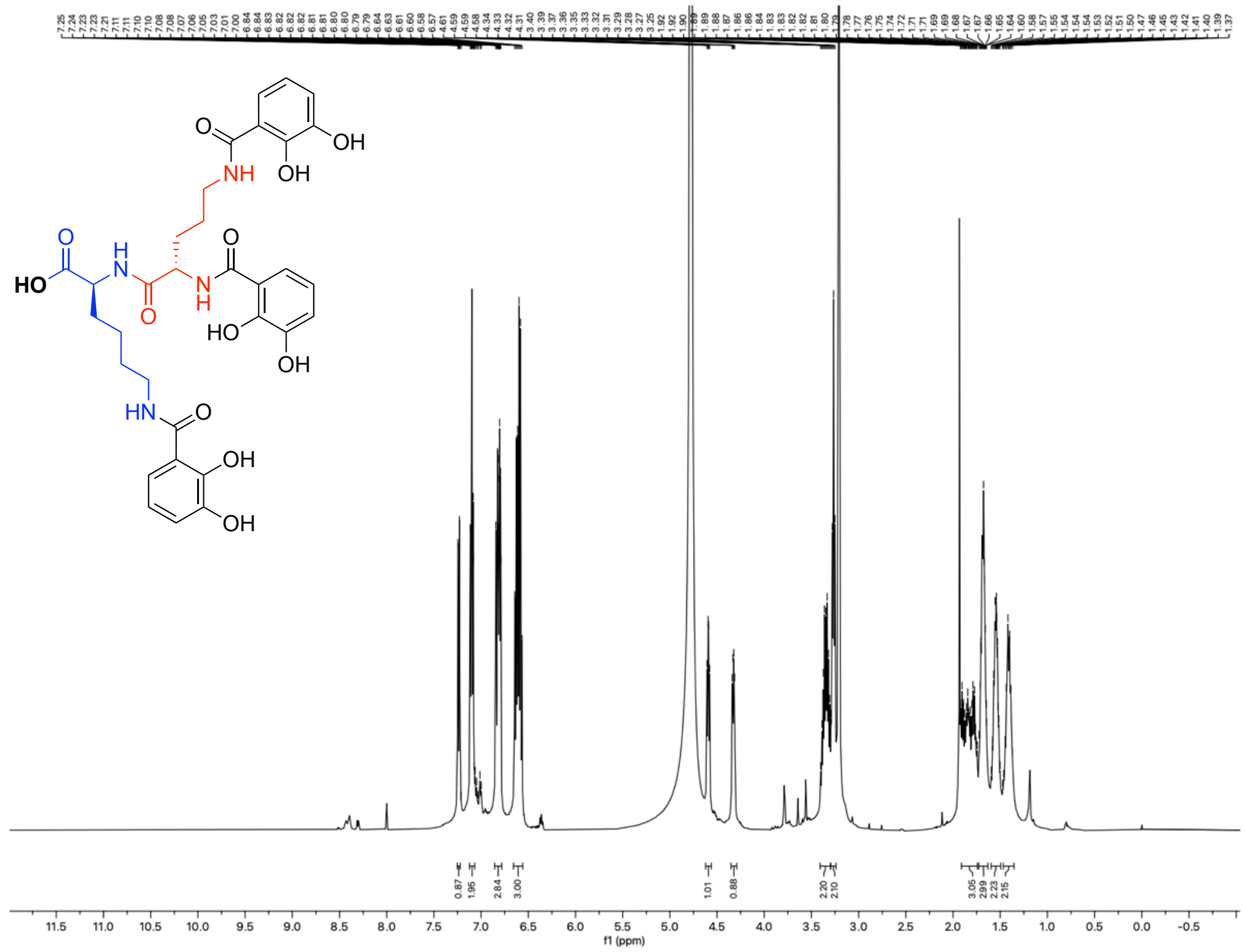

Figure S17. ^{1}H NMR (500 MHz, MeOD-*d4*, 298 K) of **KO-COOH.**

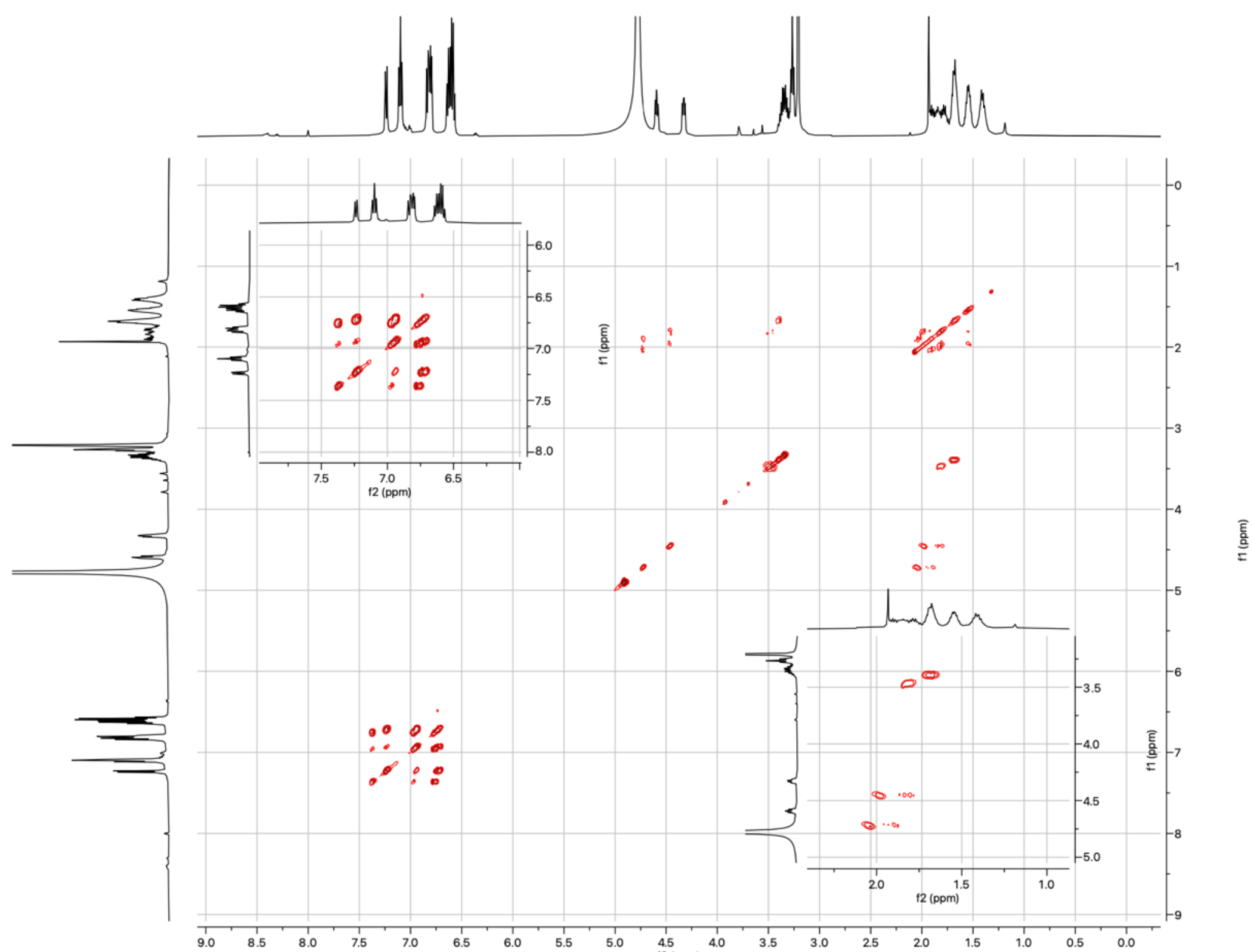

Figure S18. ^{1}H-^{1}H COSY NMR (500 MHz, MeOD-*d4*, 298 K) of **KO-COOH.**

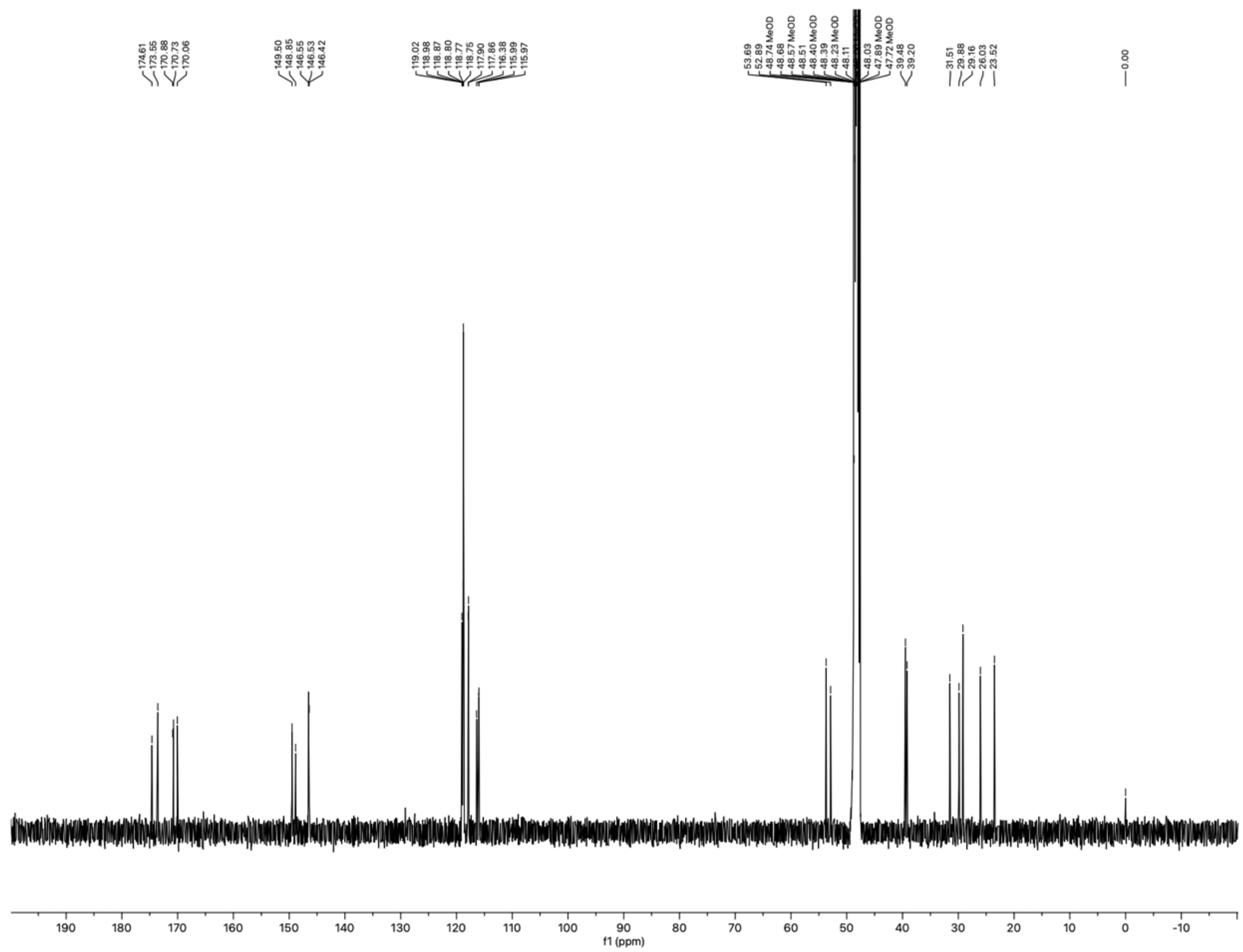

Figure S19. $^{13}C\{^{1}H\}$ NMR (126 MHz, MeOD-*d4*, 298 K) of **KO-COOH.**

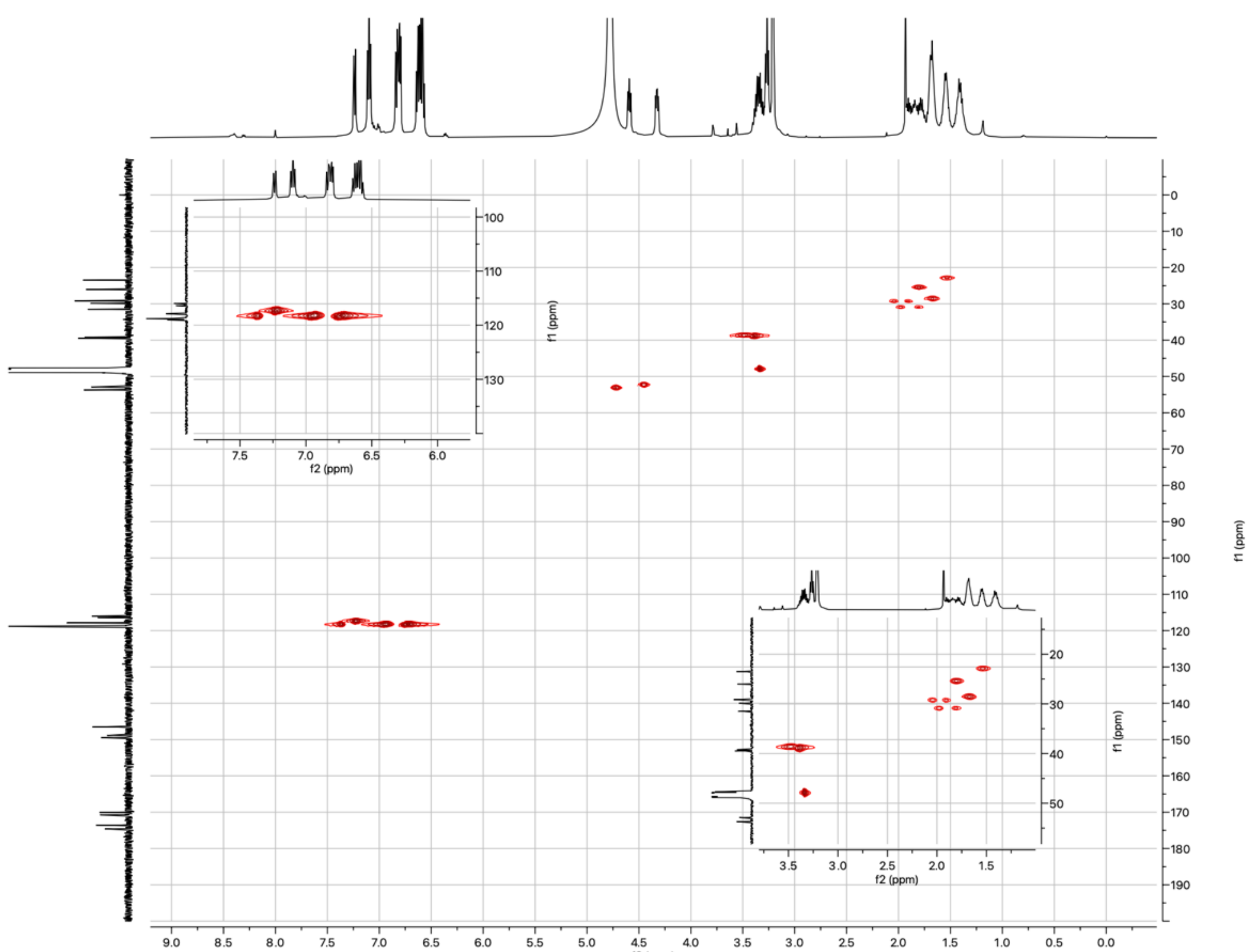

Figure S20. ^{1}H-^{13}C HSQC NMR (500 MHz, MeOD-*d4*, 298 K) of **KO-COOH.**

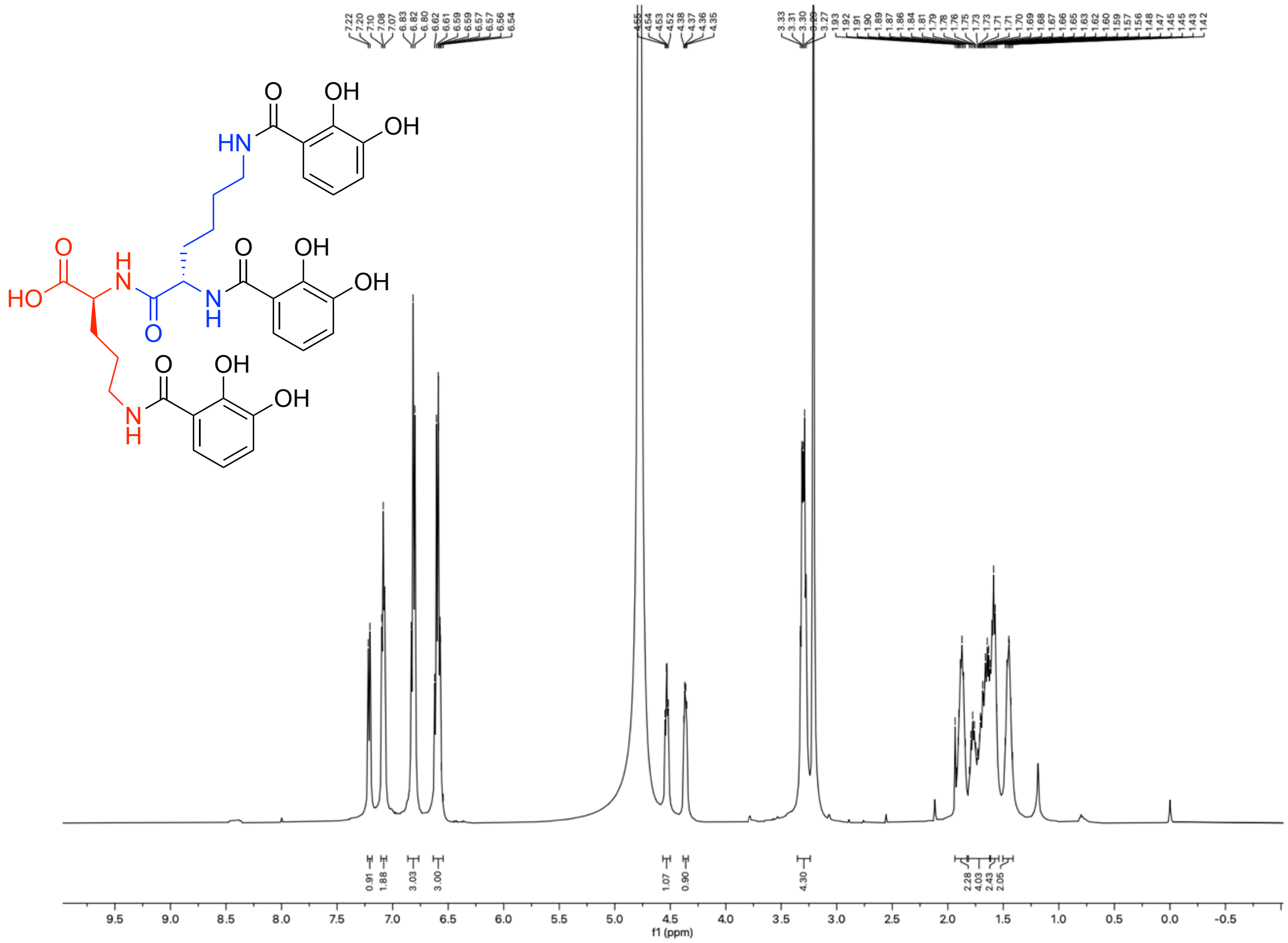

Figure S21. ^{1}H NMR (500 MHz, MeOD-*d4*, 298 K) of **OK-COOH.**

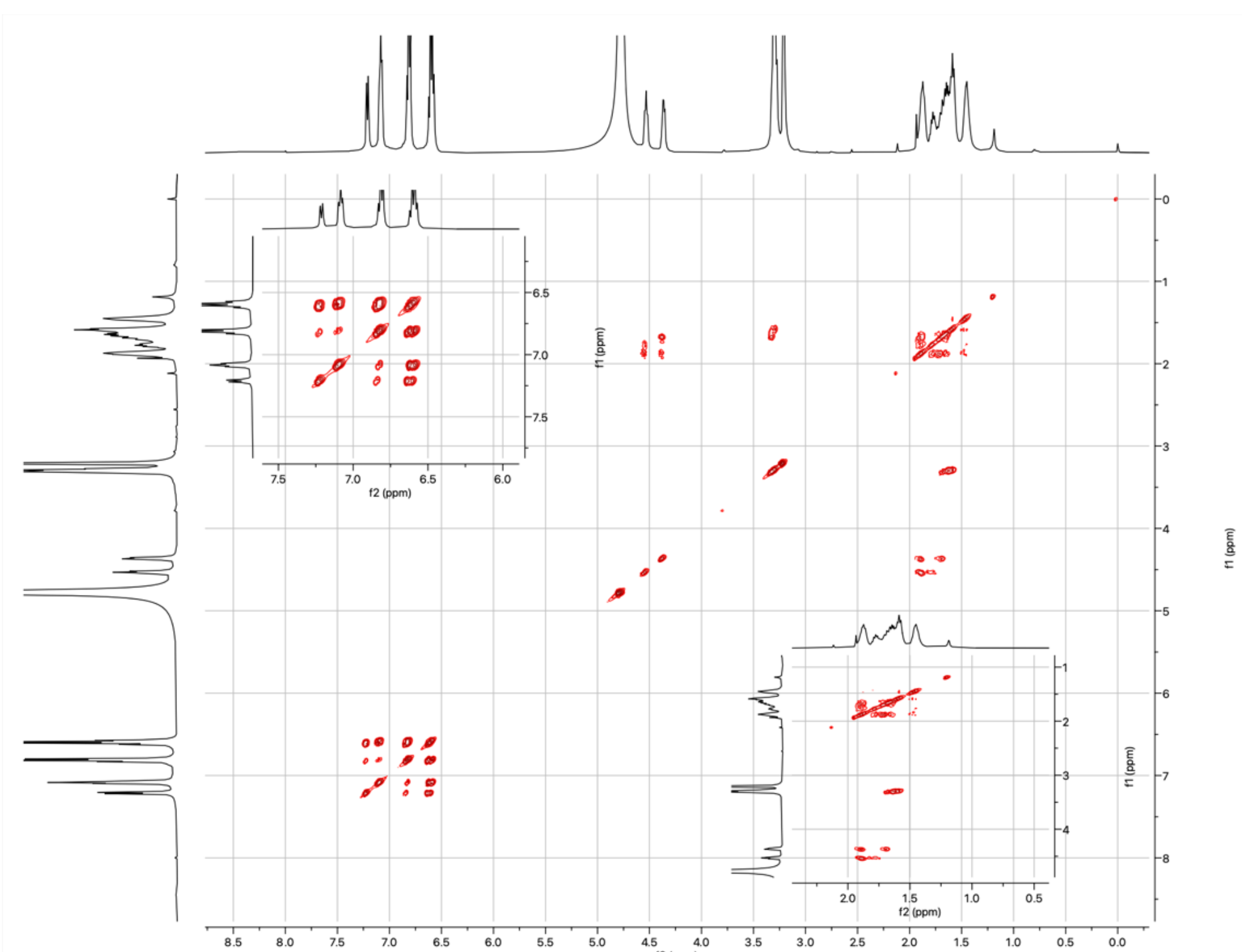

Figure S22. ^{1}H-^{1}H COSY NMR (500 MHz, MeOD-*d4*, 298 K) of **OK-COOH.**

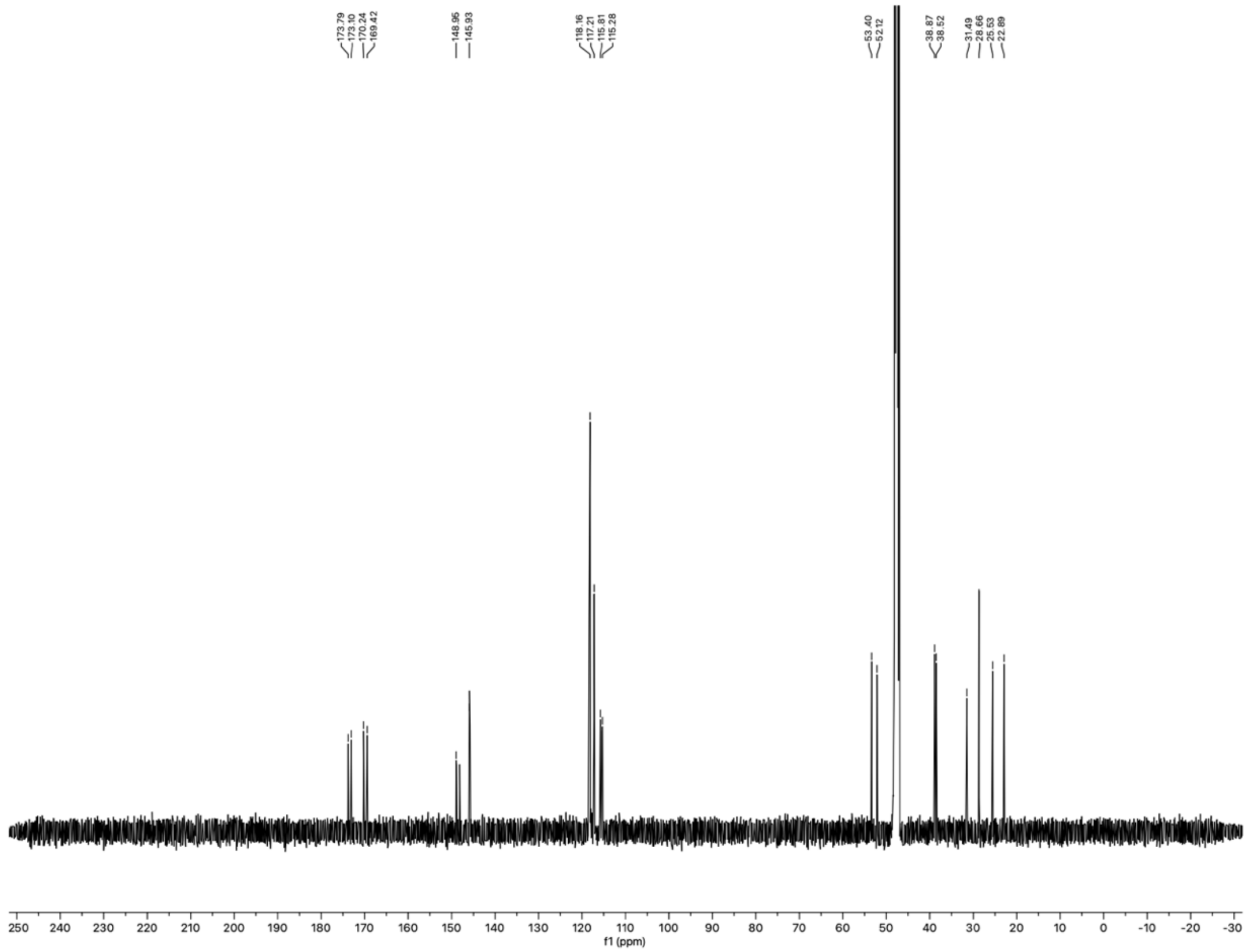

Figure S23. $^{13}C\{^{1}H\}$ NMR (126 MHz, MeOD-*d4*, 298 K) of **OK-COOH.**

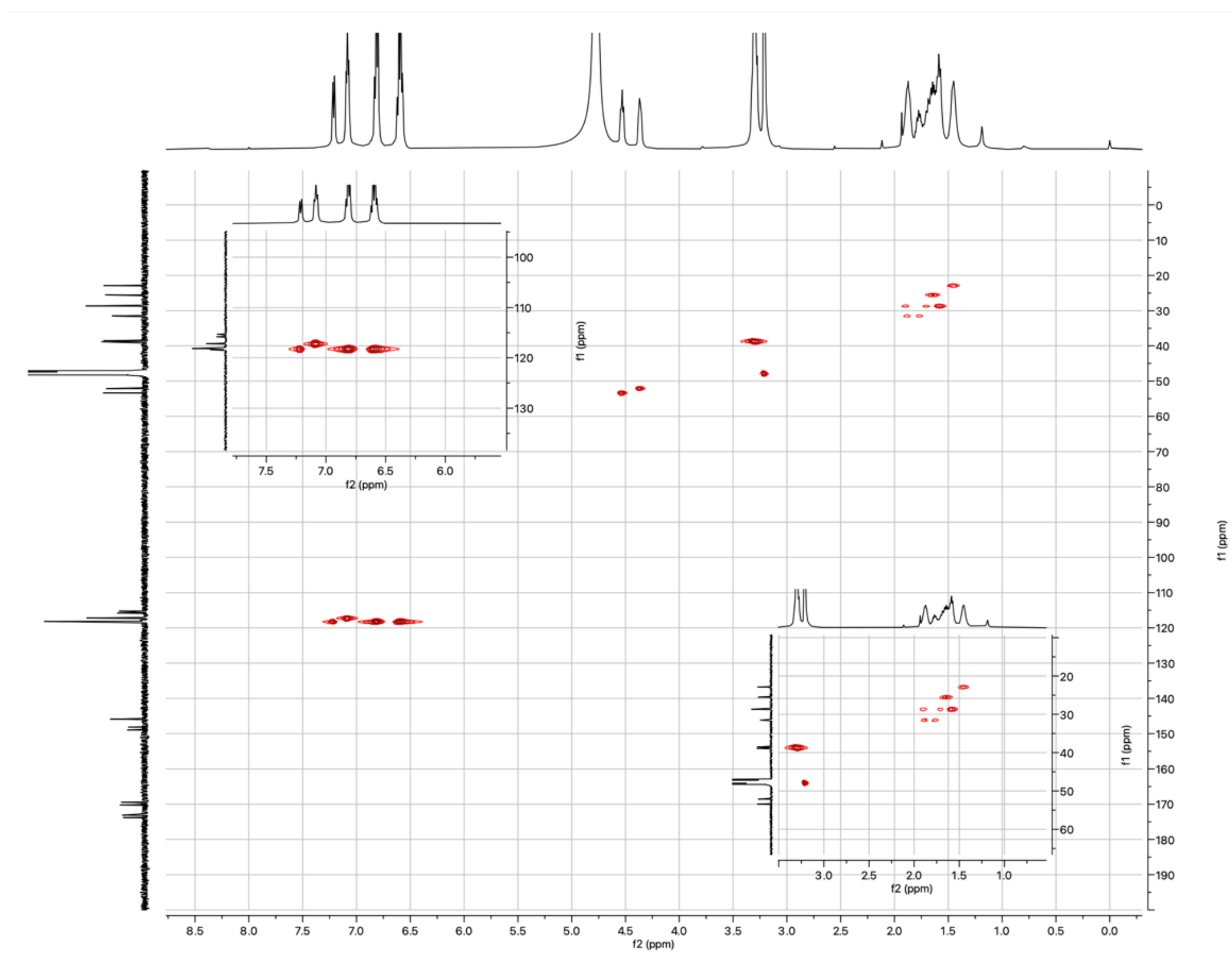

Figure S24. ^{1}H-^{13}C HSQC NMR (500 MHz, MeOD-*d4*, 298 K)) **of OK-COOH.**

1.7 Analytical characterization of conjugates and probes

1.7.1 Analytical HPLC traces

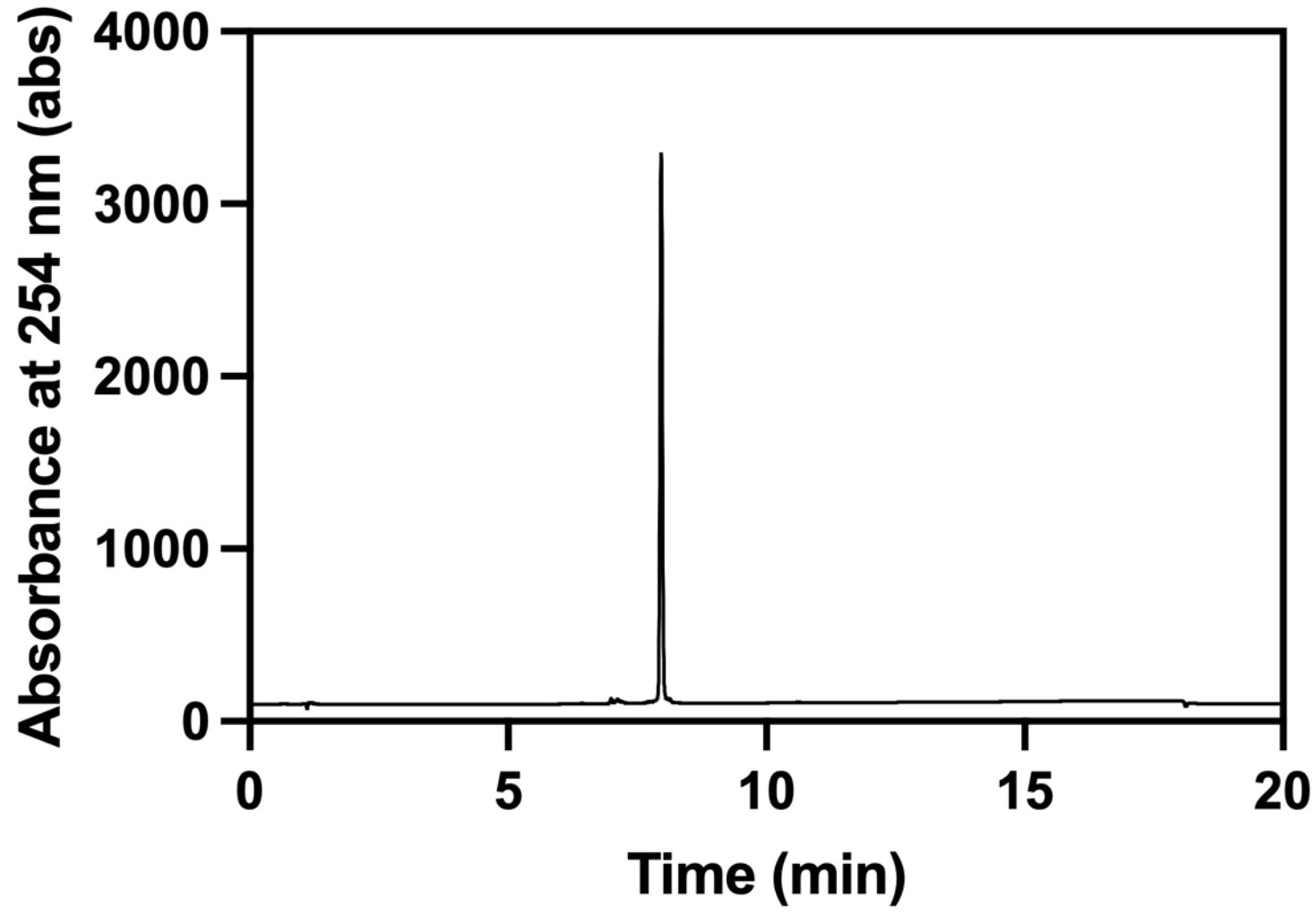

Figure S25. HPLC chromatogram of **KO-NH$_2$-N$_3$**. Retention time: 7.97 min. (Method A).

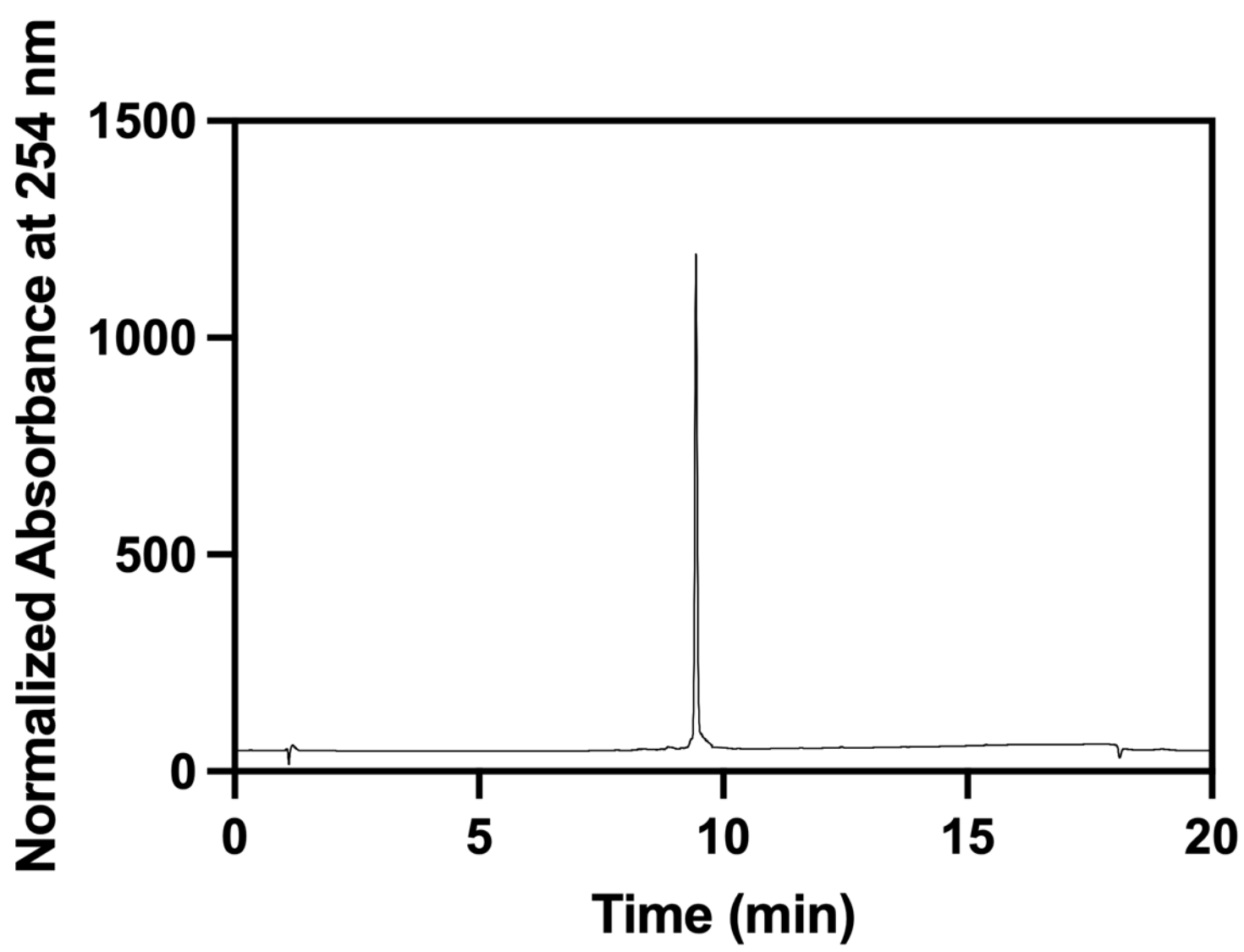

Figure S26. HPLC chromatogram of **KO-NH$_2$-Amp**. Retention time: 9.44 min. (Method A).

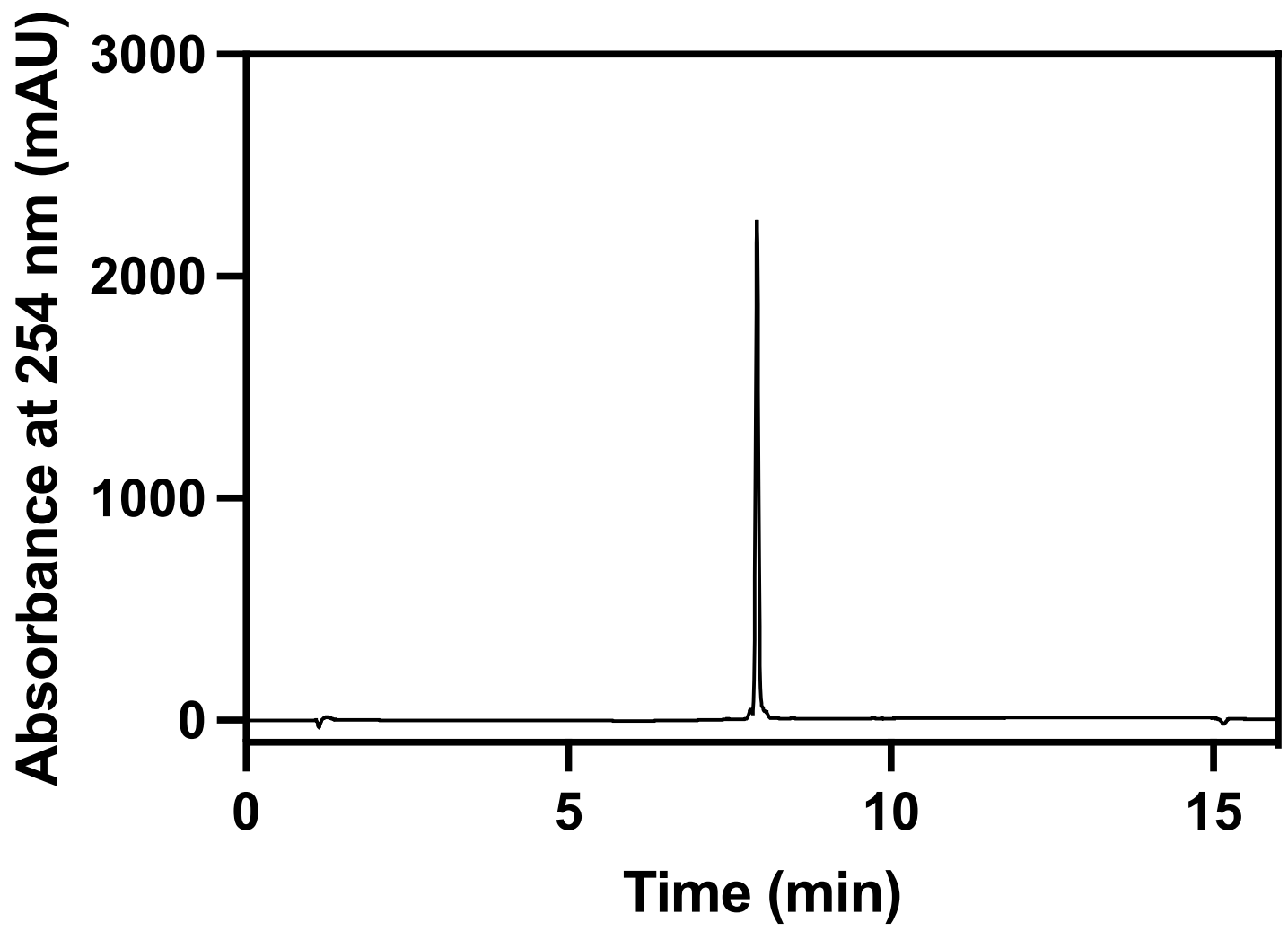

Figure S27. HPLC chromatogram of **KO-NH_2-azir-biotin**. Retention time: 7.92 min. (Method A).

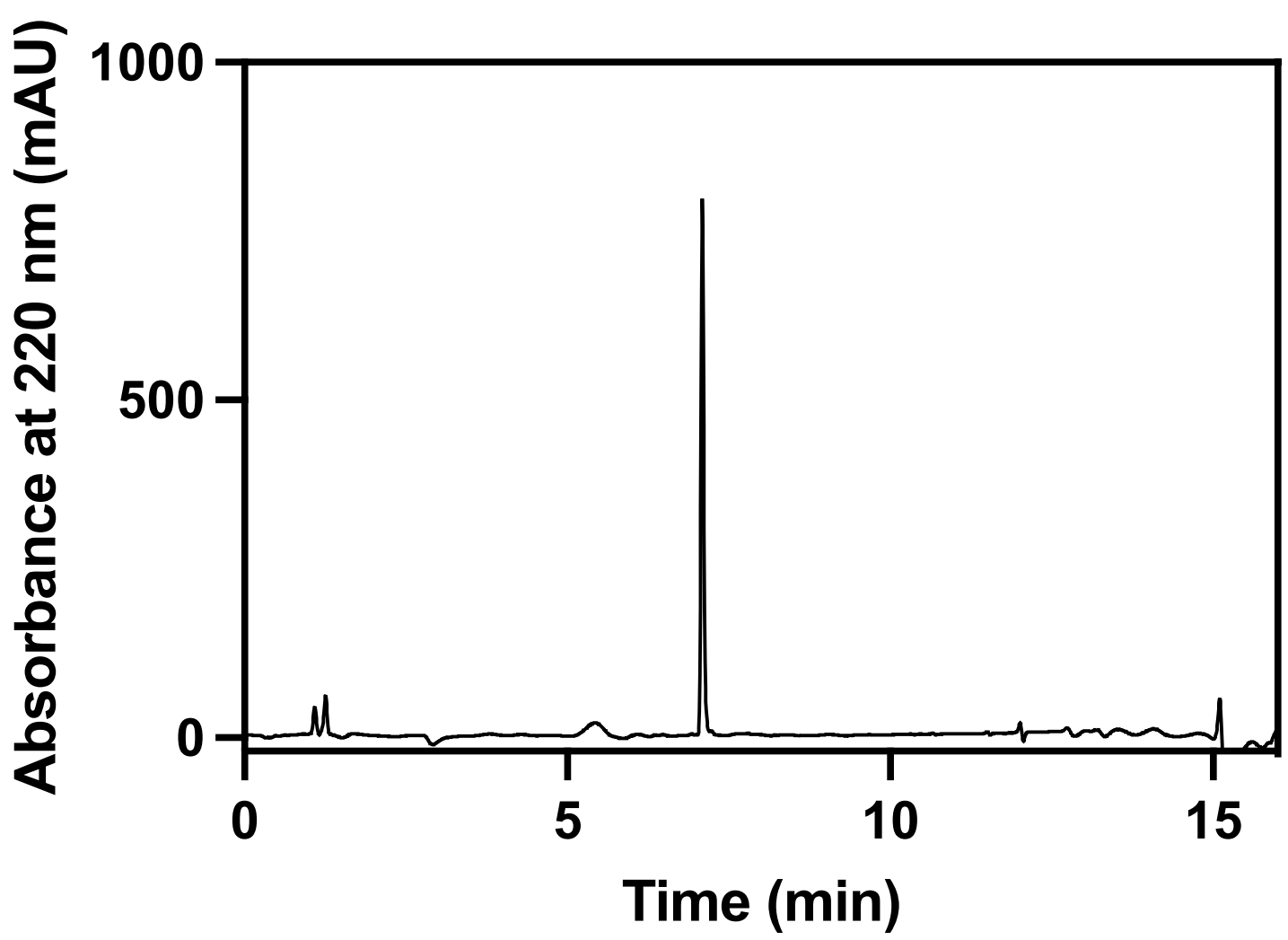

Figure S28. HPLC chromatogram of **alkyne-azir-biotin**. Retention time: 7.09 min. (Method A).

1.7.2 HRMS spectra

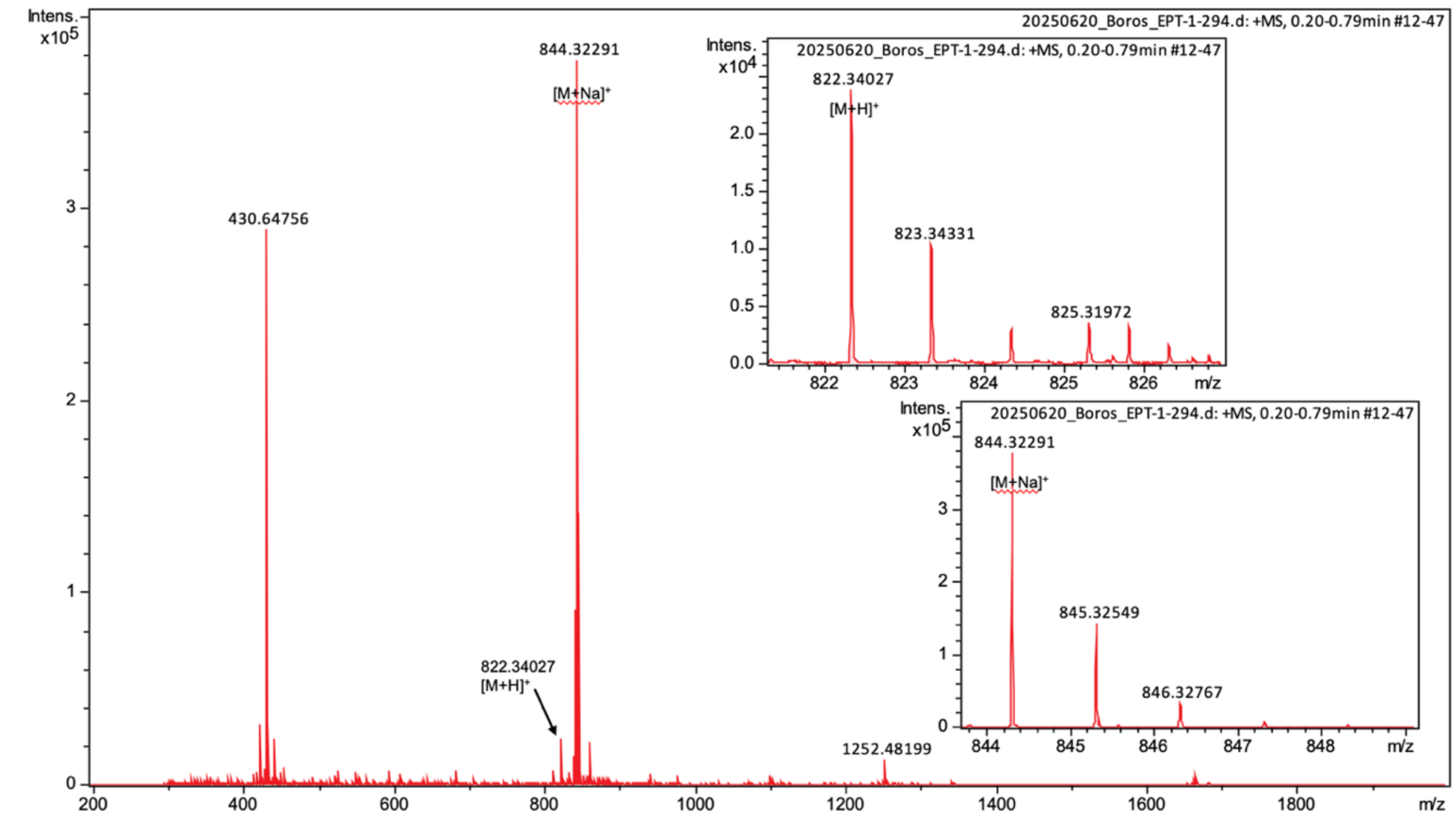

Figure S29. ESI (+) HR-MS spectrum of **KO-NH$_2$-N$_3$**. ESI-HR-MS calcd. for $C_{38}H_{47}N_9O_{12}$ $[M + H]^+$: *m/ z* 822.3416 and $[M + Na]^+$: *m/ z* 844.3236. Found $[M + H]^+$: 822.376 and $[M + Na]^+$: 844.3278.

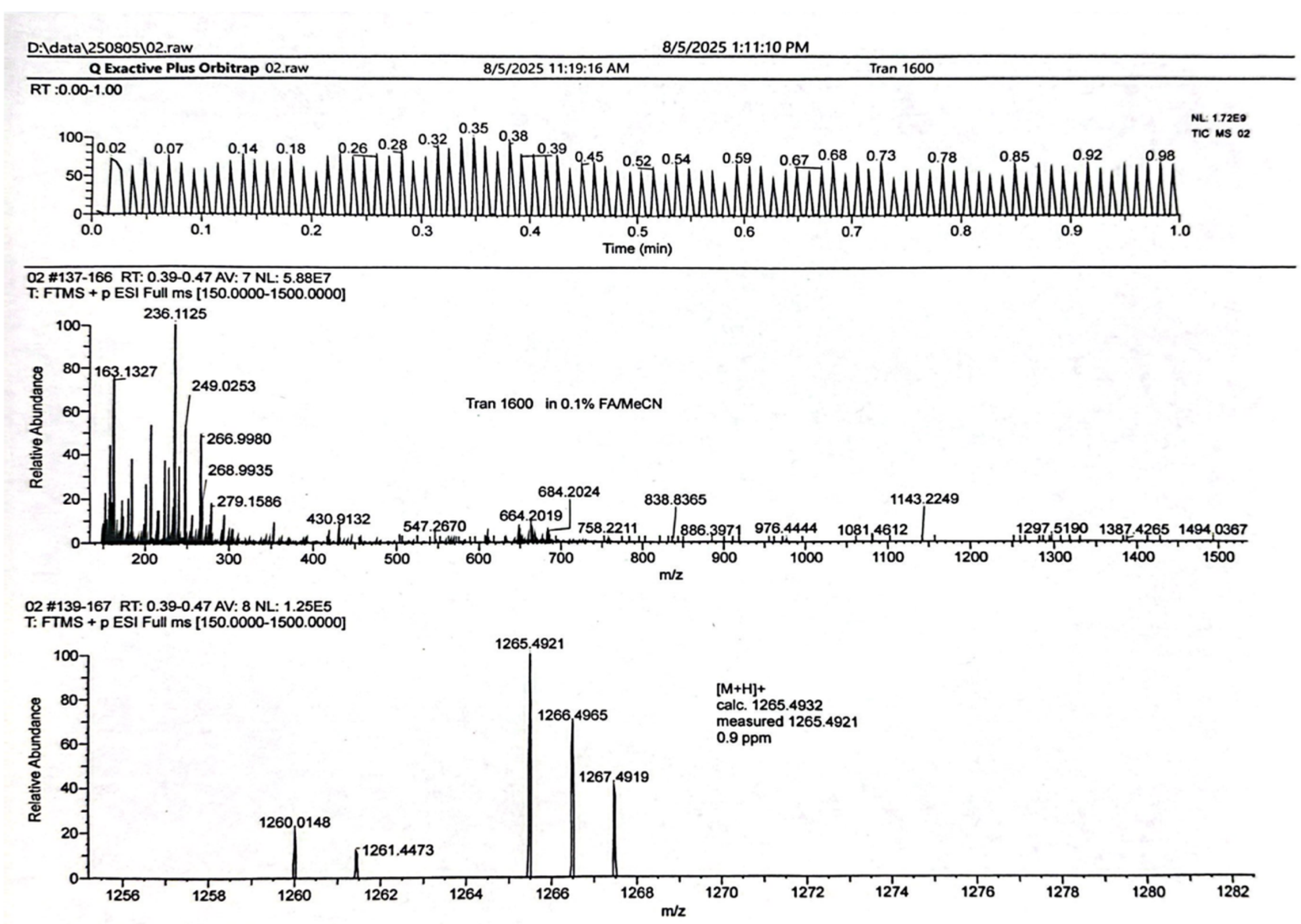

Figure S30. ESI (+) HR-MS spectrum of **KO-NH$_2$-Amp**. ESI-HR-MS calcd. for $C_{60}H_{72}N_{12}O_{17}S$ $[M + H]^+$: *m/ z* 1265.4932. Found $[M + H]^+$: 1265.4921.

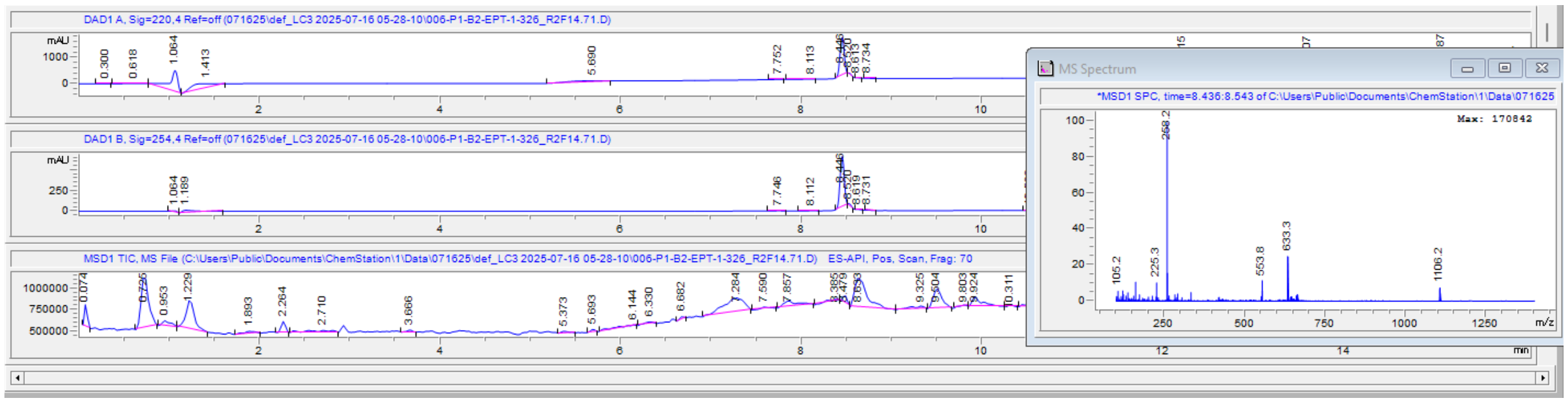

Figure S31. LR-MS spectrum of **KO-NH$_2$-Amp**. Calcd. for $C_{60}H_{72}N_{12}O_{17}S$ $[M + 2H]^{2+}$: *m/ z* 633.2502. Found $[M + 2H]^{2+}$: 633.3.

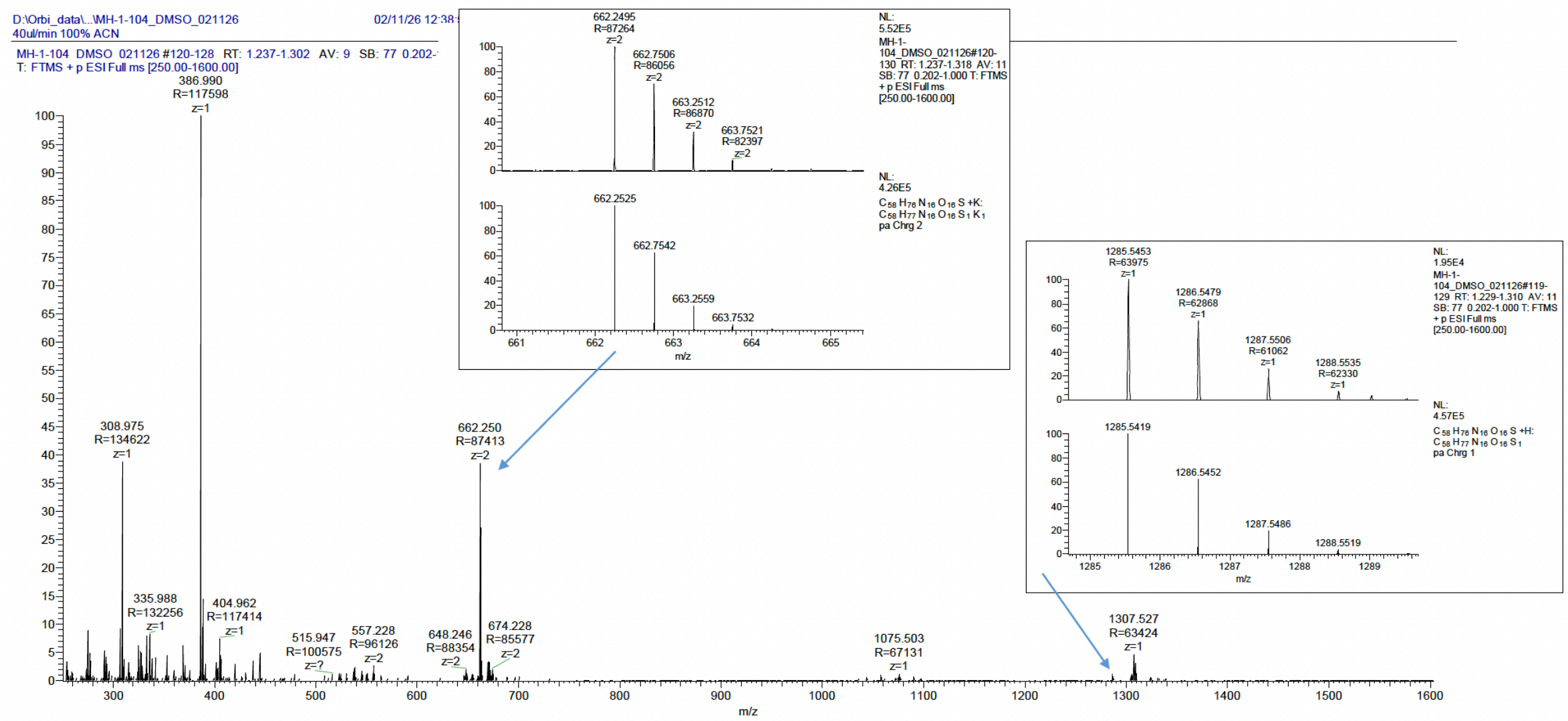

Figure S32. ESI (+) HR-MS spectrum of **KO-NH$_2$-azir-biotin**. ESI-HR-MS calcd. for $C_{58}H_{77}N_{16}O_{16}S$ $[M + H]^+$: *m/ z* 1285.5419, $[M+2H]^{2+}$: *m/z* 662.2525. Found $[M + H]^+$:1285.5453, $[M+2H]^{2+}$: 662.2495.

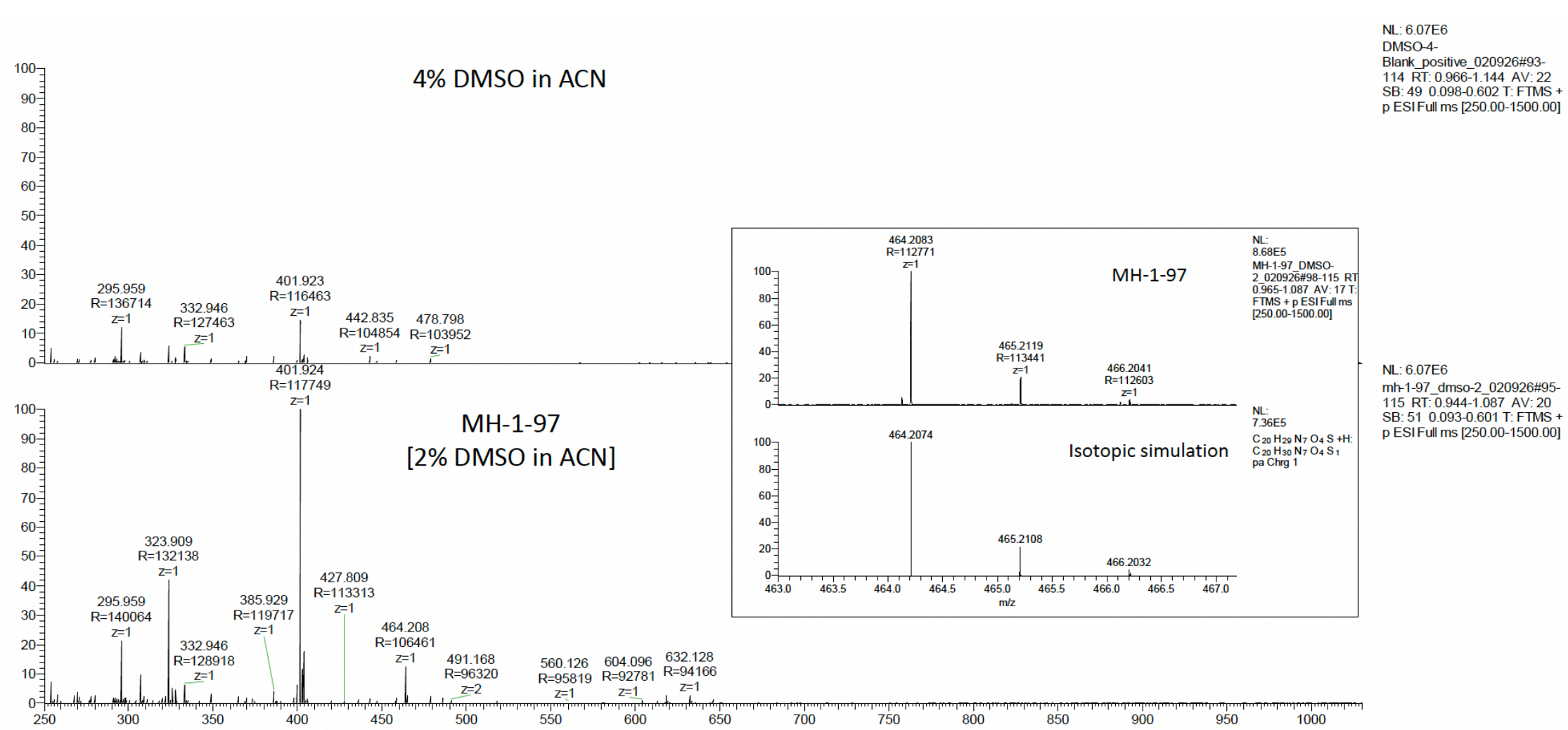

Figure S33. ESI (+) HR-MS spectrum of **alkyne-azir-biotin**. ESI-HR-MS calcd. for $C_{20}H_{29}N_7O_4S$ $[M + H]^+$: *m/ z* 464.2074. Found $[M + H]^+$:464.2083.

1.7.3 NMR spectra

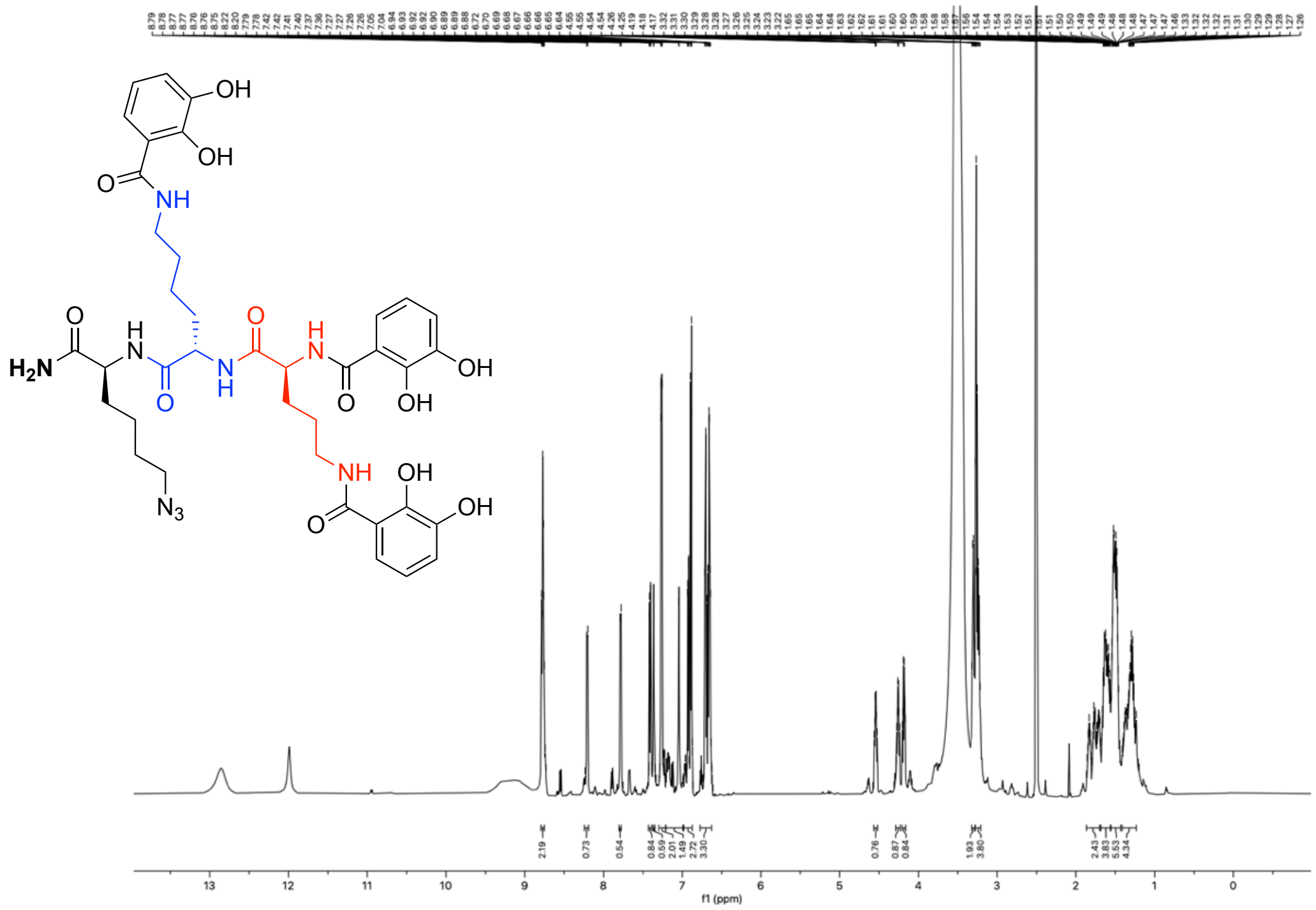

Figure S34. ^{1}H NMR (600 MHz, DMSO-d_6, 298 K) of **KO-NH$_2$-N$_3$**.

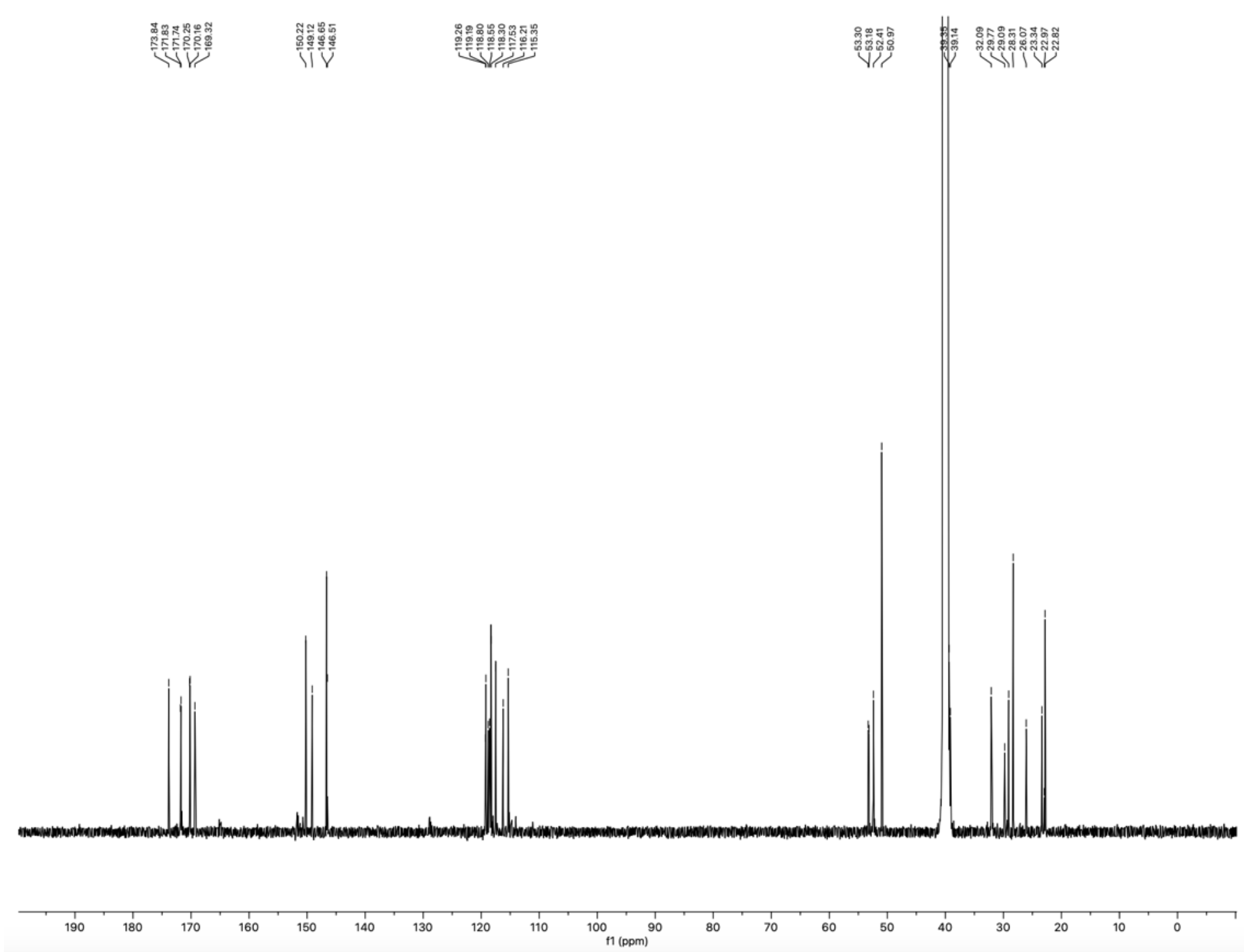

Figure S35. ^{13}C NMR (151 MHz, DMSO-d_6, 298 K) of **KO-NH$_2$-N$_3$**.

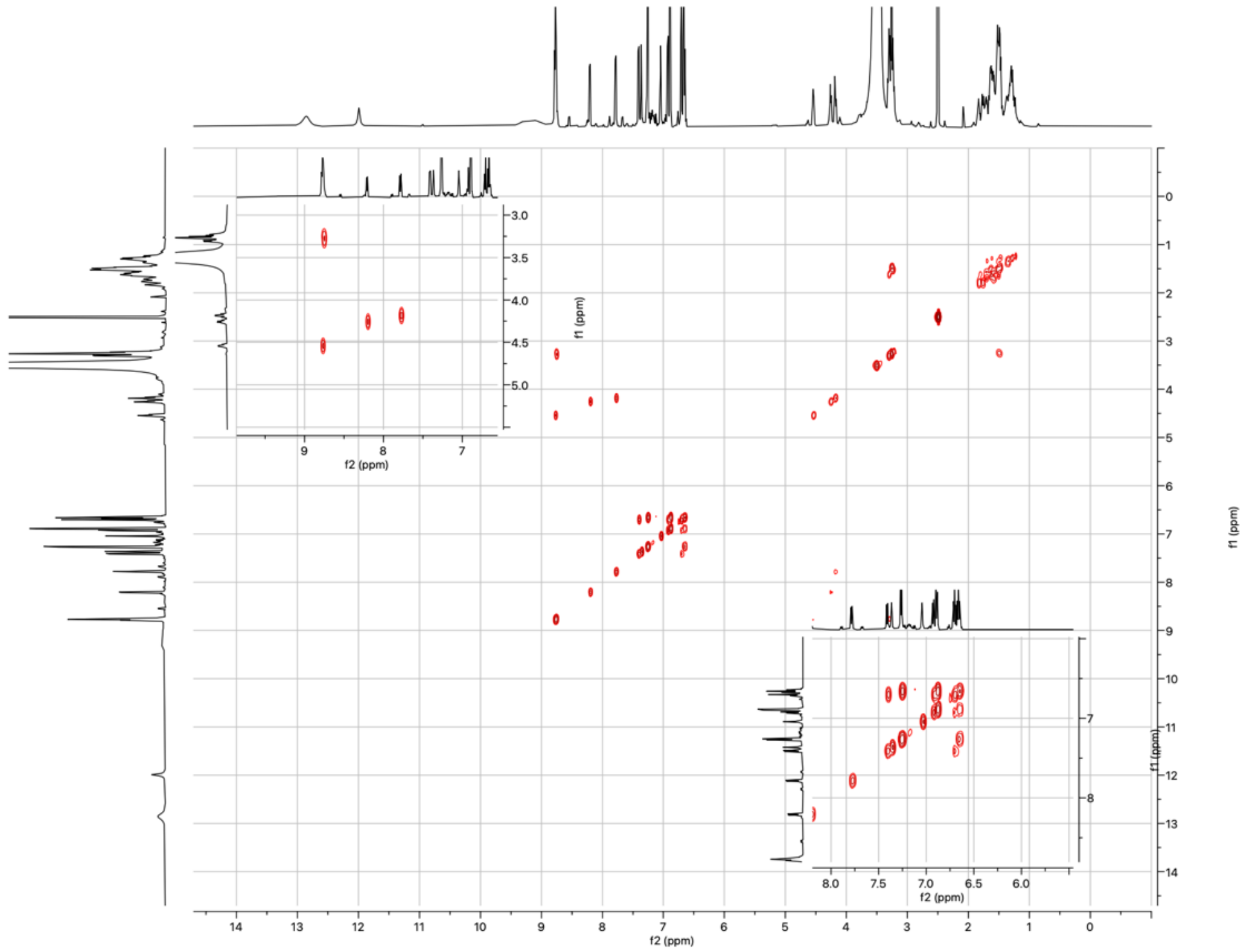

Figure S36. ^{1}H-^{1}H COSY NMR (600 MHz, DMSO-*d6*, 298 K) of **KO-NH$_2$-N$_3$**.

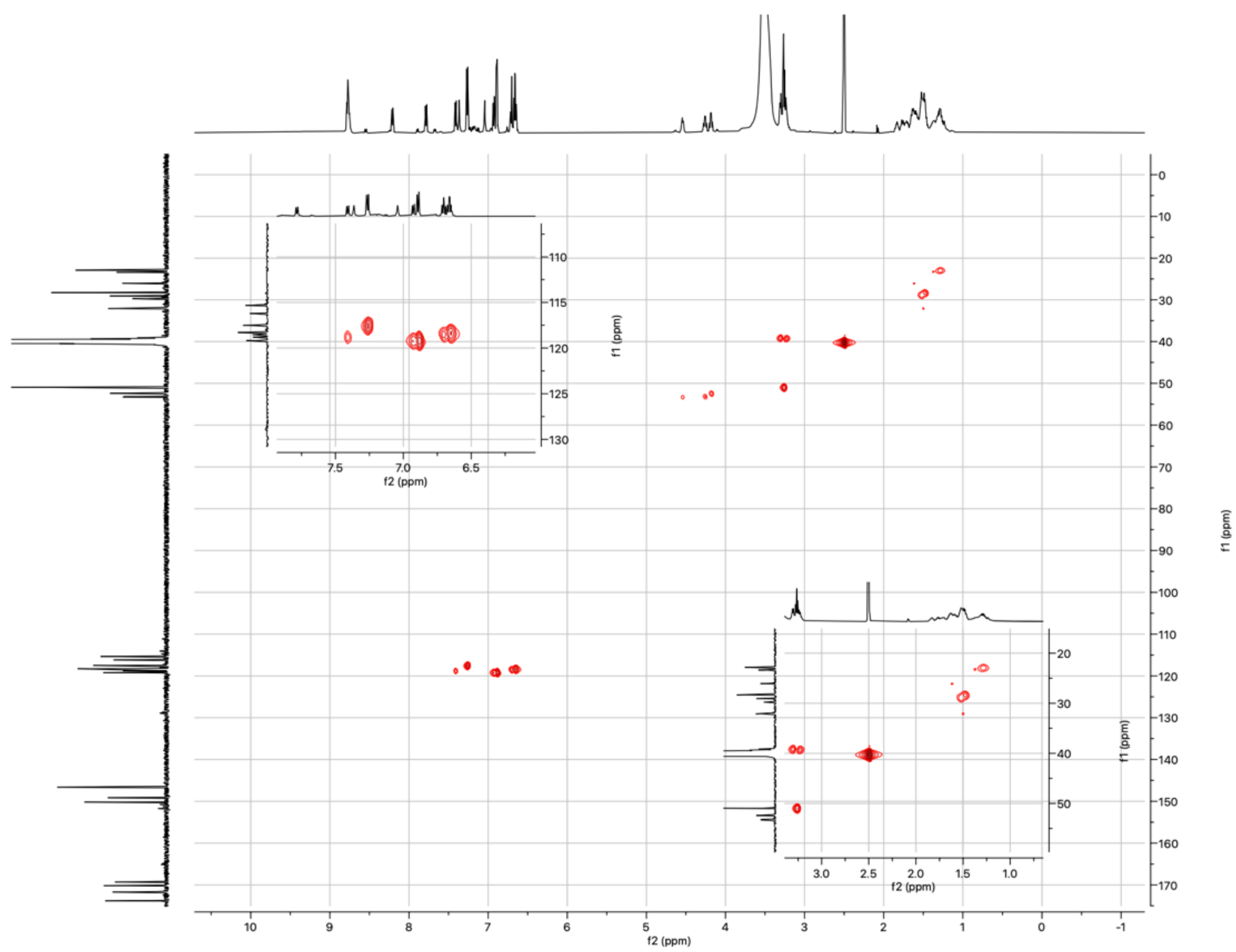

Figure S37. ^{1}H-^{13}C HSQC NMR (600 MHz, DMSO-*d6*, 298 K) **KO-NH$_2$-N$_3$**.

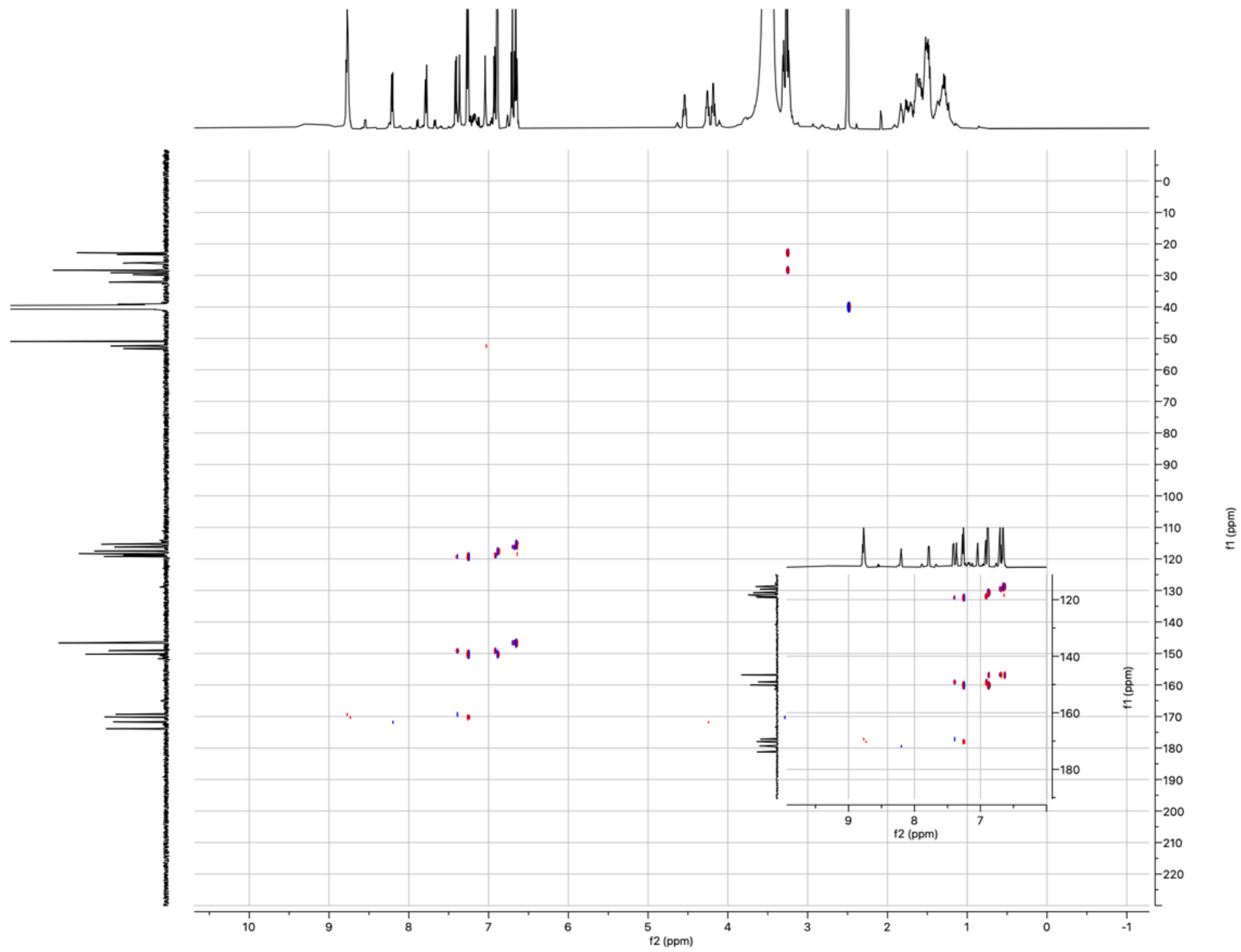

Figure S38. ^{1}H-^{13}C HMBC NMR (600 MHz, DMSO-*d6*, 298 K) **KO-NH$_2$-N$_3$**.

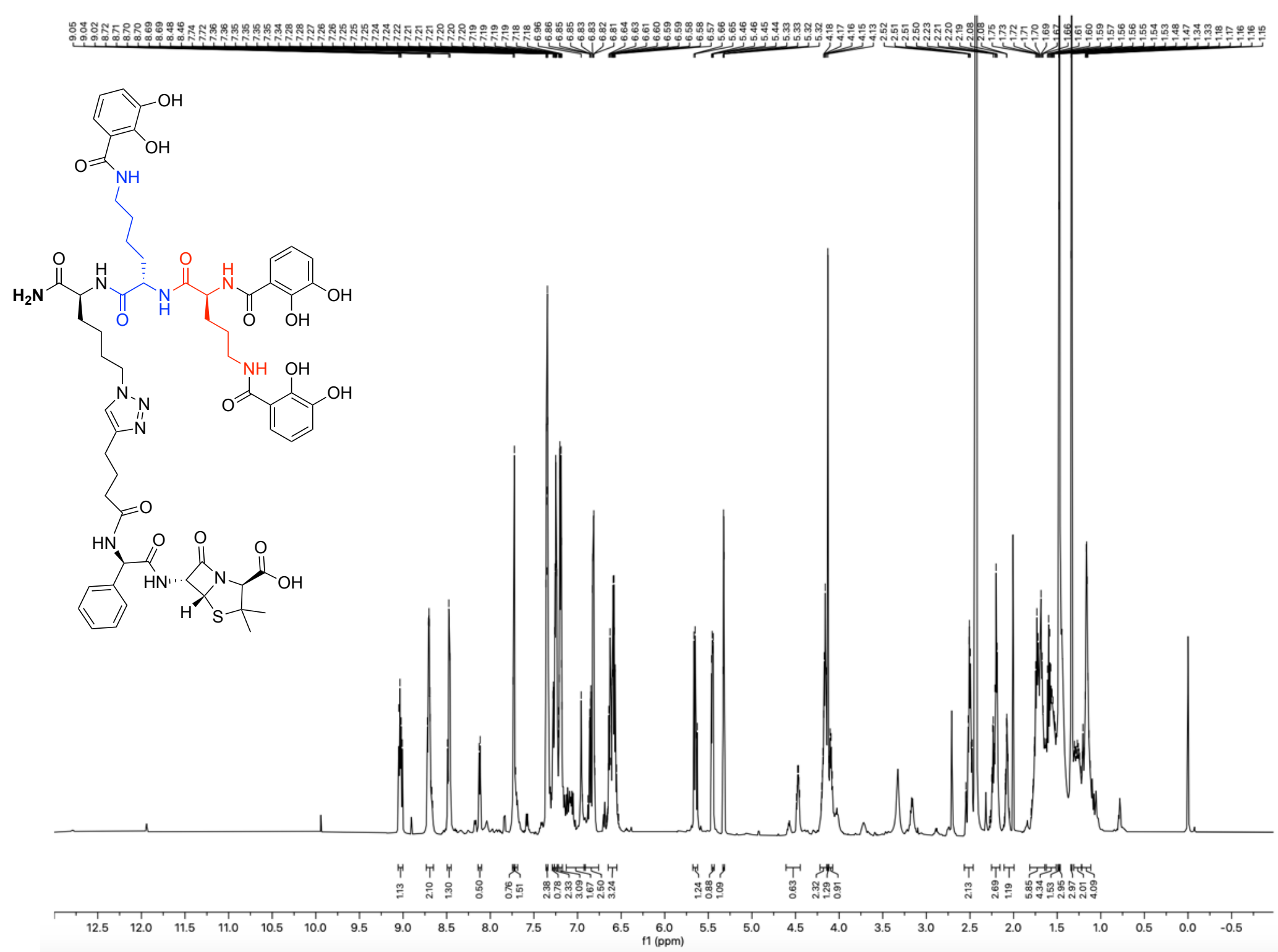

Figure S39. ^{1}H NMR (600 MHz, DMSO-*d6*, 298 K) of **KO-NH$_2$-Amp**.

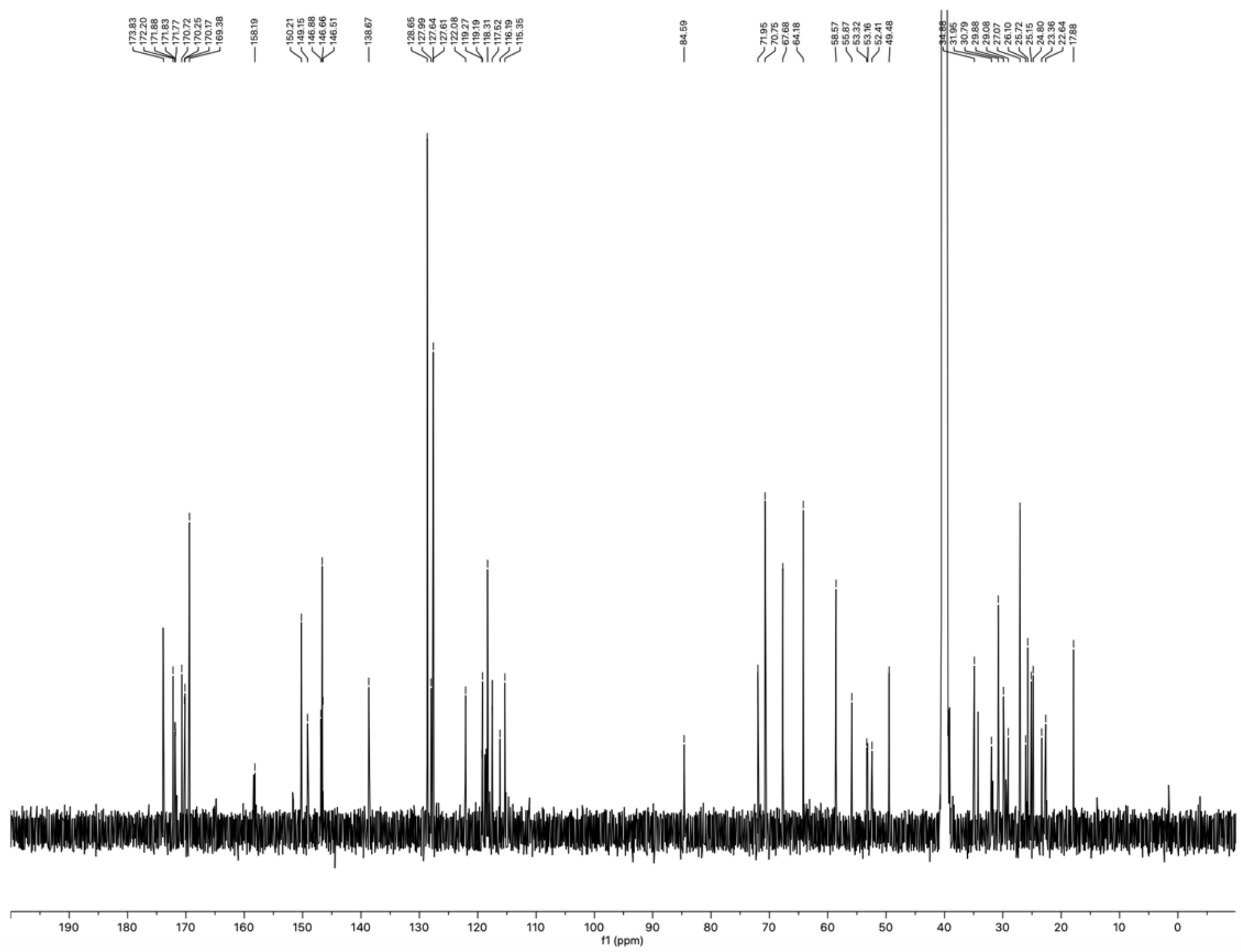

Figure S40. ^{13}C NMR (151 MHz, DMSO-*d6*, 298 K) of **KO-NH$_2$-Amp**.

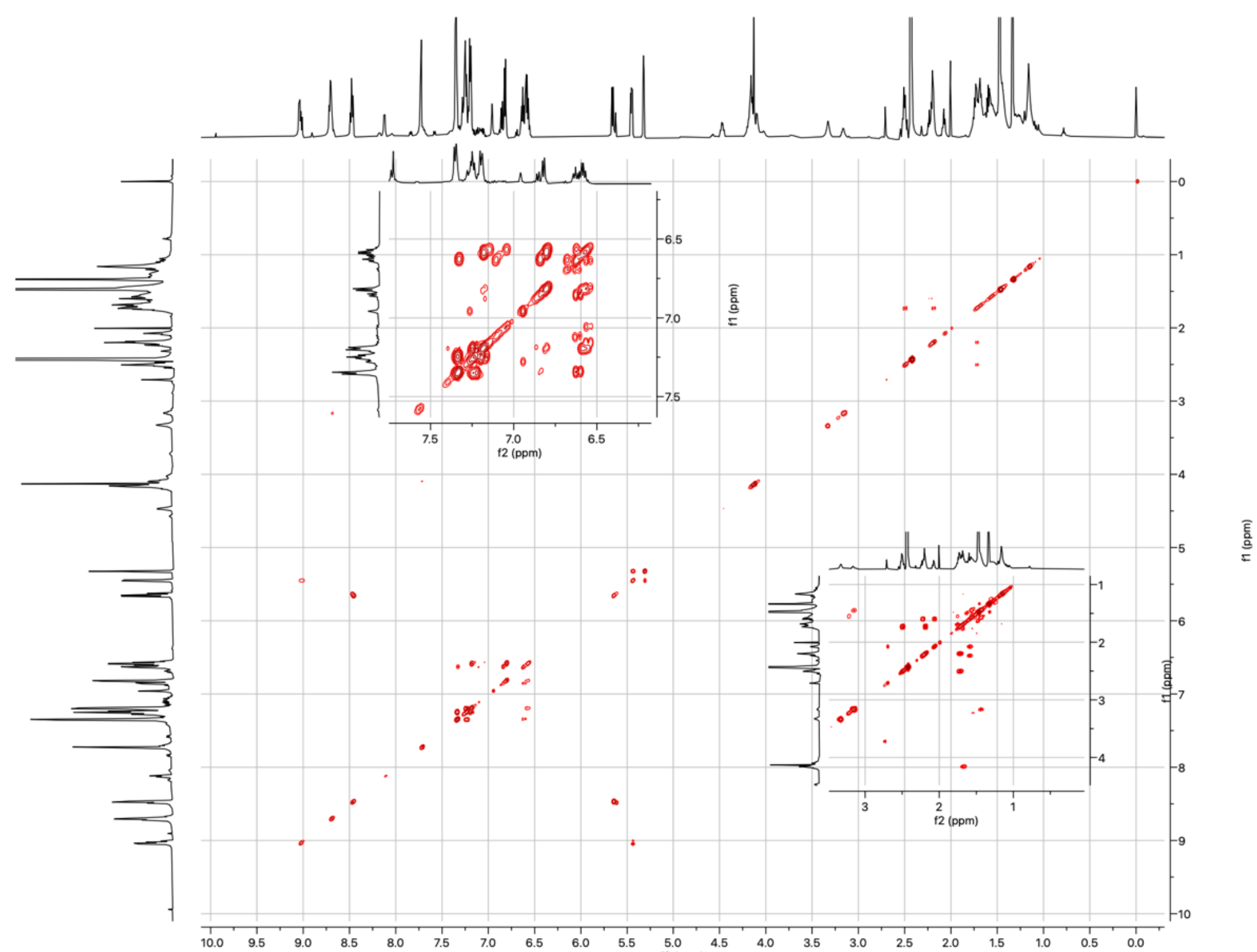

Figure S41. ^{1}H-^{1}H COSY NMR (600 MHz, DMSO-*d6*, 298 K) of **KO-NH$_2$-Amp**.

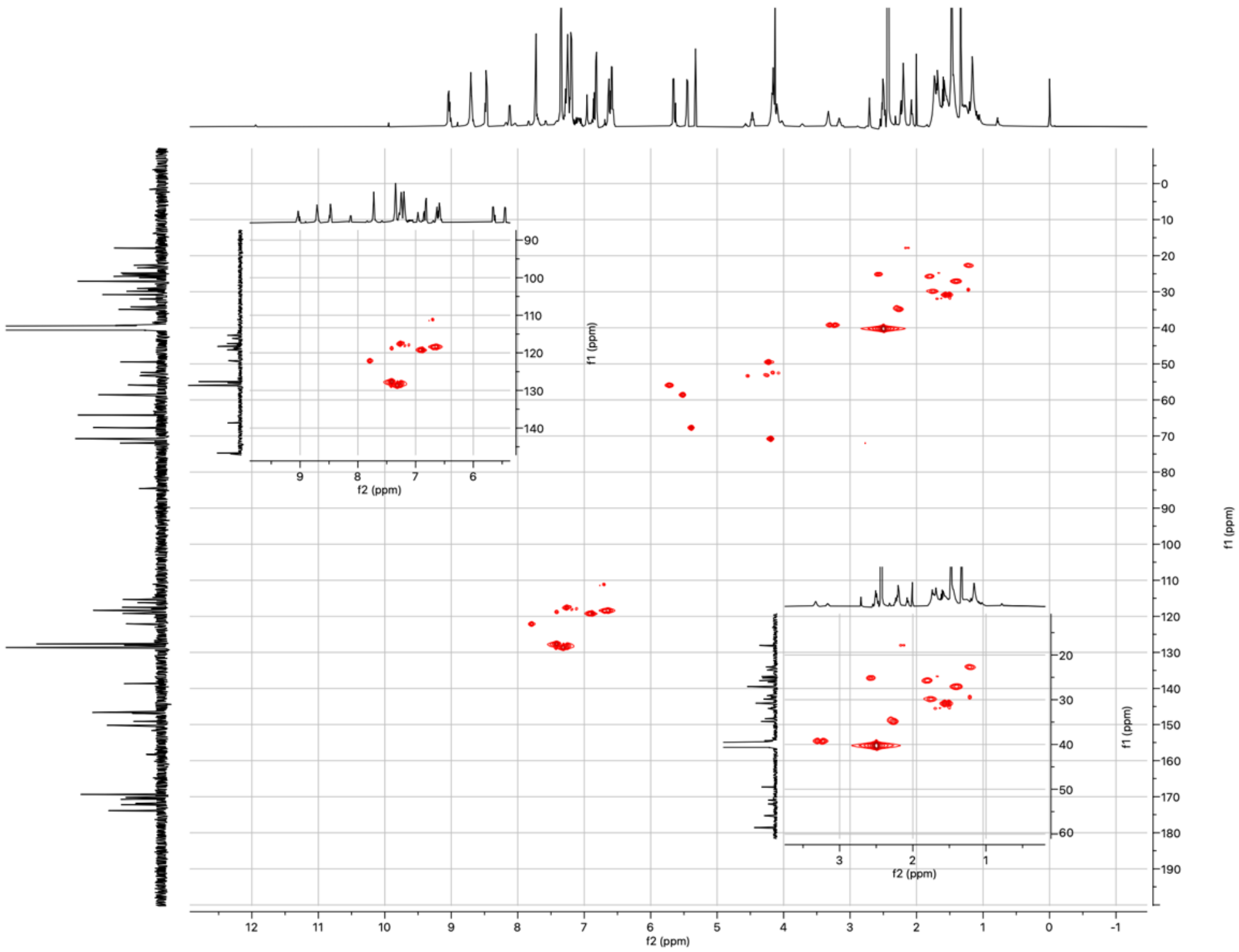

Figure S42. ^{1}H-^{13}C HSQC NMR (600 MHz, DMSO-*d6*, 298 K) of **KO-NH$_2$-Amp**.

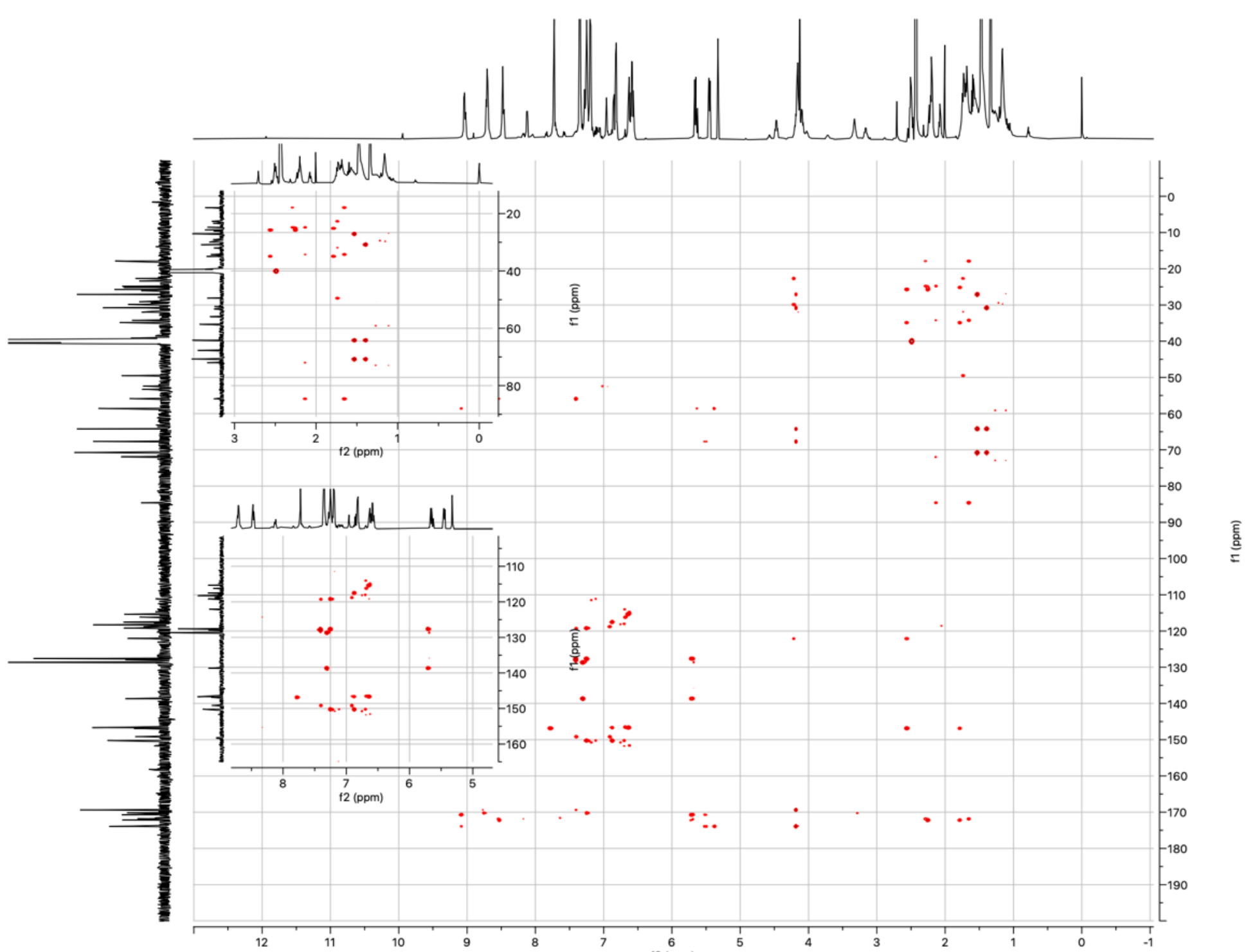

Figure S43. ^{1}H-^{13}C HMBC NMR (600 MHz, DMSO-*d6*, 298 K) of **KO-NH$_2$-Amp**.

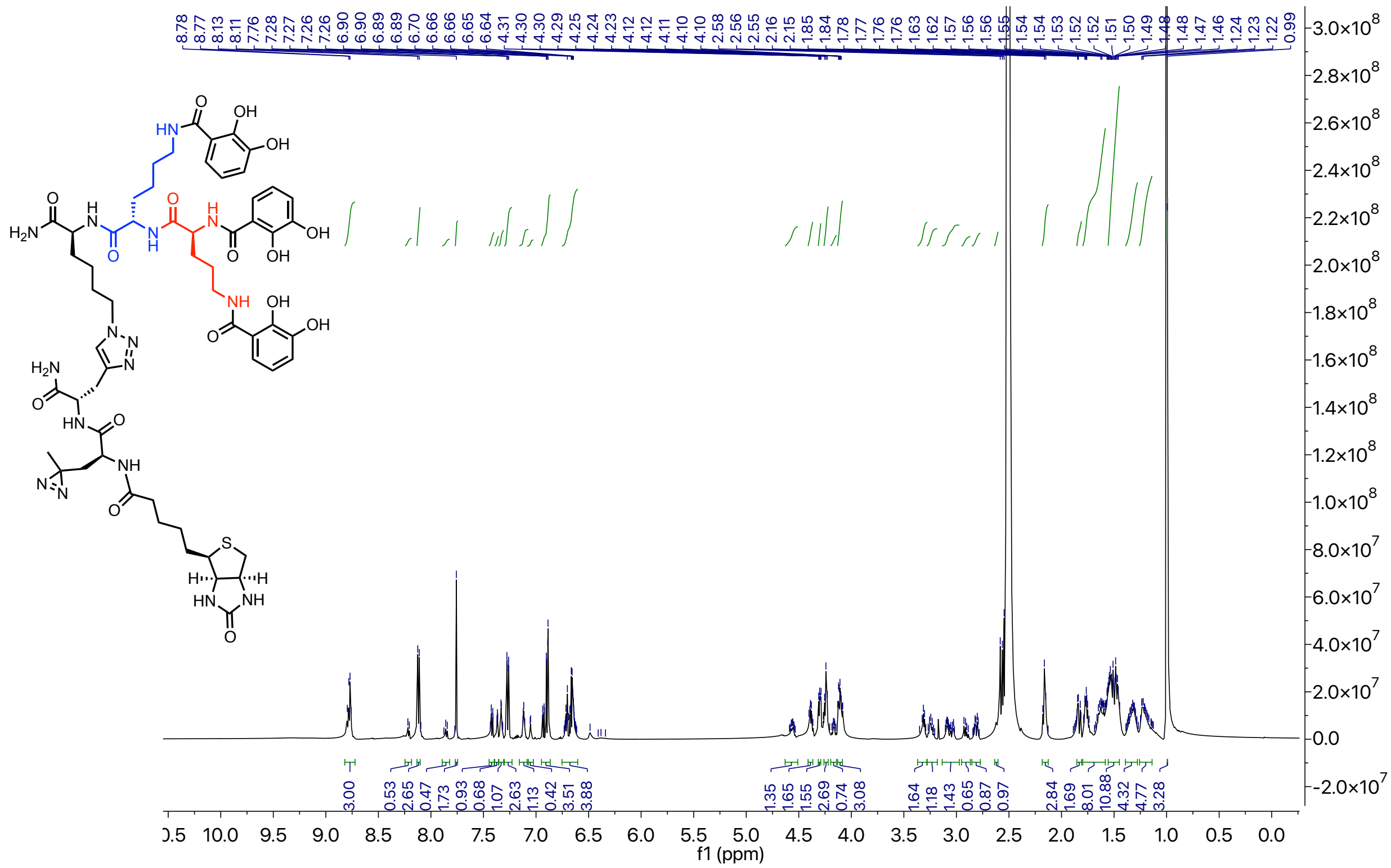

Figure S44. ^{1}H NMR (600 MHz, DMSO-*d6*, 298 K) of **KO-NH$_2$-azir-biotin**.

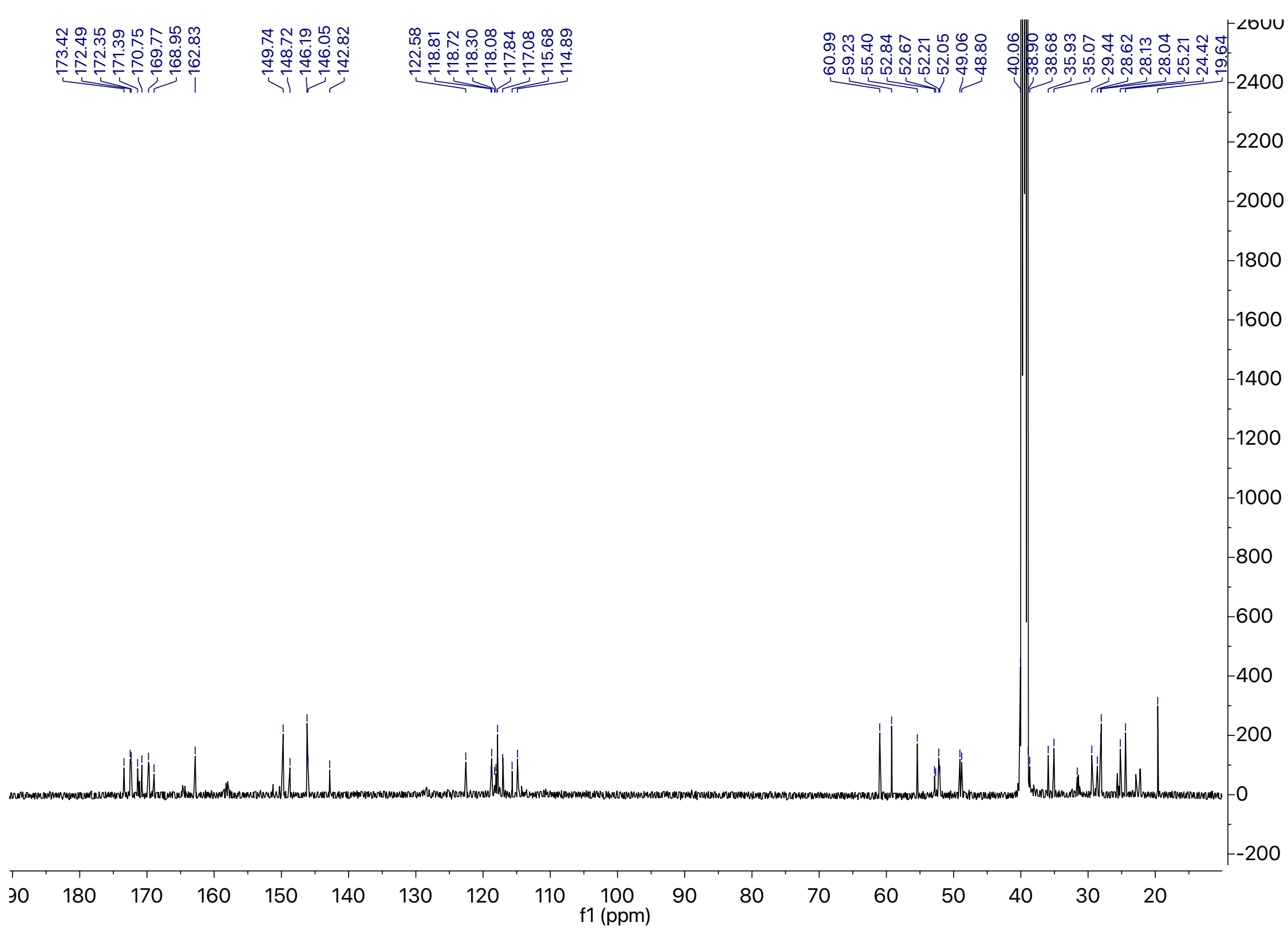

Figure S45. ^{13}C NMR (151 MHz, DMSO-*d6*, 298 K) of **KO-NH$_2$-azir-biotin**.

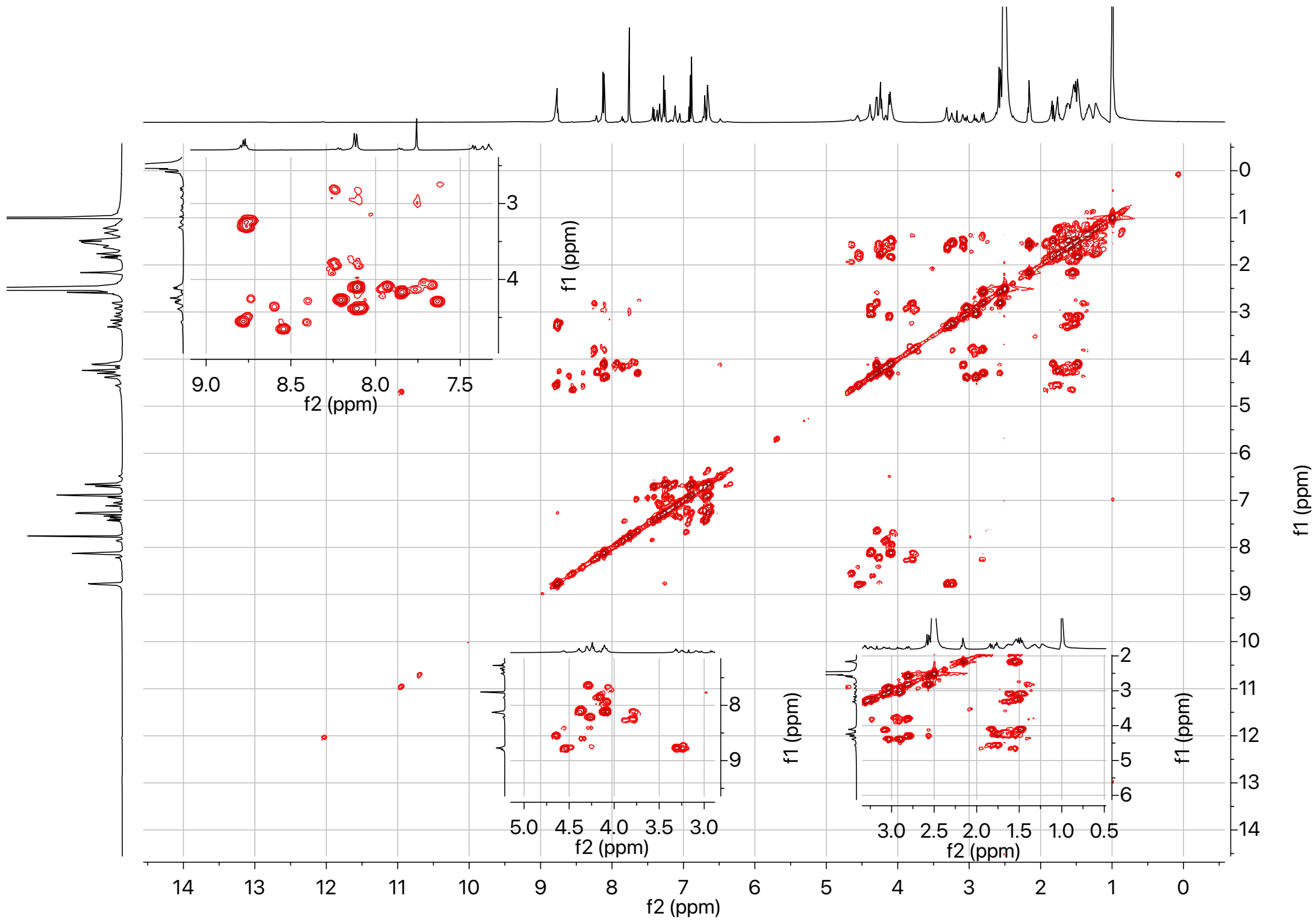

Figure S46. ^{1}H-^{1}H COSY NMR (600 MHz, DMSO-*d6*, 298 K) of **KO-NH$_2$-azir-biotin**.

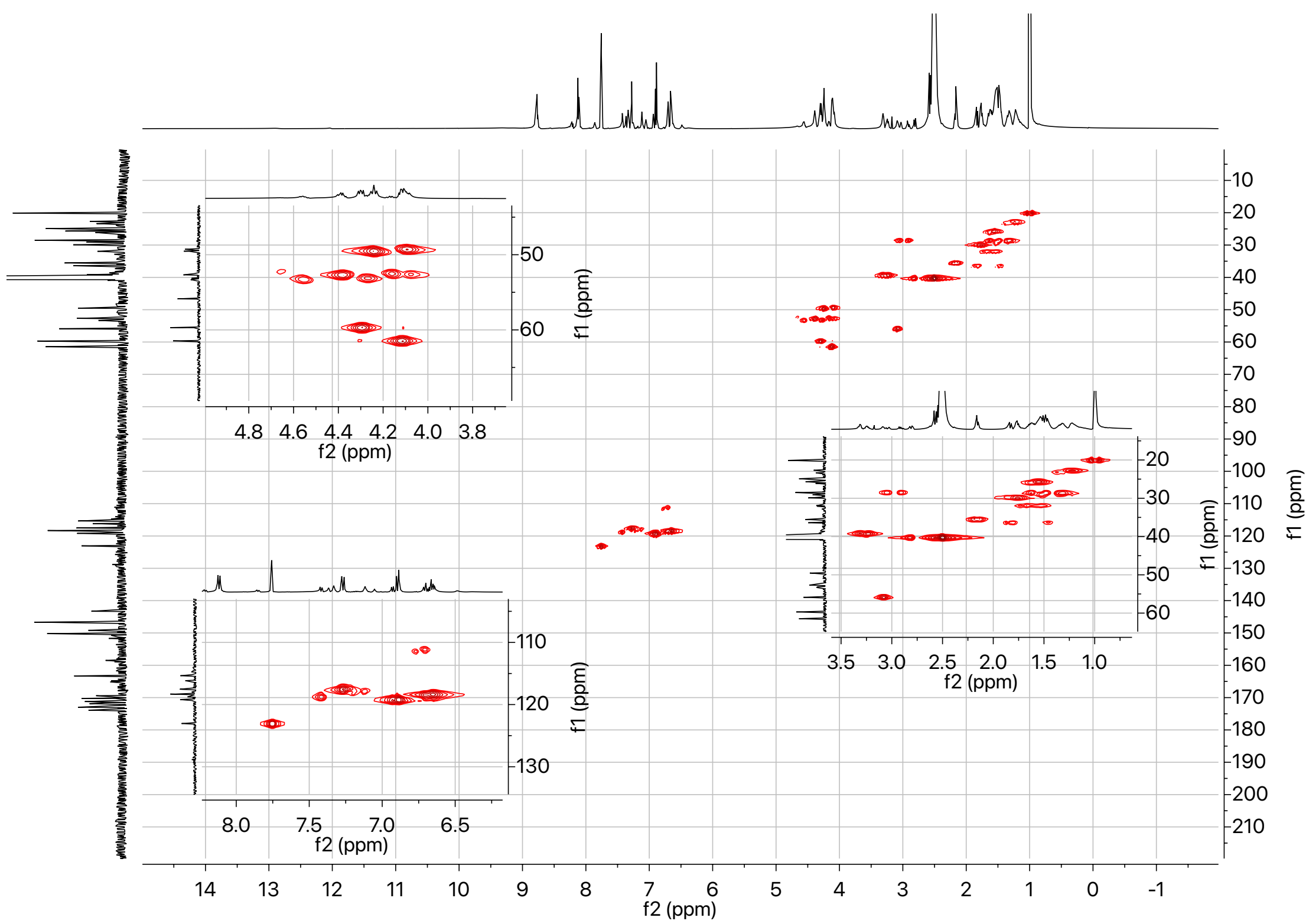

Figure S47. ^{1}H-^{13}C HSQC NMR (600 MHz, DMSO-*d6*, 298 K) of **KO-NH$_2$-azir-biotin**.

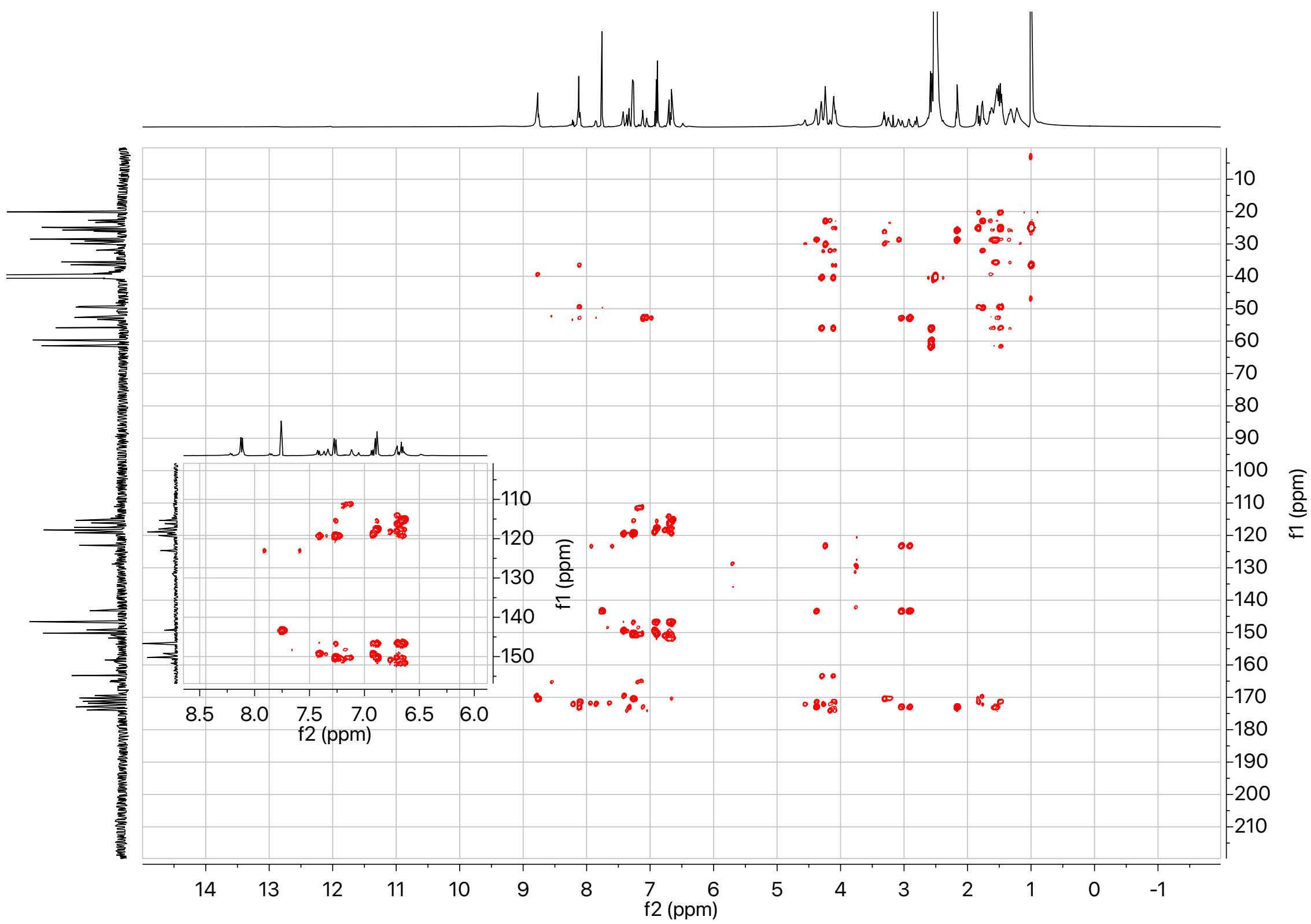

Figure S48. ^{1}H-^{13}C HMBC NMR (600 MHz, DMSO-*d6*, 298 K) of **KO-NH₂-azir-biotin**.

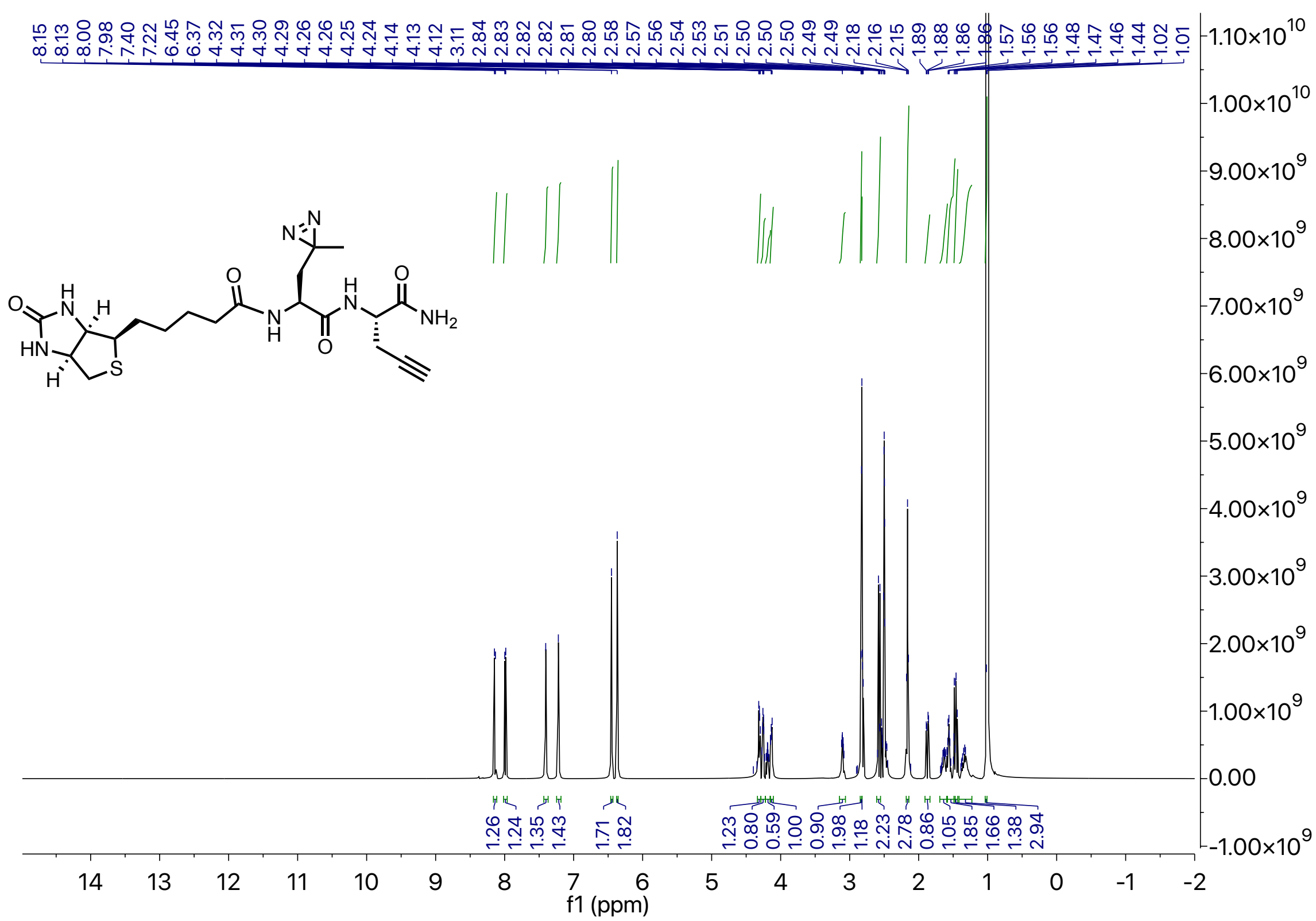

Figure S49. ^{1}H NMR (600 MHz, DMSO-*d6*, 298 K) of **alkyne-azir-biotin**.

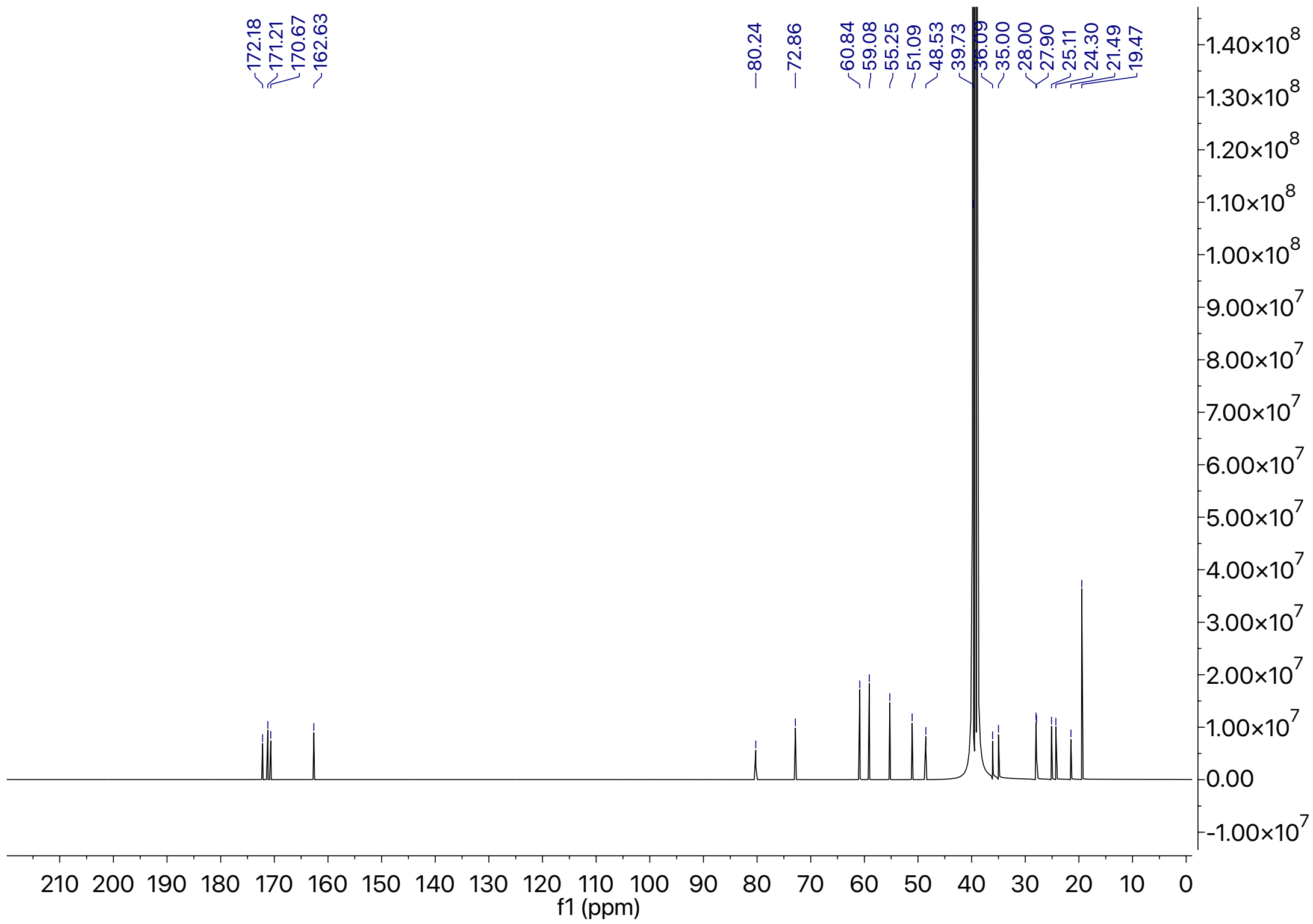

Figure S50. ^{13}C NMR (151 MHz, DMSO-*d6*, 298 K) of **alkyne-azir-biotin**.

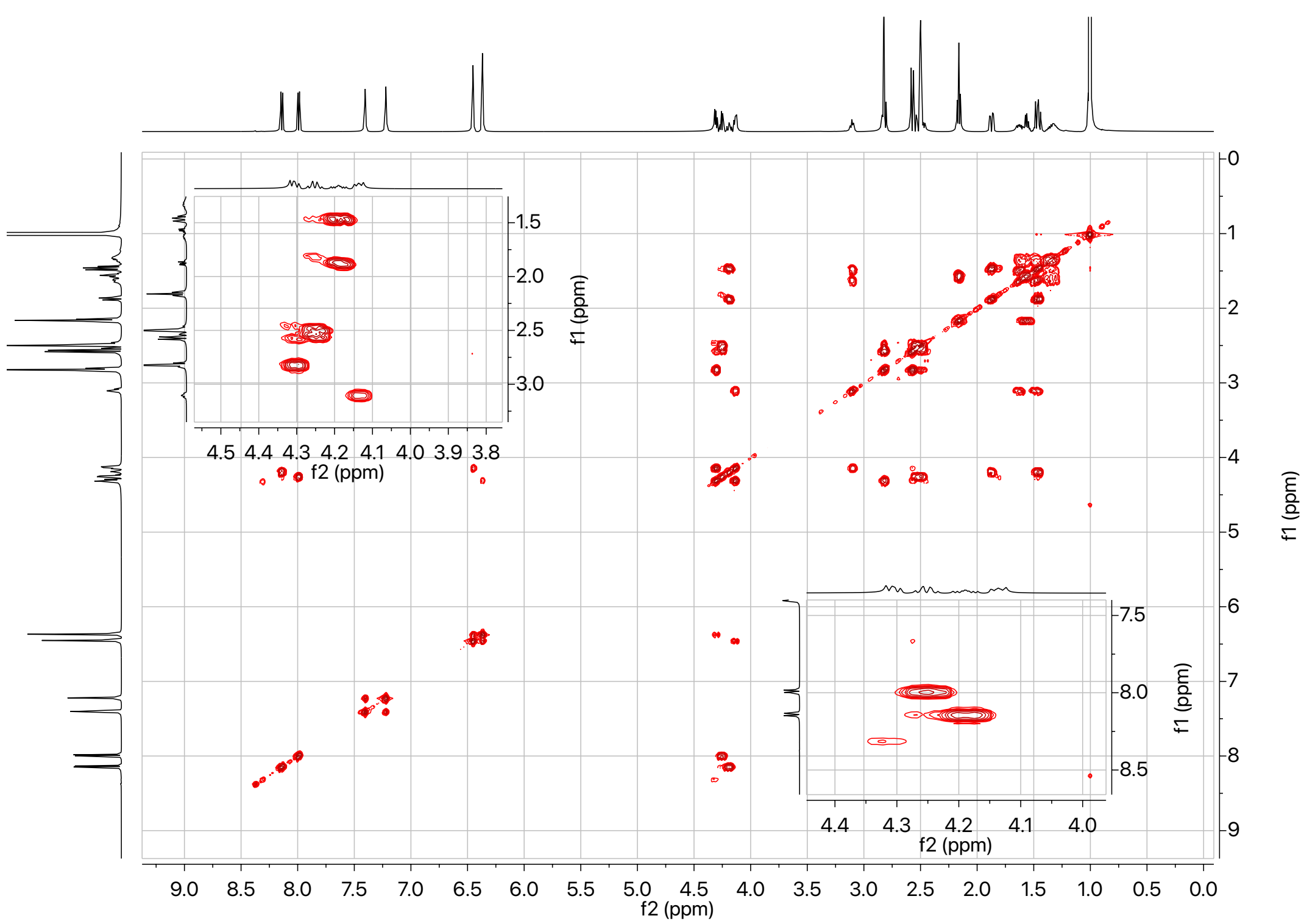

Figure S51. ^{1}H-^{1}H COSY NMR (600 MHz, DMSO-*d6*, 298 K) of **alkyne-azir-biotin**.

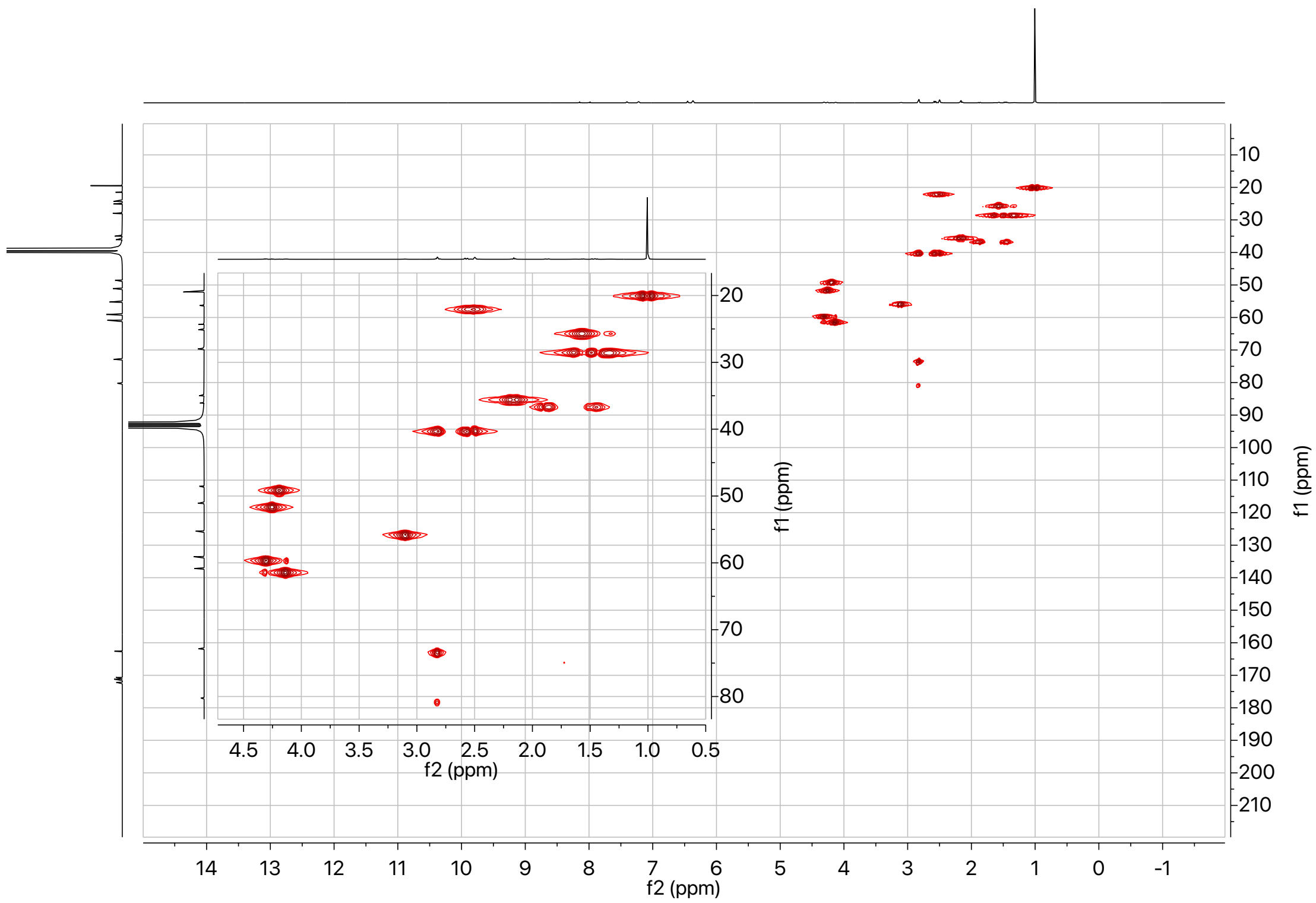

Figure S52. ^{1}H-^{13}C HSQC NMR (600 MHz, DMSO-*d6*, 298 K) of **alkyne-azir-biotin**.

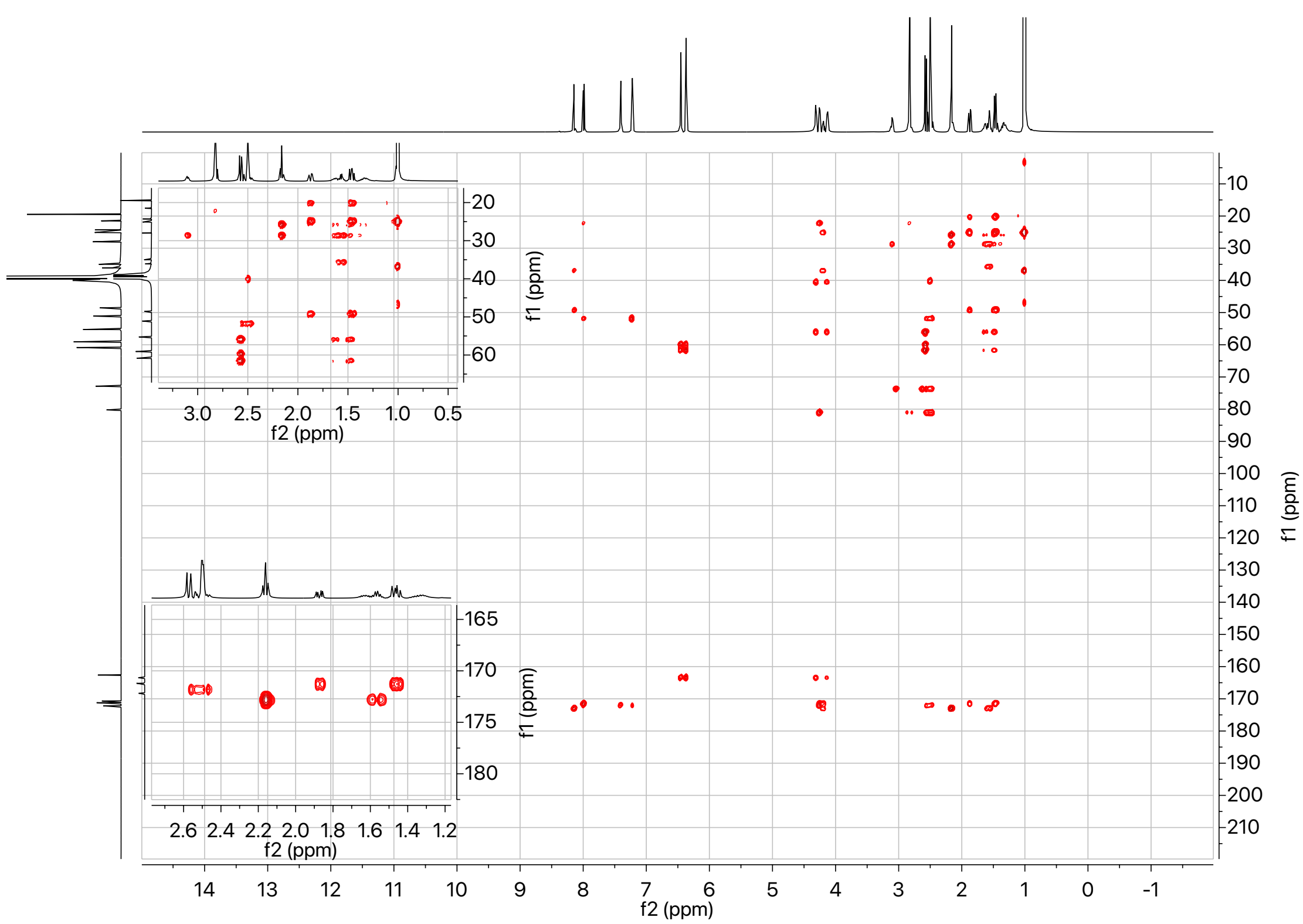

Figure S53. ^{1}H-^{13}C HMBC NMR (600 MHz, DMSO-*d6*, 298 K) of **alkyne-azir-biotin**.

1.8 Complex synthesis

1.8.1 Iron complexation

All iron complexes were formed overnight at room temperature in 30% DMSO in H_2O using a 1:1 ratio ligand:$FeCl_3$ (concentration determined by ICP-OES). After 16 h at room temperature, the solvent was removed under reduced pressure. The residue was redissolved in MeOH and purified by analytical reverse phase HPLC (Method D) to yield the pure complex. Pure complexes were then characterized by analytical HPLC (Method D/H), HRMS and UV-Vis.

1.8.2 Gallium complexation

All gallium complexes were formed overnight at room temperature in anhydrous MeOH using a 1:1 ratio ligand:$Ga(acac)_3$ (concentration determined by ICP-OES). After 16 h at room temperature, the solvent was removed under reduced pressure. The residue was redissolved in MeOH and purified by analytical reverse phase HPLC (Method D/H) to yield the pure complex. Pure complexes were then characterized by ^1H NMR, analytical HPLC (method D), HRMS and UV-Vis.

1.9 Analytical and spectroscopic characterization of the complex

1.9.1 KK-COOH

$[Fe($KK$-COOH)]^{3-}$, was synthesized by the procedure outlined in section **1.8.1**. Retention time = 6.51 min (Method H). ESI-HR-MS: calcd. for $C_{33}H_{32}FeN_4O_{12}$ $[M+2H]^-$: m/z 734.1517 and $[M + H]^{2-}$: *m/z* 366.5717. Found 734.1512 and 366.5729, respectively. d-d transition: λ = 550 nm.

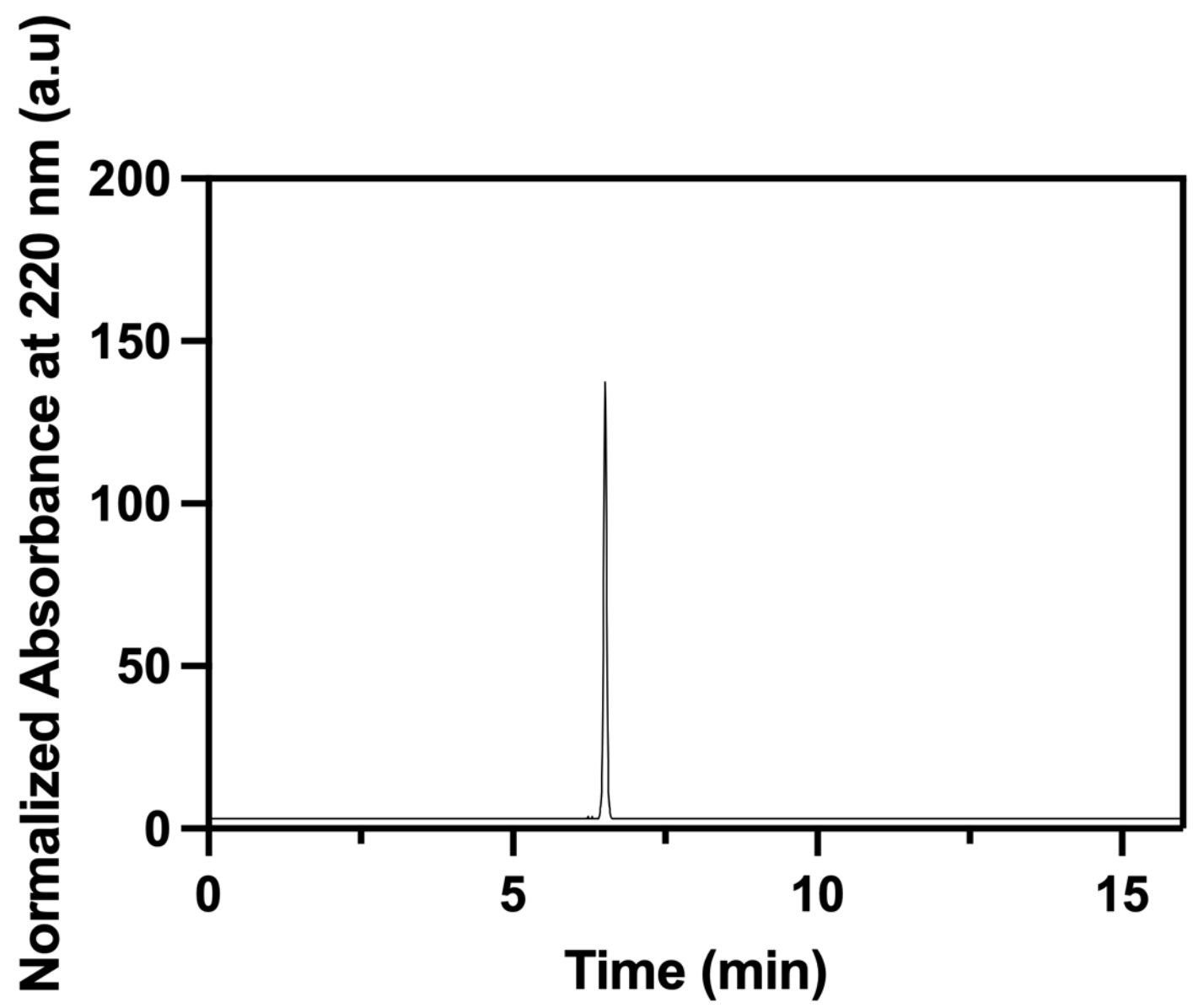

Figure S54. Normalized HPLC chromatogram of **$[Fe($KK$-COOH)]^{3-}$**. Retention time: 6.51 min (Method H).

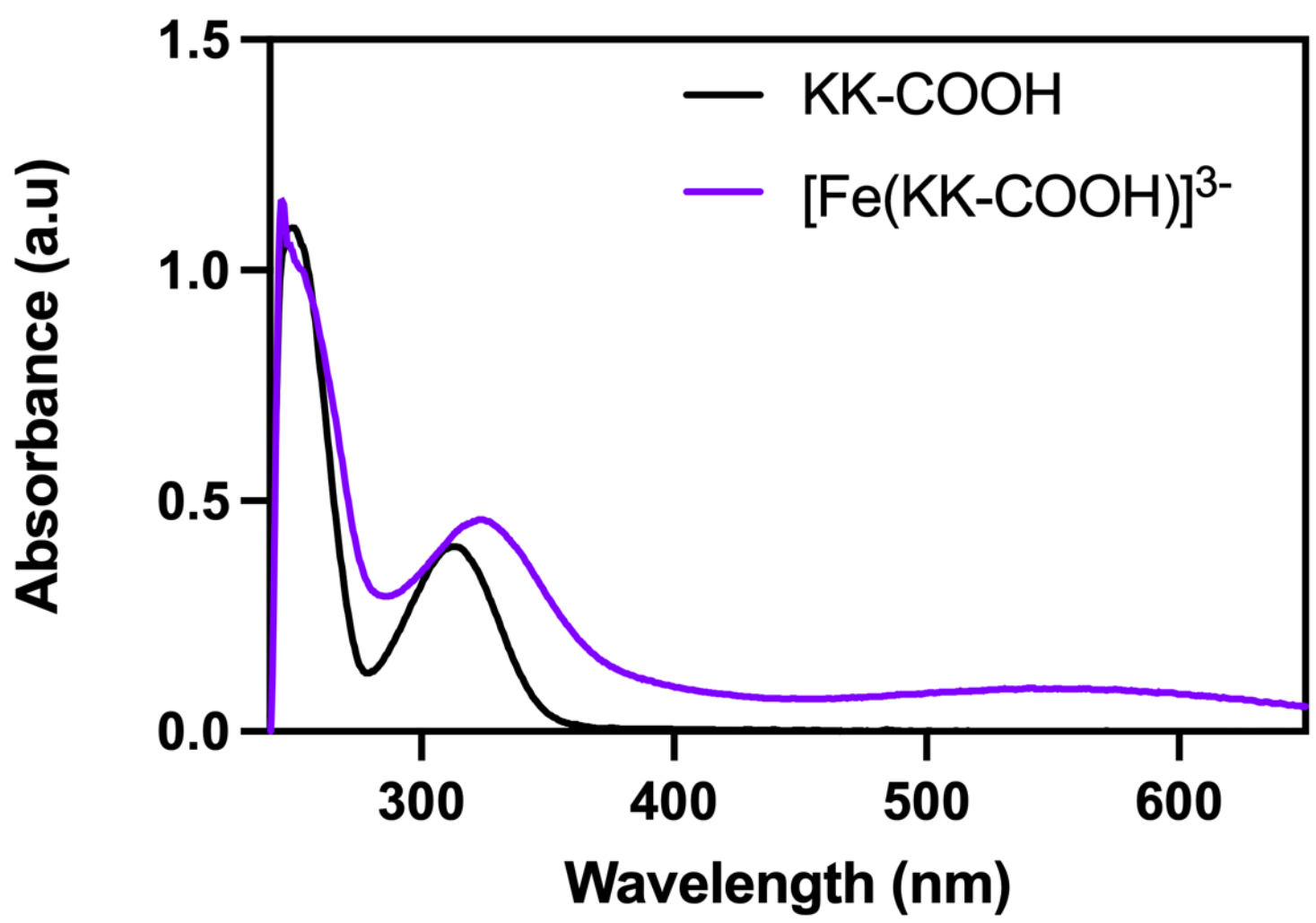

Figure S55. UV-vis spectrum of **$[Fe($KK$-COOH)]^{3-}$**. Observed LMTC, d-d transition λ = 550 nm.

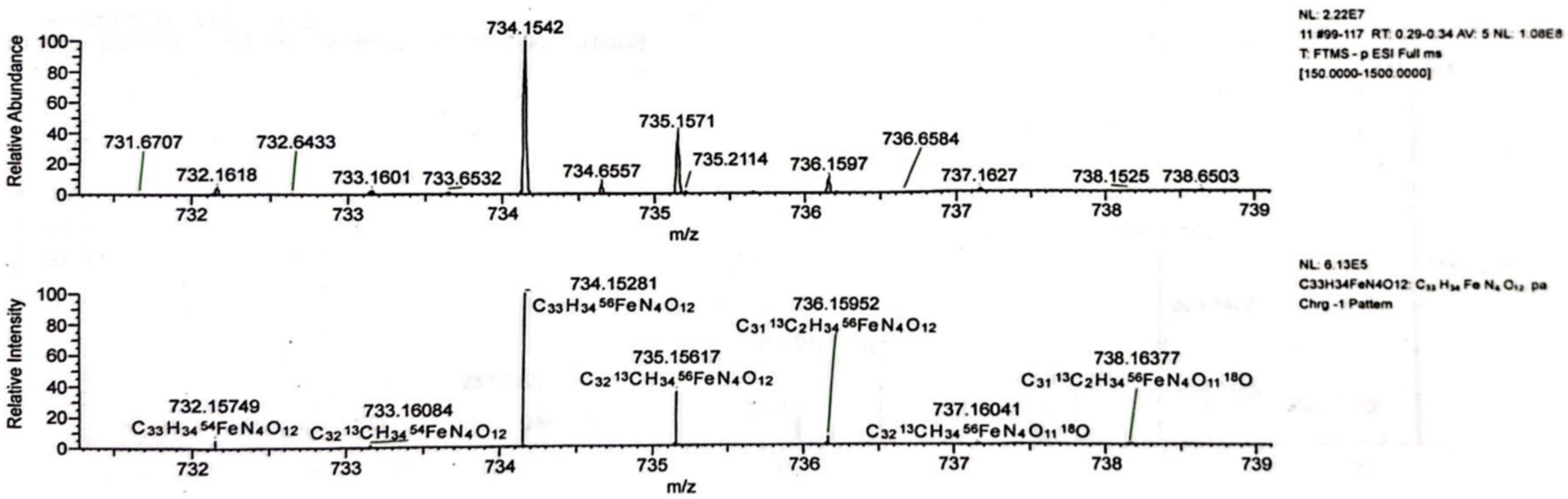

Figure S56. ESI (+) HR-MS spectrum of **[Fe(KK-COOH)]**$^{3-}$. ESI-HRMS calcd. for $C_{33}H_{32}FeN_4O_{12}$ $[M + 2H]^-$: *m/ z* 734.1517. Found 734.1512.

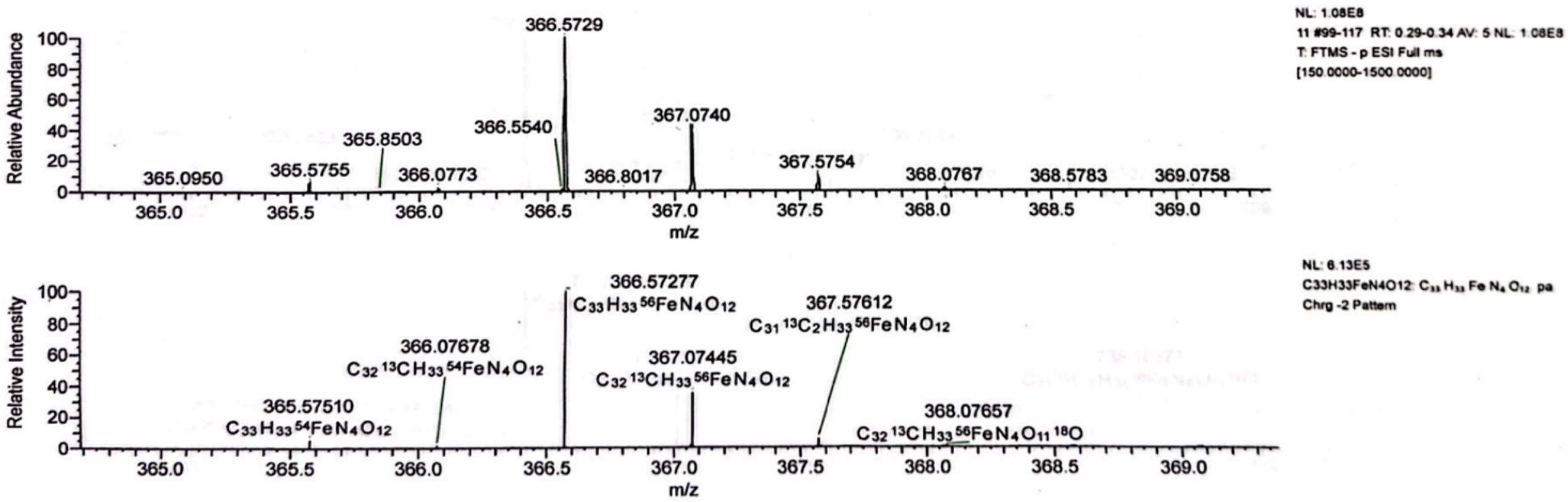

Figure S57. ESI (+) HR-MS spectrum of **[Fe(KK-COOH)]**$^{3-}$. ESI-HRMS calcd. for $C_{33}H_{32}FeN_4O_{12}$ $[M + H]^{2-}$: *m/ z* 366.5717. Found 366.5729.

[Ga(KK-COOH)]$^{3-}$, was synthesized by the procedure outlined in section **1.8.2.** Retention time= 6.64 min (Method C). ESI-HR-MS: Calcd. for $C_{33}H_{32}GaN_4O_{12}$ [M + 2H]$^-$: *m/ z* 747.1430 and [M + H]$^{2-}$: *m/ z* 373.0670. Found 747.1450 and 373.0686, respectively.

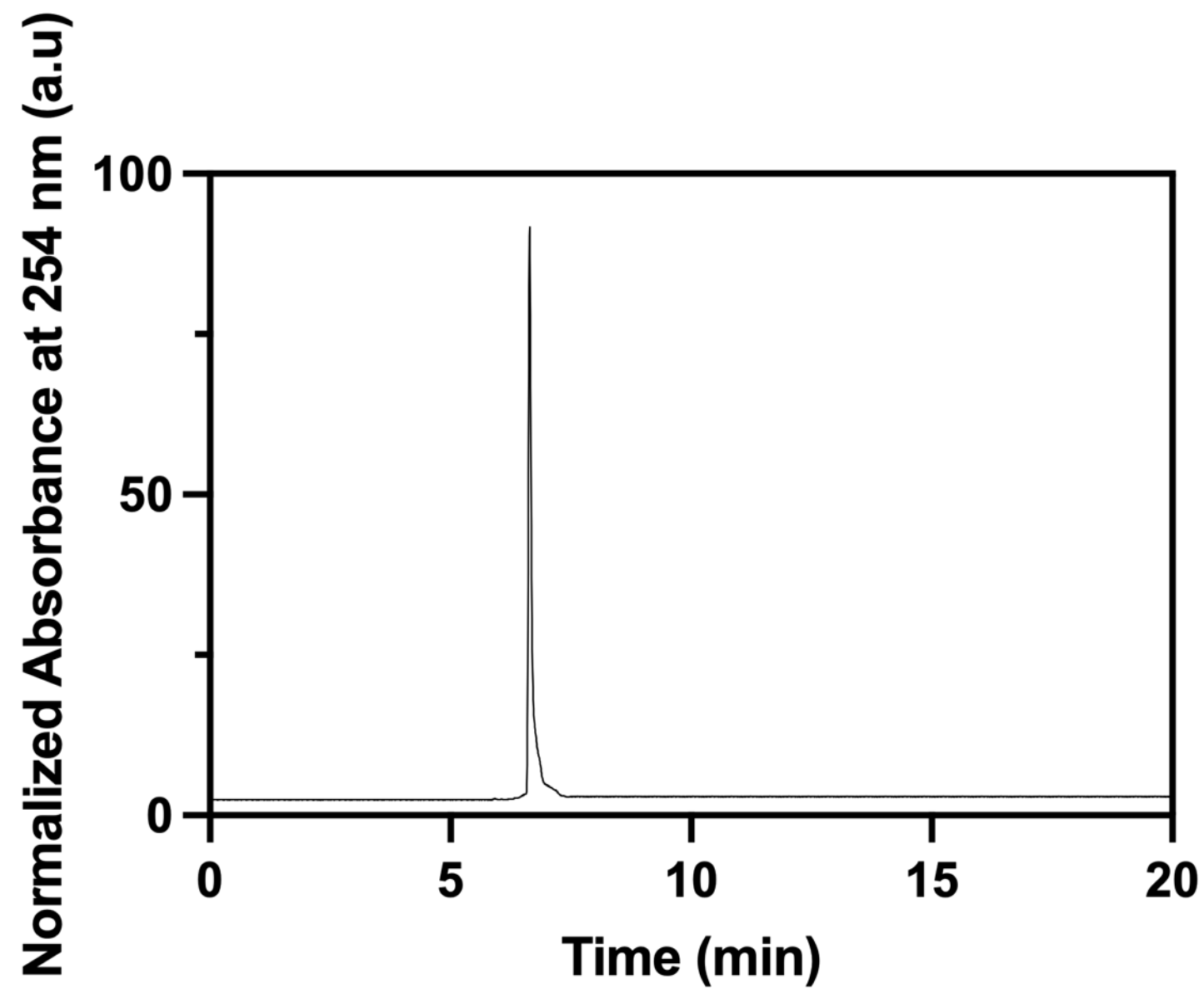

Figure S58. Normalized HPLC chromatogram of **[Ga(KK-COOH)]$^{3-}$**. Retention time: 6.64 min. (Method C).

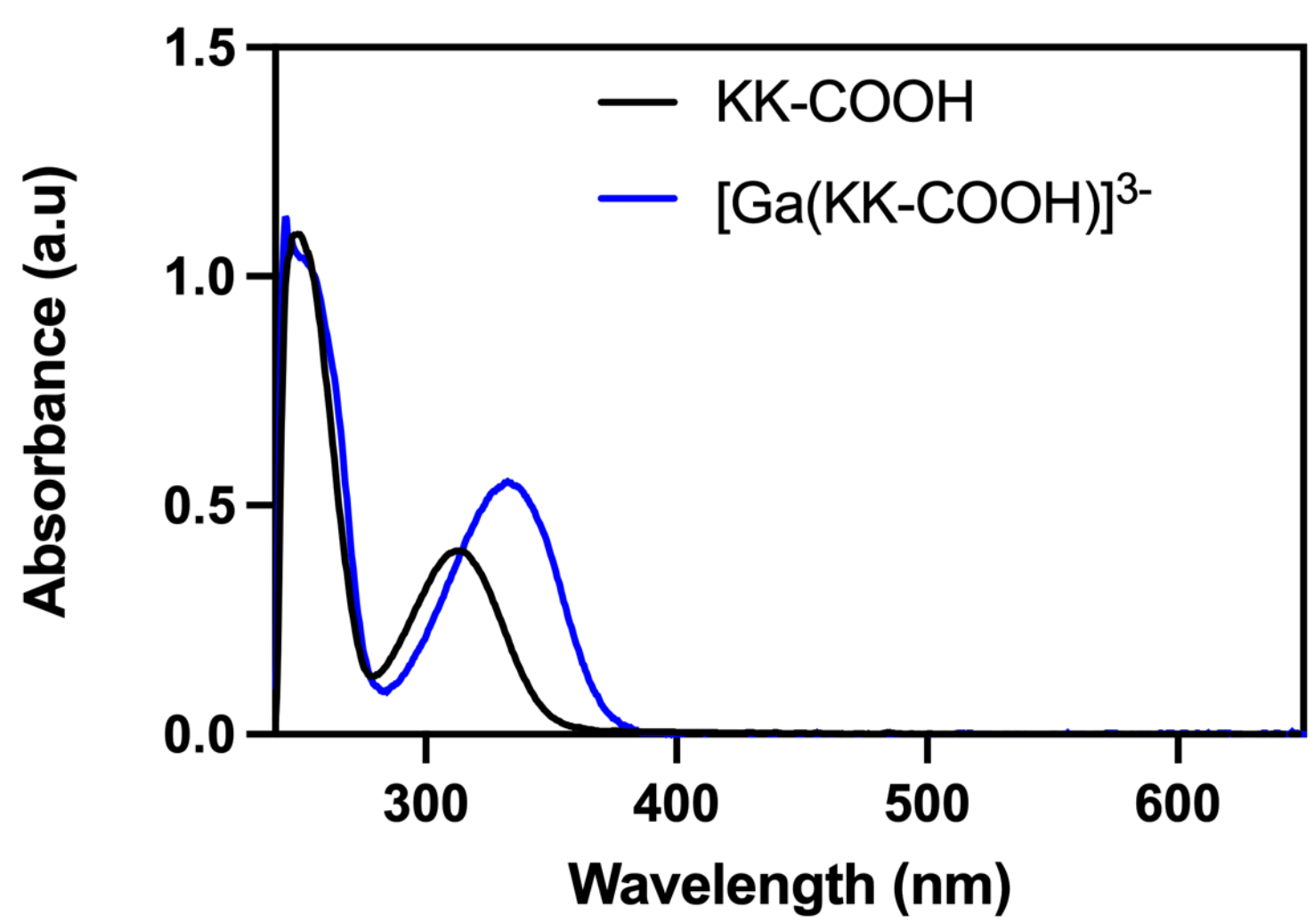

Figure S59. UV-vis spectrum of **[Ga(KK-COOH)]$^{3-}$**.

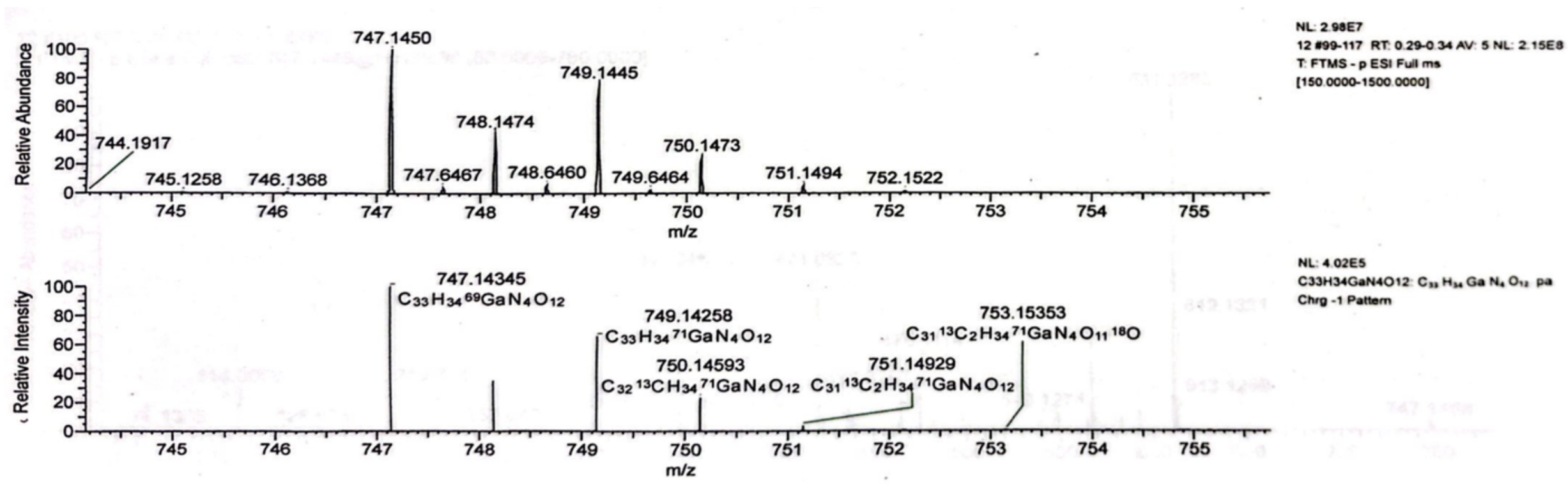

Figure S60. ESI (+) HR-MS spectrum of **[Ga(KK-COOH)]$^{3-}$**. ESI-HRMS calcd. for $C_{33}H_{32}GaN_4O_{12}$ [M + 2H]$^{-}$: *m/ z* 747.1430. Found 747.1450.

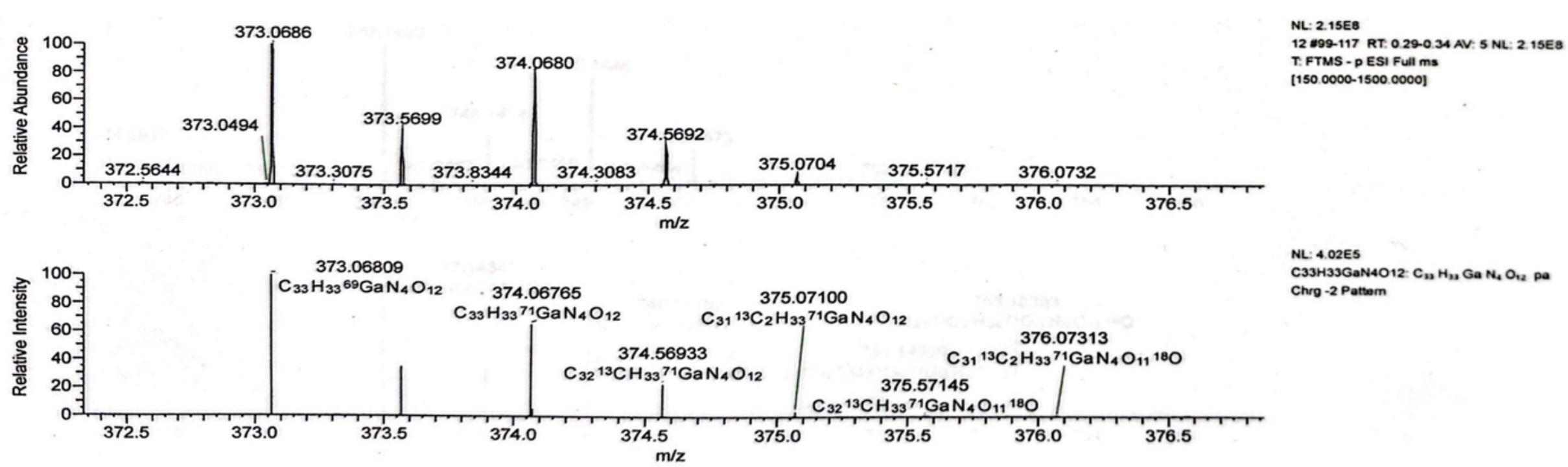

Figure S61. ESI (+) HR-MS spectrum of **[Ga(KK-COOH)]$^{3-}$**. ESI-HRMS calcd. for $C_{33}H_{32}GaN_4O_{12}$ [M + H]$^{2-}$: *m/ z* 373.0670. Found 373.0686.

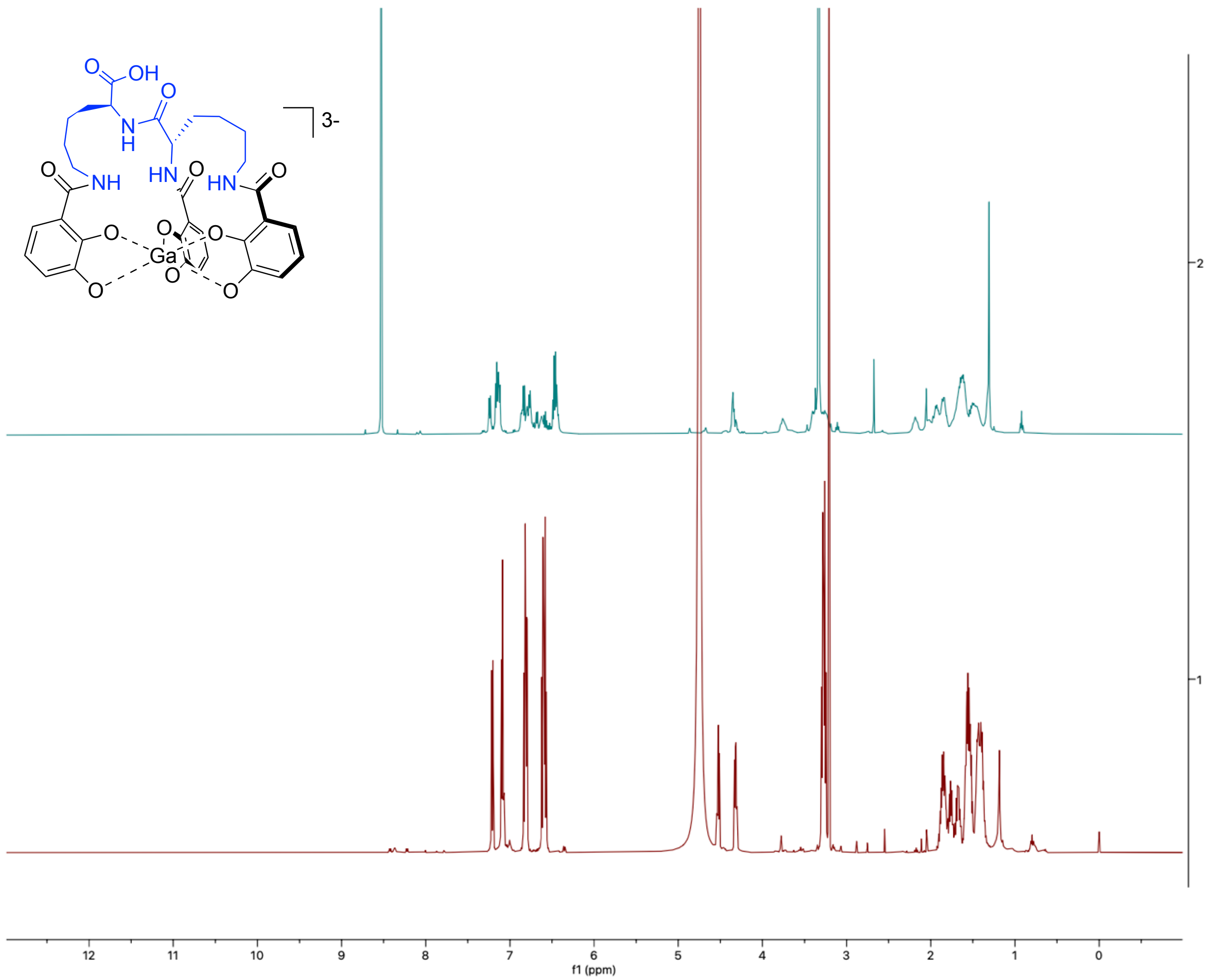

Figure S62. Full ^{1}H NMR (500 MHz, MeOD-d4, 298 K) of **KK-COOH** (maroon) and **[Ga(KK-COOH)]$^{3-}$** (teal).

1.9.2 KO-NH$_2$

[Fe(KO-NH$_2$)]$^{3-}$, was synthesized by the procedure outlined in section **1.8.1**. Retention time = 6.26 min (Method H). ESI-HR-MS: calcd. for $C_{32}H_{31}FeN_5O_{11}$ [M + 2H]$^-$: *m/z* 719.1521 and [M + H]$^{2-}$: *m/z* 359.0718. Found 719.1544 and 359.0733, respectively. d-d transition: λ = 550 nm.

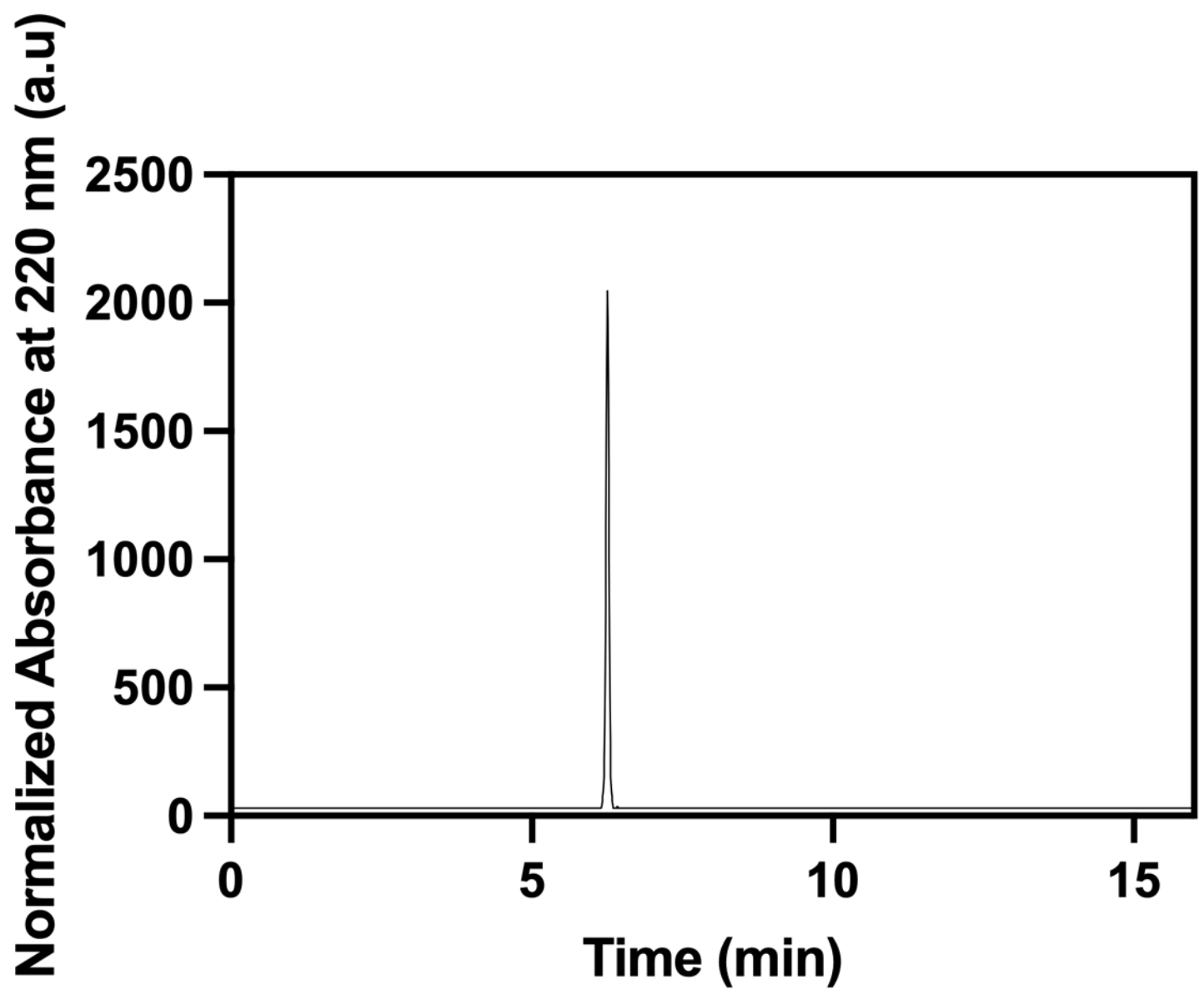

Figure S63. Normalized HPLC chromatogram of **[Fe(KO-NH$_2$)]$^{3-}$**. Retention time: 6.26 min. (Method H).

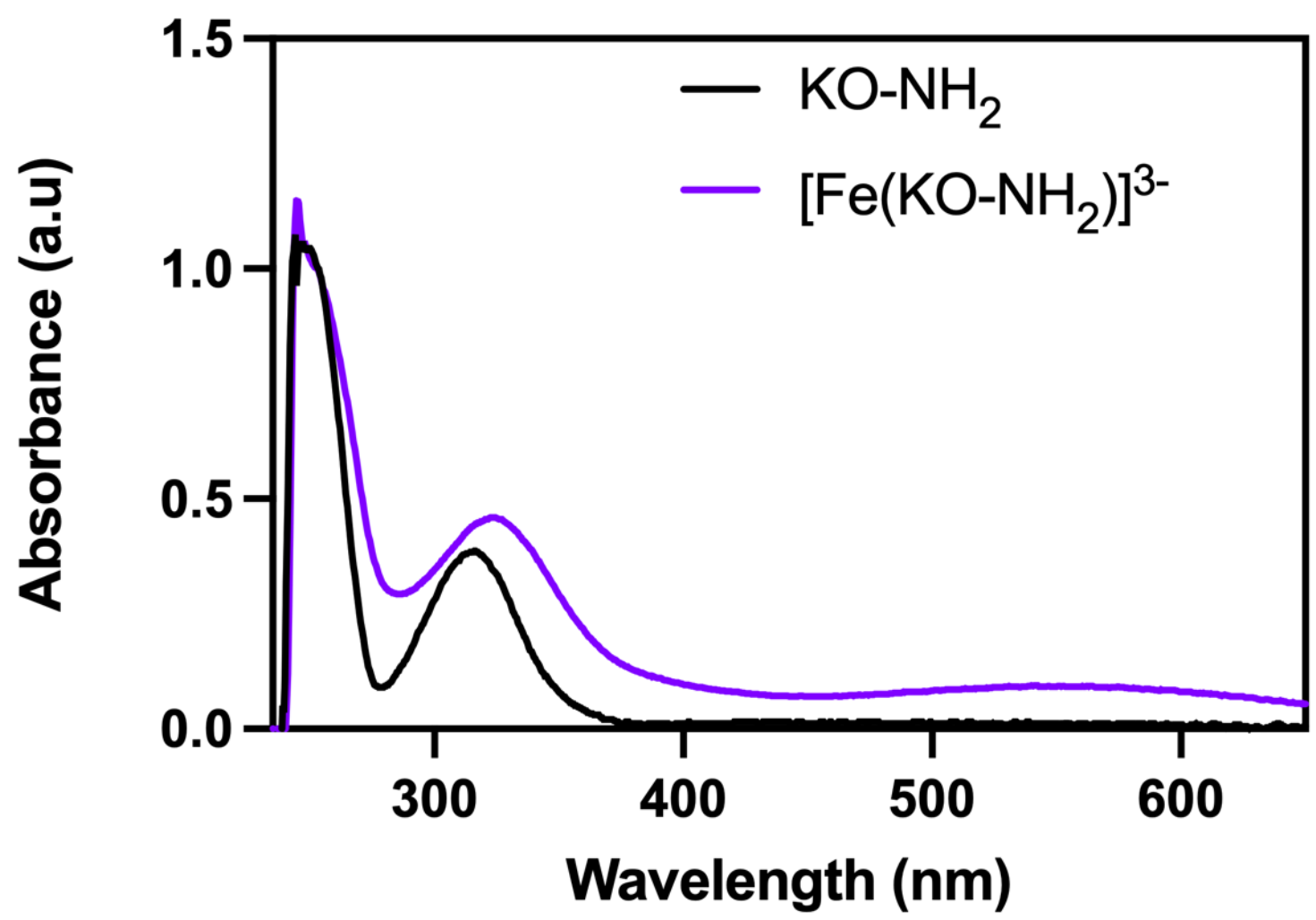

Figure S64. UV-vis spectrum of **[Fe(KO-NH$_2$)]$^{3-}$**. Observed LMTC, d-d transition λ = 550 nm.

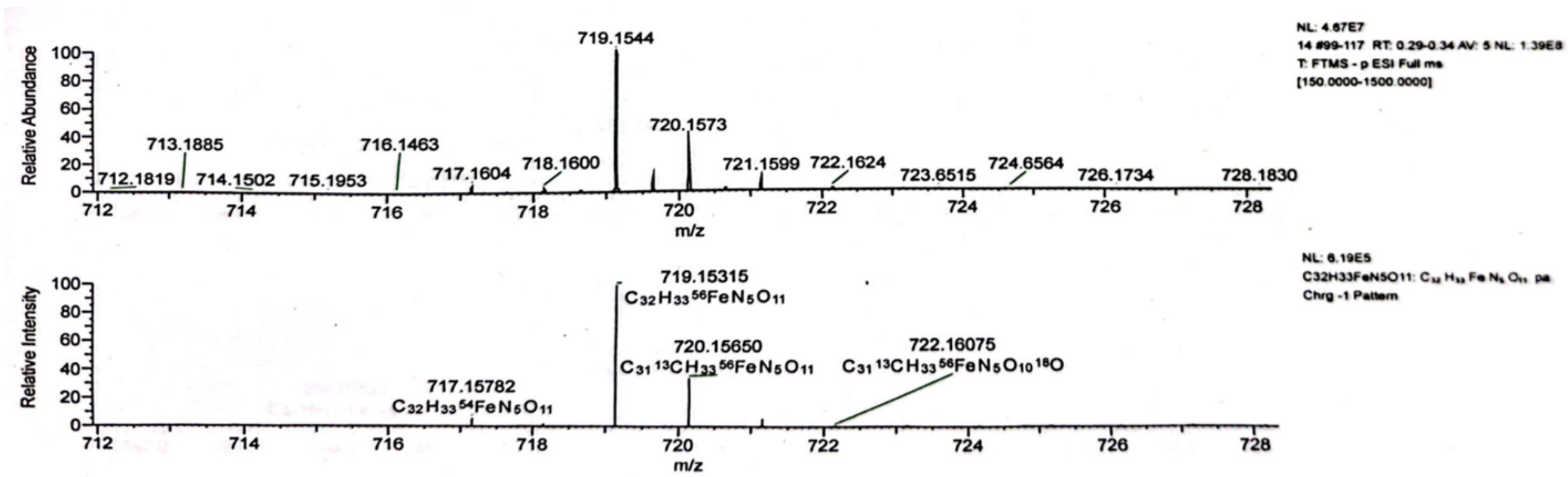

Figure S65. ESI (+) HR-MS spectrum of **[Fe(KO-NH$_2$)]$^{3-}$**. ESI-HRMS calcd. for $C_{32}H_{31}FeN_5O_{11}$ [M + 2H]$^{-}$: *m/z* 719.1521. Found 719.1544.

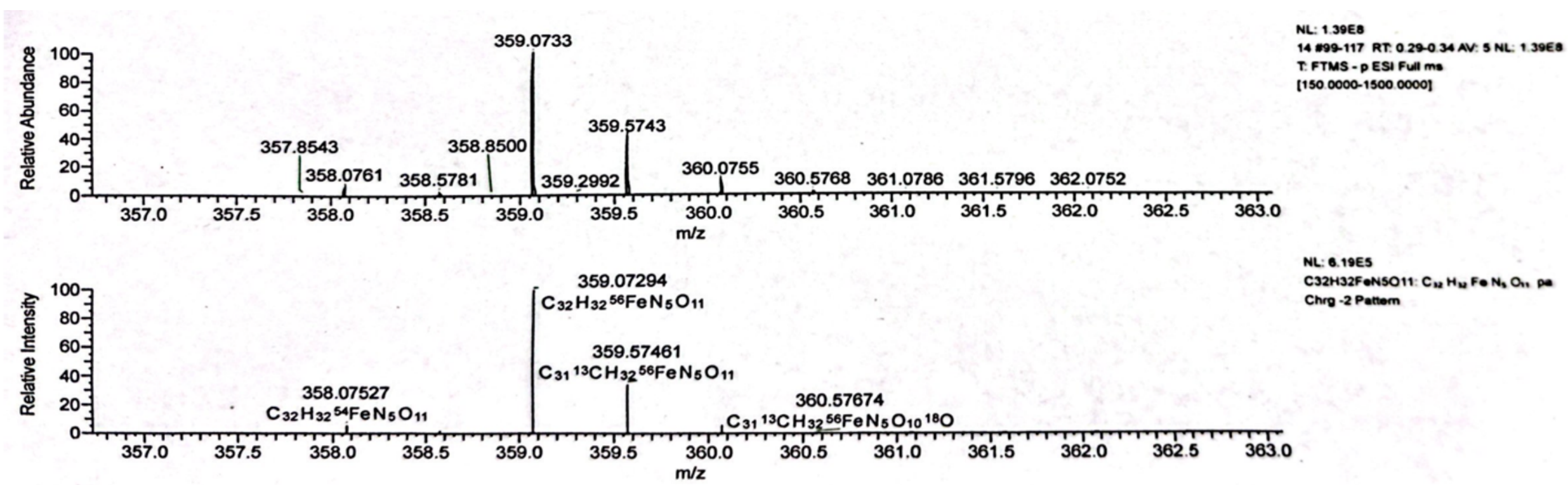

Figure S66. ESI (+) HR-MS spectrum of **[Fe(KO-NH$_2$)]$^{3-}$**. ESI-HRMS calcd. for $C_{32}H_{31}FeN_5O_{11}$ [M + H]$^{2-}$: *m/z* 359.0718. Found 359.0733.

[Ga(KO-NH₂)]³⁻, was synthesized by the procedure outlined in section **1.8.2**. Retention time = 6.90 min (Method C). ESI-HR-MS: calcd. for $C_{32}H_{31}GaN_5O_{11}$ [M + 2H]⁻: *m/ z* 732.1427 and [M + H]²⁻: *m/ z* 365.5672. Found 732.1451 and 365.5687, respectively.

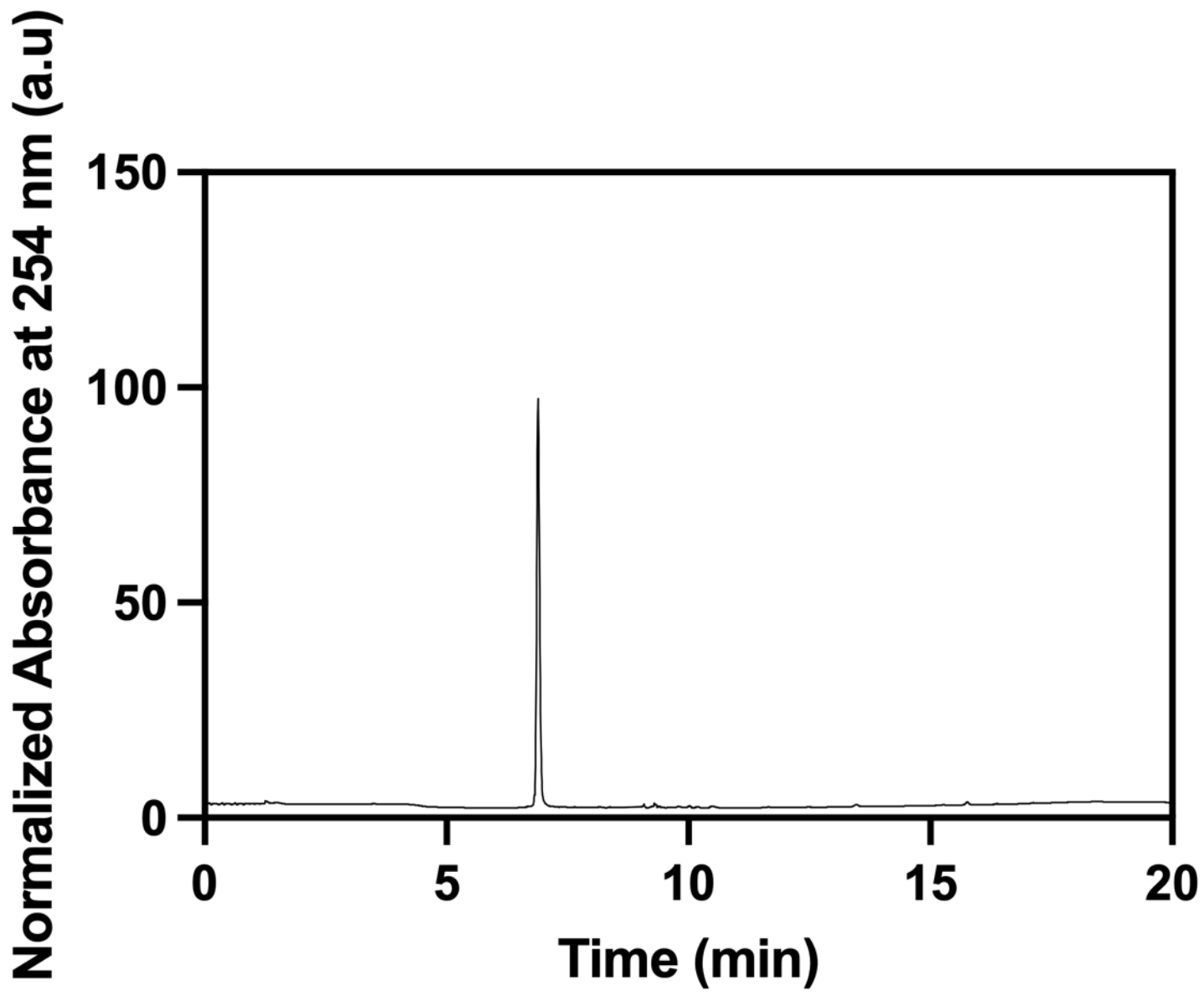

Figure S67. Normalized HPLC chromatogram of **[Ga(KO-NH₂)]³⁻**. Retention time: 6.90 min. (Method C).

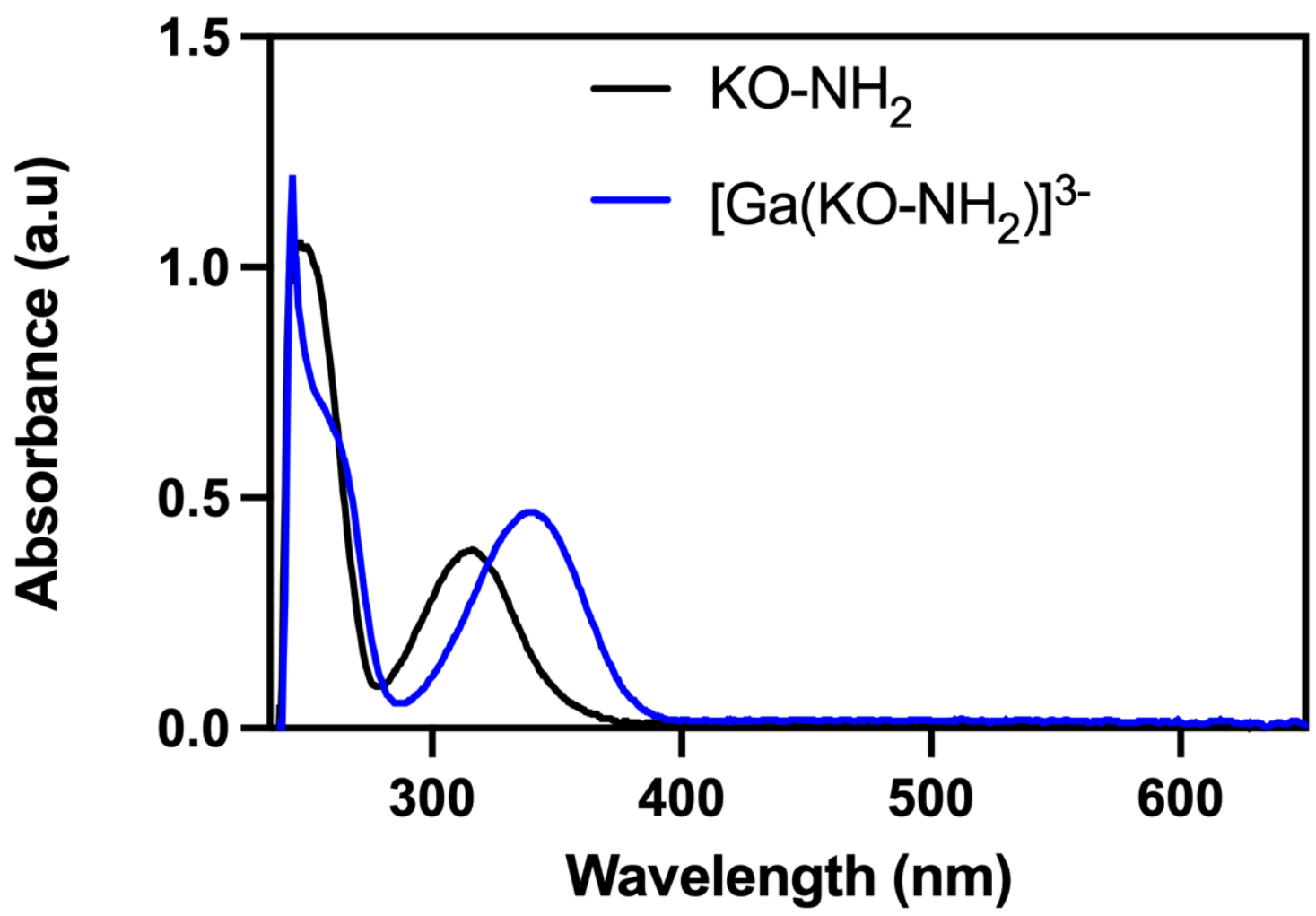

Figure S68. UV-vis spectrum of **[Ga(KO-NH₂)]³⁻**.

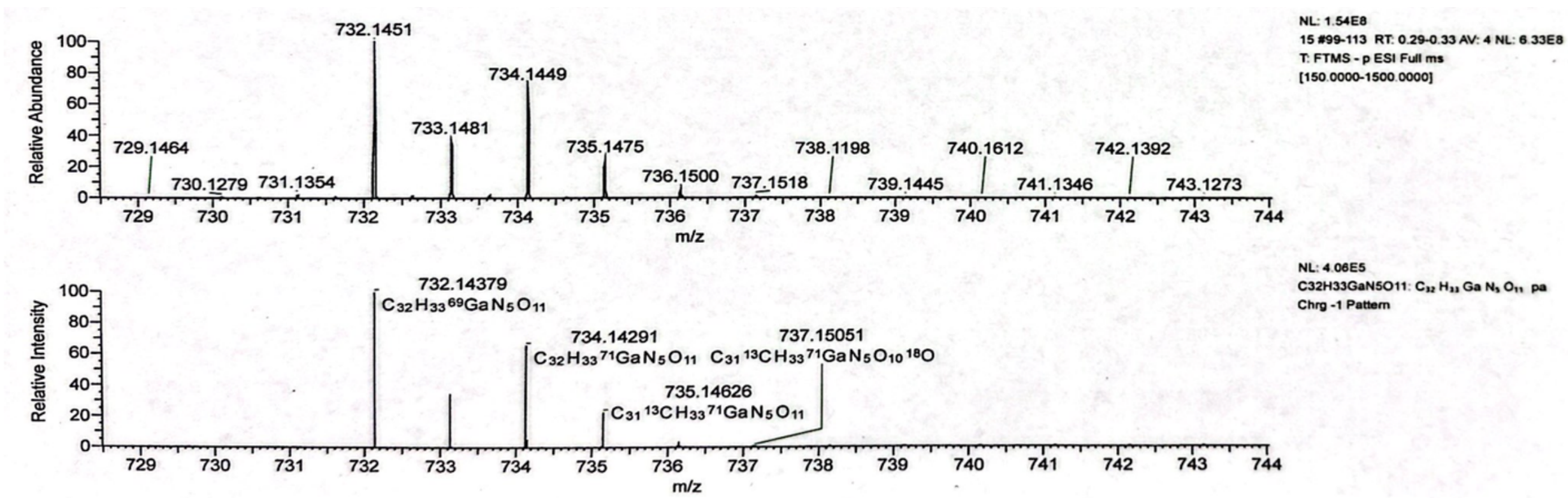

Figure S69. ESI (+) HR-MS spectrum of **[Ga(KO-NH$_2$)]$^{3-}$**. ESI-HRMS calcd. for $C_{32}H_{31}GaN_5O_{11}$ $[M + 2H]^-$: *m/ z* 732.1427. Found 732.1451.

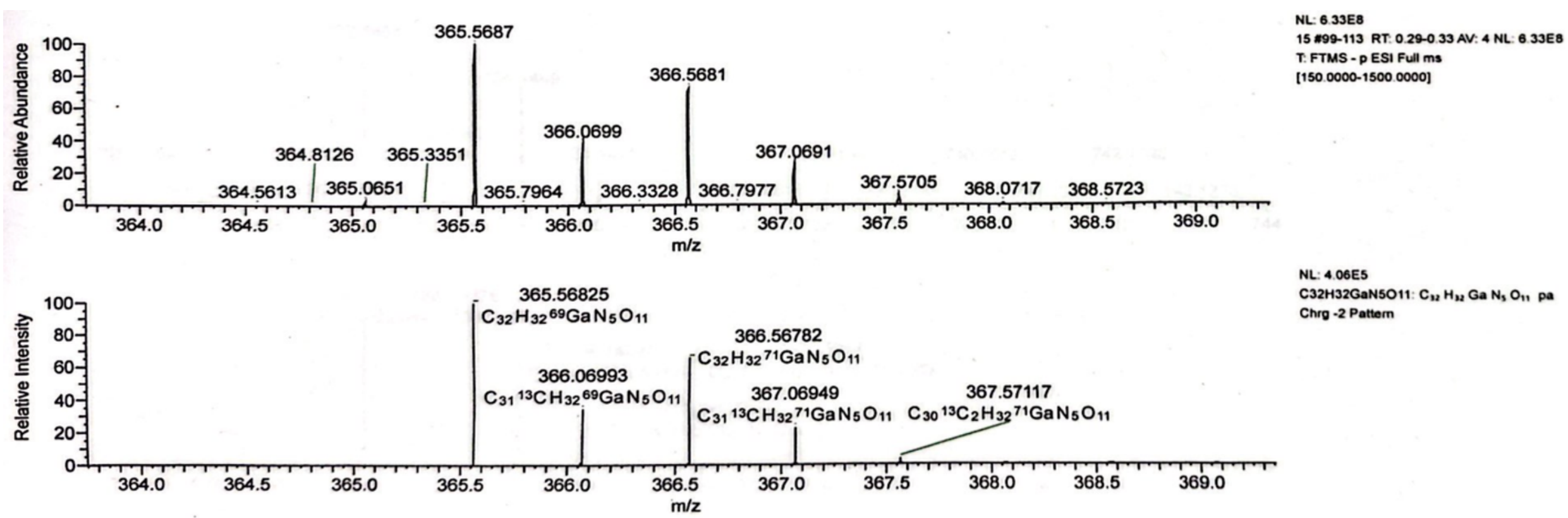

Figure S70. ESI (+) HR-MS spectrum of **[Ga(KO-NH$_2$)]$^{3-}$**. ESI-HRMS calcd. for $C_{32}H_{31}GaN_5O_{11}$ $[M + H]^{2-}$: *m/ z* 365.5672. Found 365.5687.

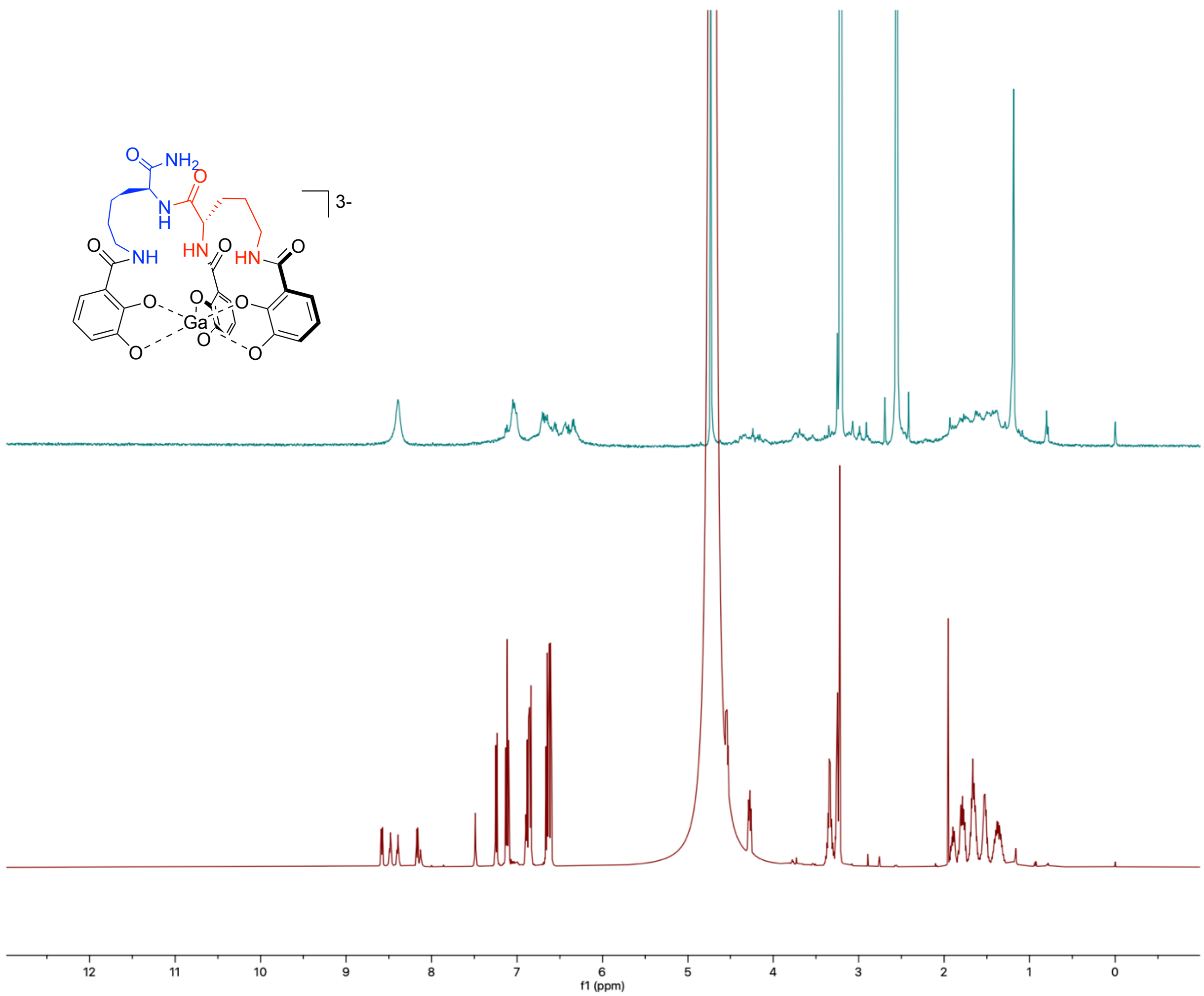

Figure S71. Full ^{1}H NMR (500 MHz, MeOD-d4, 298 K) of **KO-NH$_2$** (maroon) and **[Ga(KO-NH$_2$)]$^{3-}$** (teal).

1.9.3 KO-COOH

[Fe(KO-COOH)]$^{3-}$, was synthesized by the procedure outlined in section **1.8.1**. Retention time = 6.49 min (Method H). ESI-HR-MS: calcd. for $C_{32}H_{30}N_4O_{12}Fe$ $[M + 2H]^-$: *m/z* 720.1361 and $[M + H]^{2-}$: *m/z* 359.5638. Found 720.1382 and 359.5654, respectively. d-d transition: λ = 550 nm.

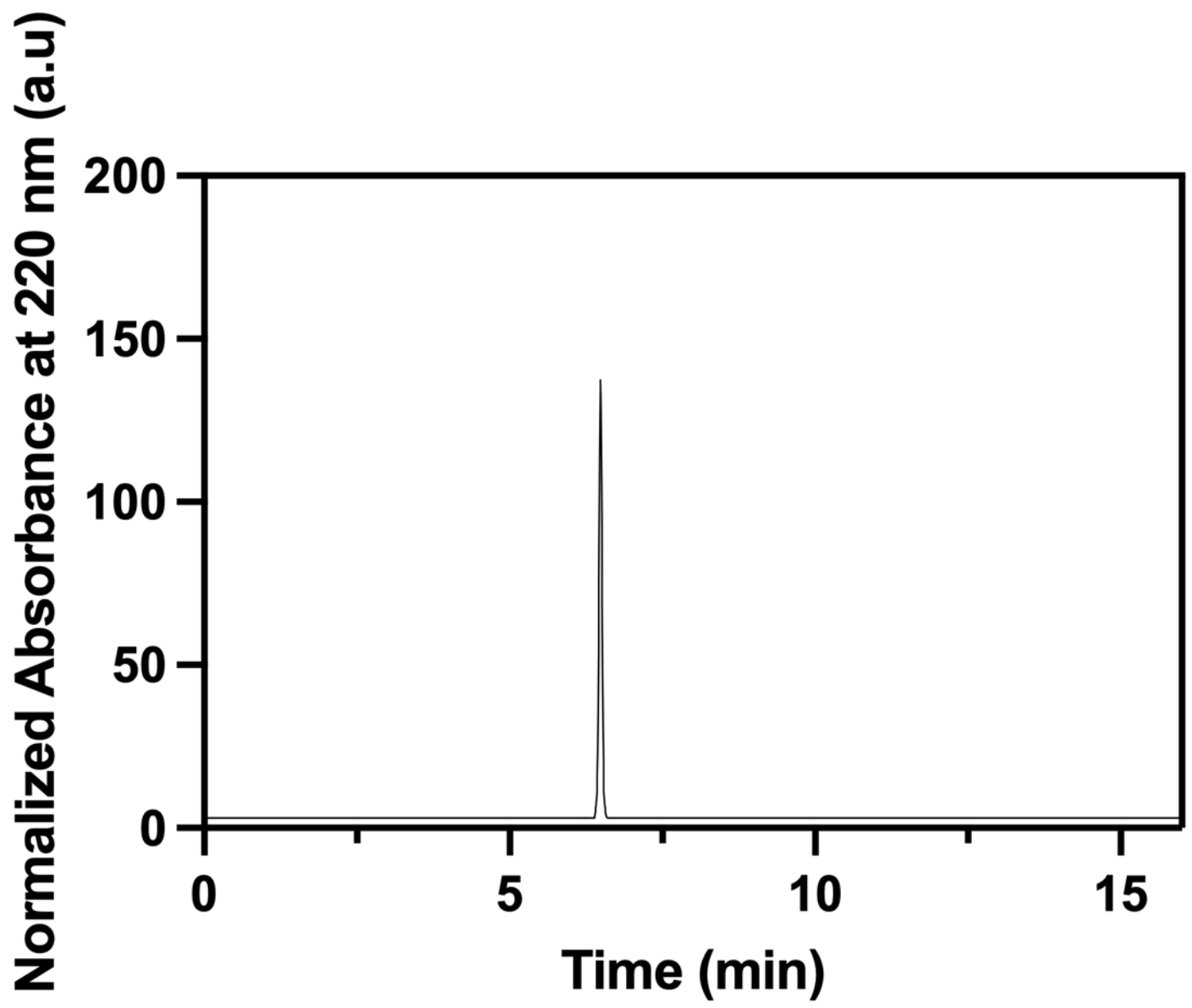

Figure S72. Normalized HPLC chromatogram of **[Fe(KO-COOH)]**$^{3-}$. Retention time: 6.49 min. (Method H).

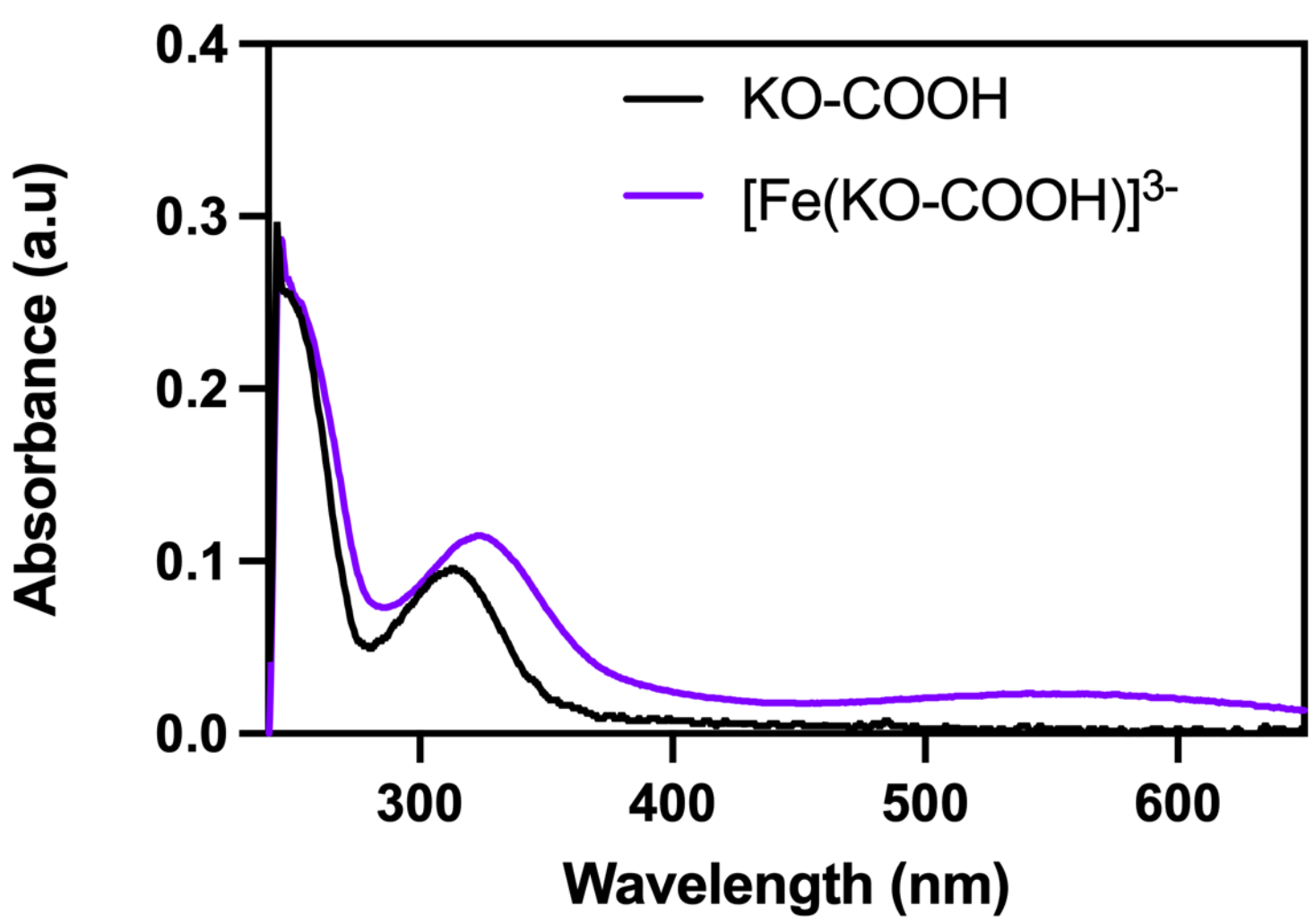

Figure S73. UV-vis spectrum of **[Fe(KO-COOH)]**$^{3-}$. Observed LMTC, d-d transition λ = 550 nm.

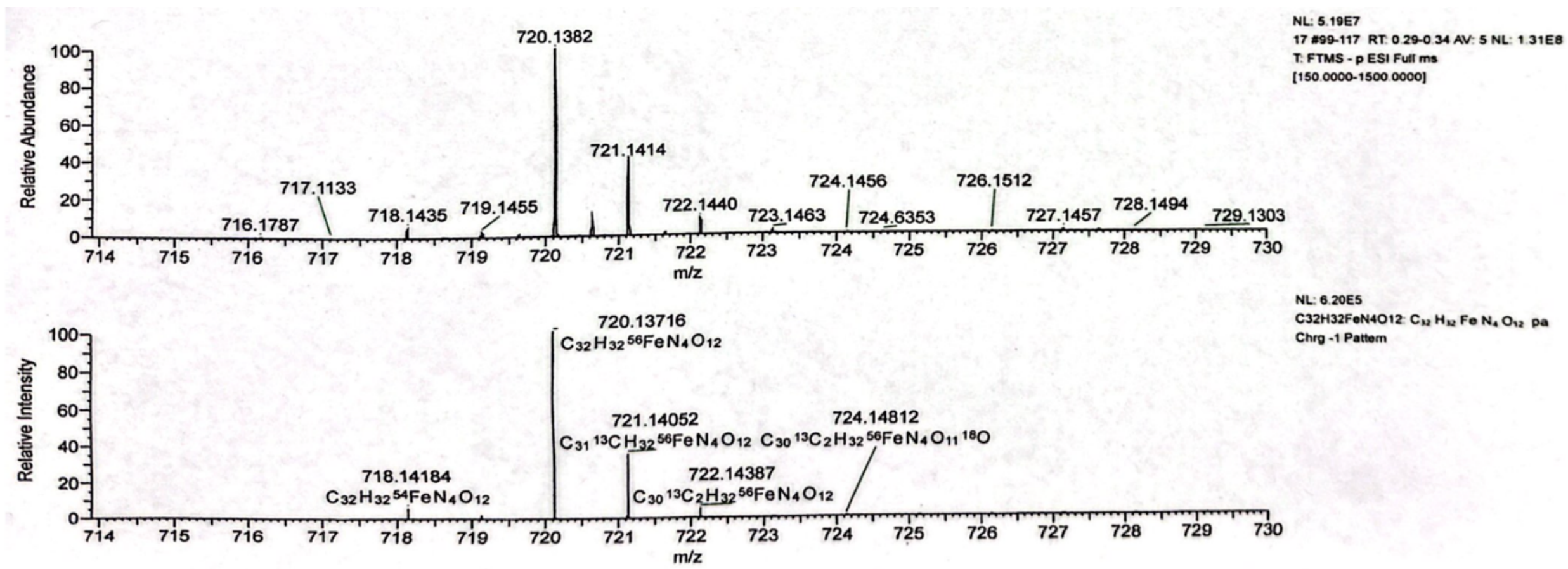

Figure S74. ESI (+) HR-MS spectrum of **[Fe(KO-COOH)]$^{3-}$**. ESI-HRMS calcd. for $C_{32}H_{30}FeN_4O_{12}$ $[M + 2H]^-$: *m/z* 720.1361. Found 720.1382.

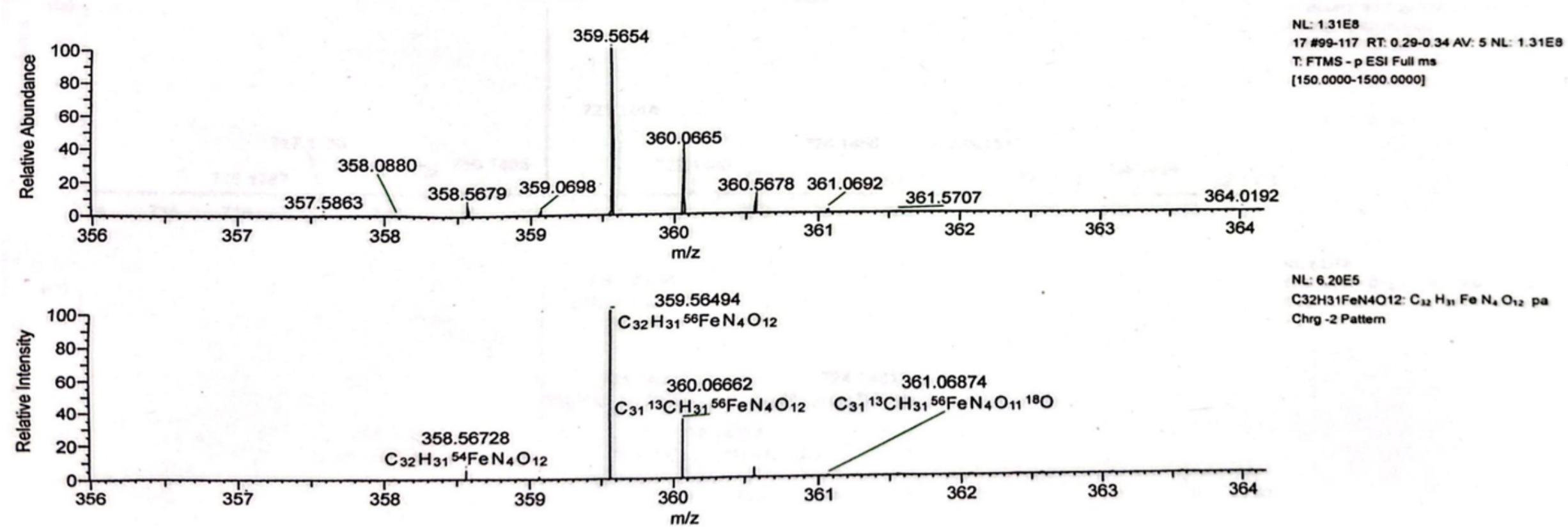

Figure S75. ESI (+) HR-MS spectrum of **[Fe(KO-COOH)]$^{3-}$**. ESI-HRMS calcd. for $C_{32}H_{30}FeN_4O_{12}$ $[M + H]^{2-}$: *m/z* 359.5638. Found 359.5654.

[Ga(KO-COOH)]$^{3-}$, was synthesized by the procedure outlined in section **1.8.2**. Retention time = 6.03 min (Method C). ESI-HR-MS calcd. for $C_{32}H_{30}GaN_4O_{12}$ [M + 2H]$^{-}$: *m/ z* 733.1267 and [M + H]$^{2-}$: *m/ z* 366.0592. Found 733.1291 and 366.0607.

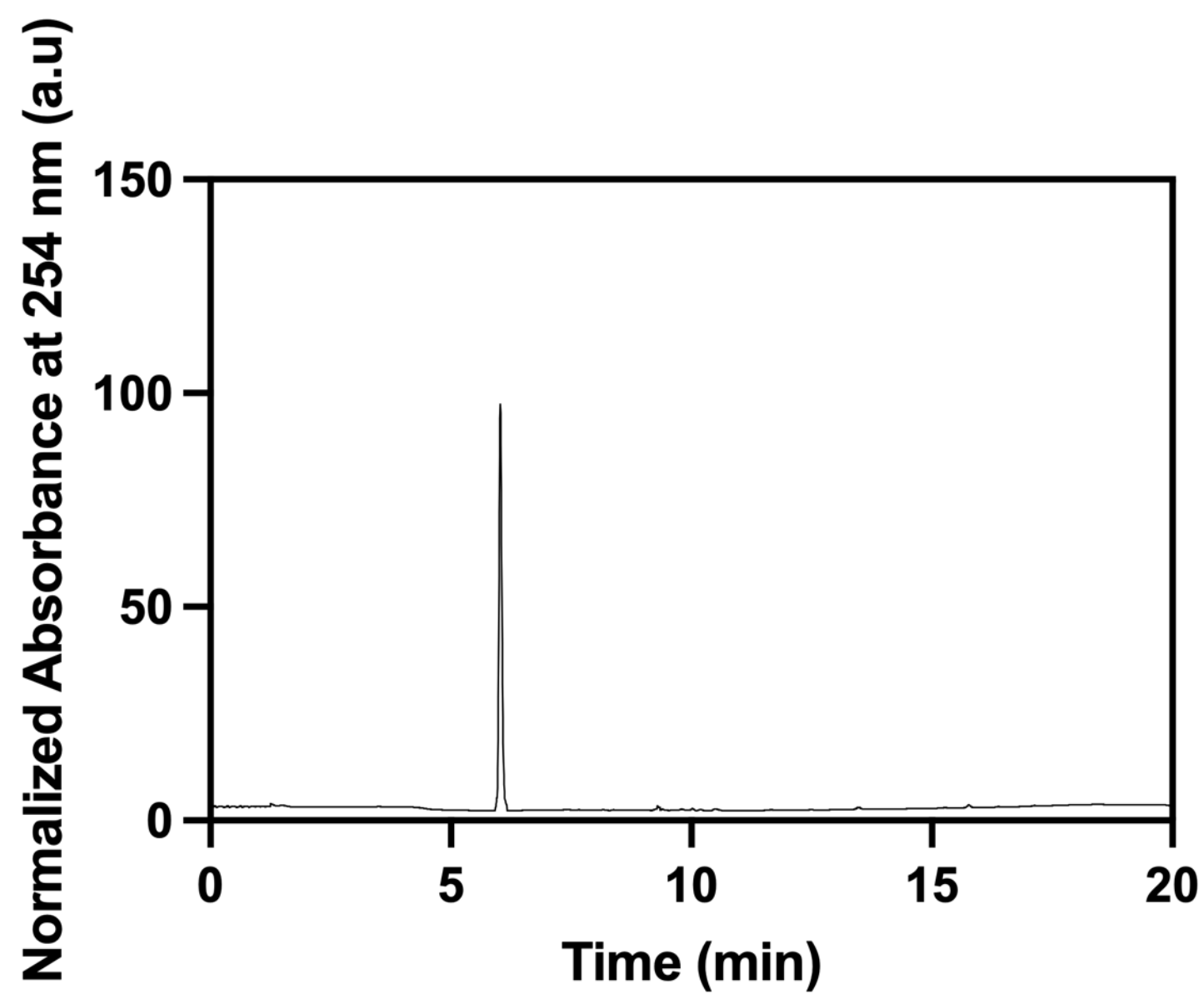

Figure S76. Normalized HPLC chromatogram of **[Ga(KO-COOH)]$^{3-}$**. Retention time: 6.03 min. (Method C).

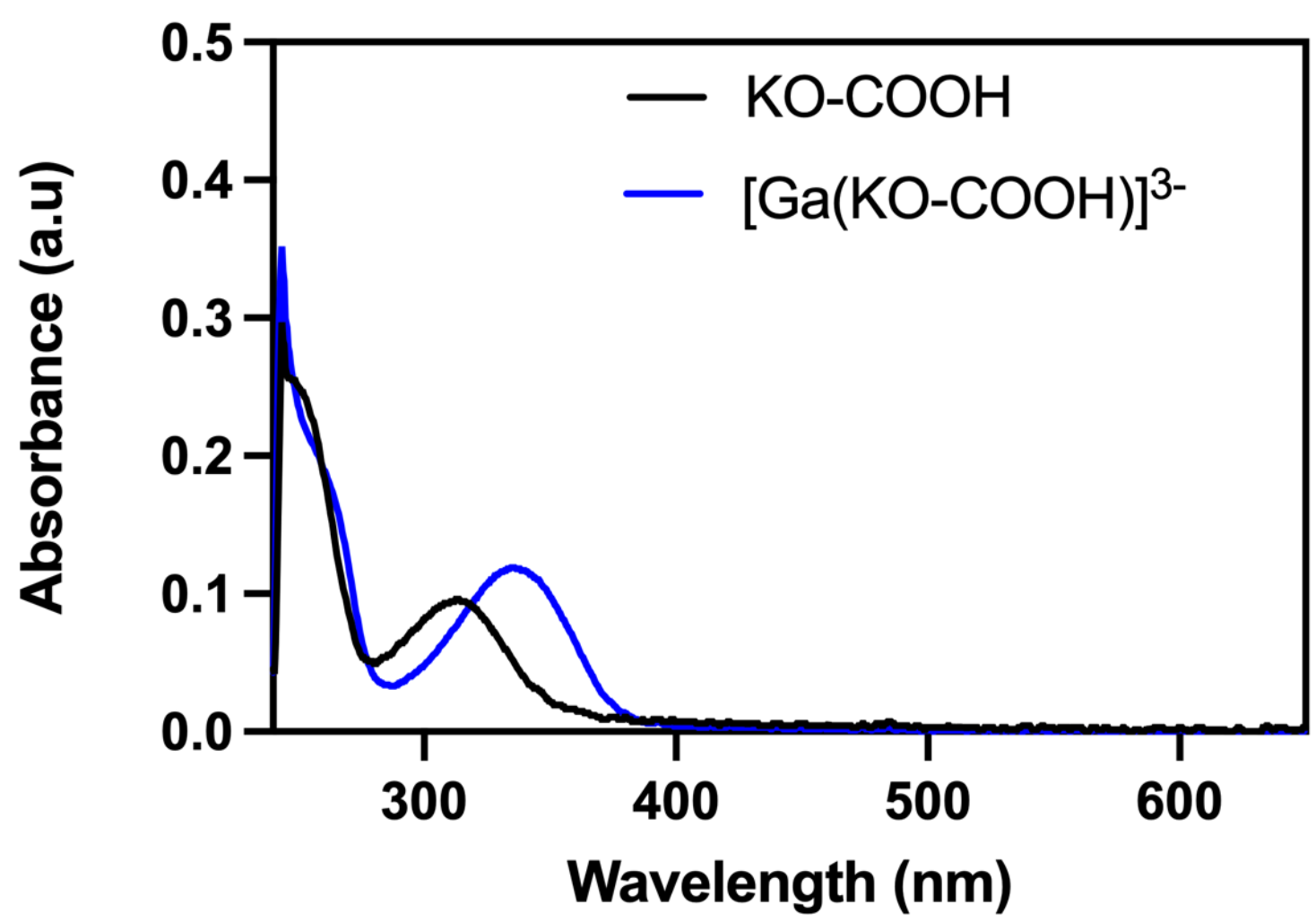

Figure S77. UV-vis spectrum of **[Ga(KO-COOH)]$^{3-}$**.

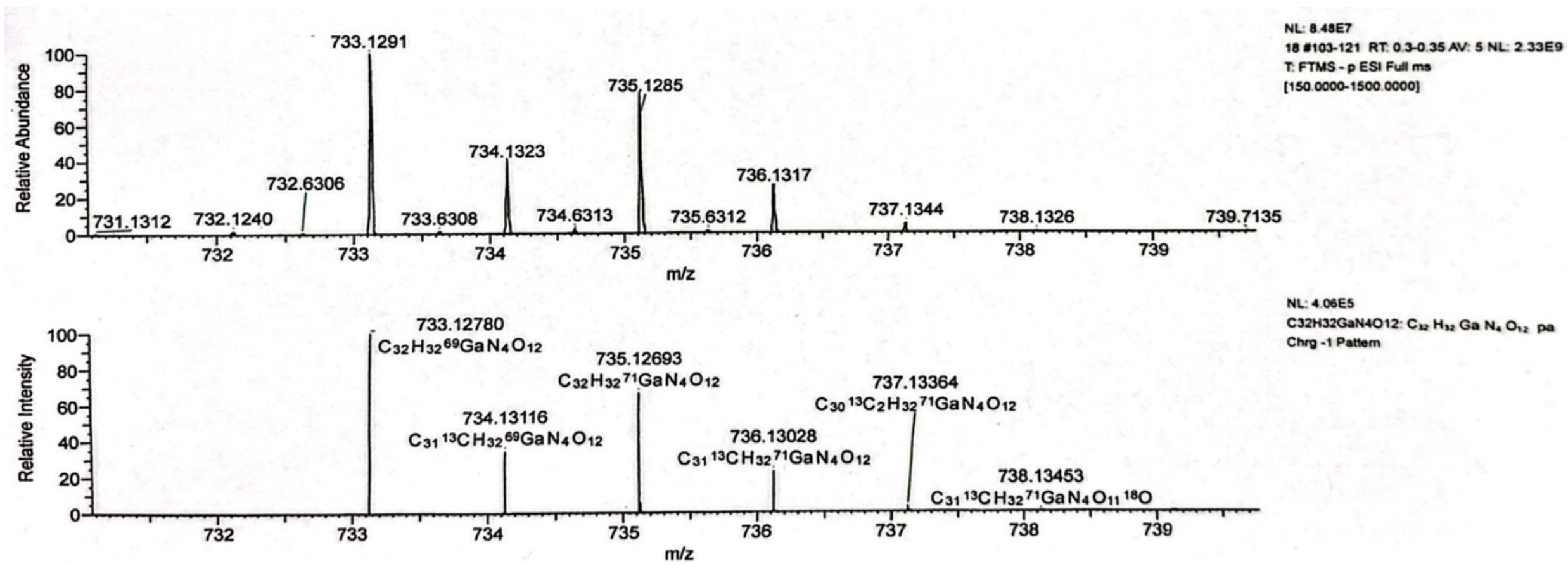

Figure S78. ESI (+) HR-MS spectrum of **[Ga(KO-COOH)]**$^{3-}$. ESI-HRMS calcd. for $C_{32}H_{30}GaN_4O_{12}$ $[M + 2H]^-$: *m/z* 733.1267. Found 733.1291.

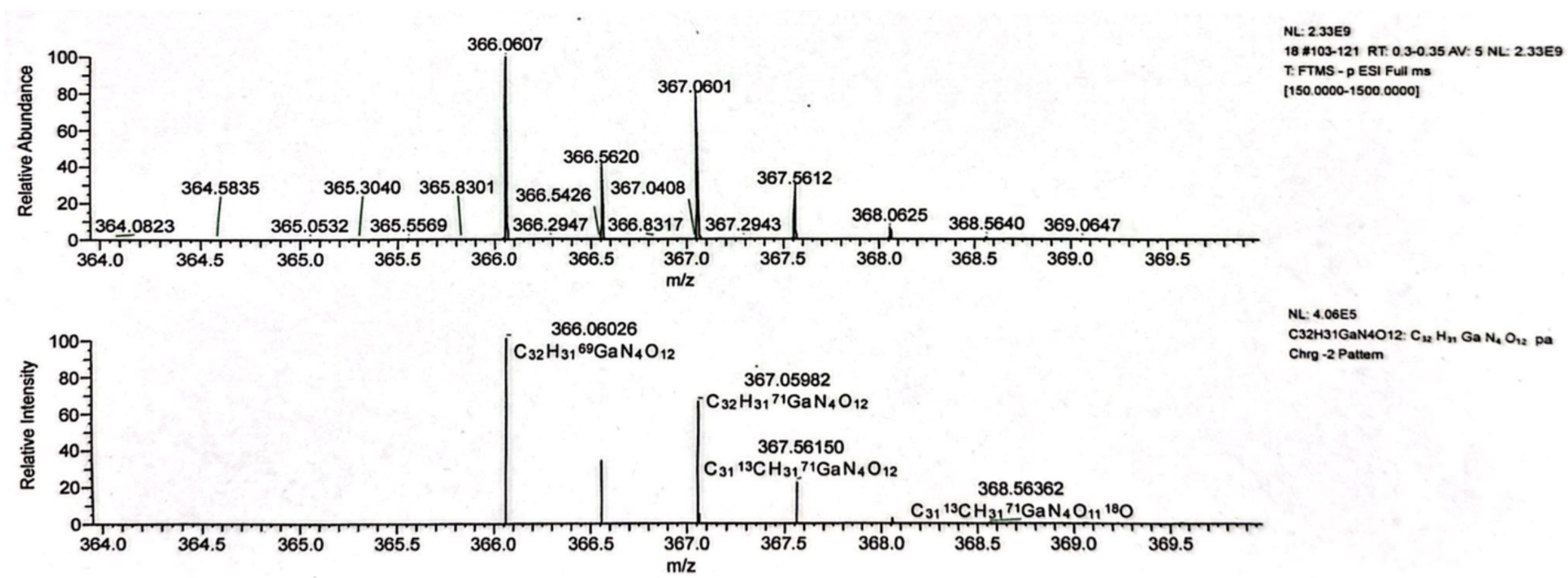

Figure S79. ESI (+) HR-MS spectrum of **[Ga(KO-COOH)]**$^{3-}$. ESI-HRMS calcd. for $C_{32}H_{30}GaN_4O_{12}$ $[M + H]^{2-}$: *m/z* 366.0592. Found 366.0607.

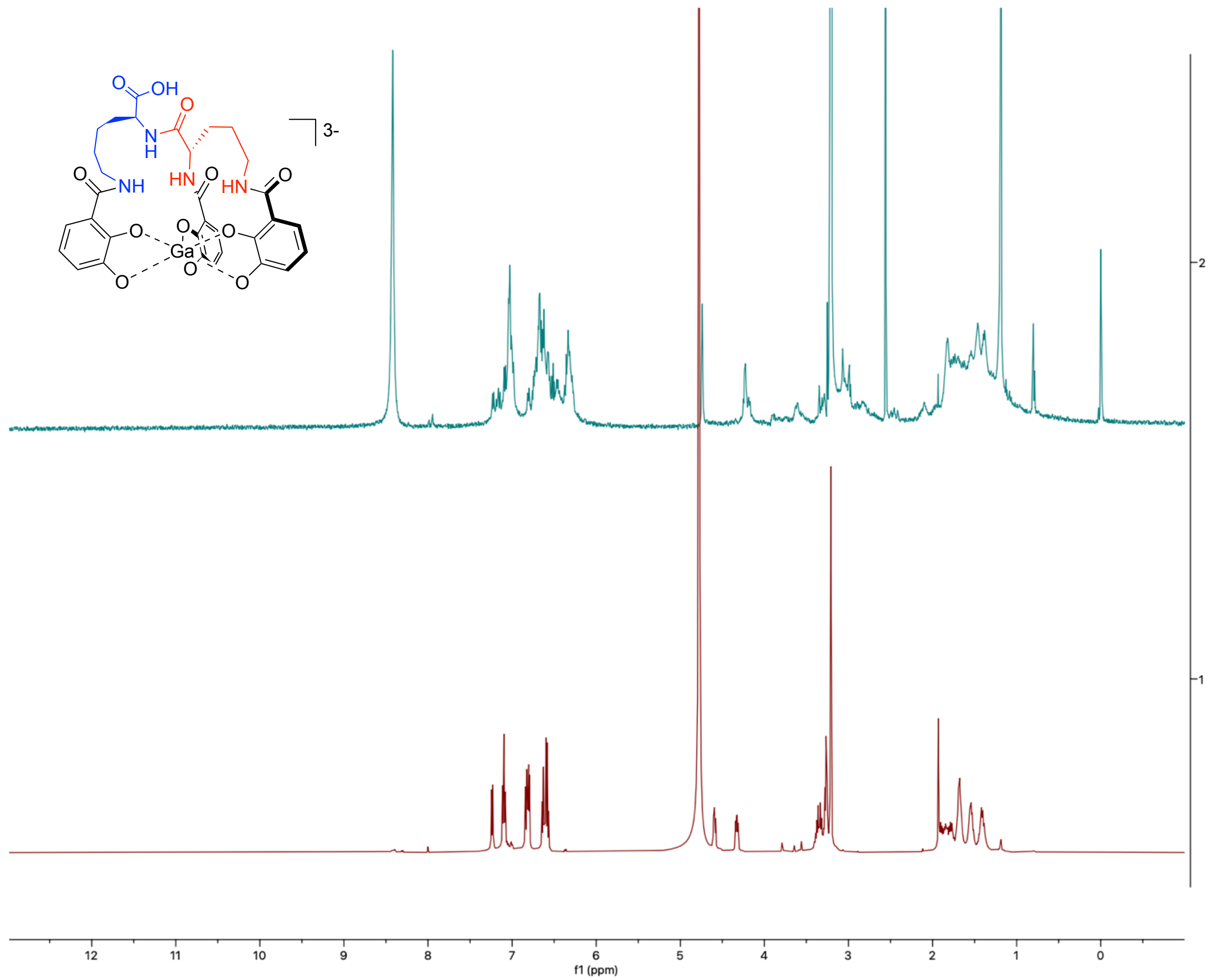

Figure S80. Full ^{1}H NMR (500 MHz, MeOD-d4, 298 K) of **KO-COOH** (maroon) and **[Ga(KO-COOH)]$^{3-}$** (teal).

1.9.4 OK-COOH

[Fe(OK-COOH)]$^{3-}$, was synthesized by the procedure outlined in section **1.8.1**. Retention time = 6.51 min (Method H). ESI-HR-MS: calcd. for $C_{32}H_{30}N_4O_{12}Fe$ $C_{32}H_{30}FeN_4O_{12}$ [M + 2H]$^-$: *m/z* 720.1361 and [M + H]$^{2-}$: *m/z* 359.5638. Found 720.1385 and 359.5654, respectively. d-d transition: λ = 550 nm.

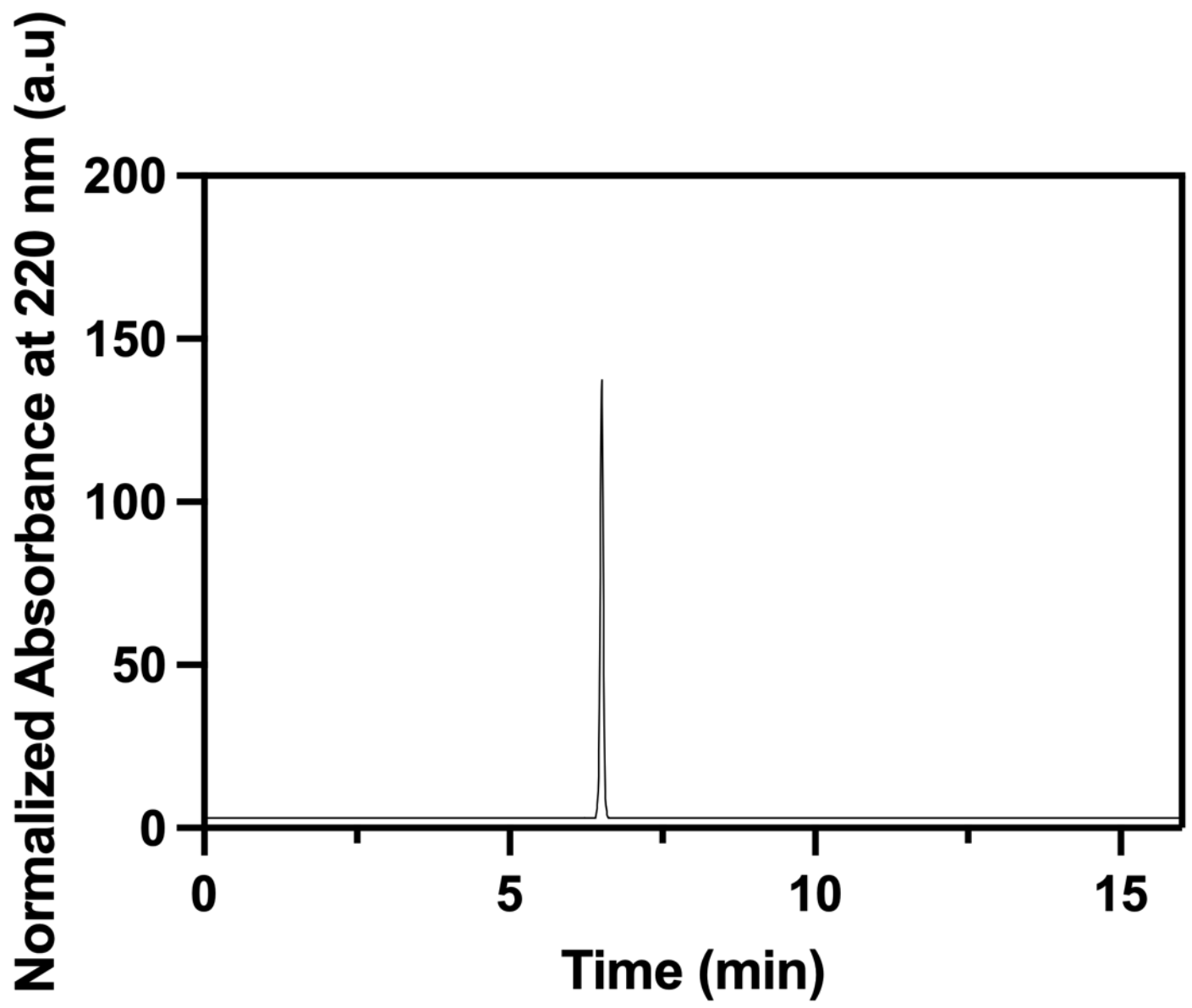

Figure S81. Normalized HPLC chromatogram of **[Fe(OK-COOH)]**$^{3-}$. Retention time: 6.51 min. (Method H).

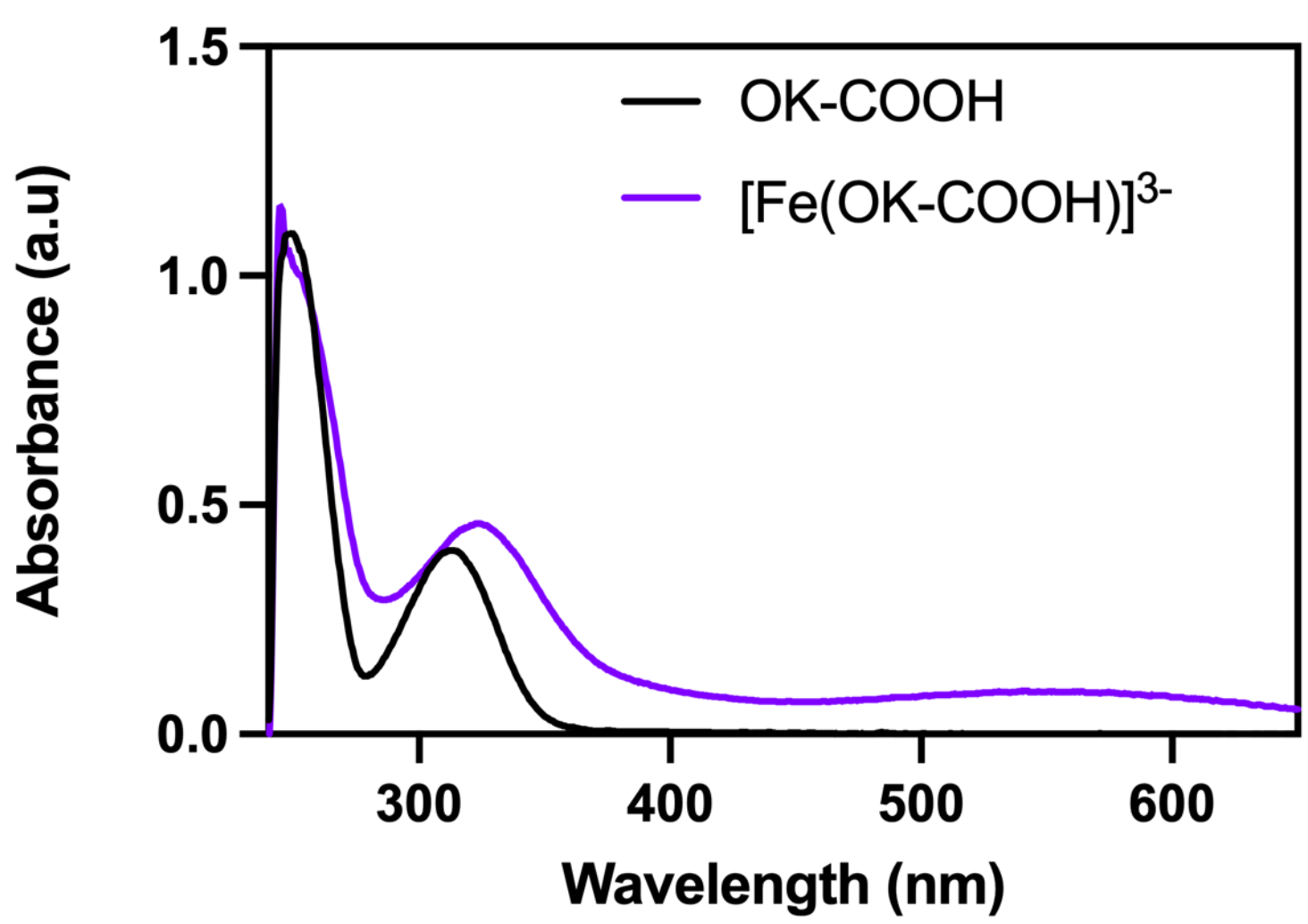

Figure S82. UV-vis spectrum of **[Fe(OK-COOH)]**$^{3-}$. Observed LMTC, d-d transition λ = 550 nm.

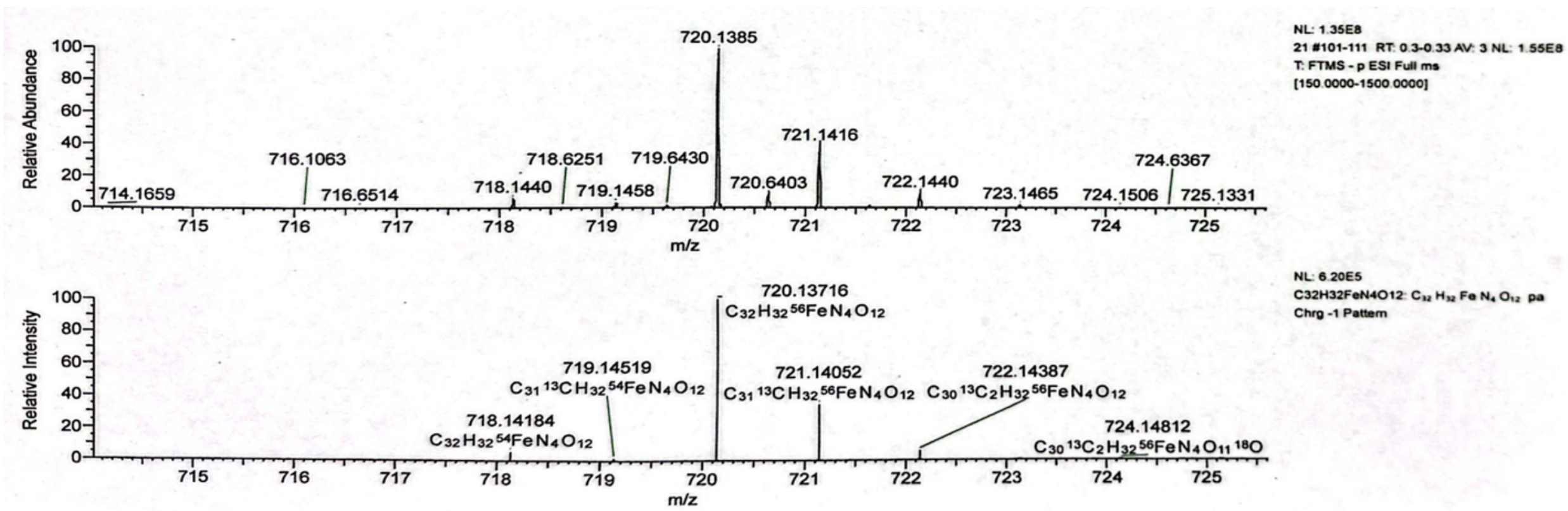

Figure S83. ESI (+) HR-MS spectrum of **[Fe(OK-COOH)]$^{3-}$**. ESI-HRMS calcd. for $C_{32}H_{30}FeN_4O_{12}$ $[M + 2H]^-$: *m/z* 720.1361. Found 720.1385.

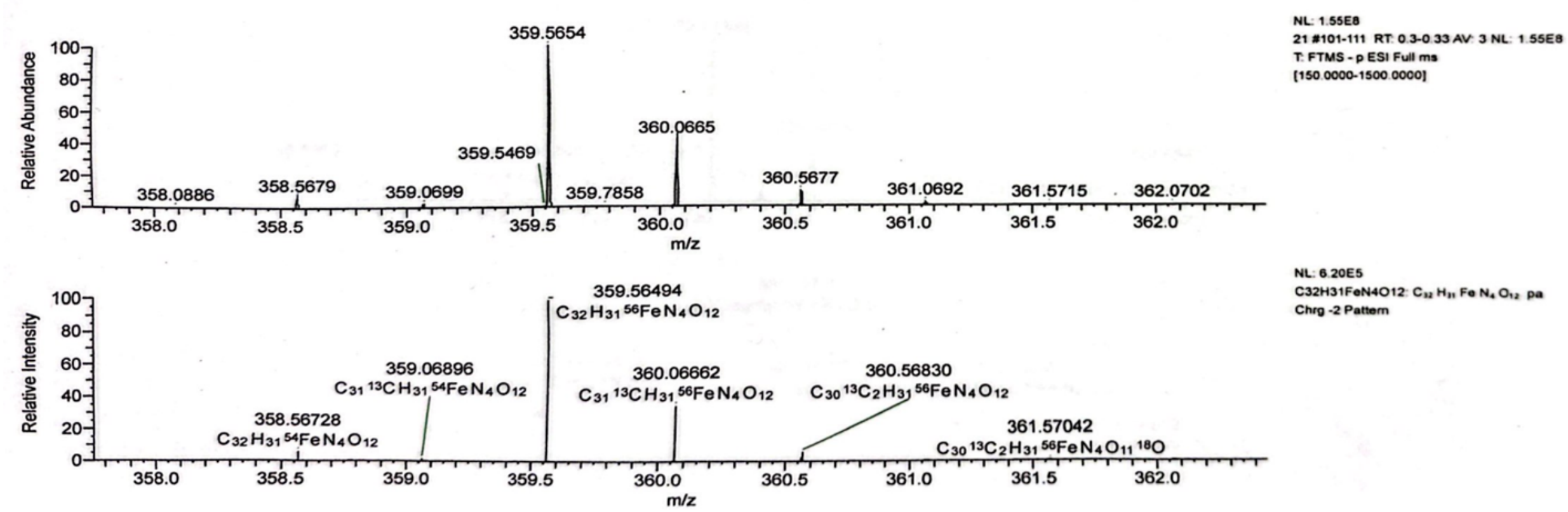

Figure S84. ESI (+) HR-MS spectrum of **[Fe(OK-COOH)]$^{3-}$**. ESI-HRMS calcd. for $C_{32}H_{30}FeN_4O_{12}$ $[M + H]^{2-}$: *m/z* 359.5638. Found 359.5654.

[Ga(OK-COOH)]$^{3-}$, was synthesized by the procedure outlined in section **1.8.2.** Retention time = 6.43 min (Method C). ESI-HR-MS calcd. for $C_{32}H_{30}GaN_4O_{12}$ [M + 2H]$^-$: *m/z* 733.1267 and [M + H]$^{2-}$: *m/z* 366.0592. Found 733.1291 and 366.0608, respectively.

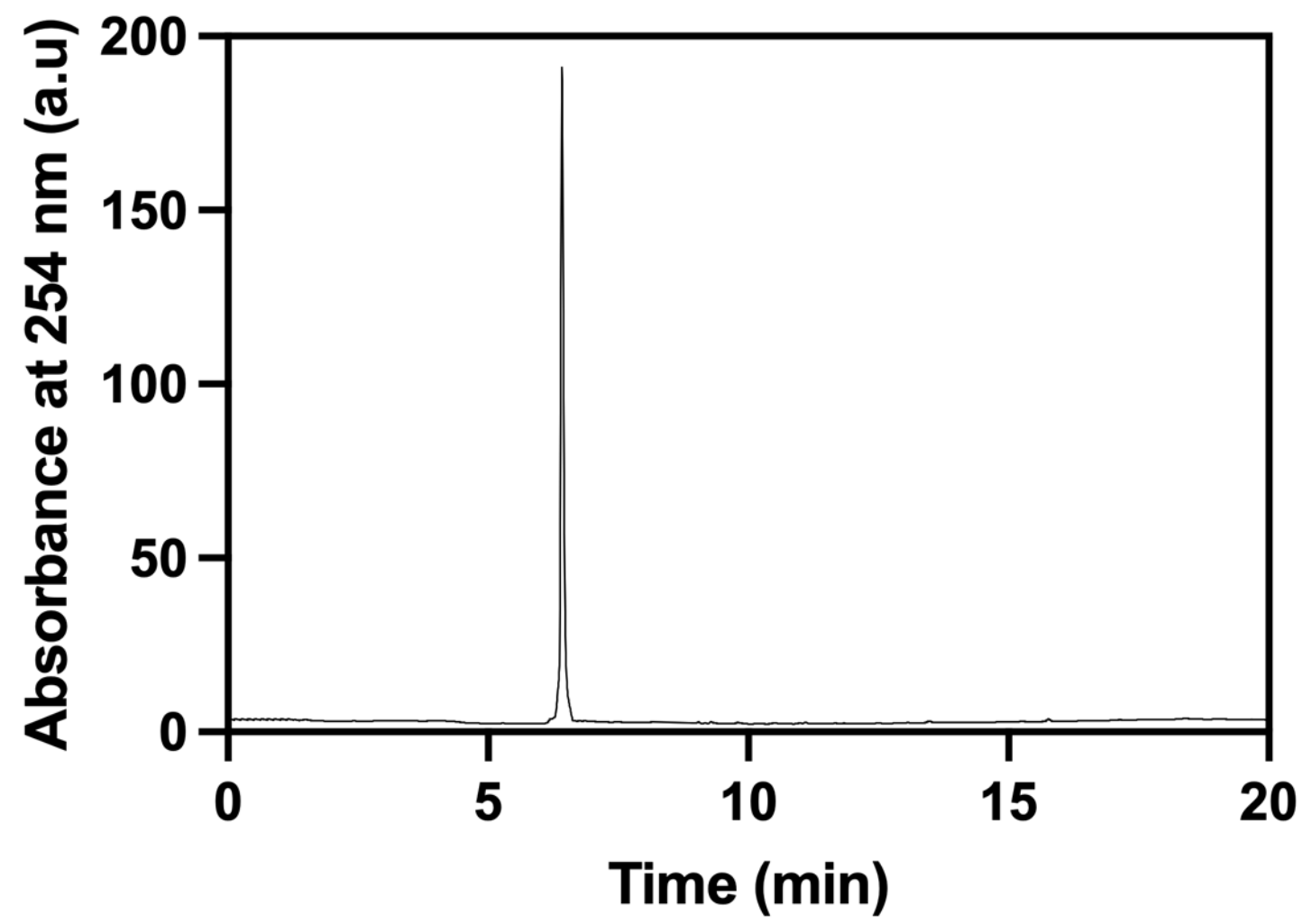

Figure S85. Normalized HPLC chromatogram of **[Ga(OK-COOH)]$^{3-}$**. Retention time: 6.43 min. (Method C).

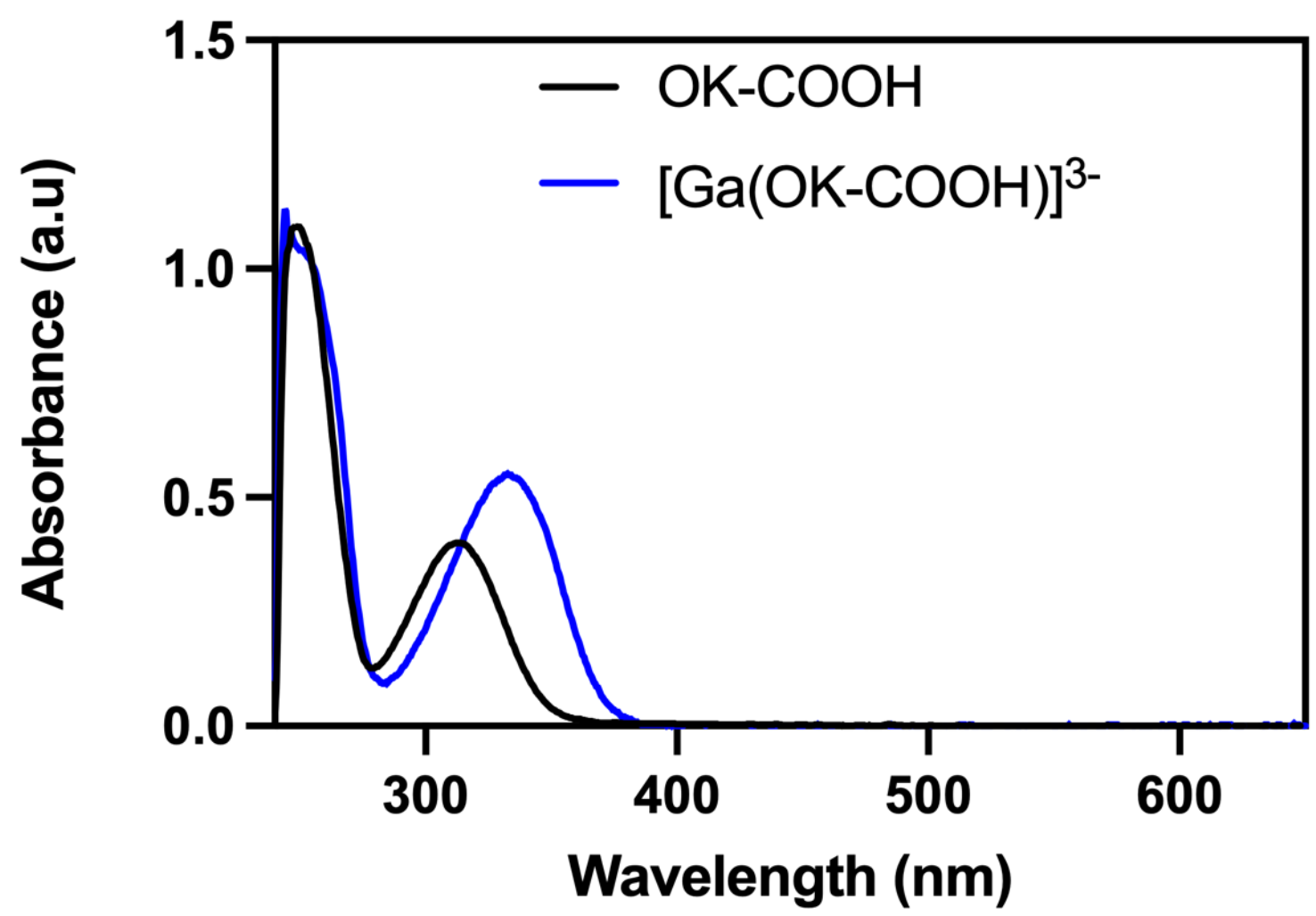

Figure S86. UV-vis spectrum of **[Ga(OK-COOH)]$^{3-}$**.

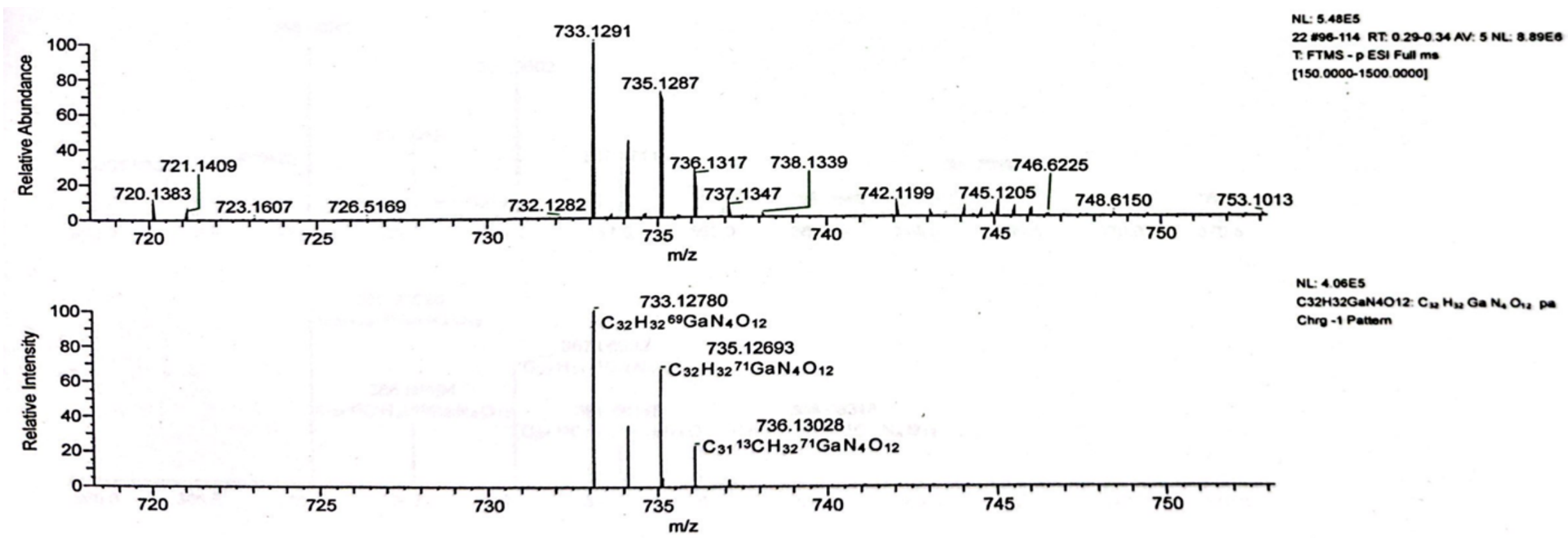

Figure S87. ESI (+) HR-MS spectrum of **[Ga(OK-COOH)]$^{3-}$**. ESI-HRMS calcd. for $C_{32}H_{30}GaN_4O_{12}$ $[M + 2H]^{-}$: *m/ z* 733.1267. Found 733.1291.

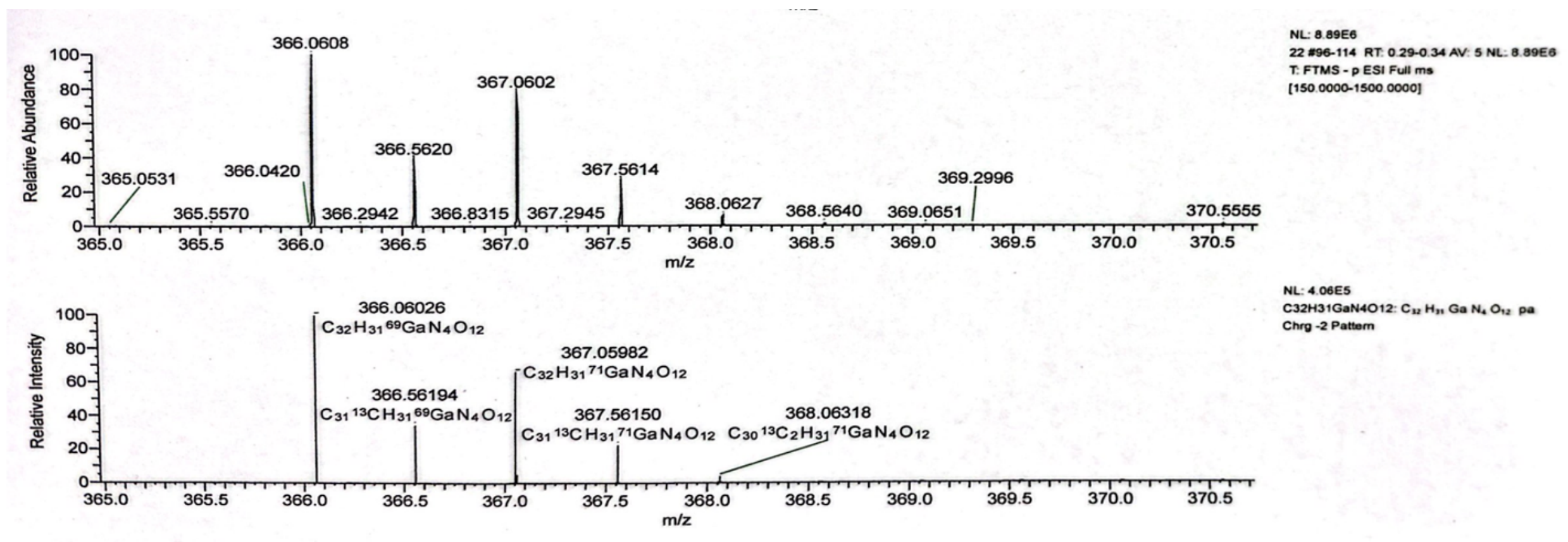

Figure S88. ESI (+) HR-MS spectrum of **[Ga(OK-COOH)]$^{3-}$**. ESI-HRMS calcd. for $C_{32}H_{30}GaN_4O_{12}$ $[M + H]^{2-}$: *m/ z* 366.0592. Found 366.0608.

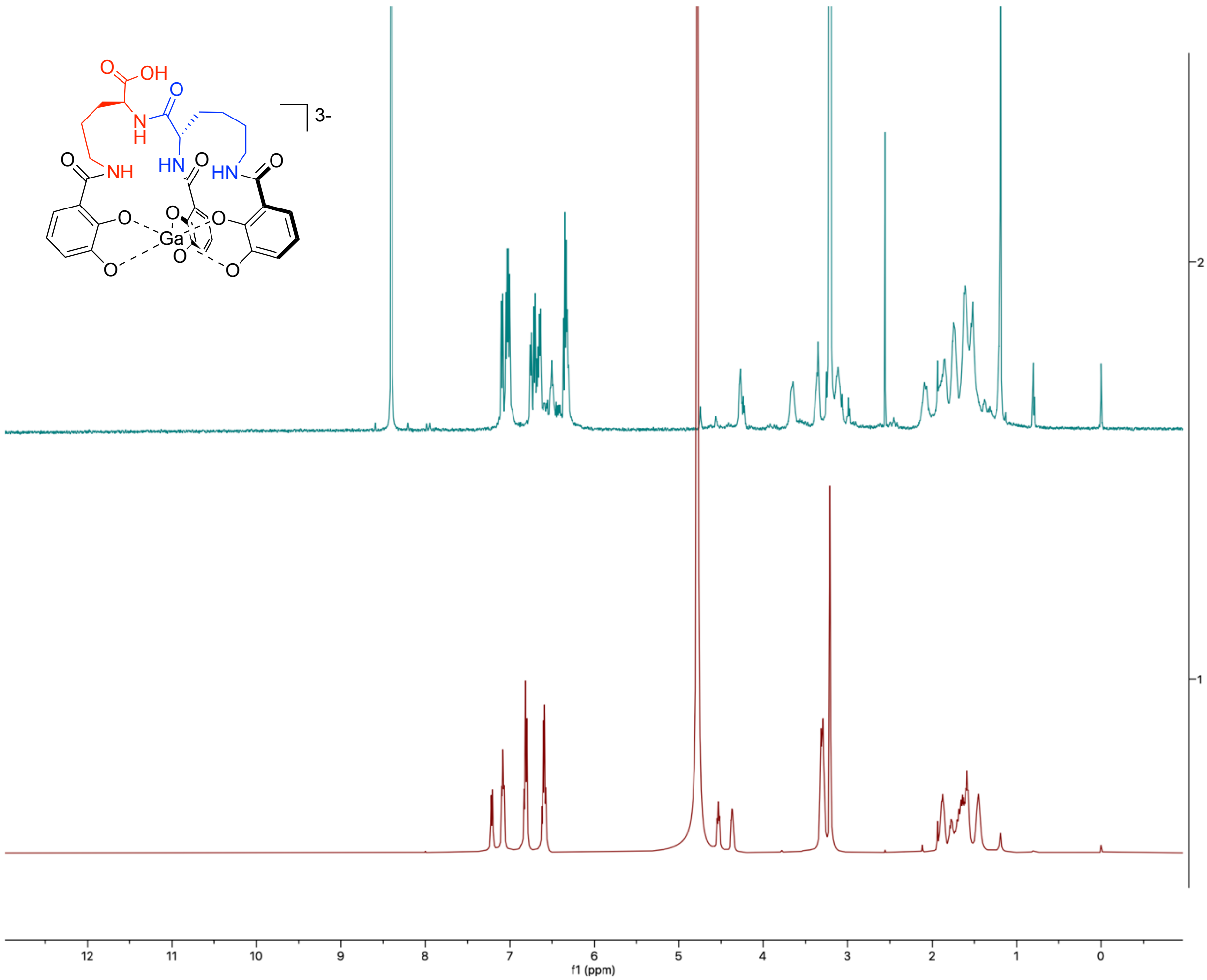

Figure S89. Full ^{1}H NMR (500 MHz, MeOD-d4, 298 K) of **OK-COOH** (maroon) and **[Ga(OK-COOH)]$^{3-}$** (teal).

1.9.5 KO-NH_2-Amp

[Fe(KO-NH_2-Amp)]$^{3-}$, was synthesized by the procedure outlined in section **1.8.1**. Retention time = 7.40 min (Method C). ESI-HRMS calcd. for $C_{60}H_{66}FeN_{12}O_{17}S$ $[M + Na]^{2-}$: *m/ z* 668.6813 and $[M + 2H]^{-}$: *m/ z* 1316.3890. Found 668.6813 and 1316.3867. LMCT d-d transition: λ_{max} = 550 nm.

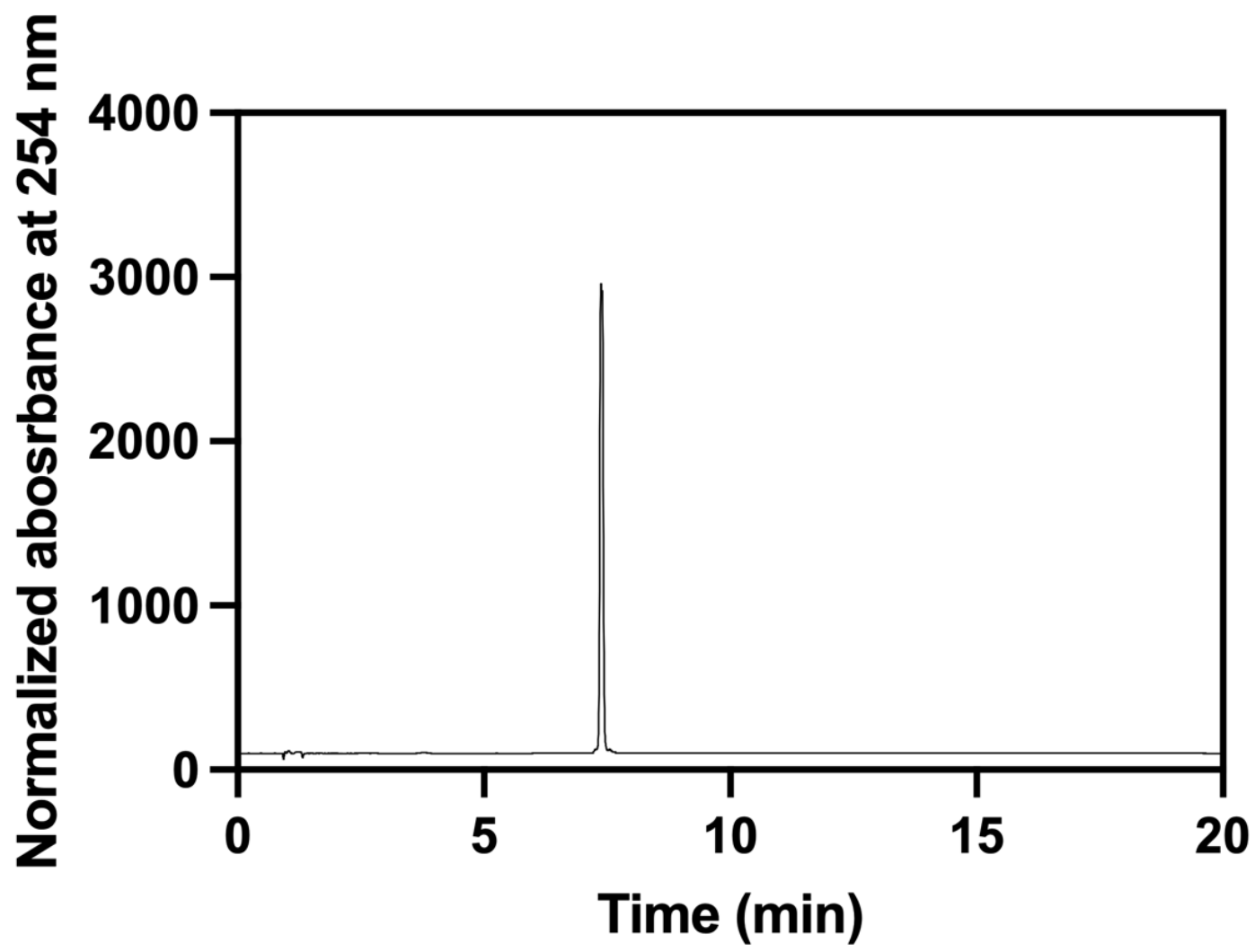

Figure S90. Normalized HPLC chromatogram of **[Fe(KO-NH_2-Amp)]$^{3-}$**. Retention time: 7.40 min (Method C).

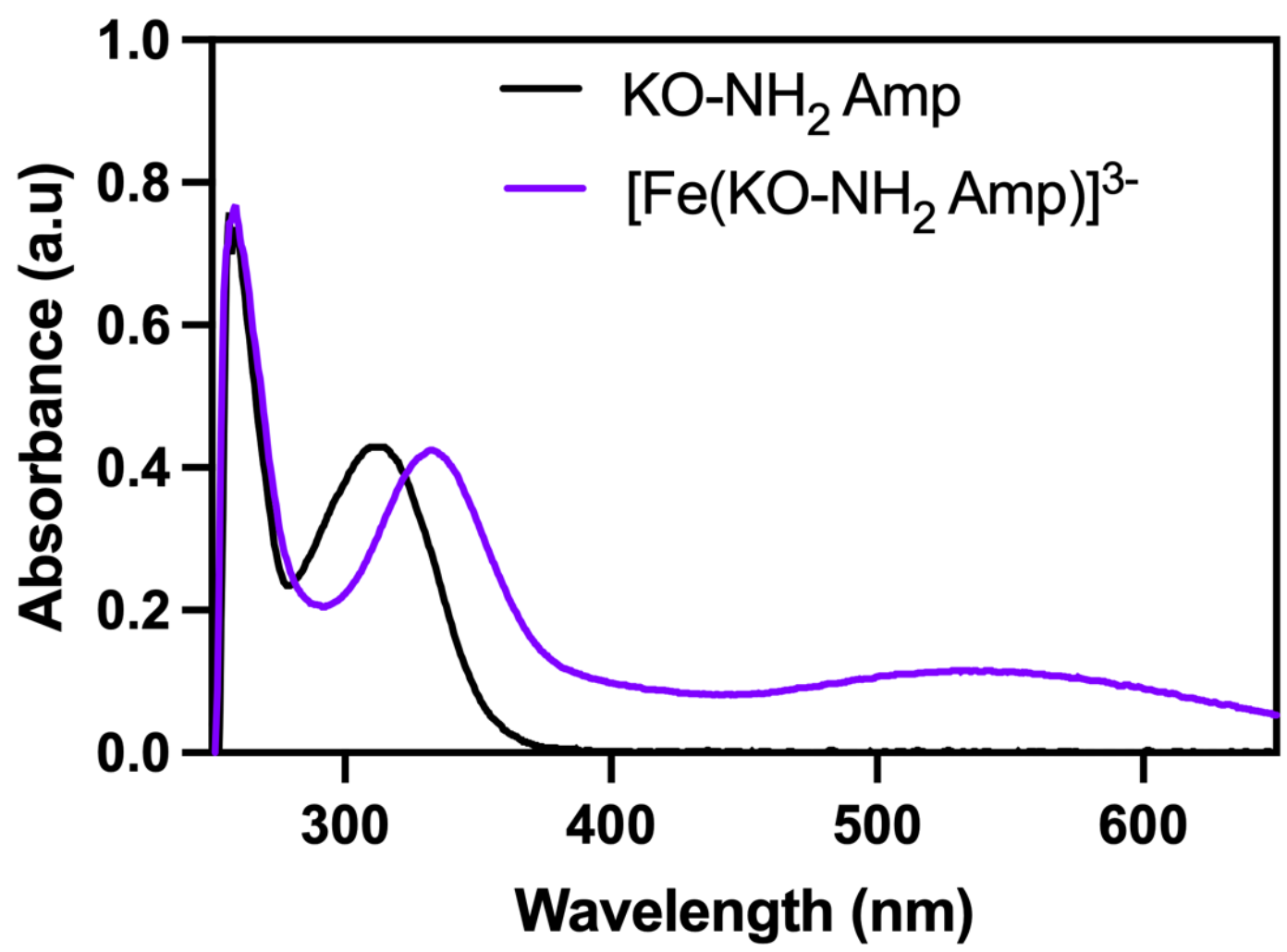

Figure S91. UV-vis spectrum of **[Fe(KO-NH_2-Amp)]$^{3-}$**. Observed LMTC, d-d transition λ = 550 nm.

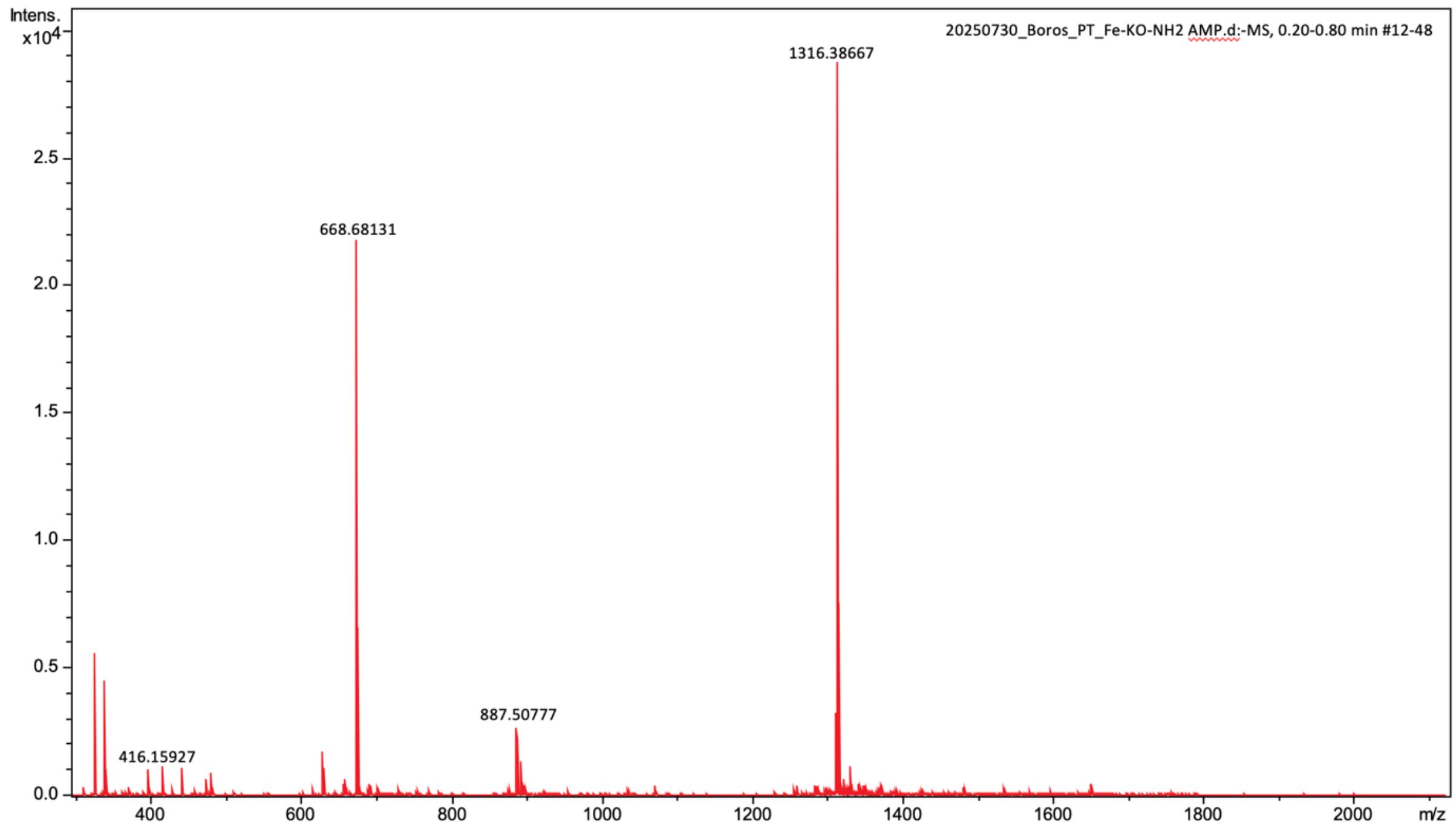

Figure S92. ESI (+) HRMS spectrum of **[Fe(KO-NH$_2$-Amp)]$^{3-}$**. ESI-HRMS calcd. for $C_{60}H_{66}FeN_{12}O_{17}S$ [M + Na]$^{2-}$: *m/ z* 668.6813 and [M + 2H]$^{-}$: *m/ z* 1316.3890. Found 668.6813 and 1316.3867.

[Ga(KO-NH$_2$-Amp)]$^{3-}$, was synthesized by the procedure outlined in section **1.8.2**. Retention time = 7.44 min (Method C). ESI-HRMS calcd. for $C_{60}H_{66}GaN_{12}O_{17}S$ [M + Na]$^{2-}$: *m/z* 675.1766 and [M + 2H]$^{-}$ *m/z* 1329.3790. Found 675.1736 and 1329.3786, respectively

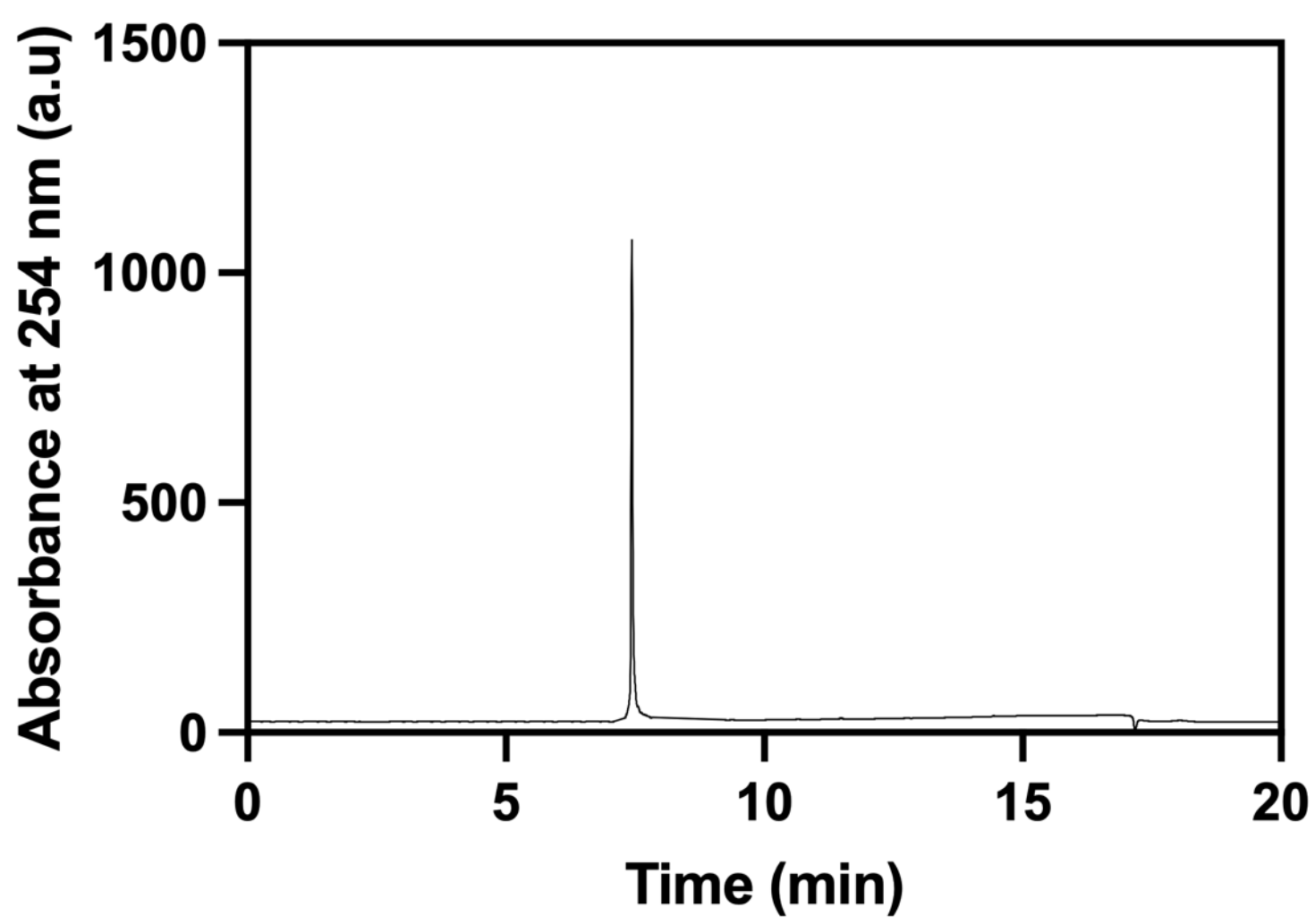

Figure S93. Normalized HPLC chromatogram of **[Ga(KO-NH$_2$-Amp)]$^{3-}$**. Retention time: 7.44 min (Method C).

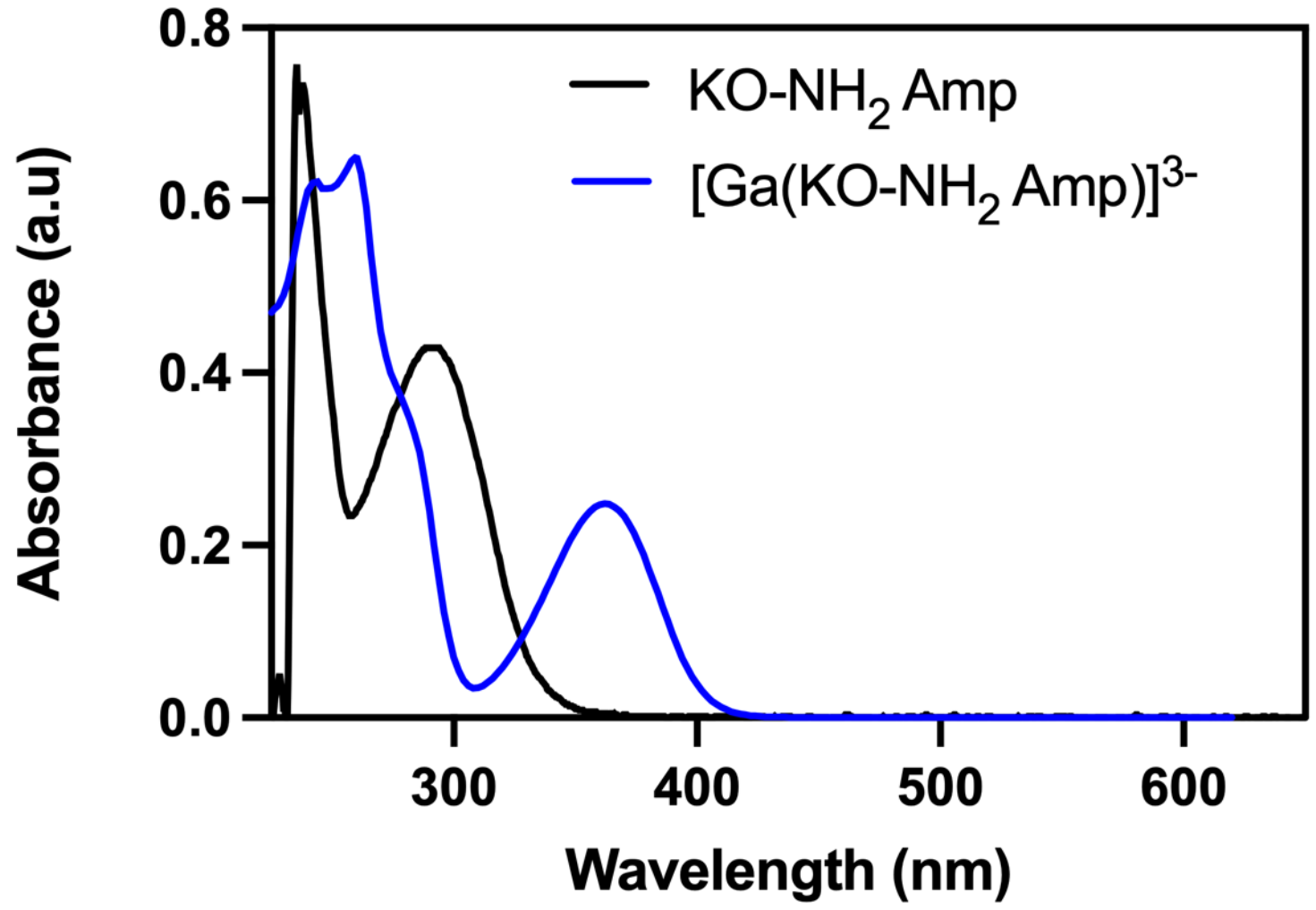

Figure S94. UV-vis spectrum of **[Ga(KO-NH$_2$-Amp)]**$^{3-}$.

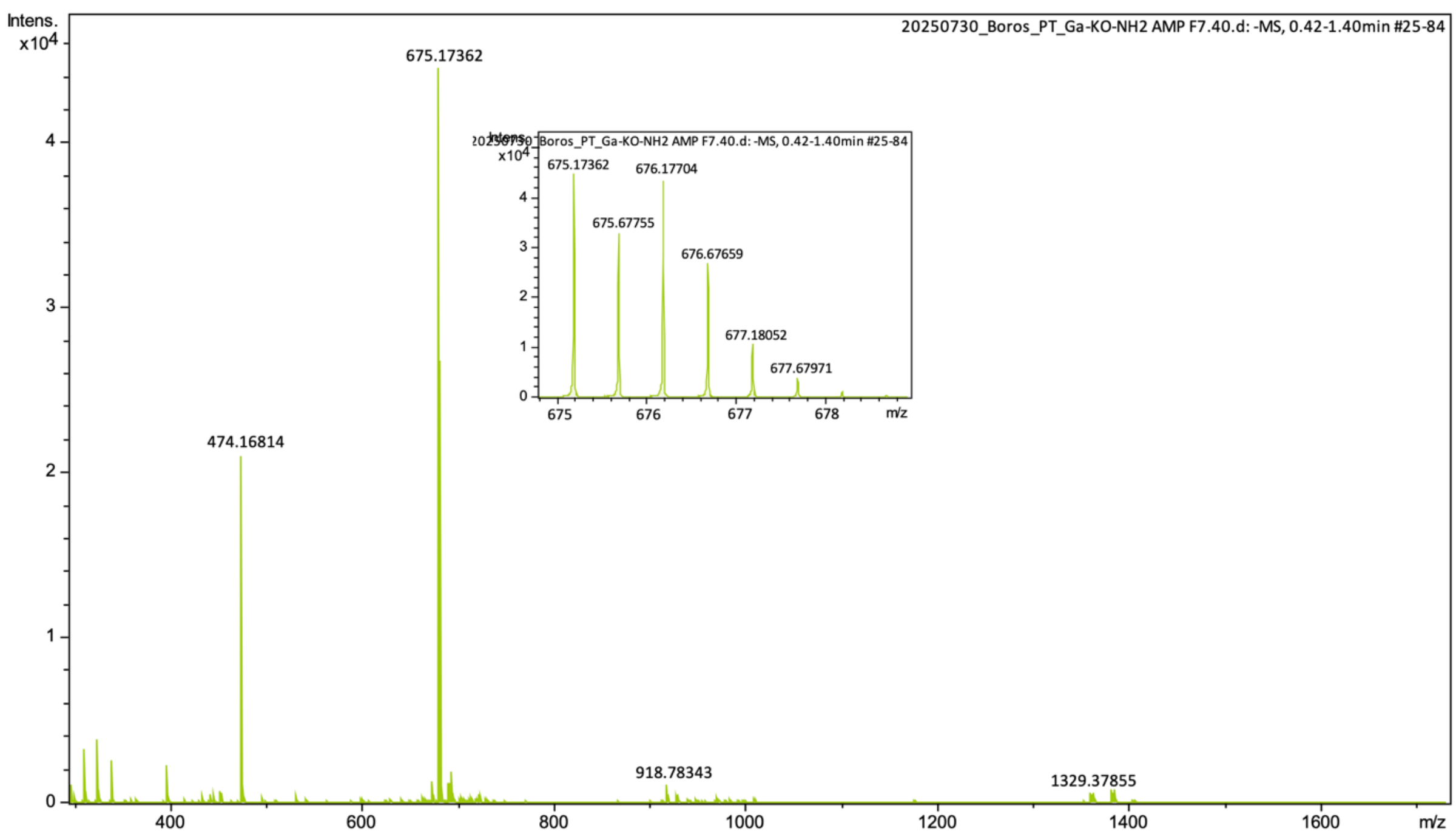

Figure S95. ESI (+) HRMS spectrum of [**Ga(KO-NH$_2$-Amp)**]$^{3-}$. ESI-HRMS calcd. for $C_{60}H_{66}GaN_{12}O_{17}S$ [M + Na]$^{2-}$: *m/z* 675.1766. Found 675.1736.

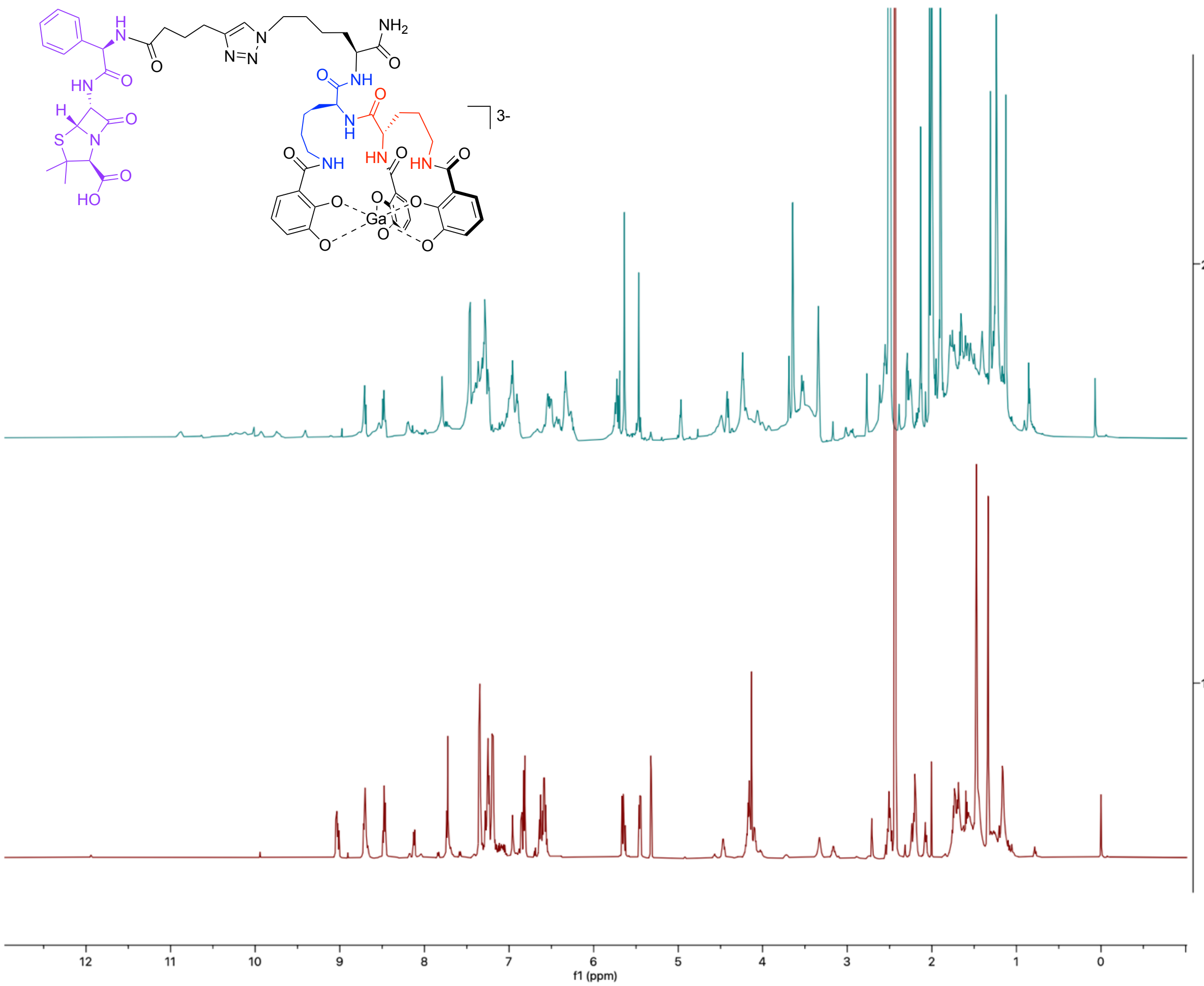

Figure S96. Full ^{1}H NMR (600 MHz, DMSO-*d6*, 298 K) of **KO-NH$_2$-Amp** (maroon) and **[Ga(KO-NH$_2$-Amp)]$^{3-}$** (teal).

1.9.6 KO-NH$_2$-azir-biotin

[Fe(KO-NH$_2$-azir-biotin)]$^{3-}$. **KO-NH$_2$-azir-biotin** was diluted in 10 mM NH_4COOH buffer, pH 7. Aqueous $FeCl_3$ from a stock solution (concentration determined by ICP-OES) was added to a 1:1.5 ratio of ligand:$FeCl_3$. pH was adjusted with NH_4OH 28-30% until pH = 9. After 16 h at room temperature, the complex was purified by HPLC Reverse Phase (Method C) to yield the pure complex.

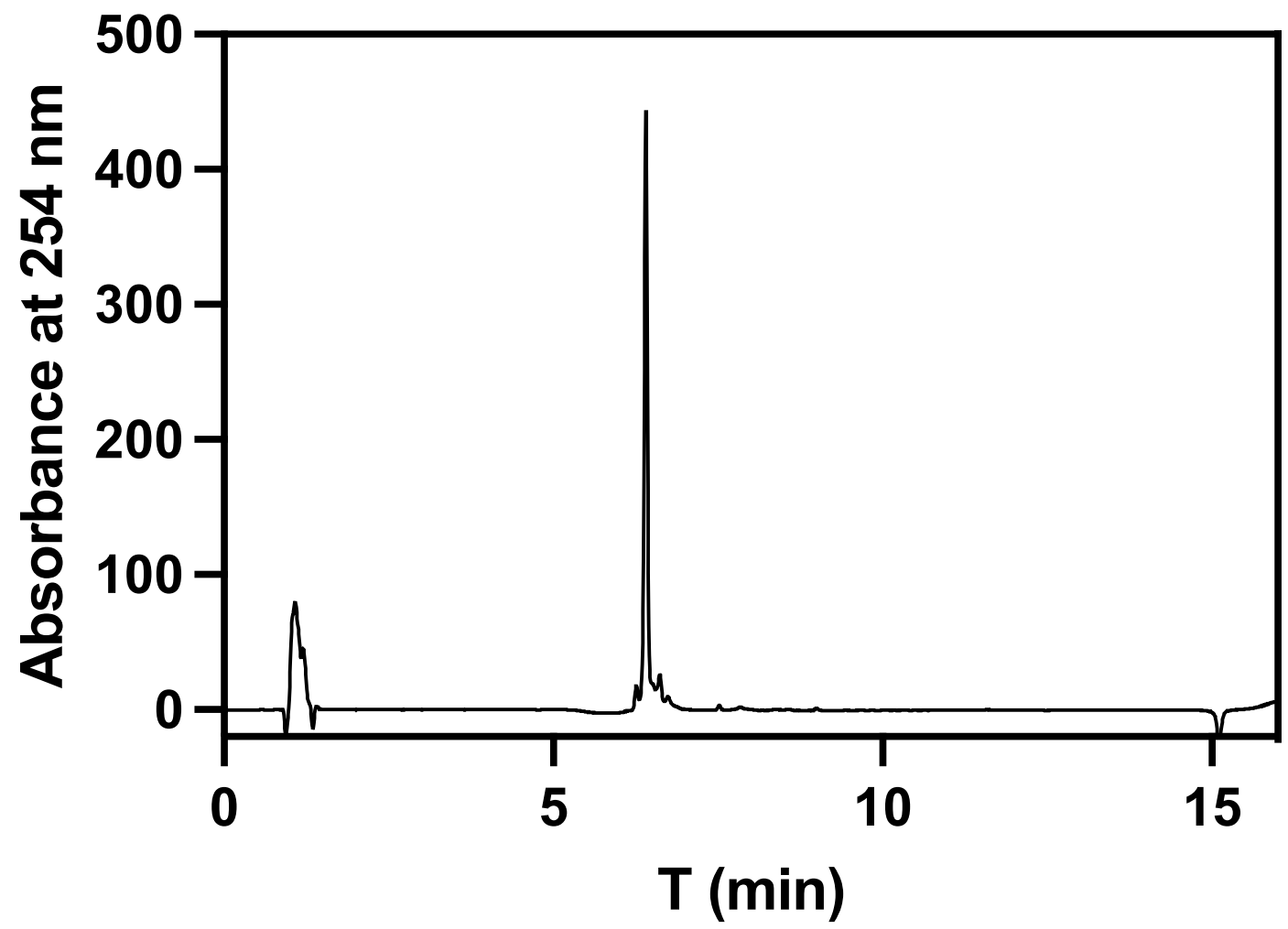

Figure S97. HPLC chromatogram of **[Fe(KO-NH$_2$-azir-biotin)]$^{3-}$**. Retention time: 6.63 min (Method C).

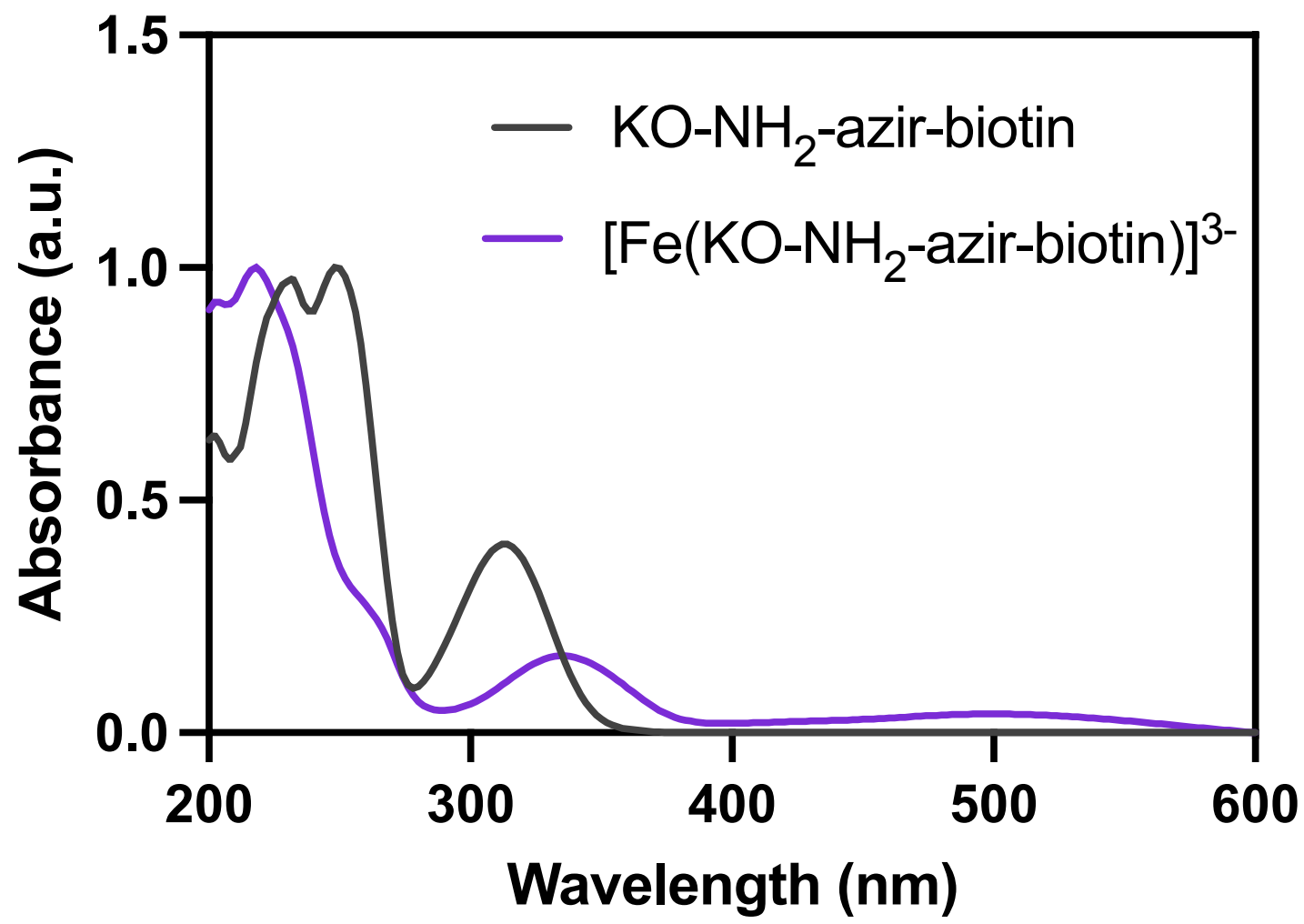

Figure S98. UV-vis spectrum of **[Fe(KO-NH$_2$-azir-biotin)]$^{3-}$**.

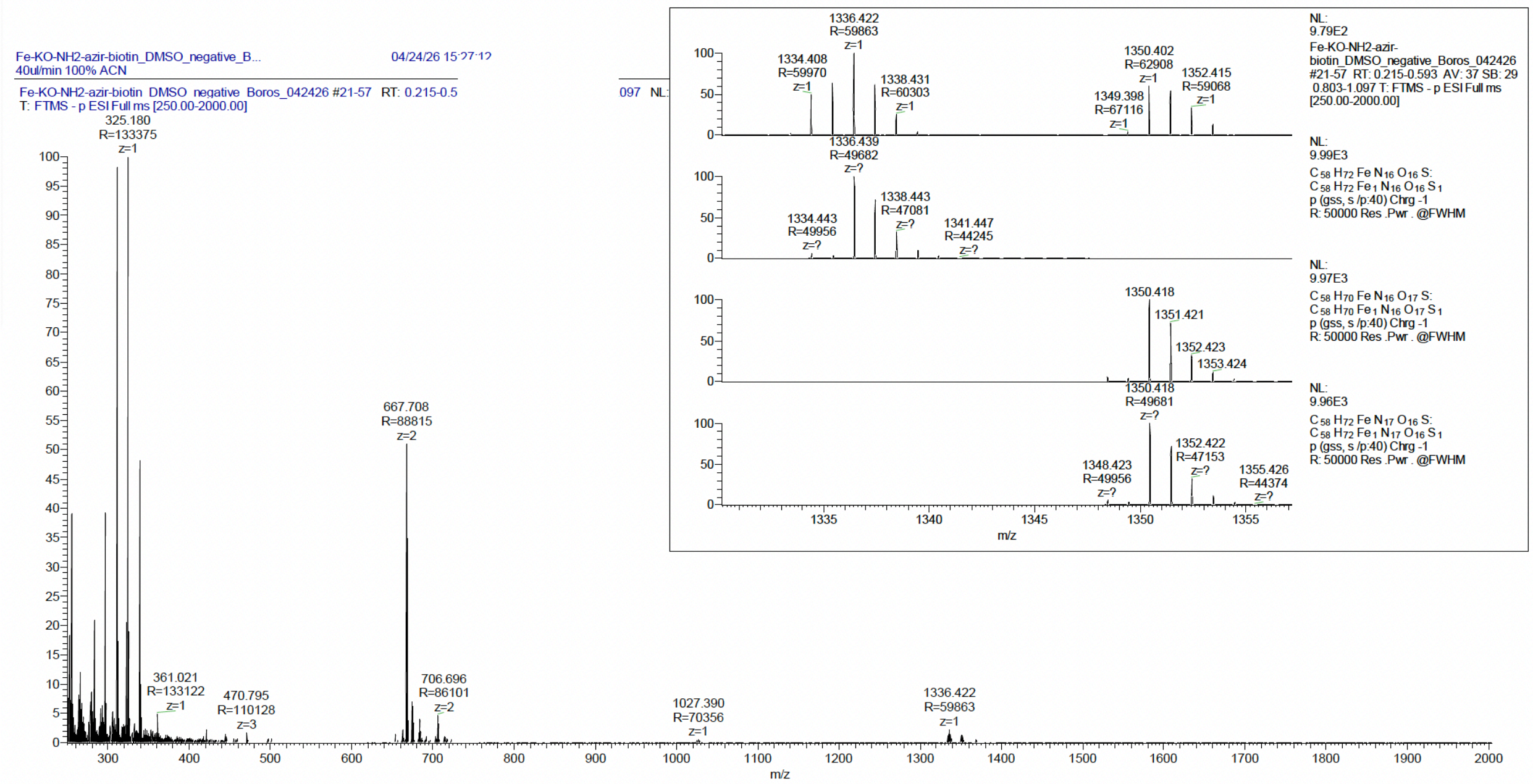

Figure S99. ESI (–) HRMS spectrum of **[Fe(KO-NH$_2$-azir-biotin)]$^{3-}$**. ESI-HRMS calcd. for $C_{58}H_{70}FeN_{16}O_{16}S$ [M+2H]$^-$ *m/z* 1336.439. Found 1336.422.

[Ga(KO-NH$_2$-azir-biotin)]$^{3-}$. KO-NH$_2$-azir-biotin was diluted in 10 mM NH_4COOH buffer, pH 7. Aqueous $Ga(NO_3)_3$ from a stock solution (concentration determined by ICP-OES) was added to a 1:1.5 ratio of ligand:$Ga(NO_3)_3$.. pH was adjusted with NH_4OH 28-30% until pH = 9. After 3 h at room temperature, the complex was purified by HPLC Reverse Phase (Method C) to yield the pure complex.

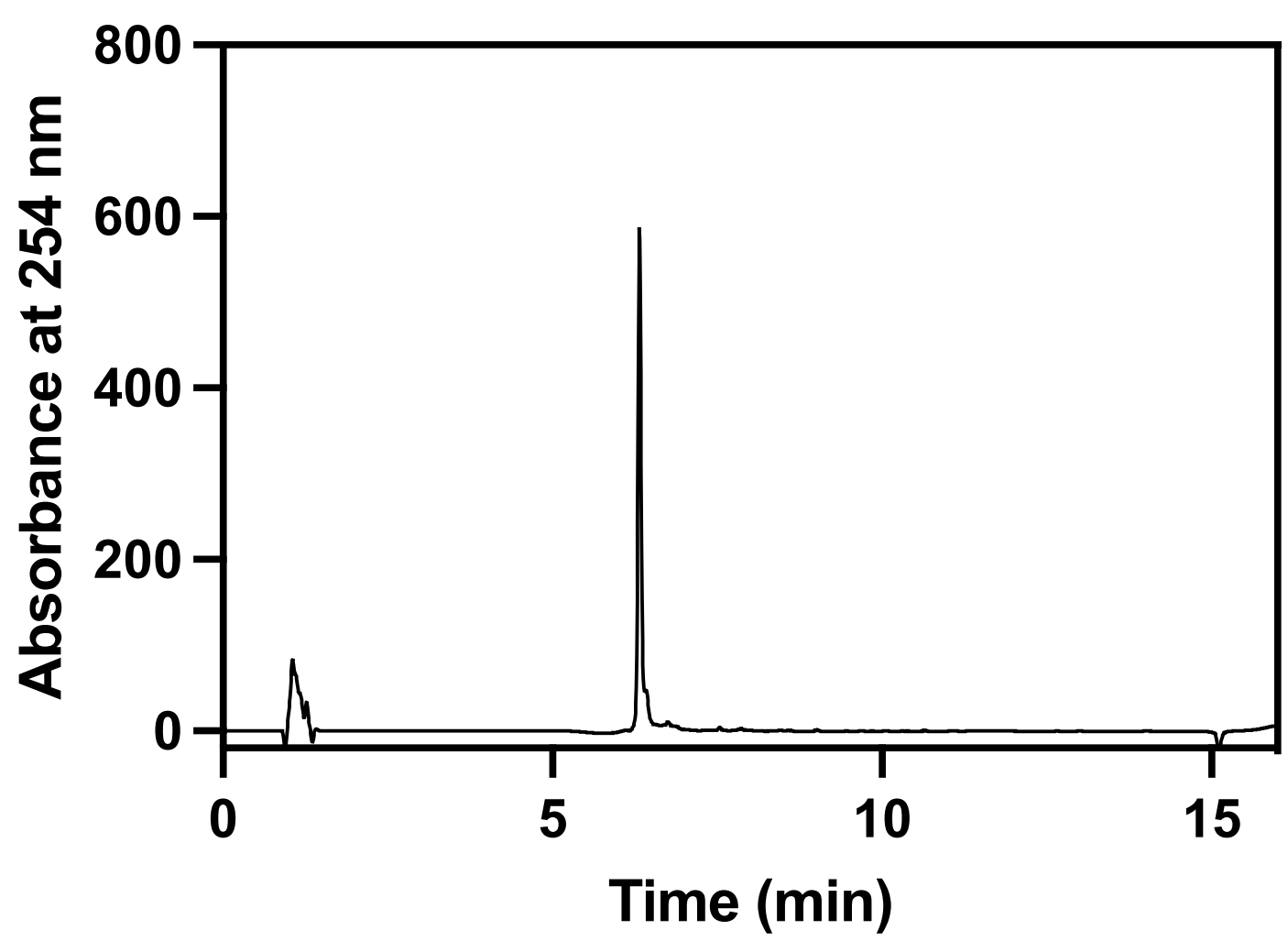

Figure S100. HPLC chromatogram of **[Ga(KO-NH$_2$-azir-biotin)]$^{3-}$**. Retention time: 6.51 min (Method C).

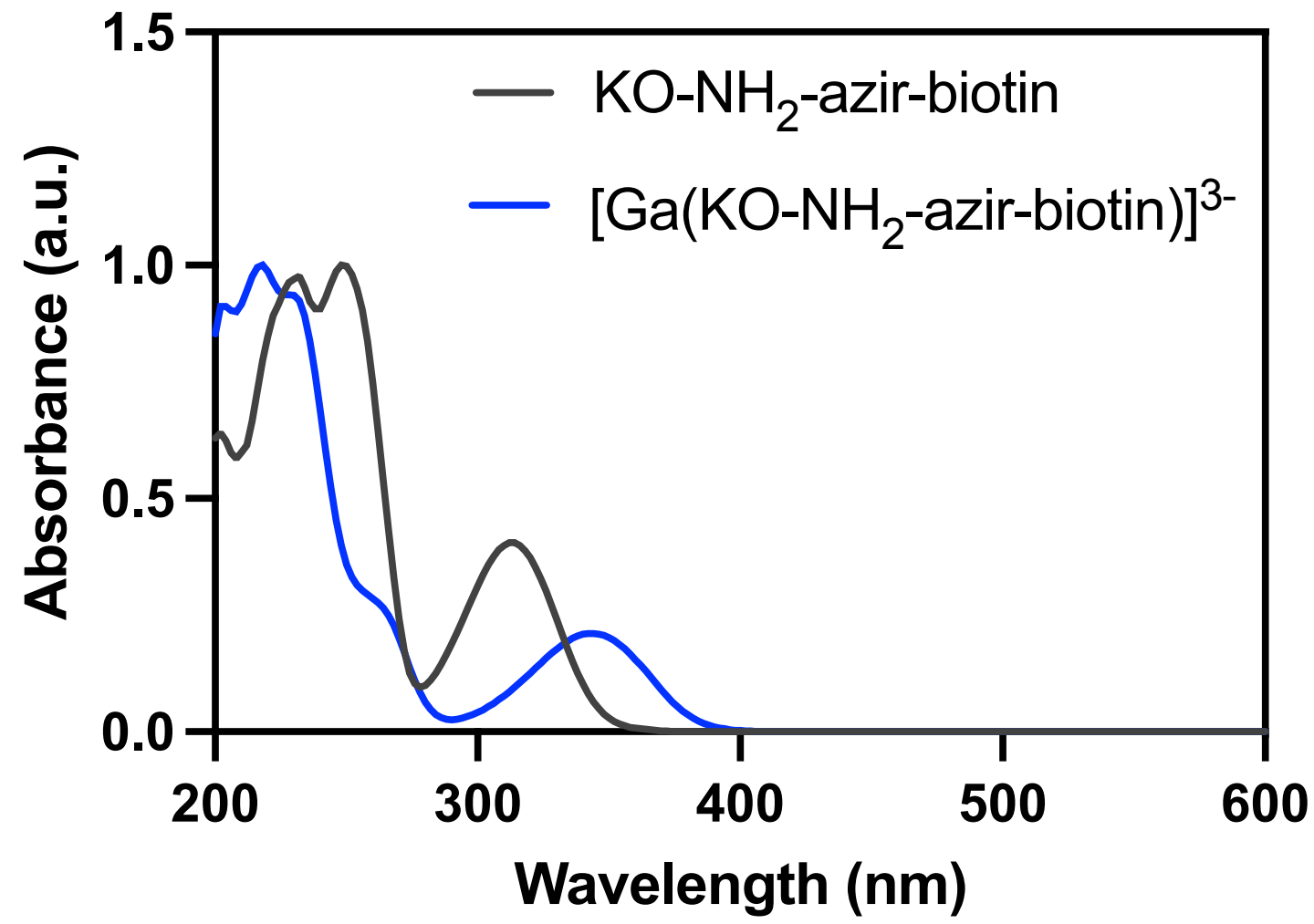

Figure S101. UV-vis spectrum of **[Ga(KO-NH$_2$-azir-biotin)]$^{3-}$**.

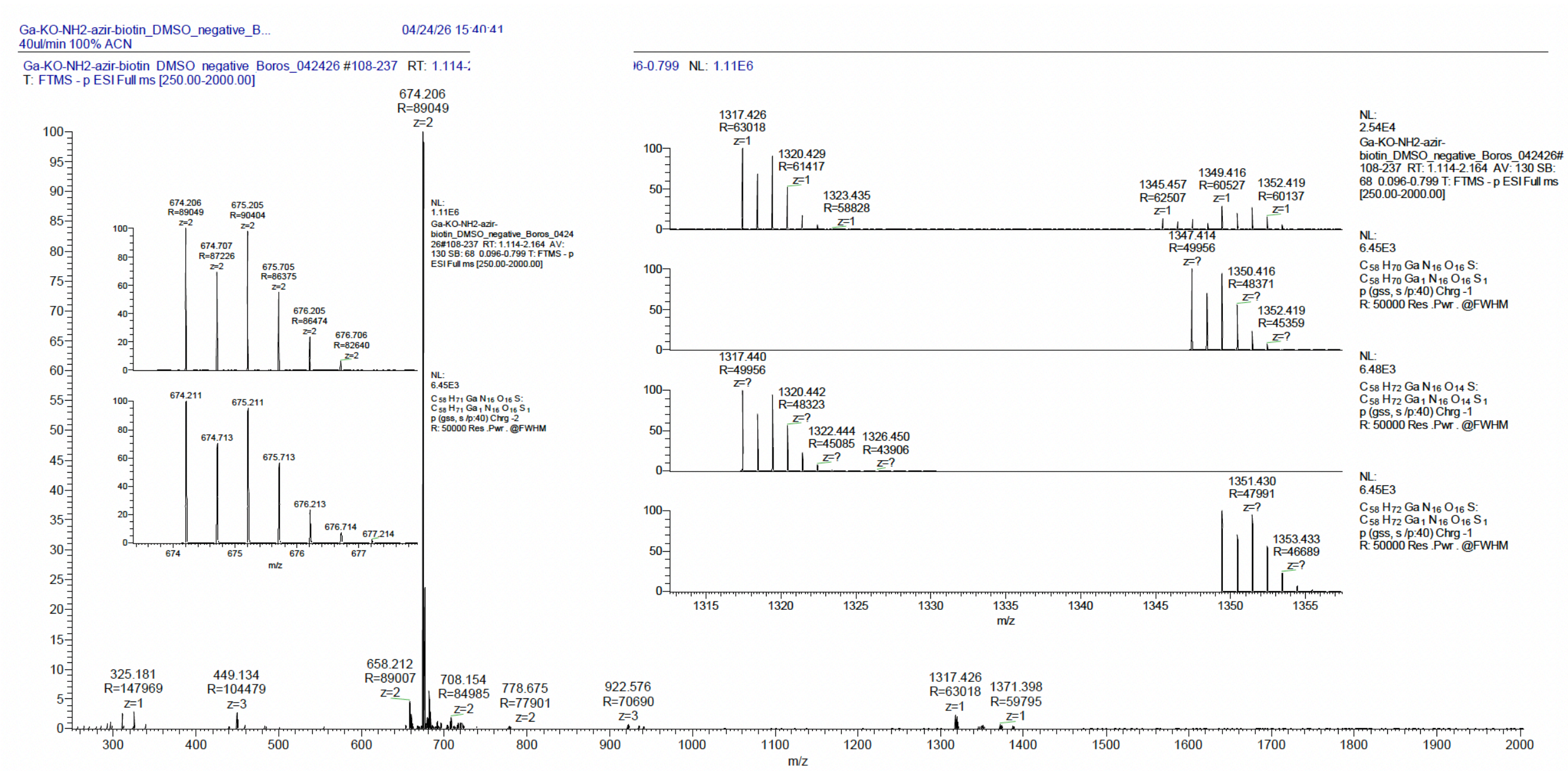

Figure S102. ESI (–) HRMS spectrum of **[Ga(KO-NH$_2$-azir-biotin)]$^{3-}$**. ESI-HRMS calcd. for $C_{58}H_{70}GaN_{16}O_{16}S$ $[M+H]^{2-}$ *m/z* 674.211. Found 674.206

1.10 Density Functional Theory (DFT) Calculations

Calculations were performed using ORCA 5.0.1 software package.[4] Computations were performed with the B3LYP functional[5] with the D3(BJ) dispersion[6] and the def2-TZVP[7] basis set. The CPCM polarizable continuum model was used with water as the solvent for all calculations, and structures for refinement were derived from crystal structures where available.[8] Optimized geometries were visualized using PyMOL, with hydrogen atoms omitted from the rendered structures for clarity.

RMSD analysis

Root mean square deviation (RMSD) values were calculated from optimized DFT structures via overlay of the coordination center using the program Mercury analogous to methods. Briefly, molecules are aligned via the maximum substructure and the distance between identical atoms are determined (ignoring hydrogen atoms). RMSD is then calculated as:

$$RMSD = \sqrt{\frac{\sum_{i=1}^{n}(d_{1,2}^{i})^2}{n}}$$

where di 1,2 represents the distance between equivalent atoms, i, for molecules 1 and 2. The resulting value represents the average deviation between identical atomic positions of two molecular substructures. A higher RMSD indicates more disparate structures. The protochelin mimics' structures discussed herein were compared against parent protochelin structure.

Table S1. RMSD relative to **$[Fe(PC)]^{3-}$** structure.

Structure	RMSD Relative to $[Fe(PC)]^{3-}$
**$[Fe(KK\text{-}COOH)]^{3-}$**	4.675
**$[Fe(KK\text{-}NH_2)]^{3-}$**	4.667
**$[Fe(OK\text{-}COOH)]^{3-}$**	4.334
**$[Fe(OK\text{-}NH_2)]^{3-}$**	4.407
**$[Fe(KO\text{-}COOH)]^{3-}$**	4.537
**$[Fe(KO\text{-}NH_2)]^{3-}$**	4.813
**$[Fe(OO\text{-}COOH)]^{3-}$**	4.449
**$[Fe(OO\text{-}NH_2)]^{3-}$**	4.437

Geometry Optimized Structures of [M(CAT)$_3$]$^{n-6}$ complexes

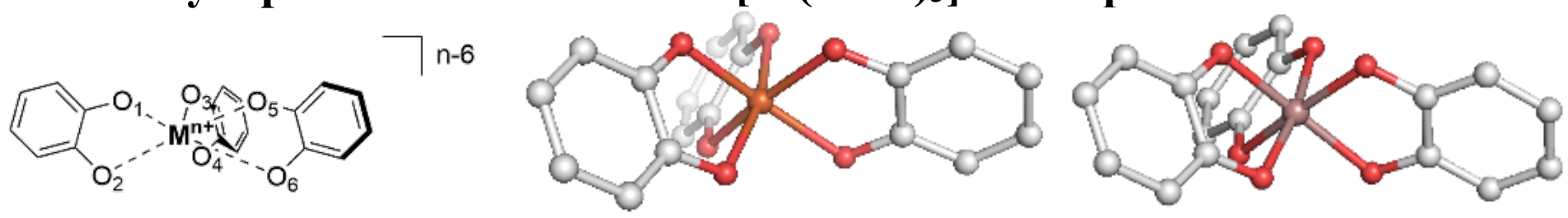

Figure S103. Chemical structure of **[M(CAT)$_3$]$^{n-6}$** with coordinating atoms annotated (left), with DFT-optimized configurational molecular model **[Fe(CAT)$_3$]$^{3-}$** and **[Ga(CAT)$_3$]$^{3-}$** (left to right).

Table S2. Optimized metal-center based bonding of **[M(CAT)$_3$]$^{n-6}$**.

**Bond**	**[Fe(CAT)$_3$]$^{3-}$**	**[Ga(CAT)$_3$]$^{3-}$**
Bond lengths [Å]		
M-O$_1$	2.036	1.988
M-O$_2$	2.043	1.993
M-O$_3$	2.043	1.995
M-O$_4$	2.034	1.991
M-O$_5$	2.048	1.991
M-O$_6$	2.037	1.994
Torsional angles [°]		
O$_1$-O$_2$	79.784	82.663
O$_3$-O$_4$	79.930	82.407
O$_5$-O$_6$	79.765	82.568
O$_1$-O$_6$	166.813	170.371
O$_2$-O$_3$	166.826	170.423
O$_4$-O$_5$	165.803	171.025

Geometry Optimized Structures of $[M(Ent)]^{n-6}$ Complexes

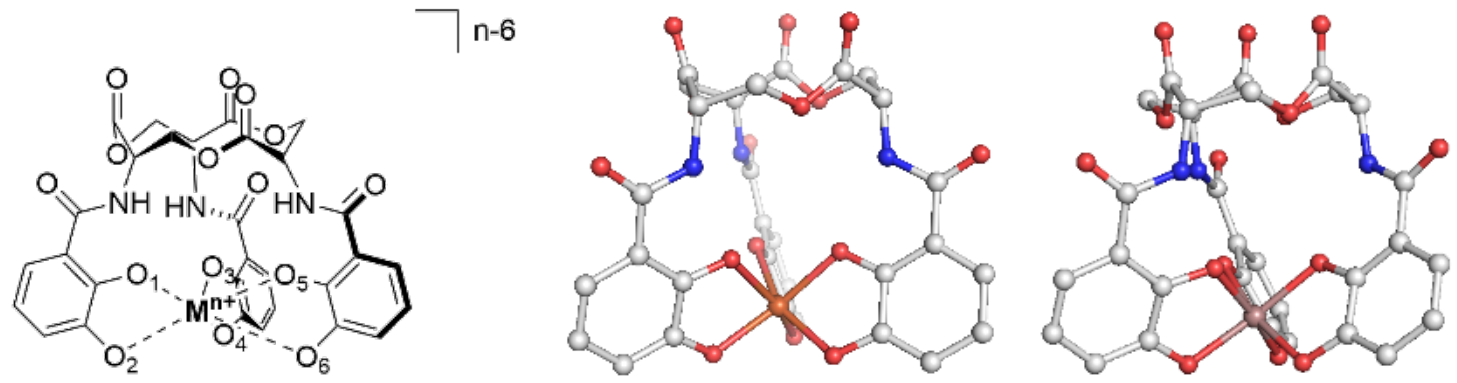

Figure S104. Chemical structure of **$[M(Ent)]^{n-6}$** with coordinating atoms annotated (left), with DFT-optimized configurational molecular model **$[Fe(Ent)]^{3-}$** and **$[Ga(Ent)]^{3-}$** (left to right).

Table S3. Optimized metal-center based bonding of **$[M(Ent)]^{n-6}$**.

Bond	$[Fe(Ent)]^{3-}$	$[Ga(Ent)]^{3-}$
Bond lengths [Å]		
M-O_1	2.095	2.018
M-O_2	1.996	1.979
M-O_3	2.090	2.017
M-O_4	2.003	1.981
M-O_5	2.072	2.020
M-O_6	2.019	1.978
Torsional angles [°]		
O_1-O_2	77.550	81.657
O_3-O_4	77.697	81.638
O_5-O_6	77.521	81.585
O_1-O_4	155.381	166.830
O_2-O_5	156.594	166.501
O_3-O_6	154.231	167.030

Geometry Optimized Structures of $[M(PC)]^{n-6}$ Complexes

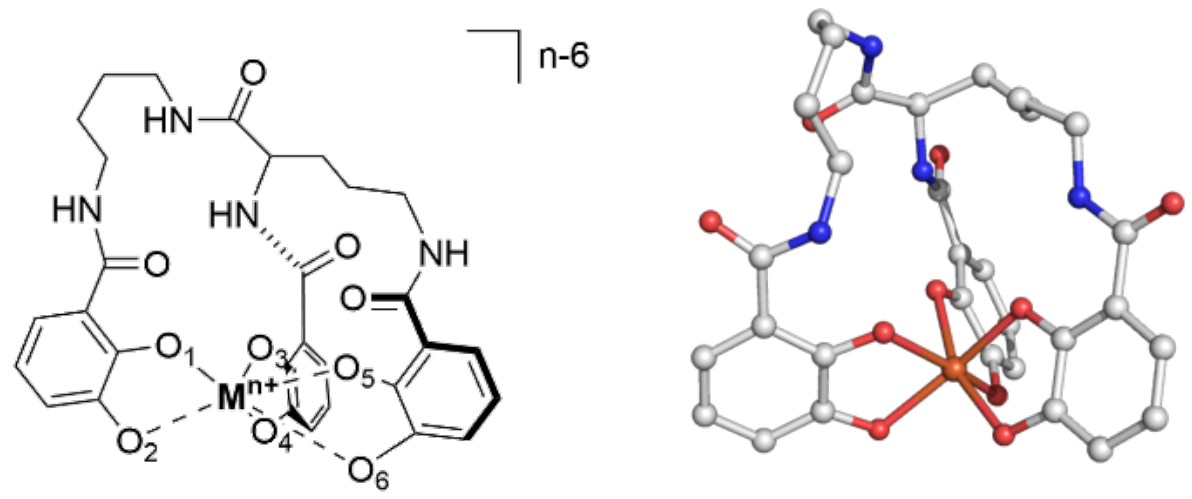

Figure S105. Chemical structure of **$[M(PC)]^{n-6}$** with coordinating atoms annotated (left), with DFT-optimized configurational molecular model **$[Fe(PC)]^{3-}$**.

Table S4. Optimized metal-center based bonding of **$[M(PC)]^{n-6}$**.

**Bond**	**$[Fe(PC)]^{3-}$**
Bond lengths [Å]	
M-O_1	2.049
M-O_2	2.041
M-O_3	2.108
M-O_4	2.002
M-O_5	2.064
M-O_6	2.015
Torsional angles [°]	
O_1-O_2	78.4
O_3-O_4	78.7
O_5-O_6	79.0
O_1-O_4	160.8
O_2-O_5	163.0
O_3-O_6	160.0

Geometry Optimized Structures of [M(KK-COOH)]$^{n-6}$ Complexes

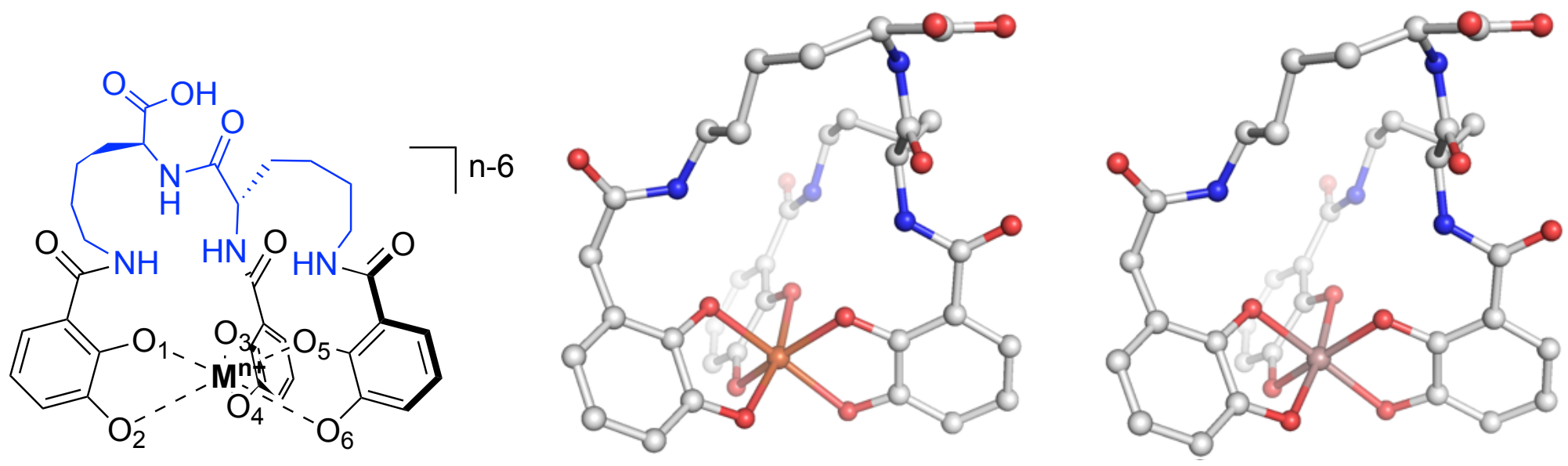

Figure S106. Chemical structure of **[M(KK-COOH)]$^{n-6}$** with coordinating atoms annotated (left), with DFT-optimized configurational molecular model **[Fe(KK-COOH)]$^{3-}$** and **[Ga(KK-COOH)]$^{3-}$** (left to right).

Table S5. Optimized metal-center based bonding of **[M(KK-COOH)]$^{n-6}$**.

**Bond**	**[Fe(KK-COOH)]$^{3-}$**	**[Ga(KK-COOH)]$^{3-}$**
Bond lengths [Å]		
M-O$_1$	2.074	2.018
M-O$_2$	2.013	1.981
M-O$_3$	2.075	2.019
M-O$_4$	2.020	1.987
M-O$_5$	2.047	2.005
M-O$_6$	2.046	2.000
Torsional angles [°]		
O$_1$-O$_2$	79.788	82.588
O$_3$-O$_4$	79.158	81.907
O$_5$-O$_6$	78.804	81.510
O$_1$-O$_6$	167.515	170.612
O$_2$-O$_3$	166.691	171.041
O$_4$-O$_5$	159.880	165.260

Geometry Optimized Structures of [M(KK-NH$_2$)]$^{n-6}$ Complexes

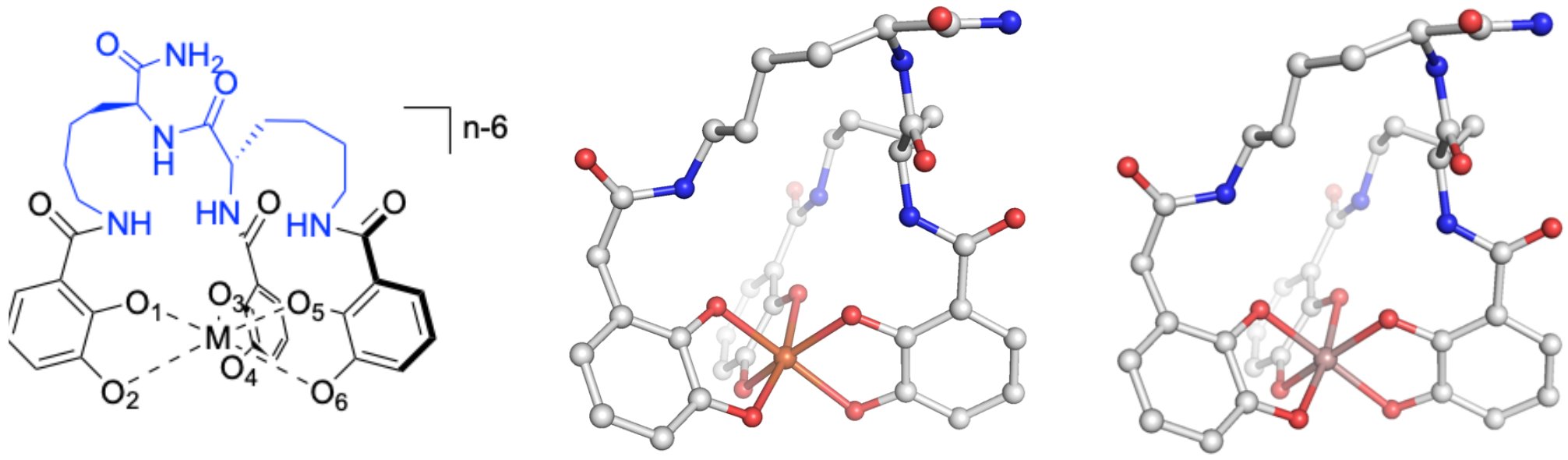

Figure S107. Chemical structure of **[M(KK-NH$_2$)]$^{n-6}$** with coordinating atoms annotated (left), with DFT-optimized configurational molecular model **[Fe(KK-NH$_2$)]$^{3-}$** and **[Ga(KK-NH$_2$)]$^{3-}$** (left to right).

Table S6. Optimized metal-center based bonding of **[M(KK-NH$_2$)]$^{n-6}$.**

**Bond**	**[Fe(KK-NH$_2$)]$^{3-}$**	**[Ga(KK-NH$_2$)]$^{3-}$**
Bond lengths [Å]		
M-O$_1$	2.073	2.016
M-O$_2$	2.012	1.981
M-O$_3$	2.079	2.020
M-O$_4$	2.019	1.987
M-O$_5$	2.047	2.004
M-O$_6$	2.047	2.002
Torsional angles [°]		
O$_1$-O$_2$	79.861	82.642
O$_3$-O$_4$	79.143	81.882
O$_5$-O$_6$	78.853	81.480
O$_1$-O$_6$	167.760	170.617
O$_2$-O$_3$	166.780	171.131
O$_4$-O$_5$	159.600	165.017

Geometry Optimized Structures of [M(KO-NH$_2$)]$^{n-6}$ Complexes

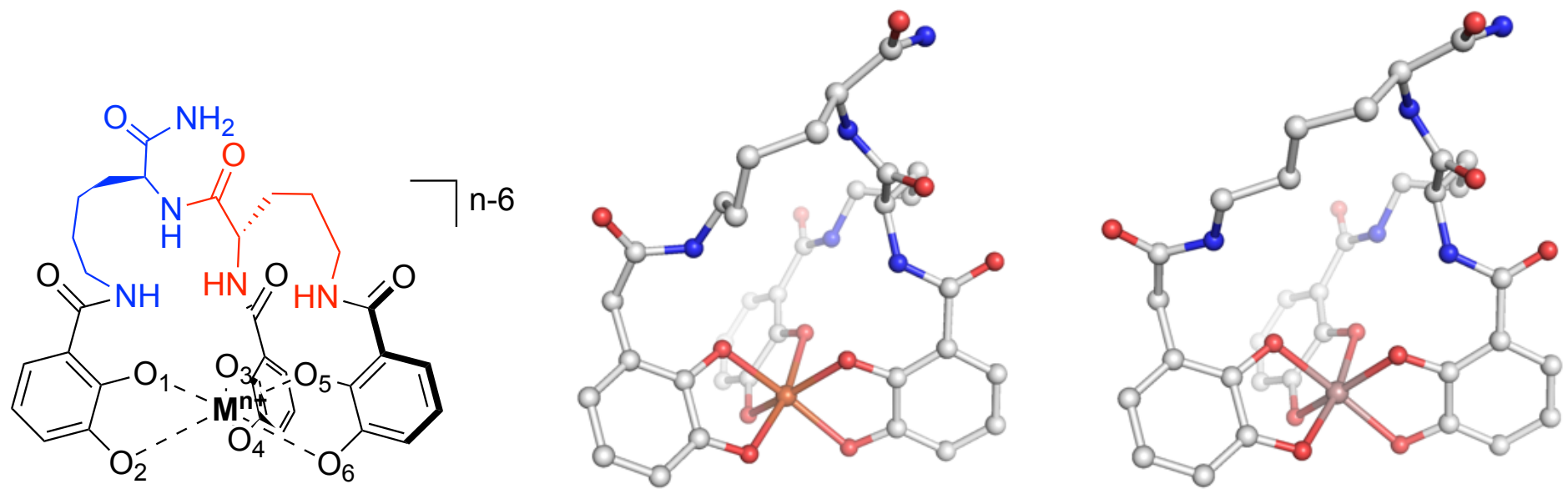

Figure S108. Chemical structure of **[M(KO-NH$_2$)]$^{n-6}$** with coordinating atoms annotated (left), with DFT-optimized configurational molecular model **[Fe(KO-NH$_2$)]$^{3-}$** and **[Ga(KO-NH$_2$)]$^{3-}$** (left to right).

Table S7. Optimized metal-center based bonding of **[M(KO-NH$_2$)]$^{n-6}$**.

**Bond**	**[Fe(KO-NH$_2$)]$^{3-}$**	**[Ga(KO-NH$_2$)]$^{3-}$**
Bond lengths [Å]		
M-O$_1$	2.071	2.021
M-O$_2$	2.011	1.983
M-O$_3$	2.072	2.026
M-O$_4$	2.030	1.998
M-O$_5$	2.054	2.011
M-O$_6$	2.044	1.999
Torsional angles [°]		
O$_1$-O$_2$	79.697	81.952
O$_3$-O$_4$	78.696	80.981
O$_5$-O$_6$	78.923	81.281
O$_1$-O$_6$	166.210	169.825
O$_2$-O$_3$	167.027	169.296
O$_4$-O$_5$	157.309	162.154

Geometry Optimized Structures of [M(KO-COOH)]$^{n-6}$ Complexes

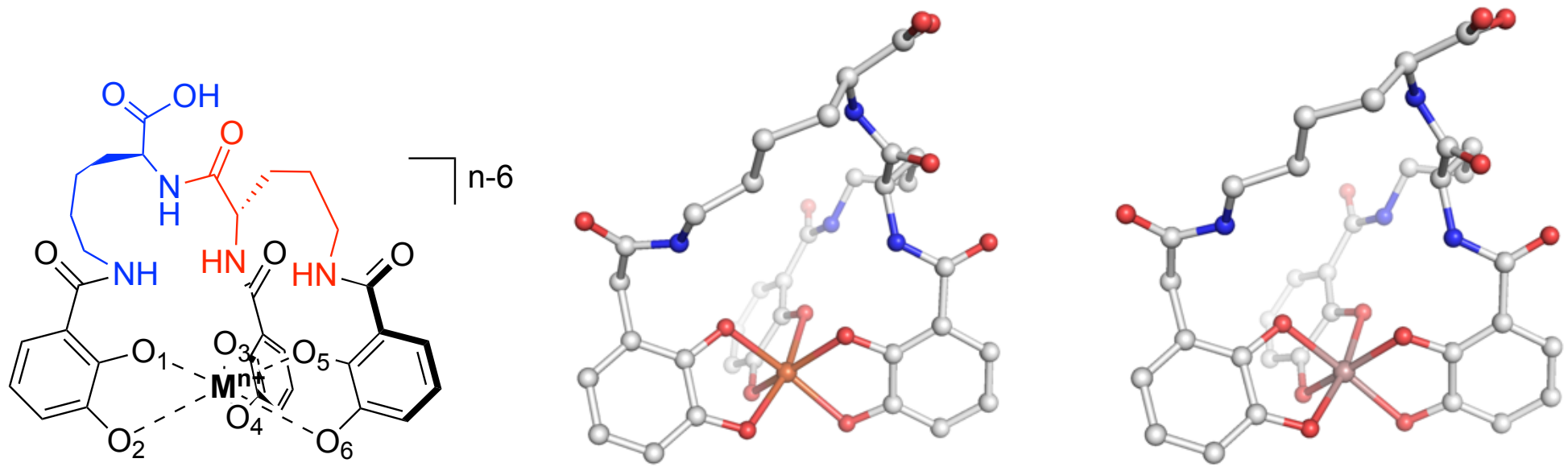

Figure S109. Chemical structure of **[M(KO-COOH)]$^{n-6}$** with coordinating atoms annotated (left), with DFT-optimized configurational molecular model **[Fe(KO-COOH)]$^{3-}$** and **[Ga(KO-COOH)]$^{3-}$** (left to right).

Table S8. Optimized metal-center based bonding of **[M(KO-COOH)]$^{n-6}$**.

**Bond**	**[Fe(KO-COOH)]$^{3-}$**	**[Ga(KO-COOH)]$^{3-}$**
Bond lengths [Å]		
M-O_1	2.066	2.022
M-O_2	2.021	1.982
M-O_3	2.081	2.024
M-O_4	2.035	1.999
M-O_5	2.066	2.013
M-O_6	2.038	1.998
Torsional angles [°]		
O_1-O_2	79.622	81.943
O_3-O_4	78.097	80.963
O_5-O_6	78.602	81.266
O_1-O_6	162.500	169.727
O_2-O_3	164.374	169.393
O_4-O_5	155.825	162.160

Geometry Optimized Structures of [M(OK-COOH)]$^{n-6}$ Complexes.

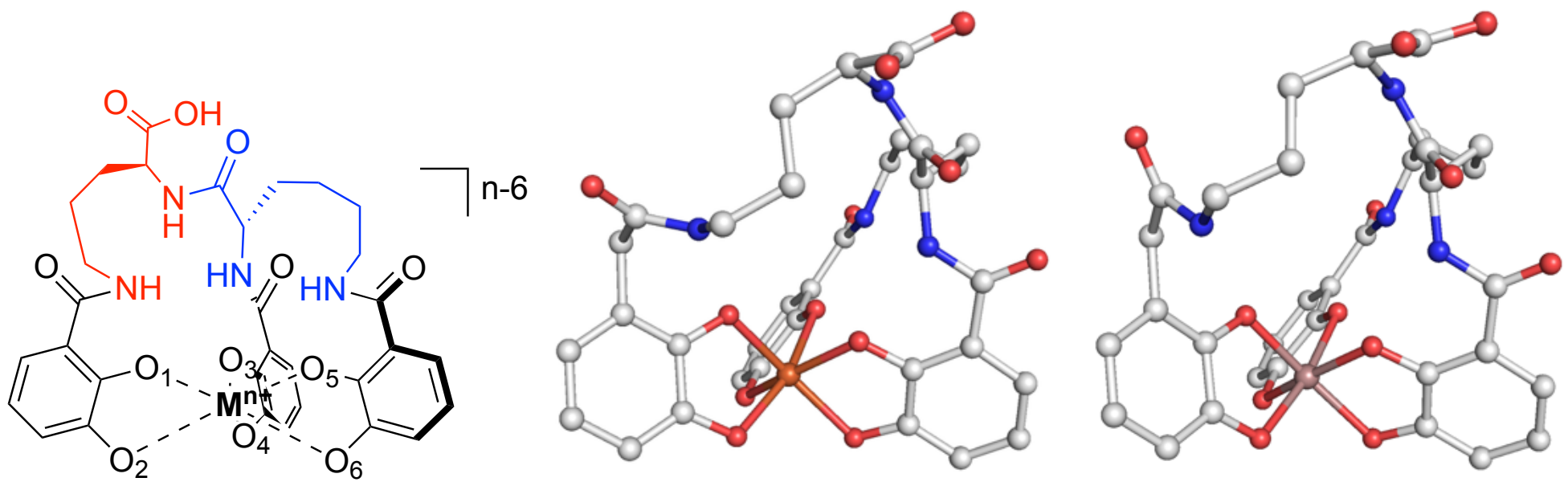

Figure S110. Chemical structure of **[M(OK-COOH)]$^{n-6}$** with coordinating atoms annotated (left), with DFT-optimized configurational molecular model **[Fe(OK-COOH)]$^{3-}$** and **[Ga(OK-COOH)]$^{3-}$** (left to right).

Table S9. Optimized metal-center based bonding of **[M(OK-COOH)]$^{n-6}$**.

**Bond**	**[Fe(OK-COOH)]$^{3-}$**	**[Ga(OK-COOH)]$^{3-}$**
Bond lengths [Å]		
M-O_1	2.058	2.006
M-O_2	2.020	1.978
M-O_3	2.115	2.030
M-O_4	2.018	1.982
M-O_5	2.050	2.008
M-O_6	2.052	2.015
Torsional angles [°]		
O_1-O_2	79.745	82.722
O_3-O_4	77.987	81.551
O_5-O_6	78.363	80.830
O_1-O_6	160.833	166.779
O_2-O_3	163.295	170.246
O_4-O_5	159.376	165.779

Geometry Optimized Structures of [M(OK-NH₂)]$^{n-6}$ Complexes.

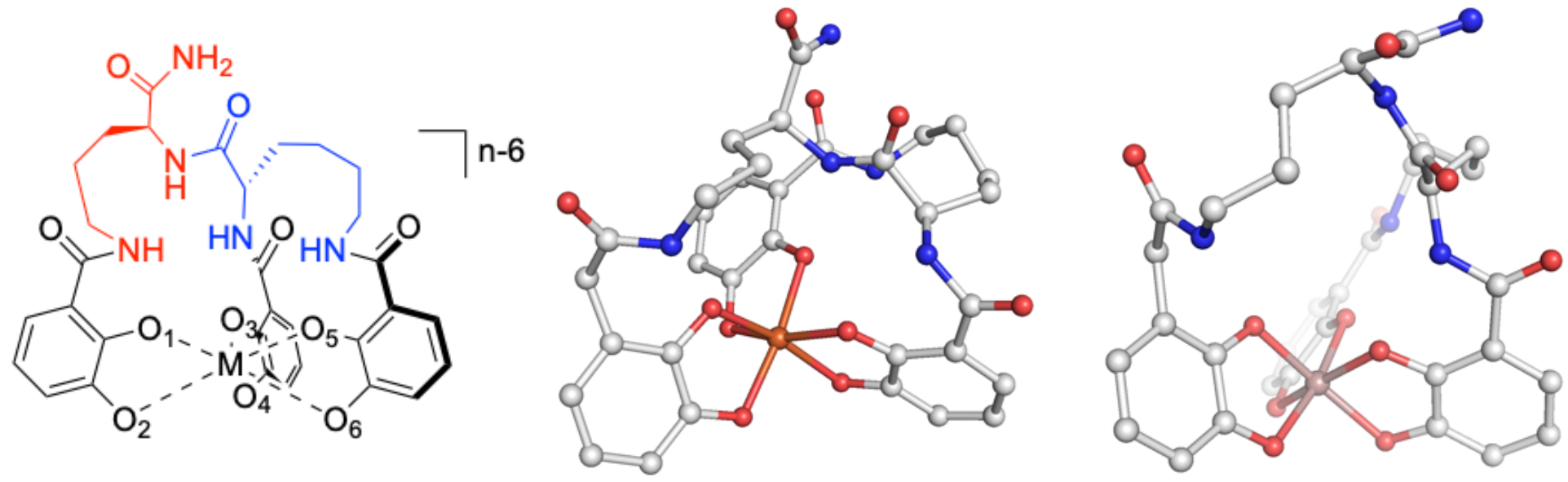

Figure S111. Chemical structure of **[M(OK-NH$_2$)]$^{n-6}$** with coordinating atoms annotated (left), with DFT-optimized configurational molecular model **[Fe(OK-NH$_2$)]$^{3-}$** and **[Ga(OK-NH$_2$)]$^{3-}$** (left to right).

Table S10. Optimized metal-center based bonding of **[M(OK-NH$_2$)]$^{n-6}$**.

Bond	[Fe(OK-NH$_2$)]$^{3-}$	[Ga(OK-NH$_2$)]$^{3-}$
Bond lengths [Å]		
M-O$_1$	2.074	2.008
M-O$_2$	2.016	1.978
M-O$_3$	2.108	2.031
M-O$_4$	2.011	1.981
M-O$_5$	2.055	2.007
M-O$_6$	2.038	2.015
Torsional angles [°]		
O$_1$-O$_2$	79.774	82.709
O$_3$-O$_4$	79.119	81.547
O$_5$-O$_6$	79.493	80.815
O$_1$-O$_6$	165.465	166.797
O$_2$-O$_3$	172.648	170.182
O$_4$-O$_5$	164.061	165.757

Geometry Optimized Structures of $[M(OO\text{-}COOH)]^{n-6}$ Complexes.

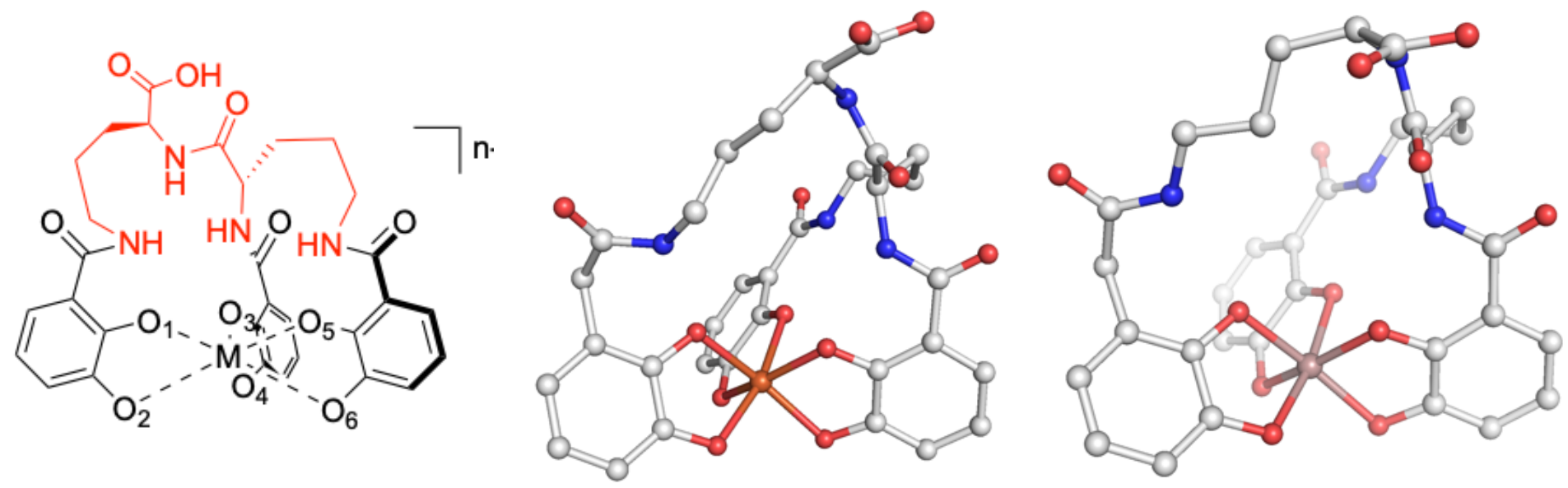

Figure S112. Chemical structure of **$[M(OO\text{-}COOH)]^{n-6}$** with coordinating atoms annotated (left), with DFT-optimized configurational molecular model **$[Fe(OO\text{-}COOH)]^{3-}$** and **$[Ga(OO\text{-}COOH)]^{3-}$**(left to right).

Table S11. Optimized metal-center based bonding of **$[M(OO\text{-}COOH)]^{n-6}$.**

**Bond**	**$[Fe(OO\text{-}COOH)]^{3-}$**	**$[Ga(OO\text{-}COOH)]^{3-}$**
Bond lengths [Å]		
M-O_1	2.066	2.034
M-O_2	2.019	1.977
M-O_3	2.088	2.043
M-O_4	2.027	1.981
M-O_5	2.050	1.988
M-O_6	2.049	2.019
Torsional angles [°]		
O_1-O_2	79.636	81.994
O_3-O_4	78.272	81.363
O_5-O_6	78.803	81.332
O_1-O_6	162.603	170.073
O_2-O_3	166.040	170.579
O_4-O_5	156.257	162.239

Geometry Optimized Structures of [M(OO-NH₂)]$^{n-6}$ Complexes.

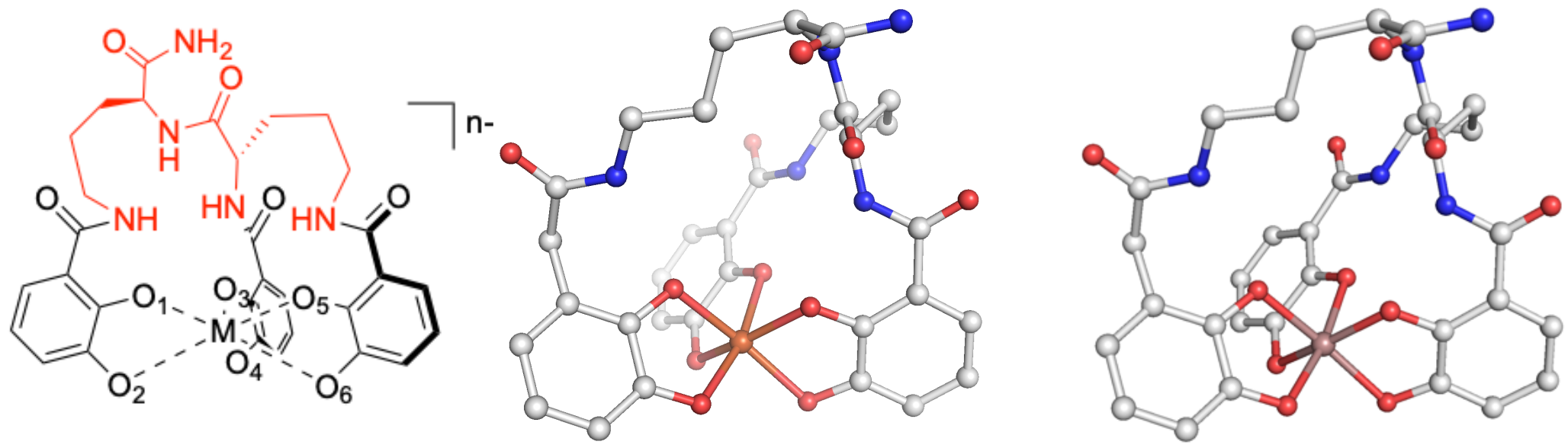

Figure S113. Chemical structure of **[M(OO-NH$_2$)]$^{n-6}$** with coordinating atoms annotated (left), with DFT-optimized configurational molecular model **[Fe(OO-NH$_2$)]$^{3-}$** and **[Ga(OO-NH$_2$)]$^{3-}$** (left to right).

Table S12. Optimized metal-center based bonding of **[M(OO-NH$_2$)]$^{n-6}$**.

**Bond**	**[Fe(OO-NH$_2$)]$^{3-}$**	**[Ga(OO-NH$_2$)]$^{3-}$**
Bond lengths [Å]		
M-O$_1$	2.078	2.032
M-O$_2$	2.011	1.976
M-O$_3$	2.016	2.040
M-O$_4$	2.015	1.983
M-O$_5$	2.018	1.990
M-O$_6$	2.075	2.018
Torsional angles [°]		
O$_1$-O$_2$	79.730	82.084
O$_3$-O$_4$	78.701	81.357
O$_5$-O$_6$	78.909	81.317
O$_1$-O$_6$	167.095	170.086
O$_2$-O$_3$	167.175	170.728
O$_4$-O$_5$	157.589	162.273

Coordinates for Geometry Optimized Structures for ferric complexes
$[Fe(CAT)_3]^{3-}$

Fe	-0.07869309492195	-0.18065176981239	0.00275688550666
C	-2.12238151114724	1.52375574354327	-0.96008438228280
O	1.41273475901752	1.11052584270889	-0.50486460472683
O	1.22620891049097	-0.50624717565386	1.54787239769391
O	0.65012268522864	-1.60009783469737	-1.27252672884973
O	-1.21767063618805	-1.77666977733426	0.54999274987988
O	-1.27732162482947	0.62532952375116	-1.42977807441532
O	-1.22268610182685	1.06182026280432	1.15178206843040
C	0.11492359305561	-2.79124537619124	-1.08510337096265
C	-0.90164050789292	-2.88922029246734	-0.08493681902223
C	2.45485627691850	1.05125686985482	0.30254865768230
C	2.35103681487350	0.17247644021296	1.42510041818320
C	-2.09849248687763	1.75470957468725	0.45046517181887
C	-2.98080140460309	2.67942426809335	0.99621218995385
C	-3.87649798806639	3.38641661217885	0.18737668321204
C	-1.49218833898882	-4.12272497374663	0.15955021000225
C	-1.10835921456931	-5.26021727539322	-0.55891989012955
C	3.62319182736367	1.78099413689334	0.12227365862934
C	4.69055402011888	1.66740975423585	1.01883359315129
C	4.58936049097315	0.82212935867675	2.10585028859800
C	3.42179646043757	0.07837528174568	2.30615492123956
H	3.69297100755285	2.44218946779268	-0.73478198084420
H	5.41155358364016	0.72926333699832	2.80508027539809
H	5.59223455720626	2.24542384062525	0.85625985704503
H	3.33567366318594	-0.59075190394132	3.15542817937256
C	-3.01819511708419	2.23782925191207	-1.74561107325436
C	-3.89478648628944	3.16773561753787	-1.17652599322899
H	-4.58561994407653	3.71148451396511	-1.80936554285249

H	-3.02604496022952	2.05693171906683	-2.81502315512390
H	-2.95691007181353	2.84676877714812	2.06761564669634
H	-4.55278778604530	4.10395148774528	0.63609857252170
C	0.47978045253569	-3.93255451753627	-1.78892047907500
C	-0.12912913121310	-5.16474891571897	-1.52842532986906
H	-2.25968180019593	-4.18677794606427	0.92326069801072
H	1.25166600274864	-3.84990456470739	-2.54639612807446
H	-1.58171082831499	-6.21207972317951	-0.35075956043908
H	0.17303074632669	-6.04152520393399	-2.08825458857536

$[Fe(Ent)]^{3-}$

Fe 0.624296 1.242668 4.167818

O -0.509933 0.562194 2.549116

O 0.949030 -0.804461 4.475598

O -1.128088 0.886687 5.215372

O 0.397925 2.906422 3.075868

O 2.555266 1.038808 3.704494

O 0.917101 2.282253 5.874172

O -3.361799 -0.065196 -0.471331

O 2.013818 -4.860904 4.395939

O -4.470361 0.480005 7.696304

O -5.045483 -2.995951 2.221082

O -2.027706 -5.503337 3.126209

O -4.269907 -3.770800 6.319071

N -2.237632 -0.933637 1.299161

N 0.365527 -3.326045 4.684428

N -3.278004 -0.354697 5.955832

C -0.884507 1.496645 1.730896

C 2.142820 -1.206536 4.172313

C -1.250906 1.555505 6.318725

C -0.331439 2.803301 2.002694

C 3.055488 -0.160804 3.773351

C -0.112998 2.374522 6.665330

C -0.617641 3.841714 1.126826

C 4.371848 -0.498324 3.490019

C -0.196469 3.174635 7.799209

C -1.477574 3.647540 0.028953

C 4.802942 -1.837898 3.543757

C -1.355711 3.174415 8.599016

C -2.056938 2.422799 -0.197391

C 3.924778 -2.842336 3.871504

C -2.420251 2.360284 8.295954

C -1.784932 1.331307 0.656512

C 2.578684 -2.549036 4.182264

C -2.375061 1.510114 7.168139

C -2.522709 0.077763 0.426301

C 1.663221 -3.674615 4.429768

C -3.466245 0.536495 6.975292

C -3.000834 -2.133022 1.292164

C -0.652882 -4.317410 4.698424

C -4.180365 -1.434653 5.758824

C -4.124594 -2.221462 2.327169

C -1.445766 -4.481077 3.399884

C -3.595387 -2.827177 5.982446

C -2.150222 -3.390216 1.411159

C -1.639243 -4.156334 5.844122

C -4.897914 -1.401316 4.413465

H -0.177779 4.814567 1.328878

H 5.052718 0.296920 3.199571

H 0.656949 3.795083 8.058002

H -1.698425 4.481601 -0.633242

H 5.835870 -2.076937 3.301849

H -1.401288 3.820152 9.472090

H -2.752536 2.261168 -1.011871

H 4.226462 -3.883417 3.886311

H -3.307128 2.326276 8.917616

H -1.569011 -0.692432 2.044271

H 0.168517 -2.315320 4.633629

H -2.442205 -0.195807 5.376862

H -3.512139 -2.178628 0.325257

H -0.156058 -5.282070 4.835695

H -4.959039 -1.329282 6.519851

H -2.768341 -4.285682 1.352591

H -1.403397 -3.389235 0.615506

H -2.389385 -4.946449 5.829113

H -1.095083 -4.176505 6.790136

H -5.563547 -2.257653 4.303221

H -5.465851 -0.471962 4.346971

O -2.290492 -2.888511 5.734314

O -3.949500 -1.389301 3.345322

O -1.439554 -3.382105 2.651737

$[Fe(PC)]^{3-}$

Fe	-0.61702142993036	0.48504707332694	-1.68407950117710
O	-2.09334739356094	-0.77328863943795	-2.33078248357789
O	-2.19390969353593	1.76214922938863	-1.90103285899574
O	0.59956778215338	-1.24476150528342	-1.90255435314179
O	0.48004229944421	1.87873597181827	-0.69530205852287
O	-0.87242361349848	-0.11036080350872	0.26428256351727
O	0.33536373490275	0.88662268276468	-3.39259310465929

C	-3.29930853592022	-0.24329084250707	-2.39038223349373
C	-3.34727966028301	1.17363227772366	-2.17303629379008
C	-4.48657050818122	-0.96839949758467	-2.63066952931780
C	-4.57435737720848	1.82566353693501	-2.25145681287195
C	-5.70859576232644	-0.26757456879365	-2.69076914271710
C	-5.75004253605448	1.10202806160098	-2.51398753319011
H	-6.61388100050566	-0.83026860650407	-2.87552381531459
H	-6.69663344829269	1.62647921031201	-2.57074550177424
H	-4.61064027001693	2.89901685582207	-2.09870604190799
C	-0.07542516887164	0.50114419686941	1.11686798731512
C	0.62110541494960	1.64648991176015	0.59671040589376
C	0.17568139994852	0.07909985934084	2.44100512486837
C	1.40068347930768	2.41477556249567	1.45455505182675
C	0.99382436918624	0.87606879122221	3.27181592627609
C	1.57173904429039	2.03496388575247	2.79724927360672
H	1.17429220809676	0.54238063989379	4.28487349834052
H	1.89674826532940	3.29640152514918	1.06301911983732
H	2.18354157679376	2.64479235848456	3.45142701425852
C	1.06527043881514	-0.10135933778959	-3.88563034167820
C	1.16187930676253	-1.29928030849207	-3.08818622654720
C	1.72179161762528	-0.03379403932375	-5.10791192072519
C	2.43104919786565	-1.14149121309373	-5.60138963127720
C	2.46159971431198	-2.31779614989444	-4.88427595240229
C	1.81340932514178	-2.43195693844762	-3.63302595780791
H	1.65850450094561	0.88357229306257	-5.68327420235283
H	2.93699731058002	-1.07311902616867	-6.55711049684936
H	2.96298804371354	-3.19090772026181	-5.27748625388187
C	-0.21759058435907	-1.27154801636331	2.91884142653405
C	-4.50542985086759	-2.45632885559261	-2.77015493385469
C	1.75755883962410	-3.78968819181333	-3.02890469201945
N	1.08434809858490	-3.91893205048129	-1.85685400515430

O	2.25721192567350	-4.77659847448752	-3.59523654173016
C	0.62565869848457	-5.21735939885488	-1.39625567747834
H	0.67315749747148	-3.05922748318310	-1.49624140827789
N	-1.19536296484744	-1.90306158025504	2.21186253298216
O	0.36202149655324	-1.82085898438633	3.86887602029004
C	-1.14857021778396	-3.35264065878860	2.01180191360680
H	-1.43600789996094	-1.38820174548090	1.36494186832945
C	-1.61708447005517	-4.22883309522276	3.16417758543072
H	-0.12242759353453	-3.62186101055456	1.76334978128264
H	-1.76214414355200	-3.55276351381325	1.13341257199360
C	-1.62099036147585	-5.71748270243843	2.79142027527648
H	-2.62468655898950	-3.93271464582772	3.47099950936500
H	-0.96038907898419	-4.07050709335222	4.02098345674483
C	-0.30886442682988	-6.25537995051677	2.19560132938787
H	-2.42926890413368	-5.92427111923406	2.08242157140257
H	-1.84399428700051	-6.29965866120526	3.68872619909147
N	-0.20996369690766	-6.03259982314591	0.75594517448728
H	-0.24556346756186	-7.33084271056275	2.37543097715262
H	0.55775250315291	-5.79073202444790	2.66270893333721
C	0.73223789174424	-5.30368961888967	0.13500520752835
H	-0.94293880510068	-6.43745054248674	0.19749218845877
O	1.66229759048150	-4.75530589322622	0.72832794215507
O	-5.56348743033987	-3.11032733835681	-2.78970195445347
N	-3.28693873741924	-3.02349732304008	-2.86323342274899
C	-2.95905464582668	-4.42922074584213	-2.73298229698964
H	-2.51759373301720	-2.35795375773393	-2.75161080890642
C	-1.85896190839302	-4.59037689547695	-1.66332625210595
H	-2.59980908370058	-4.81546783580664	-3.69238522491524
H	-3.86155604163136	-4.98294391489867	-2.47794566466929
C	-0.73745783164773	-5.56514599110287	-2.03163984692575
H	-2.31463716407032	-4.85985520755814	-0.71009737360973

H	-1.41235170956106	-3.60710059391148	-1.51214446128811
H	-0.57388914455632	-5.52993397825202	-3.11113790760905
H	-1.01060250133237	-6.60084931369016	-1.81101609673603
H	1.35013206969219	-5.94746398635061	-1.76379161313055

$[Fe(KO\text{-}NH_2)]^{3-}$

Fe	0.27168279289648	0.51518306576812	0.20595155098720
C	-2.26344598401912	1.29332551953309	-0.82645763343050
O	1.64487400787549	1.33956232417860	-1.07941909304385
O	1.76315645320696	1.03371546920309	1.50435115901717
O	0.99780352215745	-1.23648785321409	-0.63024055432621
O	-0.53791646765181	-0.87978927667838	1.43892329004396
O	-1.26964517240645	0.48160826945755	-1.17634203586727
O	-0.83168043522386	2.06448224524938	0.85976902304275
C	0.44158005758484	-2.32214843946889	-0.14106905660228
C	-0.38859201366245	-2.12557566578127	1.01876406066948
C	2.85055714683081	1.39386058482073	-0.54348708007768
C	2.89899019273602	1.31230347268359	0.89107680442508
C	-2.01296991748950	2.17348546374972	0.26924885285480
C	-2.99879194638274	3.08304221708116	0.65744449896925
C	-4.23198315710365	3.11225001269838	-0.00241049898585
C	-0.97889308898854	-3.23036843175375	1.62229094522881
C	-0.79195713430871	-4.51924917753820	1.09339020166562
C	4.05334718438841	1.46317007428104	-1.27678846120601
C	5.26844528531073	1.64661141194582	-0.58242848276469
C	5.29620713233560	1.69174256840645	0.79673355236950
C	4.11671937483720	1.50710788522456	1.53710230274498
H	6.23606752912174	1.83878131790282	1.31594056359784
H	6.18074584595575	1.71625640997915	-1.15868419943648
H	4.14438712812307	1.48032480048201	2.62126906231324

C	-3.51487949630929	1.30405240368692	-1.45666604234626
C	-4.48813460779149	2.22583941714602	-1.04058499708528
H	-5.45437885389503	2.22752828752557	-1.53259024889668
C	-3.82328688227901	0.30431521485986	-2.54683387834752
H	-2.79471194326719	3.75657612134249	1.48301610892105
H	-4.99328083760815	3.81792683389672	0.30905150172108
C	0.60216720697717	-3.61600926448187	-0.68066602410593
C	-0.02358343423600	-4.70638771193508	-0.03714596035318
H	-1.59541419993358	-3.07936802402158	2.50214755847565
C	1.36635378872156	-3.89046157592456	-1.93179840993864
H	-1.26248773224899	-5.36781873356630	1.57599924454925
H	0.10881520515612	-5.69275077965578	-0.45990502023377
H	-4.89998909407620	0.22224979723395	-2.68442911202841
H	-3.43892531388378	-0.67876625547049	-2.25795630137969
C	-3.22962420684137	0.65198686000977	-3.90639026530841
O	-3.94388907445063	0.85238434504264	-4.89709516317868
N	-1.88873984480868	0.70436573684247	-3.94740879927593
C	-1.16034887982166	1.03466151405774	-5.15958359713890
H	-1.39516834838717	0.57375127019572	-3.06265404560095
H	-0.17828666169519	0.58357896207951	-5.07009314037083
C	-1.05746459304692	2.54423495543240	-5.41201689709058
H	-1.66849355486471	0.56208778577249	-6.00204741164797
H	-0.30026267877231	2.97807557981897	-4.75170867401381
H	-2.01450751139967	2.97745172287026	-5.11313090640471
C	-0.79531461311071	2.96257319603639	-6.86760108236721
H	-1.38878880080114	2.32797680654530	-7.53582420752375
C	0.65205358328869	3.02807392849494	-7.37490834558586
H	-1.19782766325206	3.97235069569159	-6.98221054913037
H	1.28680014999862	3.51842187481268	-6.63683148012119
H	0.66289096874765	3.65610421103368	-8.26507908503446

C	1.30171434756157	1.70241261436663	-7.79315324774177
N	1.70170470476622	0.85140757514671	-6.68086447094550
C	2.44230948485169	1.91139903979441	-8.81248841504171
H	0.55716635551391	1.12372439371306	-8.34780907311113
O	2.41108783734824	2.83477086701340	-9.62267428504728
N	3.41071810404497	0.98228424116375	-8.81110865932797
C	2.57064332071880	1.22271286466804	-5.71293699990002
C	2.72877375722745	0.19237725251345	-4.58525215128231
O	3.17815753118563	2.28675543123212	-5.74061010164581
C	3.76199460886126	-0.88611110074805	-4.95869829449400
H	1.76519722206398	-0.31196164110980	-4.48123450166674
N	2.94748344903915	0.82705718008989	-3.30505067904713
H	1.24091976206786	-0.03952518099442	-6.59495164414426
H	2.15220230205007	0.88037111056117	-2.66510777660049
C	4.12495115339577	1.19013044250231	-2.74195969743371
O	5.19853373761144	1.21895397453993	-3.36061752545647
H	3.46124731162618	-1.31397698145435	-5.91902990226063
C	3.86332561039631	-2.00134045444176	-3.91427086064846
H	4.73452727959971	-0.41918490098667	-5.09609749958520
H	4.06899722705216	-1.56848281331127	-2.93383094599311
H	4.72875002982933	-2.61837505658656	-4.16320273156778
C	2.61171512092737	-2.90052810558636	-3.82261482797989
H	1.90815552502003	-2.63050184852031	-4.61950169677995
N	1.94092635138704	-2.82598532251212	-2.53659194057466
H	2.87772237857758	-3.94227172930915	-3.98545640165712
O	1.45731350670406	-5.04073003908581	-2.39995533945732
H	1.77170211935240	-1.92583689892269	-2.08631454504542
H	3.42743266071002	0.23867365231750	-8.13426937845649
H	4.15643978847678	1.04381499056362	-9.48593194795307

[Fe(KO-COOH)]$^{3-}$

Fe	0.23859236351958	0.62795674320922	0.07922266429025
C	-2.37524813766030	1.18960773727624	-0.78813497475363
O	1.62738835503864	1.39843742859325	-1.24281814860830
O	1.70801234859857	1.24036126374547	1.35127482765146
O	0.96936345370990	-1.15585672885211	-0.70567729054068
O	-0.53886113502388	-0.74525882306913	1.36346965508971
O	-1.28982851230901	0.62632107973348	-1.31132848775600
O	-0.94425799786256	2.08095113473704	0.83668458807698
C	0.48621151006551	-2.23617703009196	-0.13377739061988
C	-0.32980893833354	-2.00663405672789	1.02860367211255
C	2.82503034531543	1.49126080057399	-0.69471327798700
C	2.85194590913233	1.49185755386936	0.74308100219081
C	-2.18227443291714	2.00094208511442	0.37150293654677
C	-3.27922572589221	2.65036276284022	0.94183887783661
C	-4.55914360824526	2.48738877063047	0.39965453460020
C	-0.84529898662992	-3.09716799734022	1.72076564207222
C	-0.59591915713080	-4.40766009665472	1.27808407221711
C	4.04290212479454	1.52787711555741	-1.40675526709556
C	5.24298138590404	1.76240459653989	-0.70061398384424
C	5.24604192939467	1.88808838662932	0.67333327351637
C	4.05422475788724	1.73494883519254	1.40043014167100
H	6.17489531444120	2.07443807872029	1.19962023891619
H	6.16480508189894	1.80754073280349	-1.26385591436348
H	4.05983321377617	1.77004116939346	2.48465666927552
C	-3.66273078890471	1.01757192925717	-1.30988287106017
C	-4.74798534611459	1.67482617598195	-0.71066960041938
H	-5.74148528449030	1.53382820593016	-1.12172603556125
C	-3.85766373285788	0.15105826204375	-2.53121191829923
H	-3.12316619379536	3.27203997076356	1.81718990632687

H	-5.40491649893365	2.98829544081420	0.85607598437318
C	0.70896007531486	-3.55432725167371	-0.58654681941018
C	0.15934611277044	-4.63021858632008	0.14511895981306
H	-1.45115655439646	-2.91884085080289	2.60292589060100
C	1.46219270836299	-3.87283033608380	-1.83356271018878
H	-1.00792851592255	-5.24531549671691	1.82856894081629
H	0.33828399270004	-5.63460871982836	-0.21315431698912
H	-4.86873387117999	-0.25112006046193	-2.56275447967649
H	-3.14799434096968	-0.67922250312251	-2.51071257286391
C	-3.65242749326815	0.94667742959141	-3.81193408300423
O	-4.58401482777329	1.16755427155624	-4.59356445570992
N	-2.40041751283609	1.40744157274588	-4.00242736070220
C	-2.05811399309001	2.32664841666592	-5.07424887381050
H	-1.73127738791995	1.23334462107513	-3.25582035832654
H	-2.82984945475322	2.22995968246791	-5.83681360962287
C	-0.68844445790665	2.05150574040772	-5.68393169258896
H	-2.09492961077082	3.35969798730310	-4.70894204027181
H	-0.61188094950813	0.98452855792617	-5.90059852687226
H	0.09477956231835	2.28668625351307	-4.95634552253656
C	-0.50148468634556	2.87918597412239	-6.96014685028019
H	-1.14976224747972	2.47072303073278	-7.74257296520073
C	0.92345909619538	3.01179754030034	-7.49801561063285
H	-0.86205923541979	3.89456999910804	-6.76631411949708
H	1.56585631900016	3.47718446262704	-6.75033885963901
H	0.90758830616516	3.67689969298206	-8.36143844942769
C	1.57059758334229	1.70022532098806	-7.95266952315251
N	1.89382396106225	0.78536547211334	-6.86465327971507
C	2.79628861620351	1.93399976946975	-8.84468044806549
H	0.86473690154434	1.16297613275704	-8.59439192930697
O	2.97795528727531	2.93304975552628	-9.49529111749222

O	3.65520459986473	0.90834395995845	-8.93929498321628
C	2.75871071475049	1.12619618883883	-5.87442026930008
C	2.80539186236558	0.13763216126464	-4.70606648947404
O	3.45734498212806	2.12845131622671	-5.94636425094784
C	3.81981656106364	-0.98379439781635	-5.00489409124676
H	1.81581360611694	-0.31986892938544	-4.63967280326168
N	2.98728246986303	0.81163099258364	-3.43868219784078
H	1.27766791700999	0.00292791364907	-6.71413060827542
H	2.17325353909043	0.89172261009911	-2.82636540290848
C	4.15376726794999	1.17515678461904	-2.85129725254409
O	5.24741645323289	1.14728004797264	-3.43303534126315
H	3.52960195323638	-1.44304221698067	-5.95392623366899
C	3.87678310893572	-2.05601390634283	-3.91494959634946
H	4.80544557356639	-0.54439559533391	-5.13991664622768
H	4.06731116953557	-1.58639565004016	-2.94866120826394
H	4.73648187470196	-2.69738208286229	-4.11853839524780
C	2.60478521011598	-2.92574659676967	-3.81429617226826
H	1.90718677108216	-2.64726982438148	-4.61264286398788
N	1.93631908127384	-2.81718775133266	-2.53163282338233
H	2.84558598475140	-3.97555850741571	-3.96275521232770
O	1.62791397003729	-5.04694465583238	-2.21429338033522
H	1.72374511275214	-1.90201173832724	-2.13214828406998
H	3.38742418948437	0.19465917752465	-8.33406002789178

$[Fe(OK\text{-}NH_2)]^{3-}$

Fe	0.22679783939565	0.41047127539514	-0.50449815748373
C	-2.30575798226287	1.63655644746401	-0.86221050203456
O	1.55876349803858	1.75702236906012	-1.30099951779683
O	1.77263785931819	0.22600707349566	0.81019965395378
O	0.96878812618541	-0.94564702058549	-1.93819133017272

O	-0.73238287992515	-1.27365307560065	0.03371424351685
O	-1.37567140914531	1.08026252474651	-1.63745424491525
O	-0.70577047052815	1.57763021559187	0.84939520445191
C	0.38763645902987	-2.12947931185079	-1.84990099981411
C	-0.57266288152841	-2.29221107253386	-0.79356500494543
C	2.70854796292514	1.78706585590843	-0.67629702746425
C	2.80080971680961	1.00048377058945	0.53067660874724
C	-1.92295914512130	1.94327357561178	0.47980755749934
C	-2.82656007957709	2.59341006823284	1.32291592339854
C	-4.11231696220959	2.90731727528556	0.87248125080074
C	-1.28191929280832	-3.48694880874351	-0.69046132304205
C	-1.06165062736482	-4.53247716939878	-1.59724185261384
C	3.83993663291542	2.51783184359794	-1.11080842718486
C	4.98249024635183	2.59374227705284	-0.27301821430144
C	5.02940557122869	1.90318438370821	0.91528359355307
C	3.94662902400417	1.09459352403381	1.31137858699428
H	5.91315918040674	1.95538926425018	1.54005542530415
H	5.83179545437741	3.16827095415669	-0.61631092168441
H	3.99884057435424	0.51959418461960	2.22965787799070
C	-3.61354329133179	1.91008493908620	-1.28788873529198
C	-4.50159044844580	2.55659725599636	-0.41279452319492
H	-5.51085419832115	2.76357200578039	-0.75105407246831
C	-4.10407515968059	1.45458359380670	-2.63986705705839
H	-2.51624754875076	2.83617552378407	2.33367004202291
H	-4.80990296273246	3.40706283010749	1.53441785871853
C	0.63650698838373	-3.20872576158936	-2.71403411688440
C	-0.11789185035019	-4.39399291204121	-2.59760287320355
H	-2.01162210835699	-3.59018034614063	0.10550282708093
C	1.71807550974712	-3.20813938699993	-3.74127756933670
H	-1.62583850196107	-5.45287229210740	-1.50576336924895

H	0.07643805358364	-5.20389034931365	-3.28885424210788
H	-5.19280716472368	1.43541741321490	-2.65163447645714
H	-3.75690322194287	0.43129960453777	-2.82680997497864
C	-3.67307758676595	2.25385566845273	-3.86176800814037
O	-4.46172887464063	2.46998312026171	-4.78873428556418
N	-2.38619433687329	2.64779593886935	-3.87431416197838
C	-1.70695617074425	3.06009648757519	-5.09557728048782
H	-1.80601139911436	2.26360648972327	-3.12811739502224
H	-2.43977429092719	3.03189330164448	-5.90063127218134
C	-0.52415943184088	2.13885431387049	-5.39600700273637
H	-1.35507519768081	4.09102609297815	-5.00325918064163
H	0.24161010199466	2.30827980383160	-4.63697851252131
H	-0.08875382401135	2.42665038883318	-6.35321155199834
C	-0.91978924574784	0.66505183694058	-5.39769487963752
H	3.68033935917278	-1.23332044297518	-5.37233337240311
H	3.92434798804862	-3.69730667255009	-4.90686640113651
H	-0.15172404238034	-1.29890840968365	-5.13149312763507
H	3.01841620367948	-2.42534559013514	-2.40886740987492
H	4.94055431107702	-3.29099687704417	-3.52371394579087
C	0.23083046815430	-0.34315885589423	-5.49873059530002
N	1.34446361599744	-0.01288163802224	-4.61577406972436
C	0.67484276687956	-0.63195422998790	-6.94504238890854
C	4.13278653267225	-2.92954141515399	-4.16439928478435
O	0.24315584804317	-0.00373952755639	-7.90887472191831
N	1.50546155962065	-1.68264701638335	-7.06744552874624
C	2.36516543706347	0.78902244195927	-4.95805898696436
C	3.31615514321771	1.13848747762798	-3.80911875258466
O	2.53898942742116	1.22889595870517	-6.09517233216619
C	4.77867700669620	0.86098124930501	-4.16576469963800
H	3.05994665955588	0.51444601417044	-2.94931130360123

N	3.03323064002827	2.56874781151163	-3.46604774751187
H	1.26032561977287	-0.31734284290151	-3.63803178976534
H	2.12860886231658	2.51509338404895	-2.99261423152000
C	3.94640974845939	3.06653407874674	-2.46755446335146
O	4.82481587942945	3.84854461714398	-2.80684049627288
N	2.93657646247813	-2.80238344678531	-3.33971181015887
O	1.52391058466462	-3.64613201848119	-4.89025402127728
C	5.15164607044459	-0.59689585087154	-3.88772124831843
H	5.41900744108853	1.49644825676024	-3.55454101718498
H	4.96709597910638	1.13225868957528	-5.20703909256530
H	6.23970264492298	-0.69569822694085	-3.89530158490182
C	4.55045419633279	-1.63175841986543	-4.85401506886444
H	4.83724219986772	-0.82422587866675	-2.86438611839742
H	5.28020635043849	-1.87550118059513	-5.63053376559893
H	-1.44036737634262	0.44487974037105	-4.46487802938517
H	-1.62353144590445	0.46326173001443	-6.20773948782100
H	1.77450305914876	-1.97484640881764	-7.99401724604277
H	1.67152056839256	-2.32110447731865	-6.29391051012310

[Fe(OK-COOH)]$^{3-}$

Fe	0.13612740106296	0.21366051680025	0.01933289496738
C	-2.33851312923961	0.91083655000015	-1.15057374033382
O	1.50909452523424	1.13414780121386	-1.19280516466218
O	1.57463271390911	0.77167365014836	1.37241829764500
O	0.97790242231760	-1.58608377159492	-0.70621056094302
O	-0.66455154630539	-1.15689810192457	1.26556489648982
O	-1.21862025334955	0.30679713339904	-1.52660681280107
O	-1.14606892238964	1.59423450605068	0.74749843146280
C	0.70464137369125	-2.62840997365952	0.04735970926013
C	-0.18734777107644	-2.38404919324830	1.15133043639950

C	2.66313512985910	1.39280302219776	-0.60913952961175
C	2.67555913113243	1.24671697916040	0.82016742587851
C	-2.30203118404729	1.60462922504152	0.10059349681933
C	-3.45024173964510	2.25433639530849	0.55789139940284
C	-4.62474649850281	2.23807660306230	-0.20389363562816
C	-0.50098711858851	-3.41873023642056	2.02471608381522
C	0.02989694223260	-4.70470774035987	1.83024128401679
C	3.84474652301118	1.75542233612420	-1.29010306474139
C	4.98956176699448	2.09452768359511	-0.53681665750003
C	4.97548793334983	2.02949206165394	0.84160758279130
C	3.82515591169589	1.59695348231176	1.52292459755204
H	5.86204436734414	2.29499193509587	1.40550591880414
H	5.88661868603907	2.37973156016919	-1.06893738349940
H	3.82539126702083	1.50926586347125	2.60416274113319
C	-3.51180203297182	0.92649222292004	-1.91702773608839
C	-4.65085780696124	1.58646777060094	-1.42959237961325
H	-5.55456735664012	1.58781402177784	-2.02907724133417
C	-3.50826126909290	0.33162238603025	-3.30752231359693
H	-3.41432331645458	2.77281216336188	1.51019633492191
H	-5.51283233053082	2.73810485379298	0.16467538706381
C	1.21126531601352	-3.93257548725135	-0.15103699010565
C	0.86448940691624	-4.95385944165276	0.76187853499560
H	-1.16645004279404	-3.21507507736606	2.85694568007996
C	2.06052195608217	-4.31803429563640	-1.30945868906479
H	-0.22334874676820	-5.50069009031420	2.52061193153029
H	1.26571359434712	-5.94319894051756	0.59194448126531
H	-4.47201711142369	-0.11135220187876	-3.55409732235772
H	-2.73506949763879	-0.43452490245597	-3.38344530432397
C	-3.23984507582800	1.42971621214461	-4.33022555366198
O	-4.09881978425871	1.80075674175705	-5.13454075397731

N	-2.01426014309675	1.99024576696766	-4.24712825631150
C	-1.65962740021923	3.25995819014276	-4.86773192171530
H	-1.41700449846412	1.65529194068840	-3.50038311182354
H	-2.47334464685274	3.53279024324448	-5.53765637699195
C	-0.33090793998105	3.19453353807270	-5.61543456213227
H	-1.59896536140363	4.02534009664874	-4.08842911705082
H	0.39173286596690	2.70945035873406	-4.95914654908603
H	0.03106779619940	4.21019968892917	-5.78137369319302
C	-0.44205296530280	2.45537601925776	-6.94578422407208
H	1.96334868084801	-2.05643071421198	-4.43789175571215
H	2.41423831431981	-4.53525552503169	-3.93118614500698
H	0.62111728239864	1.53025282734690	-8.54377650803433
H	1.94545569602874	-2.44112793769945	-2.04126062559825
H	3.98717137370160	-4.04585814487857	-3.33521854644977
C	0.87568004251181	2.11316228933432	-7.65224004210732
N	1.73581474374745	1.25080952238968	-6.84833371339235
C	1.63310344765792	3.31168968318590	-8.21537540678249
C	2.96164410901734	-3.69608517155782	-3.49276863926527
O	1.16504681941216	4.41787777173973	-8.32075377156046
O	2.84462008361710	3.04631892747498	-8.73211648107721
C	2.47494163451554	1.72016538968629	-5.80335637934946
C	2.78166424380782	0.65869009545945	-4.74220553834697
O	2.81919578002711	2.89067925813657	-5.72321047971604
C	3.95021566506268	-0.26485705253337	-5.12589013181326
H	1.88422890027932	0.03849788065412	-4.70266682140777
N	2.87423290426501	1.22811351470784	-3.41641203757015
H	1.42821020940005	0.29095778020264	-6.79566363170151
H	2.08386162176781	1.05868089598830	-2.78929638444725
C	3.98126678360911	1.67366216627110	-2.77331955834337
O	5.04517263329086	1.93918473566135	-3.35184413336267

N	2.34853349030482	-3.36010869770488	-2.21628938319828
O	2.46821001076963	-5.48499389252157	-1.46543826724125
H	3.10360332484421	2.12821046224670	-8.54723930866148
C	4.04307269152303	-1.47623018025127	-4.19352659873766
H	4.87554734577189	0.30563306690231	-5.10588424283746
H	3.79189018448848	-0.60608741818197	-6.15254735719462
H	5.01928310996893	-1.95103153280238	-4.32491738113515
C	2.95537388268919	-2.51772060351392	-4.45882320027240
H	3.99945625858608	-1.14233423054699	-3.15326484252965
H	3.08985773517984	-2.90842293516888	-5.47118694288726
H	-0.95954131751338	1.50576039527871	-6.78462816984259
H	-1.05605048789159	3.03525985014303	-7.63836600158909

[Fe(KK-NH$_2$)]$^{3-}$

Fe	0.22197501137677	0.49214719013400	0.05957759043089
C	-2.20867910247524	1.41638488944436	-1.09725891981527
O	1.62530897921503	1.19787762032683	-1.25236418756758
O	1.76328017253849	0.93806442357565	1.33076713916100
O	0.78394893227763	-1.39163460555054	-0.61790874148086
O	-0.64376728669505	-0.68649892616621	1.45154981235314
O	-1.30947916343992	0.46789426828583	-1.33674836538408
O	-0.73863620456420	2.18808795474814	0.55799585475272
C	0.31414112879405	-2.37455803322571	0.11873179156823
C	-0.44716703154514	-1.98295767815319	1.27750973335436
C	2.81636650565002	1.39082560687321	-0.71533589330304
C	2.87369560940319	1.30037709478944	0.71756616319372
C	-1.88258285641804	2.37109259094643	-0.08568012837303
C	-2.76511089237162	3.42066771772675	0.17737173924829
C	-3.97623574964321	3.51734476031799	-0.51658744194613
C	-0.92971071478610	-2.96338319115372	2.13741164533329

C	-0.70661117155855	-4.32452825102060	1.86912575790396
C	3.99661776657812	1.63576313696059	-1.44797727272860
C	5.19426888017471	1.90502592024449	-0.75176265085149
C	5.22972516156325	1.88953666627396	0.62768852694608
C	4.07492857608896	1.57898592650933	1.36461603473815
H	6.15627930474489	2.09931558004563	1.14924359563507
H	6.08863708228290	2.09709558836308	-1.32850149864228
H	4.10852937552115	1.52955284082393	2.44779828980666
C	-3.44460476237421	1.49595919524691	-1.75227125516669
C	-4.31535230086884	2.55760927225842	-1.46099074918945
H	-5.26971330565183	2.60952045442881	-1.97310641350844
C	-3.85726957460173	0.41158416629155	-2.71858473278107
H	-2.50083948948636	4.14890502133734	0.93673525234623
H	-4.65722689989375	4.33254287926934	-0.30148918832810
C	0.50021194371038	-3.74592659951688	-0.16061973090091
C	-0.01155666613202	-4.70611612013031	0.74049572686376
H	-1.49083986708093	-2.65841530819275	3.01440266849800
C	1.15690783407764	-4.24303350388367	-1.40081554107884
H	-1.09129824185144	-5.07459018339801	2.55018206994227
H	0.14351308480065	-5.75181567522159	0.51412554488980
H	-4.93980325487505	0.40968129553712	-2.83360772406848
H	-3.55191383687646	-0.56078884341408	-2.32059177667951
C	-3.27229964368336	0.53884170724685	-4.11849871878723
O	-3.99461610022187	0.71521515134009	-5.10726726486353
N	-1.93547953111578	0.41668925721850	-4.20195590543920
C	-1.24247748149673	0.41275140502489	-5.47857445570322
H	-1.42439825927442	0.32037841158115	-3.32542920289097
H	-0.41828310457436	-0.30029922793590	-5.41411383764875
C	-0.70973534590117	1.77543708718593	-5.92490382176852
H	-1.94198592964744	0.03602729719984	-6.22532925105396

H	0.13673277985865	2.05720268085270	-5.29465833355018
H	-1.47951253646025	2.53498227913842	-5.76465918747023
C	-0.30306988325060	1.75762606865913	-7.40476970777744
H	0.15166478115342	0.78935994383530	-7.63277421006876
C	0.65760770556289	2.89044093085504	-7.81827178177354
H	-1.21141343870480	1.80525422870417	-8.00943080377633
H	0.72769197734882	3.63409509529341	-7.02434497589118
H	0.28374133155649	3.41007930960345	-8.69894815399799
C	2.06628266679198	2.37743310190819	-8.15964336089998
N	2.60234851310061	1.45587522630555	-7.16237845717767
C	3.04046272641561	3.51416851474294	-8.49729930304587
H	1.99407732314803	1.78834595556429	-9.07833669045431
O	2.63492354965559	4.59645342829598	-8.91471905047581
N	4.34549378775379	3.21562286125994	-8.38907278608839
C	2.75827309492603	1.81502841520793	-5.86444423144997
C	2.77112076990450	0.64757743378926	-4.86908933210841
O	2.78484988084101	2.98836337189139	-5.51659617042541
C	3.72062401318857	-0.51788057295619	-5.20941753653856
H	1.75133027481388	0.25996636910496	-4.93179187806926
N	2.91053061366144	1.10533372424797	-3.51294864081214
H	2.50650836153111	0.47514923428945	-7.36844183073278
H	2.12397335432463	0.99701296180335	-2.87059475927562
C	4.05956276190235	1.50961569709284	-2.92985657339552
O	5.10416700906934	1.70498864442663	-3.57201594778261
N	1.58244481167038	-3.31856939373968	-2.28827718640230
O	1.27996448077502	-5.45996816378302	-1.63776351450260
C	3.52320185633414	-1.71927291655979	-4.27864781213673
H	4.74679190379846	-0.16021844297426	-5.14658066993158
H	3.55186634906878	-0.84133684817375	-6.23909700238366
H	4.35452262046625	-2.41373089034027	-4.43011733071605

C	2.21430037005183	-2.47179170425835	-4.52321330342940
H	3.58095749739859	-1.38829905853234	-3.23847388701991
H	2.20001859958983	-2.81350240992061	-5.56214503166196
H	1.35434216825901	-1.80499486536927	-4.40802842088356
C	2.00923843988113	-3.69019174046965	-3.62833183743477
H	2.93286853701119	-4.27608677183719	-3.57816236073788
H	1.25167621864473	-4.34594091228868	-4.06747761370086
H	1.41029337558199	-2.34661083723202	-2.03485698608992
H	5.03427344275214	3.92024236231983	-8.59962383110552
H	4.64541359003008	2.34968464494965	-7.97312153114054

[Fe(KK-COOH)]$^{3-}$

Fe	0.24762152018606	0.43076954176712	0.08720080628896
C	-2.18509704068774	1.37765041711138	-1.05267375217567
O	1.64269817017654	1.18692300441698	-1.20590545685205
O	1.78401897975395	0.86177029569111	1.36787899625251
O	0.81429138971781	-1.42678767862794	-0.64358153473023
O	-0.61405460794650	-0.79078007060286	1.44532532251773
O	-1.28594205698905	0.43352661849822	-1.30882778209097
O	-0.71806702233474	2.11436954536247	0.62120624527072
C	0.31087244392590	-2.43408033840008	0.03666333009236
C	-0.45387741801087	-2.08162994065869	1.20516863682837
C	2.81950004252964	1.42795986913482	-0.65902014258645
C	2.87657572144399	1.29830582157978	0.77138772981763
C	-1.86049682875231	2.31117295351708	-0.02101842955689
C	-2.74374843118610	3.35466914832821	0.26298689672530
C	-3.95328470671980	3.46674470679976	-0.43148490237464
C	-0.97797841632216	-3.09160830754074	2.00453193450746
C	-0.78883816483126	-4.44259147180684	1.66770087140206
C	3.98649541869462	1.75977210377020	-1.37917472560747

C	5.16653620216042	2.06979403510042	-0.66901564702024
C	5.19849563682455	2.01377228073609	0.70918457865367
C	4.05914581366312	1.62110782982280	1.43144899944455
H	6.11092883146148	2.25660416245797	1.24111459615484
H	6.05117368331086	2.32748772227577	-1.23496452475054
H	4.09199731094797	1.54246780895748	2.51292636675701
C	-3.41945166947544	1.47183238005639	-1.70879114352335
C	-4.29046826749780	2.52743698939752	-1.39676727227760
H	-5.24360922419907	2.59044275186965	-1.90994594891003
C	-3.83300018046578	0.40797853529109	-2.69735463079852
H	-2.48102636668962	4.06639819440801	1.03837535660214
H	-4.63469648950685	4.27720192439373	-0.20041516654466
C	0.46634893061953	-3.79356546485027	-0.31026873392588
C	-0.08552261455449	-4.78462905523900	0.53130135119021
H	-1.54428642868327	-2.81706370124848	2.88821494886724
C	1.13377844951382	-4.24229819020756	-1.56363092629675
H	-1.20608371371726	-5.21601674176991	2.30184120392507
H	0.04511941418274	-5.82102245439078	0.25236315202938
H	-4.91517332387679	0.41289013678965	-2.81557137062592
H	-3.53341802805192	-0.57333364850750	-2.31701937169398
C	-3.24334086936141	0.55765555778798	-4.09286943913815
O	-3.96192202829173	0.74843381030732	-5.08162253739834
N	-1.90617506723604	0.43765676307234	-4.17385817285223
C	-1.21166496646769	0.44706985184668	-5.44944019693302
H	-1.39732332493006	0.32428191285733	-3.29800074775019
H	-0.37881439452599	-0.25569290812897	-5.38633591981636
C	-0.69646012197537	1.81819629199790	-5.89000700034878
H	-1.90484348413517	0.06474236364490	-6.19942894021437
H	0.14546431411326	2.10973924279762	-5.25773418858244
H	-1.47604753752541	2.56726141568512	-5.72879514058716

C	-0.28826205226272	1.81025368341964	-7.36912105250240
H	0.18861224334251	0.85310249007289	-7.59860205846035
C	0.64533688601515	2.96682646869837	-7.77674609668117
H	-1.19573608213837	1.83868854869947	-7.97575023145548
H	0.69766394883494	3.70905229306587	-6.98020094569902
H	0.26246040943031	3.48049217826333	-8.65746537320779
C	2.06583805081451	2.48956821444893	-8.11634018621146
N	2.64132412064920	1.57601171977565	-7.13406147048279
C	3.01214020891846	3.64829083181039	-8.42317045851870
H	2.02172388822462	1.91697379976114	-9.04801291560057
O	2.64176740093168	4.73234987336585	-8.80163052161616
O	4.32227688700423	3.37921639253834	-8.32989166244294
C	2.79836390078526	1.95481826573598	-5.83395452579088
C	2.81398554426883	0.80540312961644	-4.82270801800597
O	2.82904645522176	3.13514336902802	-5.51876957216811
C	3.79012804027623	-0.34774286370410	-5.13141899171245
H	1.80220396940156	0.39952883634698	-4.90062229883279
N	2.91994666691204	1.28015989685853	-3.46891651865232
H	2.52650929448352	0.59411825245019	-7.32830510015270
H	2.13972066838813	1.11522146776399	-2.82966073531098
C	4.06010724353822	1.68327246235519	-2.86335578022913
O	5.10391990543232	1.91862469039781	-3.49256045077702
N	1.58241952640098	-3.28155616524825	-2.39891212912794
O	1.24647989960916	-5.44828578142789	-1.85508409238521
H	4.44707947245206	2.48181066370237	-7.97372799533951
C	3.55092860111151	-1.56730442316127	-4.23390013156275
H	4.80697450956318	0.01900969464046	-5.00398461854478
H	3.68455157538779	-0.65419514602226	-6.17434119448204
H	4.39828991536479	-2.24947093251697	-4.34774203842996
C	2.26821145049527	-2.32791023730394	-4.57203648615392

H	3.54341407750852	-1.25516023932953	-3.18612436727797
H	2.30729231207268	-2.62299562787809	-5.62455589034822
H	1.39312798946226	-1.67964473056581	-4.46653061150015
C	2.04124214811287	-3.58880987180428	-3.74397206045719
H	2.96334506153095	-4.17785148541864	-3.70049077817657
H	1.29421533442014	-4.21989143893068	-4.23423221810774
H	1.42055513735660	-2.32115345065555	-2.09840757975804

$[Fe(OO\text{-}NH_2)]^{3-}$

Fe	0.36969562706516	0.81142346331604	0.07781451015964
C	-2.21133941285899	1.48586295975349	-0.84091310123239
O	1.75709253976108	1.62507847938121	-1.14147767129206
O	1.86429453326941	1.28808807082557	1.43584246349025
O	1.09672021072666	-0.97029352166105	-0.77727404783020
O	-0.48231622098506	-0.57396623637915	1.26773384169439
O	-1.17752696512883	0.79759234438686	-1.30971229220521
O	-0.72006984949330	2.36570780350780	0.74056030249354
C	0.45906990453286	-2.02804012631588	-0.33156531518355
C	-0.39968148700323	-1.81145706789582	0.80740366335514
C	2.96495632786756	1.61472654764528	-0.60292898628015
C	3.01068731488086	1.52542809614634	0.82755792403102
C	-1.95155348870067	2.37566743296139	0.24859746697240
C	-2.97955468668346	3.18178932235698	0.73981398854275
C	-4.27131584011403	3.08278770868300	0.21006737418361
C	-1.08814411980905	-2.88566248094352	1.35807286481420
C	-0.97248590189799	-4.17097304453263	0.80248153847129
C	4.15999399682399	1.60922577926946	-1.34465313847976
C	5.38971569272845	1.73128786680017	-0.66646908275412
C	5.42606835066625	1.77964260094360	0.71479433646185
C	4.24431113144671	1.65824528663557	1.46442747159184

H	6.37583720267424	1.87412543400262	1.22814795688624
H	6.30206751984132	1.74234741142667	-1.24771644183822
H	4.28164331621925	1.62606792051230	2.54820570117168
C	-3.52160824492113	1.35160640706079	-1.31951453871799
C	-4.53971856816148	2.16459897669705	-0.79572495192089
H	-5.54935842462910	2.05130645295734	-1.17515514382876
C	-3.84484610695419	0.29617568321043	-2.34887034459090
H	-2.76469519971213	3.86676334196452	1.55329384254432
H	-5.06569197199944	3.70566286389017	0.60456117296678
C	0.55388004313453	-3.32107211621903	-0.89029407840464
C	-0.17340333110334	-4.38037037591236	-0.30114629426582
H	-1.72489165027420	-2.71082501738335	2.21888823417034
C	1.36886735372952	-3.64684990193937	-2.09337168445879
H	-1.51971239894563	-4.99655342186141	1.24218495556599
H	-0.08781554568735	-5.36299423742986	-0.74338708203136
H	-4.87890089644120	-0.02761577400077	-2.24027045990182
H	-3.19522400531799	-0.56957113397658	-2.19217367195748
C	-3.68306268453414	0.73548223813579	-3.79476149674496
O	-4.62957922458987	0.72188830606671	-4.59065753625656
N	-2.44923323099610	1.11914725654879	-4.16374980837379
C	-2.14398124471991	1.42287943596388	-5.55589604461371
H	-1.70859830325789	1.06442586146174	-3.47210688946106
H	-2.49883487038254	0.59341557927132	-6.17478650343328
C	-0.65288581922501	1.62897414174394	-5.77790463764901
H	-2.70230217314534	2.30838981191495	-5.87233309746022
H	-0.11766587059761	0.85439978017228	-5.22411509165031
H	-0.33850888751474	2.59107873337663	-5.37279817830435
C	-0.31111291072570	1.51891175215161	-7.26295591987067
H	-0.63800468833177	0.53568568451462	-7.61238756132640
H	2.29521984382027	-2.93413311095308	-4.71977784593644

H	1.28555403810779	1.23340847241526	-8.64173697616350
H	2.04591649781137	-1.74310310379005	-2.21348729121046
H	4.41691051864384	-1.54034498694216	-3.07609564389409
C	1.17064527302233	1.65895400870285	-7.64123564272952
N	2.05255716049451	0.87599047421547	-6.77720644017822
C	1.64617901105509	3.11196176756160	-7.79694352460262
C	2.93101266828001	-2.92402347134939	-3.82554345650937
O	0.86440895775623	4.05652460582216	-7.76817828656987
N	2.95014379300641	3.24910435063051	-8.10571282448815
C	2.39885257033559	1.29092018919875	-5.53226861184601
C	2.77250696888285	0.17191413157303	-4.54602838528721
O	2.33262992324667	2.46382074537546	-5.19063088066251
C	3.83692194582838	-0.81865311905268	-5.03533306681263
H	1.83895814023584	-0.39111553812808	-4.43748704860919
N	3.03834740669736	0.70368162255378	-3.23792944197723
H	2.05072270319756	-0.11743379944197	-6.95162559242442
H	2.25115187714978	0.79941698707117	-2.59707818802716
C	4.17530109415206	1.29097297362487	-2.80214007522106
O	5.17664712205555	1.45418916045744	-3.51417299159248
N	2.11954646082293	-2.66294637900410	-2.64238551017827
O	1.36534171659067	-4.79226985430359	-2.58336476094007
H	-0.87129568554641	2.26014337579989	-7.83557422627585
C	4.09983220313333	-1.95032890872094	-4.03699035419113
H	4.76435997429221	-0.28451150063910	-5.23195103140420
H	3.50506478599166	-1.25900699541654	-5.97811462617591
H	3.31902658347102	-3.93627181622227	-3.72197362078987
H	4.94482959384819	-2.52651158264108	-4.41915081206891
H	3.34195591901652	4.17624886032489	-8.15938054549490
H	3.58486456747469	2.47563362627160	-7.99090776158671

[Fe(OO-COOH)]$^{3-}$

Fe	0.17658653442368	0.60410457398791	0.08190409700746
C	-2.42199991036894	1.31657580452519	-0.78094652634096
O	1.53084487438206	1.49166172269370	-1.17503991435610
O	1.65939508153532	1.12252612010817	1.39684796287418
O	0.94940700255099	-1.10742401991317	-0.83076011813669
O	-0.57920085147867	-0.86536058210352	1.25521438971863
O	-1.35892260914173	0.70264162269126	-1.29631403116587
O	-0.99221412576697	2.00902632065124	0.94014563463932
C	0.55029589833711	-2.23421768875727	-0.28546387713167
C	-0.28308162872498	-2.09813936009867	0.88037686949478
C	2.73083786089897	1.59143020666253	-0.63366106088691
C	2.78047819470777	1.47709668274360	0.79744793050717
C	-2.21494878274570	2.04191468295875	0.43130134432272
C	-3.28282076260590	2.73772655541214	1.00327077883474
C	-4.54639811223393	2.71139447272354	0.40362572839570
C	-0.72455002555277	-3.24093883200767	1.53793065666820
C	-0.37634020916441	-4.51768346623763	1.06502065041564
C	3.92988945889483	1.73786892490570	-1.36098398862275
C	5.13171835205844	1.97495455349062	-0.66020911599156
C	5.15371469813656	1.98989935919694	0.72013671896701
C	3.98493747660851	1.72263835633856	1.45222859795916
H	6.08321123964850	2.17518680254914	1.24569923447667
H	6.04081624268116	2.10433649612547	-1.23139958296697
H	4.01246620976240	1.67110872767293	2.53549586166387
C	-3.69375896588059	1.28623893103297	-1.36726490049097
C	-4.74884911133279	1.98861358232767	-0.76506508703184
H	-5.73114799077778	1.95570233799265	-1.22308177771959
C	-3.91647864235125	0.51845575539136	-2.64901403834490
H	-3.11535749228765	3.29318112110492	1.91999871452045

H	-5.37022740103110	3.24847547040484	0.85907444850632
C	0.87955704643205	-3.52112104093767	-0.76267970672443
C	0.40351779987893	-4.65335141799852	-0.06431039729303
H	-1.34570138508644	-3.12939970205764	2.42039927874261
C	1.68401798298642	-3.75979916813382	-1.99406271551918
H	-0.72884761665007	-5.39753321183258	1.59038918688827
H	0.66637047960534	-5.63143239400707	-0.44285260683968
H	-4.96997586640861	0.27254973721827	-2.77005912346143
H	-3.34418818718356	-0.41334731316685	-2.62828236145206
C	-3.49496762448085	1.30274419981123	-3.88690463119350
O	-4.29722849426380	1.59606608933148	-4.78073671539812
N	-2.19962669747322	1.65781806699878	-3.90850536153095
C	-1.59835400791841	2.46338291073608	-4.95434960570776
H	-1.62734420807154	1.35493318896950	-3.12061243013292
H	-2.38318175811661	3.05389986766089	-5.42682806238033
C	-0.85477134884403	1.61985198122763	-5.98903914280052
H	-0.89604350544512	3.15492714183976	-4.48299292033167
H	-1.56316013781875	0.98704918340073	-6.53078027284347
H	-0.19249614439215	0.94789064606686	-5.44096448707573
C	-0.04745714644096	2.48034539428865	-6.95871740191896
H	-0.70349148577043	2.95561971903184	-7.68829679271172
H	2.20124534187776	-2.57917097013417	-4.72668175107172
H	0.51127826022497	0.99570130290041	-8.41994991665336
H	1.88807492911932	-1.77040724910024	-2.23317117707421
H	4.25326440635072	-1.31240257079523	-3.00590091689691
C	1.01367426726067	1.67698828534934	-7.72715732260822
N	1.83639800079669	0.82521725256053	-6.86970577528324
C	1.87809854142857	2.57255674997052	-8.61397887857612
C	2.88988886959070	-2.74403557789384	-3.88894185244115
O	1.49137486427551	3.61009262443201	-9.09360445772938

O	3.10262738179257	2.11031565625546	-8.89917743951598
C	2.45318412939928	1.34546998683703	-5.76604076619987
C	2.74285946045233	0.32680616097428	-4.65725773753192
O	2.70416890787645	2.53724731678024	-5.67875224301611
C	3.87595103659807	-0.64702460041564	-5.02726719280730
H	1.82741041208591	-0.26538152107449	-4.56907188965686
N	2.89139154642489	0.96386890553683	-3.37250128237355
H	1.55707072103198	-0.14319453811662	-6.82467924400643
H	2.08697685312629	0.95881704169824	-2.74371141921641
C	4.01764259745196	1.48106455010723	-2.82702116401095
O	5.06749295724364	1.64410577749273	-3.46540424936954
N	2.16149241057461	-2.66752836640031	-2.63399038151475
O	1.90044463330871	-4.91196403694270	-2.41380560254299
H	0.45142474558739	3.27866970562994	-6.40920478248476
C	4.06823551544054	-1.75302351572668	-3.98711087373824
H	4.80183580654243	-0.08954299608413	-5.14894939648116
H	3.63391459001227	-1.09843449586630	-5.99245494825275
H	3.24767505285117	-3.76646068733489	-3.98201119635479
H	4.97287818986554	-2.30442281317629	-4.25062663461538
H	3.26174937358931	1.28795250871512	-8.40066083827516

Coordinates for Geometry Optimized Structures for gallium complexes

$[Ga(CAT)_3]^{3-}$

Ga	-0.03451882944743	-0.15766169870114	0.01702234010994
C	-2.07365434848342	1.43697537140595	-0.95194161943916
O	1.38587071749534	1.14015275449575	-0.49490998624442
O	1.22032701005822	-0.54340668869376	1.51768978785528
O	0.72352029777436	-1.53870746472043	-1.20769838765935
O	-1.22361832414391	-1.66317746691592	0.55004791311455
O	-1.21652205095595	0.54752206622608	-1.41750095516446
O	-1.08769159375048	1.09948512508209	1.15000108798223
C	0.14113240077843	-2.71115565585818	-1.04336398289293
C	-0.91956917805518	-2.77923769777836	-0.08580887195530
C	2.43174563003621	1.05978360613634	0.30688658381471
C	2.34165589221314	0.14411660446313	1.40188992303187
C	-2.00835143252984	1.73216418580328	0.44625367829368
C	-2.90040927554581	2.65234338623677	0.98056475296209
C	-3.85028873550488	3.29140006029001	0.17556101932912
C	-1.56447637976446	-3.99134101776363	0.11964236365640
C	-1.19048003464028	-5.13865717275330	-0.59014580956858
C	3.59285634088819	1.80334725148119	0.14507012008279
C	4.66850782153172	1.66774374288722	1.02961233993347
C	4.58163907091641	0.78498511760863	2.08684068045299
C	3.41949998431833	0.02702810158477	2.26928029542760
H	3.65174495402864	2.49420083825387	-0.68910909625460
H	5.40868941944822	0.67399283265106	2.77758892959463
H	5.56397475623487	2.25880921860264	0.88023633722791
H	3.34347021311429	-0.66977075127714	3.09700780451012
C	-3.01961133003456	2.08600652908627	-1.73364774540045
C	-3.90826714175615	3.01162608932793	-1.17495881465899
H	-4.63940064460257	3.50224729125544	-1.80610603877893

H	-3.05888164903177	1.85678956141130	-2.79309024274119
H	-2.84679777423294	2.86807295118466	2.04220453877927
H	-4.53548894078108	4.00457428146760	0.61761287939409
C	0.49807282694911	-3.86130799214234	-1.73488519213257
C	-0.16564927493894	-5.07338692026403	-1.51252133902093
H	-2.36724985247425	-4.03322486734224	0.84784165467653
H	1.30504749433015	-3.80144418955565	-2.45718280641091
H	-1.70794935883033	-6.07332410793038	-0.41090163383534
H	0.13121813588862	-5.95677884344546	-2.06484708677115

$[Ga(Ent)]^{3-}$

Ga	0.53489472376505	1.33081782268361	4.09719251648166
O	-0.34492774978348	0.53836520401540	2.46298765619015
O	0.88849027392249	-0.56758929925017	4.69039193849405
O	-1.26097791323783	1.17654596894243	5.00158150292090
O	0.23491798889565	3.00854917401759	3.09043931071520
O	2.42506473555224	1.19746847129949	3.52957860203009
O	0.98095351426547	2.26701167307548	5.78503227236701
O	-3.27336368221799	-0.30146404540225	-0.42530716264880
O	1.78674030844106	-4.66480462241647	4.46532296820217
O	-4.19930624511726	0.06855794267839	7.79159864696670
O	-4.69519688596060	-3.16530884397324	2.11672882594858
O	-2.11500127566997	-5.33606360270928	3.64993811907505
O	-4.50770726514685	-3.48883572843924	5.68932265119522
N	-1.86939107036285	-1.15009270252253	1.13824659066605
N	0.28892715680207	-3.09738007626346	5.12411764504048
N	-3.52346853543951	-0.01641922230810	5.63050546584847
C	-0.86884164821238	1.46894052183895	1.69062603975457
C	2.03075522010305	-1.02104365157858	4.21404989018782
C	-1.19773236219954	1.51659907041947	6.27351568780642

C	-0.51242637930210	2.82549844085624	2.01144638824352
C	2.89390229952883	-0.03926253865464	3.61282764347582
C	0.02239930882410	2.15399837560844	6.69335348511552
C	-0.96735138863662	3.85401410432767	1.19740524229684
C	4.14225098046101	-0.43018738159125	3.14840310998061
C	0.13601478309980	2.59798776868544	8.00388273364488
C	-1.80400155589437	3.58422347914907	0.09897821750299
C	4.54727837051277	-1.77554605398023	3.22192338466084
C	-0.90731716692053	2.39160580083681	8.92501503773272
C	-2.20781257165836	2.29498843904237	-0.16902312017642
C	3.69755841475020	-2.73141831218787	3.73322223731326
C	-2.05022990967376	1.72297468843178	8.54544420171538
C	-1.76298350735604	1.21650719941185	0.63073887493871
C	2.41956922486503	-2.37570190986539	4.22361749025267
C	-2.21003671116725	1.25817093457875	7.21924835010209
C	-2.35511666965447	-0.11877597270291	0.38885621514505
C	1.50051426602170	-3.46719231518897	4.61675246073979
C	-3.38762502139049	0.41220310068323	6.91864341172009
C	-2.60309380943412	-2.38205450099589	1.21856405006392
C	-0.77648566784246	-4.05850985136425	5.19294900185255
C	-4.40349335623785	-1.11039075667894	5.32816604185800
C	-3.82508051453635	-2.32821440741846	2.14427826140584
C	-1.44087774865765	-4.35270359742204	3.84178370500928
C	-3.82365230388918	-2.49404744942122	5.65169568298607
C	-1.72805148404907	-3.56788834068658	1.59590166693156
C	-1.83343498484484	-3.69613282855201	6.22615726426287
C	-4.90052501026786	-1.08850134341480	3.89028710133432
H	-0.67833050305130	4.87287899897032	1.43156449399051
H	4.79586149701037	0.31816815907695	2.71342074430644
H	1.05037700996751	3.09417786258566	8.31074645607001

H	-2.14443800232280	4.40157017275994	-0.52562210194459
H	5.52720758525646	-2.05830395244084	2.85613634551686
H	-0.79853057648729	2.74752036085557	9.94263530214222
H	-2.88512676432620	2.08376676426339	-0.98473994442918
H	3.98248301782395	-3.77403198742880	3.75601570723455
H	-2.83935290185868	1.52328170266613	9.25695411927912
H	-1.22940679578068	-0.87453401358019	1.88637680947779
H	0.08572826398334	-2.09735674050479	5.06751069798354
H	-2.73642408633747	0.20260585994199	5.01673324496903
H	-3.00947406094745	-2.60682588711493	0.23136045308332
H	-0.35967037077629	-5.01470728545408	5.51180844129211
H	-5.28908540331940	-1.02394420428795	5.95941778637483
H	-2.29921418178533	-4.48996504834170	1.54509118103688
H	-0.87070635962338	-3.61719837510437	0.92761442849383
H	-2.56459395080021	-4.49420788674395	6.31345284580551
H	-1.35495743418071	-3.51478765832381	7.18628957395369
H	-5.64153787433846	-1.86635608940168	3.73266005223143
H	-5.32230711716900	-0.11152678634959	3.66399099007964
O	-2.50330865912127	-2.46536468681384	5.86512523097806
O	-3.79575889645505	-1.28105946114496	2.97565981559975
O	-1.20115361040951	-3.39999564367813	2.93331201912384

$[Ga(PC)]^{3-}$

Ga	-0.61855514477395	0.45056022276394	-1.70593205751373
O	-2.03341592440214	-0.80030544178798	-2.36396249805538
O	-2.14256095919498	1.73722481076139	-1.83980639097463
O	0.61874211614191	-1.18367564384437	-1.86320544659920
O	0.54725878660776	1.78644530504069	-0.81387076662099
O	-0.93342544073818	-0.10422381248792	0.20152320143394
O	0.18999476627990	0.86959809963492	-3.45052702608836

C	-3.24656032232601	-0.27667005688223	-2.34380317652576
C	-3.30025652993576	1.13121771371504	-2.06850990182213
C	-4.43388486424633	-1.00943626359924	-2.54851628455406
C	-4.53828022211064	1.76315045526095	-2.05201297839386
C	-5.66863461145324	-0.32652943123837	-2.51636035815222
C	-5.71809665986336	1.03290490186726	-2.28225659795405
H	-6.57467264944967	-0.89571528462481	-2.67546751531796
H	-6.67241170485073	1.54611032541371	-2.26763863314979
H	-4.58139249703028	2.82884604962776	-1.85393391777936
C	-0.09210255016723	0.48083593133735	1.03481501843748
C	0.67674833714170	1.56503900360382	0.48546275918734
C	0.14008202382700	0.07376716509728	2.36601305427244
C	1.51047816550676	2.29662446668924	1.32113418763261
C	1.00714428382484	0.83920770410256	3.17902648825632
C	1.65770821063273	1.94419548288665	2.67513945348199
H	1.16801205040700	0.51830493568111	4.19934474161422
H	2.06369616847321	3.13231556802692	0.90627696487335
H	2.30824642103484	2.53119225151301	3.31264435402720
C	0.96946420770928	-0.10071943179205	-3.90933943835894
C	1.15488517078933	-1.25494132894265	-3.06402083156633
C	1.59804768093754	-0.05275521376672	-5.14577874204908
C	2.36596705643659	-1.13760776985926	-5.60374578158916
C	2.47235169293175	-2.27963093744833	-4.84181483630449
C	1.84810219894645	-2.37545587732561	-3.57541075258297
H	1.47236887111907	0.83269098937653	-5.75962515746840
H	2.85310569271937	-1.07992214612953	-6.56989909214237
H	3.01089054997005	-3.14184173698284	-5.20884508515412
C	-0.31048953799237	-1.24845170835576	2.87062241594011
C	-4.43892760333266	-2.49235248761563	-2.73456900049038
C	1.84235841428655	-3.71856705077691	-2.93529125973423

N	1.13823878975302	-3.85727600308585	-1.78058430663387
O	2.39944101932017	-4.69394768017290	-3.46580201211916
C	0.68050661185046	-5.16454270634481	-1.34082962228559
H	0.68718004478670	-3.00924584556998	-1.44385305616959
N	-1.30283210332822	-1.86643581839525	2.16873344855683
O	0.24358006744186	-1.79858655195211	3.83504066706470
C	-1.26149938345825	-3.31825100934746	1.97542682015860
H	-1.52592890392365	-1.35834857817225	1.31365605691112
C	-1.75542467349135	-4.19014100117492	3.11999629669305
H	-0.23128105607820	-3.59109950407998	1.75113999597608
H	-1.85628442716351	-3.51954511219059	1.08433633908853
C	-1.73355251887912	-5.68093191095338	2.75571794025958
H	-2.77413676210367	-3.90219324747797	3.39645860305791
H	-1.12419598655624	-4.02104302173944	3.99374069943333
C	-0.39667209485281	-6.20995598635493	2.20899346554303
H	-2.51588616646482	-5.89925775814596	2.02149256735451
H	-1.98124271265829	-6.26052369190249	3.64826389268916
N	-0.24257295293875	-5.98440979316936	0.77453521641698
H	-0.33390524833708	-7.28527709699595	2.39023355904258
H	0.44826907401047	-5.74057817256095	2.70991547417604
C	0.72414717544974	-5.25479282988732	0.19298037965373
H	-0.95260670515903	-6.38858591717318	0.18670976643274
O	1.62922070261695	-4.70707927104242	0.82436839604462
O	-5.48199223748874	-3.16649136507178	-2.67388130172029
N	-3.22498270529951	-3.03445652113367	-2.96759185368321
C	-2.85530606127436	-4.43065402570831	-2.83563401072534
H	-2.46103568561752	-2.36100983063808	-2.87924536483523
C	-1.80001142158227	-4.57302400939312	-1.71798122806285
H	-2.43688118692922	-4.78519855950164	-3.78244019456440
H	-3.74934126216291	-5.01774948105815	-2.63291746093797

C	-0.64937939721837	-5.53225478997830	-2.03391946476720
H	-2.29371815328846	-4.84419376445831	-0.78419308250968
H	-1.37620580197395	-3.58186053095696	-1.55395074438280
H	-0.43969425256484	-5.49601361814121	-3.10543593376752
H	-0.91612073214125	-6.57173418441994	-1.82326920587427
H	1.43152346384902	-5.88200057059079	-1.67850985372918

$[Ga(KO\text{-}NH_2)]^{3-}$

Ga	0.38840142464754	0.66288888776958	0.16828333327805
C	-2.14234276683505	1.24215160272264	-0.73673630038921
O	1.71183696029543	1.45371888214237	-1.12251941429280
O	1.84620443638508	1.10648461579081	1.46259762869600
O	1.10214078715330	-1.09379524329293	-0.54566406455443
O	-0.51110895359257	-0.58275369430456	1.44519630925672
O	-1.09413084459104	0.56736041228542	-1.20258783943474
O	-0.63920572171687	2.25188375304615	0.75956056343406
C	0.41089574542407	-2.11800681398925	-0.08839119432521
C	-0.45540220149150	-1.83892357738377	1.02817400595430
C	2.92736968431960	1.44817293217265	-0.59483165747739
C	2.98838224545000	1.33907747865922	0.83639539800544
C	-1.88807838750733	2.18654703637993	0.30599957733764
C	-2.93792272122110	2.96247453675614	0.79531025110234
C	-4.23827163070703	2.79009622968876	0.30127919378196
C	-1.17771777231881	-2.88034931915792	1.59748121403362
C	-1.09244969026268	-4.18298785283318	1.07419002005862
C	4.12311944047855	1.46685520456739	-1.33871369784921
C	5.35513599463155	1.57817214362565	-0.65666205445947
C	5.40060079044595	1.59618411028320	0.72182515441784
C	4.22039719020805	1.45411873526767	1.47119309323322
H	6.35160881720131	1.68708608141016	1.23344325778545

H	6.26246198173637	1.61030784607400	-1.24376281392034
H	4.25761916862000	1.40241070299790	2.55414536596480
C	-3.45124688431932	1.04376392549984	-1.18667995814752
C	-4.49285289250647	1.83254425646332	-0.66987484035429
H	-5.50242153855469	1.67762181166454	-1.03416985450389
C	-3.72135551507078	-0.00830998470120	-2.23721476829086
H	-2.73479899496003	3.68829998003896	1.57573303759546
H	-5.04702799682374	3.39610706589944	0.69294742167698
C	0.45190756394285	-3.41529327177764	-0.63715625932942
C	-0.30258982876371	-4.44258239807199	-0.02574775366605
H	-1.81955805243986	-2.66877305947055	2.44618926194545
C	1.20024677965046	-3.75085657173493	-1.88255732222700
H	-1.66326723570440	-4.98144382538584	1.53374695168542
H	-0.25466238390715	-5.43455467957123	-0.45315266958280
H	-4.72220046864277	-0.42099787934005	-2.11968997826594
H	-2.99192462297090	-0.81500066423642	-2.13974871616412
C	-3.64115489320132	0.57202492909597	-3.63825160809666
O	-4.64542723205308	0.71043647958328	-4.34539363529830
N	-2.41362158518621	0.95304319047852	-4.04225562428921
C	-2.22068247911264	1.69318582955870	-5.28041790548170
H	-1.66415515325911	0.90663967550947	-3.35970161743042
H	-2.82896833079087	1.21831398548237	-6.05194811338664
C	-0.76658198135686	1.73491930593296	-5.72548803679475
H	-2.60023762653887	2.71560883978352	-5.16681394139367
H	-0.38482561817026	0.71429525762844	-5.78078798116477
H	-0.16391426754240	2.26093028475212	-4.97776710212406
C	-0.65431357369241	2.44096190593433	-7.08152032990911
H	-1.12180931840042	1.81548339137602	-7.84982822305315
C	0.74697418897272	2.84887872532686	-7.54081027065682
H	-1.25247134165146	3.35656081475140	-7.03202274560516

H	1.24417687965823	3.42830836477684	-6.76222042228189
H	0.65076482855155	3.50160716139419	-8.40785091721493
C	1.65559466261922	1.69019797002833	-7.96123449930309
N	2.01987064234054	0.80323342148914	-6.86318973541165
C	2.89192009216618	2.17108751376377	-8.74539946120082
H	1.10444910126635	1.06573197708256	-8.67069079658850
O	2.88877482673663	3.23334324764582	-9.36261481584870
N	3.92694862598241	1.31585358123851	-8.77463393559724
C	2.69522072252694	1.23143828501929	-5.77183002567592
C	2.76332715676435	0.20607942750965	-4.63198159735056
O	3.20355482691323	2.34461986710767	-5.71076864831844
C	3.74866608145111	-0.93301517337927	-4.96381853195179
H	1.76436136201156	-0.23232634958721	-4.56149047921296
N	2.98399502599457	0.83707545475142	-3.35237386960005
H	1.55510693010005	-0.08916776080131	-6.81815341430176
H	2.19086218257604	0.91248243647778	-2.71491795475688
C	4.17021685052697	1.19146506805418	-2.80420749752740
O	5.23537697573342	1.19972962650545	-3.43786922745598
H	3.43386325996259	-1.38132855852716	-5.90914193962570
C	3.81019890063309	-2.01634465823170	-3.88408512676704
H	4.74022279696271	-0.51172105010244	-5.11579562657251
H	4.03116967187803	-1.56058054869913	-2.91752009465463
H	4.65470636176070	-2.66903013669055	-4.11305069750180
C	2.53072453412104	-2.87015597355640	-3.76602352138851
H	1.83078601705079	-2.59493530032524	-4.56459404837408
N	1.87557713639182	-2.74128129297937	-2.47662398921404
H	2.76054899342791	-3.92379954283173	-3.90798715824448
O	1.18192434050336	-4.89935900377140	-2.36370755029999
H	1.79372449304619	-1.83243368001447	-2.02124566377533
H	3.92749376140563	0.47821446801065	-8.21711484071206

H	4.76106226543736	1.56426214769375	-9.28228866009420

[Ga(KO-COOH)]$^{3-}$

Ga	0.38410178804007	0.64791623374340	0.15392266693165
C	-2.14862196827068	1.22858087145550	-0.74147291313326
O	1.70833977297841	1.46081609469253	-1.12526969444734
O	1.83745230193359	1.07979343388150	1.45550699626975
O	1.10551848542634	-1.09517544964338	-0.58022182583325
O	-0.51489594099525	-0.61859943141181	1.41198974151458
O	-1.09687664920085	0.56817169200290	-1.22100929752017
O	-0.64787958122006	2.22540855886276	0.76546562742216
C	0.41658559398996	-2.12877802313814	-0.14040758149313
C	-0.45504982228816	-1.86830052049642	0.97637359296131
C	2.92208652132979	1.45679946637005	-0.59407840164000
C	2.97978566683024	1.32747205139474	0.83575708451894
C	-1.89784573229884	2.15817452331958	0.31499355157037
C	-2.95204849545280	2.91780570240864	0.82039491858199
C	-4.25271142135127	2.74294514456177	0.32836816389357
C	-1.17896162094706	-2.91930640606687	1.52578836011545
C	-1.08815082959992	-4.21381250260478	0.98354696486612
C	4.12015169046951	1.49709469402013	-1.33370883156599
C	5.34937956110979	1.60691530623128	-0.64597863842551
C	5.39047854853476	1.60298794418681	0.73253960261924
C	4.20904681634382	1.44085708008852	1.47594002940520
H	6.33916076180302	1.69248219725237	1.24869096874117
H	6.25809576453718	1.65552039228533	-1.22973188490378
H	4.24326529298376	1.37311081895715	2.55809101102230
C	-3.45741578194603	1.02743788865944	-1.19041252120870
C	-4.50334689034759	1.79931010847559	-0.65734690678020
H	-5.51292940016568	1.64239307268899	-1.02070777229598

C	-3.72220817350492	-0.00823886090240	-2.25830384965900
H	-2.75190912769461	3.63226845952210	1.61197895961520
H	-5.06480271546902	3.33604872575349	0.73267702897470
C	0.46489596731773	-3.41807184309106	-0.70698930887841
C	-0.29086819112486	-4.45573702377208	-0.11508080214618
H	-1.82595166502395	-2.72185802641365	2.37396648660480
C	1.22435196732207	-3.73417422358622	-1.95075696841512
H	-1.66037017135018	-5.02004757163836	1.42750606436495
H	-0.23787048026051	-5.44144686046754	-0.55616535677315
H	-4.72258223429197	-0.42471619437739	-2.15098035594653
H	-2.99128967486668	-0.81485579806066	-2.17212111345172
C	-3.63855228606520	0.59764476981180	-3.64820472785579
O	-4.64161661906520	0.75330434734222	-4.35338127609324
N	-2.40913127362769	0.98265805424530	-4.04297243028636
C	-2.21394631798402	1.74880838263966	-5.26455886817186
H	-1.66162317819423	0.92308476338940	-3.35884720495250
H	-2.82480031564931	1.29353758892154	-6.04576657473938
C	-0.76027045540477	1.79446371882638	-5.71117172848508
H	-2.58865226584944	2.77030216118914	-5.12873620516684
H	-0.38240212456843	0.77338720743076	-5.78527801278709
H	-0.15448562895946	2.30577140168687	-4.95582658518320
C	-0.65183541410798	2.52450673405755	-7.05453976264566
H	-1.12047823828948	1.91378601326874	-7.83354106268250
C	0.74808541363637	2.94157941127773	-7.50771113005124
H	-1.24828023737725	3.43973852061577	-6.98728890745494
H	1.24646581687402	3.50910824866160	-6.72137326487153
H	0.65028509633254	3.60702768255190	-8.36538703776578
C	1.65611165211852	1.79009218577158	-7.94779166959813
N	2.03334205870595	0.87147673029770	-6.88086926753438
C	2.89670026700257	2.28349964892972	-8.70117107429783

H	1.11552317230625	1.18380125677679	-8.68175711893398
O	2.95803766905236	3.33826910276459	-9.28312137295805
O	3.92568143602780	1.42582995614168	-8.75609652313888
C	2.71791315555410	1.29400081229514	-5.78427436278556
C	2.78121284209872	0.27108111914103	-4.64590539012157
O	3.24194935692878	2.39830995532222	-5.74151582256978
C	3.77644193995611	-0.86038667353604	-4.97892990748235
H	1.78459037656540	-0.17366505855433	-4.58371756435287
N	2.99005696501915	0.90116458179024	-3.36397377743489
H	1.53245682671096	-0.00132298170987	-6.82716245877732
H	2.19464380469966	0.95804991797418	-2.72683428420741
C	4.17424365916750	1.24664779701897	-2.80276974619498
O	5.24135702020539	1.26725613338328	-3.43220013462226
H	3.47131341669859	-1.30313635452110	-5.92999732025890
C	3.83550820046878	-1.95056434677000	-3.90631244765085
H	4.76617796221996	-0.43125202945139	-5.11946790756694
H	4.03876546700550	-1.49950737293870	-2.93371441653828
H	4.69037037457528	-2.59179654021517	-4.12878511427876
C	2.56373632486174	-2.81851934477310	-3.81193985909148
H	1.86824225401373	-2.53884793723264	-4.61286994486801
N	1.89681964343461	-2.71243909749001	-2.52675979838506
H	2.80508880610865	-3.86789467387541	-3.96636233453276
O	1.21784317008322	-4.87683587595520	-2.44586688394446
H	1.80581098649518	-1.81026625616651	-2.05969146635887
H	3.73125025893573	0.64263332062200	-8.21128883999740

[Ga(OK-NH_2)]$^{3-}$

Ga	0.21611720284981	-0.05005161044776	0.24378035233339
C	-2.06764578822488	0.83336858075649	-1.07500322517117
O	1.42812853975915	0.98764694911755	-0.97429755803123

O	1.66176350900563	0.43097992928901	1.56202265956057
O	1.09560402089530	-1.73111686605258	-0.48105826172038
O	-0.58392593975800	-1.36030268316088	1.49587824131753
O	-1.14395720026354	-0.10479430975448	-1.23206244398864
O	-0.93095768436387	1.41928162131034	0.90491871852155
C	0.78825674583758	-2.80065004961489	0.22795327786805
C	-0.11809088232575	-2.58909586709339	1.32708769167775
C	2.61087668689163	1.22888746318835	-0.43611920292330
C	2.71568822519155	0.97205994456677	0.97407761199910
C	-1.96864037068991	1.64308226537350	0.10800117321544
C	-2.93756801266288	2.61087541900197	0.36081898796517
C	-3.99594085342342	2.81255316544240	-0.53734116757145
C	-0.45997551093738	-3.65958600272921	2.14363417475971
C	0.05327594673650	-4.94405811377231	1.89501189145986
C	3.73980117060571	1.66138181766880	-1.16068424029157
C	4.92684940489099	1.96735754926711	-0.45813209662857
C	5.00048538107234	1.79979062648635	0.90894125845413
C	3.90336058309966	1.28911115625241	1.62507336094262
H	5.91589094336223	2.04107336071311	1.43658183030011
H	5.78352139464150	2.30722554304774	-1.02352324830302
H	3.97629539557536	1.11764435842309	2.69377316234982
C	-3.10363406104966	1.07710236969817	-1.98705412700641
C	-4.07084442921250	2.05886357236713	-1.69532456682802
H	-4.87185982760604	2.23187635873353	-2.40627977787526
C	-3.16771316452671	0.33750575608241	-3.29976694820597
H	-2.85660541189004	3.20818646705859	1.26293506773418
H	-4.74696785886736	3.56454493415268	-0.32504258693076
C	1.27441448578025	-4.09890686403374	-0.02917798038272
C	0.89869778238160	-5.15836926753249	0.82806574480185
H	-1.13593672173319	-3.48660869024309	2.97431197724630

C	2.12525899851154	-4.43986054003640	-1.20250383685648
H	-0.22516639255703	-5.76696589863704	2.54284600214435
H	1.28331716906867	-6.14574601371025	0.61454204619787
H	-4.11695976805917	-0.18889124976762	-3.42307281317385
H	-2.37888158711992	-0.41710350428385	-3.33673891692901
C	-3.01500394242904	1.20533873090373	-4.55065305892595
O	-3.51144509035386	0.86456133423664	-5.62949340066585
N	-2.29929230268592	2.33156093397625	-4.39179204029092
C	-2.12973165793772	3.38229601614822	-5.38673946111165
H	-1.99182435010383	2.53292081284411	-3.45168612121874
H	-2.92749534241455	3.28073445746633	-6.12347002568398
C	-0.75764703919864	3.36479239583501	-6.06455980650744
H	-2.27129041091659	4.33463806709081	-4.87273725633854
H	-0.00489648266552	3.17414589541101	-5.30081146780036
H	-0.54863944677784	4.35631095228689	-6.46837020949118
C	-0.67322867146080	2.33127500037065	-7.18738948200944
H	1.86685279091299	-2.15664952172434	-4.27481167833096
H	2.51999679551376	-4.59987570850564	-3.80076543816228
H	0.57616342328646	1.25065544442620	-8.51608863375879
H	2.01661954328881	-2.53422287037418	-1.85584354037612
H	4.06712304467252	-3.99464041880148	-3.24365444792339
C	0.72510933257967	1.94822062219070	-7.68612787056331
N	1.48087994041200	1.19070403580369	-6.69257974814567
C	1.53028983517733	3.09193008102543	-8.31518496698153
C	3.01275513217047	-3.72489537023493	-3.36950974068233
O	1.00536543280290	4.15962636571329	-8.61728620614044
N	2.80424060883252	2.79098853301398	-8.62601379304932
C	2.14557760569935	1.71513580279877	-5.63452931915152
C	2.51911881384778	0.66320427281700	-4.58018519863488
O	2.40419722927458	2.90736877362724	-5.52623342234044

C	3.70471423791522	-0.21953455162036	-5.01003371616723
H	1.64294536418573	0.01464249452881	-4.50783379630610
N	2.65360931998450	1.23077499755000	-3.25647891654222
H	1.25255671796034	0.20926289470555	-6.64803885653821
H	1.90724024030717	1.01132477008361	-2.59278435427223
C	3.78736933054322	1.66640245140270	-2.65221222788191
O	4.81279608545861	1.98193340610522	-3.27447059510496
N	2.40446852055341	-3.44923644357929	-2.07651113546346
O	2.53520461780345	-5.59944196748719	-1.39898869769347
C	3.90188942636857	-1.42039907207664	-4.07856718978140
H	4.60431442370640	0.39062825325091	-5.03865365337755
H	3.51660488854426	-0.57576537979836	-6.02599580952312
H	4.90764704280522	-1.82265185308030	-4.22842544331908
C	2.89035010483459	-2.54104710020905	-4.32377288851949
H	3.85488228929431	-1.09004330793040	-3.03733807647793
H	3.02928826442279	-2.91330055423933	-5.34254190683122
H	-1.14139412376809	1.40070108328664	-6.85782839861810
H	-1.25473608362639	2.68278465840230	-8.04304615257966
H	3.39598471083704	3.50651728318456	-9.01763745516280
H	3.23632373265663	1.94971963054746	-8.28117668838456

[Ga(OK-COOH)]$^{3-}$

Ga	0.19464818030381	-0.03587562804497	0.22566736538358
C	-2.08408581899988	0.84046570017719	-1.10495816432037
O	1.41268103797788	1.01395243273687	-0.97696832191877
O	1.62611691721175	0.44513473657595	1.55927660489294
O	1.09009838537900	-1.70771996501556	-0.49852778717228
O	-0.61153835565474	-1.35726097333114	1.46308943694135
O	-1.15109919453338	-0.08856416310069	-1.26138965135395
O	-0.96537670756330	1.42462938156054	0.88556640002781

C	0.78049415953809	-2.78309821805494	0.20082140160092
C	-0.13781560775574	-2.58263144294633	1.29190445747877
C	2.58584207752270	1.26743220855174	-0.42426191868260
C	2.67877711715523	1.00373295058342	0.98569893693227
C	-1.99969303961532	1.64425612575120	0.08317615843380
C	-2.97828435864745	2.60267399894177	0.33460892227795
C	-4.03232340925617	2.80031243740681	-0.56955150732682
C	-0.48169924731781	-3.65960585255626	2.09916585460463
C	0.04047145117295	-4.93995079756206	1.84802037701432
C	3.71690124127801	1.71965911835955	-1.13348433732368
C	4.89183912255610	2.03815719750265	-0.41612160048239
C	4.95257606375969	1.86362884798087	0.95059542465365
C	3.85482184209977	1.33323889003664	1.65135010345138
H	5.85850765362926	2.11472953031418	1.48984024667892
H	5.74972863991153	2.39362805847222	-0.96994038773899
H	3.91816565391824	1.15678869680458	2.71984430639322
C	-3.11592136054683	1.08040608326544	-2.02263873871302
C	-4.09336253376418	2.05236742861655	-1.73202904163053
H	-4.89093841584544	2.22271703511349	-2.44749314478334
C	-3.16276529449776	0.35096197511394	-3.34159182640636
H	-2.90843186473962	3.19583885627163	1.24036951489075
H	-4.79100343649603	3.54483200807410	-0.35809869242865
C	1.27526278778448	-4.07742459867788	-0.05951226387204
C	0.89692631436183	-5.14385026273154	0.78782685383884
H	-1.16652499502986	-3.49495397161887	2.92424766731842
C	2.13833443721826	-4.40651080249997	-1.22708272951117
H	-0.23988095420008	-5.76798199452859	2.48845810311096
H	1.28845245022835	-6.12801600357197	0.57198994028176
H	-4.11241465860450	-0.17003520390629	-3.48326985667645
H	-2.37673047509196	-0.40673006425369	-3.37339848833462

C	-2.98714833631934	1.22797815176038	-4.58319411355564
O	-3.45778863013636	0.89266236054813	-5.67499362773149
N	-2.28099784474516	2.35687034543336	-4.40082554035974
C	-2.10009376789695	3.41683302727284	-5.38335151291636
H	-1.99454730638267	2.55270650821649	-3.45293053988562
H	-2.88390354420981	3.31755915250176	-6.13511375408739
C	-0.71575932664561	3.41313170900831	-6.03659038507207
H	-2.25508151691765	4.36397867332377	-4.86399399407272
H	0.02246824036355	3.20906872517799	-5.26244197985479
H	-0.50178247454027	4.41257282738297	-6.41813420588600
C	-0.61261322235499	2.40021716724055	-7.17659055355272
H	1.89915694956198	-2.10127617457276	-4.28905852621204
H	2.55423468026535	-4.54446568442477	-3.82715643466539
H	0.66358866036593	1.38696785942472	-8.54036856929493
H	2.02119612930696	-2.49817640889741	-1.87065604385853
H	4.09333008765468	-3.94075665755826	-3.24638788638337
C	0.79349075948499	2.04309322931592	-7.67278933213913
N	1.55720073884393	1.25606264448729	-6.71142892113533
C	1.60153943117711	3.20834067294870	-8.23535312960596
C	3.03985899002926	-3.67237744910151	-3.38195186242277
O	1.12721043732229	4.27560440220738	-8.53489164487225
O	2.88920107612552	2.94658536325924	-8.51487878447246
C	2.19292362708735	1.77639573539640	-5.62788509469711
C	2.54804352610012	0.72610609114982	-4.57003490909654
O	2.43718862007129	2.97051047613864	-5.52126026293819
C	3.74421773901497	-0.15199809698086	-4.98303082666545
H	1.67172956496790	0.07575610102322	-4.51662251643044
N	2.65732350766887	1.29226023705059	-3.24407063762545
H	1.32621576691930	0.27415366507949	-6.68780873214665
H	1.90458497055113	1.06269157653978	-2.59072748502779

C	3.78131168019355	1.73294401371421	-2.62368796848104
O	4.81033576296388	2.05907564167569	-3.23376479800704
N	2.41648306346473	-3.40945619341146	-2.09384261825628
O	2.55916323489004	-5.56184325836850	-1.42572710237846
H	3.13560500873829	2.06427479239370	-8.19080208338665
C	3.92735007235310	-1.35692035439198	-4.05435077161034
H	4.64174081355446	0.46170848062933	-4.99113649945077
H	3.57671691258592	-0.50381158674100	-6.00407530614492
H	4.93742918154786	-1.75399468746205	-4.18779474724797
C	2.92535086734141	-2.47994344877304	-4.32619573833774
H	3.85962690511991	-1.03277725674061	-3.01231558487732
H	3.08194236059691	-2.84134274396020	-5.34629687232868
H	-1.07108573310541	1.45759186506416	-6.86929535474862
H	-1.19092472922559	2.76024729200881	-8.03090189270822

$[Ga(KK\text{-}NH_2)]^{3-}$

Ga	0.26752806807384	0.42531030896555	0.05354111809268
C	-2.08257943153616	1.41302743366822	-1.04936348347759
O	1.60655030231969	1.16972178412389	-1.23766006374526
O	1.77460439148334	0.73368821091377	1.33409901743645
O	0.83485747231929	-1.39321722441930	-0.61903378985135
O	-0.70132705595278	-0.68245629704753	1.38866840516468
O	-1.21621473113141	0.43391376838371	-1.31178646860838
O	-0.56182770393444	2.14228193915755	0.59094908357581
C	0.27179817271223	-2.37453936309926	0.05933389037255
C	-0.55209583963182	-1.98143276887047	1.17421060258461
C	2.79854716021785	1.34878303394427	-0.69148497338972
C	2.87207309970569	1.15653792583553	0.73086324006979
C	-1.70859715173422	2.35725379554486	-0.04398955103099
C	-2.55487363262198	3.43232436523198	0.22941649539059

C	-3.77649060371152	3.56398407870972	-0.44215799841236
C	-1.14442036178250	-2.96533006861485	1.95666367224190
C	-0.95898766225461	-4.32721535677038	1.66209451547334
C	3.96514030010015	1.66831994961897	-1.41476341567963
C	5.16510362118891	1.91508464234645	-0.71158642141876
C	5.21429688154089	1.80251907617390	0.66195998676590
C	4.07356222400847	1.41113225785002	1.38379189502760
H	6.14038353829276	1.99630911294286	1.19043892983700
H	6.04949909954748	2.16502083062070	-1.28123085737383
H	4.11922130801263	1.28539269379203	2.46031938549108
C	-3.32847684732601	1.52339284091791	-1.67622916875473
C	-4.16338118987463	2.61235748004715	-1.37459700390493
H	-5.12547660836908	2.68927252694780	-1.86871021347924
C	-3.79417834298229	0.44360230092336	-2.62420638701912
H	-2.25635663057541	4.15394806136225	0.98245057022864
H	-4.42685046664862	4.40150224413949	-0.21784956172626
C	0.41931428356985	-3.74162189437960	-0.25092143600768
C	-0.19689985583706	-4.70665125545916	0.57778182403204
H	-1.75716713033995	-2.66204469897558	2.79899558231803
C	1.13852890154589	-4.22990266305600	-1.46046034846007
H	-1.42661738681021	-5.07840334919566	2.28782295970873
H	-0.06759776355419	-5.75074190304973	0.32877828028547
H	-4.87855307879531	0.47311953462748	-2.71606309936850
H	-3.50981858218433	-0.53402291851772	-2.22315938272431
C	-3.23580232198925	0.53977251993838	-4.03718484624366
O	-3.97303206542454	0.72678399956521	-5.01313910511266
N	-1.90537742183178	0.37913152638729	-4.14745405017349
C	-1.23955262121766	0.36515745945626	-5.43827850961695
H	-1.37668627805439	0.28177897448955	-3.28144233599692
H	-0.40372583633696	-0.33451409802980	-5.37943665508154

C	-0.73781064781962	1.73004678993146	-5.91348802359661
H	-1.94796598151417	-0.03269093278785	-6.16580700279189
H	0.12220529258285	2.02909053755983	-5.31042678150532
H	-1.51226808793853	2.48085523617940	-5.73626733284150
C	-0.37661778218691	1.70730290775750	-7.40481432515879
H	0.07421893725830	0.74000927206005	-7.64521702631761
C	0.56717015078776	2.84182934259094	-7.85042965053384
H	-1.30341584327076	1.75049620241129	-7.98103126600295
H	0.62982436419316	3.60658146865666	-7.07593497437685
H	0.18309180224844	3.33367809927288	-8.74275798777158
C	1.97981562288726	2.33672265638888	-8.18371609439056
N	2.53121895727328	1.44992679123228	-7.16351883919807
C	2.94140218439107	3.47306541307834	-8.55709347858421
H	1.91006448812317	1.72122074372024	-9.08513826478007
O	2.52377348025131	4.54898025627234	-8.97856409188613
N	4.24966319820538	3.17841055154928	-8.47708677332377
C	2.71978122011897	1.85715932737311	-5.88386208472431
C	2.76986725778263	0.72363682395110	-4.85129947333840
O	2.75136561170527	3.04262005932655	-5.58025586169338
C	3.76783656559946	-0.41245962152098	-5.15143018714089
H	1.76696145027449	0.29333457997348	-4.90763172082105
N	2.88130337165422	1.22308565084422	-3.50522432114963
H	2.42298380817823	0.46297635533940	-7.33173609230101
H	2.10227843447920	1.06513532167554	-2.86494697919261
C	4.02486240872108	1.61977480005201	-2.90177280127759
O	5.06640124681585	1.86008566537662	-3.53288996434381
N	1.63355574074010	-3.29926838271052	-2.30434276550041
O	1.24783737943545	-5.44472476348431	-1.71352966700816
C	3.58384462446302	-1.61279055988131	-4.21575409980029
H	4.77801331521705	-0.01767134446668	-5.06010889501404

H	3.64364055278961	-0.74857099552586	-6.18309632795096
H	4.45367067010602	-2.26782454436703	-4.31902863137195
C	2.32608337075048	-2.43077354118846	-4.51391658704925
H	3.57716990452082	-1.27053695222806	-3.17740425948684
H	2.37773645898276	-2.77853829929462	-5.54965728580072
H	1.43009498618034	-1.80658505089930	-4.44348704594425
C	2.13899901562007	-3.65418003497745	-3.62135134714678
H	3.08629884583386	-4.19661275908993	-3.52913878606989
H	1.43205686601318	-4.34537862056219	-4.08784274198358
H	1.48297007670636	-2.32850687612891	-2.03638923542367
H	4.93068221683814	3.88507580743173	-8.70536624156273
H	4.56209750190520	2.32182498826713	-8.05103676658194

[Ga(KK-COOH)]$^{3-}$

Ga	0.27058157566668	0.39432028520308	0.06652108337757
C	-2.08506659552339	1.38510615807173	-1.02687529110678
O	1.60375238056272	1.18117359926556	-1.20807759896889
O	1.77123775702468	0.69132293579984	1.35492617870030
O	0.85342893625875	-1.40361595848224	-0.64272523755633
O	-0.69545579889178	-0.74854644338924	1.37360199936556
O	-1.21162889604378	0.41604813066652	-1.30247173817063
O	-0.57444824418621	2.09464869373008	0.63174599134454
C	0.27427358966870	-2.40281515807077	-0.00451805924783
C	-0.55799629360782	-2.04067673062577	1.11415879173732
C	2.78697550446667	1.38156557144588	-0.65122279017241
C	2.85913549802591	1.15817104486516	0.76673212214154
C	-1.72042311964180	2.31365721463266	-0.00340439212803
C	-2.57441168416563	3.37819556929749	0.28650027282182
C	-3.79402747320765	3.51508328379204	-0.38775202625745
C	-1.16910929852406	-3.04564908507609	1.85425377859608

C	-0.99260314706496	-4.39810412092685	1.51409580364897
C	3.94789412637692	1.75140801486341	-1.36005855217415
C	5.13755945737001	2.01363643521088	-0.64471521940604
C	5.18343320051458	1.86882182676547	0.72576649776700
C	4.05024541967344	1.42921141370984	1.43158381785251
H	6.10121298204096	2.07479754957988	1.26397950211480
H	6.01708787847525	2.30204441812123	-1.20356034697263
H	4.09397481106831	1.27898225131854	2.50504109372464
C	-3.32887127647428	1.49992622485095	-1.65710438950578
C	-4.17135869941792	2.57838818080587	-1.33892939215873
H	-5.13182254487760	2.65905522852371	-1.83562300675132
C	-3.78648658810078	0.43588925128570	-2.62665062692847
H	-2.28334345194343	4.08795673844700	1.05358815175930
H	-4.45046568904271	4.34435042316497	-0.15085159192024
C	0.41543754231215	-3.75944606545467	-0.35955692617861
C	-0.21981796249723	-4.74690574075239	0.42696218456373
H	-1.78935626363960	-2.76645415129503	2.69940449911946
C	1.14943474617832	-4.21205025430073	-1.57424443391189
H	-1.47570726326729	-5.16604179654216	2.10685093613568
H	-0.09647857005354	-5.78272024946338	0.14264999619434
H	-4.87018617138492	0.46655641724206	-2.72565883897029
H	-3.50501711529173	-0.54858058944969	-2.24033747181049
C	-3.21846859025028	0.55478142920899	-4.03406577763377
O	-3.94960945346446	0.74958529736845	-5.01301412831481
N	-1.88623625327090	0.40434536847082	-4.13661862110048
C	-1.21245805287657	0.40587131078225	-5.42311664227035
H	-1.36370697321180	0.29561940784211	-3.26801219371751
H	-0.36774484074448	-0.28280711176903	-5.36262196057099
C	-0.72619382362583	1.78046259337108	-5.88564353638192
H	-1.91039677352746	0.00320651934836	-6.15818311878963

H	0.12261297114788	2.08910053439030	-5.27121177269416
H	-1.51367282981327	2.51863627574599	-5.71348622108913
C	-0.34801992601569	1.77134963953168	-7.37250342811141
H	0.13091358244927	0.81737797706433	-7.61128272816507
C	0.56834387169212	2.93422779444158	-7.79979293741815
H	-1.26789238768652	1.79291763995724	-7.96042629285577
H	0.60969546812384	3.69032261384612	-7.01564406718265
H	0.17802754932252	3.42748193287906	-8.68896938865089
C	1.99501200153362	2.47115545474404	-8.13211392405759
N	2.58231649252846	1.57942820776915	-7.13712704204434
C	2.92688104914177	3.63700291888909	-8.45539794854573
H	1.95785444578260	1.88473109719706	-9.05546973890906
O	2.54300048133707	4.71397302418745	-8.84058767032708
O	4.24035350481732	3.38195563246310	-8.36956642893429
C	2.75029235391984	1.98324645193452	-5.84595691924370
C	2.79703194151252	0.84957722500092	-4.81826840427917
O	2.77181163645429	3.16921839828109	-5.55181689058142
C	3.81438011083945	-0.27233621303388	-5.11001796365664
H	1.80055025472289	0.40716419302073	-4.89489210830980
N	2.88080719519720	1.34333838146843	-3.46829990854504
H	2.47235141120508	0.59329763522264	-7.31256110212803
H	2.10410828912268	1.14744090657511	-2.83480455596672
C	4.01624991699298	1.74061044436564	-2.84665993446208
O	5.05660290390712	2.01157563404063	-3.46648351092684
N	1.65592158315526	-3.25607303604279	-2.38180554096577
O	1.26119207397221	-5.41872133857352	-1.86247701823471
H	4.37768027175293	2.48953629113651	-8.00575051012219
C	3.61567282019076	-1.48955074273032	-4.19943132874860
H	4.81688191522615	0.13176055103407	-4.98311298350218
H	3.72352960567801	-0.59246504200363	-6.15017484822221

H	4.49702595254728	-2.13145614221703	-4.28540848603537
C	2.37908544804639	-2.31776695835494	-4.55203224915767
H	3.57147480106310	-1.16484489008744	-3.15639490268755
H	2.46246165775192	-2.63602482636128	-5.59515045400907
H	1.47186194337842	-1.70955001422107	-4.48685340053345
C	2.18867520586103	-3.56828245894886	-3.69847866023227
H	3.14012680601112	-4.10346856954230	-3.60512462377673
H	1.49747661353500	-4.25245893865998	-4.19744649539718
H	1.50810660333091	-2.29425217146308	-2.08177598197792

$[Ga(OO\text{-}NH_2)]^{3-}$

Ga	0.37929414445953	0.75426754524874	0.10201406758167
C	-2.12781378759150	1.45889192524489	-0.83299207355046
O	1.69853212732815	1.62365637979229	-1.10843563421868
O	1.82756224073284	1.13757319714777	1.45469318043266
O	1.12736057748928	-0.95997603218347	-0.71267324368456
O	-0.51568064623350	-0.55596375398165	1.29100462410867
O	-1.11491185603824	0.71734476456564	-1.27446106735309
O	-0.61497118114135	2.34006920586004	0.73696937356464
C	0.45683494492986	-2.01300803925608	-0.29501214718326
C	-0.43491294913943	-1.79177570624395	0.81735858154099
C	2.91099902919862	1.59957328087684	-0.57137883263378
C	2.96838453132884	1.42164635942659	0.84970885146558
C	-1.84373641841882	2.37322105156905	0.23024851639471
C	-2.84594621725599	3.22748729095241	0.68754496538298
C	-4.13764120510728	3.15906018832706	0.14933321247325
C	-1.15677251811667	-2.86102318199172	1.33070335760980
C	-1.03989614357397	-4.14330006766280	0.76572894107132
C	4.10193302851692	1.64553474274002	-1.31625140974128
C	5.33593677840347	1.74120693107092	-0.63882424196083

C	5.38047034616051	1.70390856833972	0.74144522487414
C	4.20349050971592	1.51875565619595	1.48652348202624
H	6.33147174661144	1.77825599149723	1.25577860470467
H	6.24381368765217	1.79641861789571	-1.22438684922721
H	4.24647182705969	1.41562136280352	2.56560572182355
C	-3.43791640451429	1.35308194284445	-1.31419769678308
C	-4.43157416352952	2.22032870782580	-0.82807757926531
H	-5.44077077736224	2.13225810167381	-1.21537560782088
C	-3.79282914009400	0.27897855201080	-2.31362828493392
H	-2.61331584664947	3.93013631960986	1.48087445222233
H	-4.91032004381904	3.82496778178814	0.51578649014826
C	0.54616781563875	-3.29626278366280	-0.87100963370722
C	-0.21221598644598	-4.35333941591155	-0.31568171702751
H	-1.81974080236350	-2.68742826658461	2.17169507754533
C	1.37946709285793	-3.61152295577151	-2.06489558878822
H	-1.61170622560895	-4.96510882439192	1.18063251428368
H	-0.12818805504653	-5.33187368946524	-0.76711851929369
H	-4.82739525079266	-0.03266054042246	-2.17671120037079
H	-3.14788151548694	-0.58914863976153	-2.15236417019465
C	-3.65908144154883	0.68728941353975	-3.77124145284749
O	-4.62189270080013	0.66602340402423	-4.54686161107696
N	-2.43065129726439	1.05534533352657	-4.17357854973542
C	-2.14784603630728	1.32615400005744	-5.57736083706230
H	-1.67391096714569	0.99663770034314	-3.50155906768144
H	-2.47348625105639	0.46594126179403	-6.17049500683759
C	-0.66701444789078	1.58296374973038	-5.81305114584860
H	-2.74219093482742	2.17993834965228	-5.91316613315471
H	-0.10214552098782	0.84212751451811	-5.24308357447574
H	-0.38800869774273	2.56527623838530	-5.43108940503054
C	-0.32413157434737	1.45126042040709	-7.29584437531451

H	-0.62774721793413	0.45388028792033	-7.62555158270546
H	2.31919783604400	-2.87306309082288	-4.67892310781237
H	1.28030060420904	1.17380469988137	-8.66815878595684
H	2.04971694203870	-1.70404681601251	-2.16389957405114
H	4.38975283573947	-1.43959342749648	-3.01193769526811
C	1.15463471577685	1.61782170736101	-7.67706573207211
N	2.05302434468628	0.87419750829362	-6.79524107042687
C	1.59674290634397	3.07762698667305	-7.86327427553652
C	2.94974640183448	-2.86113209998773	-3.78108616973210
O	0.79256029335571	4.00362467574027	-7.85846564050073
N	2.89846683844579	3.23875649115072	-8.17033054091701
C	2.38026266762330	1.32272107115619	-5.55609091730522
C	2.75633790887845	0.23081940311916	-4.54107107283805
O	2.29368214779636	2.50206245484703	-5.24278503780019
C	3.84558147864191	-0.75108150550830	-4.99360943415976
H	1.83114128052837	-0.34646149276269	-4.43662619373130
N	2.99041118096042	0.79054489589619	-3.23871641221832
H	2.06738477020652	-0.12331059875062	-6.94458844301267
H	2.20138528689095	0.85140621814441	-2.59645325445558
C	4.11943411747436	1.37806382767643	-2.78395078945565
O	5.12020924630531	1.57033760310591	-3.48973951081501
N	2.12827964356510	-2.61920034027658	-2.60115622641500
O	1.38700903318309	-4.75366320791722	-2.56236899252551
H	-0.90130773033387	2.16796642798600	-7.88251493606620
C	4.10488231335432	-1.86655939497982	-3.97557389998357
H	4.76896922164515	-0.20537192436419	-5.17700442275960
H	3.54098555142830	-1.20726174481913	-5.93804638974848
H	3.35190821104699	-3.86861979378927	-3.68368176767128
H	4.97130778750689	-2.42894014265353	-4.32902929976586
H	3.26850210742358	4.17375899166662	-8.24034313070609

H 3.55093432489887 2.48397593972828 -8.03258522263723

[Ga(OO-COOH)]$^{3-}$

Ga	0.37233742145509	0.75061003128579	0.09765417840558
C	-2.13821497787158	1.45491707534158	-0.82271262595726
O	1.69875950501616	1.64738151403155	-1.08130959844461
O	1.80975417764181	1.10471882088674	1.47097858080316
O	1.12732321684693	-0.94566186709875	-0.75372011373574
O	-0.52854199112120	-0.58613337683465	1.24847717271286
O	-1.11467615005070	0.74367477016587	-1.28992942911961
O	-0.63130419885347	2.31908487313903	0.76176795112124
C	0.45471580858541	-2.00788025305342	-0.36306670641326
C	-0.44445215418340	-1.81109108143947	0.74808808072613
C	2.90766507515345	1.60831965049677	-0.53649203785612
C	2.95535787428140	1.39891565043089	0.88038290920633
C	-1.86350676970861	2.34528627191298	0.26261460106307
C	-2.87841214729432	3.16782024706234	0.74943268601434
C	-4.17217072290334	3.08944171657541	0.21775573205369
C	-1.17093135596508	-2.89040112690474	1.23293761880755
C	-1.05149072650993	-4.15999279969961	0.64077480540554
C	4.10309946627351	1.66630307079917	-1.27280977785710
C	5.33352150558956	1.74370076529886	-0.58670571696186
C	5.36921535596440	1.67594412554512	0.79272930020585
C	4.18714542132757	1.47812015362169	1.52639387626904
H	6.31722257040836	1.73560044019495	1.31441025578817
H	6.24519119969061	1.80865304094521	-1.16543704050710
H	4.22353315508088	1.35065926785840	2.60311172959846
C	-3.44927430686365	1.33638171117946	-1.29789113331975
C	-4.45565491367982	2.17120094301499	-0.78217341561834
H	-5.46579884410230	2.07401337406263	-1.16472435410998

C	-3.79067550705301	0.27968020003497	-2.32031563142602
H	-2.65369074265799	3.85285034767080	1.56025254152523
H	-4.95464624051967	3.73059512852919	0.60686101924043
C	0.54804975436684	-3.27905483256544	-0.96508008342122
C	-0.21574033359889	-4.34702572920556	-0.43855923212335
H	-1.83964683163708	-2.73425437717780	2.07278587942459
C	1.39167374178896	-3.57159174936986	-2.15748684214917
H	-1.62722996684085	-4.99006890989273	1.03318272551293
H	-0.12896368871333	-5.31566373261287	-0.91023291325547
H	-4.82574407334394	-0.03686937623094	-2.20005316828038
H	-3.14556818998673	-0.59011484256958	-2.16729207022848
C	-3.64080645546586	0.71191385793154	-3.76933295987091
O	-4.59140604854792	0.68956420445992	-4.55984543408687
N	-2.41152749462588	1.10217418389301	-4.14744002129741
C	-2.11728672599149	1.40463925350913	-5.54165523881705
H	-1.66386161791170	1.04354924102433	-3.46431610475780
H	-2.41905981926140	0.55033511098730	-6.15565614313913
C	-0.63947018284445	1.69459393191097	-5.75484780679921
H	-2.72310182149875	2.25379781253464	-5.86868156791212
H	-0.06618096408741	0.94275055096349	-5.20846368549519
H	-0.38005133585874	2.66710684392120	-5.33576450212041
C	-0.28716302887200	1.62344750759203	-7.23968155446939
H	-0.56610441718841	0.63353879809206	-7.60997787845881
H	2.35137479668291	-2.79506214854061	-4.74902640372805
H	1.32523801630242	1.47492350467289	-8.62925959664317
H	2.06371081576975	-1.66295447063306	-2.21524183534380
H	4.41545556585895	-1.38616776916179	-3.04827959257042
C	1.18903679739903	1.83280642463222	-7.60358358357039
N	2.10557808143988	1.04904327767404	-6.77920194398318
C	1.61482909614327	3.29788541299695	-7.67869230178966

C	2.97565248459049	-2.79141914764497	-3.84676824710678
O	0.84450792035238	4.22506594286578	-7.66979113541396
O	2.92045874118155	3.51349147917872	-7.91116734258342
C	2.40800135541243	1.44221492901707	-5.51074600552852
C	2.77556804535098	0.31951527337915	-4.53179970171551
O	2.30242062882278	2.60936526732865	-5.16261141830103
C	3.86479154635457	-0.64992478281711	-5.01085971643985
H	1.84777970440007	-0.25813581653364	-4.45285249436641
N	3.00329492802457	0.83890810240031	-3.21274976618911
H	2.12822339047962	0.06130242396015	-6.98383458806576
H	2.20957965404104	0.88884262143932	-2.57520227470235
C	4.12699786900524	1.42815592735850	-2.74489258357419
O	5.12616120927152	1.64190498635405	-3.44593330892576
N	2.14433634640049	-2.56942309543697	-2.66955348831208
O	1.40468913650705	-4.70449372581829	-2.67549136198412
H	-0.87532048990400	2.34868309228405	-7.80494705445471
C	4.12732336025438	-1.78965591235951	-4.02095808181794
H	4.78742319864634	-0.09896611312716	-5.18161838471648
H	3.55893225129144	-1.08350678395563	-5.96548275469163
H	3.38275758224587	-3.79796454687807	-3.75969399674044
H	4.99290866138926	-2.34187052201727	-4.39195308504983
H	3.42073280232744	2.68392473633345	-7.83920680776563

1.11 pH-dependent UV-Vis spectrophotometric speciation

1.11.1 KO-NH2

$FeCl_3$ and $Ga(NO_3)_3$ stocks were generated in DI water, and their exact concentrations were determined by ICP-OES using a 6-point calibration curve (1-100 ppm). **KO-NH2** stocks were also generated in DI water, and their exact concentrations were determined by UV-vis using the known triscatecholate extinction coefficient $\varepsilon_{316\ nm}$ = 9500 $M^{-1}cm^{-1}$.

$FeCl_3$ (52 µL, 15.08 mM, 0.00078 mmol), **KO-NH2** (14.61 mL, 0.054 mM, 0.00078 mmol), KCl (0.186 g, 2.49 mmol), and HCl (2.511 g, 0.1 M, 0.2511 mmol) were combined and diluted to 25.0 mL total volume to create the initial stock **$[Fe(KO\text{-}NH_2)]^{3-}$** solution at approximately pH 2 (0.031 mM **KO-NH2**, 0.031 mM Fe^{3+}, 0.10 M KCl, 0.01 M HCl). $Ga(NO_3)_3$ (117 µL, 6.89 mM, 0.00081 mmol), **KO-NH2** (14.49 mL, 0.056 mM, 0.00081 mmol), KCl (0.192 g, 2.58 mmol), and HCl (2.506 g, 0.1 M, 0.2506 mmol) were combined and diluted to 25.0 mL total volume to create the initial stock **$[Ga(KO\text{-}NH_2)]^{3-}$** solution at approximately pH 2 (0.032 mM **KO-NH2**, 0.032 mM Ga^{3+}, 0.10 M KCl, 0.01 M HCl). 1 mL aliquots of each stock solution were removed, and the pH was adjusted via addition of 0.1 M KOH to generate samples ranging from pH 2-12 with increments of approximately 0.5. Solutions were allowed to be equilibrated overnight prior to analysis. UV-visible spectra were recorded for each sample, and speciation calculations were performed using the HypSpec2014 program. The appearance of a peak at 580 nm in the **$[Fe(KO\text{-}NH_2)]^{3-}$** speciation followed by a shift to 500 nm with increasing pH was interpreted as the stepwise binding of the three catecholate arms of **KO-NH2** to Fe^{3+} in a salicylate mode, generating $[Fe(LH_5)]^{2+}$, $[Fe(LH_4)]^{+}$, then $[Fe(LH_3)]$ species. Between pH 7 and 8, a conversion from the neutral $[Fe(LH_3)]$ species to $[Fe(L)]^{3-}$ is accompanied by a minor (~10 nm) blue shift and increase in intensity, indicating a switch from the salicylate to catecholate binding mode as the second hydroxyl group in each arm is deprotonated. This stepwise coordination was also observed in the red shifting of the catechol peak from 250 nm to 340 nm, followed by an increase intensity. In the $[Ga($**KO-NH2**$)]^{3-}$ speciation, the catechol peak alone was used to model the same coordination behavior. Speciation plots were generated from pH 2-9.5 for **$[Ga(KO\text{-}NH_2)]^{3-}$** and from pH 2-10 for **$[Fe(KO\text{-}NH_2)]^{3-}$**.

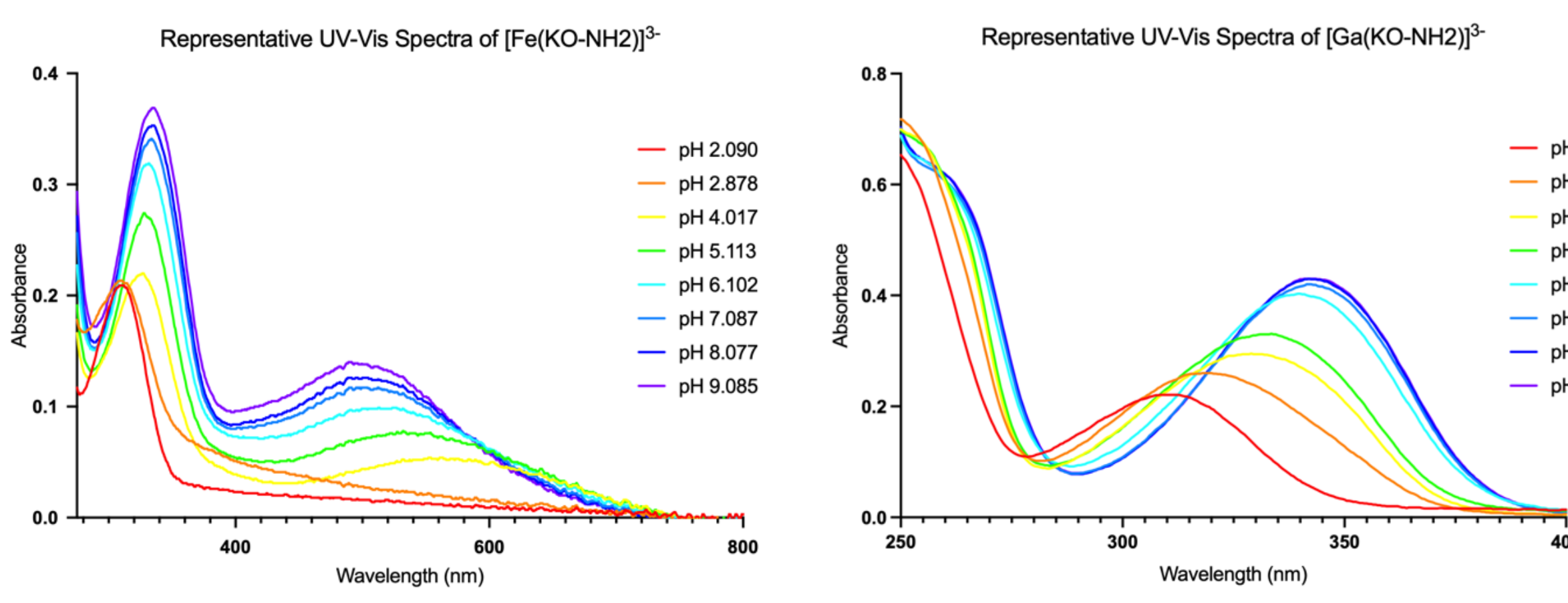

Figure S114. Select pH-dependent UV-vis spectra of **$[M(KO\text{-}NH_2)]^{3-}$** (M = Fe^{3+}, Ga^{3+}) that were used to calculate Logß values for relevant species.

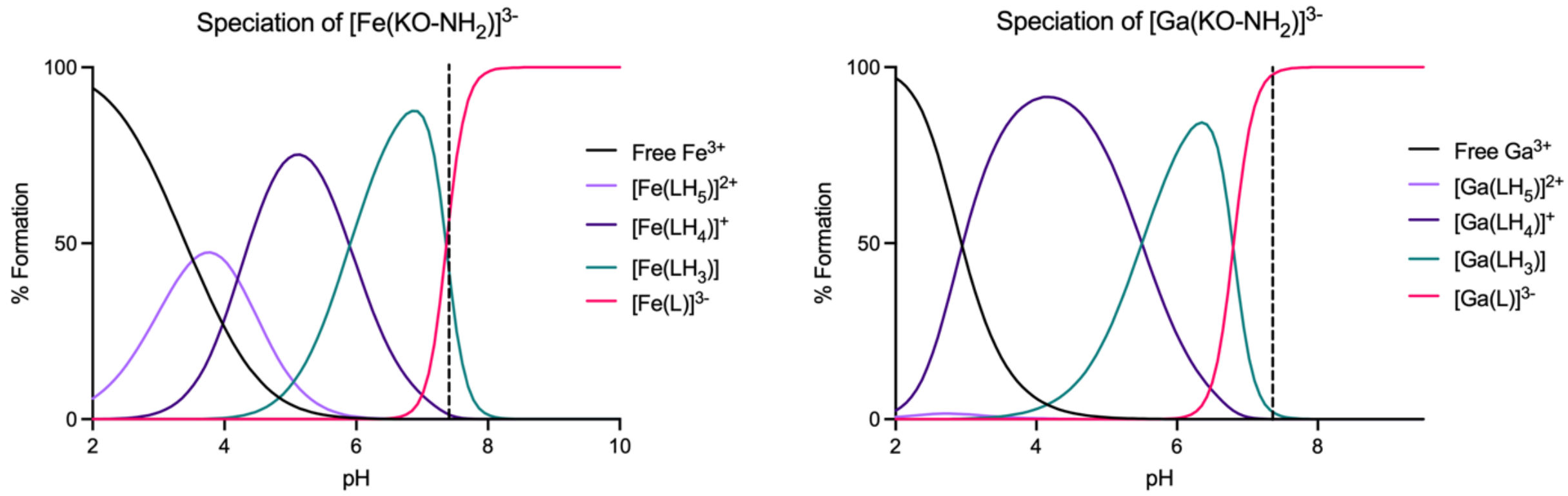

Figure S115. Speciation plots of **[M(KO-NH$_2$)]$^{3-}$** (M = Fe^{3+}, Ga^{3+}). The dashed line denotes physiologically relevant pH (7.4).

In speciation calculations, Logß values for the six relevant **KO-NH$_2$** protonations were approximated from reported values for the structurally similar tricatecholate ligand **TREN-CAM**.[3] Starting from this estimation, ligand Logß values were refined in the HypSpec2014 model, and the same final values were used for speciation of both **[Fe(KO-NH$_2$)]$^{3-}$** and **[Ga(KO-NH$_2$)]$^{3-}$**.

Table S13. Tabulated Logß values for LH_6 = **KO-NH$_2$**.

Species	Logß
(LH_6)	66.86
$(LH_5)^-$	57.87
$(LH_4)^{2-}$	48.04
$(LH_3)^{3-}$	39.12
$(LH_2)^{4-}$	26.50
$(LH)^{5-}$	14.68

Table S14. Tabulated Logß values for Fe^{3+} and Ga^{3+} complexes with **KO-NH$_2$** species discussed in this work.

Species	Logß, M = Ga^{3+}	Logß, M = Fe^{3+}
$[M(LH_5)]^{2+}$	67.2	68.2
$[M(LH_4)]^+$	65.8	64.0
$[M(LH_3)]$	60.3	58.1
$[M(L)]^{3-}$	39.9	36.0

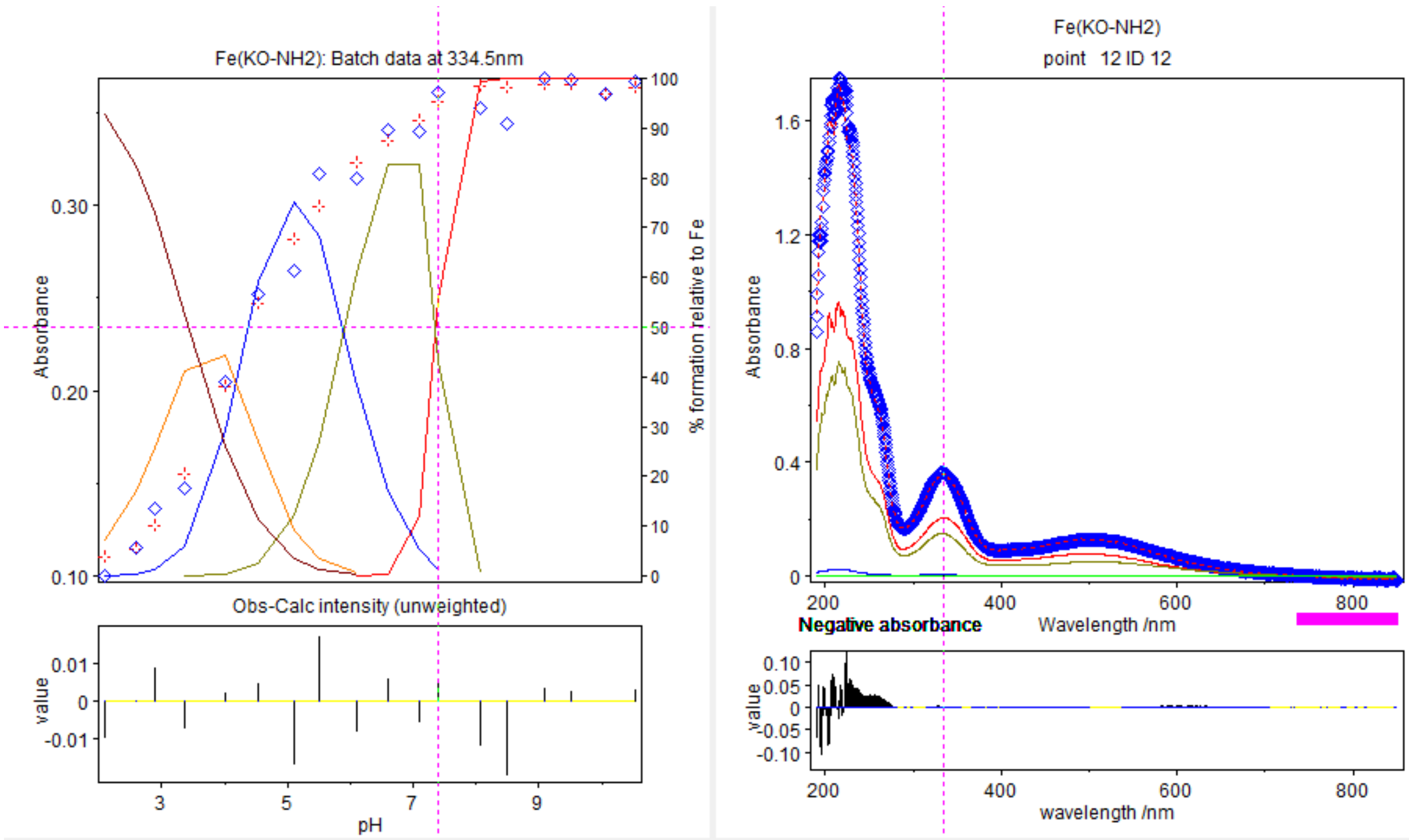

Figure S116. Representative data fit of **[Fe(KO-NH$_2$)]$^{3-}$** in HypSpec2014 using the catechol peak.

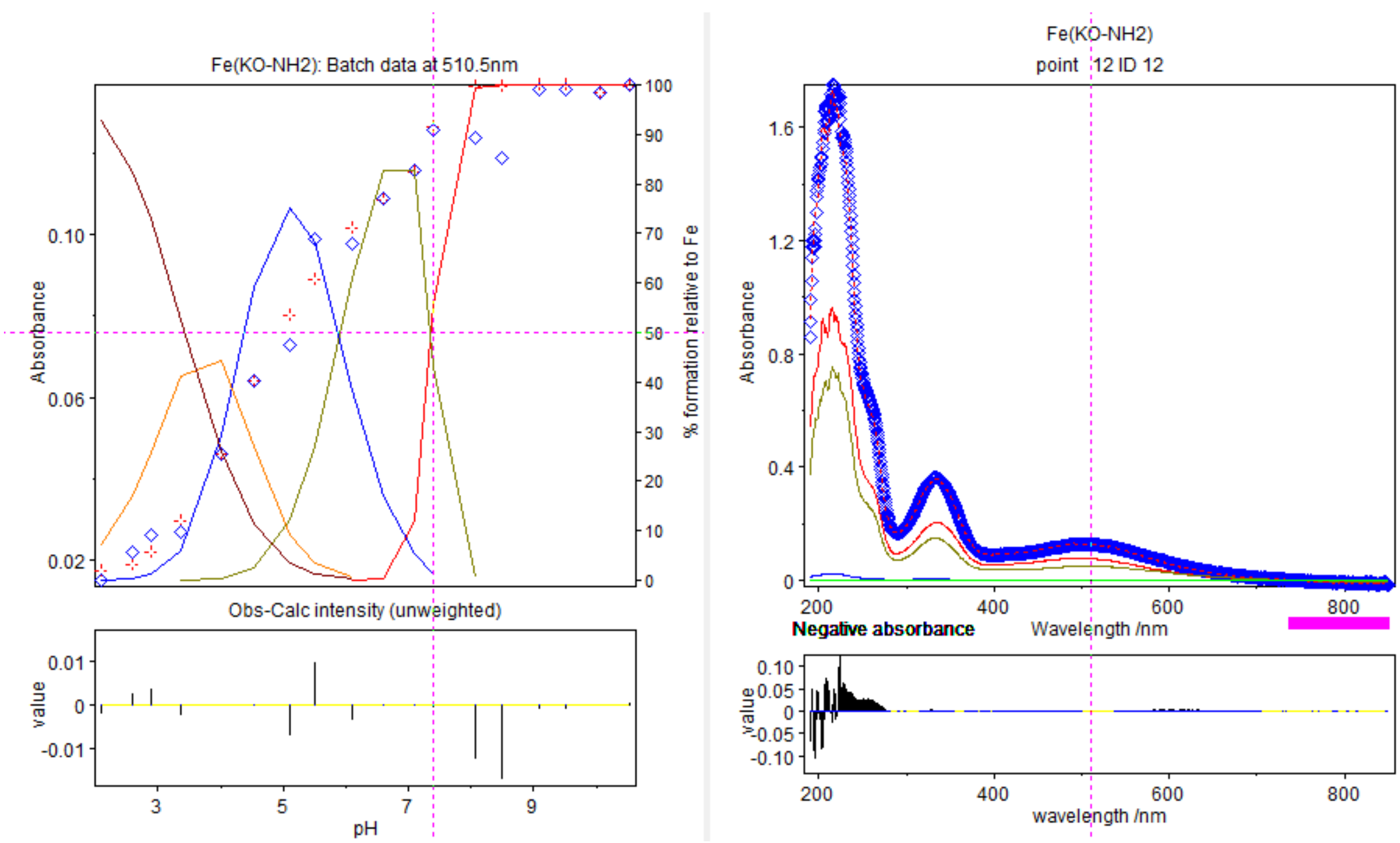

Figure S117. Representative data fit of **[Fe(KO-NH$_2$)]$^{3-}$** in HypSpec2014 using the LMCT peak.

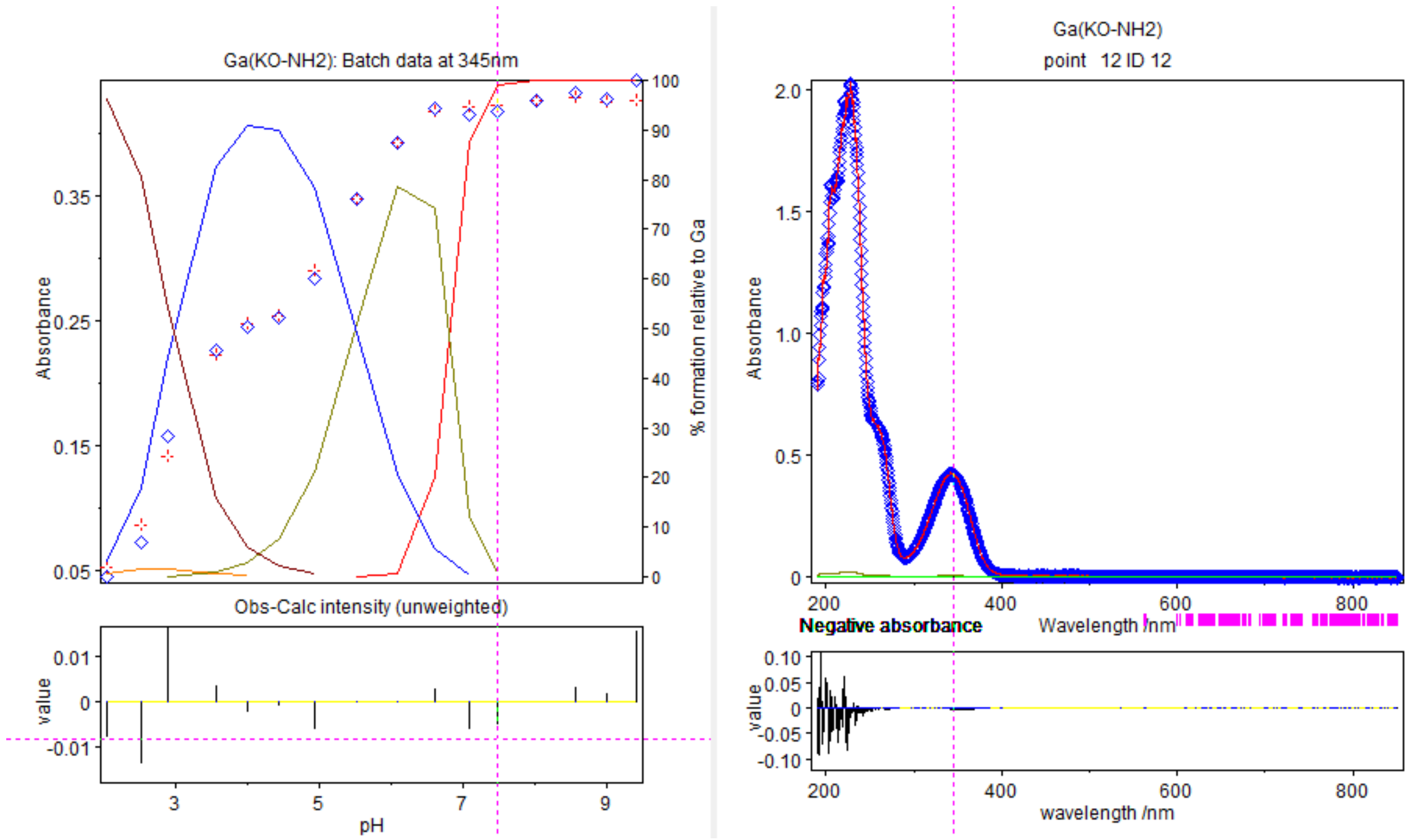

Figure S118. Representative data fit of **[Ga(KO-NH₂)]$^{3-}$** in HypSpec2014 using the catechol peak.

1.11.2 Ent

$FeCl_3$ and $Ga(NO_3)_3$ stocks were generated in DI water, and their exact concentrations were determined by ICP-OES using a 6-point calibration curve (1-100 ppm). **Ent** stocks were also generated in DI water, and their exact concentrations were determined by UV-vis using the known tris-catecholate extinction coefficient $\varepsilon_{316\ nm}$ = 9500 $M^{-1}cm^{-1}$.

$FeCl_3$ (85 µL, 12.0 mM, 0.0010 mmol), **Ent** (9.47 mL, 0.108 mM, 0.0010 mmol), KCl (0.186 g, 2.49 mmol), and HCl (2.498 g, 0.1 M, 0.2498 mmol) were combined and diluted to 25.0 mL total volume to create the initial stock **$[Fe(Ent)]^{3-}$** solution at approximately pH 2 (0.041 mM **Ent**, 0.041 mM Fe^{3+}, 0.10 M KCl, 0.01 M HCl). $Ga(NO_3)_3$ (192 µL, 8.47 mM, 0.0016 mmol), **Ent** (12.52 mL, 0.130 mM, 0.0016 mmol), KCl (0.186 g, 2.49 mmol), and HCl (2.498 g, 0.1 M, 0.2498 mmol) were combined and diluted to 25.0 mL total volume to create the initial stock **$[Ga(Ent)]^{3-}$** solution at approximately pH 2 (0.064 mM **Ent**, 0.064 mM Ga^{3+}, 0.10 M KCl, 0.01 M HCl). 1 mL aliquots of each stock solution were removed, and the pH was adjusted via addition of 0.1 M KOH to generate samples ranging from pH 2-12 with increments of approximately 0.5. Solutions were allowed to equilibrate overnight prior to analysis. UV-visible spectra were recorded for each sample, and speciation calculations were performed using the HypSpec2014 program.

The appearance of a broad peak at approximately 580 nm in the **$[Fe(Ent)]^{3-}$** speciation followed by a blue shift to 500 nm was interpreted as the stepwise binding of the three catecholate arms of **Ent** to Fe^{3+} in a salicylate mode, generating $[Fe(LH_5)]^{2+}$, $[Fe(LH_4)]^{+}$, then $[Fe(LH_3)]$ species. Between pH 5.5 and 7.5, a conversion from the neutral $[Fe(LH_3)]$ species to $[Fe(L)]^{3-}$ is accompanied by a minor (~10 nm) blue shift and a slight increase in intensity, indicating a switch from the salicylate to catecholate binding mode as the second hydroxyl group in each arm is deprotonated. This stepwise coordination was also observed in the red shifting of the catechol peak from 320 nm to 335 nm, followed by an increase in intensity. Above pH 10, both the catechol and LMCT peaks blue shift, the former to 325 nm and the latter to approximately 450 (with peak broadening). These changes were hypothesized to result from some combination of catechol oxidation and hydrolysis of the trilactone backbone of **Ent**. The hydrolyzed species $[M(LH_{-1})]^{4-}$ and $[M(LH_{-2})]^{5-}$, with one and two trilactone ester bonds hydrolyzed respectively, were included in the speciation model to account for this high-pH behavior. (With the cause of the high-pH spectral changes in the **Ent** complexes unclear, these two species could also represent oxidized complexes.) In the **$[Ga(Ent)]^{3-}$** speciation, the catechol peak alone was used to model the same coordination behavior described above. Speciation plots were generated from pH 2-12 for both **$[Fe(Ent)]^{3-}$** and **$[Ga(Ent)]^{3-}$**.

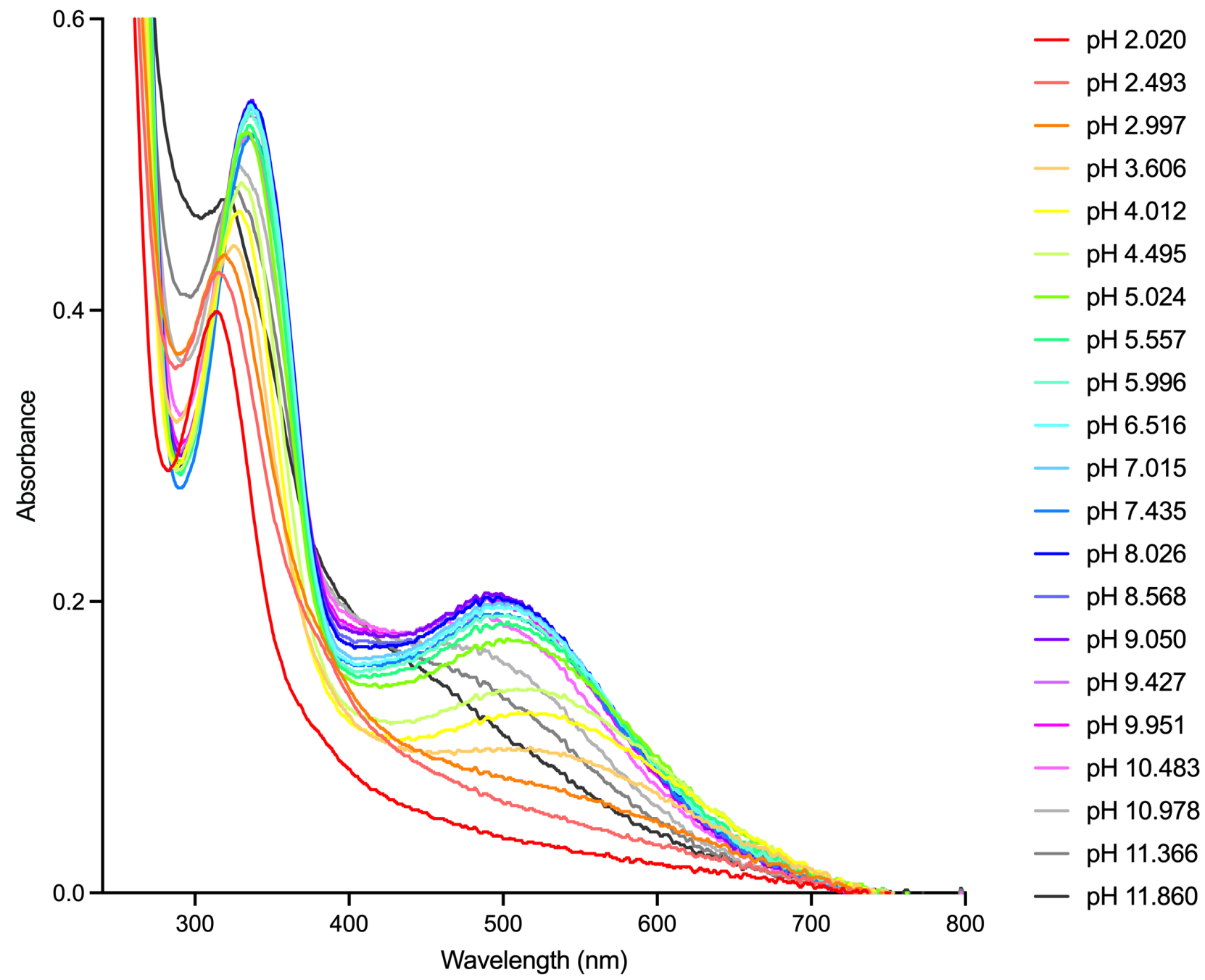

Figure S119. pH-dependent UV-vis spectra of **[Fe(Ent)]$^{3-}$** that were used to calculate Logß values for relevant species.

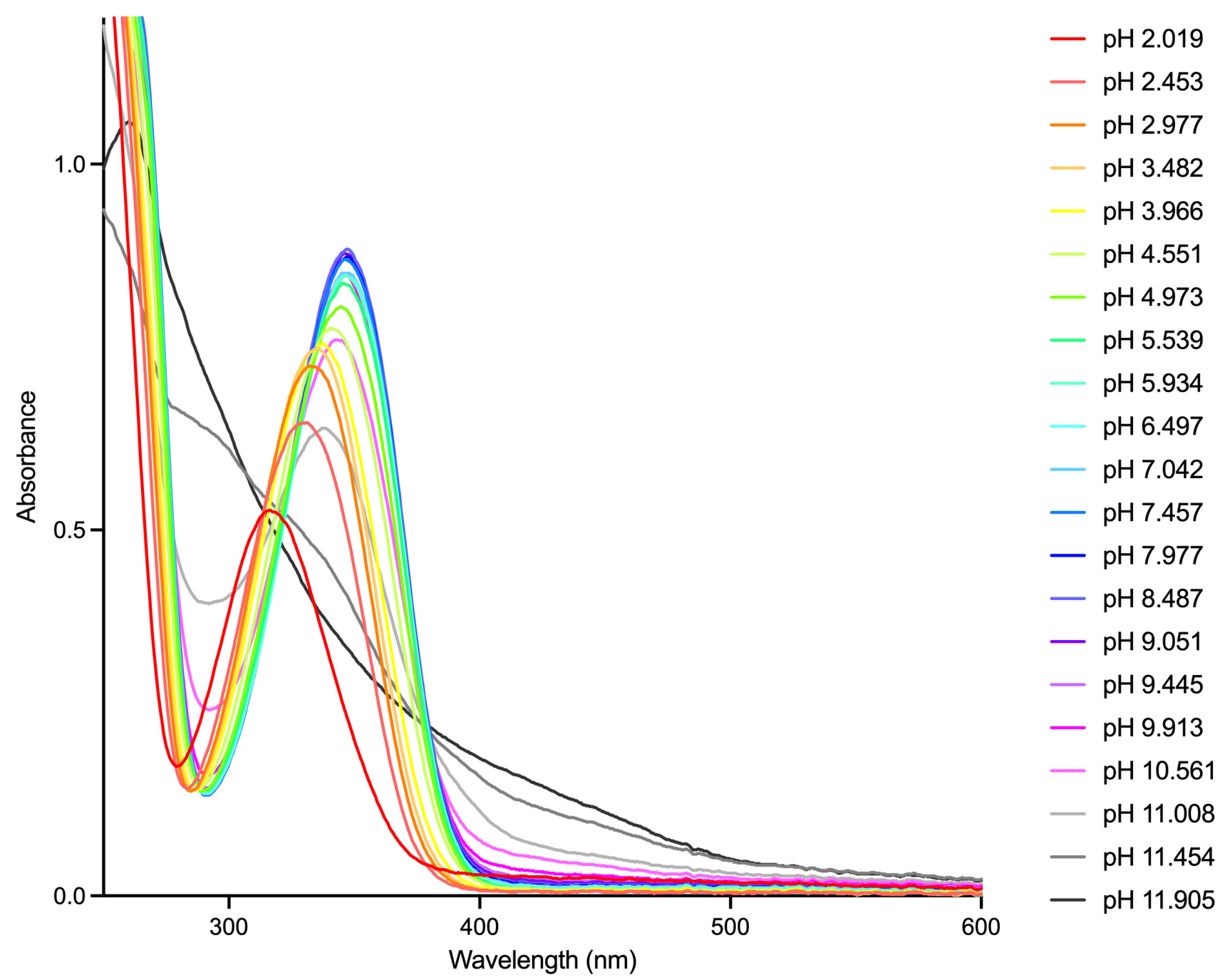

Figure S120. pH-dependent UV-vis spectra of **[Ga(Ent)]$^{3-}$** that were used to calculate Logß values for relevant species.

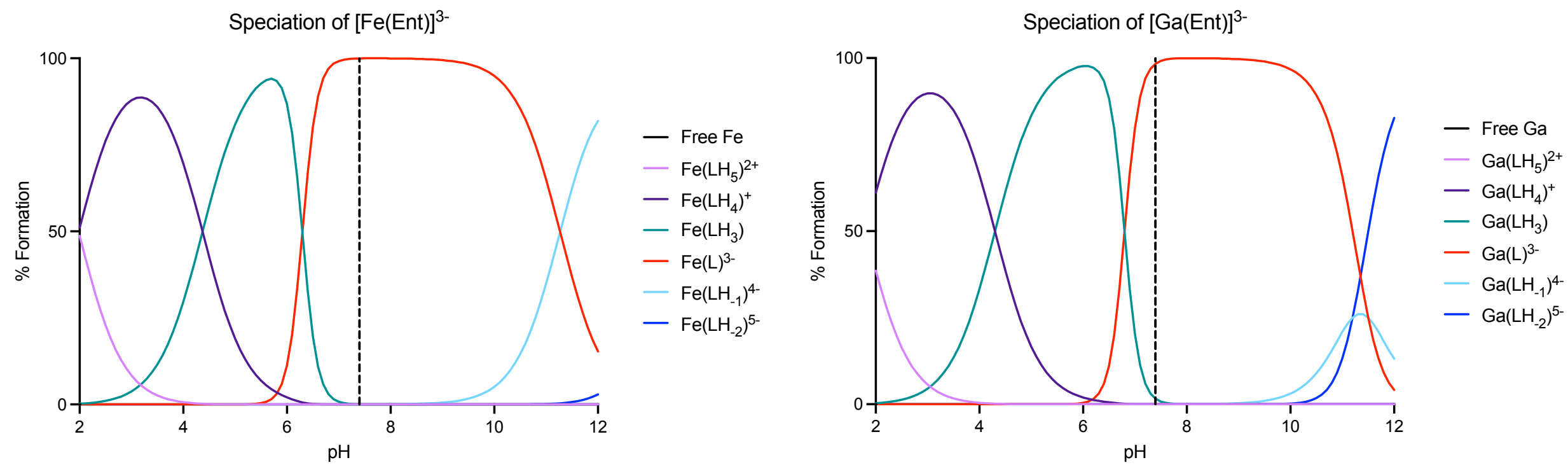

Figure S121. Speciation plots of **[M(Ent)]$^{3-}$** (M = Fe^{3+}, Ga^{3+}). The dashed line denotes physiologically relevant pH (7.4).

Table S15. Tabulated Logß values for LH_6 = Ent

**Species**	**Logß**
(LH_6)	57.7
$(LH_5)^-$	51.7
$(LH_4)^{2-}$	44.2
$(LH_3)^{3-}$	35.4
$(LH_2)^{4-}$	23.9
$(LH)^{5-}$	12.1

Table S16. Tabulated Logß values for Fe^{3+} and Ga^{3+} complexes with **Ent** species discussed in this work.

**Species**	**Logß, M = Ga^{3+}**	**Logß, M = Fe^{3+}**
$[M(LH_5)]^{2+}$	75.00	75.40
$[M(LH_4)]^{+}$	73.20	73.42
$[M(LH_3)]$	68.90	69.05
$[M(L)]^{3-}$	48.50	50.16
$[M(LH_{-1})]^{4-}$	37.00	38.89
$[M(LH_{-2})]^{5-}$	25.80	25.43

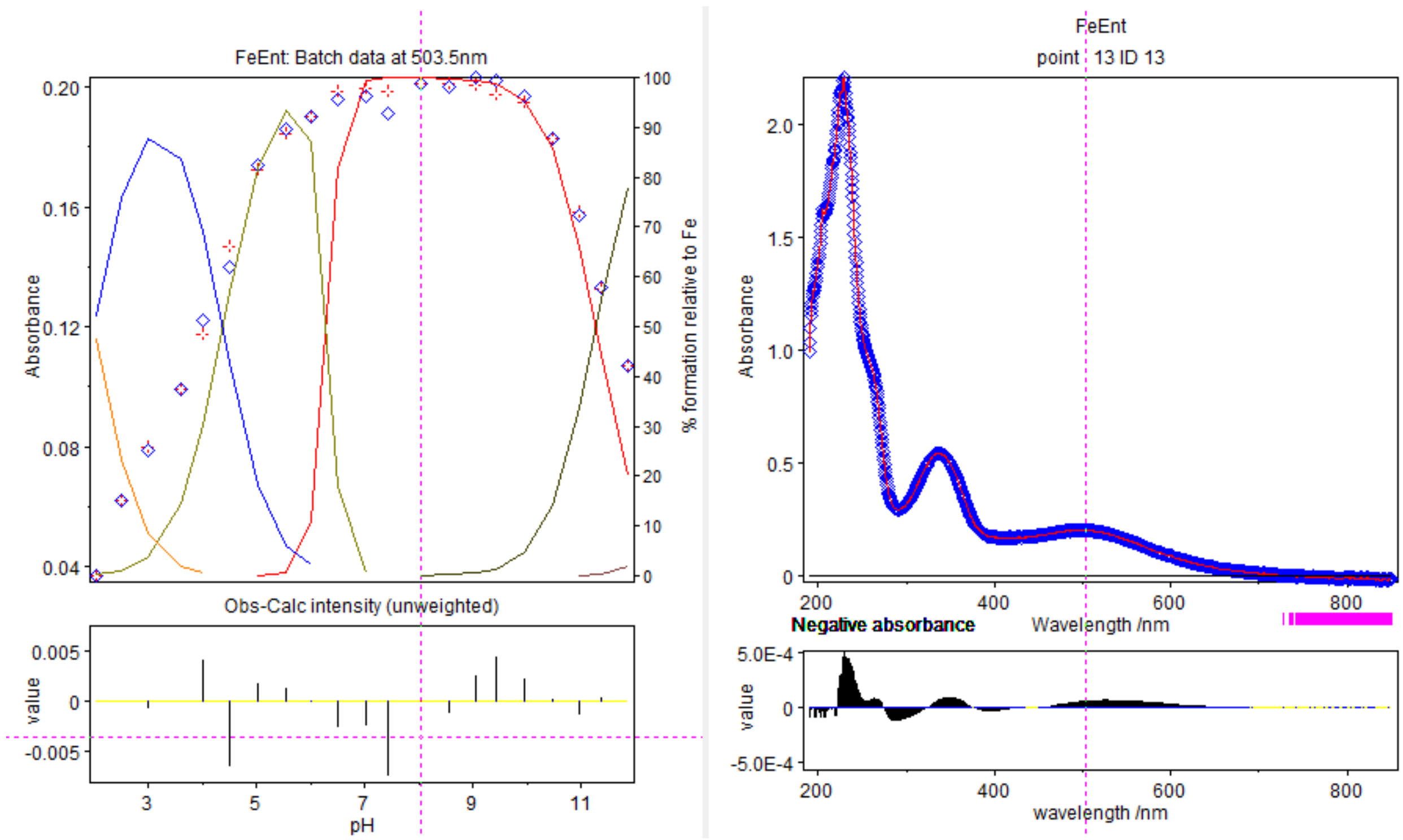

Figure S122. Representative data fit of **[Fe(Ent)]$^{3-}$** in HypSpec2014 using the LMCT peak.

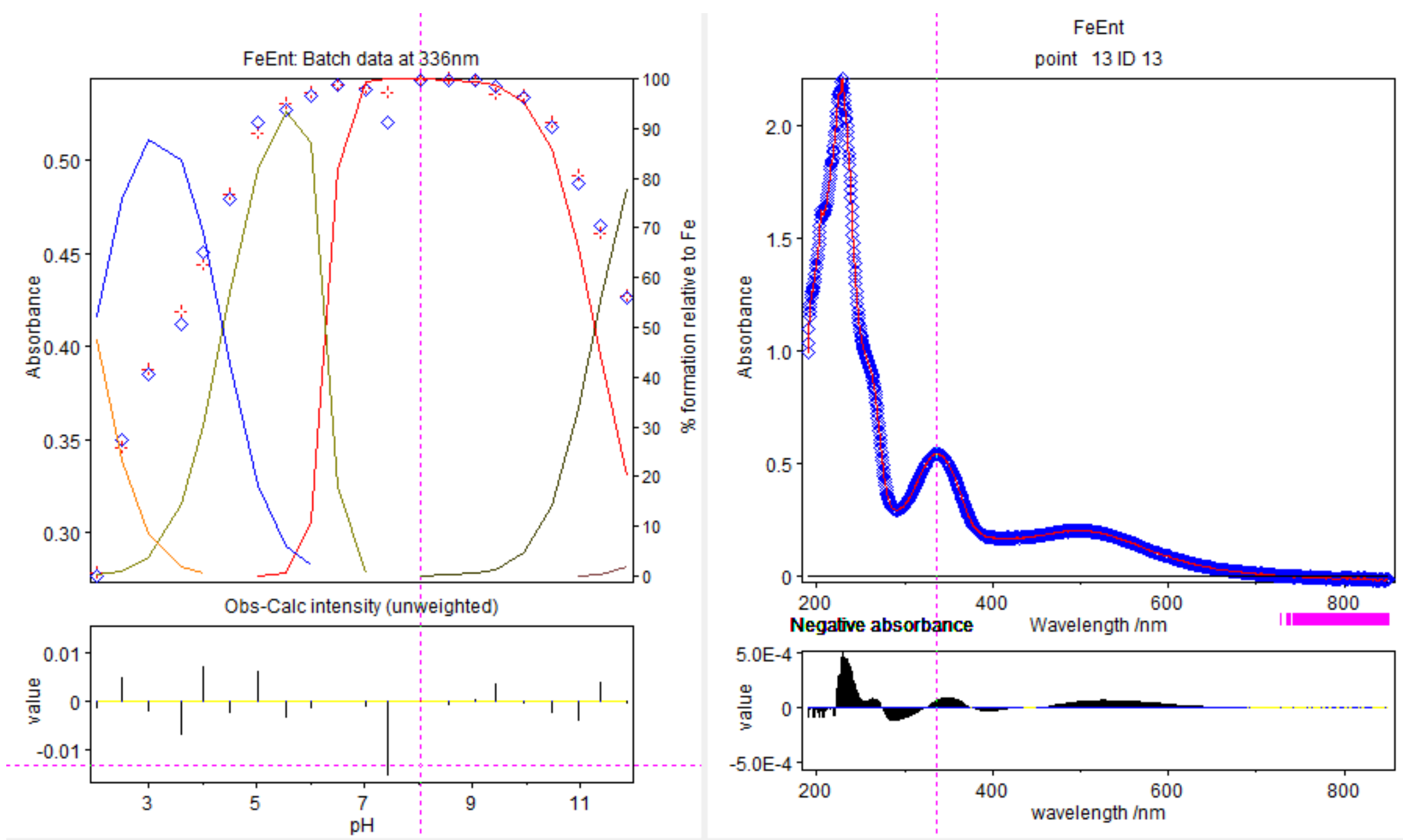

Figure S123. Representative data fit of **[Fe(Ent)]$^{3-}$** in HypSpec2014 using the catechol peak.

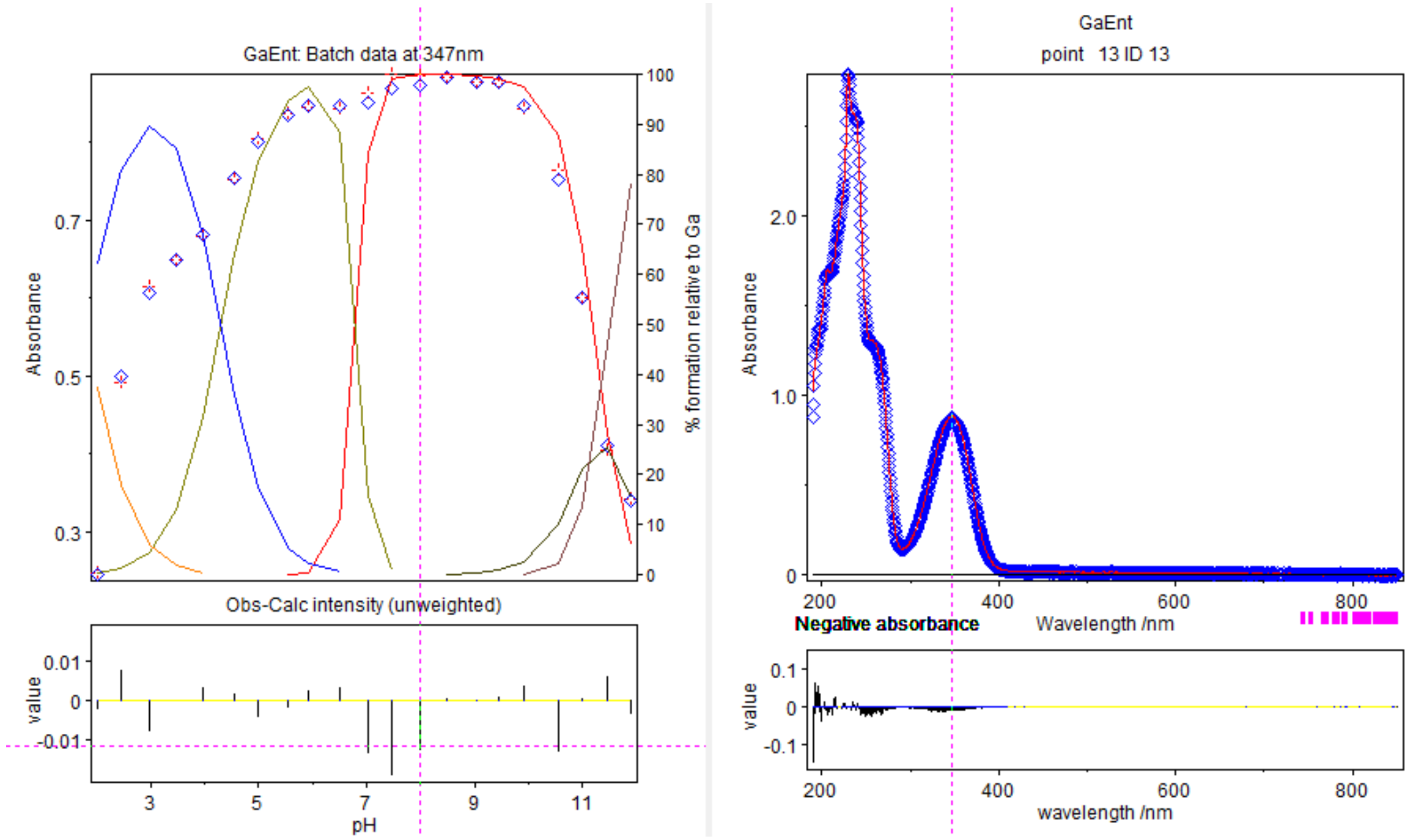

Figure S124. Representative data fit of **[Ga(Ent)]$^{3-}$** in HypSpec2014 using the catechol peak.

1.11.3 TREN-CAM

$FeCl_3$ and $Ga(NO_3)_3$ stocks were generated in DI water, and their exact concentrations were determined by ICP-OES using a 6-point calibration curve (1-100 ppm). **TREN-CAM** stocks were also generated in DI water, and their exact concentrations were determined by UV-vis using the known tris-catecholate extinction coefficient $\varepsilon_{316\ nm} = 9500\ M^{-1}cm^{-1}$.

$FeCl_3$ (29 µL, 51.77 mM, 0.0015 mmol), **TREN-CAM** (4.68 mL, 0.321 mM, 0.0015 mmol), KCl (0.186 g, 2.49 mmol), and HCl (2.506 g, 0.1 M, 0.2506 mmol) were combined and diluted to 25.0 mL total volume to create the initial stock **[Fe(TREN-CAM)]$^{3-}$** solution at approximately pH 2 (0.060 mM **TREN-CAM**, 0.060 mM Fe^{3+}, 0.10 M KCl, 0.01 M HCl). $Ga(NO_3)_3$ (217 µL, 6.91 mM, 0.0015 mmol), **TREN-CAM** (5.58 mL, 0.269 mM, 0.0015 mmol), KCl (0.186 g, 2.49 mmol), and HCl (2.500 g, 0.1 M, 0.2500 mmol) were combined and diluted to 25.0 mL total volume to create the initial stock **[Ga(TREN-CAM)]$^{3-}$** solution at approximately pH 2 (0.060 mM **TREN-CAM**, 0.060 mM Ga^{3+}, 0.10 M KCl, 0.01 M HCl). 1 mL aliquots of each stock solution were removed, and the pH was adjusted via addition of 0.1 M KOH to generate samples ranging from pH 2-12 with increments of approximately 0.5. Solutions were allowed to equilibrate overnight prior to analysis. UV-visible spectra were recorded for each sample, and speciation calculations were performed using the HypSpec2014 program.

In the absorbance spectra of **[Fe(TREN-CAM)]$^{3-}$**, the growth of a broad peak centered at approximately 530 nm coincides with the formation of a dark purple precipitate between pH 2.5 and 5 in samples refrigerated overnight, with the most precipitate visible at pH 4. These features were interpreted as the formation of a neutral [$Fe(LH_3)$] species in which two ligand arms are coordinated in the salicylate mode and one arm is coordinated in the catecholate mode, with the central tertiary amine of **TREN-CAM** protonated as well. The presence of one species with mixed coordination modes suggests fully stepwise coordination of the ligand's three arms, in which each catechol coordinates in the salicylate mode before each switches sequentially to the catecholate mode as the second hydroxyl group is deprotonated. The shift from salicylate to catecholate binding was modeled as three distinct species rather than three simultaneous deprotonations, with the final $[Fe(L)]^{3-}$ species generated by deprotonation of the central amine. The blue shift of the charge transfer peak from 530 nm to 500 nm and subsequent growth in intensity were interpreted as stepwise salicylate coordination and stepwise catecholate coordination respectively. (The initial blue shift between pH 2 and 5 is less apparent in this case due to peak broadening.) This stepwise coordination was also observed in the red shifting of the catechol peak from 320 nm to 340 nm, followed by an increase in intensity. In the **[Ga(TREN-CAM)]$^{3-}$** speciation, the catechol peak alone was used to model the same coordination behavior. The earlier red shift of the catechol peak for **[Ga(TREN-CAM)]$^{3-}$** was attributed to the harder Lewis acidity of Ga^{3+} relative to Fe^{3+}, which may drive coordination at lower pH values. The slight growth of a broad feature at around 400 nm after pH 9 in both complexes is likely indicative of catechol oxidation; an oxidized ligand species was incorporated into the speciation model accordingly. Speciation plots were generated from pH 2-9.5 for **[Fe(TREN-CAM)]$^{3-}$** and from pH 2-12 for **[Ga(TREN-CAM)]$^{3-}$**.

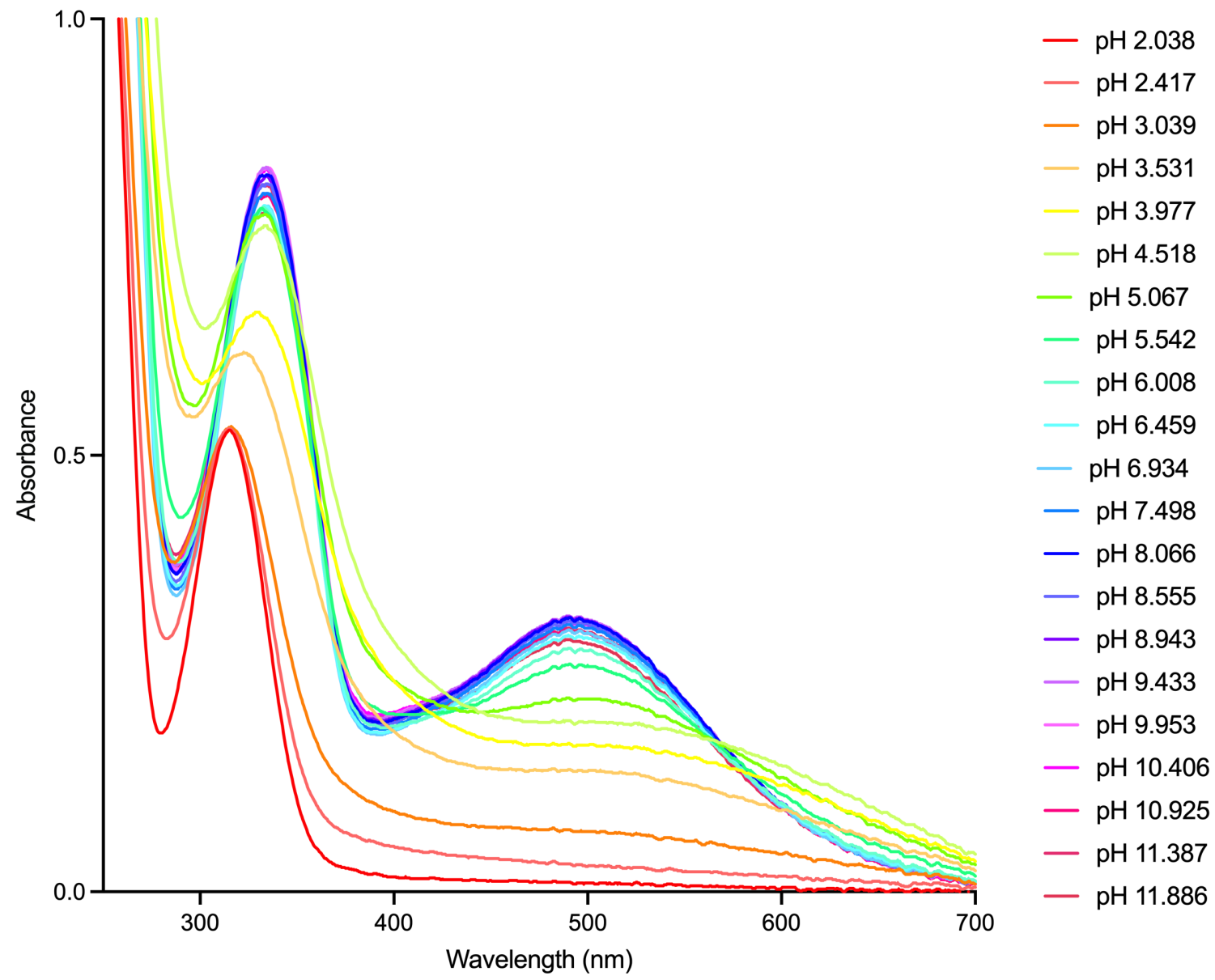

Figure S125. pH-dependent UV-vis spectra of **[Fe(TREN-CAM)]$^{3-}$** that were used to calculate Logß values for relevant species.

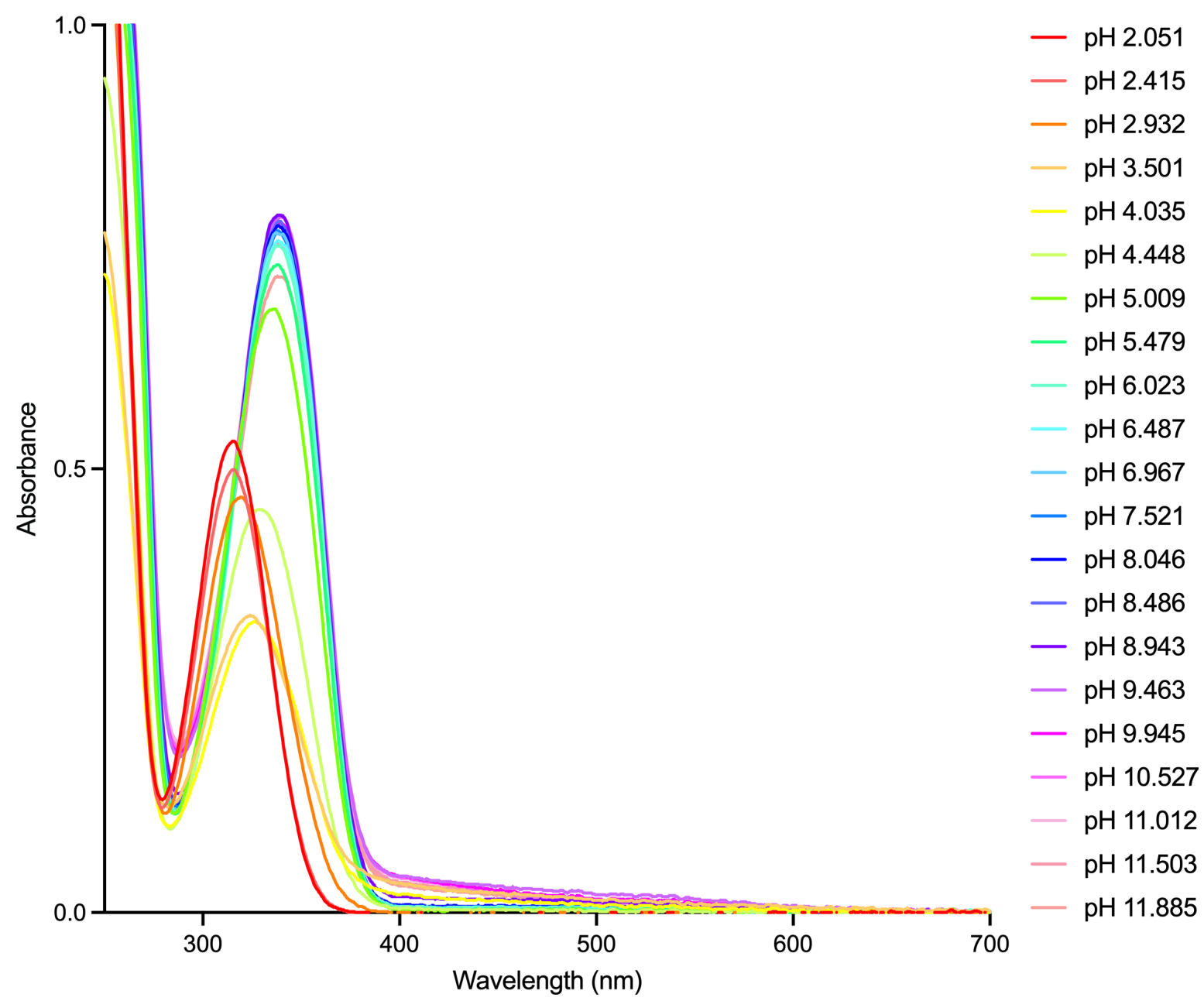

Figure S126. pH-dependent UV-vis spectra of **[Ga(TREN-CAM)]$^{3-}$** that were used to calculate Logß values for relevant species.

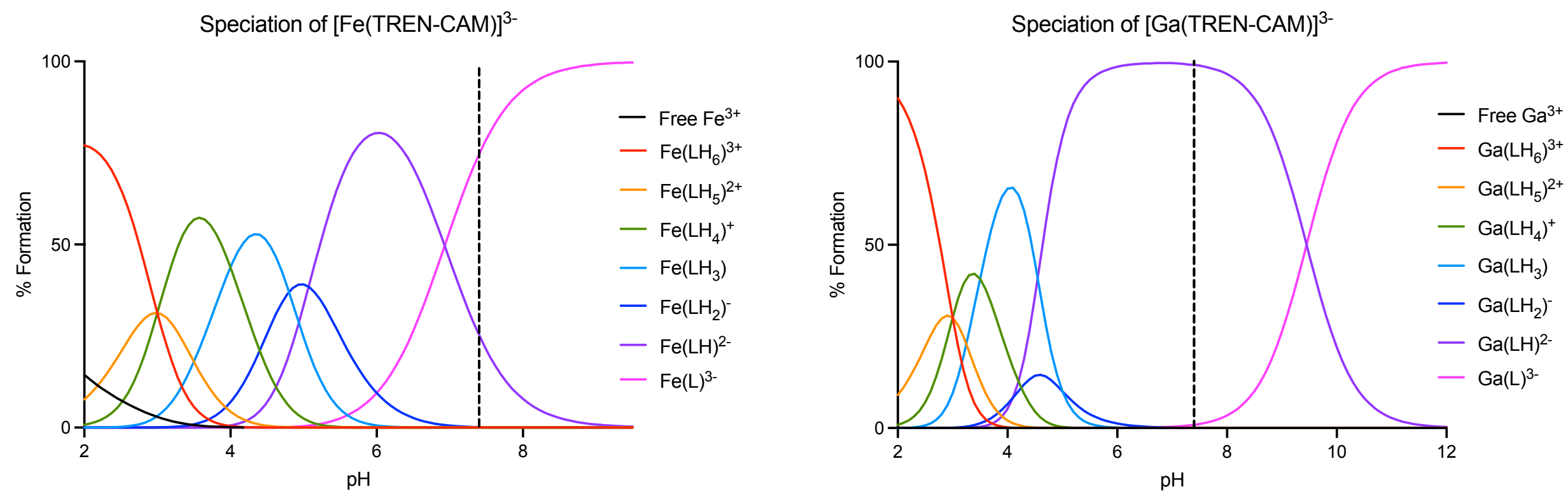

Figure S127. Speciation plots of **$[M(TREN\text{-}CAM)]^{3-}$** (M = Fe^{3+}, Ga^{3+}). The dashed line denotes physiologically relevant pH (7.4).

Table S17. Tabulated Logß values for LH_6 = **TREN-CAM**

Species	Logß
$(LH_7)^{+}$	66.21
(LH_6)	60.33
$(LH_5)^{-}$	53.62
$(LH_4)^{2-}$	45.01
$(LH_3)^{3-}$	36.26
$(LH_2)^{4-}$	25.00
$(LH)^{5-}$	12.90
$(LH_{-1})^{4-}$	11.00

Table S18. Tabulated Logß values for Fe^{3+} and Ga^{3+} complexes with **TREN-CAM** species discussed in this work.

**Species**	**Logß, M = Ga^{3+}**	**Logß, M = Fe^{3+}**
$[M(LH_6)]^{3+}$	68.0	70.0
$[M(LH_5)]^{2+}$	65.0	67.0
$[M(LH_4)]^{+}$	62.0	64.0
$[M(LH_3)]$	58.5	60.0
$[M(LH_2)]^{-}$	53.5	55.2
$[M(LH)]^{2-}$	49.4	50.1
$[M(L)]^{3-}$	40.0	43.2

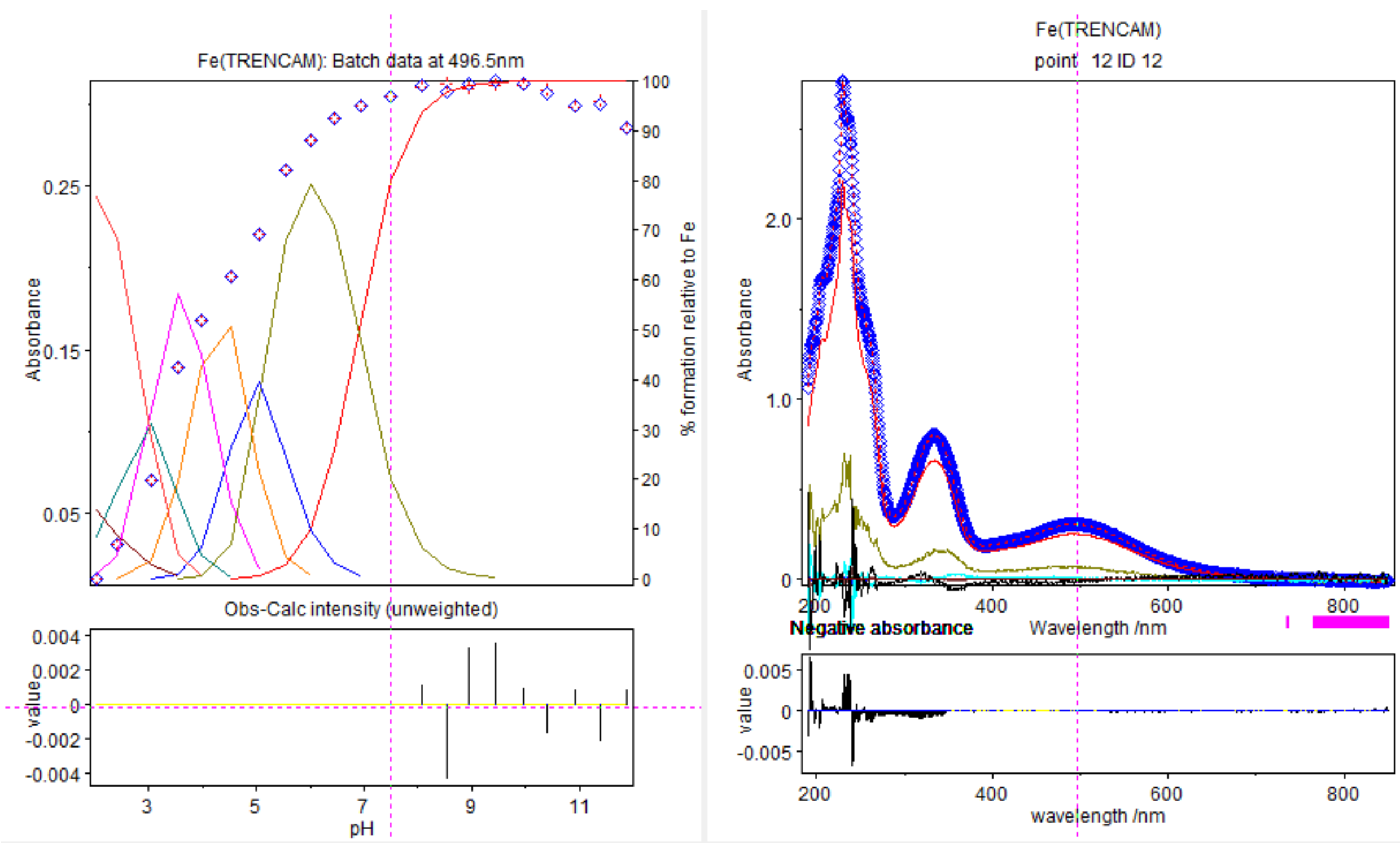

Figure S128. Representative data fit of $\mathbf{[Fe(TREN\text{-}CAM)]^{3-}}$ in HypSpec2014 using the LMCT peak.

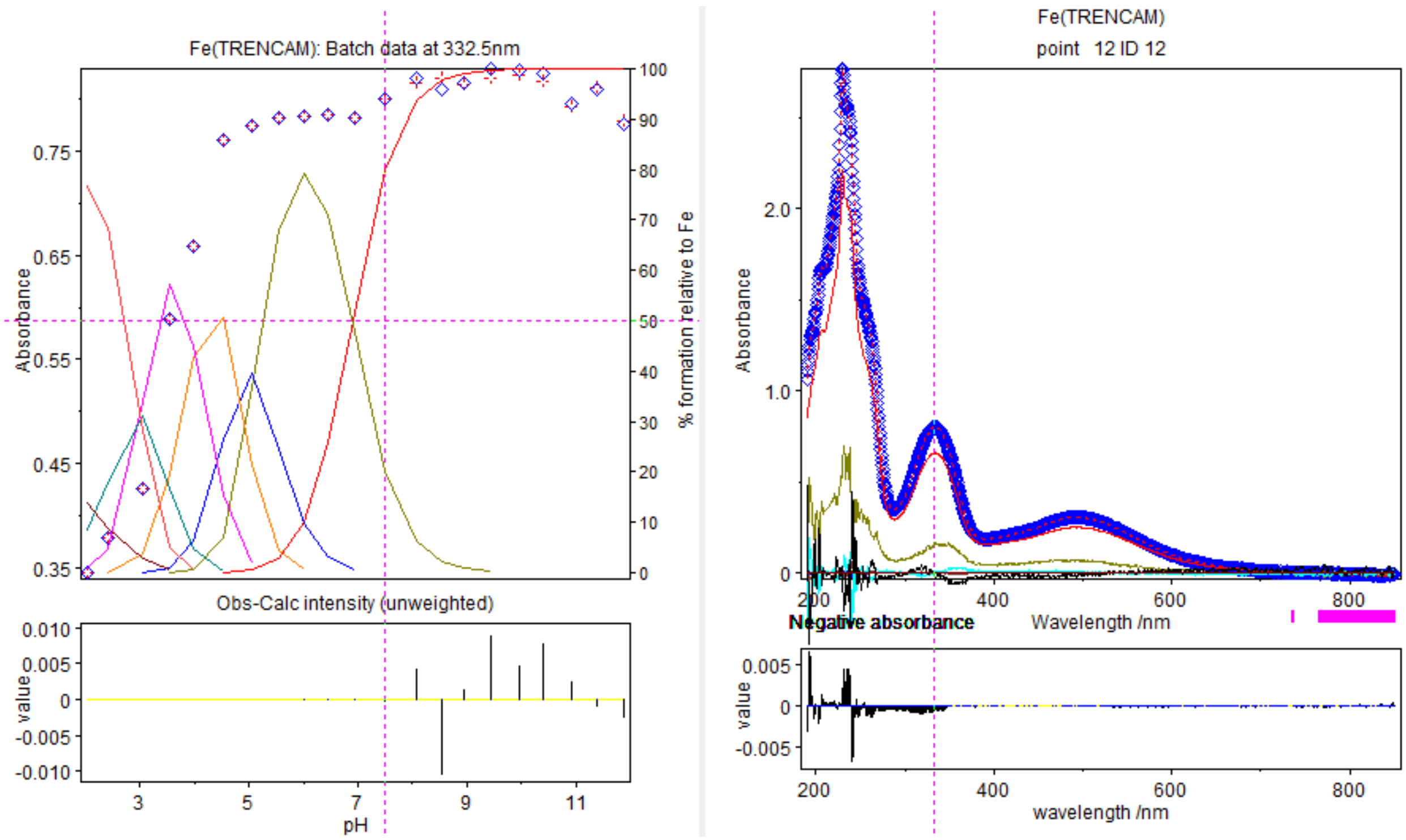

Figure S129. Representative data fit of **[Fe(TREN-CAM)]$^{3-}$** in HypSpec2014 using the catechol peak.

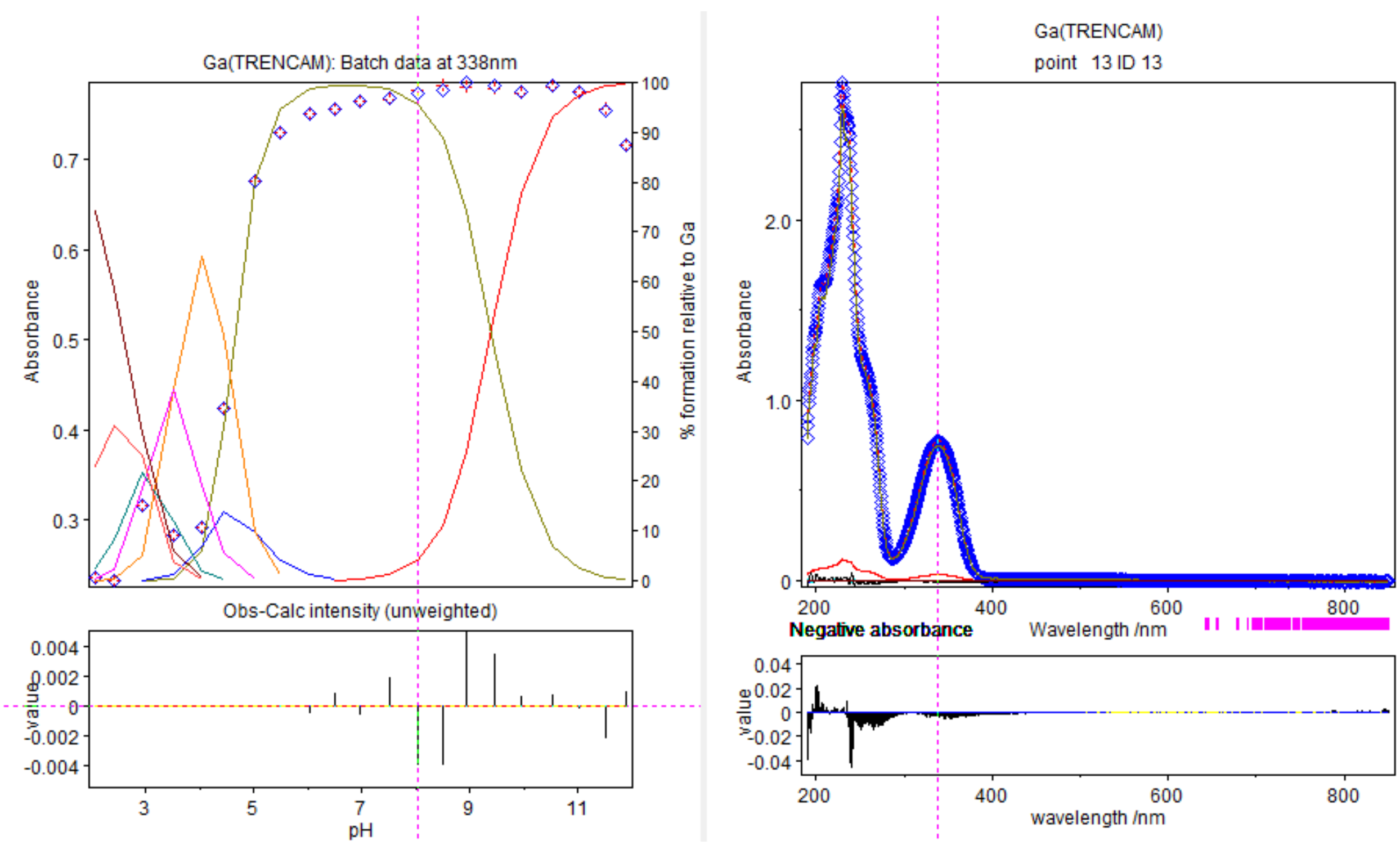

Figure S130. Representative data fit of **[Ga(TREN-CAM)]$^{3-}$** in HypSpec2014 using the catechol peak.

1.11.4 OK-COOH

$FeCl_3$ and $Ga(NO_3)_3$ stocks were generated in DI water, and their exact concentrations were determined by ICP-OES using a 6-point calibration curve (1-100 ppm). **OK-COOH** stocks were also generated in DI water, and their exact concentrations were determined by UV-vis using the known tris-catecholate extinction coefficient $\varepsilon_{316\ nm} = 9500\ M^{-1}cm^{-1}$.

$FeCl_3$ (96 µL, 11.02 mM, 0.0011 mmol), **OK-COOH** (14.52 mL, 0.073 mM, 0.0011 mmol), KCl (0.186 g, 2.49 mmol), and HCl (2.501 g, 0.1 M, 0.2501 mmol) were combined and diluted to 25.0 mL total volume to create the initial stock **$[Fe(OK\text{-}COOH)]^{4-}$** solution at approximately pH 2 (0.042 mM **OK-COOH**, 0.042 mM Fe^{3+}, 0.10 M KCl, 0.01 M HCl). $Ga(NO_3)_3$ (182 µL, 6.91 mM, 0.0013 mmol), **OK-COOH** (14.57 mL, 0.086 mM, 0.0013 mmol), KCl (0.186 g, 2.49 mmol), and HCl (2.500 g, 0.1 M, 0.2500 mmol) were combined and diluted to 25.0 mL total volume to create the initial stock **$[Ga(OK\text{-}COOH)]^{4-}$** solution at approximately pH 2 (0.050 mM **OK-COOH**, 0.050 mM Ga^{3+}, 0.10 M KCl, 0.01 M HCl). 1 mL aliquots of each stock solution were removed, and the pH was adjusted via addition of 0.1 M KOH to generate samples ranging from pH 2-12 with increments of approximately 0.5. Solutions were allowed to equilibrate overnight prior to analysis. UV-visible spectra were recorded for each sample, and speciation calculations were performed using the HypSpec2014 program.

The appearance of a peak at 580 nm in the **$[Fe(OK\text{-}COOH)]^{4-}$** speciation followed by a shift to 500 nm with increasing pH was interpreted as the stepwise binding of the three catecholate arms of **OK-COOH** to Fe^{3+} in a salicylate mode, generating $[Fe(LH_6)]^{2+}$, $[Fe(LH_5)]^{+}$, $[Fe(LH_4)]$, then $[Fe(LH_3)]^{-}$ species. (Both $[Fe(LH_6)]^{2+}$ and $[Fe(LH_5)]^{+}$ are coordinated through one arm, with the acid terminus of **OK-COOH** protonated in the former and deprotonated in the latter.) Between pH 7 and 9, a conversion from the $[Fe(LH_3)]^{-}$ species to $[Fe(L)]^{4-}$ is accompanied by a minor (~10 nm) blue shift and increase in intensity, indicating a switch from the salicylate to catecholate binding mode as the second hydroxyl group in each arm is deprotonated. This stepwise coordination was also observed in the red shifting of the catechol peak from 315 nm to 335 nm, followed by an increase in intensity. In the **$[Ga(OK\text{-}COOH)]^{3-}$** speciation, the catechol peak alone was used to model the same coordination behavior. The more significant formation of $[M(LH_6)]^{2+}$ species at low pH for Ga^{3+} was attributed to its harder Lewis acidity relative to Fe^{3+}, which may drive earlier coordination to the first catechol arm. Speciation plots were generated from pH 2-12 for both **$[Ga(OK\text{-}COOH)]^{3-}$** and **$[Fe(OK\text{-}COOH)]^{3-}$**.

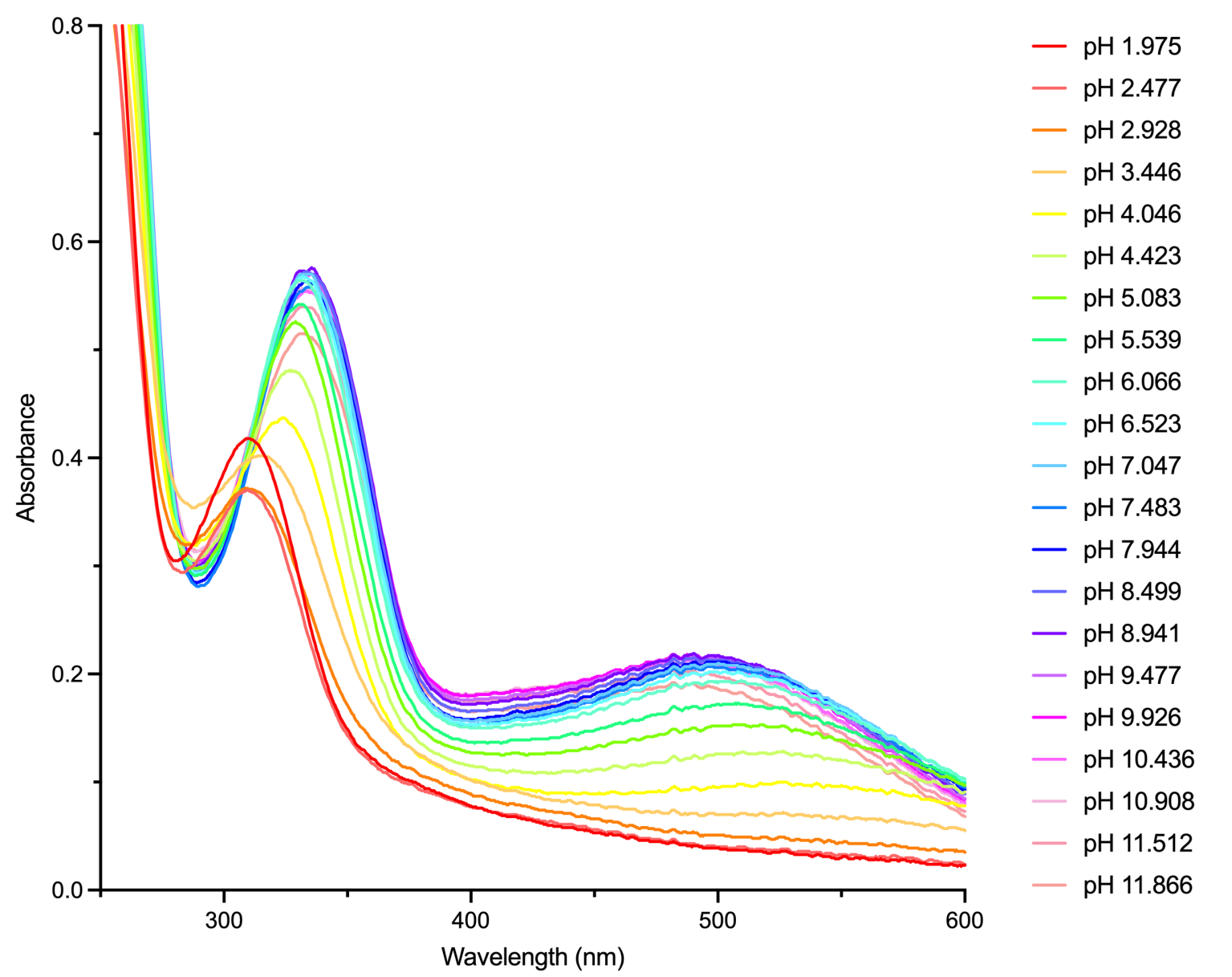

Figure S131. pH-dependent UV-vis spectra of **[Fe(OK-COOH)]$^{3-}$** that were used to calculate Logß values for relevant species.

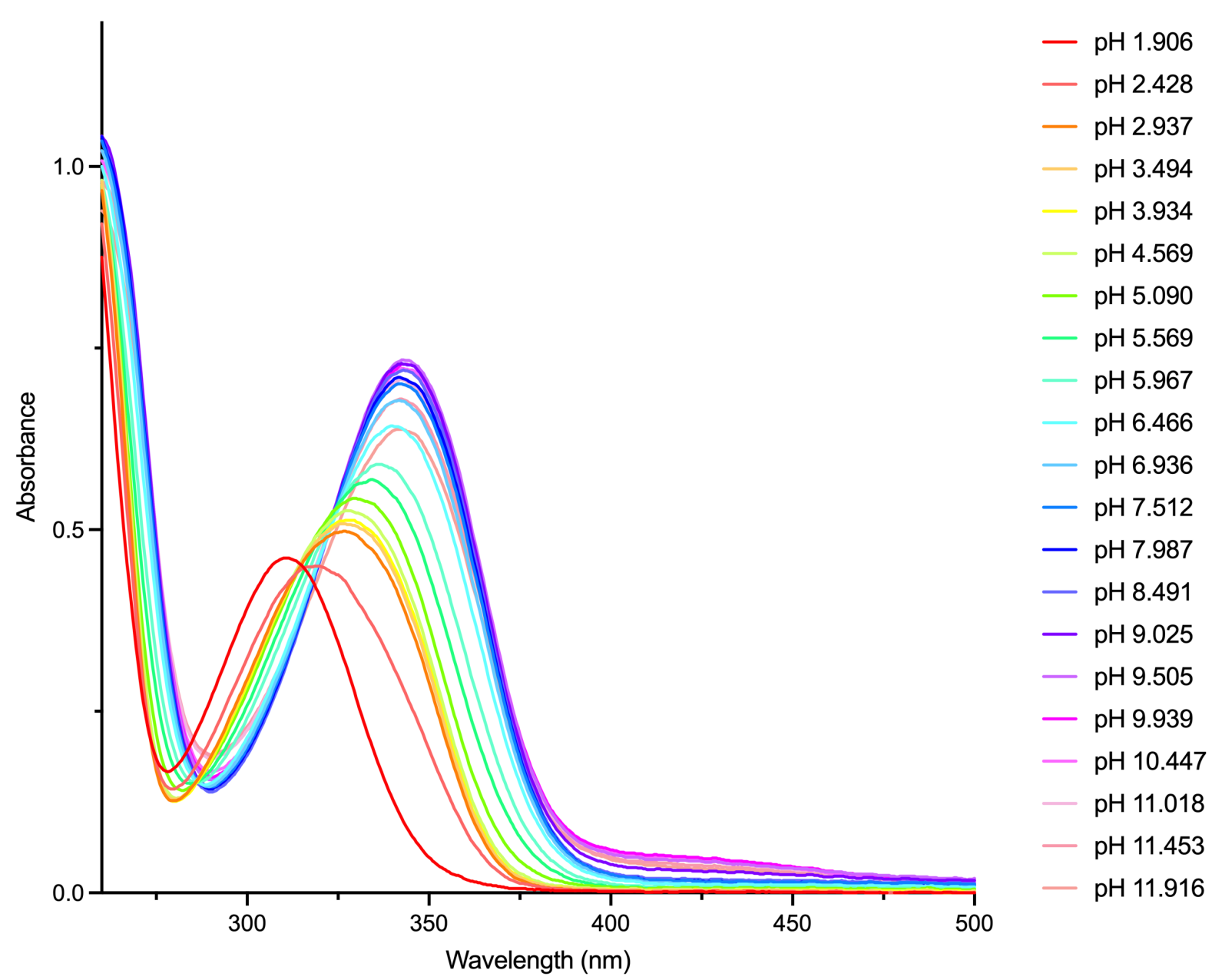

Figure S132. pH-dependent UV-vis spectra of **[Ga(OK-COOH)]$^{3-}$** that were used to calculate Logß values for relevant species.

Table S19. Tabulated Logß values for LH_6 = **OK-COOH**

Species	Logß
(LH_7)	69.06
$(LH_6)^-$	66.86
$(LH_5)^{2-}$	57.87
$(LH_4)^{3-}$	48.04
$(LH_3)^{4-}$	39.12
$(LH_2)^{5-}$	26.50
$(LH)^{6-}$	14.68

Table S20. Tabulated Logß values for Fe^{3+} and Ga^{3+} complexes with **OK-COOH** species discussed in this work.

Species	Logß, M = Ga^{3+}	Logß, M = Fe^{3+}
$[M(LH_6)]^{2+}$	73.6	70.2
$[M(LH_5)]^+$	68.3	67.8
$[M(LH_4)]$	62.4	62.6
$[M(LH_3)]^-$	56.1	56.3
$[M(L)]^{4-}$	30.0	31.1

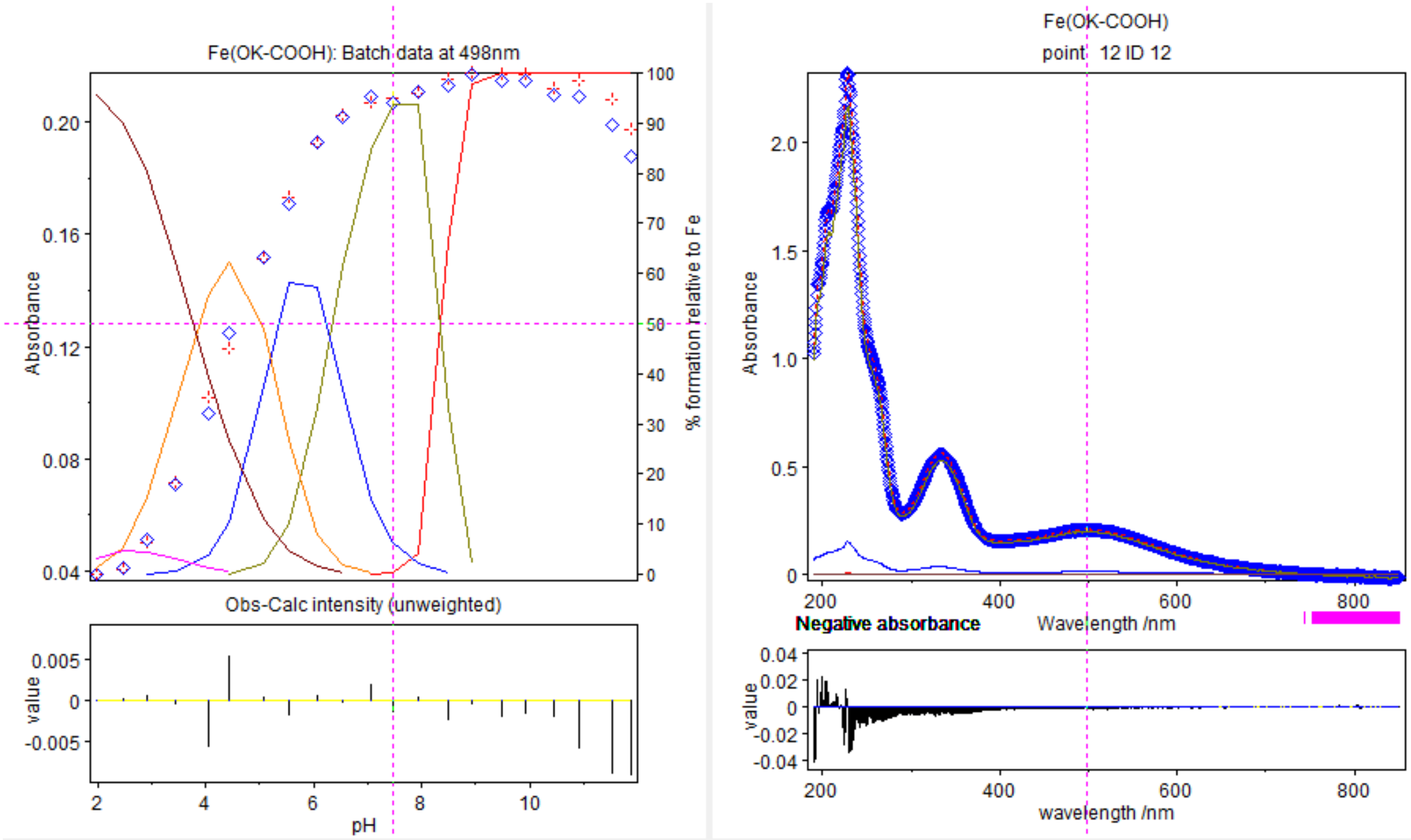

Figure S133. Representative data fit of **[Fe(OK-COOH)]**$^{3-}$ in HypSpec2014 using the LMCT peak.

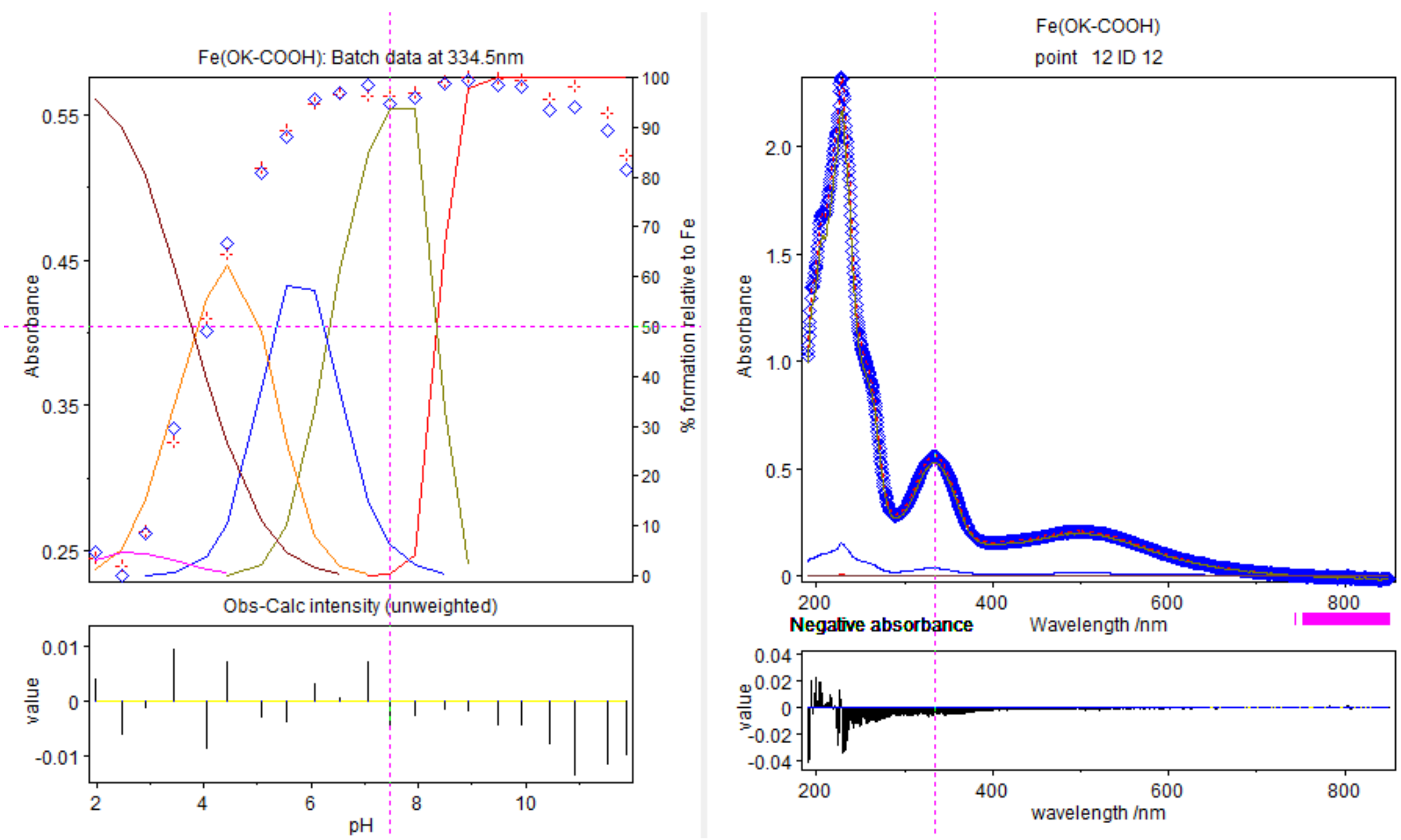

Figure S134. Representative data fit of **[Fe(OK-COOH)]**$^{3-}$ in HypSpec2014 using the catechol peak.

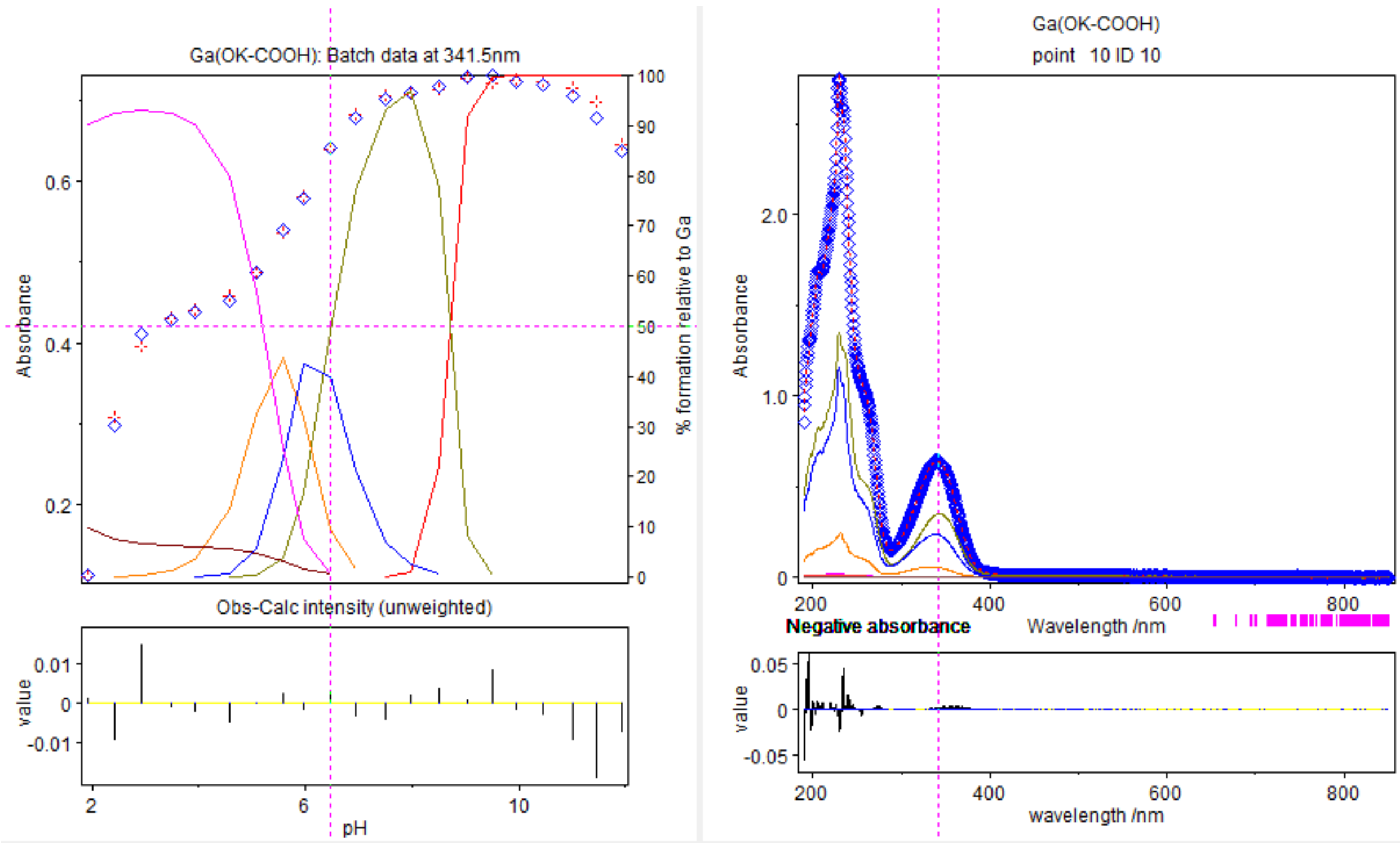

Figure S135. Representative data fit of **[Ga(OK-COOH)]**$^{3-}$ in HypSpec2014 using the catechol peak.

2. Microbiology assays

2.1 Growth promotion assay

Single colony *E. coli ATCC 33475* (ATCC) from plate was incubated in 5 mL LB medium supplemented with 10 μg/mL streptomycin at 37°C with shaking at 210 rpm overnight. Next day, 5 mL overnight culture was diluted into 5 mL LB with 10 μg/mL streptomycin and incubated for 3.5 h at 37°C with shaking to reach OD_{600} = 0.6. The culture was diluted to an OD600 = 0.001 in LB with 200 μM DP. A 90 mL aliquot of the diluted culture was mixed with a 10-mL aliquot of a 10x solution of siderophore in 96-well plate which was sealed with permeable microplate sealing and incubated at 37°C with shaking 282 cpm for 18 h in Biotek Synergy HTX multi-mode plate reader. The growth was recorded every 30 min to reconstruct the growth behavior with exogenous siderophore as well as relative growth compared to enterobactin. Assay was repeated in triplicates per day and three biological independent replicates on different days. The resulting mean OD_{600} are reported, and the error bar are the standard deviation of the mean obtained from the three independent replicates.[11,12]

2.2 Antimicrobial activity assay (MIC_{98})

Antibacterial activity of ligands, conjugates, and their corresponding Fe^{3+} and Ga^{3+} complexes was determined by measuring their minimum inhibitory concentrations (MICs) using the broth microdilution method according to the Clinical and Laboratory Standards Institute (CLSI) guidelines. All aqueous solutions and media were prepared using deionized water. All liquids and media were sterilized by autoclaving (220 °C, 1 h) before use. Iron deficient, cation adjusted Mueller–Hinton broth (MHB) was used for these assays. 5.25 g of MHB was added to 250 mL of deionized water. Aqueous solutions of 1 M Ca^{2+} (0.418 mL) and 1 M Mg^{2+} (0.155 mL) to 250 mL of MHB were added to the broth. The broth was autoclaved for 1 h at 220 °C. To make the broth iron-deficient, 4.06 mL of 1 mg mL^{-1} sterile (autoclaved) aqueous solution of 2,2′-bipyridine (DP) was added to 250 mL of cation adjusted MHB.

In general, 0.3 mM stock solutions of testing compounds were prepared using UV-Vis and ICP-OES to obtain the concentrations. 10 μL solution of testing compound was added to the first well of the 96-well plate and serial dilutions were made down each row. 40 μL of growth media and 50 μL of diluted bacterial inoculum was also added to each well, resulting in a total volume of 100 μL and a concentration gradient of 0.3×10^{-4} M to 0.92×10^{-12} M. The plates were incubated at 37 °C for 18 h and each plate was examined for bacterial growth using a plate reader (Biotek Synergy HTX multi-mode plate reader). The MIC_{98} was recorded as the lowest compound concentration (μM) required to inhibit 98% of bacterial growth as determined by the absorbance of the culture media relative to the negative control. MIC_{98} experiments were performed in triplicate on three separate occasions, giving n = 9 for each reported MIC_{98} value and ensuring a maximum error margin of < 5%.

For growth-curve analysis, plates were prepared as described above. Each plate was sealed with a breathable microplate seal and incubated at 37 °C for 24 h in a BioTek Synergy HTX multimode plate reader. Bacterial growth was recorded every 30 min to reconstruct growth behavior in the presence of antibiotic conjugates.

2.2.1. E. coli K12 MIC experiments for ligands

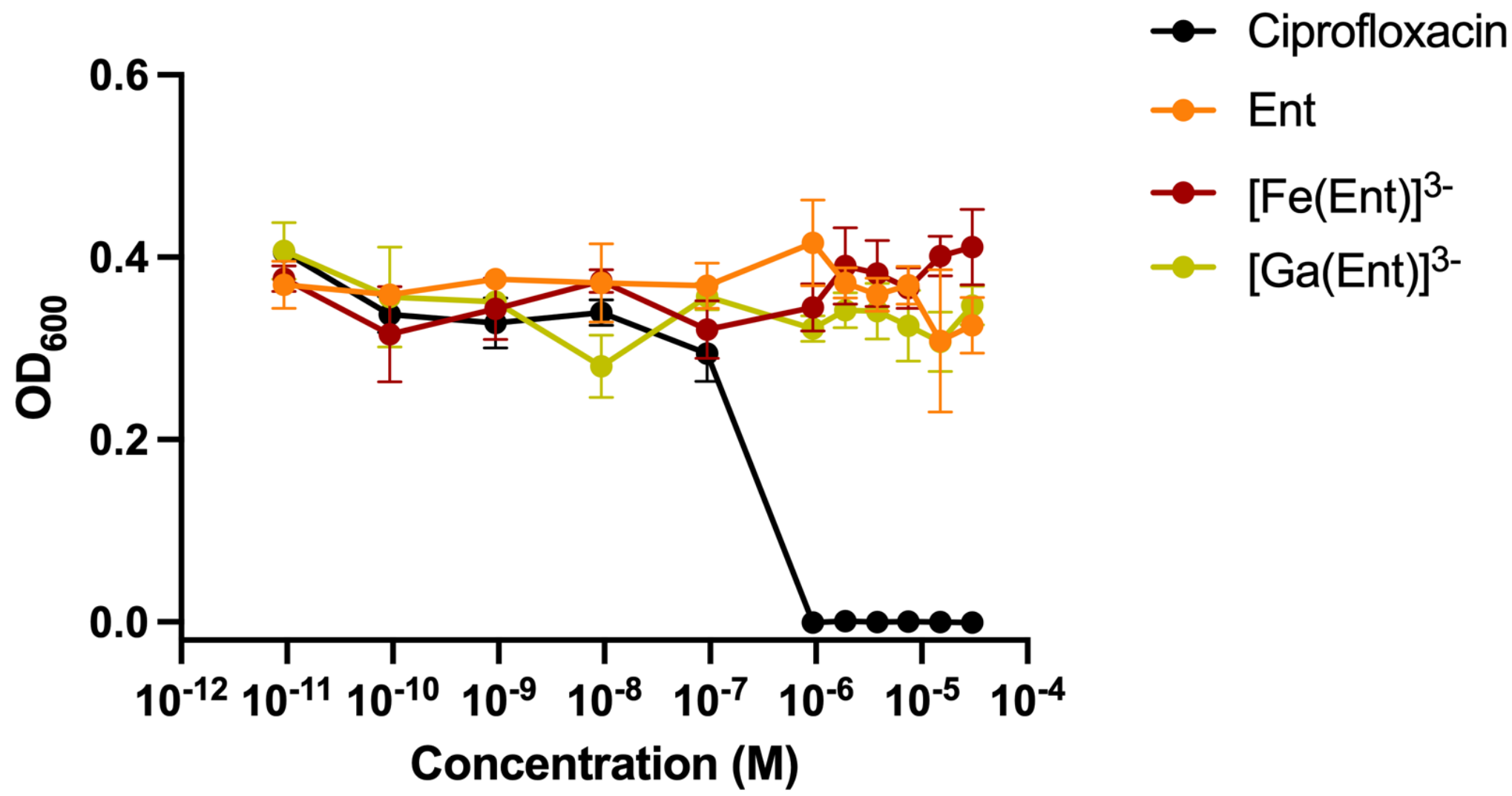

Figure S136. MIC assay of **Ent** in *E. coli K12* (n = 9).

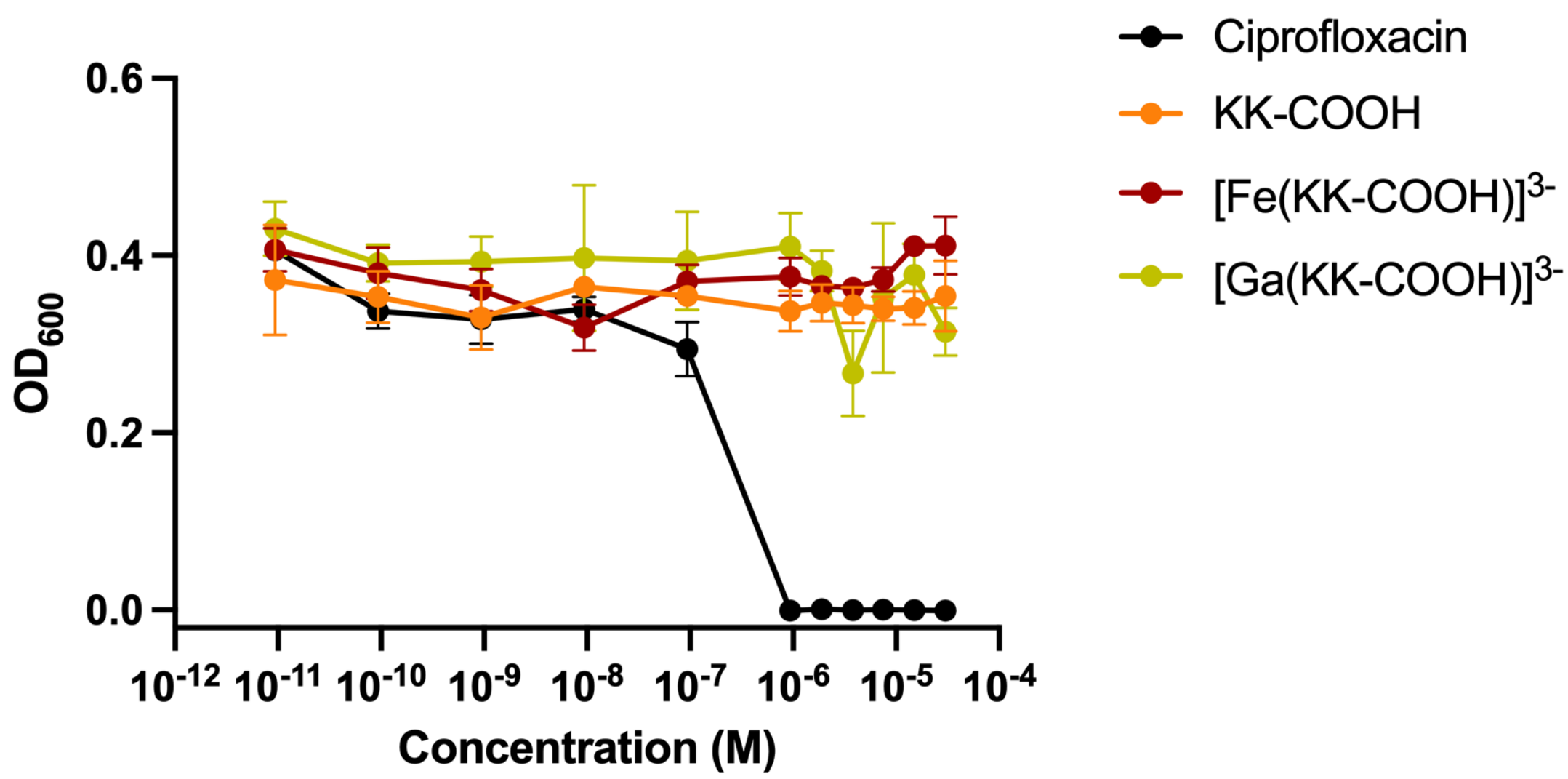

Figure S137. MIC assay of **KK-COOH** in *E. coli K12* (n = 9).

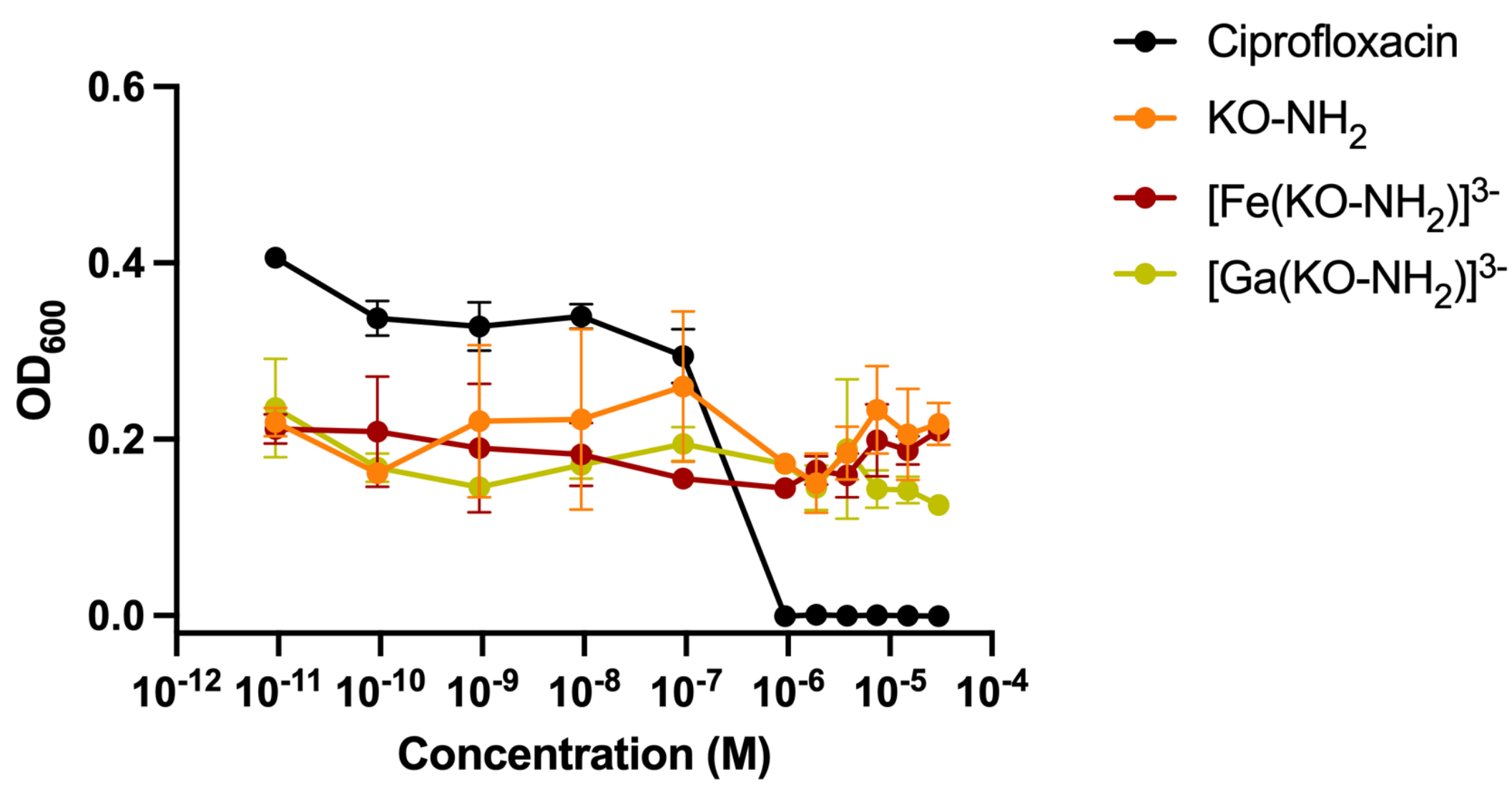

Figure S138. MIC assay of **KO-NH₂** in *E. coli K12* (n = 9).

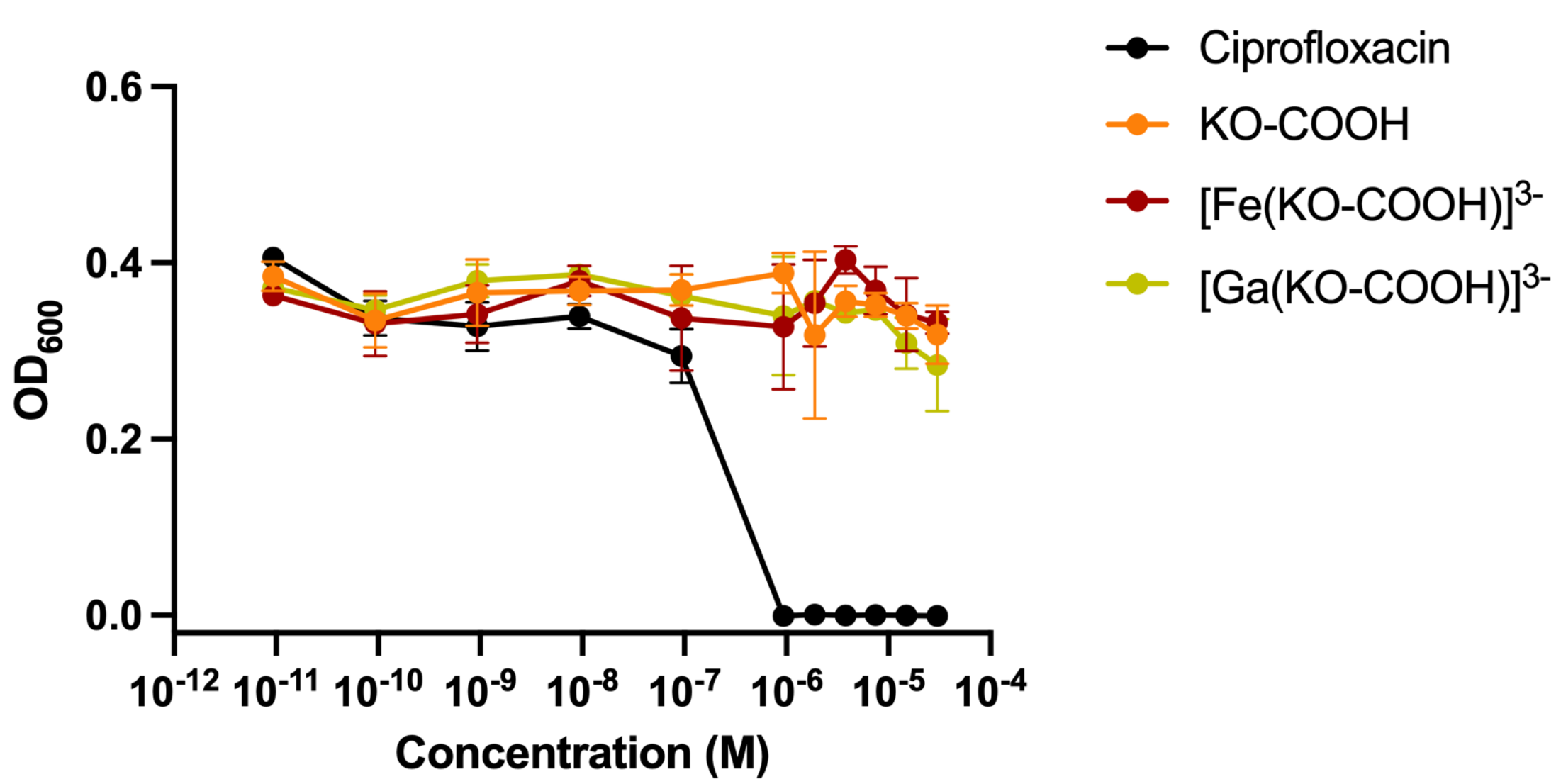

Figure S139. MIC assay of **KO-COOH** in *E. coli K12* (n = 9).

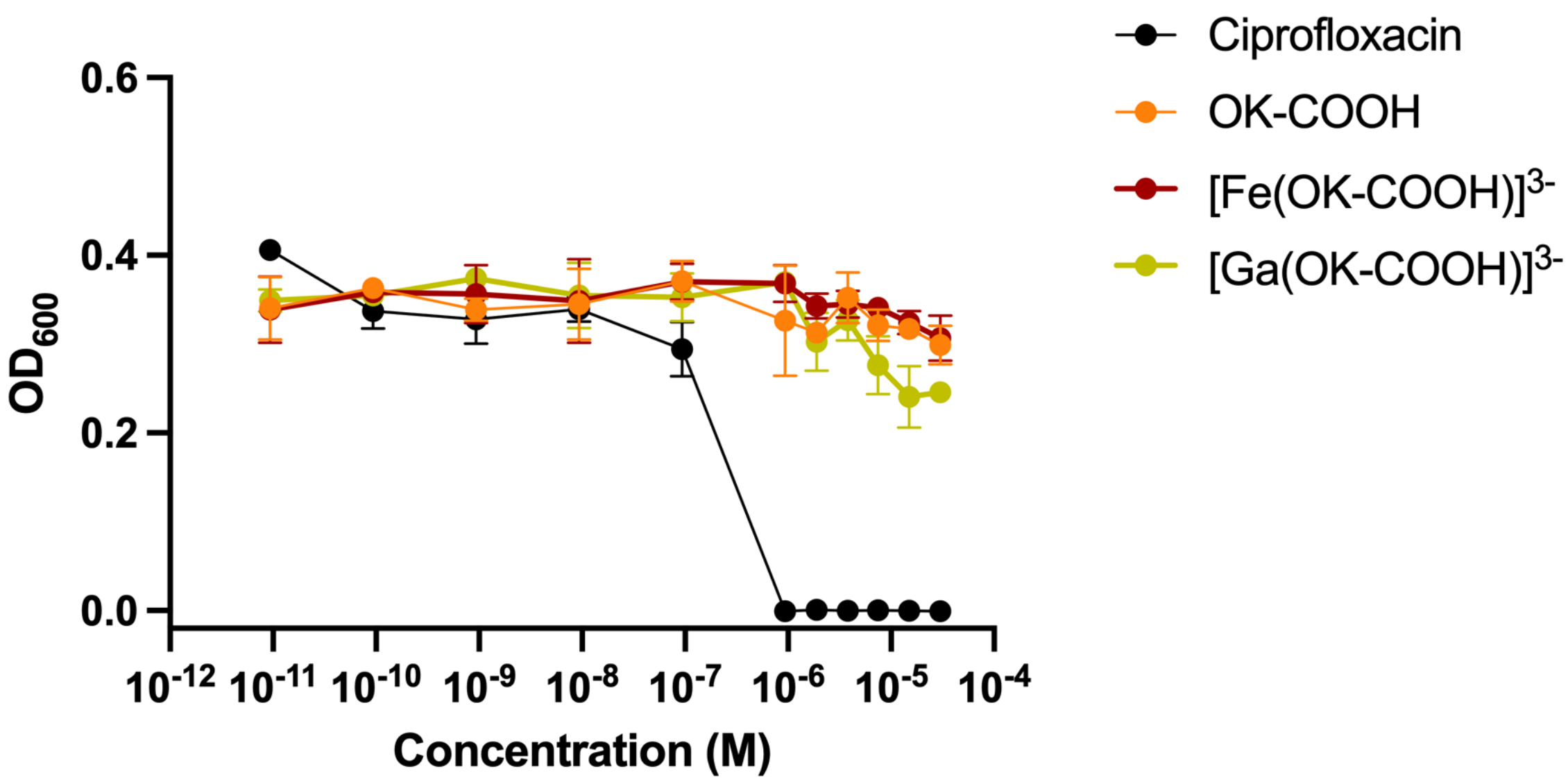

Figure S140. MIC assay of **OK-COOH** in *E. coli K12* (n = 9).

2.2.2. E. coli (K12, mutants) MIC and growth curves for conjugates

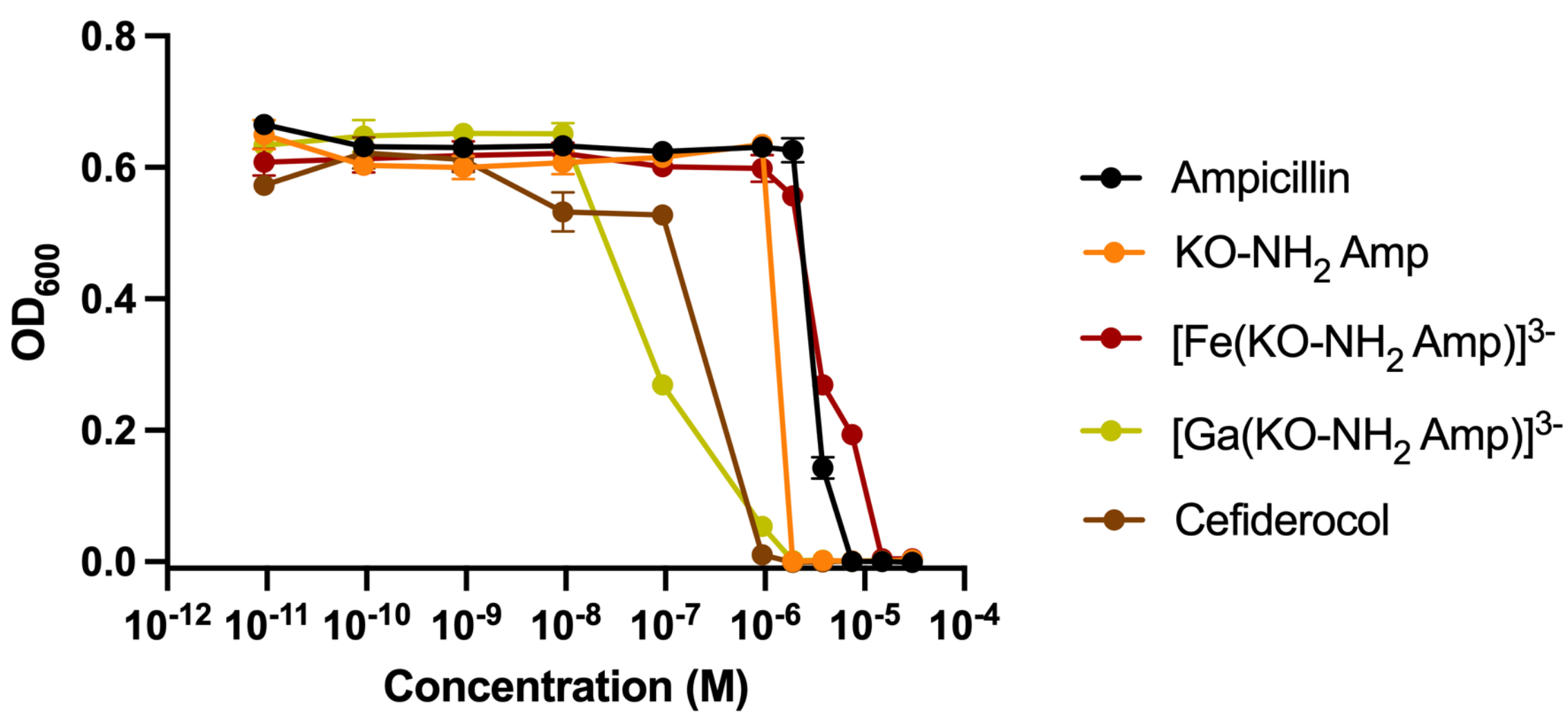

Figure S141. MIC assay **KO-NH_2-Amp** in *E. coli K12* (n = 9).

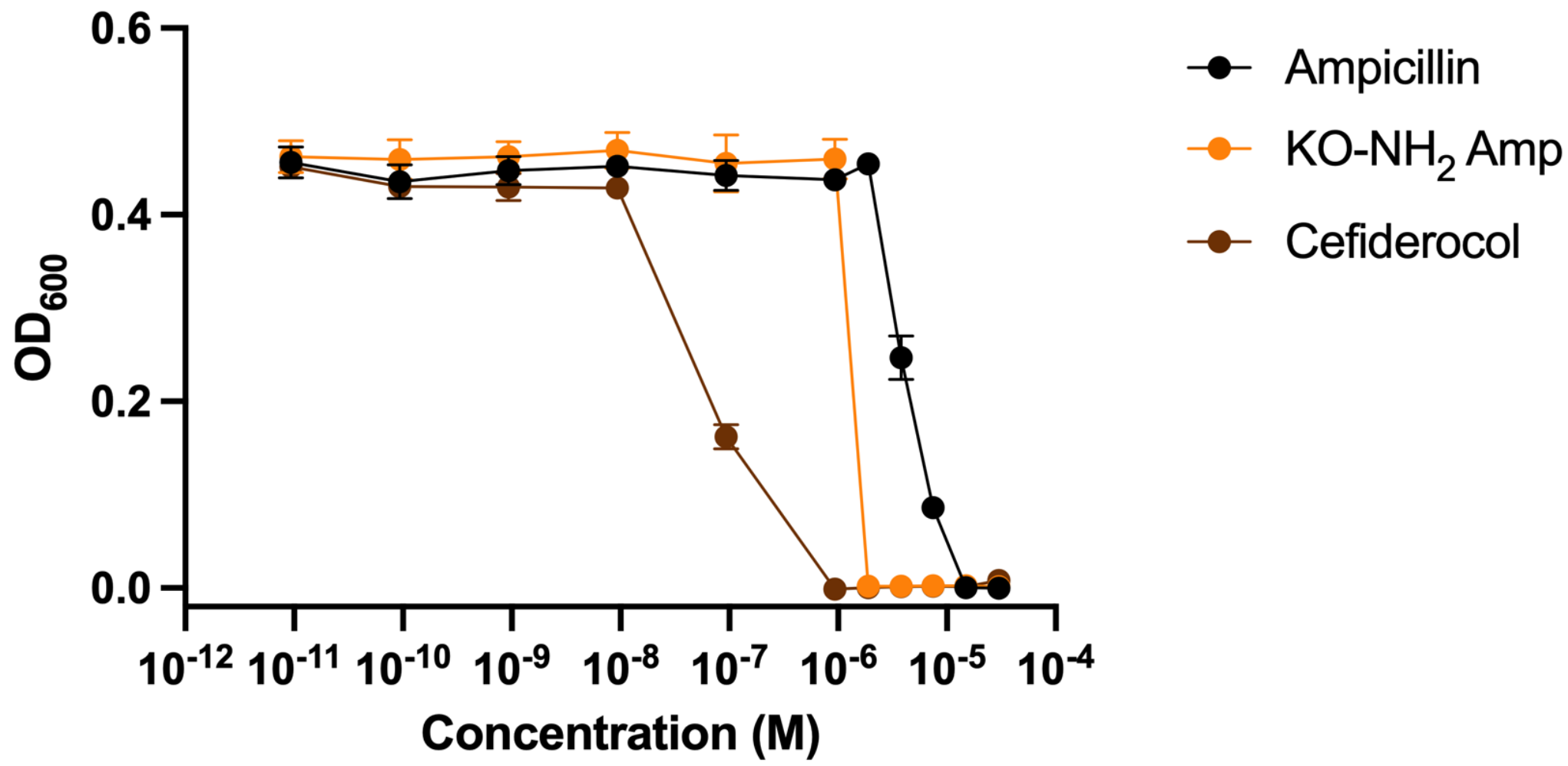

Figure S142. MIC assay **KO-NH₂-Amp** and **cefiderocol** in *E. coli ΔfepA* (n = 9).

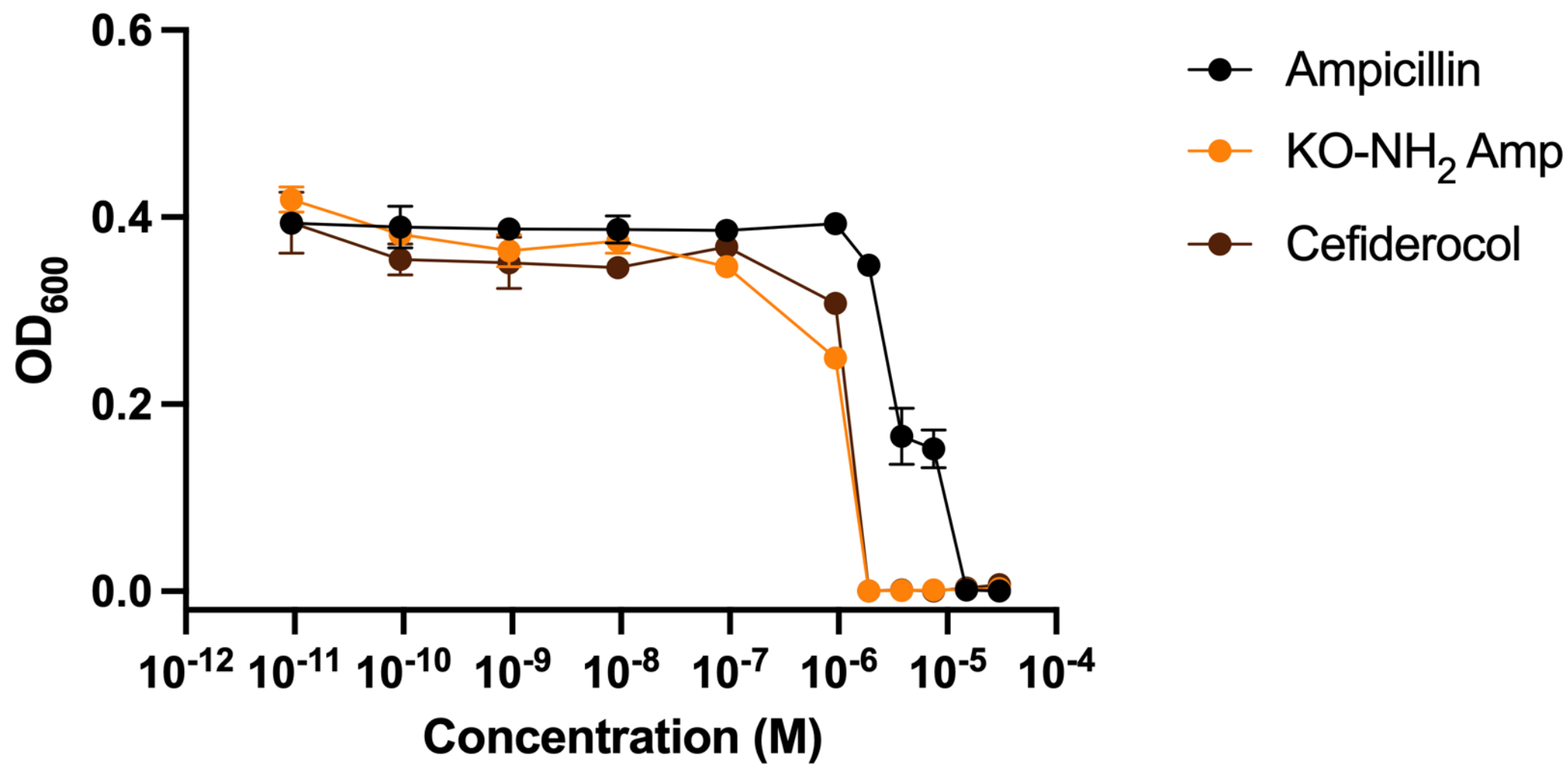

Figure S143. MIC assay **KO-NH₂-Amp** and **cefiderocol** in *E. coli ΔcirA* (n = 9).

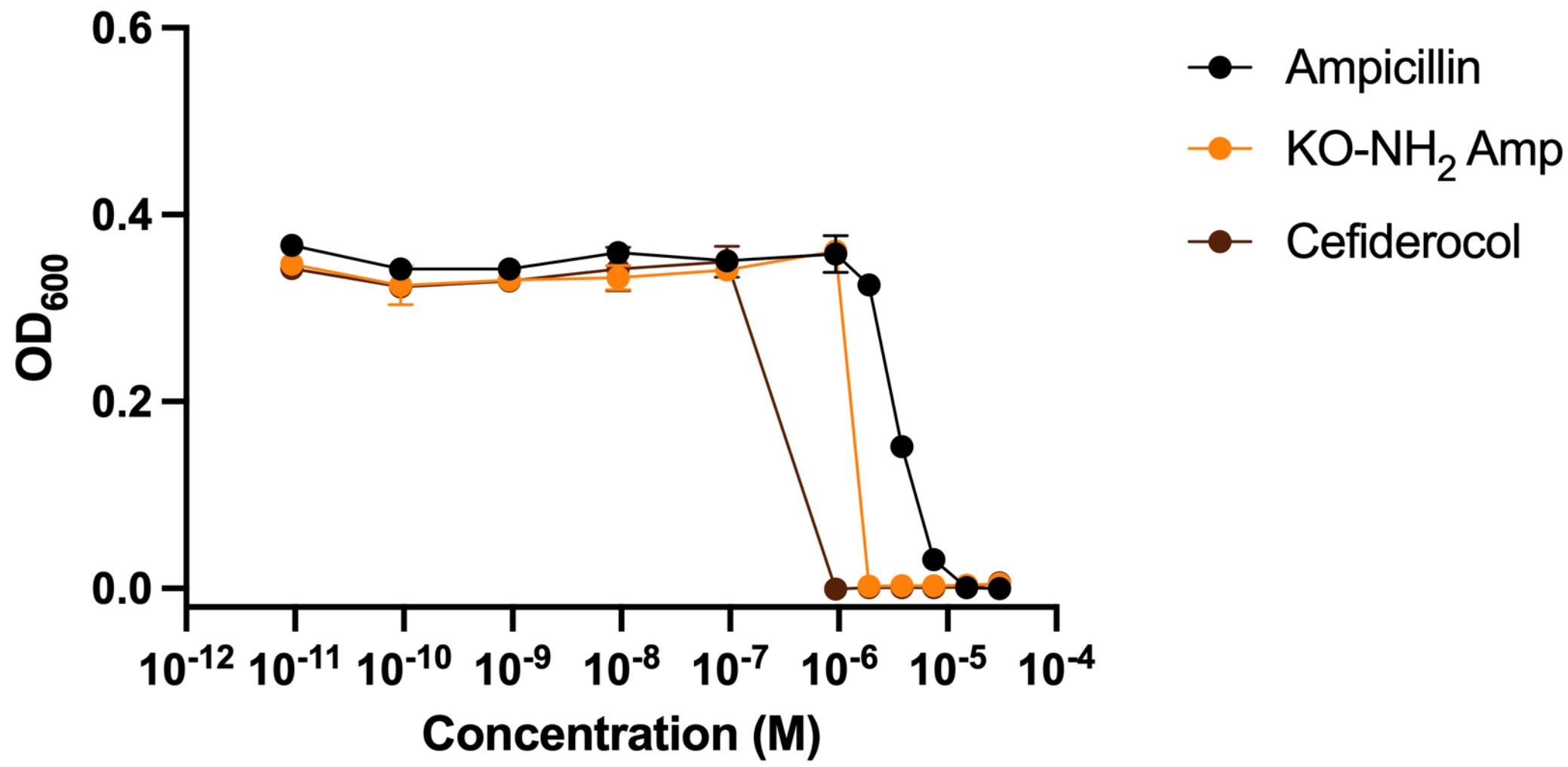

Figure S144. MIC assay **KO-NH$_2$-Amp** and **cefiderocol** in *E. coli Δfiu* (n=9).

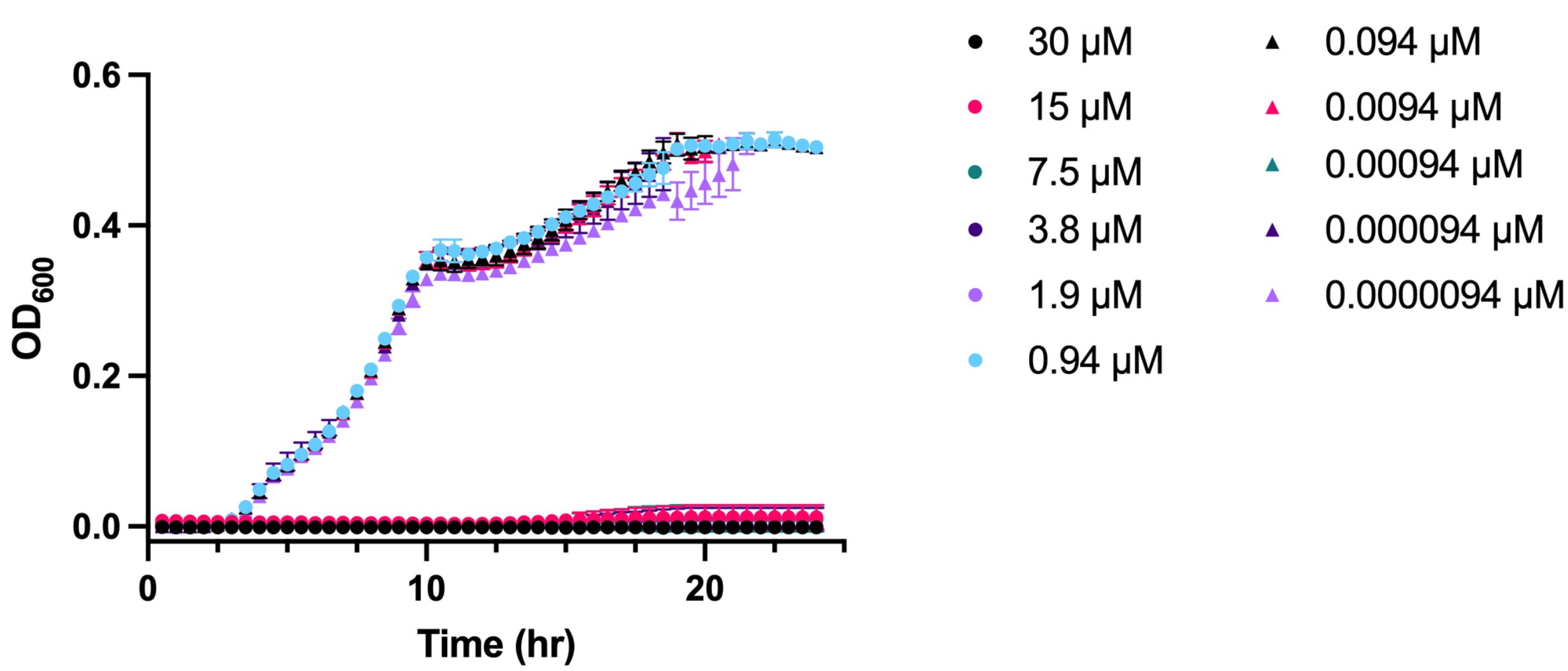

Figure S145. Growth curve of **KO-NH$_2$-Amp** in *E. coli K12* (n = 3).

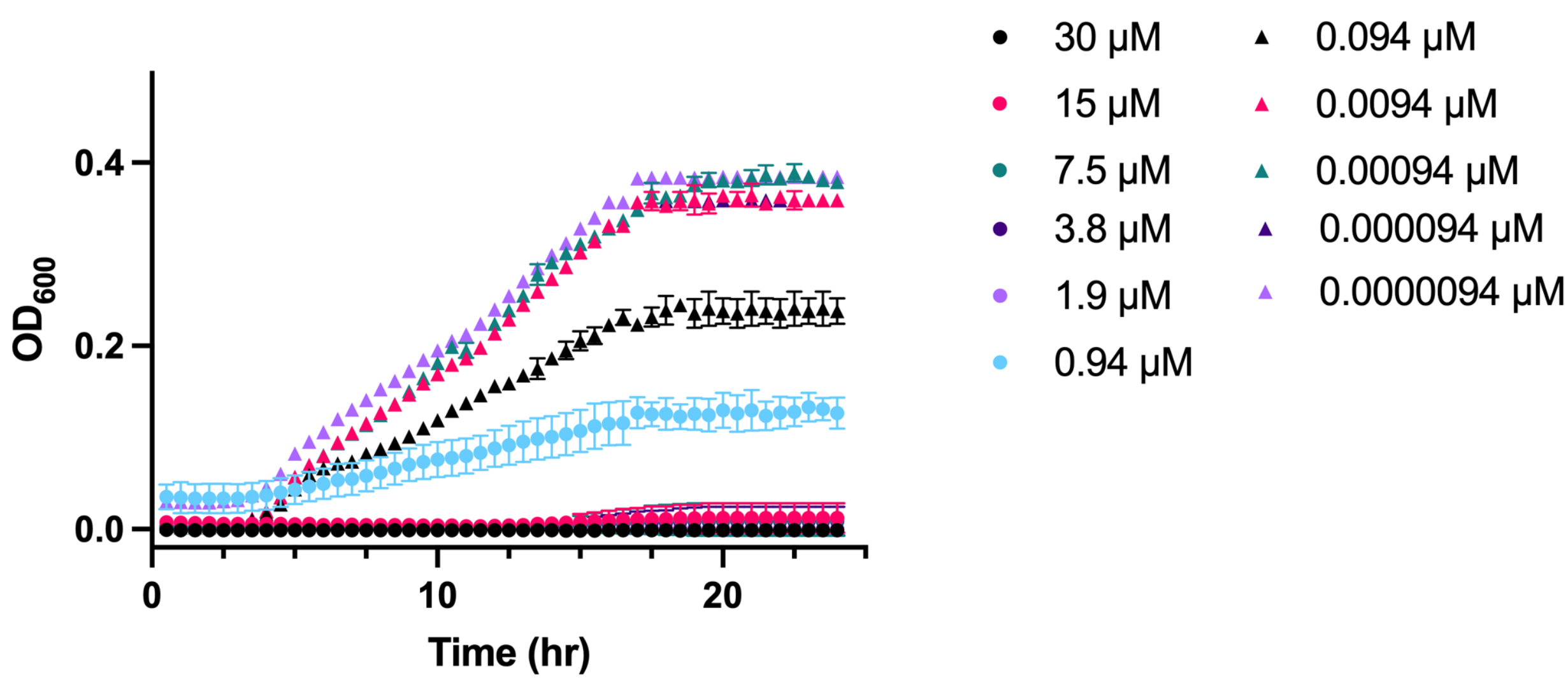

Figure S146. Growth curve of **[Ga(KO-NH$_2$-Amp)]$^{3-}$** in *E. coli K12* (n = 3).

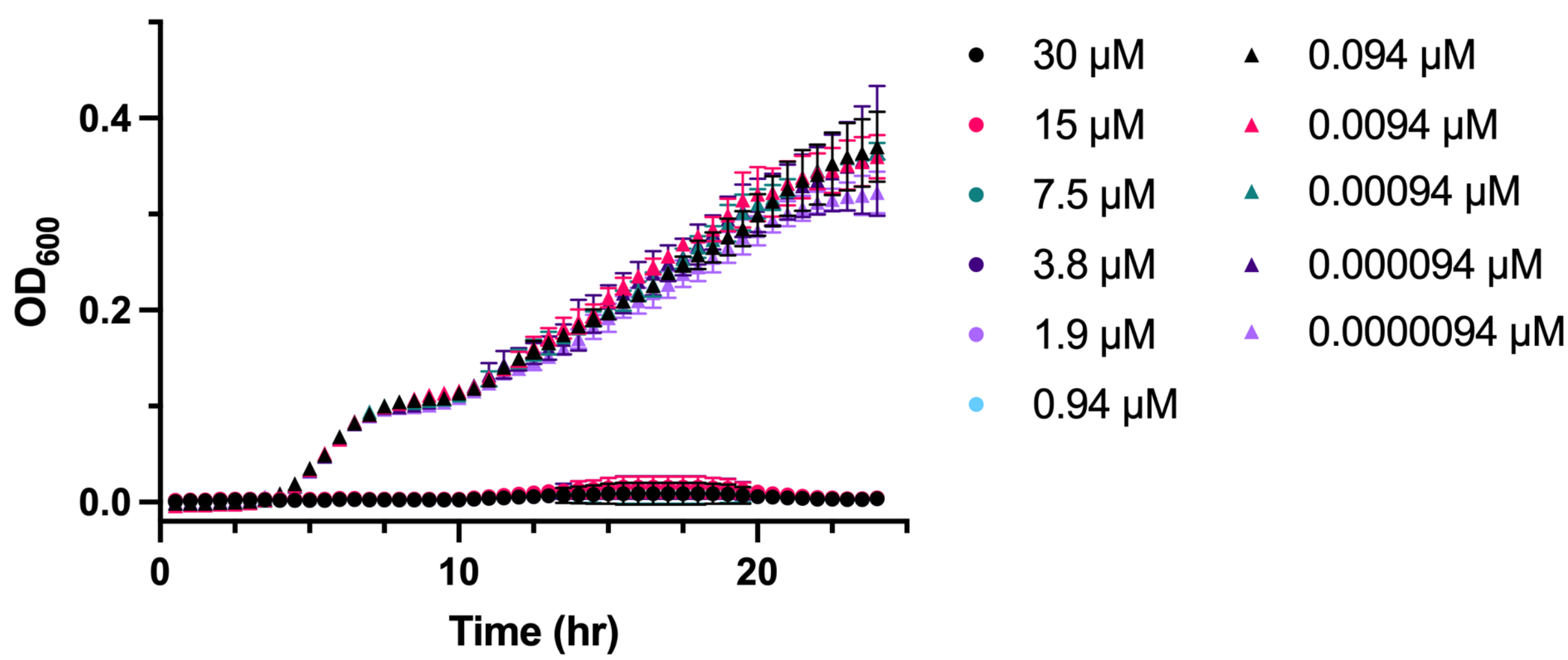

Figure S147. Growth curve of **cefiderocol** in *E. coli K12* (n = 3).

2.2.3. PAO1 MIC experiments for ligands

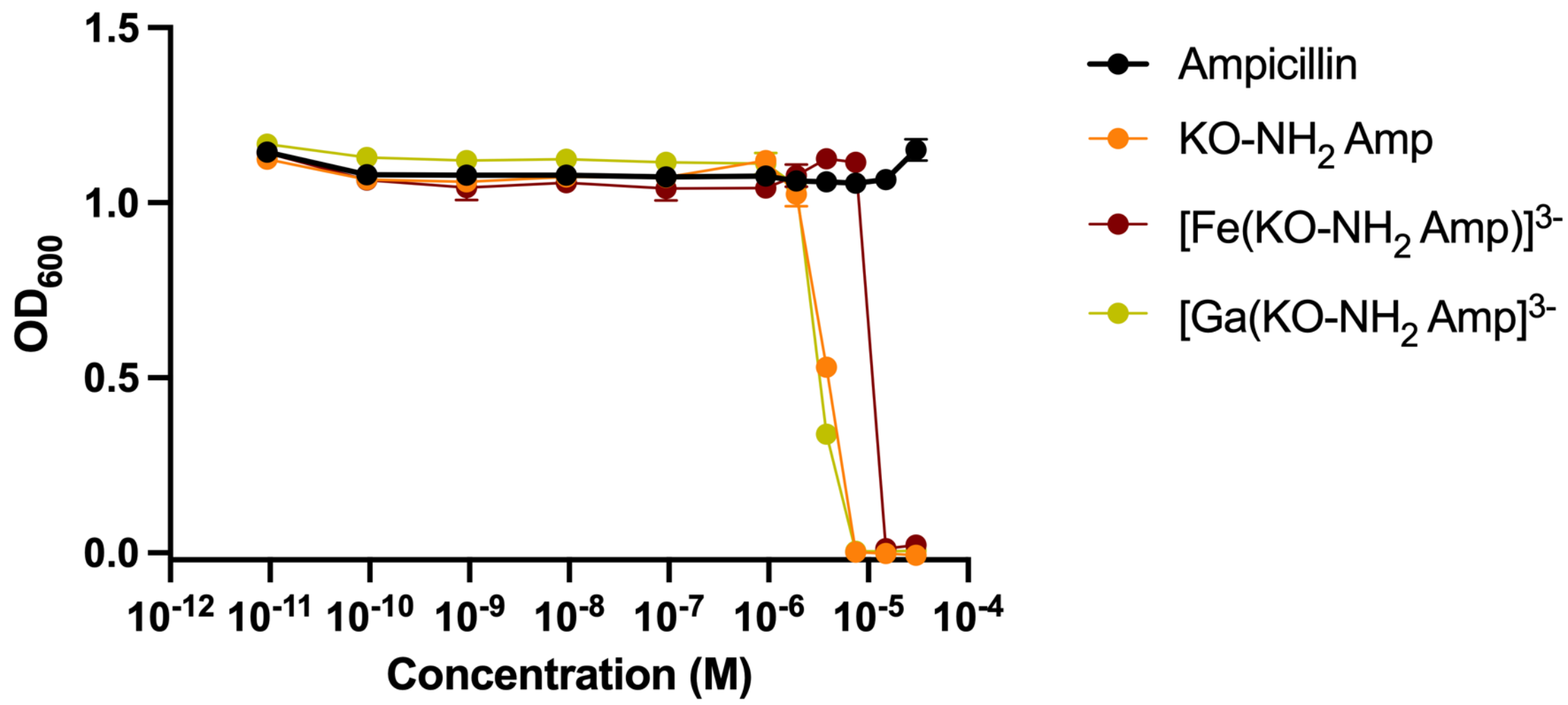

Figure S148. MIC assay **KO-NH₂-Amp** in *PAO1* (n = 9).

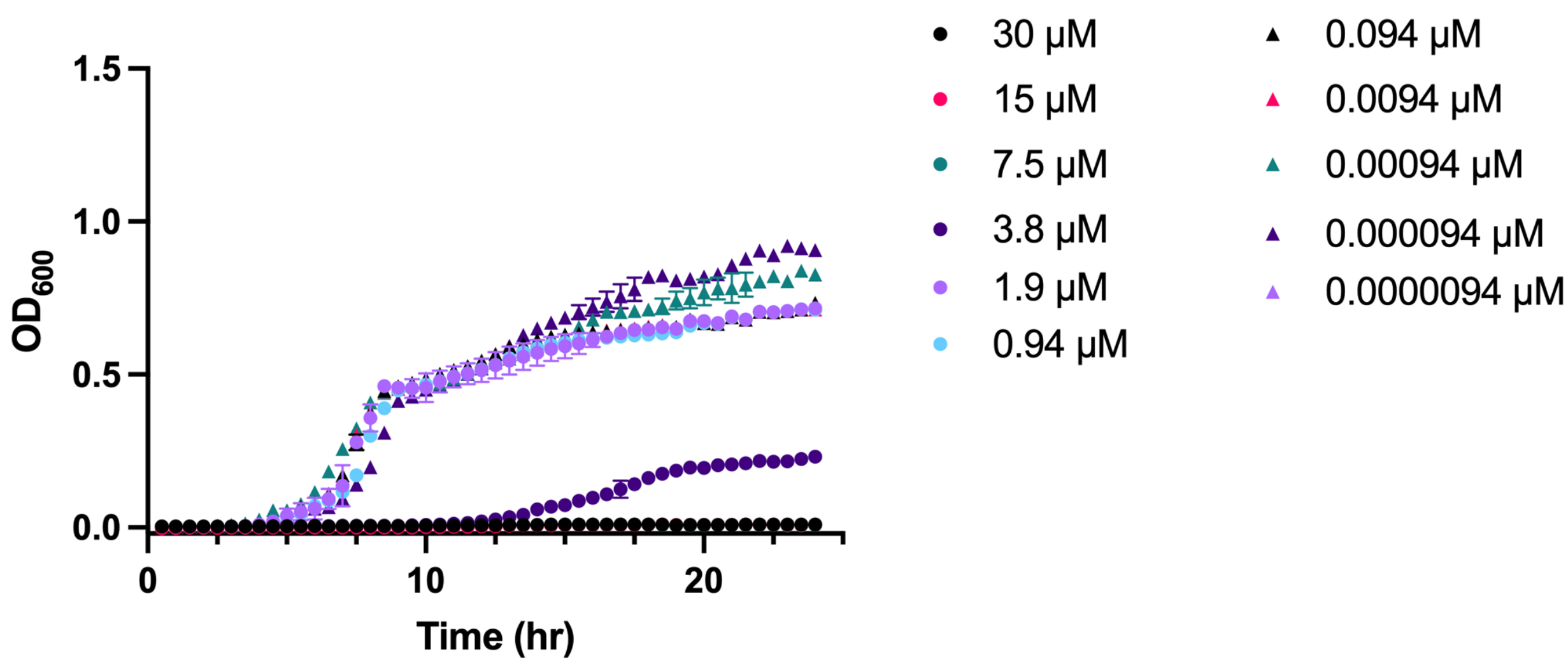

Figure S149. Growth curve of **KO-NH₂-Amp** in *PAO1* (n = 3).

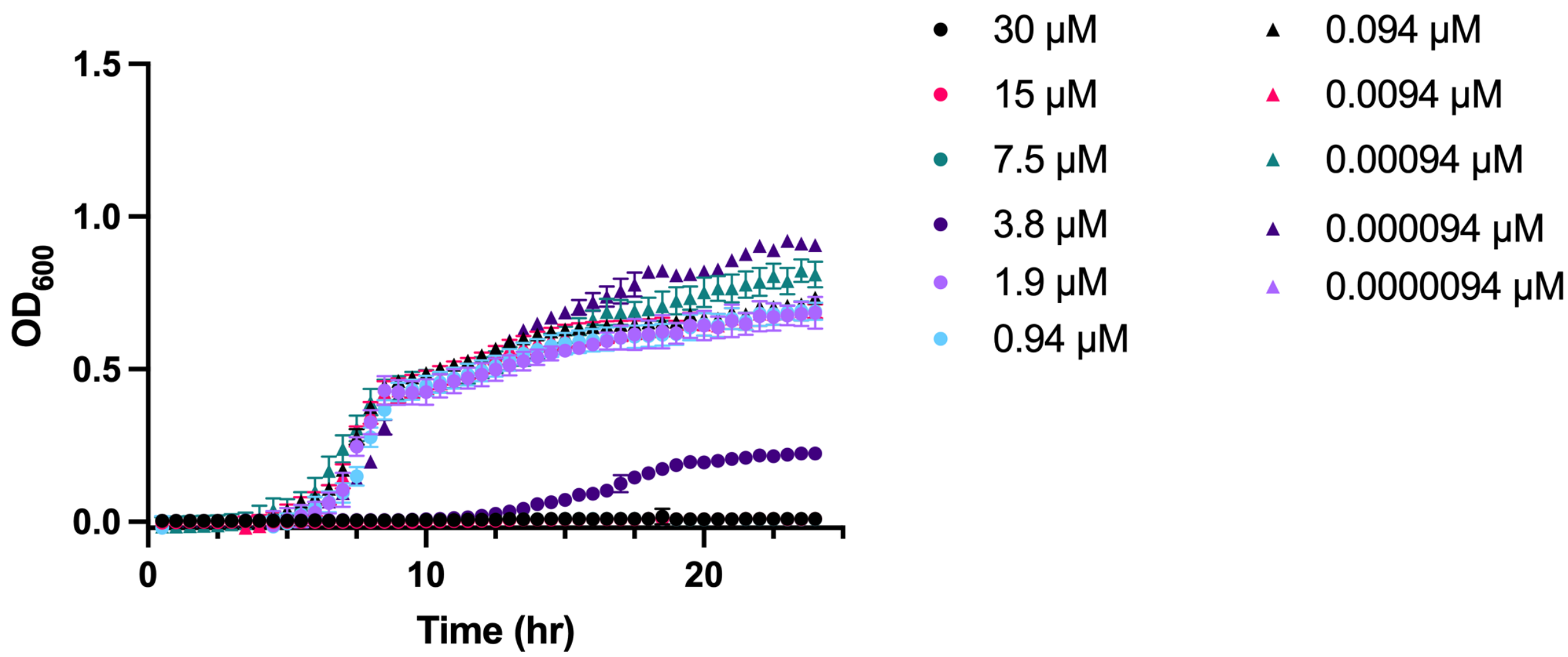

Figure S150. Growth curve of **[Ga(KO-NH$_2$-Amp)]$^{3-}$** in *PAO1* (n = 3).

2.3 Bacterial uptake using ^{67}Ga-based complexes

^{67}Ga-citrate was converted to $^{67}GaCl_3$. ^{67}Ga-complexes of siderophore were formed under mild radiolabeling conditions (pH 7.4, 50 mM HEPES, 1 min) quantitatively with ligand concentration of 1 mM before the study. *E. coli* (4.8 × 10^8 CFU) was incubated with 25 µCi of ^{67}Ga-complex at 37 °C. Aliquots were removed at 0.16, 0.33, 0.5, 1, and 2 h following initial incubation, and centrifuged for 3 min (14 500 rpm). The supernatant was removed, and the pellet was washed with DPBS (3x, 500 µL). The assay was performed in five replicates. All tubes were counted using an automated Liquid Scintillation Counter (PerkinElmer, Tri-Carb 2900TR) to quantify retained radioactivity in pellets in comparison to the 1 mL bacteria inoculum.

Uptake was expressed as the % radioactivity found in the pellet:

$$\% \, uptake = \frac{cpm \; (pellet)}{cpm \; (1 \; mL \; bacterial \; inoculum)}$$

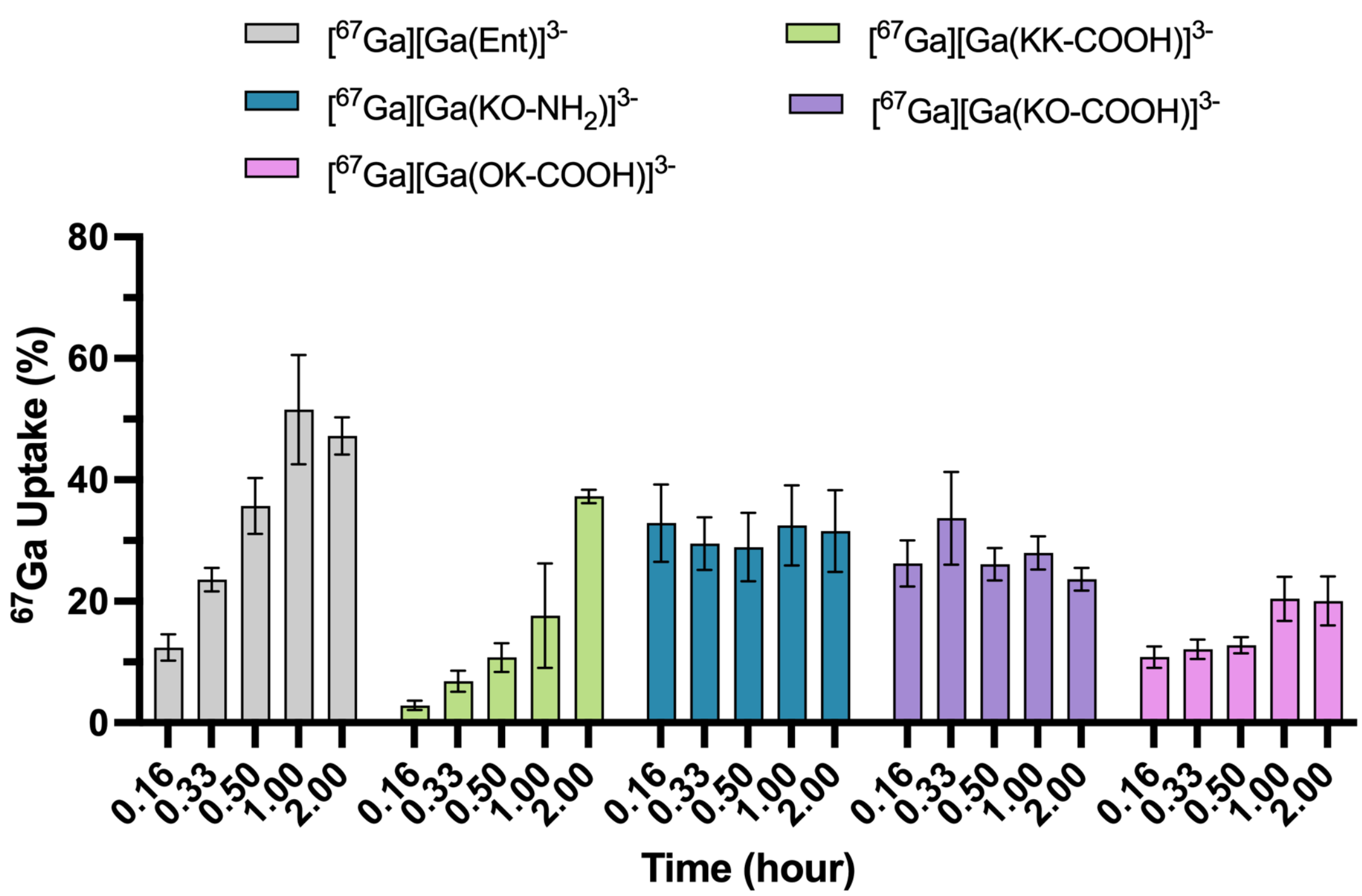

Figure S151. Time-dependent, radiochemical bacterial uptake studies in *E. coli K12* of **[^{67}Ga][Ga-complexes]$^{3-}$** (25 µCi) in iron-depleted, DP treated media pH 7.4.

Table S21. Percentage of internalized **[^{67}Ga][Ga-complexes]$^{3-}$** in *E. coli K12* in iron deficient bacterial culture (n = 5).

Incubation time (h)	**[^{67}Ga][Ga(Ent)]$^{3-}$** (%)	**[^{67}Ga][Ga(KK-COOH)]$^{3-}$** (%)	**[^{67}Ga][Ga(KO-NH$_2$)]$^{3-}$** (%)	**[^{67}Ga][Ga(KO-COOH)]$^{3-}$** (%)	**[^{67}Ga][Ga(OK-COOH)]$^{3-}$** (%)
0.16	12.399 ± 2.170	1.851 ± 0.767	32.869 ± 6.373	26.255 ± 3.397	10.809 ± 1.580
0.33	23.576 ± 1.720	6.853 ± 1.721	29.504 ± 4.318	33.681 ± 6.811	12.080 ± 1.442
0.5	35.713 ± 4.098	10.735 ± 2.362	28.925 ± 5.648	26.095 ± 2.369	12.752 ± 1.200
1	51.555 ± 8.037	17.646 ± 8.620	32.502 ± 6.573	27.984 ± 2.438	20.404 ± 3.258
2	47.258 ± 3.063	37.290 ± 1.107	31.551 ± 6.723	23.624 ± 1.659	20.060 ± 3.607

Table S22. Time-dependent, radiochemical bacterial uptake studies when challenged with 200x **[Fe(Ent)]$^{3-}$** in *E. coli* K12 of **[^{67}Ga][Ga-complexes]$^{3-}$** in iron-depleted, DP treated media pH 7.4.

Incubation time (h)	**[^{67}Ga][Ga(Ent)]$^{3-}$** (%)	**[^{67}Ga][Ga(KK-COOH)]$^{3-}$** (%)	**[^{67}Ga][Ga(KO-NH$_2$)]$^{3-}$** (%)	**[^{67}Ga][Ga(KO-COOH)]$^{3-}$** (%)	**[^{67}Ga][Ga(OK-COOH)]$^{3-}$** (%)
1	0.436 ± 0.079	4.860 ± 1.716	1.249 ± 0.098	2.209 ± 0.393	1.942 ± 0.266
2	0.441 ± 0.130	6.339 ± 1.564	1.658 ± 0.225	5.466 ± 1.093	4.655 ± 0.852

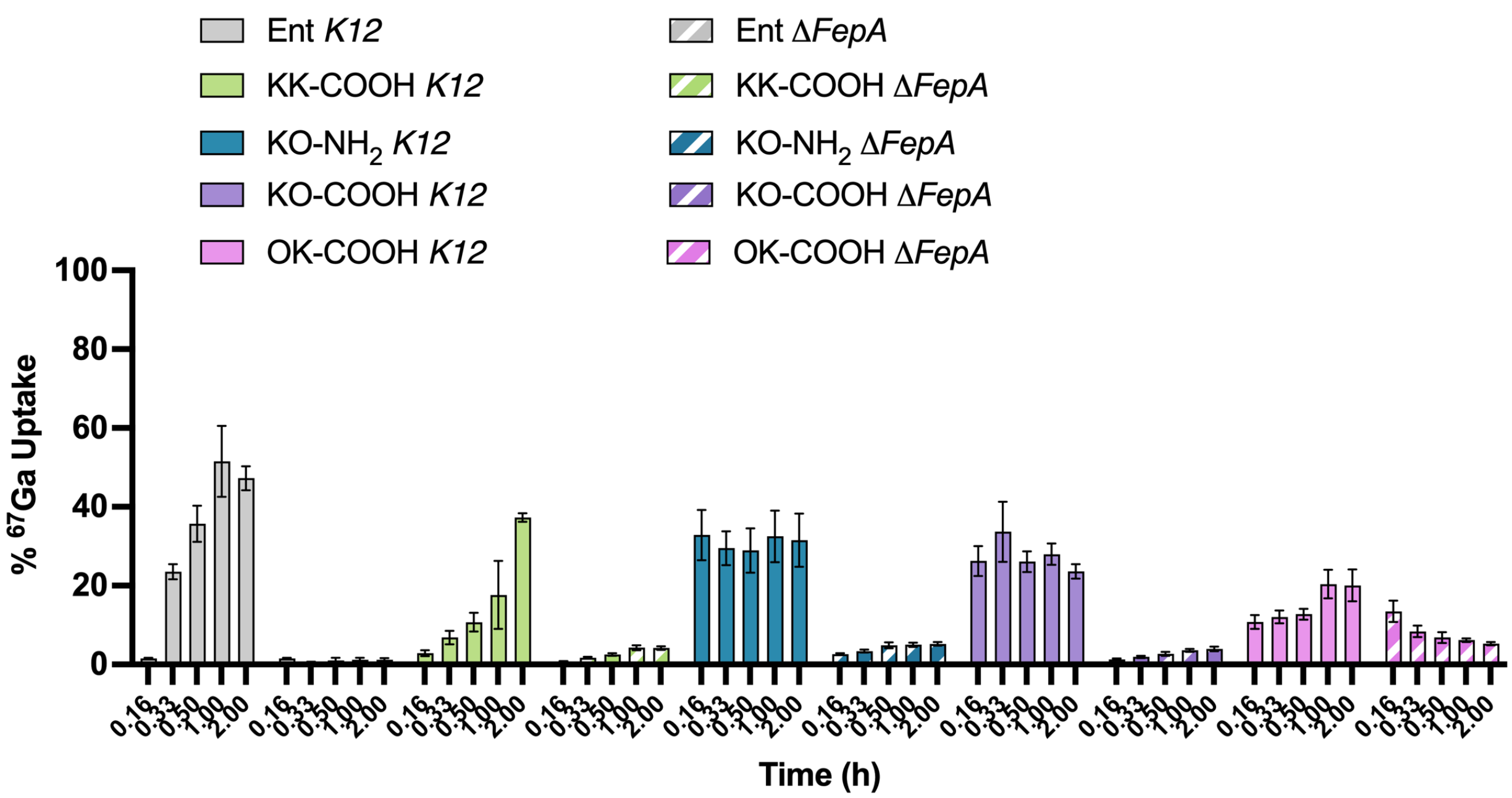

Figure S152. Time-dependent, radiochemical bacterial uptake studies in *E. coli ΔfepA* of **[^{67}Ga][Ga-complexes]$^{3-}$** (25 µCi) in iron-depleted, DP treated media pH 7.4.

Table S23. Percentage of internalized **[^{67}Ga][Ga-complexes]$^{3-}$** in *E. coli ΔfepA* upon incubation in iron deficient bacterial culture (n = 5).

Incubation time (h)	**[^{67}Ga][Ga(Ent)]$^{3-}$** (%)	**[^{67}Ga][Ga(KK-COOH)]$^{3-}$** (%)	**[^{67}Ga][Ga(KO-NH$_2$)]$^{3-}$** (%)	**[^{67}Ga][Ga(KO-COOH)]$^{3-}$** (%)	**[^{67}Ga][Ga(OK-COOH)]$^{3-}$** (%)
0.16	1.541 ± 0.171	0.762 ± 0.088	2.627 ± 0.229	1.332 ± 0.174	13.496 ± 2.707
0.33	0.643 ± 0.055	1.734 ± 0.233	3.403 ± 0.350	1.929 ± 0.277	8.411 ± 1.477
0.5	1.044 ± 0.673	2.541 ± 0.338	4.855 ± 0.731	2.703 ± 0.470	6.843 ± 1.349
1	1.190 ± 0.545	4.260 ± 0.630	5.013 ± 0.550	3.591 ± 0.398	6.188 ± 0.458
2	1.234 ± 0.360	4.169 ± 0.419	5.219 ± 0.451	3.941 ± 0.590	5.314 ± 0.412

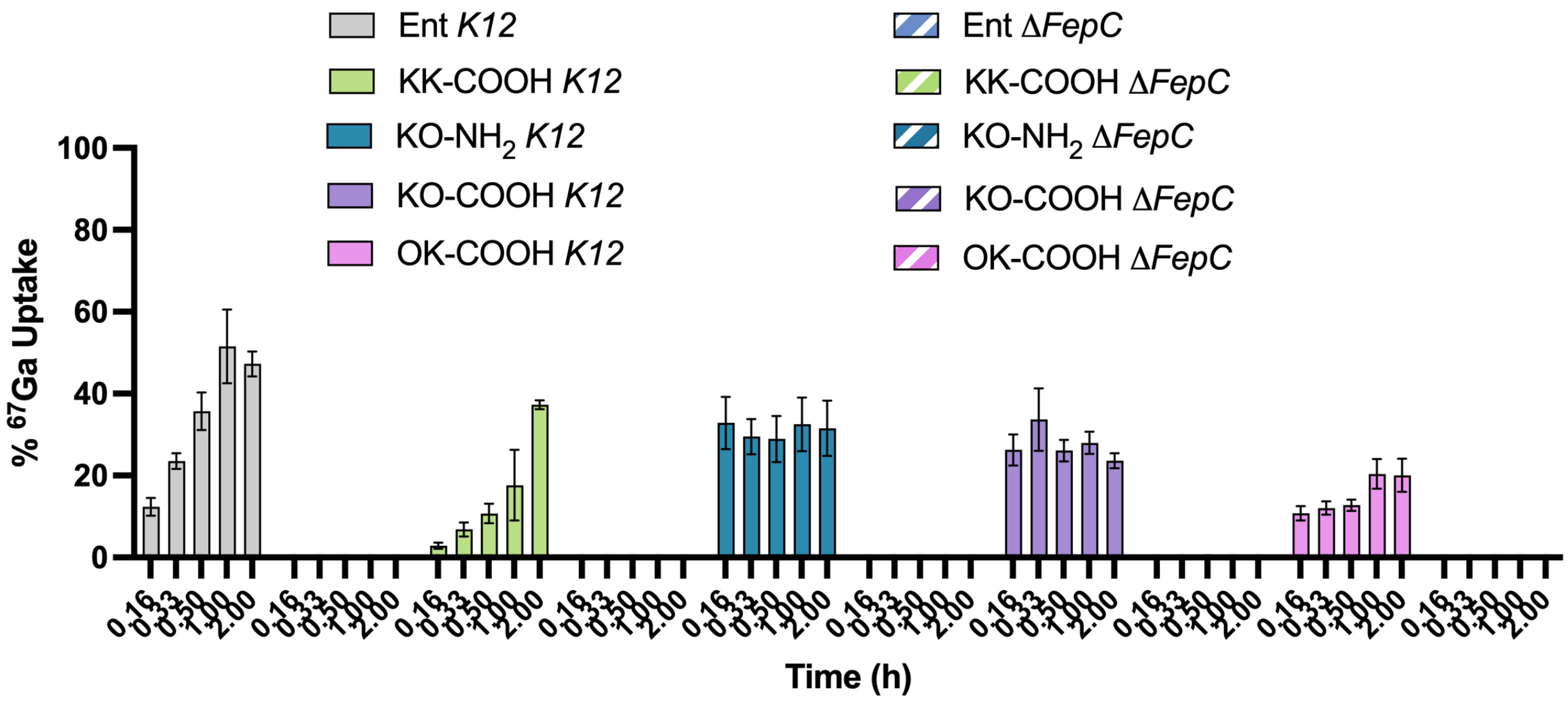

Figure S153. Time-dependent, radiochemical bacterial uptake studies in *E. coli ΔfepC* of **[^{67}Ga][Ga-complexes]$^{3-}$** (25 μCi)in iron-depleted, DP treated media pH 7.4.

Table S24. Percentage of internalized **[^{67}Ga][Ga-complexes]$^{3-}$** in *E. coli ΔfepC* upon incubation in iron deficient bacterial culture (n = 5).

Incubation time (h)	**[^{67}Ga][Ga(Ent)]$^{3-}$** (%)	**[^{67}Ga][Ga(KK-COOH)]$^{3-}$** (%)	**[^{67}Ga][Ga(KO-NH$_2$)]$^{3-}$** (%)	**[^{67}Ga][Ga(KO-COOH)]$^{3-}$** (%)	**[^{67}Ga][Ga(OK-COOH)]$^{3-}$** (%)
0.16	0.235 ± 0.033	0.762 ± 0.088	2.627 ± 0.229	0.187 ± 0.017	0.154 ± 0.028
0.33	0.329 ± 0.091	1.739 ± 0.233	3.403 ± 0.350	0.197 ± 0.023	0.183 ± 0.028
0.5	0.320 ± 0.102	2.541 ± 0.335	4.856 ± 0.717	0.375 ± 0.174	0.210 ± 0.025
1	0.312 ± 0.0518	4.260 ± 0.630	5.013 ± 0.550	0.294 ± 0.046	0.204 ± 0.032
2	0.351 ± 0.056	4.170 ± 0.419	5.219 ± 0.451	0.344 ± 0.106	0.226 ± 0.025

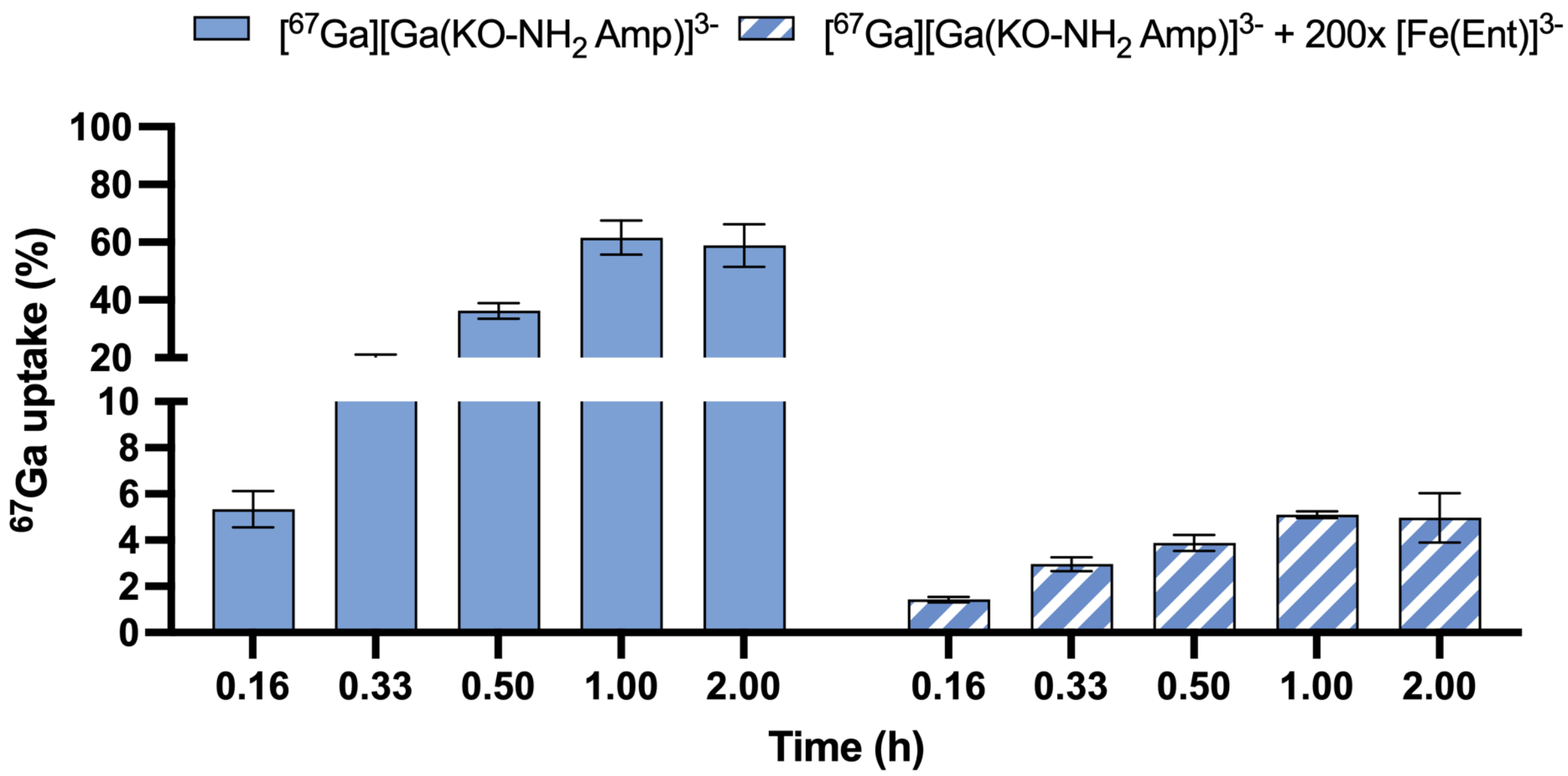

Figure S154. Time-dependent, radiochemical bacterial uptake studies in *E. coli K12* of **[^{67}Ga][Ga(KO-NH$_2$-Amp)]$^{3-}$** (25 μCi) in iron-depleted, DP treated media pH 7.4.

Time (h)	**[^{67}Ga][Ga(KO-NH$_2$-Amp)]$^{3-}$** (%)	**[^{67}Ga][Ga(KO-NH$_2$-Amp)]$^{3-}$** + 200x [Fe(Ent)]$^{3-}$ (%)
0.16	5.333 ± 0.787	1.426 ± 0.108
0.33	18.324 ± 2.771	2.957 ± 0.275
0.50	36.214 ± 2.666	3.873 ± 0.314
1	61.562 ± 5.911	5.101 ± 0.137
2	58.839 ± 7.362	4.969 ± 0.952

Table S25. Percentage of internalized of **[^{67}Ga][Ga(KO-NH$_2$-Amp)]$^{3-}$** in *E. coli K12* and when challenged with 200x [Fe(Ent)]$^{3-}$ in iron deficient bacterial culture (n=5).

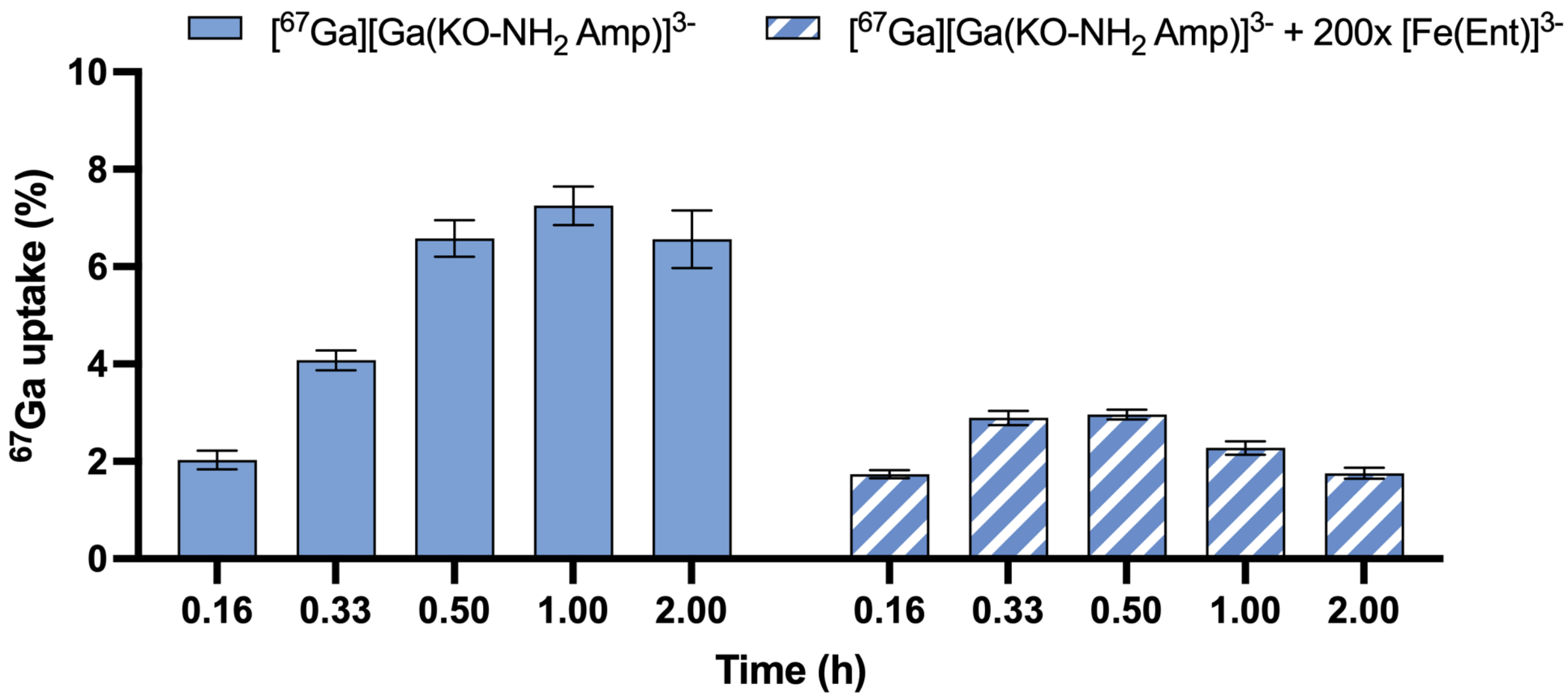

Figure S155. Time-dependent, radiochemical bacterial uptake studies in *E. coli ΔfepA* of **[^{67}Ga][Ga(KO-NH$_2$-Amp)]$^{3-}$** (25 µCi) in iron-depleted, DP treated media pH 7.4.

Table S26. Percentage of internalized of [^{67}Ga][Ga(**KO-NH$_2$-Amp**)]$^{3-}$ in *E. coli ΔfepA* and when challenged with 200x [Fe(Ent)]$^{3-}$ in iron deficient bacterial culture (n=5).

Time (h)	**[^{67}Ga][Ga(KO-NH$_2$-Amp)]$^{3-}$** (%)	**[^{67}Ga][Ga(KO-NH$_2$-Amp)]$^{3-}$** + 200x [Fe(Ent)]$^{3-}$ (%)
0.16	2.031 ± 0.173	1.734 ± 0.083
0.33	4.077 ± 0.181	2.893 ± 0.147
0.50	6.579 ± 0.338	2.961 ± 0.99
1	7.252 ± 0.355	2.277 ± 0.135
2	6.564 ± 0.531	1.757 ± 0.115

3. Radiochemistry

3.1 Production and labeling procedures

3.1.1. $^{67}GaCl_3$

^{67}Ga-citrate was received from Jubilant Radiopharma at an average specific activity of 4 mCi.mL^{-1}. $^{67}GaCl_3$ (70 µL, 0.5 mCi) was added to a solution of ligand (30 µL, 1 mM) in DMSO/H_2O (20/80). The pH of the labeling solution was adjusted to 7 using HEPES (250 µL, 50 mM, pH 7.4). Quantitative radiolabeling was confirmed via radio-HPLC (method D). To the labeling solution was added 330 µL of PBS buffer for injection.

3.1.2. $^{68}GaCl_3$

^{68}Ga radioisotope was obtained from an IGG100 ^{68}Ge/^{68}Ga generator (The Eckert & Ziegler Isotope) and eluted with 0.1 M HCl. $^{68}GaCl_3$ (60 µL, 1.00 mCi) was added to a solution of ligand (20 µL, 1 mM) in DMSO/H_2O (20/80). The pH of the labeling solution was adjusted to 7 using HEPES (60 µL, 1 M, pH 7.0). Quantitative radiolabeling was confirmed via radio-HPLC (method C). To the labeling solution was added 330 µL of PBS buffer for injection.

3.2 Radio-HPLC traces

3.2.1. ^{67}Ga-based complexes

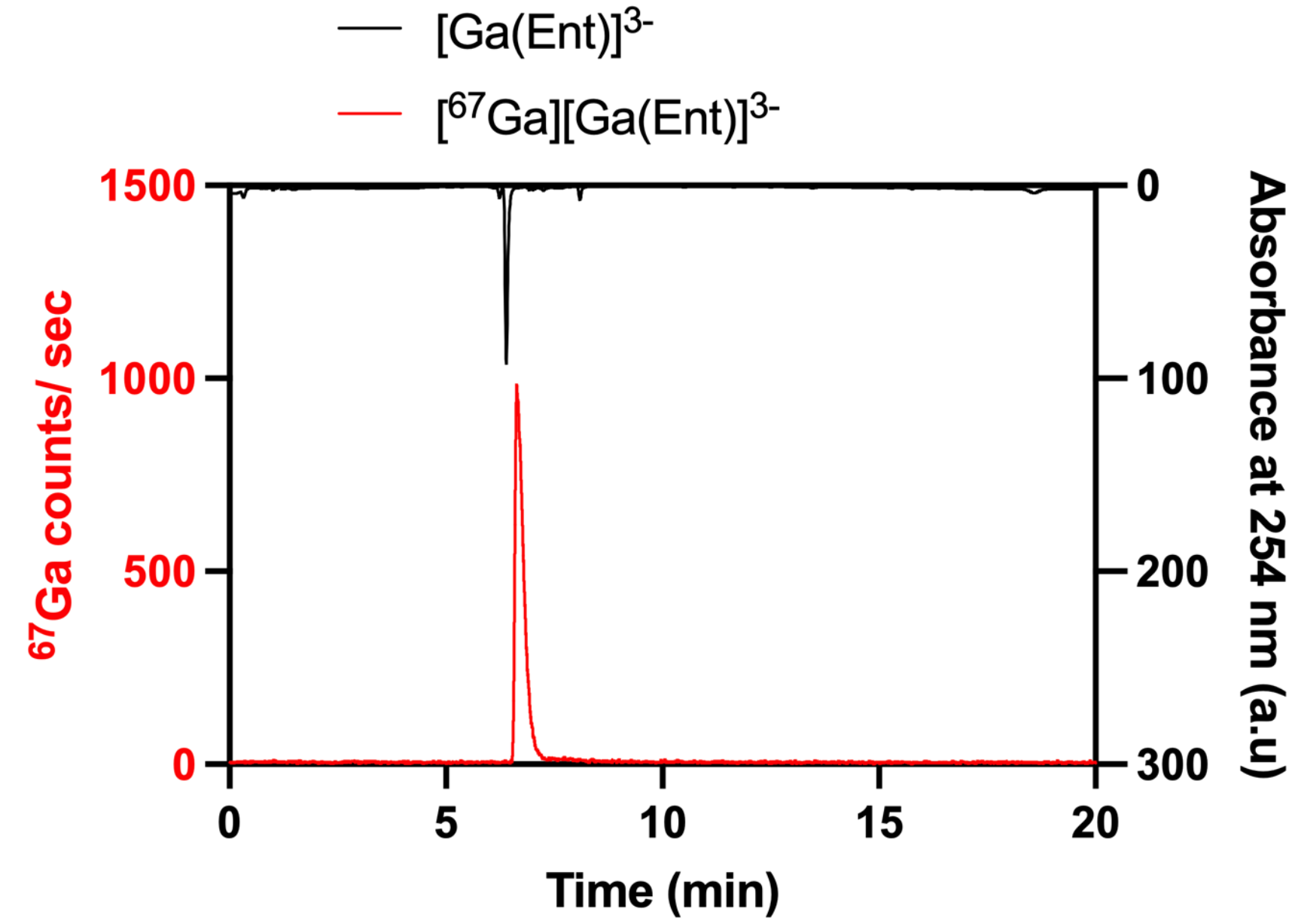

Figure S156. Radio-HPLC of **[^{67}Ga][Ga(Ent)]$^{3-}$**, Retention time = 6.63 min (Method D).

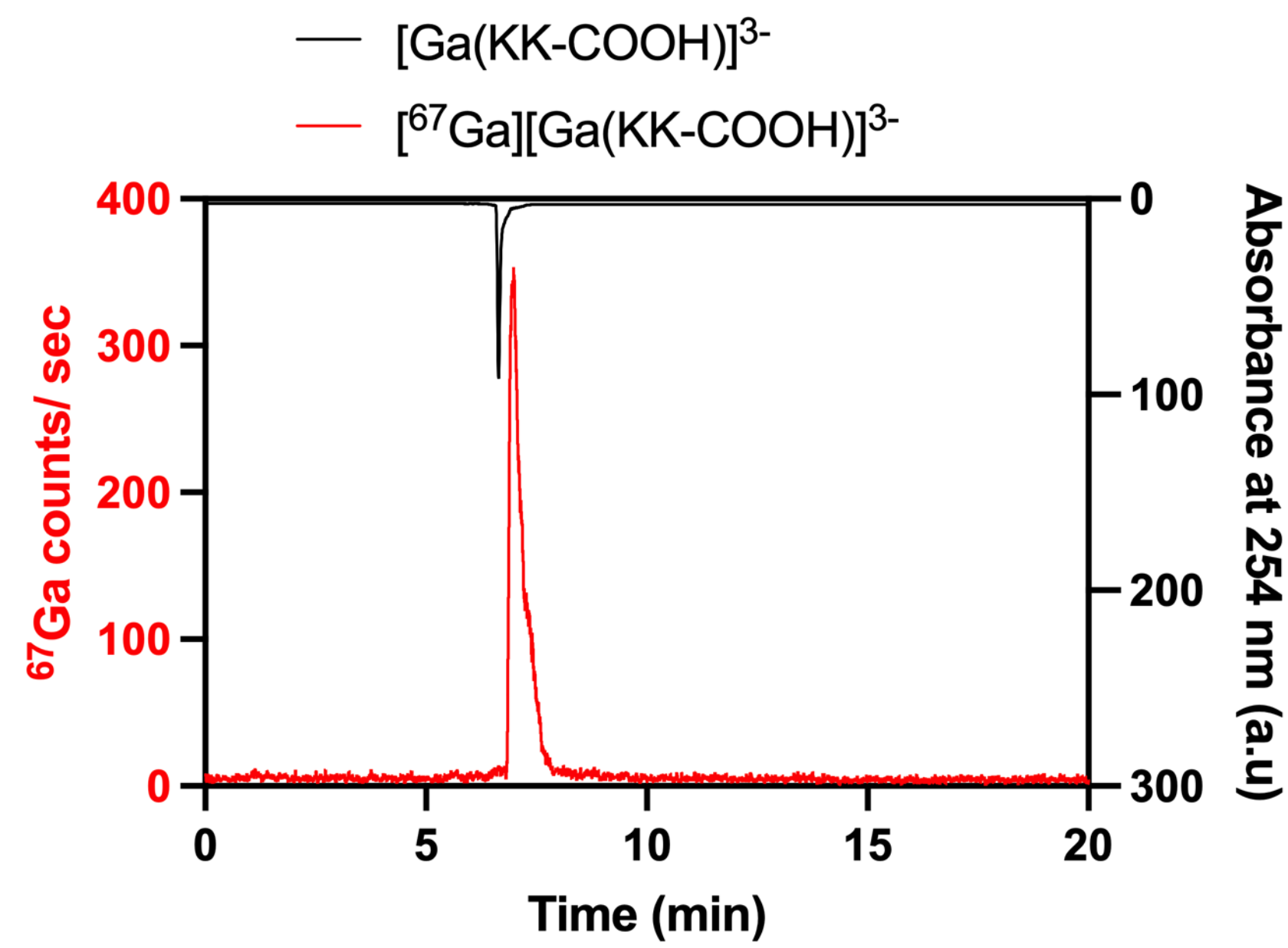

Figure S157. Radio-HPLC of **[^{67}Ga][Ga(KK-COOH)]$^{3-}$**, Retention time = 7.00 min (Method D).

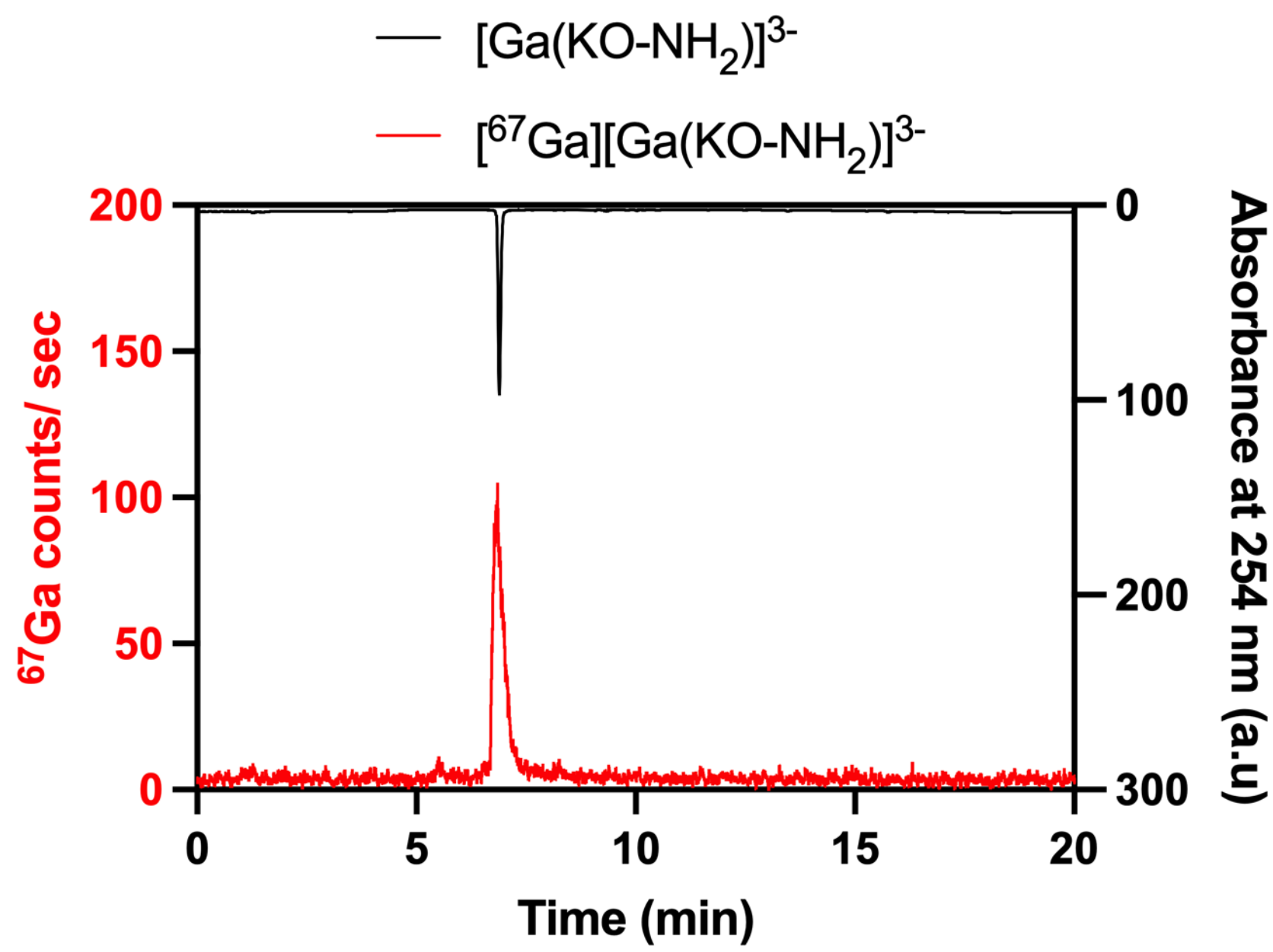

Figure S158. Radio-HPLC of **[**^{67}**Ga][Ga(KO-NH**$_2$**)]**$^{3-}$, Retention time = 6.85 min (Method D).

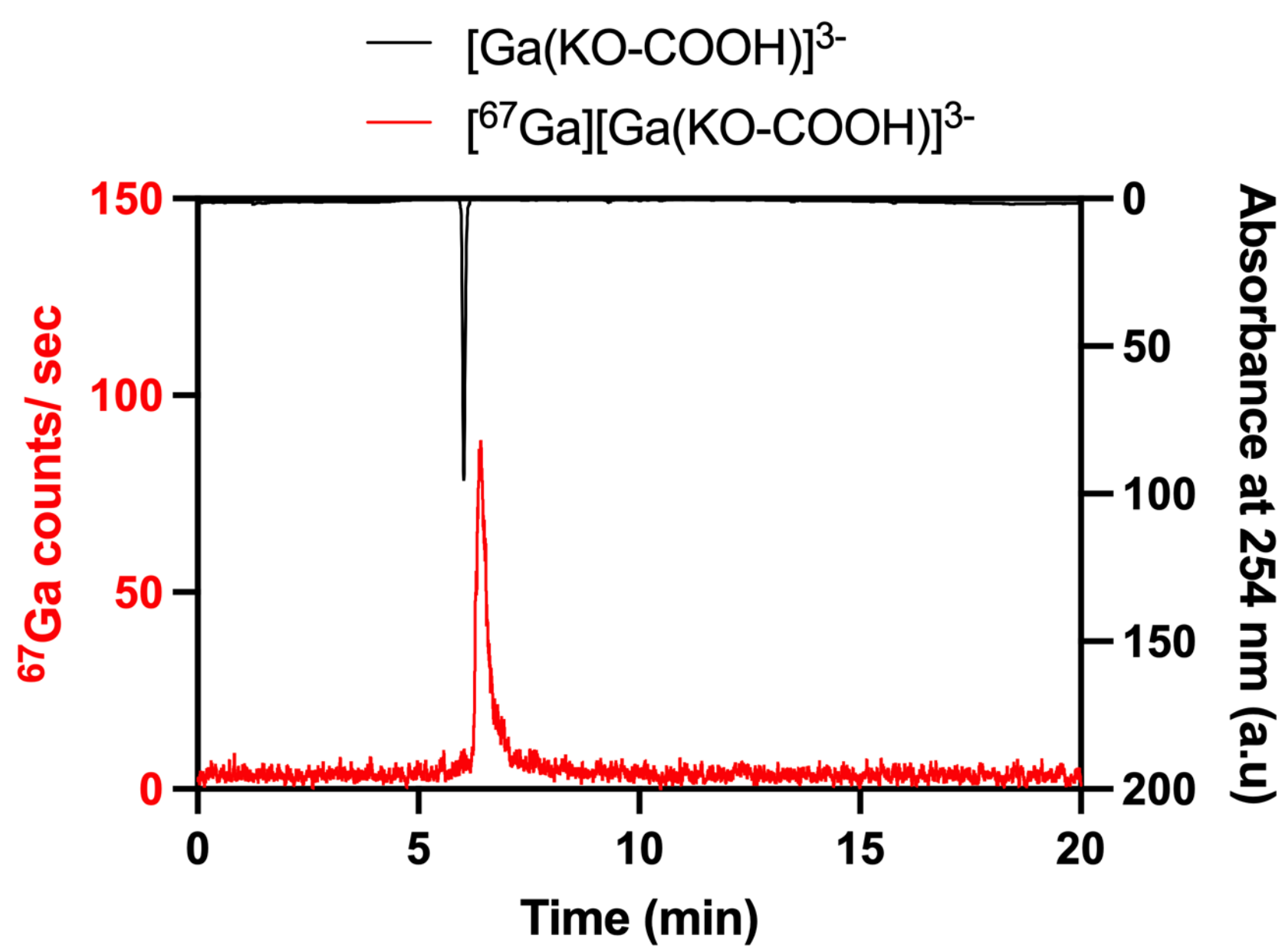

Figure S159. Radio-HPLC of **[**^{67}**Ga][Ga(KO-COOH)]**$^{3-}$, Retention time = 6.42 min (Method D).

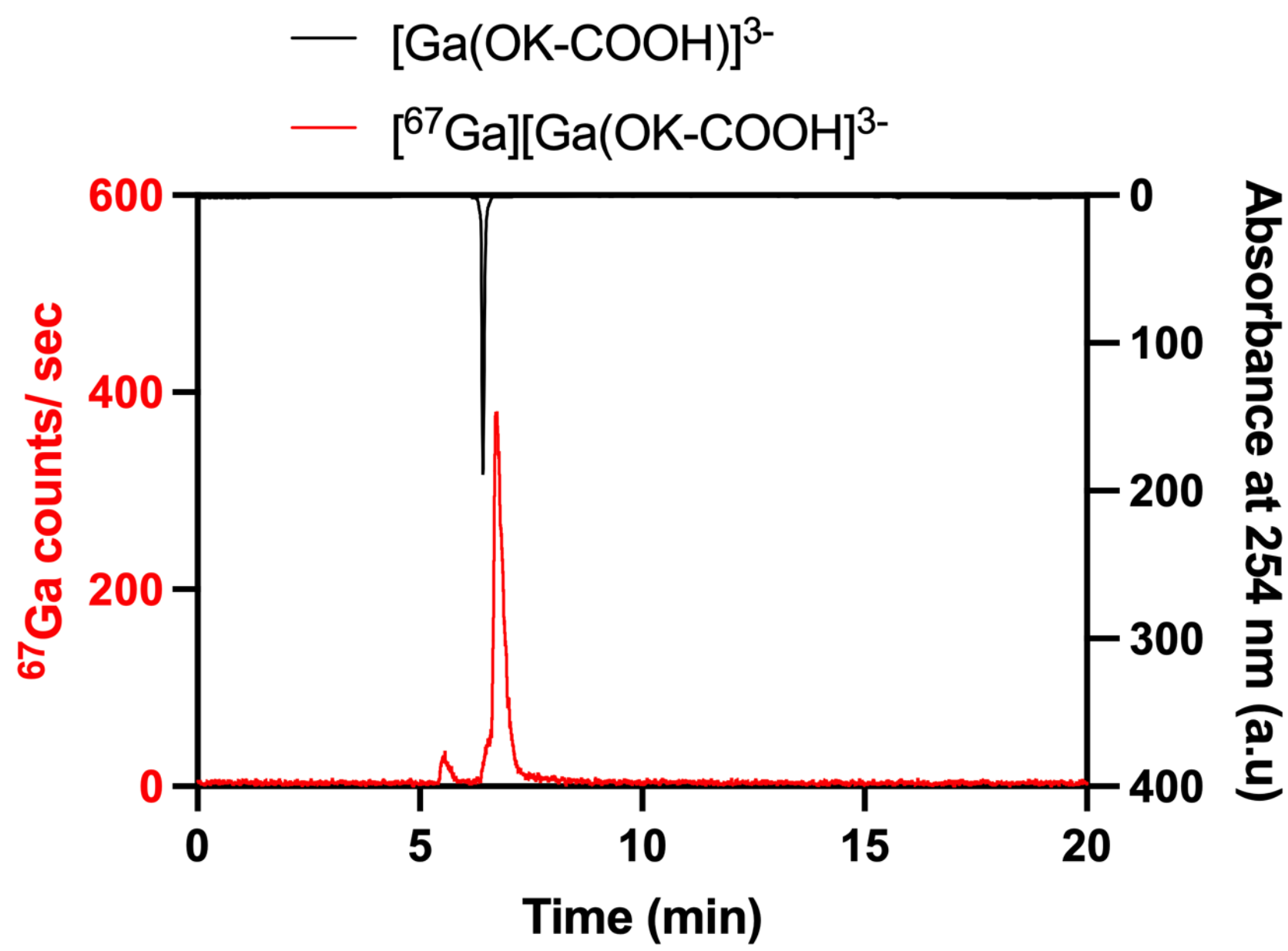

Figure S160. Radio-HPLC of **[^{67}Ga][Ga(OK-COOH)]$^{3-}$**, Retention time = 6.74 min (Method D).

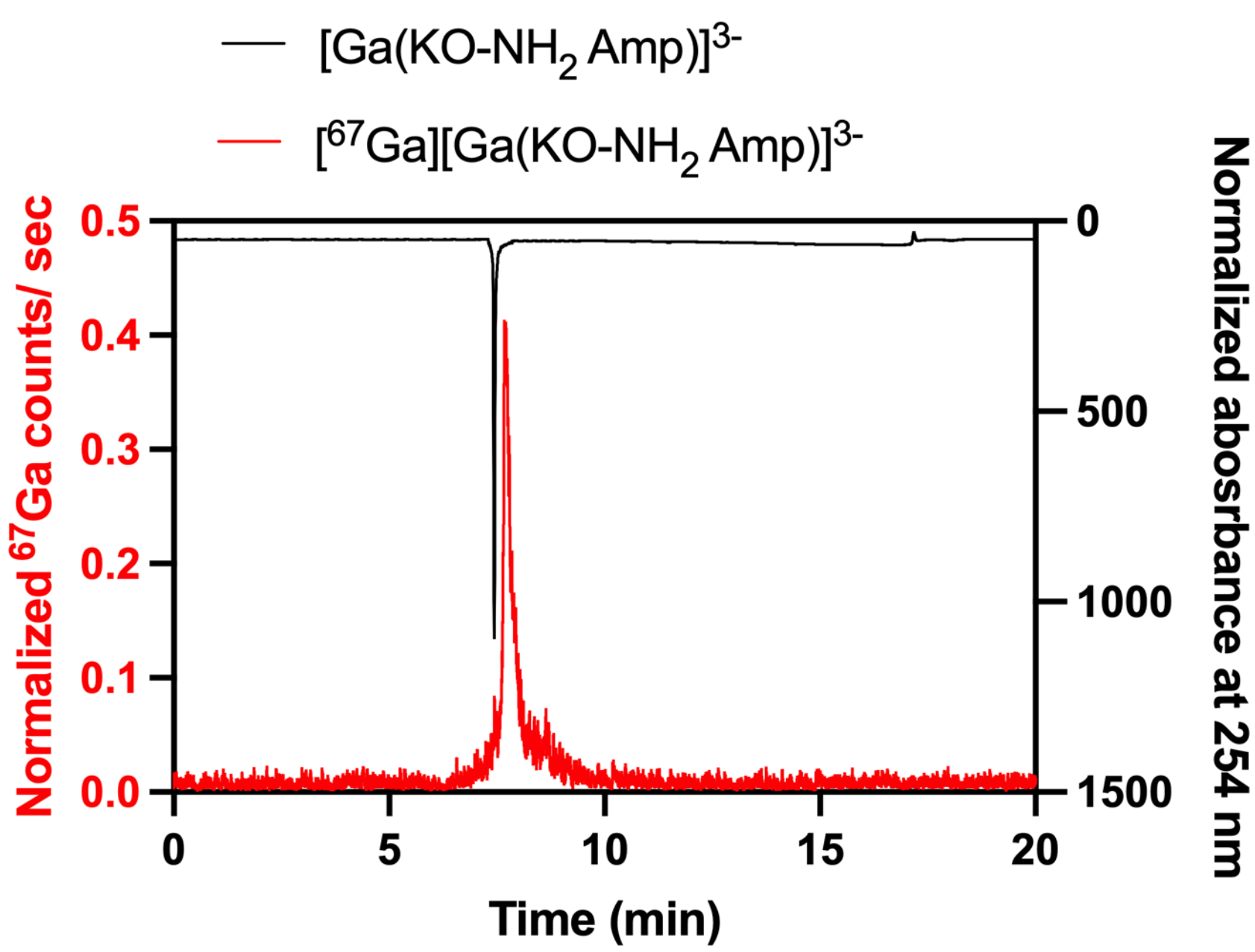

Figure S161. Radio-HPLC of **[^{67}Ga][Ga(KO-NH$_2$-Amp)]$^{3-}$**, Retention time = 7.43 min (Method D).

3.2.2. ^{68}Ga-based complexes

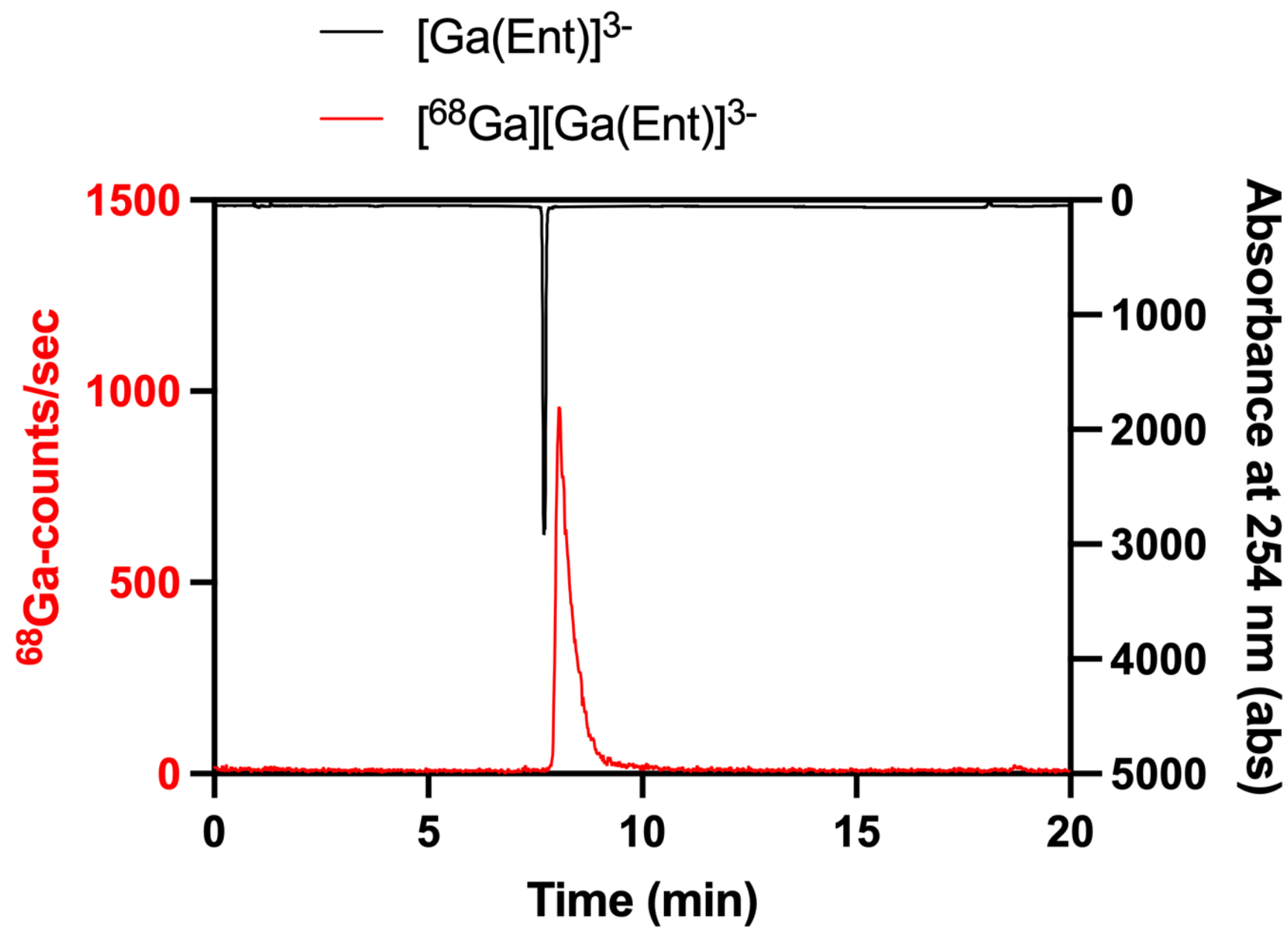

Figure S162. Radio-HPLC of **[^{68}Ga][Ga(Ent)]$^{3-}$**, Retention time = 8.08 min (Method D).

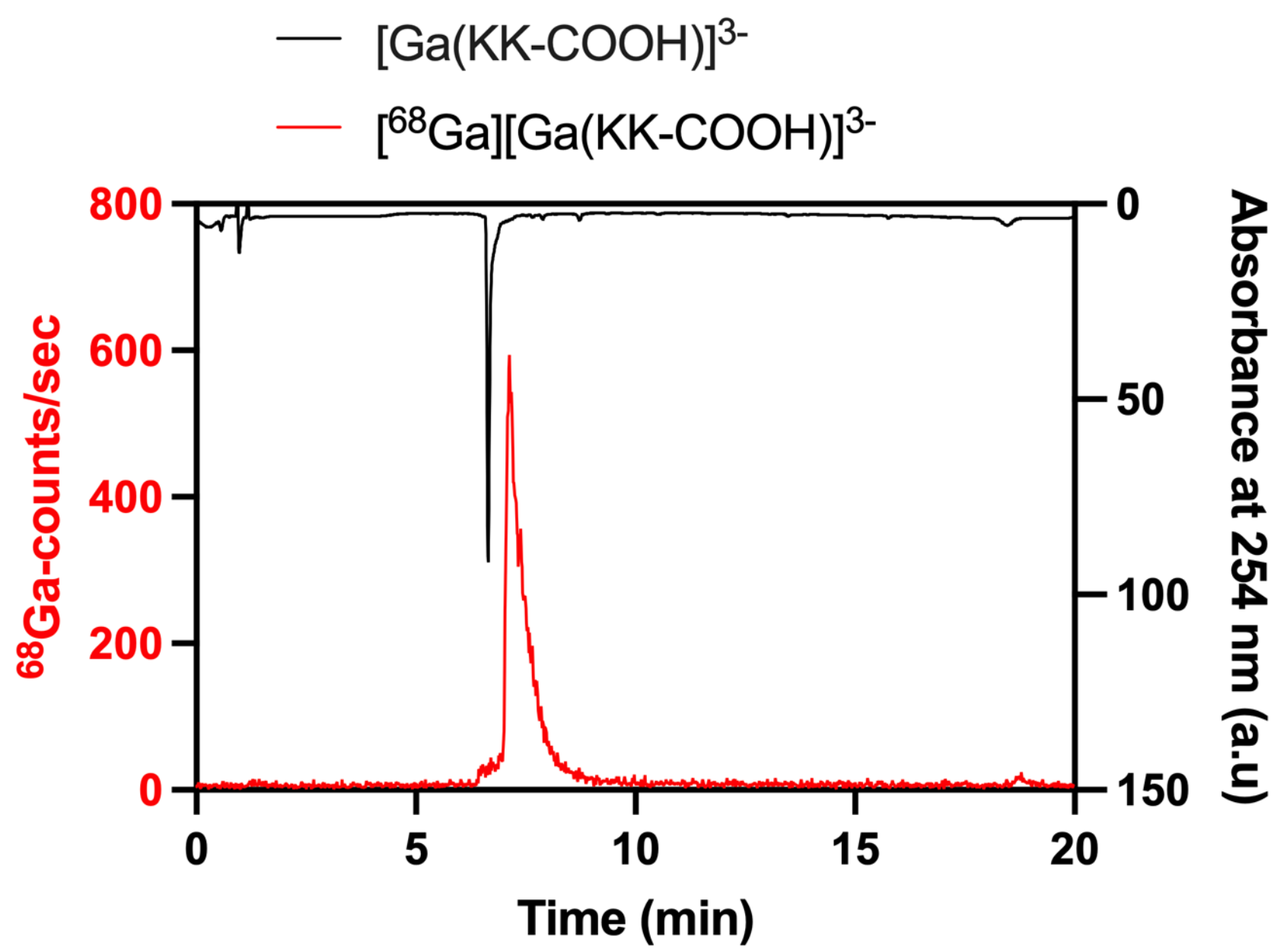

Figure S163. Radio-HPLC of **[^{68}Ga][Ga(KK-COOH)]$^{3-}$**, Retention time = 7.13 min (Method D).

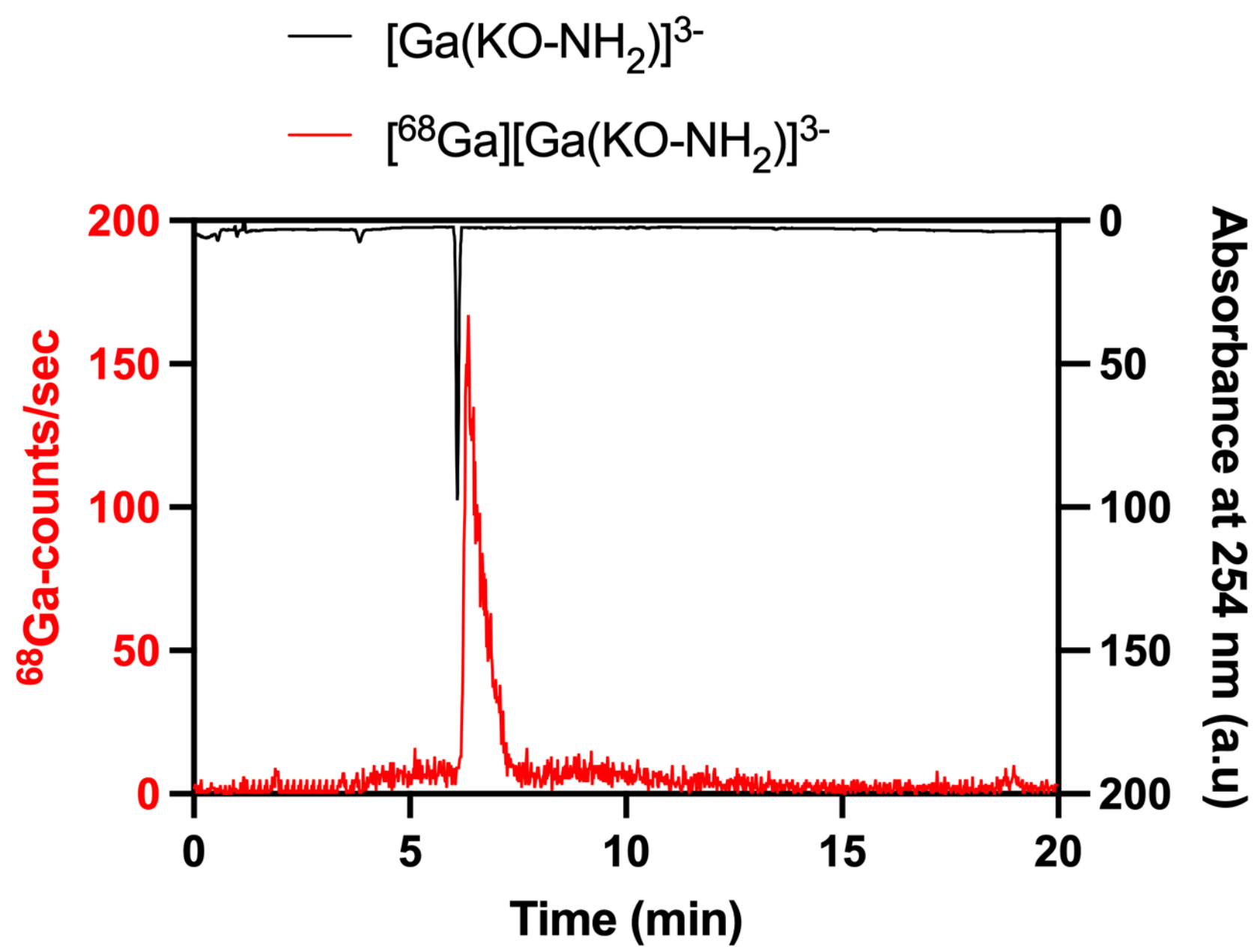

Figure S164. Radio-HPLC of **[^{68}Ga][Ga(KO-NH$_2$)]$^{3-}$**, Retention time = 6.14 min (Method D).

4. Pharmacokinetics in naive and infected mice models

All animal experiments were conducted with the approval of the University of Wisconsin-Madison Institutional Animal Care and Use Committee (IACUC). All studies were conducted in accordance with the relevant guidelines and regulations and approved under protocol number M006739 (PI: Boros) and conducted at UW-Madison School of Medicine and Public Health, at the Small Animal Imaging and Radiotherapy Facility (SAIRF).

4.1 Evaluation of naive mice with $[^{68}Ga][Ga(KK\text{-}COOH)]^{3-}$ and $[^{68}Ga][Ga(KO\text{-}NH_2)]^{3-}$

4.1.1 Biodistribution

50-100 µCi of **$[^{68}Ga][Ga\text{-complexes}]^{3-}$** were intravenously injected via tail vein catheter. Mice (female Balb-C mice, 6-8 weeks old) were sacrificed 1 h p.i., and selected organs were harvested. Radioactivity was counted by using a gamma counter, and the radioactivity associated with each organ was expressed as % ID/g. 100 µL of urine was directly injected to the radio HPLC. Eluate was collected in 30 s increments from 0–20 min. Activity in each tube was quantified using a gamma counter. The counts were used to reconstruct the metabolite trace which was then compared to the HPLC traces of the original 68Ga complexes.

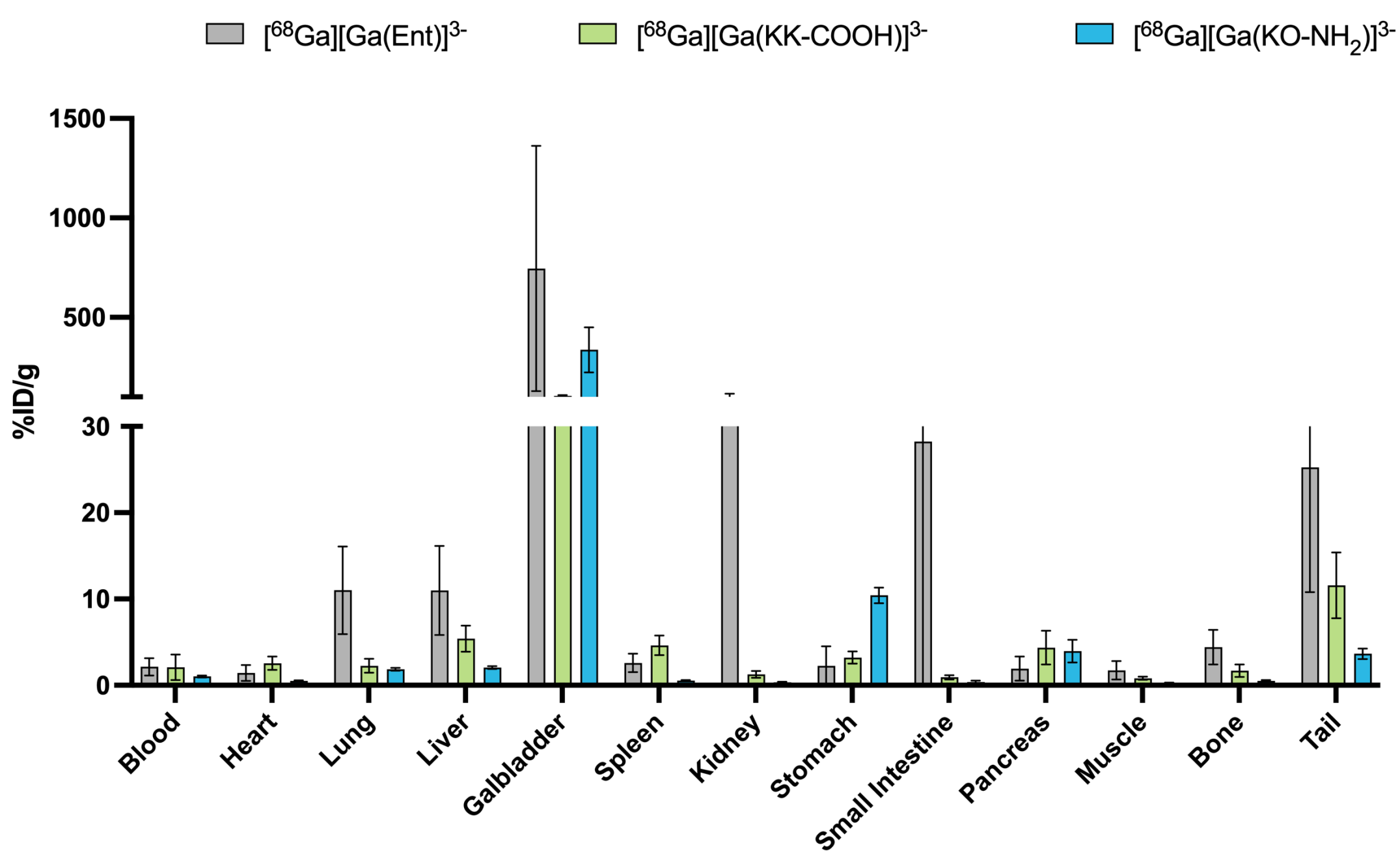

Figure S165. Biodistribution of **$[^{68}Ga][Ga(Ent)]^{3-}$**, **$[^{68}Ga][Ga(KK\text{-}COOH)]^{3-}$**, and **$[^{68}Ga][Ga(KO\text{-}NH_2)]^{3-}$** in naïve mice. Data is expressed as the average percentage of injected dose per gram of organs, and error bars are the standard deviation of the mean.

Table S27. Tabulated values for the biodistribution of $[^{68}Ga][Ga(Ent)]^{3-}$, $[^{68}Ga][Ga($**KK-COOH**$)]^{3-}$, $[^{68}Ga][Ga($**KO-NH$_2$**$)]^{3-}$ at 1 h post injection. Values expressed as %ID/g.

	**$[^{68}Ga][Ga(Ent)]^{3-}$**	**$[^{68}Ga][Ga(KK\text{-}COOH)]^{3-}$**	**$[^{68}Ga][Ga(KO\text{-}NH_2)]^{3-}$**
Blood	2.14±0.99	2.09±1.47	1.02±0.12
Heart	1.43±0.91	2.56±0.77	0.50±0.07
Lung	11.01±5.07	2.26±0.80	1.86±0.17
Liver	10.99±5.15	5.41±1.52	2.05±0.16
Kidney	90.53±26.55	1.27±0.40	0.36±0.06
Spleen	2.59±1.07	4.63±1.13	0.54±0.06
Pancreas	1.93±1.39	4.36±1.95	3.96±1.33
Stomach	2.25±2.27	3.22±0.71	10.42±0.91
Small intestine	28.25±33.91	0.93±0.25	0.37±0.17
Muscle	1.74±1.08	0.82±0.17	0.25±0.06
Bone	4.43±2.01	1.69±0.71	0.51±0.10
Gallbladder	745.71±616.86	104.87±3.78	337.33±113.33
Tail	25.26±14.48	11.59±3.81	3.65±0.61

4.1.2 Metabolite analysis

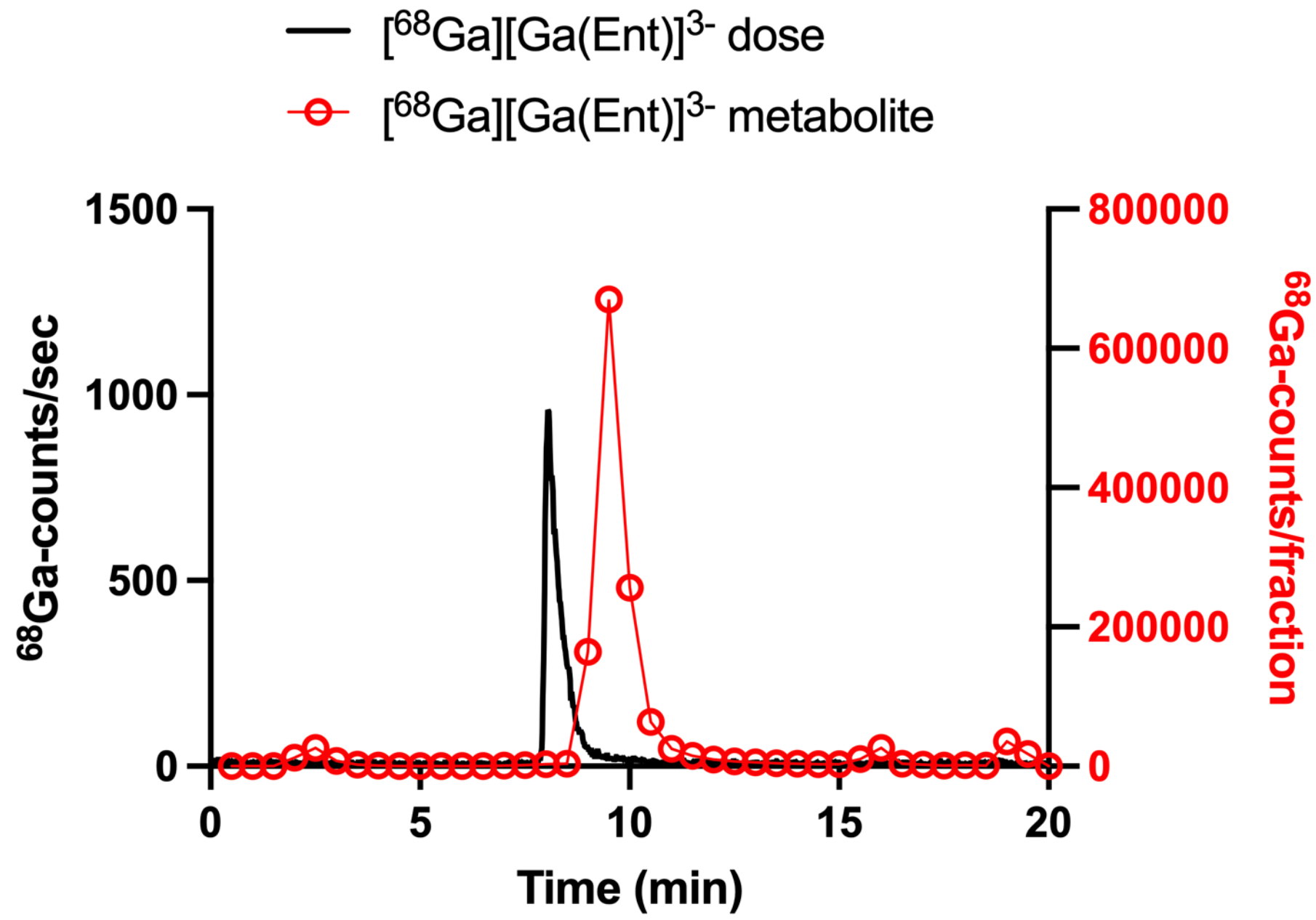

Figure S166. Radio-HPLC chromatogram of urine from dosed mouse, 1 h p.i and the **$[^{68}Ga][Ga(Ent)]^{3-}$** formulation solution.

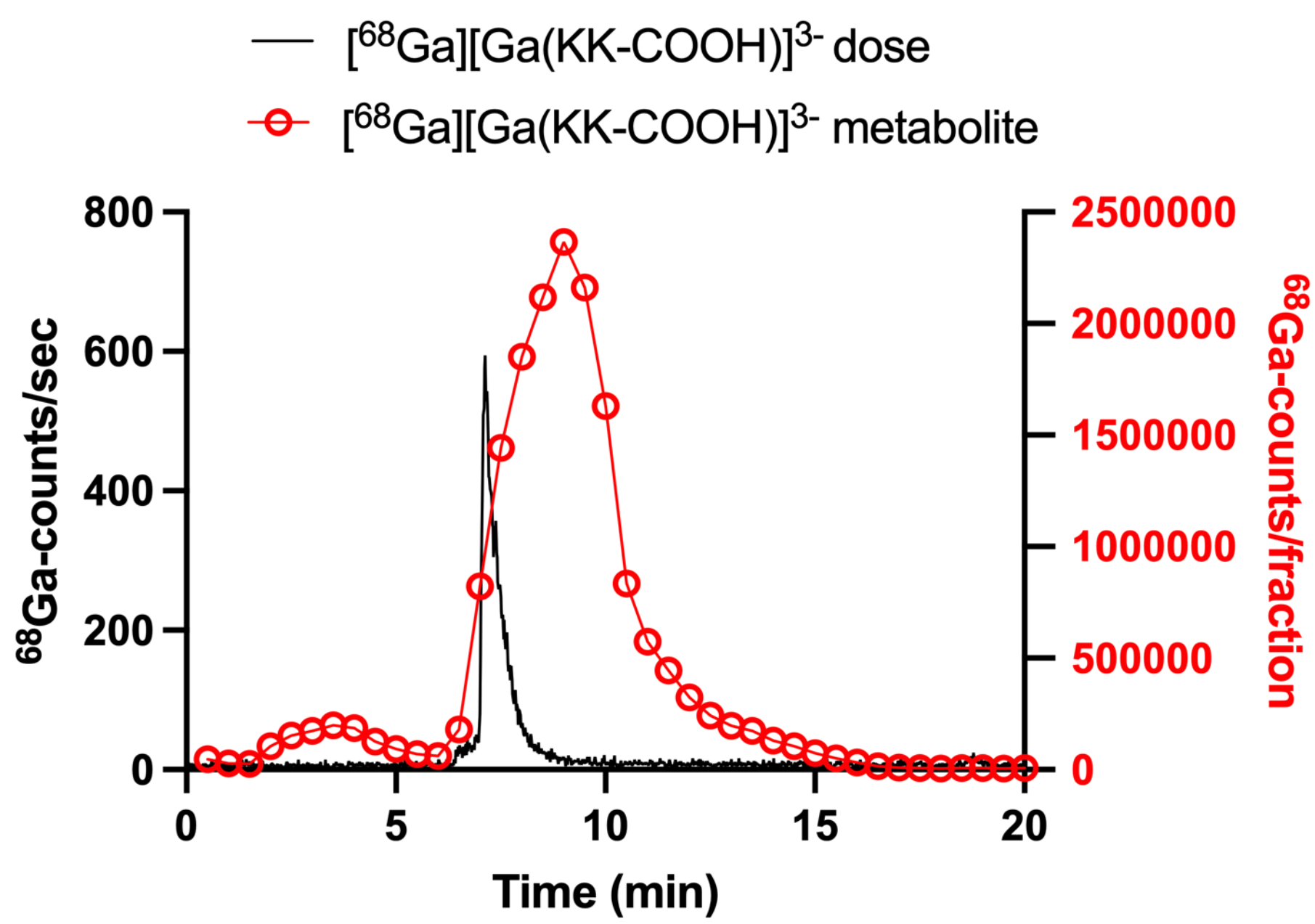

Figure S167. Radio-HPLC chromatogram of urine from dosed mouse, 1 h p.i and the **$[^{68}Ga][Ga(KK\text{-}COOH)]^{3-}$** formulation solution.

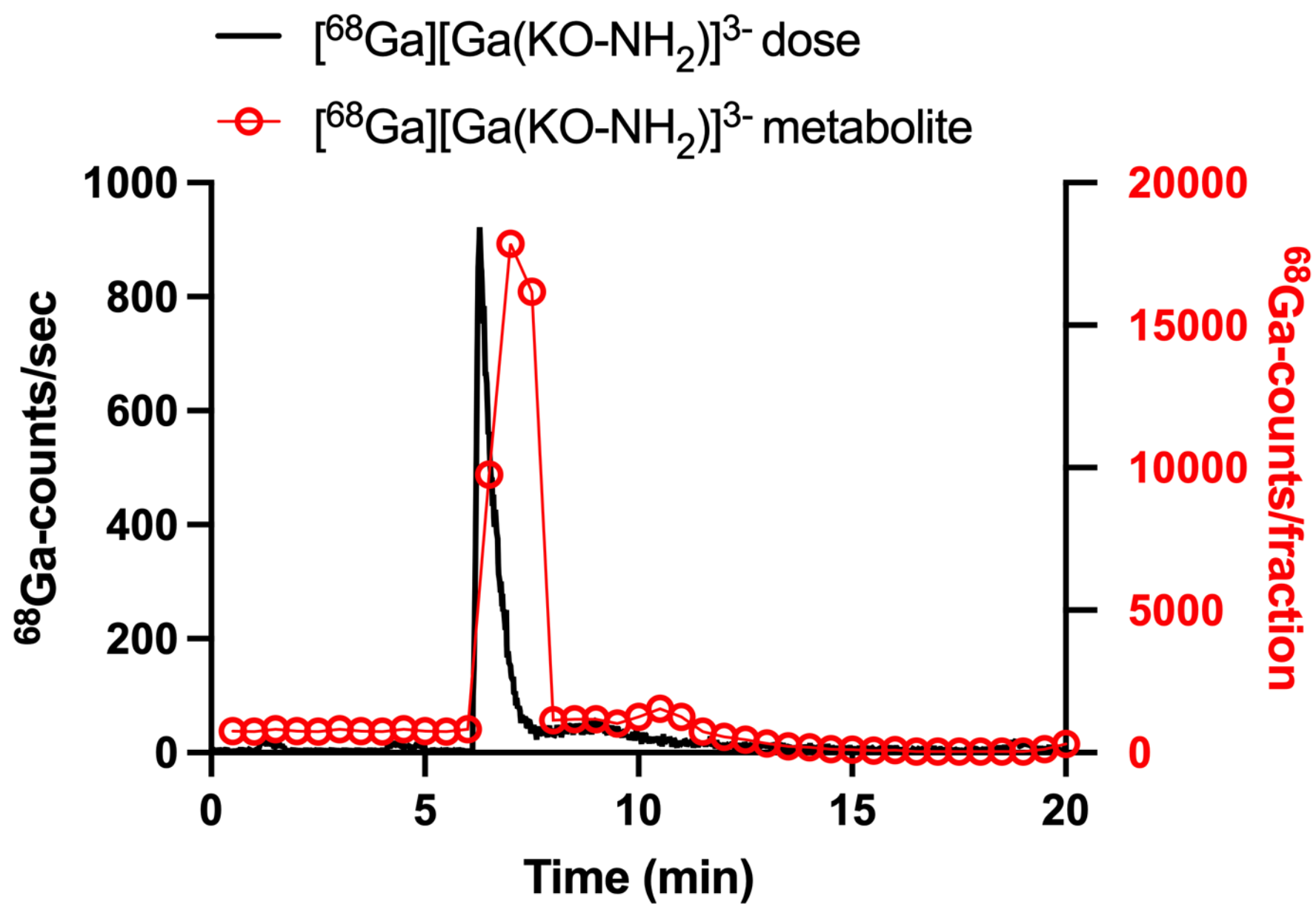

Figure S168. Radio-HPLC chromatogram of urine from dosed mouse, 1 h p.i and the **[^{68}Ga][Ga(KO-NH$_2$)]$^{3-}$** formulation solution.

4.2 Evaluation of E. coli infected mice with [^{68}Ga][Ga(KO-NH$_2$)]$^{3-}$

Procedure

E. coli K12 was inoculated into LB iron-deficient medium (10 mL) and incubated in a shaking incubator at 37°C and 210 rpm overnight. The overnight cultures were then diluted 1:100 in fresh LB iron-deficient medium and incubated at 37°C (210 rpm) until the OD_{600} = 0.3 - 0.4 (2 h) was reached corresponding to a concentration of 1.2 - 1.6 x 10^8 CFU/mL. Six weeks old female balb/c mice were anesthetized with isoflurane and a 60 µL aliquot of bacterial inoculum (1 x 10^8 CFU/mL) was injected in the left triceps of the mouse (infected muscle), followed by administration of LPS (30 µL of LPS at 0.9 mg/mL) in the right triceps to provide a contralateral sterile inflammation control (inflamed muscle). The microbial infection was allowed to develop for 5 h. Upon development of infection/inflammation, 100 µL (containing 180-200 µCi of radiotracer) of the PBS formulation of **[^{68}Ga][Ga(KO-NH$_2$)]$^{3-}$** was administered into mice (n = 4 per cohort) via tail vein injection.

4.2.1 PET-CT imaging

To determine the pharmacokinetics of the imaging agent, PET-CT scans were collected 1.5 h post injection of the radiotracer. The two mice that received the highest doses (190 µCi) were used for this purpose. Scans were acquired in a Siemens Inveon Hybrid MicroPET/CT Scanner (Siemens Medical Solutions USA, Inc., Knoxville, TN). Mice were initially anesthetized isoflurane gas and maintained during scans at 2% isoflurane in oxygen. CT scans were acquired prior to PET scans for anatomical coregistration as well as attenuation correction. CT scan parameters were as follows: 220 rotation degrees, 120 rotation steps, binning factor of 4, exposure time of 250 ms, x-ray energy of 80 kVp, 1 mA current, and 105 µm resolution. PET scans were acquired with 40 million coincidence events per mouse, an energy window of 350-650 keV, and a timing window of 3.432 ns. PET-CT images were normalized to units of percentage of injected dose per cubic centimeter and presented as maximum intensity projected scan (MIPS) images.

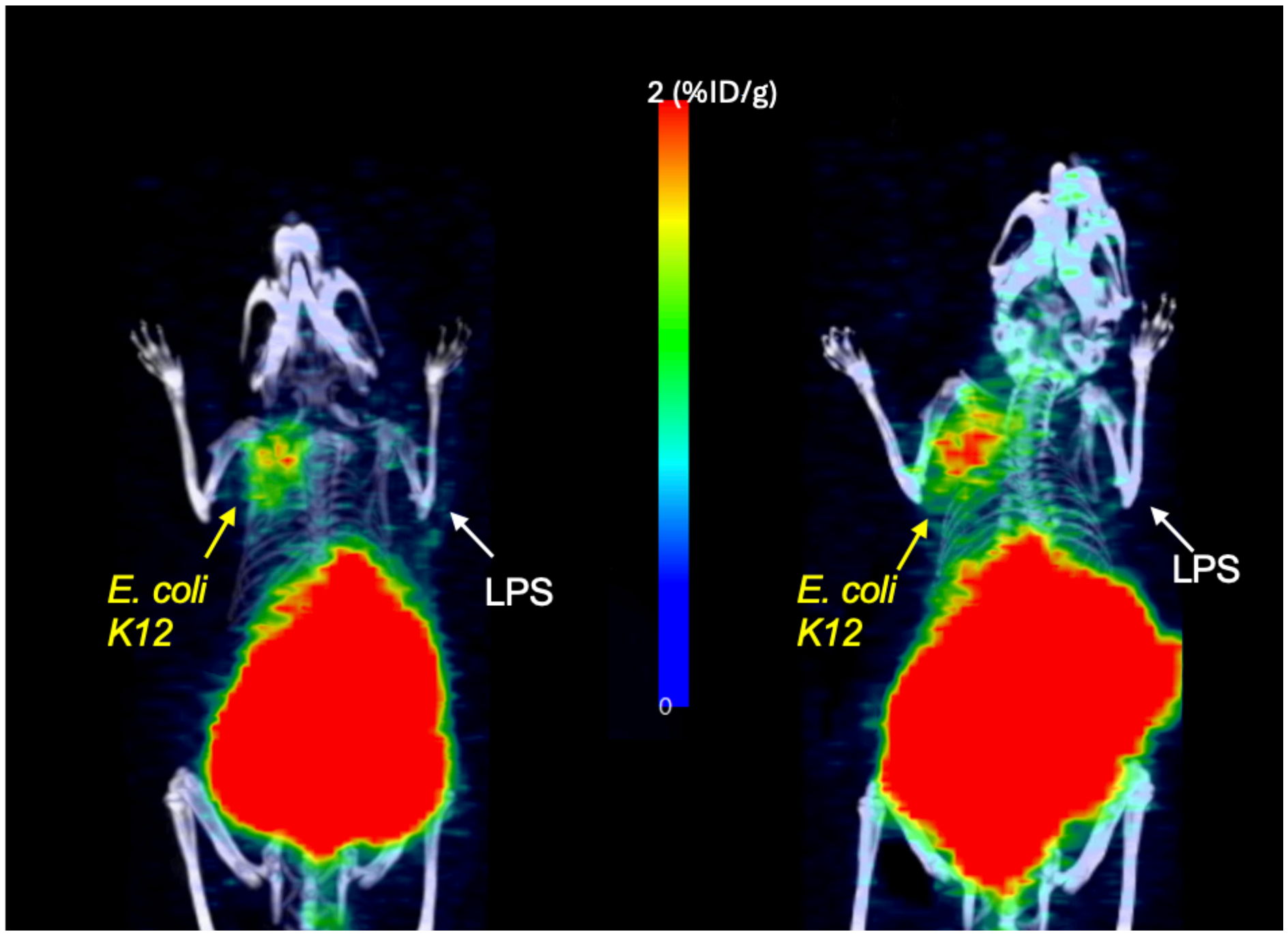

Figure S169. Full PET/CT imaging of [^{68}Ga][Ga(**KO**-**NH$_2$**)]$^{3-}$ in infected mice.

4.2.2 Biodistribution

Following PET-CT imaging, mice (n= 4 per cohort) were euthanized 2 h post-injection of radiotracer. Urine and blood were collected, organs were harvested, and activity assessed via a gamma counting (Hidex Automatic Gamma Counter). Recorded measurement of counts per minute (cpm) were decay-corrected to the time of injection of the ^{68}Ga-radiotracer, converted to milicurie (mCi) units by using the efficiency of the Gamma Counter for Ga-68, and the amount of activity present in each organ was expressed as percentage of injected dose per gram of organ weight (% ID/g).

Table S28. Tabulated values for the biodistribution in *E. coli K12* infected mice of $[^{68}Ga][Ga(\mathbf{KO}\text{-}\mathbf{NH_2})]^{3-}$ at 2 h post injection. Values expressed as %ID/g.

	$[^{68}Ga][Ga(KO\text{-}NH_2)]^{3-}$
Blood	1.67 ± 1.48
Heart	1.10 ± 0.52
Lung	2.68 ± 1.35
Liver	8.91 ± 4.09
Gallbladder	22.65 ± 3.60
Spleen	1.27 ± 0.67
Pancreas	1.32 ± 0.83
Kidney	1127.20 ± 246.80
Stomach	1.32 ± 0.96
Small Intestine	2.62 ± 1.70
Healthy Muscle	0.41 ± 0.19
Infected Muscle	1.28 ± 0.12
LPS Inflamed Muscle	0.42 ± 0.082
Bone	0.95 ± 0.31
Tail	1.95 ± 0.79

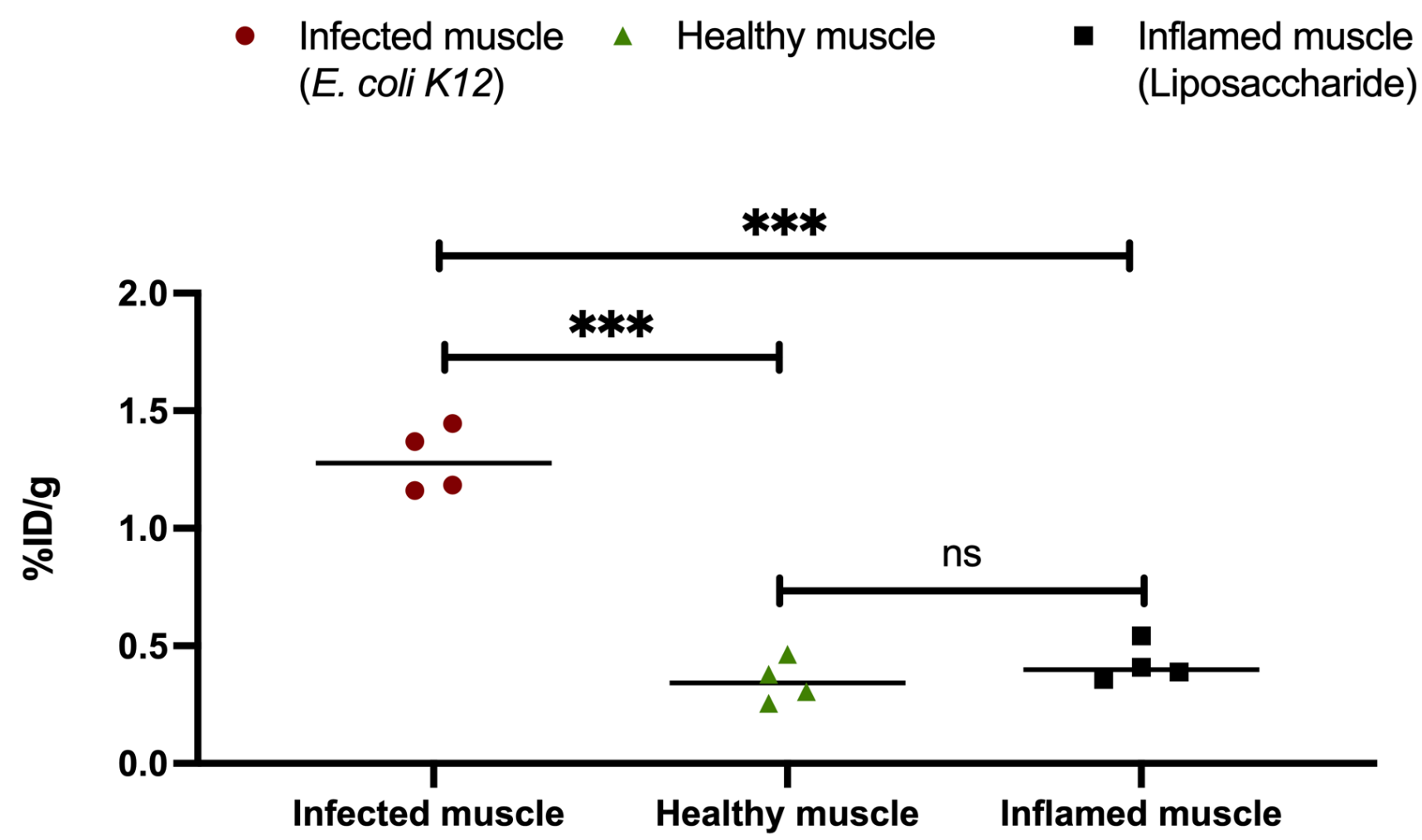

Figure S170. Quantification of [^{68}Ga][Ga(**KO-NH$_2$**)]$^{3-}$ uptake in infected triceps, inflamed triceps (LPS), and non-injected triceps (healthy) for n = 4. Paired t-test was performed revealing: ***p value = 0.0009 (healthy muscle vs infected muscle), *p = 0.02 (healthy muscle vs inflamed muscle), and ***p value = 0.0009 (infected muscle vs inflamed muscle).

4.2.3 Tissue dissociation and plating

Muscle tissues from the infected right triceps, inflamed left triceps, and healthy quadriceps were collected immediately after biodistribution studies to confirm the presence of viable *E. coli* K12 cells at the infection site and their absence in the healthy and inflamed tissues. The harvested samples were placed in gentleMACS™ C tubes, combined with 5 mL of sterile 1X DPBS buffer, and homogenized using a gentleMACS™ Dissociator for 40 minutes. The resulting homogenates were plated on LB-agar plates and incubated overnight at 37 °C.

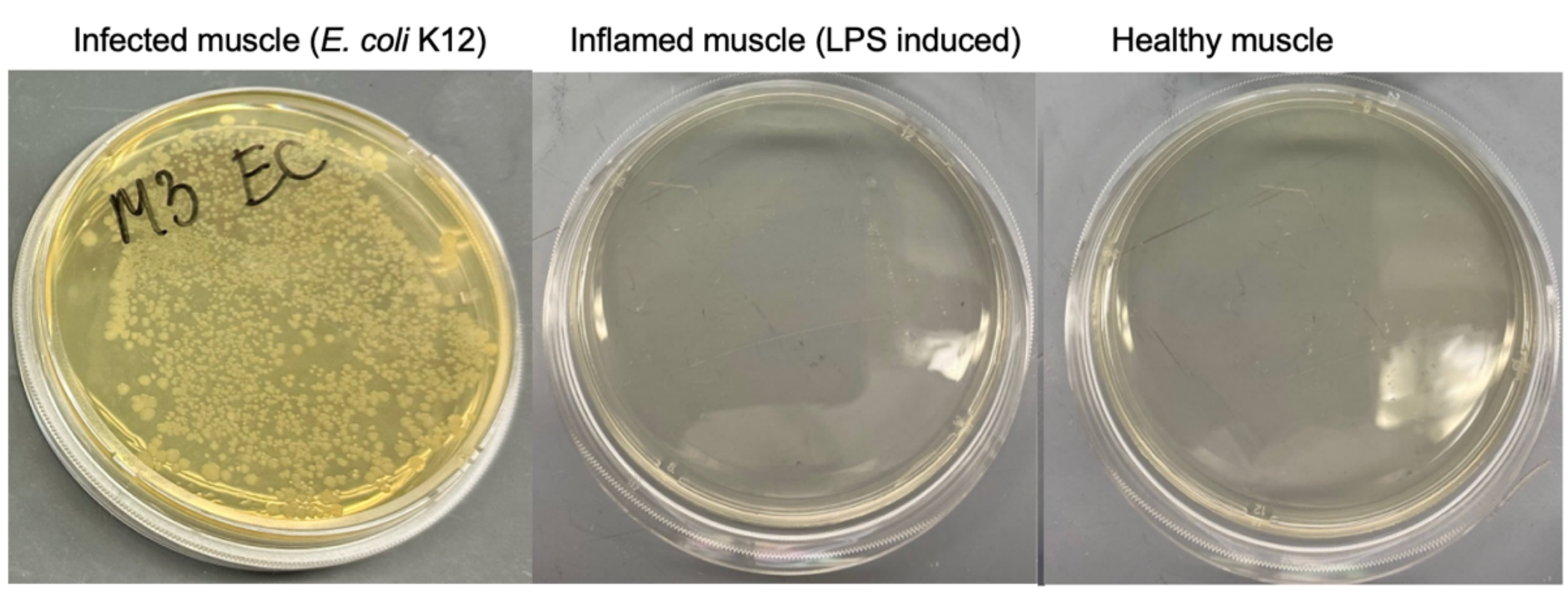

Figure S171. Representative of growth bacterial colonies on LB agar plates inoculated with the homogenized tissues derived from *E. coli* K12 infected muscle, inflamed tissue (LPS induced), and healthy muscle harvested from mice used for PET/CT imaging and *ex vivo* biodistribution with [^{68}Ga][Ga(**KO-NH$_2$**)]$^{3-}$. Viable bacterial colonies are only observed in the plates inoculated with the *E. coli* K12 infected sample tissue.

5. Biotin-Streptavidin Pull Down Assay

5.1.1 Biotin-Streptavidin enrichment

Stock solutions of **KO-NH$_2$-azir** and **alkyne-azir-biotin** were prepared using their extinction coefficient at 320 nm in DMSO and 240 nm in MeOH, respectively: $\varepsilon_{KO\text{-}NH2\text{-}azir}$= 7897 $M^{-1}cm^{-1}$ (320 nm); $\varepsilon_{alkyne\text{-}azir\text{-}biotin}$ = 923.9 $M^{-1}cm^{-1}$ (240 nm).

Bacterial cultures of *E. coli K-12* and *P. aeruginosa PAO1* were grown from cryostocks in 10 mL iron-deficient LB (containing 200 µM DP) overnight. Then, bacterial cultures were inoculated in 300 mL iron-deficient LB at an OD_{600nm} of 0.01 and grown until OD_{600nm} of 0.6. The bacteria were spun down at 4,000 g, 4 °C for 20 minutes and washed once with 300 mL PBS before being resuspended in 6 mL PBS. The suspension was distributed into a 12-well plate (1 mL per well, for 3 wells per condition). The probe was added to a concentration of 125 µM to the bacterial solution. Bacteria were incubated with the probe for 4 hours at 37 °C, 145 rpm, protected from light. The plate was irradiated at 365 nm for 15 minutes on ice. Samples were gathered, pelleted and washed three times with 2 mL PBS. Pellets were resuspended in lysis buffer (50 mM Tris, pH 7.6, 250 mM NaCl, 1% w/v Triton X-100, 1% w/v OG, 10% w/v glycerol; 10 mL per 1 g of pellet) containing Roche cOmplete™, Mini, EDTA-free Protease Inhibitor Cocktail (Sigma-Aldrich, 1183617000). Cells were lysed using a fine-tip sonicator with cycles of alternating 1-second pulses on and 1-second pulses off for a total of 3 minutes on and 3 minutes off. Lysates were incubated on a rotary shaker for 20 hours at 4 °C. Subsequently, samples were centrifuged at 30,000 g for 30 min at 4 °C to separate the supernatant (SN1) from the pellet. Pellets were resuspended in 800 µL of 5% Triton-X-100, 5% OG lysis buffer (50 mM Tris, pH 7.6, 250 mM NaCl, 5% w/v Triton X-100, 5% w/v OG, 10% w/v glycerol) containing Roche cOmplete™, Mini, EDTA-free Protease Inhibitor Cocktail (Sigma-Aldrich, 1183617000), sonicated for 1 min, and incubated on a rotary shaker for 5 hours at 4 °C. Samples were centrifuged at 14,100 g for 10 min at 20 °C to separate the supernatant (SN2) and debris. Supernatants SN1 and SN2 were combined and protein concentration was determined by BCA assay using Pierce™ BCA protein assay kit (#23227). 200 µL of Pierce™ high-capacity streptavidin agarose slurry (#20353) was equilibrated with lysis buffer (3 × 500 µL) and incubated with combined SN1 and SN2 for each sample. Total protein content was normalized to 1 mg across all samples. Incubation on streptavidin was done at 4 °C for 16 h on a rotary shaker. The resin was washed three times with 1 mL 0.2% SDS in PBS 1X, with a 10 min incubation on a rotary shaker for each wash. Elution of enriched proteins was achieved by adding 100 µL of elution buffer (25 mM biotin, 50 mM Tris, 5% DMSO, pH 8) and heating at 95 °C for 5 minutes. 25 µL of each eluate was set aside for visualization on SDS-PAGE, the remaining volume was submitted as liquid for MS/MS analysis.

5.1.2 SDS-PAGE

Protein samples were mixed with loading buffer (5% β-mercaptoethanol in 5X loading buffer containing 5% SDS, 50% glycerol, 0.1% bromophenol blue, 250 mM Tris-HCl pH 6.8) at a 1:5 sample-to-buffer ratio and heated to 95 °C for 5 minutes. The samples were then subjected to electrophoresis using 4–20% Mini-PROTEAN® TGX Stain-Free™ Protein Gels (#4561094) at 130 volts in 1 hour in 1X TGS buffer (Tris/Glycine/SDS, #1610732).

PageRuler™ Prestained Protein Ladder (180-10 kDa, #26616) was used as molecular weight marker. Following electrophoresis, the gels were washed twice with DI water for 5 minutes, fixed in a solution of 30% ethanol and 10% acetic acid in water for 15 min twice, and stained with Pierce™ Silver Stain kit (#24612) according to manufacturer's instructions. Gels were visualized using a iBright™ FL1500 Imaging System (Invitrogen, A4415) in visible protein gels mode, using a white contrast tray.

5.1.3 MS/MS analysis

Enzymatic "In Liquid" Digestion

IP eluates (~90 µL) were diluted with 110 µL of MilliQ water, 110 µL of TCA and 800 µL of acetone (10% TCA and 70% acetone v/v final concentration) to facilitate protein precipitation while incubating on ice for 30minutes. The precipitated proteins were collected by centrifugation for 10 minutes at room temperature at 16,000 x g then washed twice with cold acetone while supernatants were discarded, and centrifugation steps were kept the same as before. Subsequently, protein pellets were air-dried briefly and re-solubilized in 15 µL of 8 M Urea in 50 mM NH_4HCO_3 (pH 8.5) overnight at 4ºC. Reduction followed with 2.5 µl addition of 25 mM DTT plus 42.5µl of 25mM NH_4HCO_3 (pH 8.5) and incubation for 15 minutes at 56°C. Samples were cooled on ice to room temperature and 3 µL of 55 mM CAA (chloroacetamide) was added for the alkylation step performed in darkness at room temperature for 15 minutes. Reaction was quenched with 8 µL addition of 25 mM DTT. Finally, 4 µL of Trypsin/LysC solution [100 ng/µl 1:1 Trypsin (Promega):LysC (FujiFilm) mix in 25 mM NH_4HCO_3] and 25 µL of 25 mM NH_4HCO_3 (pH 8.5) was added to 100 µL final volume. Samples were digested overnight at 37°C in a circulating water bath. Reaction was terminated by acidification with 12 µL 2.5% TFA (Trifluoroacetic Acid) to 0.3% final.

NanoLC-MS/MS

Digests were desalted using Pierce™ C18 SPE pipette tips (100µl volume) per manufacturer protocol and eluted in 20µl of 70/30/0.1% ACN/H_2O/TFA. Dried to completion in the speed-vac and finally reconstituted in 15µl of 0.1% formic acid containing 2% acetontitrile. Peptides were analyzed by nanoLC-MS/MS using the Agilent 1100 nanoflow system (Agilent) connected to hybrid linear ion trap-orbitrap mass spectrometer (LTQ-Orbitrap Elite™, Thermo Fisher Scientific) equipped with an EASY-Spray™ electrospray source (held at constant 45°C). Chromatography of peptides prior to mass spectral analysis was accomplished using capillary emitter column (PepMap® C18, 3µM, 100Å, 150x0.075mm, Thermo Fisher Scientific) onto which 3µl of extracted peptides was automatically loaded. NanoHPLC system delivered solvents A: 99.9% (v/v) H_2O, 0.1% (v/v) formic acid, and B: 99.9% (v/v) acetonitrile, 0.1% (v/v) formic acid at 0.50 µL/min to load the peptides (over a 30 minute period) and 0.3µl/min to elute peptides directly into the nano-electrospray with gradual gradient from 0% (v/v) B to 30% (v/v) B over 80 minutes and concluded with 5 minute fast gradient from 30% (v/v) B to 50% (v/v) B at which time a 5 minute flash-out from 50-95% (v/v) B took place. As peptides eluted from the HPLC-column/electrospray source survey MS scans were acquired in the Orbitrap with a resolution of 120,000 followed by CID-type MS/MS fragmentation of 30 most intense peptides detected in the MS1 scan from 350 to 1800 m/z; redundancy was limited by dynamic exclusion.

Data analysis

Resulting mgf files were used to search against *Escherichia coli* (UP000000625; 4,521 entries) or *Pseudomonas aeruginosa* (UP000002438; 5,681 entries) Uniprot reference proteome database along with a list of common lab contaminants (172 total entries) using in-house Mascot search engine 2.7.0 [Matrix Science] with variable Methionine oxidation, Asparagine and Glutamine deamidation plus user defined **azir** (435.1940 Da) and **KO-NH_2-azir** (1256.53 Da) as additional modifications on Glutamate, Aspartate or Tyrosine. Peptide mass tolerance was set at 10ppm and fragment mass at 0.6 Da. Protein annotations, significance of identification and spectral based quantification was done with Scaffold software (version 5.3.3, Proteome Software Inc., Portland, OR). Peptide identifications were accepted if they could be established at greater than 11.0% probability to achieve an FDR less than 1.0% by the Peptide Prophet algorithm (Keller, A et al., *Anal. Chem.* **2002***,* 74(20), 5383) with Scaffold delta-mass correction. Protein

identifications were accepted if they could be established at greater than 99.0% probability to achieve an FDR less than 1.0% and contained at least 2 identified peptides. Protein probabilities were assigned by the Protein Prophet algorithm (Nesvizhskii, Al et al., *Anal. Chem*. **2003**, 75(17), 4646). Proteins that contained similar peptides and could not be differentiated based on MS/MS analysis alone were grouped to satisfy the principles of parsimony. Proteins sharing significant peptide evidence were grouped into clusters.

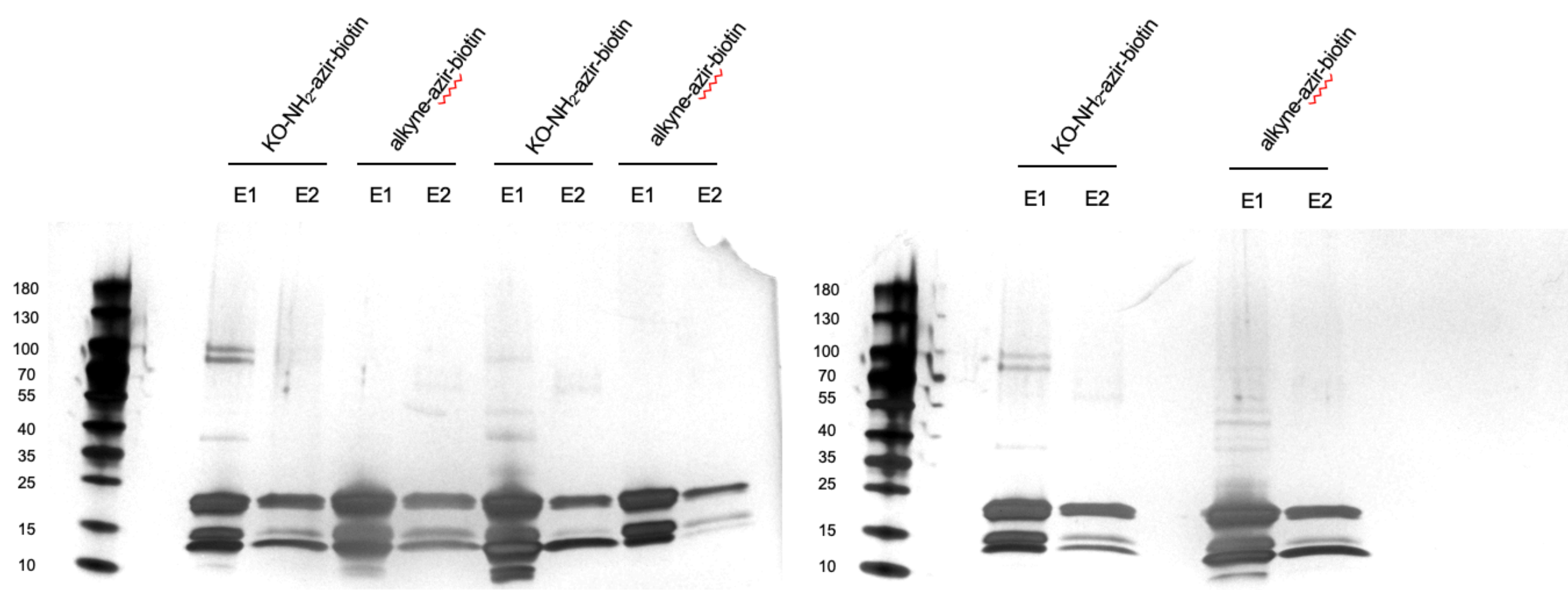

Figure S172. Enrichment of biotinylated proteins from *E. coli K-12* after live-cell incubation with **KO-NH₂-azir-biotin** and **alkyne-azir-biotin** control visualized by SDS-PAGE with silver staining (independent replicates).

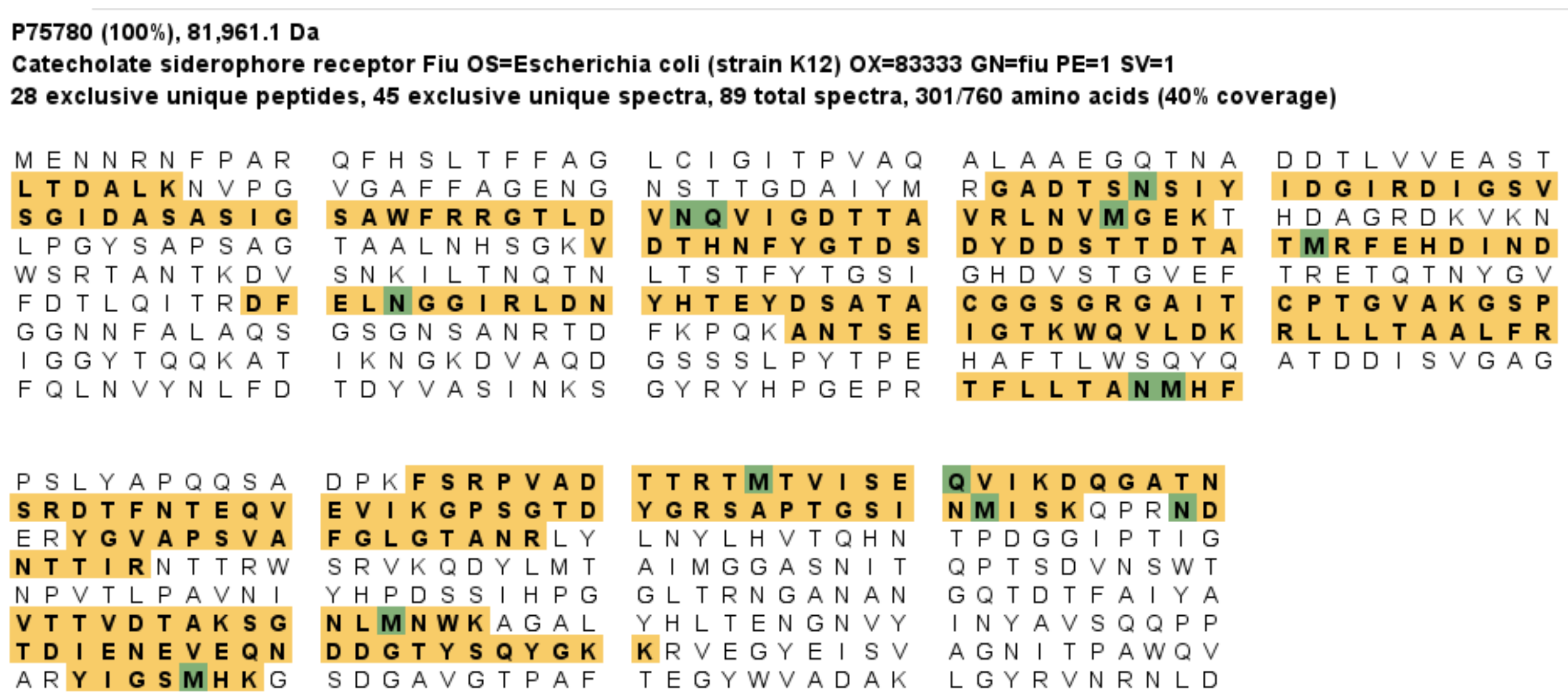

Figure S173. Identification of catecholate siderophore receptor Fiu by in-gel digestion and MS/MS of the band visualized at 100 kDa.

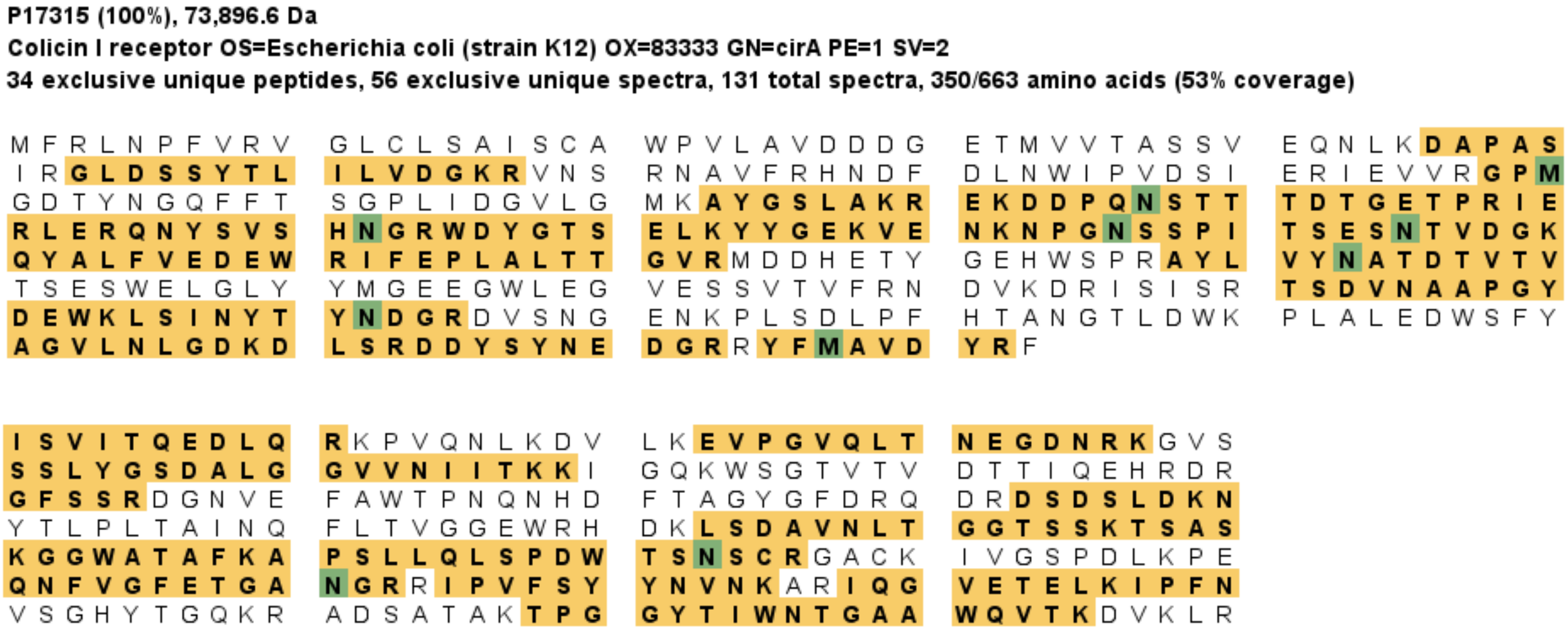

Figure S174. Identification of colicin I receptor CirA by in-gel digestion and MS/MS of the band visualized at 80 kDa.

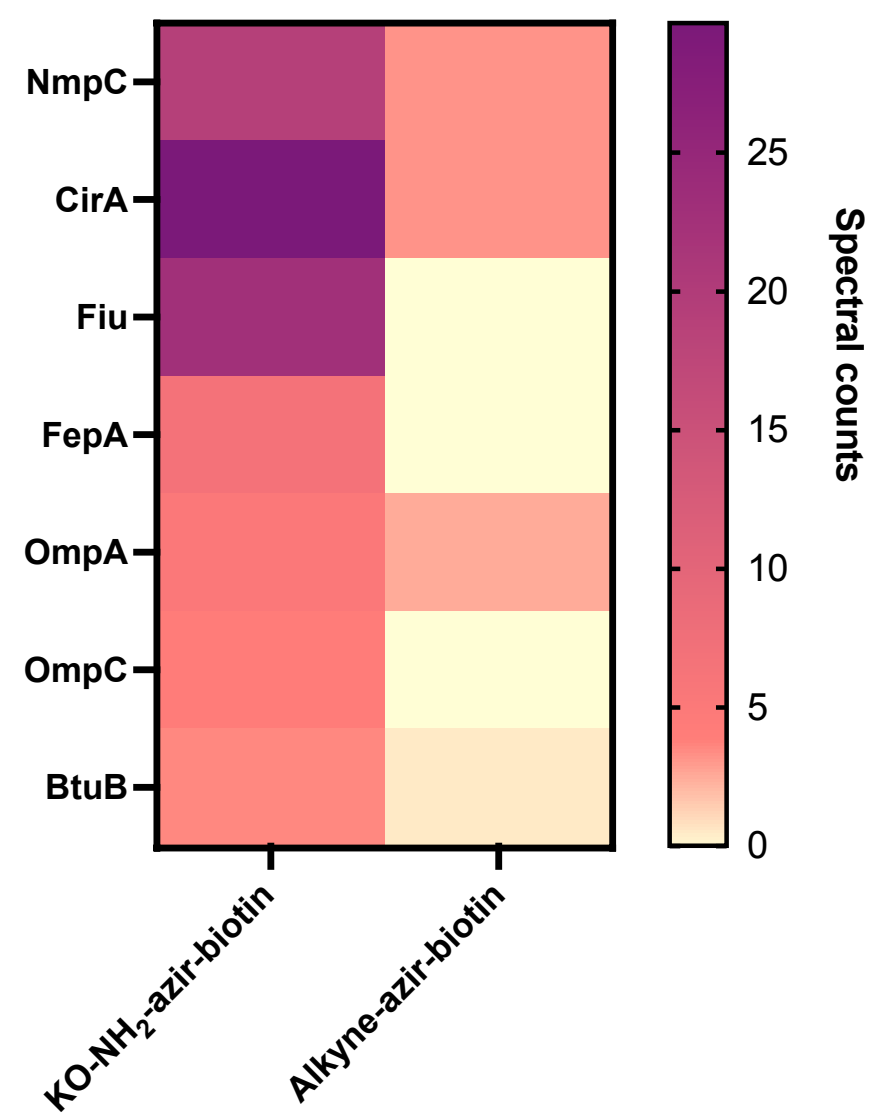

Figure S175. Heatmap of enriched outer membrane proteins from enrichment experiments conducted on *E. coli* K-12 with **KO-NH$_2$-azir-biotin** and **alkyne-azir-biotin** control.

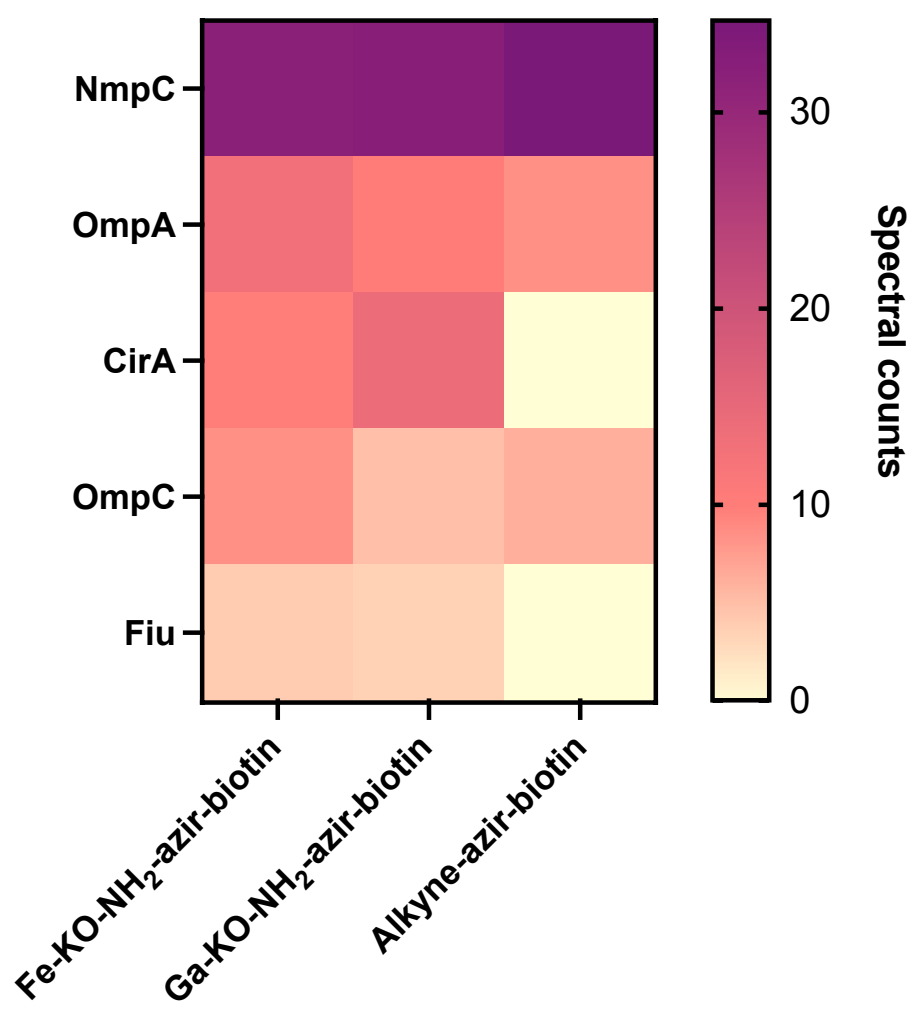

Figure S176. Heatmap of enriched outer membrane proteins from enrichment experiments conducted on *E. coli* K-12 with **[Fe(KO-NH$_2$-azir-biotin)]$^{3-}$, [Ga(KO-NH$_2$-azir-biotin)]$^{3-}$** and **alkyne-azir-biotin** control.

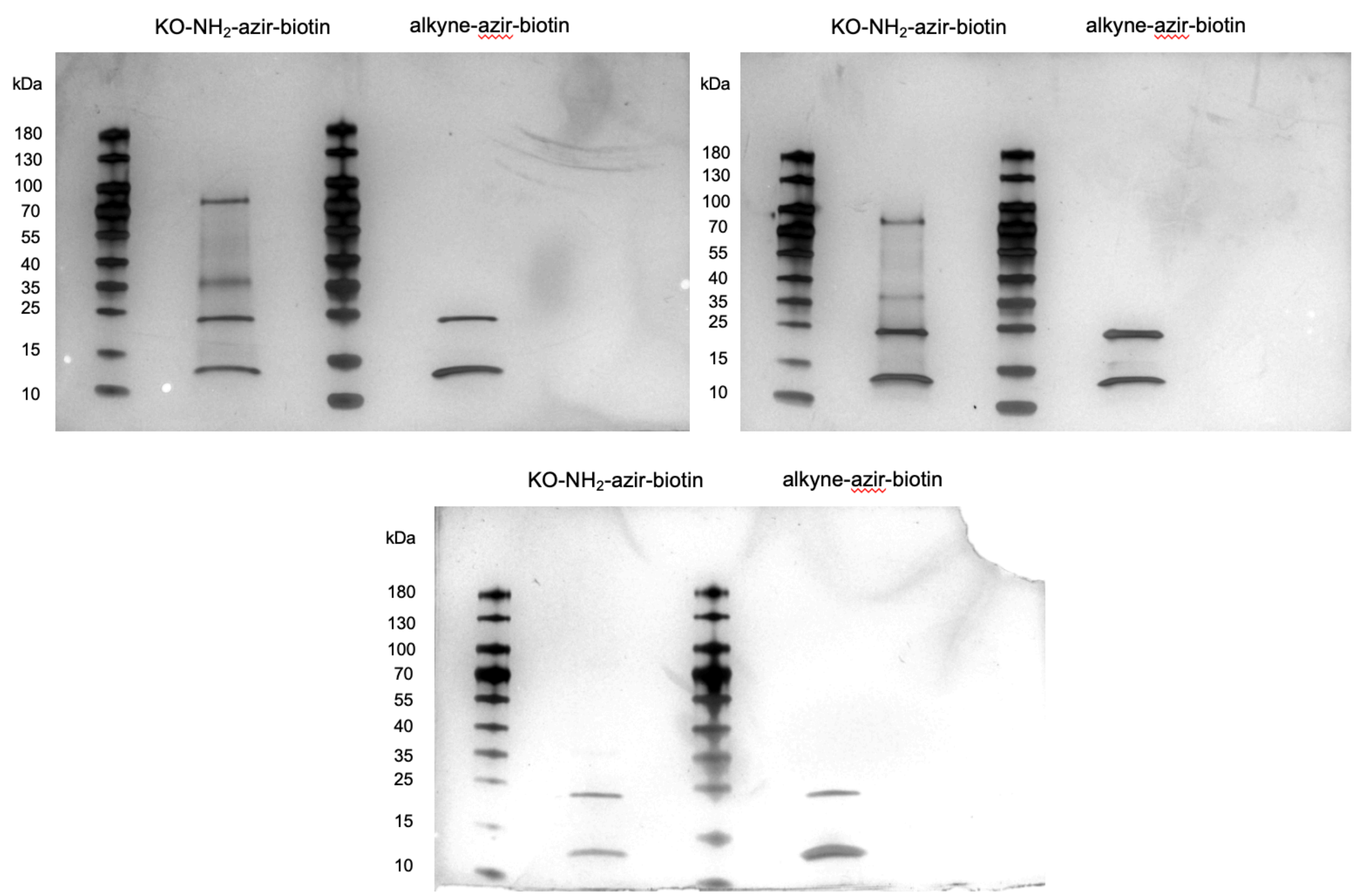

Figure S177. Enrichment of biotinylated proteins from *P. aeruginosa* PAO1 after live-cell incubation with **KO-NH$_2$-azir-biotin** and **alkyne-azir-biotin** control visualized by SDS-PAGE with silver staining (independent replicates).

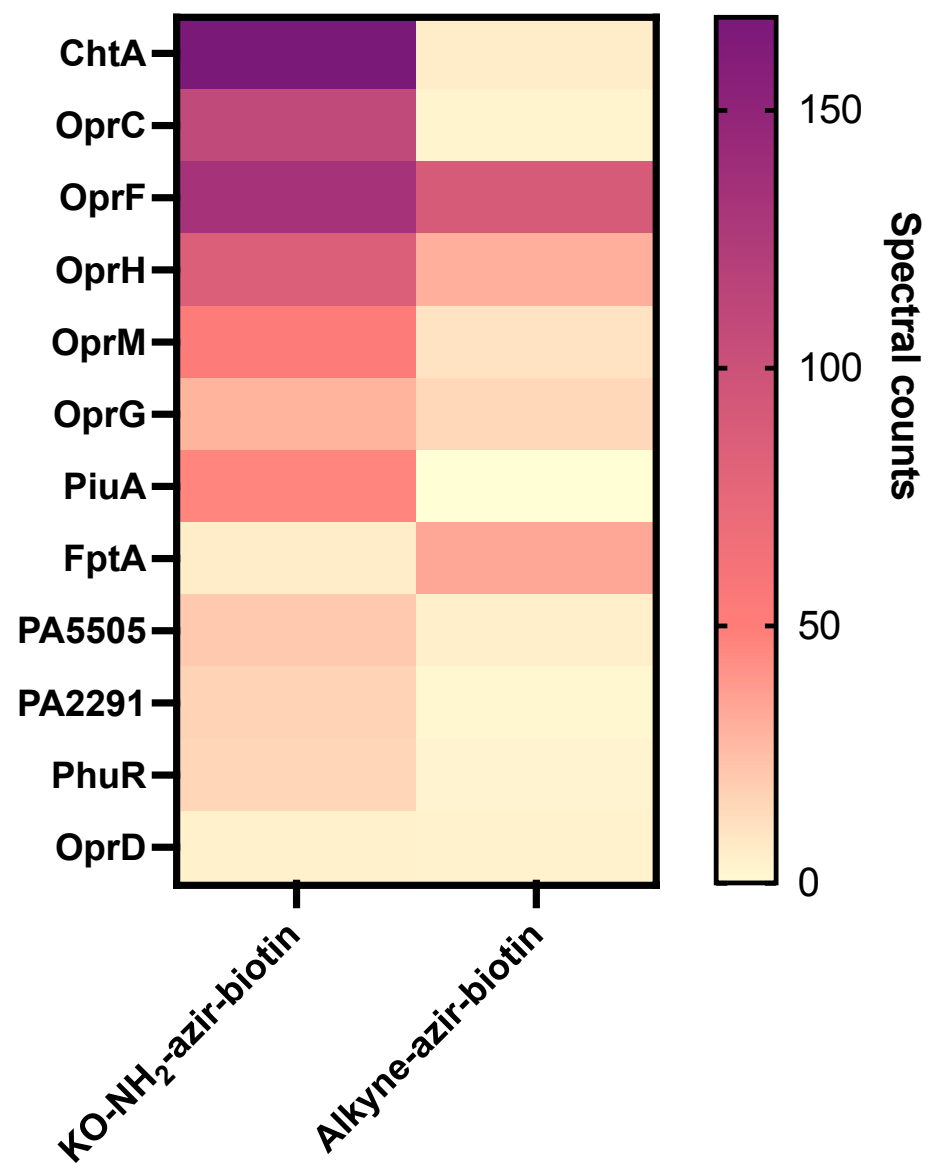

Figure S178. Heatmap of enriched outer membrane proteins from enrichment experiments conducted on *P. aeruginosa* PAO1 with **KO-NH₂-azir-biotin** and **alkyne-azir-biotin** control.

Table S29. Spectral counts and statistics analysis of top 50 identified proteins in enrichment experiments conducted in *E. coli* K-12 with **KO-NH$_2$-azir-biotin** and **alkyne-azir-biotin** control.

				Total Spectrum Count						
				Azir			KO-NH2			
#	Accession Number	Alternate ID	Molecular Weight	Azir-1	Azir-2	Azir-3	KO-NH2-1	KO-NH2-2	KO-NH2-3	Welch T-Test (p-value)
1	P0ABD8	accB	17 kDa	36	29	32	28	35	34	0.5
2	P21420	nmpC	40 kDa	3	5	2	19	16	22	0.002064338
3	P04949	fliC	51 kDa	8	17	9	43	15	18	0.125631361
4	P17315	cirA	74 kDa	6	4	0	58	15	16	0.101321832
5	P09373 [2]	pflB	85 kDa	0	0	0	0	9	0	0.211324865
6	P0A6P9	eno	46 kDa	3	16	7	15	8	6	0.42172122
7	P0A6F5	groL	57 kDa	3	10	9	13	8	7	0.262431321
8	P0A6S3	flgI	38 kDa	4	6	4	9	8	10	0.004194137
9	P11557	damX	46 kDa	0	5	3	10	8	4	0.056621851
10	P69776	lpp	8 kDa	4	3	5	15	7	6	0.09909773
11	P0A6Y8	dnaK	69 kDa	5	5	3	11	7	5	0.095398834
12	P0ABT2	dps	19 kDa	0	6	5	4	7	0	0.5
13	P06996	ompC	40 kDa	0	0	0	4	7	2	0.04821877
14	P0CE47 (+1)	tufA	43 kDa	4	4	4	6	6	4	0.09175171
15	P02943	lamB	50 kDa	0	0	0	0	6	3	0.112701665
16	P75780	fiu	82 kDa	0	0	0	47	5	16	0.106614257
17	P76116	yncE	39 kDa	4	10	0	33	5	12	0.143932319
18	P05825	fepA	82 kDa	0	0	0	12	5	3	0.067257678
19	P06129	btuB	68 kDa	0	2	0	6	5	0	0.12128762
20	P0C0V0	degP	49 kDa	10	18	15	6	4	4	0.022358788
21	P0AEX9	malE	43 kDa	0	8	11	2	4	0	0.158753362
22	P0A910	ompA	37 kDa	2	4	2	7	4	5	0.039077492
23	P06959	aceF	66 kDa	0	0	0	3	4	5	0.010102051
24	P0AFG8	aceE	100 kDa	0	0	0	0	4	0	0.211324865
25	P0AEU7	skp	18 kDa	0	3	0	6	4	2	0.061224214
26	P02930	tolC	54 kDa	0	4	0	5	4	0	0.229020368
27	P0A905	slyB	16 kDa	0	2	0	5	4	0	0.132092175
28	P02925	rbsB	31 kDa	2	4	0	7	3	4	0.09248121
29	P0A8A0	yebC	26 kDa	0	0	0	3	3	0	0.09175171
30	P0ACF0	hupA	10 kDa	0	2	0	2	3	0	0.21019801
31	P0A8Z0	yciA	14 kDa	0	0	0	0	3	2	0.099679615
32	P0A7V0	rpsB	27 kDa	0	2	2	4	3	2	0.066616281
33	P0A7R9	rpsK	14 kDa	3	4	3	5	3	6	0.133346514
34	P0A8T7	rpoC	155 kDa	0	0	0	3	3	2	0.007634036
35	P0AEE5	mglB	36 kDa	5	5	2	6	3	4	0.40754239
36	P0A9K9	slyD	21 kDa	0	0	0	0	3	0	0.211324865
37	P39180	flu	107 kDa	0	0	0	3	3	0	0.09175171
38	P76550	yffS	30 kDa	0	0	0	2	2	2	#DIV/0!
39	P68066	grcA	14 kDa	0	0	0	0	2	0	0.211324865
40	P0A7K2	rplL	12 kDa	0	2	2	2	2	2	0.211324865
41	P21179	katE	84 kDa	0	0	0	0	2	0	0.211324865
42	P60422	rplB	30 kDa	0	0	0	2	2	0	0.09175171
43	P0A817	metK	42 kDa	0	2	0	0	2	0	0.5
44	P07017	tar	60 kDa	0	3	0	2	2	0	0.398585479
45	P0AG44	rplQ	14 kDa	0	0	0	5	2	5	0.028595479
46	P0A6Q6	fabZ	17 kDa	0	0	0	3	2	0	0.099679615
47	P31057	panB	28 kDa	0	0	0	0	2	0	0.211324865
48	P0A7L0	rplA	25 kDa	0	0	0	7	2	0	0.143126786
49	P0A903	bamC	37 kDa	0	3	2	0	2	2	0.38955348
50	P02358	rpsF	16 kDa	0	0	0	3	2	0	0.099679615

Table S30. Spectral counts and statistics analysis of top 50 identified proteins in enrichment experiments conducted in *E. coli* K-12 with **Fe-KO-NH$_2$-azir-biotin, Ga-KO-NH$_2$-azir-biotin** and **alkyne-azir-biotin** control.

				Total Spectrum Count									Welch T-Test (p-value)		
				azir			Fe-KO-NH2			Ga-KO-NH2					
#	Accession Numb	Alternate ID	Molecular Weight	azir1	azir2	azir3	Fe1	Fe2	Fe3	Ga1	Ga2	Ga3	Fe/azir	Ga/azir	Fe/Ga
1	P0ABD8	accB	17 kDa	110	104	104	188	112	134	115	92	59	0.114111	0.19898	0.060736
2	P0A6Y8	dnaK	69 kDa	21	34	15	47	42	21	15	20	39	0.125209	0.44619	0.16484
3	P21420 [3]	nmpC	40 kDa	28	36	40	35	30	30	32	34	31	0.250301	0.290172	0.373392
4	P0ABT2	dps	19 kDa	21	20	33	41	27	27	25	23	32	0.163496	0.35605	0.209441
5	P04949	fliC	51 kDa	18	20	19	28	24	22	21	21	19	0.036396	0.103263	0.060111
6	P0A6P9	eno	46 kDa	16	20	14	27	17	18	14	12	26	0.174387	0.448822	0.286907
7	P0AEE5	mglB	36 kDa	19	30	25	21	16	12	12	14	11	0.057615	0.025858	0.130276
8	P0A6F5	groL	57 kDa	8	14	13	43	16	23	11	12	40	0.093747	0.215303	0.319597
9	P02925	rbsB	31 kDa	20	22	23	22	15	6	7	9	12	0.125908	0.00195	0.197859
10	P06959	aceF	66 kDa	0	2	2	17	14	7	7	7	64	0.027916	0.161849	0.27843
11	P0A910	ompA	37 kDa	6	12	8	16	14	9	10	6	15	0.094669	0.313893	0.235276
12	P32176 [2]	fdoG	113 kDa	2	7	3	35	13	22	5	10	12	0.043347	0.065626	0.071951
13	P02930	tolC	54 kDa	8	17	15	10	13	9	8	6	4	0.22122	0.049639	0.02445
14	P09152 [2]	narG	140 kDa	0	2	8	40	12	9	7	8	23	0.111478	0.102995	0.270308
15	P17315	cirA	74 kDa	0	0	0	7	11	12	20	12	10	0.011274	0.022233	0.16382
16	P0CE47 (+1)	tufA	43 kDa	3	10	3	12	11	13	11	11	24	0.048061	0.066511	0.261521
17	P0A6F3	glpK	56 kDa	6	8	9	13	11	12	7	10	20	0.009928	0.178414	0.470242
18	P76550	yffS	30 kDa	0	0	0	9	10	3	5	9	5	0.039261	0.020788	0.359967
19	P0A805	frr	21 kDa	9	12	10	15	10	8	13	12	8	0.394655	0.364574	0.5
20	P0ACD4	iscU	14 kDa	3	7	4	8	9	5	9	8	6	0.095872	0.060396	0.417476
21	P06996 [4]	ompC	40 kDa	3	10	6	10	9	7	5	7	3	0.187798	0.302842	0.034695
22	P02943	lamB	50 kDa	4	9	10	15	9	3	5	3	4	0.378176	0.089505	0.142186
23	P0AEU7	skp	18 kDa	4	7	11	8	9	8	8	10	8	0.336209	0.292936	0.342799
24	P0ADG7	guaB	52 kDa	0	0	0	25	8	13	3	8	13	0.04666	0.054638	0.14578
25	P0A7K2	rplL	12 kDa	3	6	8	8	8	5	0	4	5	0.24838	0.137368	0.05223
26	P75780	fiu	82 kDa	0	0	0	0	8	4	6	3	2	0.112702	0.046368	0.453099
27	P0A6Q6	fabZ	17 kDa	0	5	0	9	8	4	3	4	11	0.039117	0.117361	0.377298
28	P0A7L0	rplA	25 kDa	3	6	3	4	8	2	6	6	10	0.381435	0.060815	0.149404
29	P0C0V0	degP	49 kDa	22	38	34	7	7	6	5	6	10	0.0177	0.013905	0.424697
30	P0A7J3	rplJ	18 kDa	0	2	2	3	7	4	5	5	8	0.045153	0.011519	0.221652
31	P11349 [2]	narH	58 kDa	0	0	4	22	7	6	8	9	16	0.088876	0.020891	0.457665
32	P0ACF8	hns	16 kDa	4	9	0	10	7	7	4	4	3	0.146668	0.411277	0.01919
33	P0A6F9	groS	10 kDa	5	11	10	10	7	5	0	7	6	0.301736	0.103545	0.162776
34	P0A6M8	fusA	78 kDa	0	5	3	4	7	3	6	2	21	0.175294	0.174803	0.240117
35	P09127	hemX	43 kDa	3	7	16	13	7	4	5	9	10	0.447071	0.441793	0.5
36	P0A6A8	acpP	9 kDa	0	6	3	6	7	6	5	6	6	0.095269	0.130697	0.1151
37	Q46871	yqjH	29 kDa	0	0	0	4	6	5	4	3	4	0.006536	0.004082	0.066792
38	P0A7M9	rpmE	8 kDa	0	0	0	6	6	3	6	5	3	0.018875	0.016954	0.407542
39	P0AAJ5 [2]	fdoH	33 kDa	0	2	3	13	6	5	0	2	4	0.058	0.415269	0.062491
40	P45523	fkpA	29 kDa	9	13	7	9	6	5	4	5	6	0.120759	0.053289	0.151671
41	P11557	damX	46 kDa	3	5	4	8	6	6	5	9	6	0.020027	0.071624	0.5
42	P0A7V8	rpsD	23 kDa	0	0	0	3	6	0	3	3	3	0.112702	#DIV/0!	0.5
43	P60422	rplB	30 kDa	0	2	4	7	6	4	5	4	11	0.034695	0.077209	0.351797
44	P31554	lptD	90 kDa	0	2	6	6	6	2	2	3	0	0.210198	0.32383	0.072329
45	P69441	adk	24 kDa	0	5	4	9	6	2	3	4	0	0.178474	0.374887	0.12277
46	P60624	rplX	11 kDa	0	7	6	5	6	5	2	2	5	0.346825	0.31014	0.067029
47	P0A6S3	flgI	38 kDa	6	4	10	7	6	4	4	6	9	0.32383	0.445665	0.359441
48	P0A8A0	yebC	26 kDa	0	3	0	12	5	6	7	8	7	0.037464	0.008124	0.446726
49	P0AFM2	proX	36 kDa	2	6	2	9	5	4	0	2	2	0.12998	0.136946	0.037715
50	P77754	spy	18 kDa	0	0	0	3	5	5	8	5	0	0.01143	0.102206	0.5

Table S31. Spectral counts and statistics analysis of top 50 identified proteins in enrichment experiments conducted in *P. aeruginosa* PAO1 with **KO-NH_2-azir-biotin** and **alkyne-azir-biotin** control.

				Total Spectrum Count						
				azir			KO-NH2			Welch T-Test (p-value)
#	Accession Number	Alternate ID	Molecular Weight	azir-1	azir-2	azir-3	KO-NH2-1	KO-NH2-2	KO-NH2-3	
1	Q9I762	PA0070	32 kDa	25	20	58	346	273	362	0.001657936
2	Q9HVC0	PA4675	81 kDa	5	2	12	170	235	99	0.026749407
3	G3XD89	oprC	79 kDa	5	3	4	110	174	43	0.054453941
4	P13794	oprF	38 kDa	59	85	126	151	141	106	0.077928081
5	Q9HW93 [20]	pctC	68 kDa	23	21	54	151	139	38	0.078689982
6	Q9HXI0	yajC	12 kDa	2	0	4	22	126	15	0.141049492
7	P37799	accB	16 kDa	71	213	127	43	109	57	0.120972679
8	G3XD11	oprH	22 kDa	27	24	43	76	105	76	0.006865685
9	Q9HV43	dnaK	68 kDa	22	66	57	64	90	51	0.16095977
10	Q9HXZ5 [2]	eno	45 kDa	3	11	27	55	90	32	0.049697779
11	Q9HW32	icmP	47 kDa	12	11	43	75	75	46	0.019456782
12	G3XD17	dsbC	26 kDa	0	0	3	63	69	82	0.002566759
13	Q9HZA6	fimV	97 kDa	3	2	18	58	68	35	0.012299818
14	P21175	braC	40 kDa	18	21	29	68	66	67	0.002209608
15	Q9HUJ1	PA4974	53 kDa	0	3	7	35	66	15	0.068860198
16	P30718	groL	57 kDa	8	95	30	49	64	35	0.434788456
17	Q9I120 [2]	PA2462	573 kDa	0	3	6	51	64	21	0.038601308
18	Q9I5Z9	PA0537	22 kDa	3	3	8	43	61	56	0.003719915
19	Q51487	oprM	53 kDa	7	6	19	54	59	47	0.000811812
20	Q9HVA2	ilvC	36 kDa	3	9	35	33	58	32	0.062201981
21	Q9HVZ2	PA4423	66 kDa	0	2	0	41	57	15	0.046883334
22	Q9HXY5	PA3647	19 kDa	2	2	12	55	55	54	0.002110512
23	Q9HVY6	PA4429	29 kDa	0	3	4	40	53	35	0.006911485
24	Q9I2T8	ppiD	69 kDa	0	8	10	39	53	14	0.057675444
25	Q9I4W2	PA1011	43 kDa	6	6	32	57	51	27	0.037087223
26	Q59638	aceF	57 kDa	11	28	36	45	48	36	0.059390423
27	Q9HXM5	guaB	52 kDa	0	7	10	21	47	7	0.11841299
28	Q9HWW1	oprG	25 kDa	10	16	21	32	46	12	0.140430848
29	Q9HYX8	PA3262	27 kDa	15	21	32	55	44	31	0.039631682
30	P11221	oprI	9 kDa	9	17	29	59	43	50	0.006757463
31	Q9I5A7	PA0833	25 kDa	0	0	6	41	42	21	0.016384647
32	G3XCY8	PA4514	82 kDa	0	0	0	62	41	38	0.012422948
33	G3XD47	aotJ	28 kDa	4	10	18	37	40	26	0.007929135
34	Q9HVH0	PA4621	104 kDa	0	0	3	30	40	19	0.019420061
35	Q9I4S1	PA1053	16 kDa	15	13	11	32	38	24	0.01861713
36	Q9HXU8	lptF	29 kDa	7	11	14	37	38	18	0.038905216
37	P72151	fliC	49 kDa	34	61	46	23	37	32	0.080202764
38	Q9HW30	PA4372	38 kDa	3	3	7	31	37	16	0.028709502
39	Q9I690	PA0423	21 kDa	0	6	19	26	36	34	0.016220342
40	Q9HVA8	hitA	36 kDa	0	0	11	29	36	33	0.002724355
41	Q9HUM9	rpsF	16 kDa	0	2	7	19	35	17	0.027323568
42	P42257	pilJ	73 kDa	10	14	22	35	33	15	0.092415859
43	Q9HWC8	rplL	12 kDa	0	7	49	3	33	3	0.387188885
44	Q14T72	fdnG	114 kDa	0	0	0	41	33	22	0.014183976
45	G3XDB2	cpoB	29 kDa	0	2	4	24	33	18	0.013758767
46	Q9I4Z4	pal	18 kDa	8	0	29	29	31	27	0.095934262
47	Q9I787	PA0045	24 kDa	3	0	7	35	31	26	0.000743055
48	Q9I3M9	cycH	43 kDa	0	0	2	19	31	14	0.026237459
49	Q9HVY4	PA4431	21 kDa	0	2	5	15	31	11	0.052569922
50	P04739	pilA	16 kDa	12	35	19	38	30	21	0.208723389

6. Efficacy assessment in bioluminescent E. coli XEN 14 infected mice

6.1.1 Sample preparation

Cefiderocol was prepared in 10% DMSO in DPBS. Concentration of **cefiderocol** was analyzed using the UV-Vis spectrophotometry at 259 nm (ε = 9430 M^{-1} cm^{-1}) to calculate the stock concentration. **KO-NH_2-Amp** stock concentration was determined by UV-Vis at 316 nm (ε = 9500 M^{-1} cm^{-1}) and prepared in 10 % DMSO and 40 % PEG400 in DPBS. Stock of **[Ga(KO-NH_2-Amp)]$^{3-}$** was determined by ICP-OES based on a 6-point standard with respect to gallium and lines of best fit were found with R^2 of 0.999. Sample concentration was found based on this calibration curve and prepared with 10% DMSO and 40% PEG400 in DPBS.

Table S32. Conversion table for doses of test compounds in mg/kg to µmol/kg.

Compound	Single dose (mg/kg)	Single dose (µmol/kg)
**Cefiderocol**	70.71	94
**KO-NH_2-Amp**	240.35	190
**[Ga(KO-NH_2-Amp)]$^{3-}$**	252.52	190

6.1.2 Bacteria inoculum preparation and compounds administration

E. coli Xen14 was inoculated into MHB iron-deficient medium (10 mL) and incubated in a shaking incubator at 37°C and 210 rpm overnight. The overnight cultures were then diluted 1:100 in fresh MHB iron-deficient medium and incubated at 37°C (210 rpm) until OD_{600} = 0.4 (2 h) was reached corresponding to a concentration of 2 x 10^8 CFU/mL. Bacterial cells were harvested by centrifugation (34 000 rpm, 3 min) and washed with sterilized MHB. Final inoculums were generated by diluting bacteria to the desired concentrations in MHB. Four weeks old female balb/c mice were anesthetized with isoflurane and a 50 µL aliquot of bacterial inoculum (2 x 10^8 CFU/mL) was injected in the left triceps of the mice.

Test compounds were administered subcutaneously 1 h and 25 h post bacterial inoculation. The study included four groups of mice: saline as a negative control (5 mice); **cefiderocol**: 94 µmol kg^{-1} (4 mice); **KO-NH_2-Amp**: 190 µmol kg^{-1} (5 mice); and [**Ga(KO-NH_2-Amp)**]$^{3-}$: 190 µmol kg^{-1} (5 mice). Treatment progression was monitored for 3 days post-infection. The mice were imaged on a small animal imager to assess treatment efficiency. After 3 days, mice were euthanized, and infected muscles from each cohort were extracted and plated for CFU determination.

6.1.3 Bioluminescent imaging

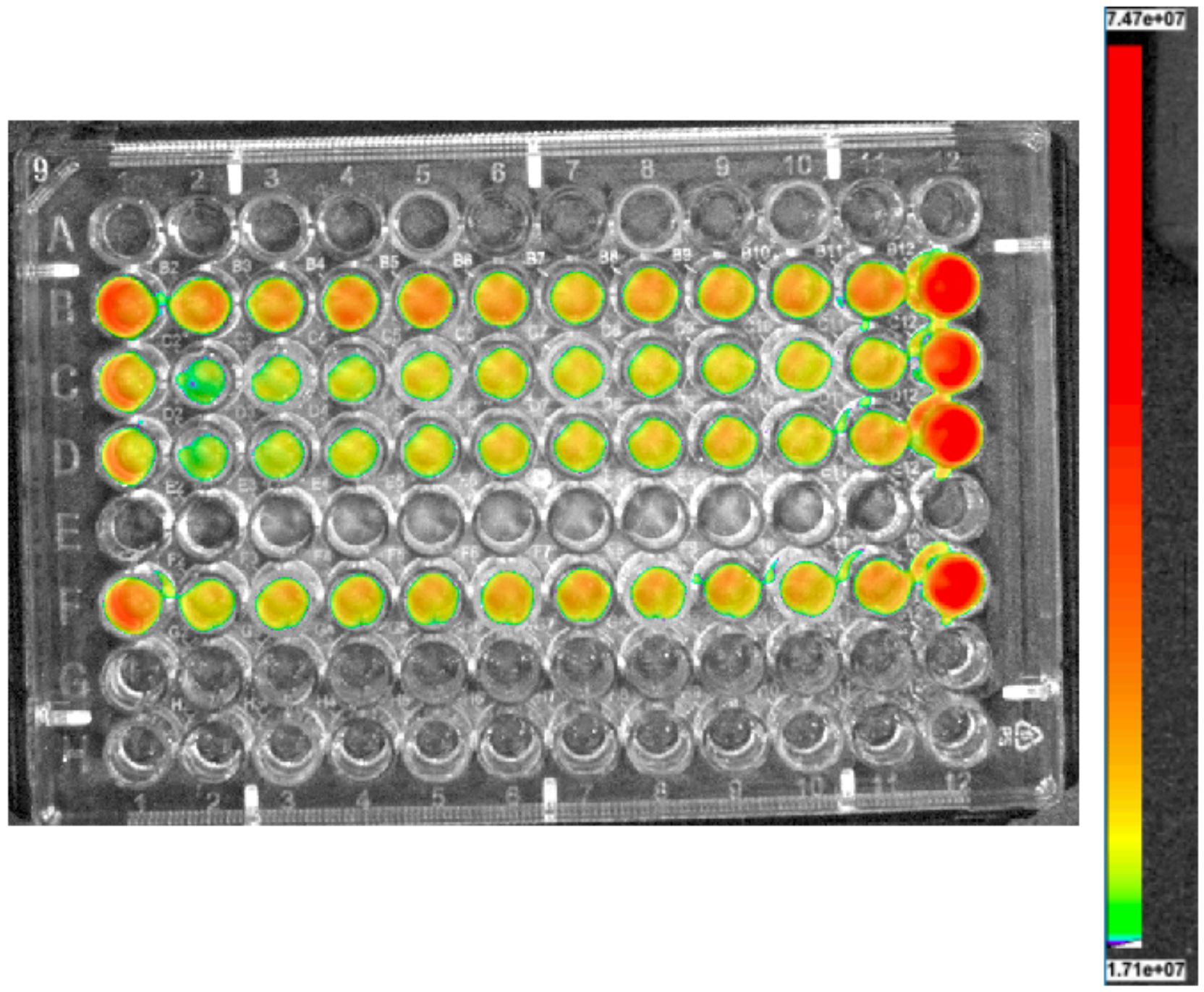

Figure S179. Bioluminescence image of **ampicillin** in 96 well plate tested in *E. coli* Xen14 and imaged on a Lago X small animal scanner.

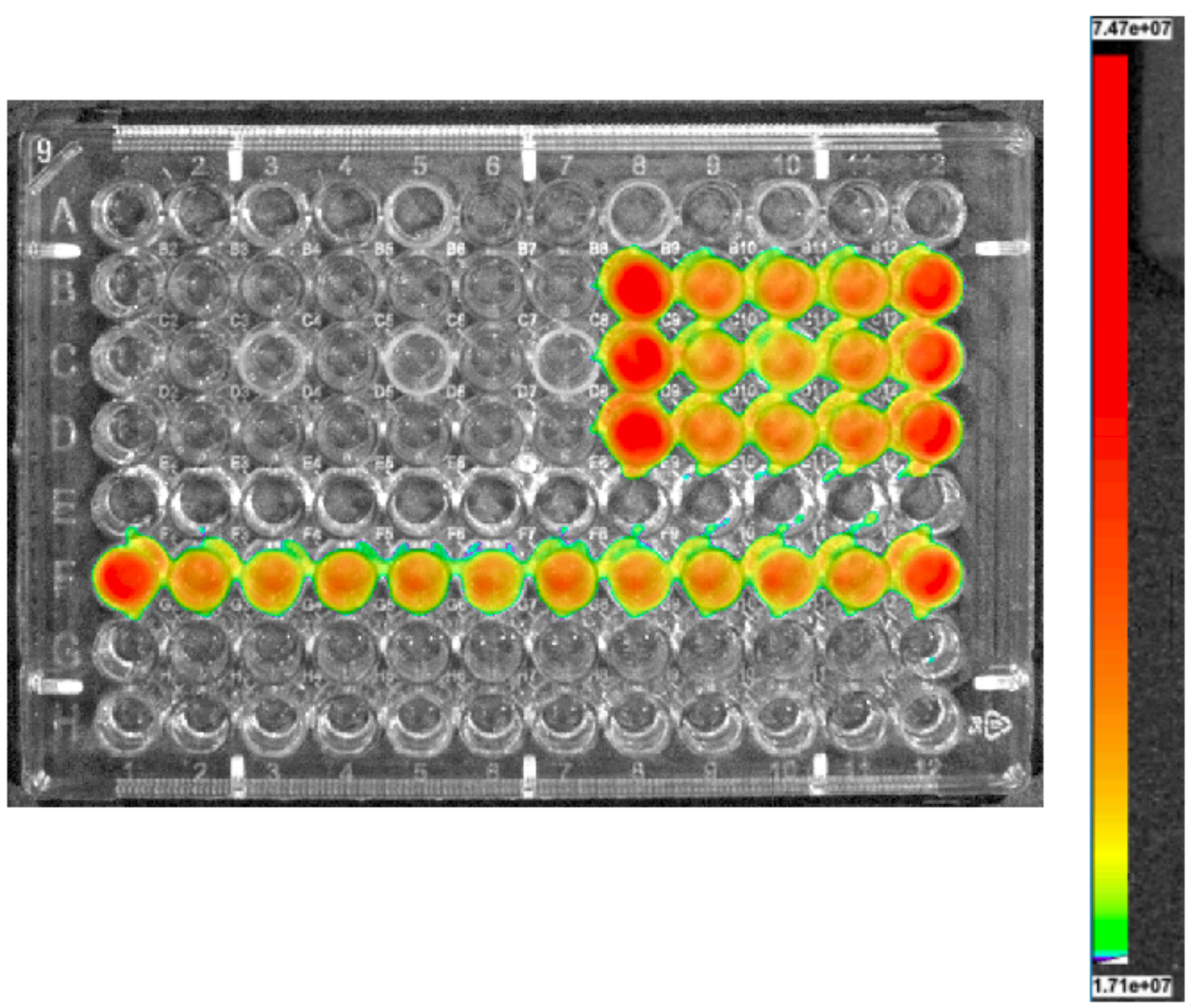

Figure S180. Bioluminescence image of **cefiderocol** in 96 well plate tested in *E. coli* Xen14 and imaged on a Lago X small animal scanner.

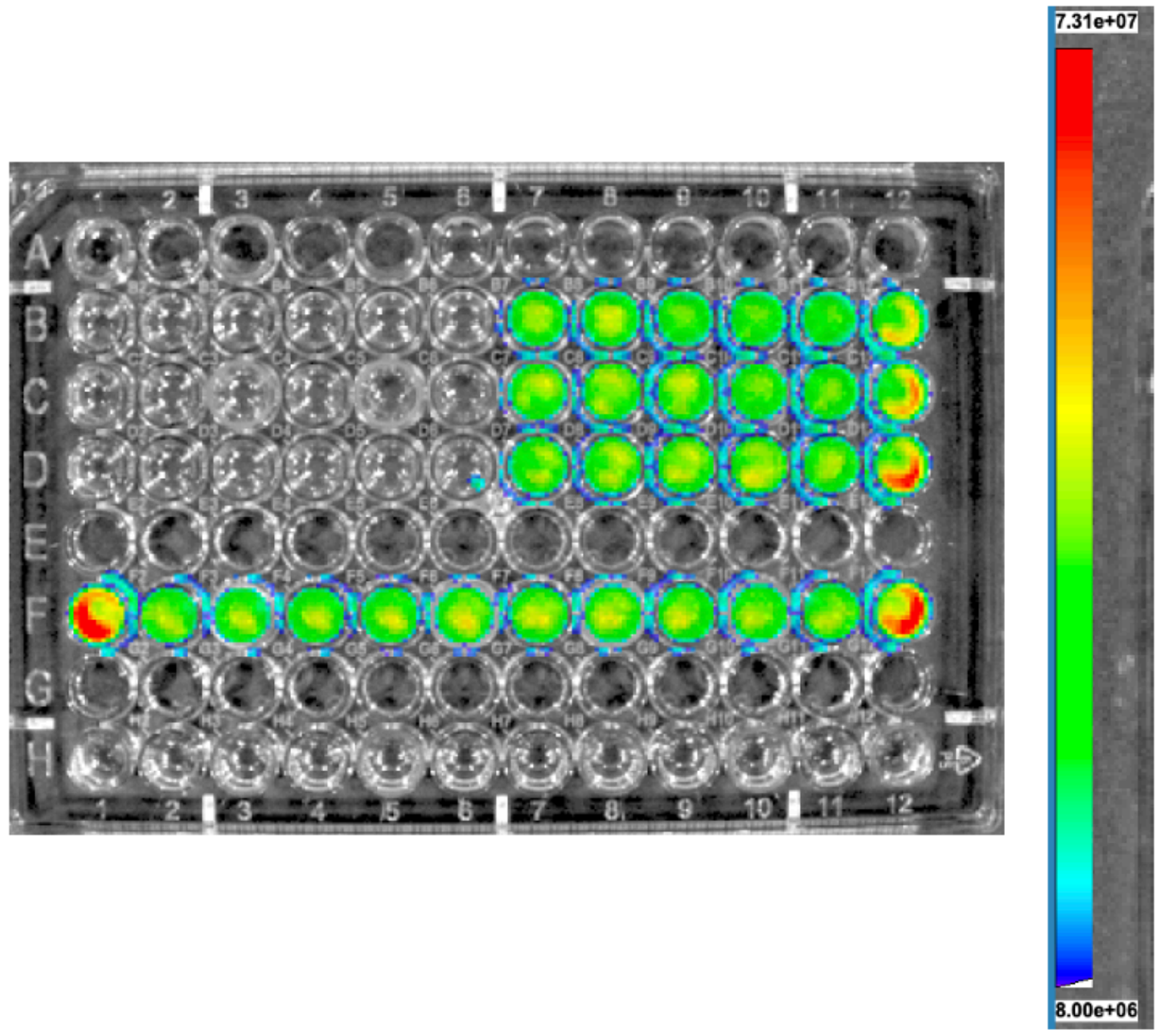

Figure S181. Bioluminescence image of **KO-NH$_2$-Amp** in 96 well plate tested in *E. coli* Xen14 and imaged on an IVIS small animal scanner.

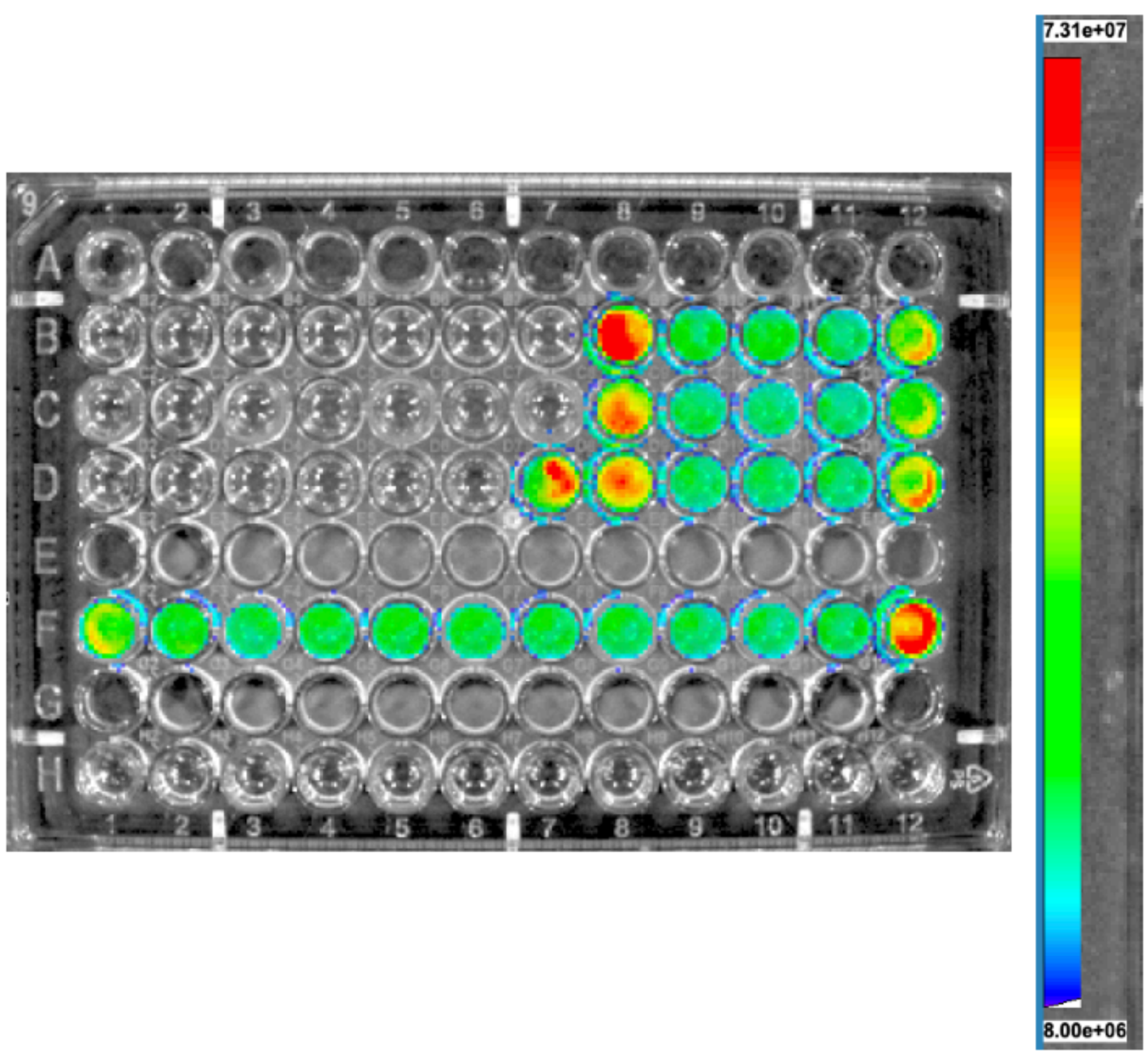

Figure S182. Bioluminescence image of **[Ga(KO-NH$_2$-Amp)]$^{3-}$** in 96 well plate tested in *E. coli* Xen14 and imaged on an IVIS small animal scanner.

Mice were imaged every 1 h in the first 6-h time points, every 2 h until 12-h time points, at 24-h, at 48-h, and at 72-h using Lago X small animal imager from Bruker company. Infected muscles from mice were plated for CFU determination and the dilution plate was also imaged on IVIS animal imager after overnight incubation at 37ºC for 18h. All scans were collected with blocked excitation filter, open emission filter (from 515 nm to 840 nm) and collection time t = 3 minutes (mice) and 1 minute (bacterial plates). Images were analyzed with Aura Analysis software from Spectral Instruments Imaging.

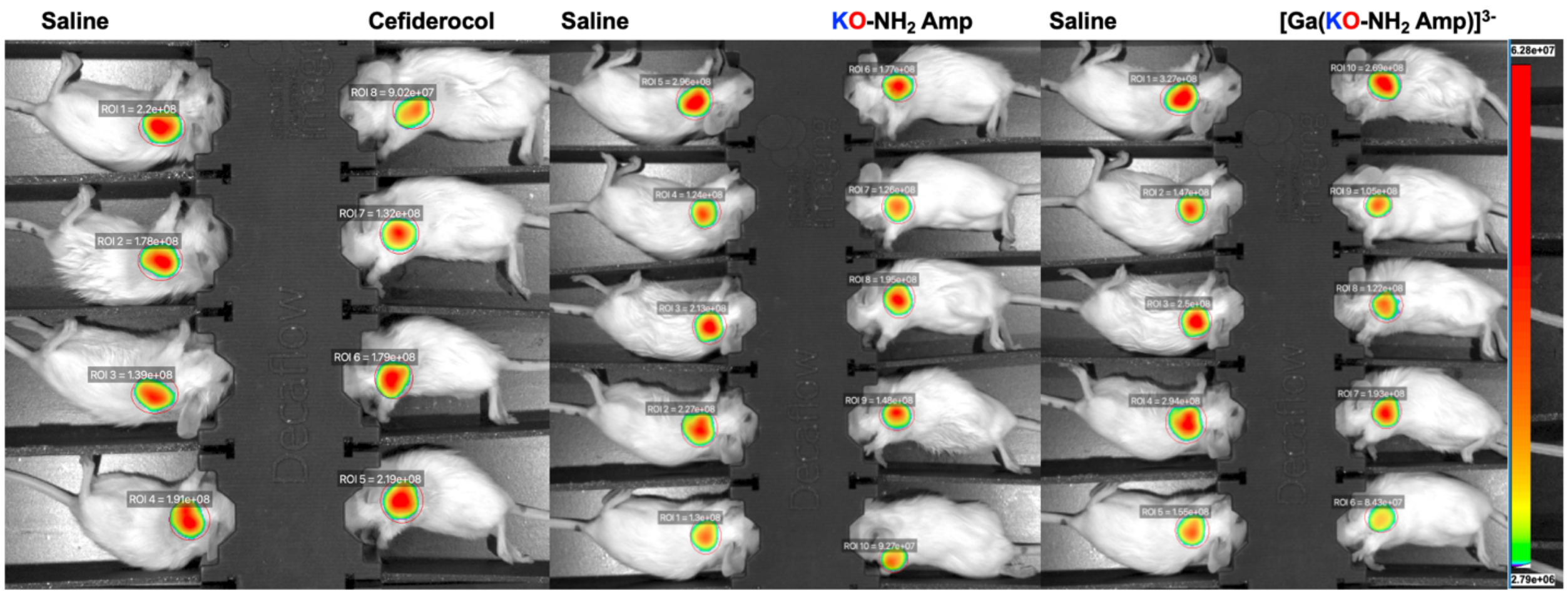

Figure S183. All cohorts compared to saline control at t = 0.5 h.

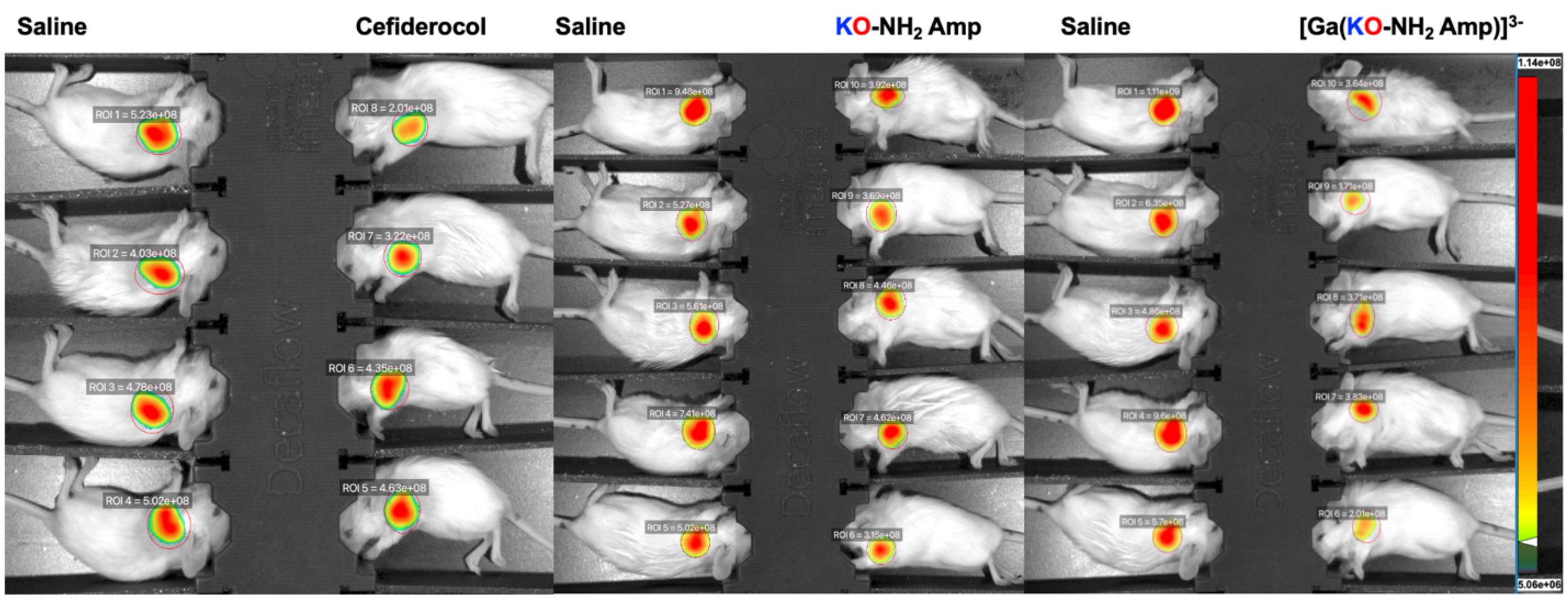

Figure S184. All cohorts compared to saline control at t = 1 h.

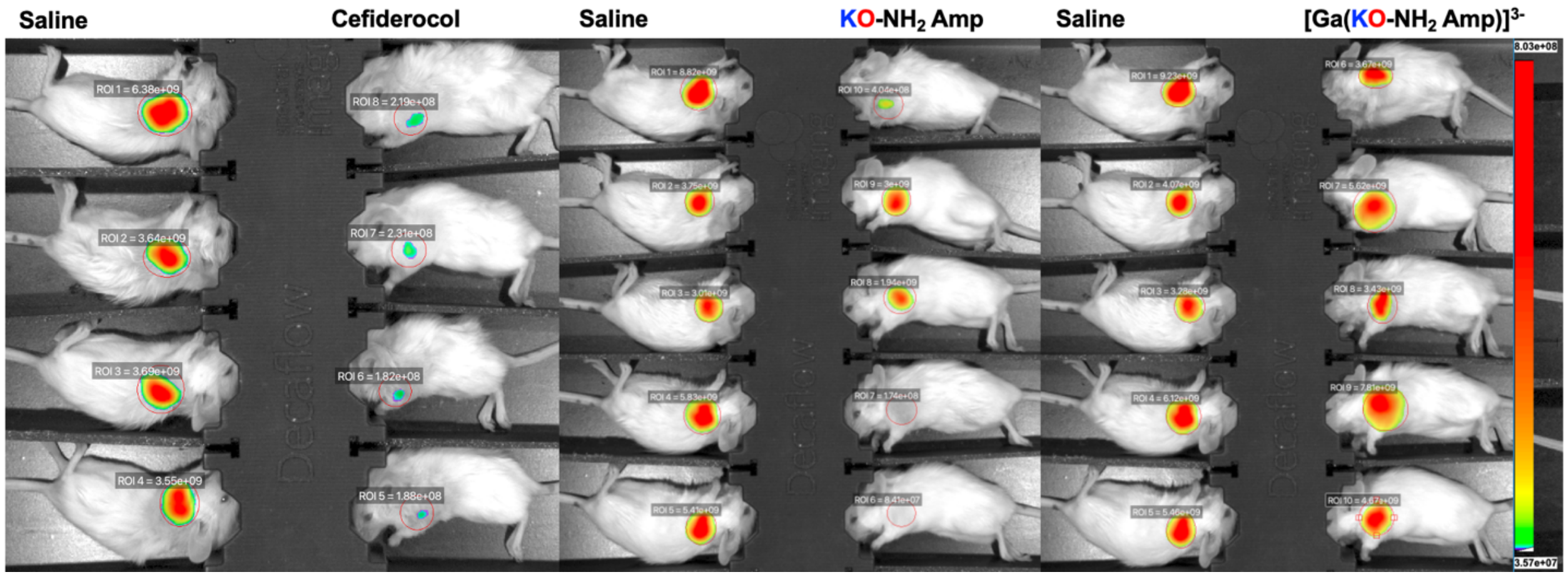

Figure S185. All cohorts compared to saline control at t = 3 h.

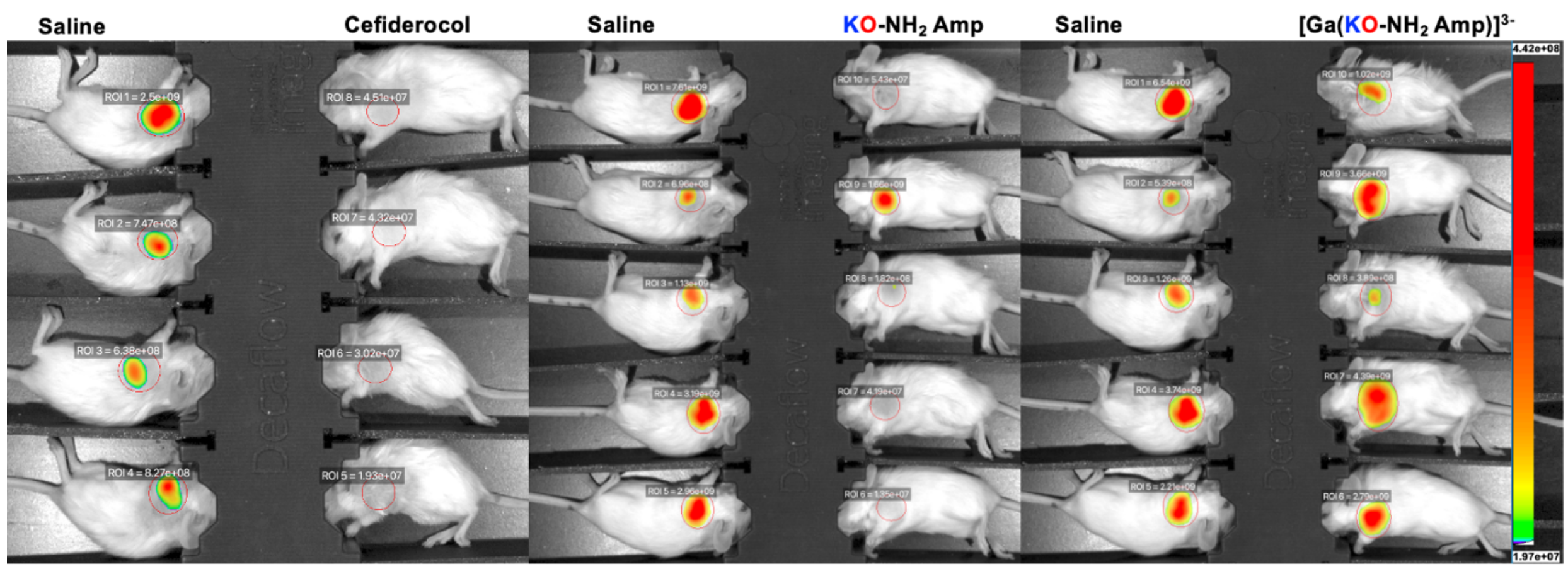

Figure S186. All cohorts compared to saline control at t = 6 h.

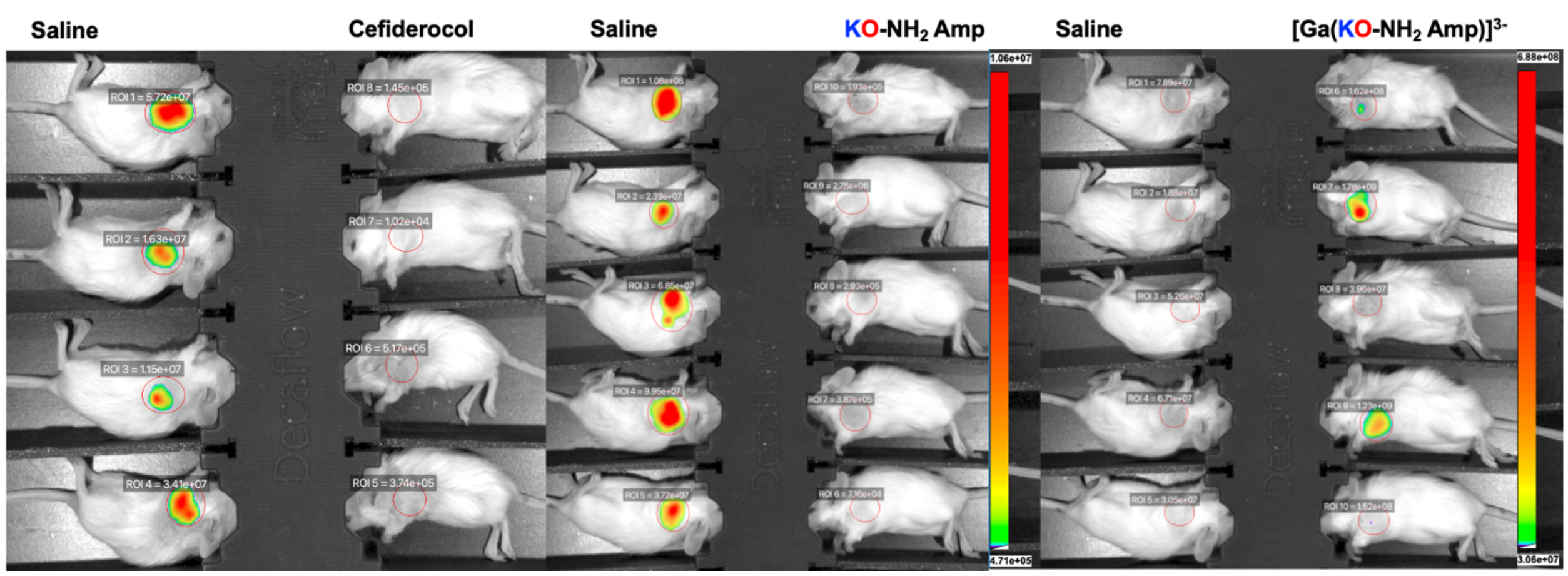

Figure S187. All cohorts compared to saline control at t = 12 h.

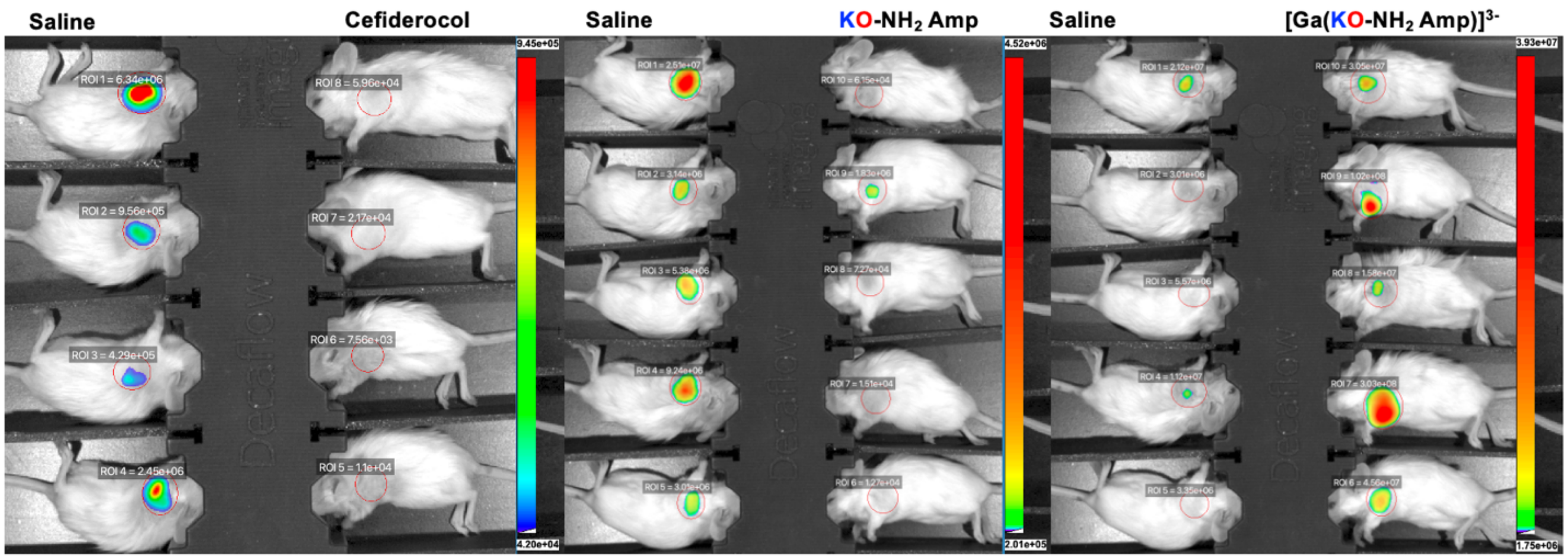

Figure S188. All cohorts compared to saline control at t = 24 h.

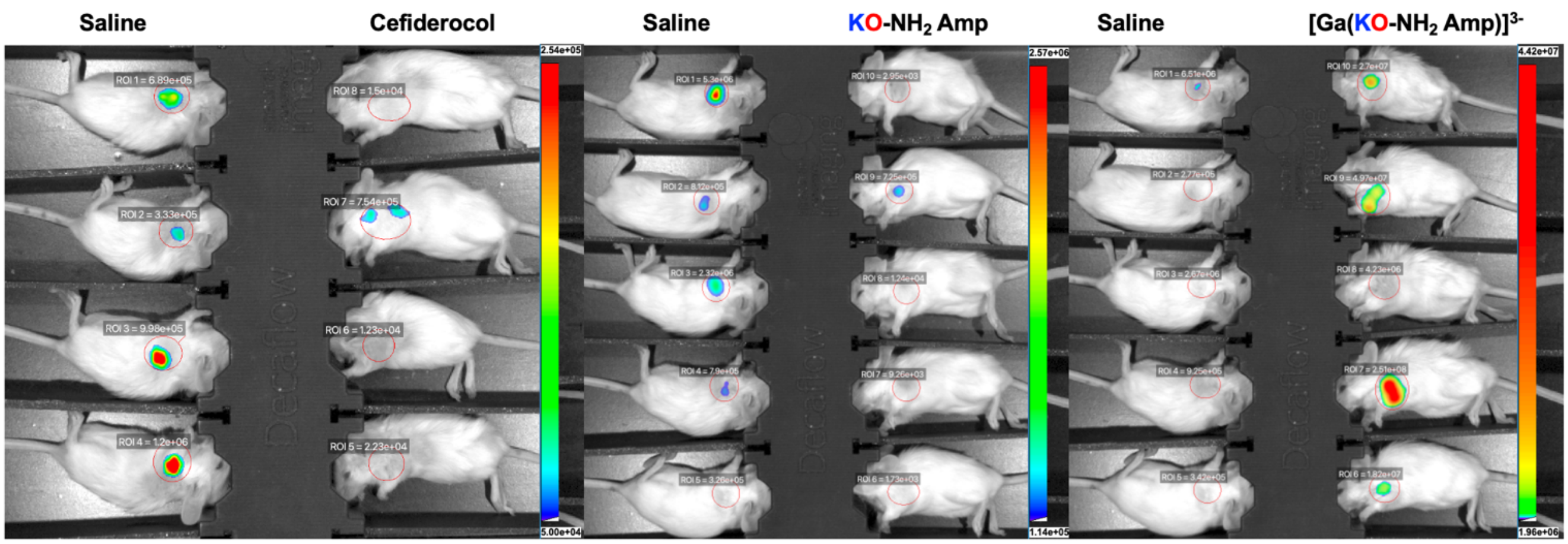

Figure S189. All cohorts compared to saline control at t = 48 h.

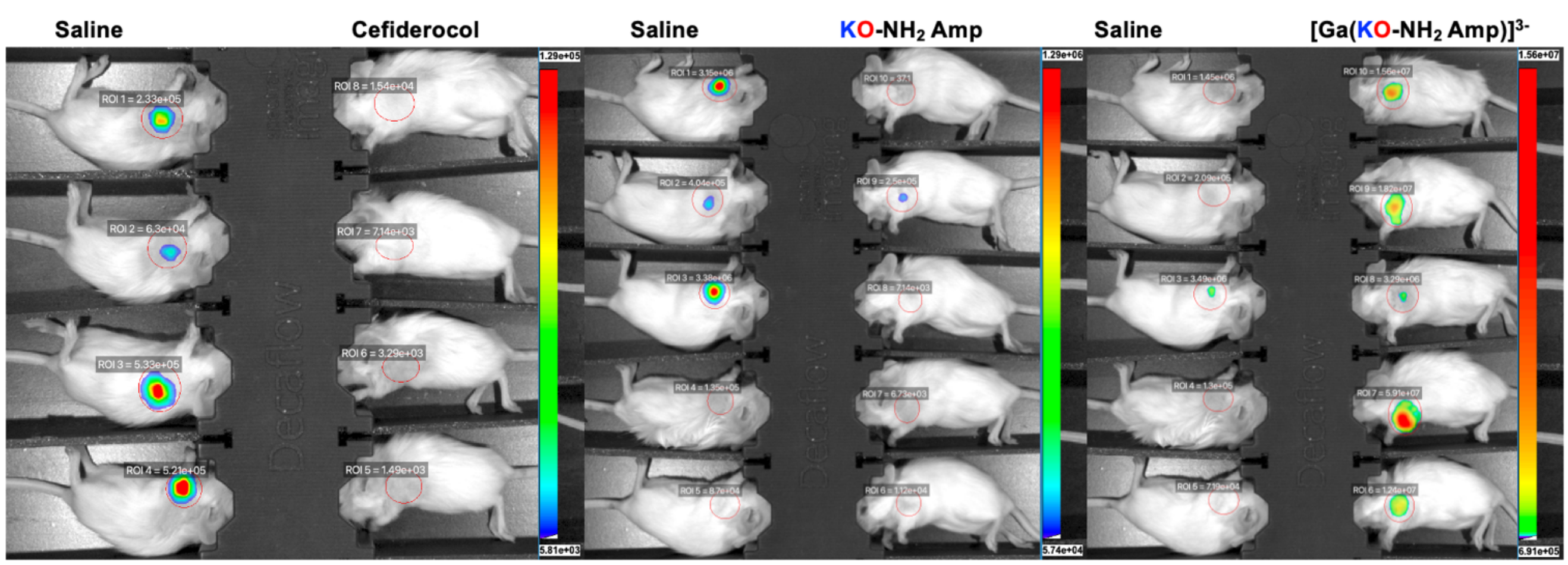

Figure S190. All cohorts compared to saline control at t = 72 h.

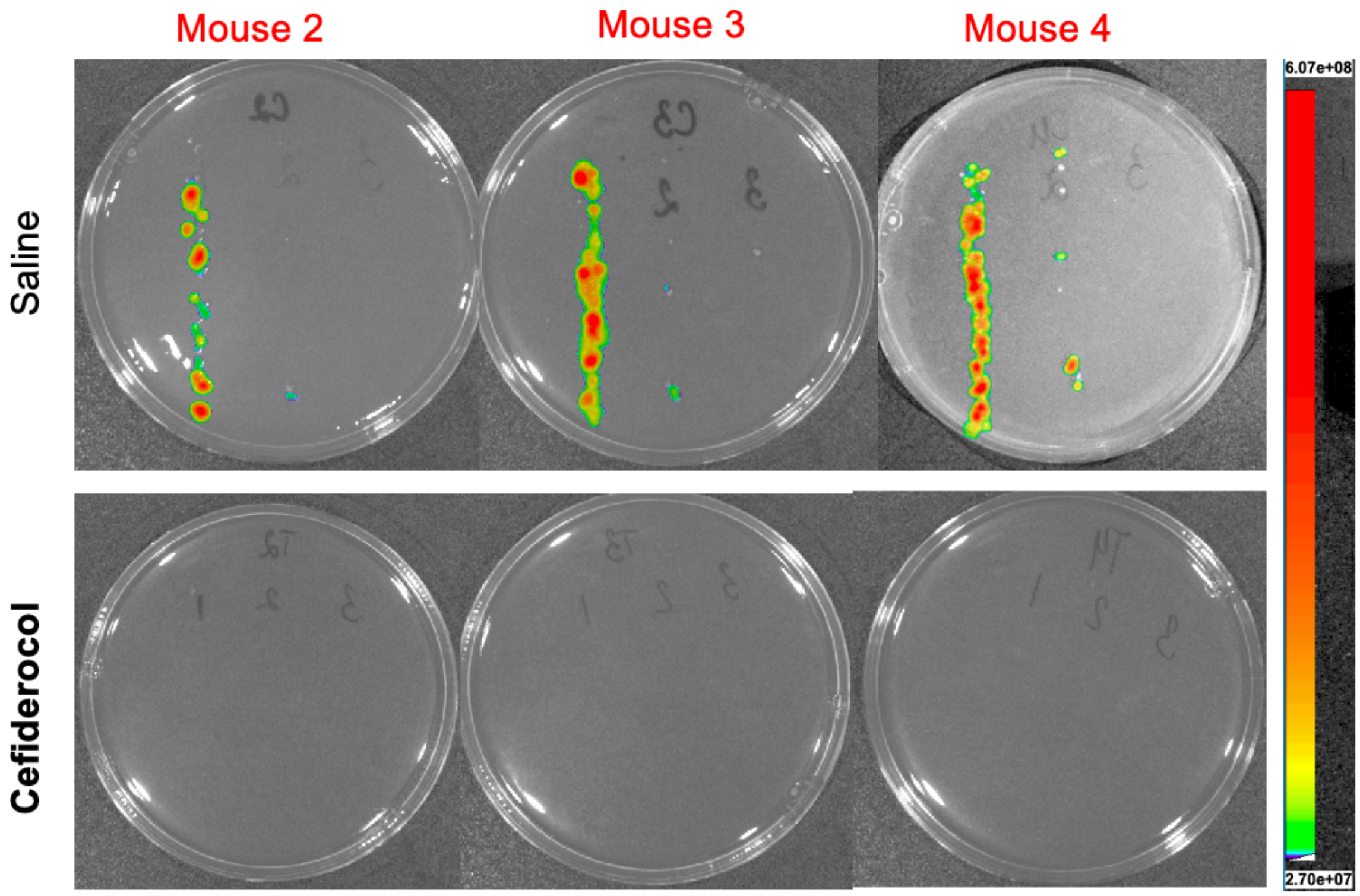

Figure S191. Dilution plates of infected muscle from saline and cefiderocol cohorts (t = 96 h)

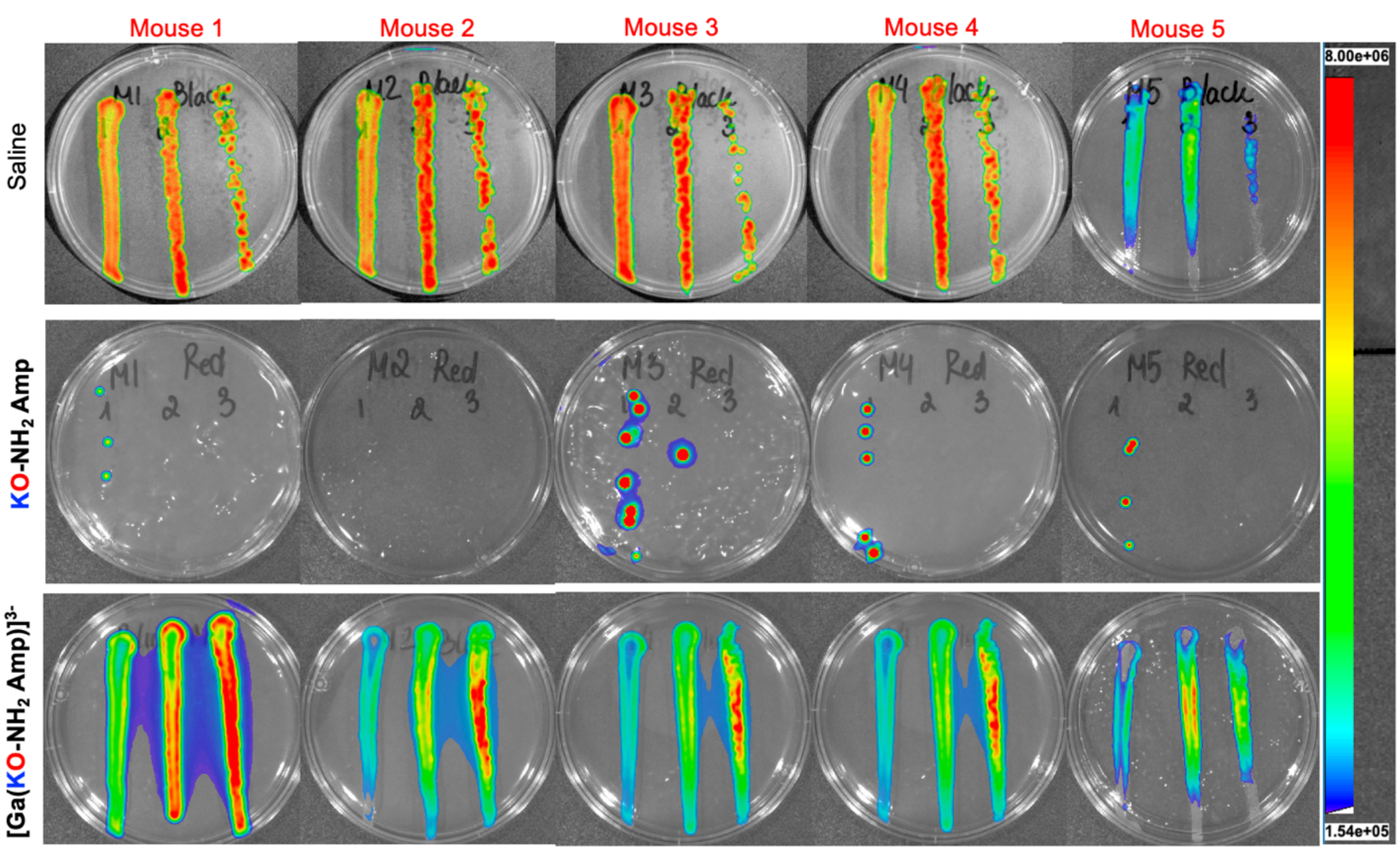

Figure S192. Dilution plates of infected muscle from saline, **KO-NH_2-Amp**, and **[Ga(KO-NH_2-Amp)]$^{3-}$**.

6.1.4 CFU determination

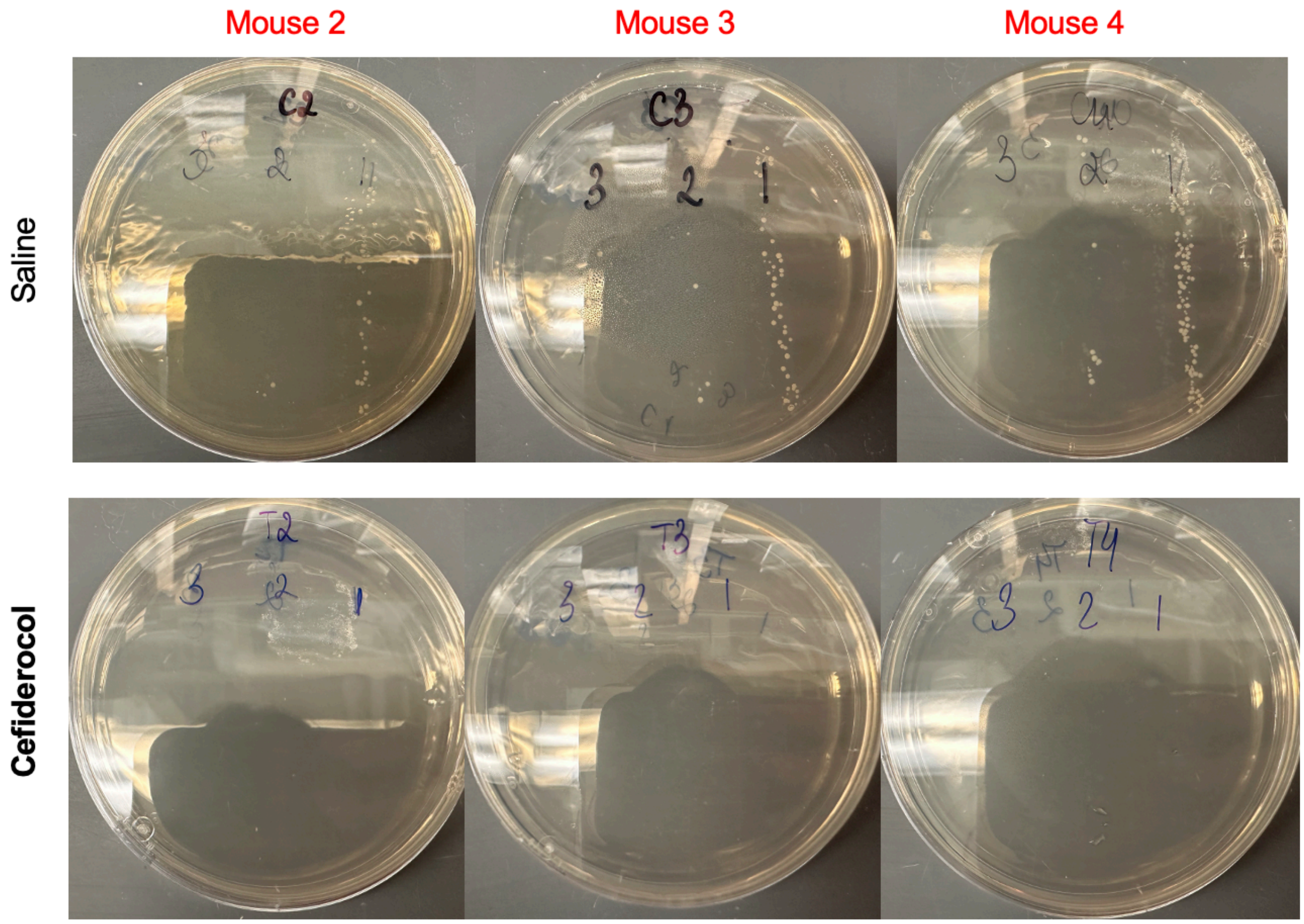

Figure S193. Growth of bacterial colonies on LB-agar plates inoculated with the homogenization products derived from *E. coli* Xen14 infected muscle tissue from saline and cefiderocol cohorts. Viable bacterial colonies are used to determined CFU/mL.

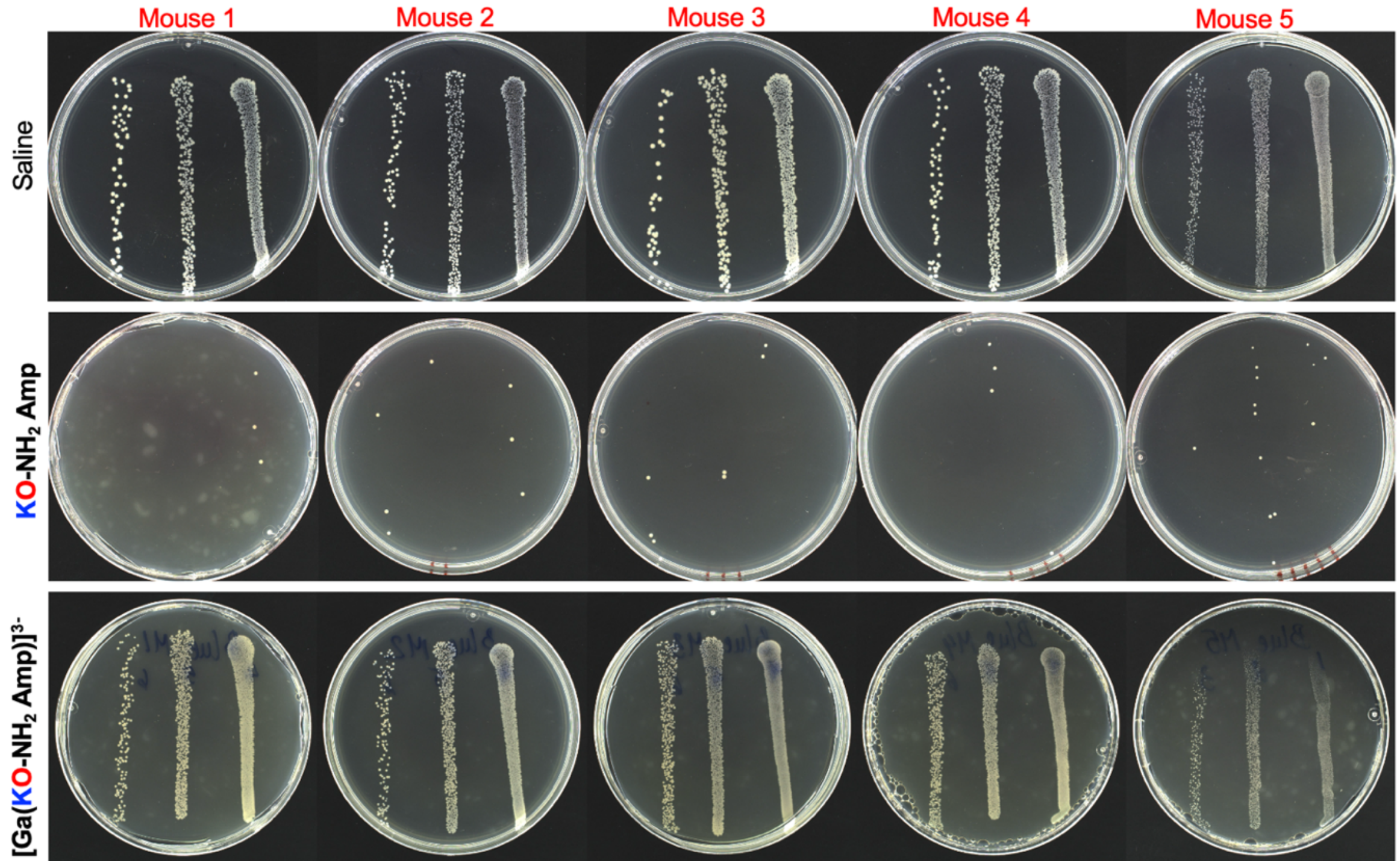

Figure S194. Growth of bacterial colonies on LB-agar plates inoculated with the homogenization products derived from *E. coli* Xen14 infected muscle tissue from saline, **KO-NH_2-Amp**, and **[Ga(KO-NH_2-Amp)]$^{3-}$** cohorts. Viable bacterial colonies are used to determined CFU/g.

Table S33. Tabulated CFU values for each cohort: saline (n = 5), cefiderocol (n = 4), **KO-NH_2-Amp** (n =5), and **[Ga(KO-NH_2-Amp)]$^{3-}$** (n = 5) after euthanizing the mice. No CFU were detected for cefiderocol cohort.

Mouse	Saline (CFU/mL)	**KO-NH_2-Amp** (CFU/mL)	**[Ga(KO-NH_2-Amp)]$^{3-}$** (CFU/mL)
1	2.75×10^8	0	6.75×10^8
2	4.05×10^8	1.50×10^7	6.35×10^8
3	1.60×10^8	1.50×10^7	1.78×10^9
4	3.00×10^8	0	1.69×10^9
5	1.67×10^9	5.00×10^6	7.80×10^8
Average	$5.62 \times 10^8 \pm 6.25 \times 10^8$	$7.00 \times 10^6 \pm 7.58 \times 10^6$	$1.11 \times 10^9 \pm 5.71 \times 10^8$

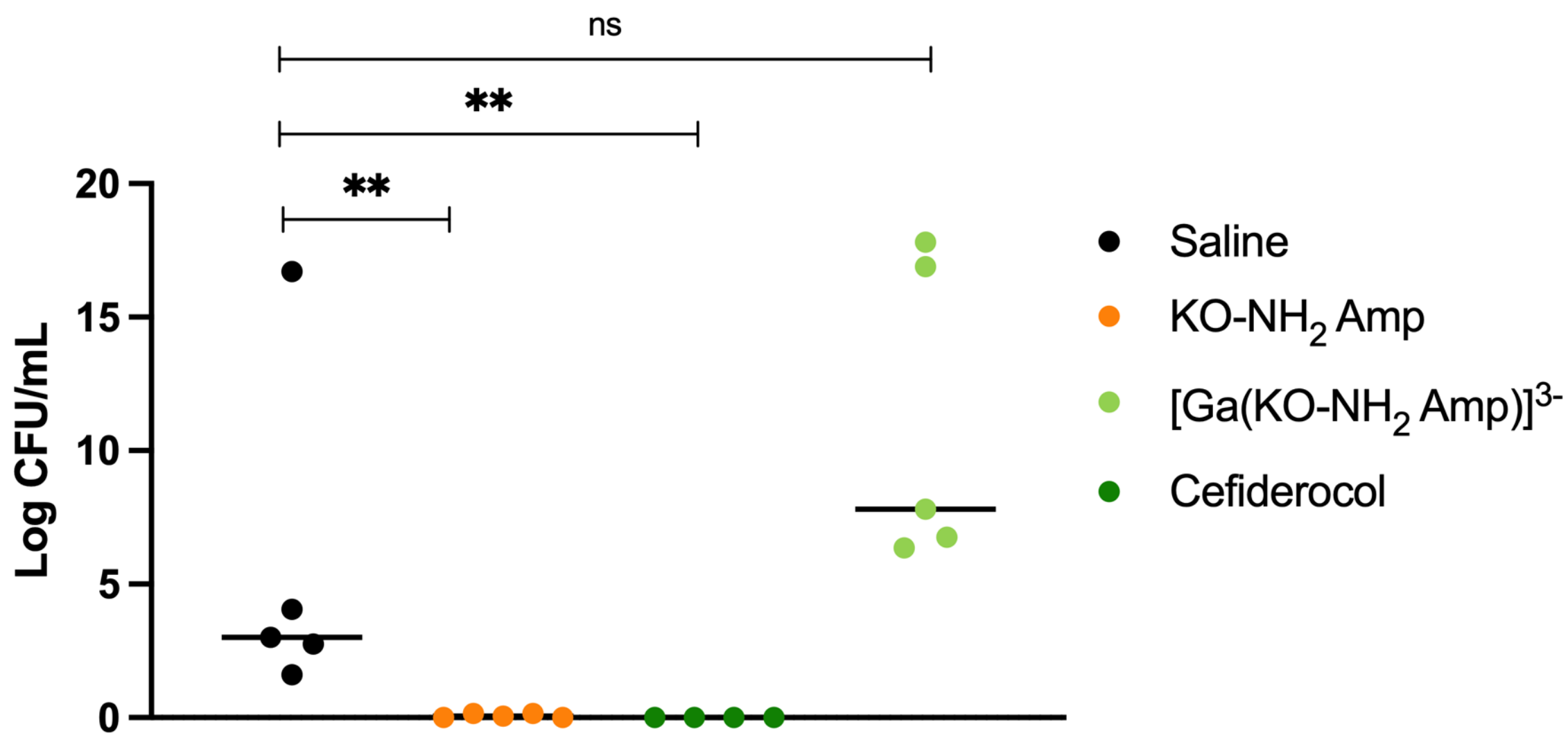

Figure S195. Quantification of CFU values in infected triceps for n = 4 for cefiderocol cohort and n = 5 for others. Mann-Whitney test was used to verify statistically significant between cohorts; **p value = 0.008 (saline vs KO-NH_2 Amp) and **p value = 0.007 (saline vs Cefiderocol).

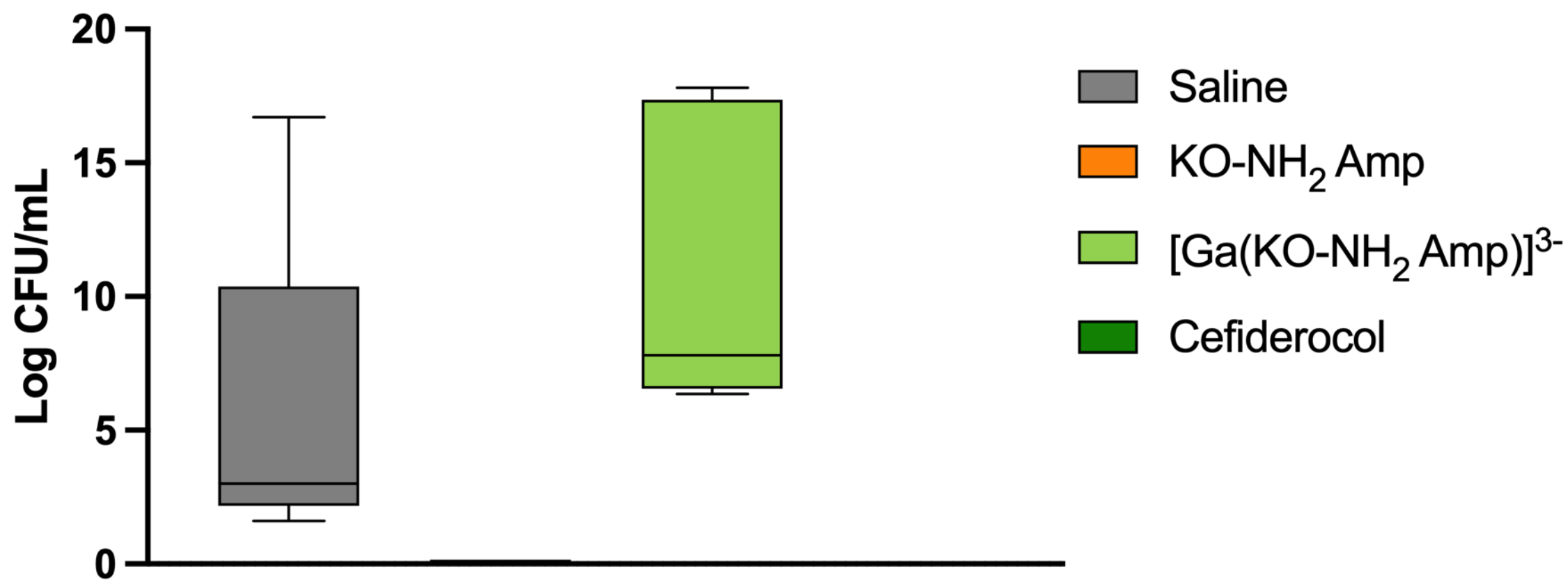

Figure S196. CFU values in infected triceps for n = 4 for cefiderocol cohort and n = 5 for others.

6.1.5 *Bioluminescence ROI analysis*

Table S34. Quantitative ROI analysis for infection burden over the time-course study. Unpaired t-test was used to validate statistically significant between cohorts; *p value = 0.022 (saline vs **KO-NH$_2$-Amp**), *p value = 0.047 (Cefiderocol vs **KO-NH$_2$-Amp**), **p value = 0.008 (saline vs Cefiderocol).

Time	Saline (×10^8) (ρ/sec/cm^2/sr)	Cefiderocol (×10^8) (ρ/sec/cm^2/sr)	**KO-NH$_2$-Amp** (×10^8) (ρ/sec/cm^2/sr)	**[Ga(KO-NH$_2$-Amp)]$^{3-}$** (×10^8) (ρ/sec/cm^2/sr)
0.5	1.98 ± 0.71	1.59 ± 0.55	1.48 ± 0.42	1.57 ± 0.77
1	6.72 ± 1.84	3.67 ± 1.22	4.00 ± 0.58	3.06 ± 1.06
2	37.20 ± 14.78	2.44 ± 0.33	5.88 ± 5.03	13.84 ± 3.99
3	54.70 ± 21.70	2.04 ± 0.27	11.50 ± 13.60	51.56 ± 17.98
4	51.00 ± 24.80	1.38 ± 0.37	11.10 ± 13.41	60.88 ± 24.74
5	50.00 ± 41.00	0.78 ± 0.26	7.39 ± 10.70	41.38 ± 21.31
6	31.00 ± 27.00	0.37 ± 0.13	4.10 ± 7.56	31.72 ± 13.17
8	8.80 ± 7.47	0.16 ± 0.05	0.30 ± 0.57	12.67 ± 7.34
10	3.80 ± 5.70	0.0096 ± 0.0050	0.031 ± 0.06	11.19 ± 8.65
12	0.67 ± 0.36	0.0030 ± 0.0018	0.0077 ± 0.01	6.72 ± 7.80
24	0.047 ± 0.03	0.00048 ± 0.00041	0.0038 ± 0.01	1.00 ± 1.19
48	0.019 ± 0.02	0.00018 ± 0.000067	0.0016 ± 0.00	11.12 ± 8.69
72	0.014 ± 0.02	0.0000022 ± 0.000016	0.00054 ± 0.00	0.10 ± 0.078

7. References

(1) Gardner, R. A.; Kinkade, R.; Wang, C.; Phanstiel, O. t. Total synthesis of petrobactin and its homologues as potential growth stimuli for Marinobacter hydrocarbonoclasticus, an oil-degrading bacteria. *J Org Chem* **2004**, *69* (10), 3530-3537. DOI: 10.1021/jo049803l From NLM.

(2) Motz, R. N.; Guo, C.; Sargun, A.; Walker, G. T.; Sassone-Corsi, M.; Raffatellu, M.; Nolan, E. M. Conjugation to Native and Nonnative Triscatecholate Siderophores Enhances Delivery and Antibacterial Activity of a β-Lactam to Gram-Negative Bacterial Pathogens. *J Am Chem Soc* **2024**, *146* (11), 7708-7722. DOI: 10.1021/jacs.3c14490 From NLM.

(3) Rodgers, S. J.; Lee, C. W.; Ng, C. Y.; Raymond, K. N. Ferric Ion Sequestering Agents. 15. Synthesis, Solution Chemistry, and Electrochemistry of a New Cationic Analogue of Enterobactin. *Inorganic Chemistry* **1987**, *26*, 1622-1625.

(4) Neese, F. The ORCA program system. *WIREs Computational Molecular Science* **2012**, *2* (1), 73-78. DOI: https://doi.org/10.1002/wcms.81 (acccessed 2026/04/14).

(5) Yanai, T.; Tew, D.; Handy, N. A new hybrid exchange-correlation functional using the Coulomb-Attenuating Method (CAM-B3LYP). *Chemical Physics Letters* **2004**, *393*, 51-57. DOI: 10.1016/j.cplett.2004.06.011.

(6) Grimme, S.; Ehrlich, S.; Goerigk, L. Effect of the damping function in dispersion corrected density functional theory. *Journal of Computational Chemistry* **2011**, *32* (7), 1456-1465. DOI: https://doi.org/10.1002/jcc.21759 (acccessed 2026/04/14).

(7) Weigend, F.; Ahlrichs, R. Balanced basis sets of split valence, triple zeta valence and quadruple zeta valence quality for H to Rn: Design and assessment of accuracy. *Physical Chemistry Chemical Physics* **2005**, *7* (18), 3297-3305, 10.1039/B508541A. DOI: 10.1039/B508541A.

(8) Johnstone, T. C.; Nolan, E. M. Beyond iron: non-classical biological functions of bacterial siderophores. *Dalton Transactions* **2015**, *44* (14), 6320-6339, 10.1039/C4DT03559C. DOI: 10.1039/C4DT03559C.
